# Identification of the Putative Binding Site of a Benzimidazole Opioid (Etazene) and Its Metabolites at µ-Opioid Receptor: A Human Liver Microsomal Assay and Systematic Computational Study

**DOI:** 10.3390/molecules28041601

**Published:** 2023-02-07

**Authors:** Krishna Chaturvedi, Isuru Hewamanna, Pankaj Pandey, Washim Khan, Yan-Hong Wang, Amar G. Chittiboyina, Robert J. Doerksen, Murrell Godfrey

**Affiliations:** 1 Department of Chemistry and Biochemistry, University of Mississippi, University, MS 38677, USA; 2 National Center for Natural Products Research, University of Mississippi, University, MS 38677, USA; 3 Division of Medicinal Chemistry, Department of BioMolecular Sciences, School of Pharmacy, University of Mississippi, University, MS 38677, USA

**Keywords:** synthetic opioids, etazene, docking, molecular dynamics, µ-opioid receptor, liver microsomal assay

## Abstract

The synthetic benzimidazole opioid etazene (which has a 70-times higher analgesic activity than morphine), a recreational drug, has gained popularity as a novel psychoactive substance (NPS) on the illegal/darknet market; however, no experimental information is available at the molecular level on the binding mechanism and putative binding site of etazene and its metabolites at the µ-opioid receptor (MOR). In the present study, we investigated the metabolism of etazene in human liver microsomes using ultra-high-performance liquid chromatography–mass spectrometry (UHPLC–MS). We also explored the possibilities of MOR activation by etazene and its metabolites by studying their binding mechanisms and interaction profiles at an active-state MOR model via molecular docking, binding free energy calculations, and all-atom molecular dynamics (MD) simulations. The putative metabolites of etazene were also predicted using the ADMET Predictor 10.1. The molecular docking studies and free energy calculations showed that etazene and its metabolites (M1, M2, and M5–M7) exhibited strong predicted binding affinity at MOR and showed overlapped binding orientation with MOR-bound agonist BU72, which was co-crystallized in the MOR X-ray crystal structure (PDB ID: 5C1M). MD also confirmed the stability of the MOR–etazene and MOR–M6 complexes. These results suggest that etazene and its metabolites may act as strong MOR agonists, highlighting the necessity of experimental validation. The insights from this study, such as key interactions between etazene and its metabolites and the MOR, will allow authorities to predict potential analogs and clarify the target–protein interactions associated with this illicit substance, granting advanced or rapid reactions to confiscating or banning potential emerging drugs.

## 1. Introduction

Among recreational users and regular abusers, novel psychoactive substances (NPSs) continue to rise in popularity. According to a report from the European Monitoring Centre for Drugs and Addiction (EMCDDA) [1], etazene appears to be the most popular opioid drug in the darknet market. The synthesis and properties of benzimidazole opioids were initially published between 1957 and 1961 [2,3,4,5,6,7,8], indicating the possibility of these opioids being used as painkillers. Hungar et al. estimated that etazene’s analgesic properties are around 70-fold stronger than morphine [4]. Originally discovered in the illegal drug market of Poland during the 1950s, etazene did not reappear on the market until 2019. Despite the early appearance of etazene, until recently, information regarding etazene was restricted to databases such as TG Chemicals [9]. Etazene has been reported to interact with various receptors, such as the µ-opioid receptor (MOR), serotonin receptors, dopamine receptors, and cannabinoid receptors.

The growing popularity associated with synthetic opioids (SOs) is widely concerning due to their potential adverse effects on the human body, with the most severe symptoms starting with depression and leading to respiratory arrest and death. In addition, SOs can cause tactile and visual hallucinations by inducing specific perception disorders. However, the real danger with NPSs is the low effective dose that can cause narcotic effects and lead to a high risk of overdose [10]. The CDC has reported that this opioid overdose risk led to over 50,000 deaths in 2019 [11].

2-[(4-ethoxyphenyl)methyl]-*N, N*-diethyl-1*H*-benzimidazole-1-ethanamine, or etazene, is a 2-benzylbenzimidazole with a similar structure to those of etonitazene and clonitazene, substances internationally controlled as narcotics. Etazene is readily available as a liquid or a prepared nasal spray formulation for sale as a reference substance in online stores, where it is marketed as “similar to fentanyl but safer.” Abuse dosages of etazene begin from 0.5 to 1 mg and can be administered by oral or intranasal routes [12]. Sleepiness was reported with doses from 1.5 to 3 mg, and in previous studies, Hungar et al. [4] have assessed the analgesic properties and determined that the effect is caused by the activation of the MOR in the central nervous system, with the after-effects leading to an alleviation of pain, relaxation, euphoria, bradycardia, decrease in body temperature, and decrease in breathing, which can cause a serious health risk with an increased dosage.

It has been established that many compounds that are not inherently toxic can become harmful after they have been metabolized in vivo due to the appearance of their active metabolites. Biotransformation of exogenous compounds, such as SOs, in vivo may occur through a series of phases to yield products that are inactive, active, or toxic [13,14]. In Phase I metabolism, a polar, water-soluble metabolite that remains active is often generated after oxidation of the parent compound by cytochrome P450 (CYP450) [13]. As the liver is abundant in heme-containing CYP450 enzymes, the use of liver microsomes can help reveal downstream characteristics and the process of metabolism of exogenous compounds under study [15]. Furthermore, liver microsomes also harbor advantages such as low cost, simplicity of use, and retention of enzyme activity in long-term storage [15,16]. To quantify the downstream metabolic products of SOs, high-performance liquid chromatography (HPLC) with mass spectrometry can be employed due to the technique’s inherent specificity, increased sensitivity, and higher throughput [17] compared to other methods of chromatography. The first report on etazene metabolites in urine was published in 2022 by Grigoryev et al. [18]. Using gas chromatography–mass spectrometry (GC–MS) and liquid chromatography–mass spectrometry (LC–MS) techniques, eight etazene metabolites were found in rat urine and serum by Grigoryev et al. [18]. These were tentatively identified as products of N-deethylation, O-deethylation, hydroxylation, or N-oxidation of the benzimidazole moiety and combinations of these processes. 

Computational modeling is an appropriate method for predicting downstream metabolites of drug compounds, including SOs, and is also frequently used to predict protein–ligand interactions. Implementing computational modeling allows the researcher to predict structural modifications to SOs by human metabolic processes and predict advanced chemical properties of drug-like molecules. A multitude of algorithms are available to advance work beyond structure prediction and include modeling with a selected protein structure, yielding modes, dynamics, and strengths of the interaction of protein–ligand complexes and predicted metabolite ligands. These approaches can help to limit the need for animal or human testing in the preliminary stages of investigations [19].

**Figure 1:**
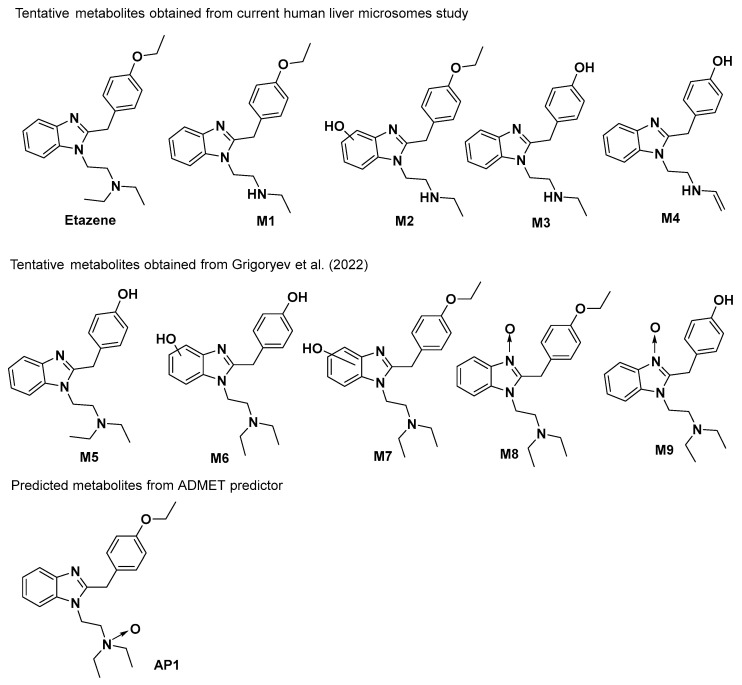
Possible metabolites of etazene were found in the human liver microsomal assay and rat serum and urine [18] and predicted by the ADMET predictor v10.0.0.11 (Simulation Plus, Lancaster, CA, USA).

Given the rising popularity of etazene and the lack of available experimental information on the molecular level, in this work, we set out to perform a rigorous examination of etazene’s mechanism of binding to target receptors to help understand the pharmacology of the SOs, as well as to uncover etazene’s metabolites and their associated potential for abuse. Therefore, this study investigated the possible metabolites of etazene using the in vitro human liver microsomal assay. In addition, extensive protein–ligand interaction profiles of etazene and its metabolites against the µ-opioid receptor (MOR) were studied using docking, binding free energy calculations, and molecular dynamics simulations (Figure 1). Finally, this study also explored the impact of specific structural characteristics on the binding of etazene and its metabolites and their putative binding mechanism at the MOR.

## 2. Results and Discussion

### 2.1. In Vitro Liver Microsomal Assay and Tentative Metabolite Identification

Etazene was subjected to in vitro human liver microsomal metabolism, involving incubation of the compound with microsomes for 120 min at 37 °C in the presence of NADP and glucose-6-phosphate dehydrogenase (G6PD). In this work, etazene was incubated with microsomes for 120 min, while monitoring the metabolism at 15-min intervals to assess the enzymatic activity and thermal degradation of the drug. At each interval, some of the reaction mixture was extracted and further analyzed by UHPLC–MS.

**Figure 2:**
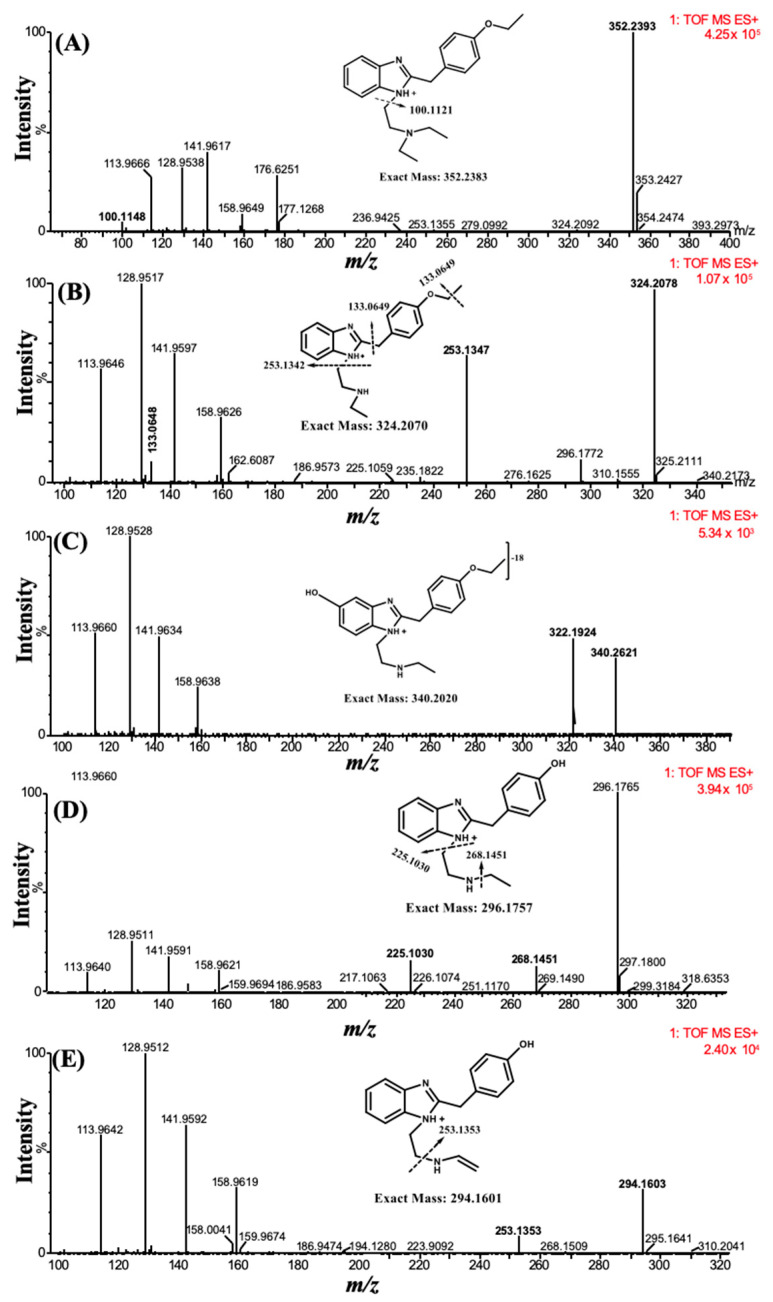
High-resolution mass spectra, their corresponding structure, and fragmentation pattern of (**A**) etazene, (**B**) M1, (**C**) M2, (**D**) M3, and (**E**) M4. All metabolites were tentatively identified based on high-resolution MS and MS/MS of key fragments.

The parent compound and its metabolites formed after incubation with the human liver microsomes (HLM) were characterized through MS and MS/MS spectra. A total of four metabolites were tentatively identified based on high-resolution mass spectra and MS/MS fragmentation patterns. The first metabolite [M1] (*m/z* 324.2078) was formed through deethylation of the ‘N’ terminal, which was detected at t_R_ 5.41 min. The second metabolite [M2], with an *m/z* of 340.2621 at t_R_ 4.71 min was the product of the parent compound via deethylation of the ‘N’ terminal and hydroxylation. The third metabolite [M3], characterized at an *m/z* 296.1765, was detected at t_R_ 2.72 min and formed through deethylation of both ‘N’ and ‘O’ terminals. Similarly, the fourth metabolite [M4] with an *m/z* 294.1603 was eluted at t_R_ 5.92 min and was formed by deethylation of the ‘N’ and ‘O’ terminals, followed by hydroxylation and desaturation of the ‘N’ terminal ethyl group (Figure 2).

Quantitative analyses of the parent compound (etazene) and positive control (THC) were also carried out with UHPLC–MS. The results of the incubation of etazene with HLM indicated that more than 90% of the parent compound was converted within 30 min of incubation, and it followed a similar pattern to that of the positive control [20].

Grigoryev et al. [18] reported eight tentative metabolites (M1–M3) and (M5–M9) of etazene (Figure 1) in rat serum and urine by GC–MS and high-resolution accurate mass (HRAM) LC–MS. They tentatively identified these metabolites as products of N-deethylation, O-deethylation, hydroxylation, or N-oxidation of the benzimidazole moiety or a combination of these reactions. However, our in vitro HLM assay by UHPLC–MS only identified four metabolites (M1–M4), wherein M4 metabolite was reported for the first time. The first three metabolites (M1–M3) were also reported by Grigoryev et al.; however, the use of in vitro HLM assay is reported for the first time.

### 2.2. Molecular Docking Studies

Molecular docking studies were performed to comprehend the putative binding pose and orientation of etazene and M6 in the active site of the MOR protein crystal structure using XP Glide docking. The docking protocol was verified by redocking the co-crystalized ligand to check the quality of the approach. The docking protocol was validated by self-docking (re-docking), in which the native ligand, BU72, was docked into the human MOR. We calculated the RMSD between the docked pose and the experimental pose of the native ligand with the human MOR. The superimposition of the experimental pose of the native ligand with the docked pose showed a nearly identical conformation with a very small RMSD of 0.22 Å (Supplementary Materials, Figure S1). Following the docking process of all ligands, the most favorable poses for the ligands were selected based on the individual GlideScore and Glide Emodel values. In this manuscript, we have frequently used the following scoring functions to describe our findings on the interactions profile. Emodel score [a mathematical combination of the GlideScore, the ligand strain (Einternal), and the Coulomb and van der Waals energies] was used to determine the best pose among the multiple poses of each ligand. However, GlideScore itself was used to rank the best poses against one another to choose the starting point for the Prime MM-GBSA binding free energy calculations followed by molecular dynamics (MD) simulations. Increasingly negative values indicated better binding. Subsequently, the best poses for each ligand were used to calculate their binding free energies (Prime MM-GBSA). The docking results revealed that etazene and most of its metabolites interacted with Asp147 and Tyr148 (Table 1), which are critical residues for binding to the MOR. The interaction profiles of all the metabolites are presented in Table 1. M1, M2, and M5–M8 metabolites of etazene (Table 1) possessed more negative GlideScore and binding free energy values compared to those of the rest of the metabolites and therefore are the most promising compounds. Amongst them, M6 has the best GlideScore and ΔG values; thus, it was selected for further study through MD simulations. The binding free energy data and overlapped binding orientations with the MOR support a strong predicted binding affinity of the metabolites.

**Figure 3:**
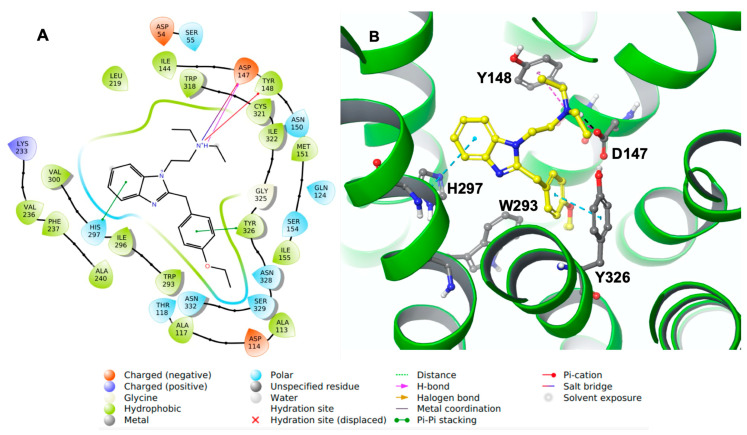
(**A**) The two-dimensional (2D) and (**B**) three-dimensional (3D) interaction diagram of etazene with MOR. The key residues are shown in the ball and stick model (carbon in gray), and transmembrane regions are shown as ribbons (green-colored). Etazene is shown in a ball and stick model (carbon in yellow). The distance between H297 and benzimidazole moiety (edge to face; π–π stacking): 4.7 Å and between Y326 and ethoxy phenyl (face to face; π–π stacking): 3.75 Å. The H-bond and salt bridge distances are 1.95 Å and 2.90 Å, respectively.

Docking results showed that etazene formed π–π stacking interactions with His297 and Tyr326 (Figure 3) of the MOR. In addition, the N^+^H group of etazene formed a cation–π bond with Tyr148.

**Figure 4:**
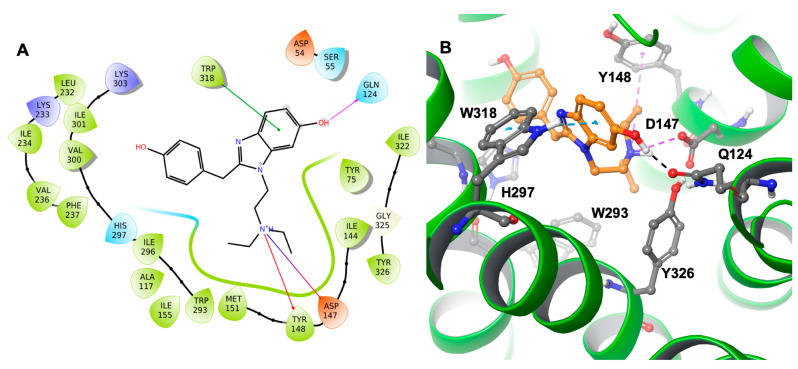
(**A**) The two-dimensional (2D) and (**B**) three-dimensional (3D) interaction diagram of the M6 metabolite with MOR. The key residues are shown in the ball and stick model (carbon in gray), and transmembrane regions are shown as ribbons (green-colored). M6 is shown in the ball and stick model (carbon in orange). The distance between W318 and benzimidazole moiety (edge to face; π–π stacking) is 4.7 Å. The H-bond, salt bridge, and cation–π distances are 1.85 Å, 3.09 Å, and 5.97 Å, respectively.

Asp147 was also involved with the N^+^H group through hydrogen bonding and a salt bridge. This type of interaction is prevalent in opioids and opiates within the sphere of psychoactive substances. In addition, the ligand–receptor complex of the M6 metabolite (Figure 4) showed π–π stacking with Trp318. Similar to etazene, Asp147 formed a salt bridge with the N^+^H group of M6 without forming an additional hydrogen bond. Instead, it exhibited a hydrogen bond between Gln124 and a hydroxyl group of M6. Lastly, Tyr148 showed a cation–π interaction with the N^+^H group. Interestingly, M6 did not exhibit a π–π stacking interaction with His297, which was observed in the MOR–etazene docked pose.

### 2.3. ADMET-Predicted Metabolites

ADMET predictor indicated four possible metabolites of etazene. Upon comparison to the metabolites found in the human liver microsomal study and in rat serum and urine studies, a few metabolites (M1–M2 and M5) were found to be structurally identical (Figure 1). The pharmaceutically important physicochemical properties of etazene and its metabolites, including lipophilicity, solubility, and hERG (human ether-a-go-go-related gene) K+ channel-blocking properties, were calculated using the ADMET predictor 10.1. The calculated logP and logS values of etazene and its metabolites predict that they possess good aqueous solubility and low lipophilicity and follow the Lipinski’s Rule of Five. Etazene and its metabolites (M1–M3 and M5–M8) are likely to block the hERG K+ channel. Blockage of these channels in the heart cells can lead to fatal cardiac toxicity (Table 2).

### 2.4. Molecular Dynamics Simulations

**Figure 5:**
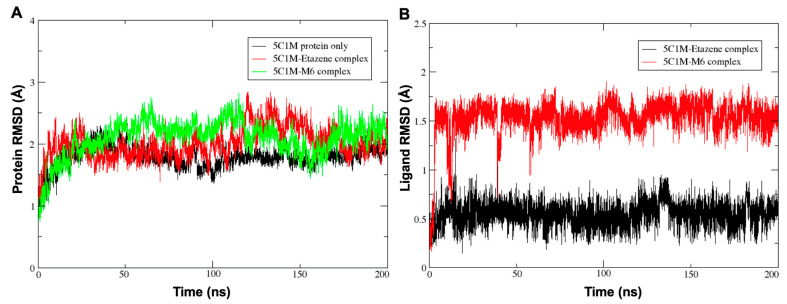
(**A**) The protein RMSD plot for Cα atoms of the protein MOR (PDB ID: 5C1M) only and MOR–etazene and MOR–M6 complexes; and (**B**) the ligand RMSD plot for ligand–heavy atoms for MOR–etazene and MOR–M6 complexes (PDB ID: 5C1M) for the reference frame at 0 ns.

Since the docking method generates a single snapshot of the protein–ligand interaction with sometimes questionable accuracy, molecular dynamics (MD) simulation is the best method for understanding the protein–ligand interactions involved by considering simulated parameters that are similar to human physiological conditions. MD simulates the natural motion of the molecular system and is used to analyze the physical movements of atoms, including proteins and molecules, under physiological conditions. The stabilities of the MOR–etazene and MOR–M6 complexes were evaluated using the Desmond software at 300 K for 200 ns. The root-mean-square deviation (RMSD) plot of atom locations vs. simulation time (Figure 5) confirms the stability of the structure of the protein and indicates whether the simulation equilibrated or not. The RMSD analyses of the protein Cα atoms (average RMSD (MOR protein only) = 1.79 Å; average RMSD (MOR–etazene complex) = 2.01 Å; average RMSD (MOR–M6 complex) = 2.09 Å) suggested that the protein MOR reached equilibration after ∼40 ns and remained on a plateau throughout each 200-ns simulation. Low RMSD values suggest that the equilibrium structure was somewhat close to the starting structure. Similarly, the RMSD values of the ligand’s heavy atoms for MOR–etazene (average RMSD = 0.55 Å) and MOR–M6 (average RMSD = 1.23 Å) were very stable throughout the 200-ns simulation, indicating that the starting conformation of the ligand did not change significantly throughout the simulation.

**Figure 6:**
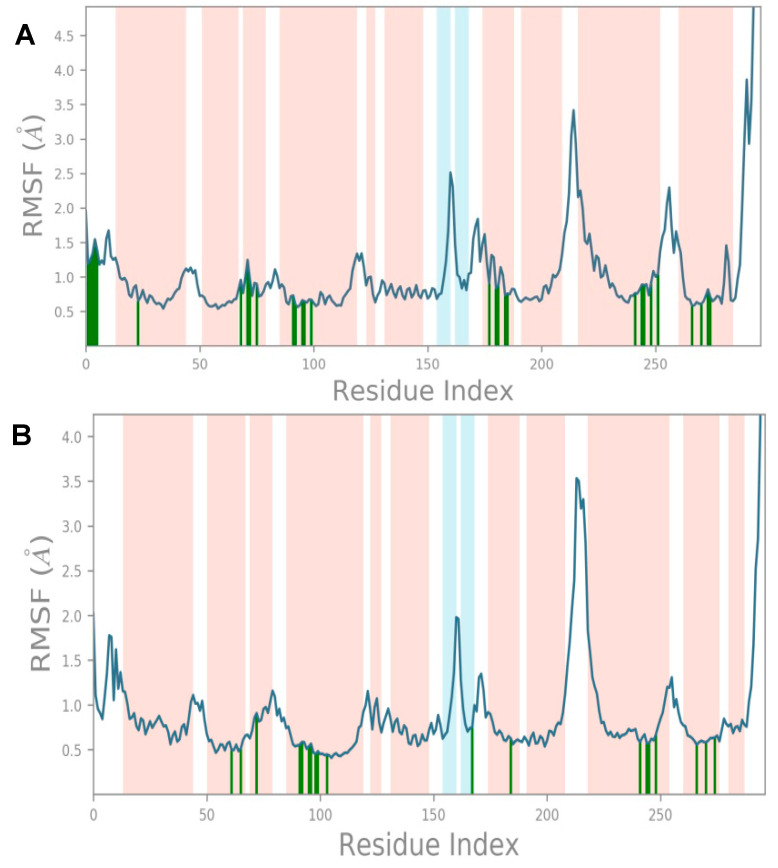
(**A**) The protein RMSF plot for etazene and (**B**) M6 with MOR complex (PDB ID: 5C1M) relative to the reference frame at 0 ns. The green-colored vertical bars on the bottom of the plot represent protein residues that have interacted with the ligand.

As mentioned previously, the root-mean-square fluctuation (RMSF) plot characterizes local changes along the protein chain. The peaks on the plot include the protein residues that more frequently fluctuate during the simulation (Figure 6). Typically, the protein’s termini (N- and C-terminal) fluctuate more than any other protein part. Secondary structure elements like α-helices and β-strands, which are highlighted in orange and cyan backgrounds, respectively, are usually more rigid than the unstructured part of the protein and thus fluctuate less than the loop regions (white background). The RMSF based on the Cα atoms of the MOR with MOR–etazene and MOR–M6 complexes showed very low fluctuations for the residues that form the ligand–binding site. The average RMSF values were observed to be 0.89 Å (MOR–etazene complex) and 1.042 Å (MOR–M6) (Figure 6), supporting the stability of the complex. The high fluctuations are depicted as the places where the blue line reaches high RMSF values. Those mostly correspond to the loop regions, which are depicted as white regions.

**Figure 7:**
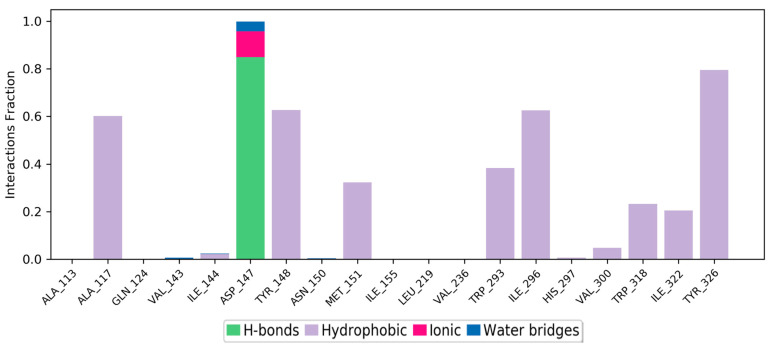
Simulation interaction diagram (SID) plot showing the protein–ligand interactions between the binding site and etazene. The stacked bar charts include the fraction of occupancy, among all of the MD simulation snapshots.

**Figure 8:**
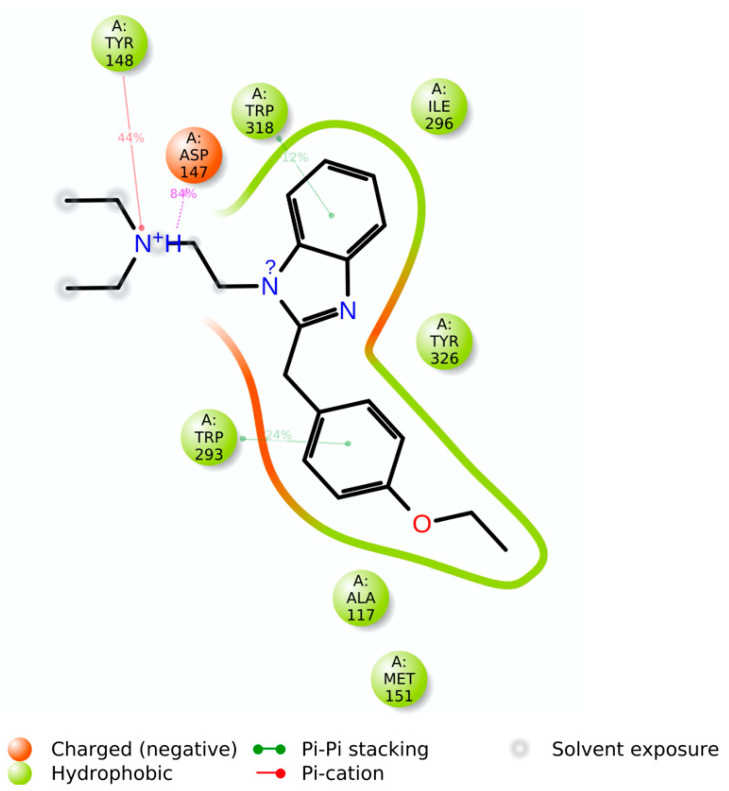
2D interaction diagram showing detailed etazene–atom interactions with key protein residues during the 200-ns MD simulation.

The simulation interaction histogram (Figure 7) and 2D contact map (Figure 8) of etazene showed that Asp147 (hydrogen bonds, ionic interactions, and water bridges, 84% contribution), Tyr148 (hydrophobic, 44% contribution), Trp293 (hydrophobic, 24% contribution), and Trp318 (hydrophobic, 12% contribution) were the crucial amino acids for the interaction with the MOR.

Footnotes: The stacked bar charts are normalized over the course of the trajectory; for example, a value of 0.8 suggests that for 80% of the simulation time, the specific interaction is maintained. Values over 1.0 are possible as some protein residues may make multiple contacts of the same subtype with the ligand.

**Hydrogen bonds**: The geometric criteria for a protein–ligand H-bond are: a distance of ≤2.5 Å between the donor and acceptor atoms (D—H···A); a donor angle of ~120° in the donor–hydrogen–acceptor atoms (D—H···A); and an acceptor angle of ~90° in the hydrogen–acceptor–bonded-atom atoms (H···A—X).

**Hydrophobic contacts**: The geometric criteria for hydrophobic interactions are as follows: cation–π, aromatic and charged groups within 4.5 Å; π–π, two aromatic groups stacked face-to-face or face-to-edge within 5.5 Å; and other, non-specific hydrophobic sidechain within 3.6 Å of a ligand’s aromatic or aliphatic carbons.

**Ionic interactions or polar interactions**: They are between two oppositely charged atoms that are within 3.7 Å of each other and do not involve a hydrogen bond.

**Water bridges**: The geometric criteria for a protein–water or water–ligand H-bond are: a distance of ≤2.8 Å between the donor and acceptor atoms (D—H···A); a donor angle of ~110° in the donor–hydrogen–acceptor atoms (D—H···A); and an acceptor angle of ~90° in the hydrogen–acceptor–bonded-atom atoms (H···A—X).

**Figure 9:**
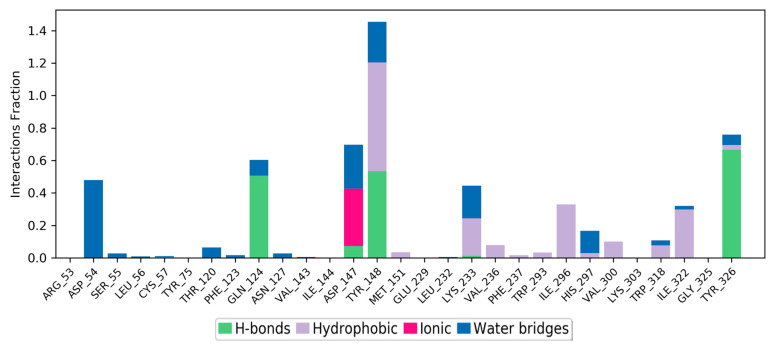
Simulation interaction diagram (SID) plot showing the protein–ligand interactions between the binding site and the MOR–M6 complex. Stacked bar charts include the fraction of occupancy among all of the MD simulation snapshots.

**Figure 10:**
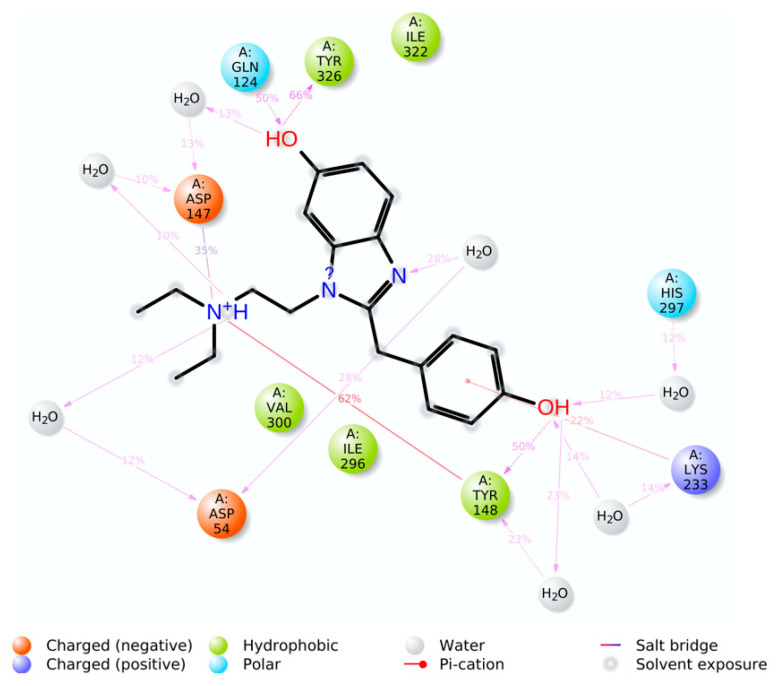
A two-dimensional interaction diagram of the detailed MOR–M6 complex atom interactions with key protein residues during the 200-ns MD simulation.

The simulation interaction histogram (Figure 9) and 2D contact map (Figure 10) of metabolite M6 showed that Asp54 (water bridges, 40% contribution), Gln124 (hydrogen bonds and water bridges, 50% contribution), Asp147 (hydrogen bonds, ionic interactions, and water bridges, 35% contribution), Tyr148 (hydrogen bonds, hydrophobic, and water bridges, 62% contribution), Lys233 (hydrophobic and water bridges, 22% contribution), and Tyr326 (hydrogen bonds and water bridges, 66% contribution) of the MOR–M6 complex were the crucial amino acids for the interaction with the MOR. The predicted strong binding interactions of M6 with the MOR indicate that M6 may behave as a potential MOR agonist during in vitro experimental validation.

## 3. Materials and Methods

### 3.1. Chemicals and Reagents

Human liver microsomes (Corning^®^ Gentest™ HLM, ~20-Donor Pool, Mixed Gender; catalog# 452161) were obtained from Corning^®^, Glendale, AR, USA. Glucose-6-phosphate dehydrogenase (G6PD), glucose-6-phosphate, NADP+, potassium phosphate monobasic, potassium phosphate dibasic, and MgCl_2_ were purchased from Sigma Chemical (St. Louis, MO, USA). Etazene and Δ^9^-tetrahydrocannabinol (Δ^9^-THC) were acquired from Cayman Chemical (Ann Arbor, MI, USA). HPLC grade acetonitrile and methanol were obtained from Thermo Fisher Scientific (Waltham, MA, USA).

### 3.2. Human Liver Microsomal Assay Protocol

Oxidative metabolism via cytochrome P450 was carried out in a total volume of 3 mL using pooled human liver microsomes as described by ElSohly et al. [21]. Microsomes (0.5 mg/mL) were pre-incubated at 37 °C for 5 min with etazene (3 µM), MgCl_2_ (10 mM), glucose-6-phosphate (20 mM), and G-6-PDH (2 U/mL) in 100 mM potassium phosphate buffer (pH 7.4). The reaction was initiated by adding NADP+ (1 mM) to begin oxidative metabolism. A total of nine samples, each consisting of 100 μL of the solution, were collected from 0 h until 2 h at intervals of 15 min at specified time points (0, 15, 30, 45, 60, 75, 90, 105, and 120 min). The reaction was terminated by adding 200 µL of ice-cold methanol, and the collected samples were vortexed for 2 min. After vortexing, the samples were incubated at −60 °C for 1 h, then centrifuged at 12,000 rpm for 15 min at 4 °C. The clear supernatant was collected and analyzed using UHPLC–MS, with Δ^9^-tetrahydrocannabinol (THC) as the positive control for the experiment. The negative control consisted of all the components present in the positive controls except the liver microsomes. Since the negative control did not contain liver microsomes, no response in terms of the metabolism of the test compound was expected as a comparison with the test group. For the positive control of THC in this experiment, methanol was used as the vehicle. Other compounds in this experiment utilized DMSO as the vehicle. Solvent control was also processed similarly.

### 3.3. UHPLC–QToF MS System

Analysis was carried out with a Waters Ultra-High-Performance Liquid Chromatography (Waters Acquity UPLC^TM^ system, Waters Corp., Milford, MA, USA) connected with a Xevo-G2-S QToF high-resolution mass spectrometer. Chromatographic separation was performed using a Waters Acquity UPLC BEH C18 column (1.7 µm, 2.1 × 100 mm) (Waters, USA). The solvent system was composed of acetonitrile with 0.05% formic acid (A) and water with 0.05% formic acid (B). Gradient elution at a flow rate of 0.25 mL/min was followed throughout the complete separation as follows: the initial solvent condition was 2% A; increased to 30% A over 7 min; further increased to 55% A over 10 min, and finally ramped to 100% A over 16 min and maintained up to 19 min. The total run time was 23 min, including four minutes for re-equilibrium with the initial condition. The separated compounds were detected by a quadrupole time-of-flight (QToF) mass spectrometer in positive ionization mode. Capillary voltage, cone voltage, source temperature, desolvation temperature, cone gas flow, and desolvation gas flow were 1.2 kV, 30 V, 80 °C, 400 °C, 50 L/h, and 800 L/h, respectively. Data were collected in centroid mode and processed through MassLynx™ NT 4.1 software. During the analysis, parent and fragment ions of leucine-enkephalin were used as lock mass in MS/MS mode (*m/z* 556.2771 and 278.1141) and were monitored to ensure mass accuracy.

### 3.4. Computational Study

#### 3.4.1. Metabolite Selection

All tentative metabolites of etazene were collected from the current in vitro human liver microsomal assay, the literature [13], and by using the ADMET predictor v10.0.0.11 (ADMET Predictor. Lancaster, CA, USA: Simulations Plus, Inc.; 2021. https://www.simulations-plus.com (accessed on 16 March 2021). The metabolites chosen include M1–M9 and AP-1 (Figure 1). In addition, Grigoryev et al. [18] also reported the tentative metabolites (M1–M3) in addition to M5–M9 in their study. Furthermore, ADMET also predicted metabolites (M1–M2 and M5) in addition to AP1. The main physicochemical properties of etazene and its metabolites, including lipophilicity and solubility, were calculated using the ADMET predictor.

#### 3.4.2. Protein Selection and Preparation

The active-state X-ray crystal structure of the MOR (PDB ID: 5C1M) [22] was downloaded from the Protein Data Bank. Any issues involving the protein X-ray structure, such as missing residues or hydrogens, misoriented structural groups, and incomplete side chains or loops, were corrected using the Protein Preparation Wizard module implemented in the Schrödinger software (Schrödinger Release 2020-4, Maestro, Schrödinger, LLC, New York, NY, USA). We used the Prime module of the Schrödinger software suite to remodel the protein for some residues (Lys269, Glu270, and Arg273) that had missing side chains. We also mutated residues 53, 54, 57, and 59 (N-terminal residues) from 5C1M to wild-type human MOR which forms a lid over the ligand-binding pocket. These N-terminus residues were refined using the Prime module. This preparation process helped to ensure that the most accurate protein structure was used to dock each ligand. The OPLS3e force field was used during the protein preparation steps. Finally, the molecule etazene and its tentative metabolites were prepared at a pH of 7.4, and the 3D minimized structures were generated using the OPLS3e force field.

#### 3.4.3. Grid Generation

The grid generation step was used to prepare and determine the binding pocket of the MOR for docking. The centroid of the morphinan derivative, BU72, a co-crystallized ligand in the binding pocket of the MOR, was used to create the grid and determine where the binding pocket region should be specified. The box was centered on the co-crystallized ligand as a cube of side length 10 Å.

#### 3.4.4. Glide Docking

The chosen ligands (etazene, M1–M9, and AP1) were docked using the extra precision (XP) [23] method implemented in the Glide module of the Schrödinger software (Schrödinger 2020- Release 2020-4). In our docking study, we used Glide’s default flexible docking approach in which the protein was considered as a rigid entity, and the ligand was considered flexible. No additional constraints were used on the present binding pocket residues of the receptor during the Glide docking process. A total of 5 poses per ligand were retained, with a threshold for rejecting any minimized pose set at 0.50 kcal/mol. The best pose of each compound was selected based on Emodel and GlideScores. Visual inspection of each scored pose in combination with the crystal structure of the receptor aided in the determination of the best overall binding pose for each ligand. The GlideScores maximize the separation of the compounds with a strong binding affinity from those with little to no binding affinity [24,25]. As an empirical scoring function, the value incorporates the physics of the binding process, which includes the following terms: rotatable bond penalty, hydrogen bond terms, protein–ligand energy contributions, hydrophobic enclosure, and lipophilic–lipophilic term. Conversely, the calculated Glide Emodel score emphasizes the force field components of the binding, such as electrostatic energies and van der Waals forces. Glide Emodel scores compare and rank poses of the same ligand to determine the most likely pose.

#### 3.4.5. Binding Free Energy Calculations

Following completion of the docking process, the binding free energy estimations were obtained using the Prime Molecular Mechanics/Generalized Born Surface Area (Prime MM-GBSA) method implemented in the Schrödinger software, which estimates the relative binding affinities for each ligand. Each ligand’s relative binding free energy (ΔG) was estimated using the Prime MM-GSBA, which uses the following equation: MM-GBSA ΔG = E_complex (minimized) − (E_ligand (minimized) − E_receptor (minimized). The calculations were performed using the OPLS3e force field. Protein flexibility was applied for any residues having atoms within 5 Å of the ligand. The Prime MM-GBSA method included the VSGB solvation model where the radius of the probe (the radius of the sphere used to calculate solvent-accessible surface area) was set to 1.4 Å, and the variable dielectric constants were set to be 1 to 4 for the protein and 80 for the solvent (water).

#### 3.4.6. Molecular Dynamics Simulations

The Desmond Molecular Dynamics System, ver. 2.3, 2019.1 (Schrödinger) was used to perform 200-ns MD simulations for etazene and its metabolite, M6, which had the best binding free energy score [26]. MD simulations were run using the OPLS3e force field in the Desmond program. The OPLS3e force has comprehensive coverage of small molecules, gives an accurate description of the protein–ligand interactions, and is a good choice for 1-palmitoyl-2-oleoyl-sn-glycero-3-phosphocholine (POPC) bilayer simulations under many biologically relevant conditions. The best scoring pose of etazene and its metabolite M6 in complex with the MOR was embedded into a pre-equilibrated POPC membrane, and the rest of the system was solvated with a TIP3P explicit water solvent model [27], which specifies a 3-site rigid water molecule with charges. All simulations contained one MOR embedded in a lipid bilayer with 92 POPC molecules. The whole system was neutralized using sodium chloride (NaCl) and was set to an ionic strength of 0.15 M. The constructed system was simulated with the default Desmond Molecular Dynamics System, ver. 2.3, 2019.1 (Schrödinger) relaxation protocol with a slight modification. The protocol involved an initial minimization of the solvent while keeping restraints on the solute, followed by short MD simulations, including the following steps: (1) simulation (1 ns) using Brownian dynamics in the NVT ensemble at 10 K with heavy solute atoms restrained; (2) simulation (100 ps) in the NVT ensemble using a Berendsen thermostat (10 K) with heavy solute atoms restrained; (3) simulation (100 ps) in the NPT ensemble using a Berendsen thermostat (10 K) and a Berendsen barostat (1 atm) with non-hydrogen solute atoms restrained; (4) simulation (100 ps) in the NPT ensemble using a Berendsen thermostat (300 K) and a Berendsen barostat (1 atm) with non-hydrogen solute atoms restrained; (5) simulation (200 ps) in the NPT ensemble using a Berendsen thermostat (300 K) and a Berendsen barostat (1 atm) with no restraints; and (6) simulation (5000 ps) in the NPT ensemble using a Langevin thermostat (300 K) and a Langevin barostat (1 atm) with no restraints. Equilibration dynamics for all simulations were performed at a constant temperature of 300 K using the Langevin thermostat with a collision frequency of 1.0 ps^−1^. For the Brownian thermostat, we used a collision frequency of 0.5 ps^−1^. The 200-ns MD simulations were run in the canonical ensemble (NPT) using a Langevin thermostat at 300 K [28]. The trajectory analysis was performed using Event Analysis and the Simulation Interaction Diagram (SID) implemented in the Desmond software.

The Root Mean Square Deviation (RMSD) was used to measure the average change in the displacement of a selection of atoms for a particular frame with respect to a reference frame. For example, the RMSD for frame *x* is:

$${\mathrm{RMSD}}_{x}=\sqrt{\frac{1}{N}\overset{N}{\underset{i=1}{\sum }}\left(r_{i}^{\prime }\left(t_{x}\right)\right)-r_{i}\left(t_{ref}\right){)}^{2}}$$

where N is the number of atoms in the atom selection, $t_{ref}$ is the reference time (typically, the first frame is used as the reference, and it is regarded as time t = 0), and $r_{i}^{\prime }$ is the position of the selected atoms in the frame x after superimposing on the reference frame, where frame *x* is recorded at time *t_x_*. The procedure is repeated for every frame in the simulation trajectory. For the protein RMSD, protein frames are first aligned on the reference frame backbone, with the RMSD calculated based on the atom selection. The protein RMSD can potentially give insights into the structural conformation throughout the simulation. On the other hand, ligand RMSD indicates how stable the ligand is with respect to the protein and its binding pocket. If the values observed are significantly larger than the RMSD of the protein, then it is likely that the ligand has diffused away from its initial binding site.

The Root Mean Square Fluctuation (RMSF) is useful for characterizing local changes along the protein chain. The RMSF for residue *i* is:

$${\mathrm{RMSF}}_{i}=\sqrt{\frac{1}{T}\overset{T}{\underset{t=1}{\sum }}<\left(r_{i}^{\prime }\left(t\right)\right)-r_{i}\left(t_{ref}\right){)}^{2}>}$$

where T is the trajectory time over which the RMSF is calculated, $t_{ref}$ is the reference time, $r_{i}$ is the position of residue *i*, $r^{\prime }$ is the position of atoms in the residue *i* after superposition on the reference, and the angle brackets indicate that the average of the squared distance is taken over the selection of atoms in the residue.

## 4. Conclusions

This study presented the possible metabolites of etazene generated through an in vitro study by liver microsomal assay and also investigated the putative binding site of etazene and its metabolites to the MOR via in silico experimentation. A select number of metabolites (M1–M2 and M5) from this study were found to be structurally identical upon comparison to liver microsomal metabolites and tentative metabolites of etazene previously found in rat urine and serum.

The docking results showed that etazene and its metabolites formed strong H-bonding/π–cation interactions with Asp147 and π–π stacking with Tyr148 of the MOR. A select few of etazene’s metabolites (M1, M2, and M6–M7) displayed strongly calculated binding free energies at the MOR and showed an overlapped binding orientation with the co-crystallized, MOR-bound agonist BU72. The 200-ns MD simulations of MOR–etazene and MOR–M6 complexes revealed that etazene and M6 formed stable and strong interactions with the MOR, and similar interactions were also observed in our docking study. The docking and MD simulations suggest that etazene metabolites may act as strong MOR agonists, highlighting the necessity of experimental validation.

## Figures and Tables

**Table 1:** Docking scores and key amino acid residues involved in the interactions of etazene and its metabolites with the MOR.

Compounds	Glide-GScore (kcal/mol)	Binding Free Energies (ΔG) (kcal/mol)	Key Interactions (H-bond, π–π Stacking, and Cation–π) Involved with MOR
BU72	−5.827	−52.26	Asp54, Asp147, Tyr148
Etazene	−6.521	−58.97	Asp147, Tyr148, His297, Tyr326
M1	−6.497	−56.41	Asp54, Asp147, Tyr326
M2	−6.013	−51.26	Asp147, Tyr148, Lys233
M3	−4.130	−42.11	Asp147, Tyr148
M4	−5.112	−41.21	Asp147, Tyr148
M5	−6.228	−53.42	Asp147, His297, Tyr326
M6	−7.568	−57.80	Gln124, Asp147, Tyr148, Trp318
M7	−5.747	−56.10	Asp54, Asp147, Tyr326
M8	−2.184	−54.20	Asp147, Tyr148, His297
M9	−1.016	−44.04	Asp147, Tyr148, Lys303
AP1	−4.918	−43.37	Gln124, Asn127

**Table 2:** Physicochemical properties of etazene and its metabolites calculated using the ADMET predictor.

Compounds	MWt	S + LogP	S + S_W_	hERG_Filter	T_PSA (Å^2^)	S + MDCK	BBB_Filter	Lipinski Rule-of-5 Violations
Etazene	351.50	4.26	0.58	Yes (99%)	30.29	800.83	High (89%)	NO
M1	323.44	3.54	1.77	Yes (99%)	39.08	440.84	High (82%)	NO
M2	339.44	2.87	0.89	Yes (99%)	59.31	141.28	Low (42%)	NO
M3	295.39	2.23	1.46	Yes (85%)	50.08	224.68	Low (42%)	NO
M4	293.371	2.373	0.33	NO (60%)	50.08	506.377	High (96%)	NO
M5	323.44	3.02	0.88	Yes (99%)	41.29	556.65	High (84%)	NO
M6	339.44	2.56	1.21	Yes (70%)	61.52	196.56	Low (84%)	NO
M7	367.49	3.60	0.70	Yes (99%)	50.52	437.58	High (82%)	NO
M8	367.49	1.93	15.84	Yes (99%)	42.86	582.08	High (94%)	NO
M9	339.44	1.04	13.33	NO (67%)	53.86	371.13	Low (48%)	NO
AP1	339.44	1.36	14.37	NO (69%)	55.12	282.02	High (89%)	NO

## Data Availability

Not applicable.

## Supplementary Materials

The following supporting information can be downloaded at https://www.mdpi.com/article/10.3390/molecules28041601/s1: Figure S1: Overlay of the docked pose and the co-crystalized ligand pose of BU72 with the MOR; Table S1: SMILES notations of all the ligands used in this study; Tables S2 and S3: The 3D coordinates of the minimized structure of MOR with etazene and M6 metabolite.

## Sample Availability

The PDB coordinates of all the compounds [etazene and M6] with the MOR are available on request.
