# Supplementary material for: Identification of the Putative Binding Site of a Benzimidazole Opioid (Etazene) and Its Metabolites at µ-Opioid Receptor: A Human Liver Microsomal Assay and Systematic Computational Study
Source: Molecules. 2023 Feb 7;28(4):1601. doi: 10.3390/molecules28041601 (PMC9959024; doi:10.3390/molecules28041601)
Supplement: Supplementary file 1 [file molecules-28-01601-s001.zip › molecules-2143206-supplementary.pdf]

SUPPORTING INFORMATION

Identification of the Putative Binding Site of a Benzimidazole Opioid (Etazene) and its Metabolites at μ-Opioid Receptor: A Human Liver Microsomal Assay and Systematic Computational Study

Krishna Chaturvedi [1], Isuru Hewamanna [1], Pankaj Pandey [2], Washim Khan [2], Yan-Hong Wang [2], Amar G. Chittiboyina [2], Robert J. Doerksen [3] and Murrell Godfrey [1,*]

[1] Department of Chemistry and Biochemistry, University of Mississippi, University, MS 38677, USA
2 National Center for Natural Products Research, University of Mississippi, University, MS 38677, USA
3 Division of Medicinal Chemistry, Department of BioMolecular Sciences, School of Pharmacy, University of Mississippi, University, MS 38677, USA
* Correspondence: mgodfrey@olemiss.edu; Tel.: +1-662-915-5143

Table of Contents

Figure S1. Overlay of the docked pose and the co-crystalized ligand pose of BU72 with MOR S3
Table S1. SMILES notations of etazene and its metabolites used in this study. S4
Table S2. The 3D coordinates of the minimized structure of MOR with etazene. S5
Table S3. The 3D coordinates of the minimized structure of MOR with M6. S132

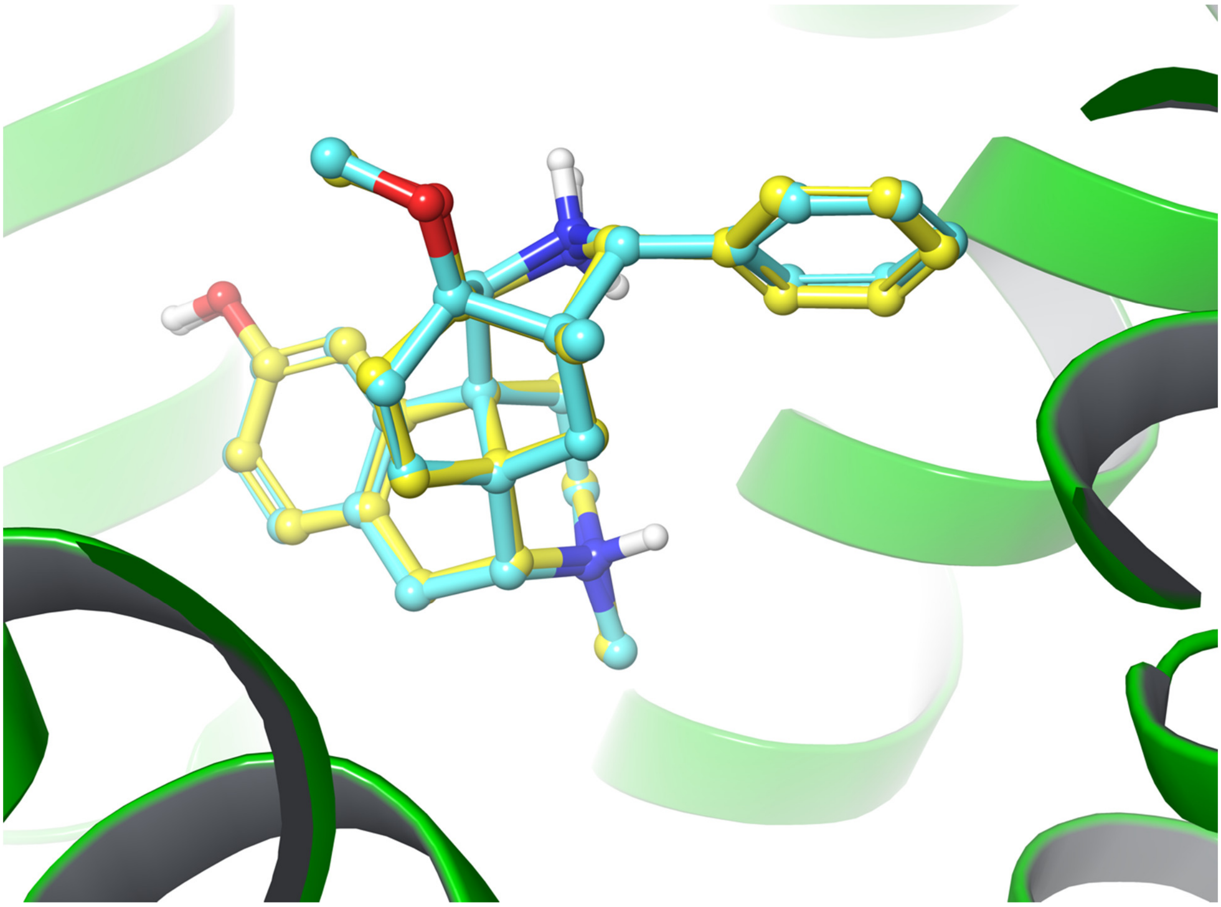

Figure S1. Overlay of the docked pose (carbon in cyan) and the co-crystalized ligand pose of BU72 (carbon in yellow) with MOR.

Table S1. SMILES notations of etazene and its metabolites used in this study.

Compounds	SMILES notations
Etazene	CC[NH+](CC)CCn(c(c12)cccc1)c(n2)Cc3ccc(cc3)OCC
M1	CC[NH2+]CCn(c(c12)cccc1)c(n2)Cc3ccc(cc3)OCC
M2	CC[NH+](CC)CCn(c(c12)cccc1)c(n2)Cc3ccc(O)cc3
M3	CC[NH2+]CCn(c(c12)cccc1)c(n2)Cc3ccc(O)cc3
M4	C=CNCCn(c(c12)cccc1)c(n2)Cc3ccc(O)cc3
M5	CC[NH+](CC)CCn(c(c12)cccc1)c(n2)Cc3ccc(O)cc3
M6	CC[NH+](CC)CCn1c(nc(c12)ccc(c2)O)Cc3ccc(O)cc3
M7	CC[NH+](CC)CCn(c(c12)ccc(c1)O)c(n2)Cc3ccc(cc3)OCC
M8	CC[NH+](CC)CCn1c([n+]([O-])c(c12)cccc2)Cc3ccc(cc3)OCC
M9	CC[NH+](CC)CCn1c([n+]([O-])c(c12)cccc2)Cc3ccc(O)cc3
AP1	CC[N+]([O-])(CC)CCn(c(c12)cccc1)c(n2)Cc3ccc(O)cc3

Table S2. The 3D coordinates of the minimized structure of MOR with etazene.

ATOM	1	N	GLY	A	52	5.641	11.638	-72.150	1.00110.27	N1+
ATOM	2	CA	GLY	A	52	4.612	11.828	-71.143	1.00107.88	C
ATOM	3	C	GLY	A	52	4.546	13.235	-70.580	1.00106.83	C
ATOM	4	O	GLY	A	52	5.549	13.777	-70.112	1.00102.57	O
ATOM	5	HA2	GLY	A	52	3.684	11.499	-71.610	1.00107.88	H
ATOM	6	HA3	GLY	A	52	4.887	11.120	-70.360	1.00107.88	H
ATOM	7	H1	GLY	A	52	5.666	10.675	-72.456	1.00110.27	H
ATOM	8	H2	GLY	A	52	5.476	12.217	-72.960	1.00110.27	H
ATOM	9	H3	GLY	A	52	6.551	11.862	-71.773	1.00110.27	H
ATOM	10	N	ARG	A	53	3.355	13.827	-70.628	1.00107.33	N
ATOM	11	CA	ARG	A	53	3.135	15.162	-70.092	1.00107.30	C
ATOM	12	C	ARG	A	53	2.948	15.108	-68.582	1.00 97.40	C
ATOM	13	O	ARG	A	53	2.361	14.167	-68.040	1.00101.33	O
ATOM	14	CB	ARG	A	53	1.869	15.738	-70.785	1.00 0.00	C
ATOM	15	CG	ARG	A	53	0.542	15.021	-70.425	1.00 0.00	C
ATOM	16	CD	ARG	A	53	-0.655	15.433	-71.293	1.00 0.00	C
ATOM	17	NE	ARG	A	53	-1.847	14.613	-71.017	1.00 0.00	N
ATOM	18	CZ	ARG	A	53	-2.069	13.353	-71.428	1.00 0.00	C
ATOM	19	NH1	ARG	A	53	-1.186	12.712	-72.201	1.00 0.00	N1+
ATOM	20	NH2	ARG	A	53	-3.190	12.728	-71.055	1.00 0.00	N1+
ATOM	21	H	ARG	A	53	2.550	13.344	-70.998	1.00107.33	H
ATOM	22	HA	ARG	A	53	3.997	15.793	-70.321	1.00107.30	H
ATOM	23	HB2	ARG	A	53	1.774	16.798	-70.546	1.00 0.00	H
ATOM	24	HB3	ARG	A	53	2.024	15.696	-71.864	1.00 0.00	H
ATOM	25	HG2	ARG	A	53	0.638	13.935	-70.421	1.00 0.00	H
ATOM	26	HG3	ARG	A	53	0.307	15.298	-69.396	1.00 0.00	H
ATOM	27	HD2	ARG	A	53	-0.957	16.424	-70.958	1.00 0.00	H
ATOM	28	HD3	ARG	A	53	-0.416	15.524	-72.353	1.00 0.00	H
ATOM	29	HE	ARG	A	53	-2.537	15.058	-70.428	1.00 0.00	H
ATOM	30	HH11	ARG	A	53	-0.347	13.178	-72.509	1.00 0.00	H
ATOM	31	HH12	ARG	A	53	-1.354	11.766	-72.511	1.00 0.00	H
ATOM	32	HH21	ARG	A	53	-3.892	13.202	-70.509	1.00 0.00	H
ATOM	33	HH22	ARG	A	53	-3.358	11.759	-71.294	1.00 0.00	H
ATOM	34	N	ASP	A	54	3.451	16.137	-67.902	1.00 90.90	N
ATOM	35	CA	ASP	A	54	3.338	16.212	-66.450	1.00 84.22	C

ATOM	36	C	ASP	A	54	2.840	17.587	-66.021	1.00	72.09	C
ATOM	37	O	ASP	A	54	1.631	17.799	-65.885	1.00	72.61	O
ATOM	38	CB	ASP	A	54	4.667	15.776	-65.767	1.00	0.00	C
ATOM	39	CG	ASP	A	54	4.626	15.632	-64.238	1.00	0.00	C
ATOM	40	OD1	ASP	A	54	3.523	15.715	-63.656	1.00	0.00	O
ATOM	41	OD2	ASP	A	54	5.710	15.382	-63.668	1.00	0.00	O1-
ATOM	42	H	ASP	A	54	3.955	16.879	-68.369	1.00	90.90	H
ATOM	43	HA	ASP	A	54	2.571	15.525	-66.090	1.00	84.22	H
ATOM	44	HB2	ASP	A	54	4.952	14.803	-66.166	1.00	0.00	H
ATOM	45	HB3	ASP	A	54	5.455	16.482	-66.027	1.00	0.00	H
ATOM	46	N	SER	A	55	3.764	18.526	-65.803	1.00	72.40	N
ATOM	47	CA	SER	A	55	3.367	19.877	-65.424	1.00	79.19	C
ATOM	48	C	SER	A	55	2.828	20.654	-66.618	1.00	85.33	C
ATOM	49	O	SER	A	55	1.866	21.419	-66.481	1.00	86.66	O
ATOM	50	CB	SER	A	55	4.562	20.561	-64.717	1.00	81.52	C
ATOM	51	OG	SER	A	55	5.734	20.624	-65.514	1.00	87.70	O
ATOM	52	H	SER	A	55	4.749	18.335	-65.926	1.00	72.40	H
ATOM	53	HA	SER	A	55	2.584	19.819	-64.664	1.00	79.19	H
ATOM	54	HB2	SER	A	55	4.290	21.570	-64.409	1.00	81.52	H
ATOM	55	HB3	SER	A	55	4.802	20.023	-63.798	1.00	81.52	H
ATOM	56	HG	SER	A	55	6.257	19.822	-65.328	1.00	87.70	H
ATOM	57	N	LEU	A	56	3.432	20.473	-67.787	1.00	84.34	N
ATOM	58	CA	LEU	A	56	2.998	21.175	-68.987	1.00	84.78	C
ATOM	59	C	LEU	A	56	1.677	20.625	-69.503	1.00	85.67	C
ATOM	60	O	LEU	A	56	1.599	19.481	-69.951	1.00	83.26	O
ATOM	61	CB	LEU	A	56	4.092	21.083	-70.070	1.00	85.66	C
ATOM	62	CG	LEU	A	56	3.814	21.862	-71.374	1.00	87.52	C
ATOM	63	CD1	LEU	A	56	3.682	23.376	-71.149	1.00	87.52	C
ATOM	64	CD2	LEU	A	56	4.894	21.551	-72.419	1.00	90.95	C
ATOM	65	H	LEU	A	56	4.237	19.862	-67.869	1.00	84.34	H
ATOM	66	HA	LEU	A	56	2.874	22.229	-68.730	1.00	84.78	H
ATOM	67	HB2	LEU	A	56	5.038	21.430	-69.653	1.00	85.66	H
ATOM	68	HB3	LEU	A	56	4.228	20.031	-70.322	1.00	85.66	H
ATOM	69	HG	LEU	A	56	2.876	21.505	-71.802	1.00	87.52	H
ATOM	70	HD11	LEU	A	56	4.212	23.937	-71.919	1.00	87.52	H
ATOM	71	HD12	LEU	A	56	2.639	23.691	-71.192	1.00	87.52	H
ATOM	72	HD13	LEU	A	56	4.087	23.694	-70.188	1.00	87.52	H
ATOM	73	HD21	LEU	A	56	4.506	21.656	-73.433	1.00	90.95	H
ATOM	74	HD22	LEU	A	56	5.754	22.216	-72.323	1.00	90.95	H
ATOM	75	HD23	LEU	A	56	5.260	20.534	-72.316	1.00	90.95	H

```
ATOM     76  N   CYS A  57       0.642  21.453 -69.436  1.00 90.27           N
ATOM     77  CA  CYS A  57      -0.660  21.086 -69.924  1.00100.97           C
ATOM     78  C   CYS A  57      -0.870  21.460 -71.386  1.00113.61           C
ATOM     79  O   CYS A  57      -0.693  22.610 -71.800  1.00110.96           O
ATOM     80  CB  CYS A  57      -1.740  21.856 -69.128  1.00  0.00           C
ATOM     81  SG  CYS A  57      -1.670  21.477 -67.354  1.00  0.00           S
ATOM     82  H   CYS A  57       0.749  22.382 -69.056  1.00 90.27           H
ATOM     83  HA  CYS A  57      -0.790  20.022 -69.745  1.00100.97           H
ATOM     84  HB2 CYS A  57      -1.630  22.934 -69.255  1.00  0.00           H
ATOM     85  HB3 CYS A  57      -2.738  21.598 -69.489  1.00  0.00           H
ATOM     86  HG  CYS A  57      -2.098  20.215 -67.465  1.00  0.00           H
ATOM     87  N   PRO A  58      -1.243  20.464 -72.205  1.00124.15           N
ATOM     88  CA  PRO A  58      -1.641  20.732 -73.590  1.00135.47           C
ATOM     89  C   PRO A  58      -2.849  21.662 -73.630  1.00143.48           C
ATOM     90  O   PRO A  58      -3.984  21.211 -73.479  1.00143.53           O
ATOM     91  CB  PRO A  58      -1.985  19.342 -74.136  1.00134.79           C
ATOM     92  CG  PRO A  58      -1.220  18.397 -73.270  1.00129.71           C
ATOM     93  CD  PRO A  58      -1.214  19.021 -71.907  1.00126.49           C
ATOM     94  HA  PRO A  58      -0.791  21.141 -74.140  1.00135.47           H
ATOM     95  HB2 PRO A  58      -3.041  19.086 -74.045  1.00134.79           H
ATOM     96  HB3 PRO A  58      -1.726  19.234 -75.190  1.00134.79           H
ATOM     97  HG2 PRO A  58      -1.668  17.406 -73.297  1.00129.71           H
ATOM     98  HG3 PRO A  58      -0.196  18.320 -73.639  1.00129.71           H
ATOM     99  HD2 PRO A  58      -2.097  18.746 -71.328  1.00126.49           H
ATOM    100  HD3 PRO A  58      -0.305  18.735 -71.384  1.00126.49           H
ATOM    101  N   PRO A  59      -2.596  22.956 -73.821  1.00149.23           N
ATOM    102  CA  PRO A  59      -3.615  23.983 -73.663  1.00155.25           C
ATOM    103  C   PRO A  59      -4.064  24.617 -74.970  1.00153.72           C
ATOM    104  O   PRO A  59      -4.914  25.514 -74.933  1.00158.07           O
ATOM    105  CB  PRO A  59      -3.107  25.090 -72.721  1.00  0.00           C
ATOM    106  CG  PRO A  59      -1.645  25.187 -73.004  1.00  0.00           C
ATOM    107  CD  PRO A  59      -1.432  23.865 -73.762  1.00  0.00           C
ATOM    108  HA  PRO A  59      -4.526  23.582 -73.215  1.00155.25           H
ATOM    109  HB2 PRO A  59      -3.613  26.053 -72.810  1.00  0.00           H
ATOM    110  HB3 PRO A  59      -3.250  24.757 -71.692  1.00  0.00           H
ATOM    111  HG2 PRO A  59      -1.397  26.046 -73.630  1.00  0.00           H
ATOM    112  HG3 PRO A  59      -1.065  25.248 -72.083  1.00  0.00           H
ATOM    113  HD2 PRO A  59      -1.368  24.203 -74.796  1.00  0.00           H
ATOM    114  HD3 PRO A  59      -0.420  23.462 -73.799  1.00  0.00           H
ATOM    115  N   THR A  60      -3.526  24.173 -76.110  1.00149.99           N
```

ATOM 116 CA THR A 60 -3.749 24.778 -77.423 1.00148.17 C
ATOM 117 C THR A 60 -5.177 25.279 -77.601 1.00148.90 C
ATOM 118 O THR A 60 -6.143 24.544 -77.371 1.00148.23 O
ATOM 119 CB THR A 60 -3.399 23.784 -78.533 1.00146.14 C
ATOM 120 CG2 THR A 60 -3.132 24.522 -79.837 1.00147.10 C
ATOM 121 OG1 THR A 60 -2.220 23.059 -78.165 1.00142.32 O
ATOM 122 H THR A 60 -2.854 23.422 -76.079 1.00149.99 H
ATOM 123 HA THR A 60 -3.068 25.630 -77.477 1.00148.17 H
ATOM 124 HB THR A 60 -4.201 23.057 -78.676 1.00146.14 H
ATOM 125 HG1 THR A 60 -2.472 22.361 -77.555 1.00142.32 H
ATOM 126 HG21 THR A 60 -2.835 23.824 -80.620 1.00147.10 H
ATOM 127 HG22 THR A 60 -4.017 25.046 -80.197 1.00147.10 H
ATOM 128 HG23 THR A 60 -2.330 25.253 -79.729 1.00147.10 H
ATOM 129 N GLY A 61 -5.310 26.535 -78.012 1.00149.88 N
ATOM 130 CA GLY A 61 -6.559 27.252 -77.869 1.00150.23 C
ATOM 131 C GLY A 61 -6.469 28.159 -76.659 1.00148.79 C
ATOM 132 O GLY A 61 -7.088 27.891 -75.625 1.00147.77 O
ATOM 133 H GLY A 61 -4.488 27.079 -78.228 1.00149.88 H
ATOM 134 HA2 GLY A 61 -6.717 27.858 -78.762 1.00150.23 H
ATOM 135 HA3 GLY A 61 -7.432 26.602 -77.784 1.00150.23 H
ATOM 136 N SER A 62 -5.682 29.236 -76.790 1.00147.63 N
ATOM 137 CA SER A 62 -5.277 30.147 -75.721 1.00142.05 C
ATOM 138 C SER A 62 -6.404 30.450 -74.741 1.00135.94 C
ATOM 139 O SER A 62 -7.563 30.616 -75.147 1.00137.86 O
ATOM 140 CB SER A 62 -4.742 31.451 -76.317 1.00144.61 C
ATOM 141 OG SER A 62 -5.694 32.049 -77.178 1.00148.40 O
ATOM 142 H SER A 62 -5.256 29.419 -77.686 1.00147.63 H
ATOM 143 HA SER A 62 -4.462 29.637 -75.208 1.00142.05 H
ATOM 144 HB2 SER A 62 -4.471 32.165 -75.538 1.00144.61 H
ATOM 145 HB3 SER A 62 -3.835 31.257 -76.890 1.00144.61 H
ATOM 146 HG SER A 62 -6.474 32.274 -76.663 1.00148.40 H
ATOM 147 N PRO A 63 -6.098 30.545 -73.450 1.00128.05 N
ATOM 148 CA PRO A 63 -7.155 30.699 -72.446 1.00118.94 C
ATOM 149 C PRO A 63 -7.864 32.039 -72.573 1.00113.02 C
ATOM 150 O PRO A 63 -7.345 33.006 -73.134 1.00114.91 O
ATOM 151 CB PRO A 63 -6.397 30.598 -71.119 1.00115.15 C
ATOM 152 CG PRO A 63 -5.015 31.064 -71.451 1.00117.95 C
ATOM 153 CD PRO A 63 -4.753 30.575 -72.848 1.00123.25 C
ATOM 154 HA PRO A 63 -7.885 29.890 -72.524 1.00118.94 H
ATOM 155 HB2 PRO A 63 -6.846 31.167 -70.303 1.00115.15 H

```
ATOM    156  HB3 PRO A  63      -6.365  29.554 -70.803  1.00115.15           H
ATOM    157  HG2 PRO A  63      -5.000  32.155 -71.446  1.00117.95           H
ATOM    158  HG3 PRO A  63      -4.266  30.727 -70.733  1.00117.95           H
ATOM    159  HD2 PRO A  63      -4.053  31.224 -73.374  1.00123.25           H
ATOM    160  HD3 PRO A  63      -4.349  29.562 -72.830  1.00123.25           H
ATOM    161  N   SER A  64      -9.079  32.079 -72.036  1.00107.37           N
ATOM    162  CA  SER A  64      -9.865  33.300 -72.012  1.00106.28           C
ATOM    163  C   SER A  64      -9.489  34.146 -70.796  1.00122.18           C
ATOM    164  O   SER A  64      -8.639  33.777 -69.981  1.00114.78           O
ATOM    165  CB  SER A  64     -11.357  32.975 -72.013  1.00106.54           C
ATOM    166  OG  SER A  64     -11.723  32.270 -70.841  1.00103.81           O
ATOM    167  H   SER A  64      -9.469  31.271 -71.572  1.00107.37           H
ATOM    168  HA  SER A  64      -9.666  33.893 -72.907  1.00106.28           H
ATOM    169  HB2 SER A  64     -11.963  33.879 -72.088  1.00106.54           H
ATOM    170  HB3 SER A  64     -11.608  32.365 -72.881  1.00106.54           H
ATOM    171  HG  SER A  64     -12.653  32.037 -70.908  1.00103.81           H
ATOM    172  N   MET A  65     -10.142  35.302 -70.672  1.00 86.88           N
ATOM    173  CA  MET A  65      -9.847  36.213 -69.572  1.00 87.64           C
ATOM    174  C   MET A  65     -10.326  35.647 -68.241  1.00 82.86           C
ATOM    175  O   MET A  65      -9.552  35.550 -67.281  1.00 79.45           O
ATOM    176  CB  MET A  65     -10.492  37.573 -69.839  1.00 96.03           C
ATOM    177  CG  MET A  65     -10.392  38.547 -68.679  1.00105.14           C
ATOM    178  SD  MET A  65      -8.705  39.117 -68.427  1.00112.18           S
ATOM    179  CE  MET A  65      -8.374  39.884 -70.011  1.00103.68           C
ATOM    180  H   MET A  65     -10.829  35.593 -71.352  1.00 86.88           H
ATOM    181  HA  MET A  65      -8.766  36.355 -69.512  1.00 87.64           H
ATOM    182  HB2 MET A  65     -10.093  38.001 -70.757  1.00 96.03           H
ATOM    183  HB3 MET A  65     -11.553  37.426 -70.050  1.00 96.03           H
ATOM    184  HG2 MET A  65     -10.996  39.425 -68.909  1.00105.14           H
ATOM    185  HG3 MET A  65     -10.796  38.165 -67.742  1.00105.14           H
ATOM    186  HE1 MET A  65      -7.480  40.503 -69.937  1.00103.68           H
ATOM    187  HE2 MET A  65      -8.193  39.136 -70.782  1.00103.68           H
ATOM    188  HE3 MET A  65      -9.204  40.521 -70.317  1.00103.68           H
ATOM    189  N   ILE A  66     -11.603  35.264 -68.168  1.00 83.31           N
ATOM    190  CA  ILE A  66     -12.193  34.856 -66.897  1.00 87.12           C
ATOM    191  C   ILE A  66     -11.557  33.571 -66.381  1.00 81.04           C
ATOM    192  O   ILE A  66     -11.418  33.385 -65.165  1.00 78.67           O
ATOM    193  CB  ILE A  66     -13.719  34.713 -67.051  1.00  0.00           C
ATOM    194  CG1 ILE A  66     -14.133  33.232 -67.169  1.00  0.00           C
ATOM    195  CG2 ILE A  66     -14.234  35.536 -68.245  1.00  0.00           C
```

```
ATOM    196  CD1 ILE A  66     -15.627  32.906 -67.327  1.00  0.00           C
ATOM    197  H   ILE A  66    -12.217  35.337 -68.965  1.00 83.31           H
ATOM    198  HA  ILE A  66     -11.998  35.642 -66.163  1.00 87.12           H
ATOM    199  HB  ILE A  66     -14.180  35.119 -66.150  1.00  0.00           H
ATOM    200 HG12 ILE A  66     -13.643  32.793 -68.036  1.00  0.00           H
ATOM    201 HG13 ILE A  66     -13.859  32.650 -66.288  1.00  0.00           H
ATOM    202 HG21 ILE A  66     -15.323  35.565 -68.242  1.00  0.00           H
ATOM    203 HG22 ILE A  66     -13.901  36.574 -68.197  1.00  0.00           H
ATOM    204 HG23 ILE A  66     -13.940  35.116 -69.208  1.00  0.00           H
ATOM    205 HD11 ILE A  66     -15.827  31.860 -67.091  1.00  0.00           H
ATOM    206 HD12 ILE A  66     -16.235  33.519 -66.662  1.00  0.00           H
ATOM    207 HD13 ILE A  66     -15.963  33.084 -68.348  1.00  0.00           H
ATOM    208  N   THR A  67     -11.152  32.671 -67.279  1.00 80.92           N
ATOM    209  CA  THR A  67     -10.598  31.394 -66.838  1.00 73.47           C
ATOM    210  C   THR A  67      -9.184  31.556 -66.292  1.00 64.70           C
ATOM    211  O   THR A  67      -8.847  30.976 -65.253  1.00 57.65           O
ATOM    212  CB  THR A  67     -10.617  30.382 -67.985  1.00 72.78           C
ATOM    213  CG2 THR A  67     -12.048  30.098 -68.417  1.00 57.52           C
ATOM    214  OG1 THR A  67      -9.875  30.899 -69.096  1.00 85.80           O
ATOM    215  H   THR A  67     -11.243  32.836 -68.271  1.00 80.92           H
ATOM    216  HA  THR A  67     -11.219  30.989 -66.035  1.00 73.47           H
ATOM    217  HB  THR A  67     -10.147  29.448 -67.672  1.00 72.78           H
ATOM    218  HG1 THR A  67     -10.447  31.484 -69.604  1.00 85.80           H
ATOM    219 HG21 THR A  67     -12.055  29.415 -69.259  1.00 57.52           H
ATOM    220 HG22 THR A  67     -12.618  29.636 -67.611  1.00 57.52           H
ATOM    221 HG23 THR A  67     -12.585  30.989 -68.731  1.00 57.52           H
ATOM    222  N   ALA A  68      -8.344  32.337 -66.976  1.00 65.22           N
ATOM    223  CA  ALA A  68      -6.972  32.525 -66.513  1.00 65.95           C
ATOM    224  C   ALA A  68      -6.937  33.226 -65.160  1.00 66.66           C
ATOM    225  O   ALA A  68      -6.132  32.873 -64.290  1.00 62.13           O
ATOM    226  CB  ALA A  68      -6.169  33.310 -67.549  1.00 70.78           C
ATOM    227  H   ALA A  68      -8.623  32.791 -67.835  1.00 65.22           H
ATOM    228  HA  ALA A  68      -6.511  31.552 -66.374  1.00 65.95           H
ATOM    229  HB1 ALA A  68      -5.141  33.466 -67.219  1.00 70.78           H
ATOM    230  HB2 ALA A  68      -6.128  32.773 -68.497  1.00 70.78           H
ATOM    231  HB3 ALA A  68      -6.611  34.288 -67.744  1.00 70.78           H
ATOM    232  N   ILE A  69      -7.807  34.219 -64.962  1.00 69.34           N
ATOM    233  CA  ILE A  69      -7.883  34.889 -63.667  1.00 67.41           C
ATOM    234  C   ILE A  69      -8.378  33.925 -62.598  1.00 64.05           C
ATOM    235  O   ILE A  69      -7.874  33.919 -61.467  1.00 61.98           O
```

```
ATOM    236  CB  ILE A  69      -8.780  36.137 -63.758  1.00 66.30           C
ATOM    237  CG1 ILE A  69      -8.198  37.145 -64.747  1.00 66.14           C
ATOM    238  CG2 ILE A  69      -8.948  36.778 -62.388  1.00 66.51           C
ATOM    239  CD1 ILE A  69      -9.021  38.408 -64.871  1.00 71.79           C
ATOM    240  H   ILE A  69      -8.448  34.511 -65.688  1.00 69.34           H
ATOM    241  HA  ILE A  69      -6.880  35.218 -63.388  1.00 67.41           H
ATOM    242  HB  ILE A  69      -9.766  35.837 -64.120  1.00 66.30           H
ATOM    243 HG12 ILE A  69      -7.197  37.426 -64.417  1.00 66.14           H
ATOM    244 HG13 ILE A  69      -8.055  36.714 -65.734  1.00 66.14           H
ATOM    245 HG21 ILE A  69      -9.588  37.658 -62.419  1.00 66.51           H
ATOM    246 HG22 ILE A  69      -9.436  36.125 -61.665  1.00 66.51           H
ATOM    247 HG23 ILE A  69      -7.988  37.082 -61.968  1.00 66.51           H
ATOM    248 HD11 ILE A  69      -8.663  39.015 -65.697  1.00 71.79           H
ATOM    249 HD12 ILE A  69     -10.074  38.187 -65.047  1.00 71.79           H
ATOM    250 HD13 ILE A  69      -8.941  39.041 -63.988  1.00 71.79           H
ATOM    251  N   THR A  70      -9.367  33.095 -62.936  1.00 59.61           N
ATOM    252  CA  THR A  70      -9.900  32.141 -61.968  1.00 62.79           C
ATOM    253  C   THR A  70      -8.822  31.165 -61.510  1.00 53.25           C
ATOM    254  O   THR A  70      -8.626  30.958 -60.308  1.00 54.69           O
ATOM    255  CB  THR A  70     -11.089  31.389 -62.570  1.00 66.88           C
ATOM    256  CG2 THR A  70     -11.650  30.389 -61.571  1.00 63.88           C
ATOM    257  OG1 THR A  70     -12.114  32.323 -62.929  1.00 74.48           O
ATOM    258  H   THR A  70      -9.771  33.107 -63.862  1.00 59.61           H
ATOM    259  HA  THR A  70     -10.246  32.699 -61.095  1.00 62.79           H
ATOM    260  HB  THR A  70     -10.796  30.860 -63.477  1.00 66.88           H
ATOM    261  HG1 THR A  70     -11.824  32.818 -63.706  1.00 74.48           H
ATOM    262 HG21 THR A  70     -12.584  29.965 -61.940  1.00 63.88           H
ATOM    263 HG22 THR A  70     -10.979  29.547 -61.396  1.00 63.88           H
ATOM    264 HG23 THR A  70     -11.864  30.858 -60.610  1.00 63.88           H
ATOM    265  N   ILE A  71      -8.106  30.562 -62.462  1.00 53.94           N
ATOM    266  CA  ILE A  71      -7.037  29.630 -62.114  1.00 58.39           C
ATOM    267  C   ILE A  71      -5.945  30.344 -61.329  1.00 59.04           C
ATOM    268  O   ILE A  71      -5.357  29.781 -60.395  1.00 54.06           O
ATOM    269  CB  ILE A  71      -6.495  28.867 -63.361  1.00 57.59           C
ATOM    270  CG1 ILE A  71      -7.613  27.942 -63.919  1.00 51.97           C
ATOM    271  CG2 ILE A  71      -5.219  28.040 -63.082  1.00 51.59           C
ATOM    272  CD1 ILE A  71      -7.318  27.299 -65.284  1.00 53.42           C
ATOM    273  H   ILE A  71      -8.275  30.742 -63.444  1.00 53.94           H
ATOM    274  HA  ILE A  71      -7.461  28.848 -61.482  1.00 58.39           H
ATOM    275  HB  ILE A  71      -6.255  29.603 -64.131  1.00 57.59           H
```

```
ATOM    276 HG12 ILE A  71      -7.823  27.154 -63.195  1.00 51.97           H
ATOM    277 HG13 ILE A  71      -8.544  28.500 -64.016  1.00 51.97           H
ATOM    278 HG21 ILE A  71      -4.908  27.455 -63.948  1.00 51.59           H
ATOM    279 HG22 ILE A  71      -4.370  28.675 -62.827  1.00 51.59           H
ATOM    280 HG23 ILE A  71      -5.371  27.347 -62.256  1.00 51.59           H
ATOM    281 HD11 ILE A  71      -7.251  26.214 -65.204  1.00 53.42           H
ATOM    282 HD12 ILE A  71      -8.101  27.532 -66.005  1.00 53.42           H
ATOM    283 HD13 ILE A  71      -6.381  27.650 -65.706  1.00 53.42           H
ATOM    284  N   MET A  72      -5.667  31.600 -61.683  1.00 52.90           N
ATOM    285  CA  MET A  72      -4.676  32.377 -60.947  1.00 57.12           C
ATOM    286  C   MET A  72      -5.130  32.638 -59.518  1.00 55.10           C
ATOM    287  O   MET A  72      -4.369  32.426 -58.567  1.00 49.99           O
ATOM    288  CB  MET A  72      -4.395  33.694 -61.669  1.00 66.43           C
ATOM    289  CG  MET A  72      -3.244  33.580 -62.628  1.00 88.45           C
ATOM    290  SD  MET A  72      -1.977  32.566 -61.852  1.00103.04           S
ATOM    291  CE  MET A  72      -1.334  33.696 -60.618  1.00106.69           C
ATOM    292  H   MET A  72      -6.135  32.046 -62.461  1.00 52.90           H
ATOM    293  HA  MET A  72      -3.877  31.678 -60.743  1.00 57.12           H
ATOM    294  HB2 MET A  72      -5.281  34.057 -62.184  1.00 66.43           H
ATOM    295  HB3 MET A  72      -4.150  34.483 -60.956  1.00 66.43           H
ATOM    296  HG2 MET A  72      -3.560  33.085 -63.547  1.00 88.45           H
ATOM    297  HG3 MET A  72      -2.845  34.557 -62.901  1.00 88.45           H
ATOM    298  HE1 MET A  72      -0.467  33.265 -60.119  1.00106.69           H
ATOM    299  HE2 MET A  72      -1.030  34.634 -61.082  1.00106.69           H
ATOM    300  HE3 MET A  72      -2.087  33.911 -59.861  1.00106.69           H
ATOM    301  N   ALA A  73      -6.366  33.114 -59.351  1.00 58.28           N
ATOM    302  CA  ALA A  73      -6.901  33.318 -58.011  1.00 56.01           C
ATOM    303  C   ALA A  73      -6.957  32.013 -57.232  1.00 53.49           C
ATOM    304  O   ALA A  73      -6.779  32.013 -56.009  1.00 55.46           O
ATOM    305  CB  ALA A  73      -8.288  33.956 -58.090  1.00 56.84           C
ATOM    306  H   ALA A  73      -6.971  33.309 -60.141  1.00 58.28           H
ATOM    307  HA  ALA A  73      -6.243  34.006 -57.476  1.00 56.01           H
ATOM    308  HB1 ALA A  73      -8.702  34.130 -57.096  1.00 56.84           H
ATOM    309  HB2 ALA A  73      -8.245  34.919 -58.600  1.00 56.84           H
ATOM    310  HB3 ALA A  73      -8.989  33.325 -58.638  1.00 56.84           H
ATOM    311  N   LEU A  74      -7.191  30.893 -57.920  1.00 52.68           N
ATOM    312  CA  LEU A  74      -7.207  29.593 -57.257  1.00 54.76           C
ATOM    313  C   LEU A  74      -5.863  29.295 -56.604  1.00 44.00           C
ATOM    314  O   LEU A  74      -5.784  29.059 -55.393  1.00 46.36           O
ATOM    315  CB  LEU A  74      -7.575  28.498 -58.259  1.00 59.23           C
```

ATOM 316 CG LEU A 74 -8.996 27.937 -58.197 1.00 63.81 C
ATOM 317 CD1 LEU A 74 -9.197 26.878 -59.271 1.00 60.68 C
ATOM 318 CD2 LEU A 74 -9.279 27.363 -56.819 1.00 60.26 C
ATOM 319 H LEU A 74 -7.377 30.919 -58.915 1.00 52.68 H
ATOM 320 HA LEU A 74 -7.955 29.635 -56.463 1.00 54.76 H
ATOM 321 HB2 LEU A 74 -7.393 28.852 -59.267 1.00 59.23 H
ATOM 322 HB3 LEU A 74 -6.902 27.644 -58.151 1.00 59.23 H
ATOM 323 HG LEU A 74 -9.694 28.754 -58.388 1.00 63.81 H
ATOM 324 HD11 LEU A 74 -10.095 27.083 -59.855 1.00 60.68 H
ATOM 325 HD12 LEU A 74 -8.366 26.839 -59.977 1.00 60.68 H
ATOM 326 HD13 LEU A 74 -9.286 25.882 -58.853 1.00 60.68 H
ATOM 327 HD21 LEU A 74 -9.838 26.427 -56.845 1.00 60.26 H
ATOM 328 HD22 LEU A 74 -8.367 27.171 -56.251 1.00 60.26 H
ATOM 329 HD23 LEU A 74 -9.880 28.060 -56.235 1.00 60.26 H
ATOM 330 N TYR A 75 -4.789 29.304 -57.398 1.00 45.87 N
ATOM 331 CA TYR A 75 -3.469 28.984 -56.864 1.00 46.23 C
ATOM 332 C TYR A 75 -3.040 29.985 -55.799 1.00 44.98 C
ATOM 333 O TYR A 75 -2.413 29.609 -54.802 1.00 43.70 O
ATOM 334 CB TYR A 75 -2.415 28.953 -57.987 1.00 46.16 C
ATOM 335 CG TYR A 75 -2.370 27.679 -58.805 1.00 52.53 C
ATOM 336 CD1 TYR A 75 -1.926 26.485 -58.199 1.00 52.84 C
ATOM 337 CD2 TYR A 75 -2.731 27.679 -60.168 1.00 61.70 C
ATOM 338 CE1 TYR A 75 -1.845 25.296 -58.947 1.00 59.53 C
ATOM 339 CE2 TYR A 75 -2.657 26.487 -60.915 1.00 61.15 C
ATOM 340 CZ TYR A 75 -2.218 25.296 -60.302 1.00 56.42 C
ATOM 341 OH TYR A 75 -2.153 24.135 -61.009 1.00 47.68 O
ATOM 342 H TYR A 75 -4.869 29.500 -58.388 1.00 45.87 H
ATOM 343 HA TYR A 75 -3.521 28.002 -56.389 1.00 46.23 H
ATOM 344 HB2 TYR A 75 -2.511 29.827 -58.634 1.00 46.16 H
ATOM 345 HB3 TYR A 75 -1.426 29.029 -57.532 1.00 46.16 H
ATOM 346 HD1 TYR A 75 -1.648 26.481 -57.157 1.00 52.84 H
ATOM 347 HD2 TYR A 75 -3.067 28.586 -60.649 1.00 61.70 H
ATOM 348 HE1 TYR A 75 -1.502 24.379 -58.486 1.00 59.53 H
ATOM 349 HE2 TYR A 75 -2.937 26.494 -61.958 1.00 61.15 H
ATOM 350 HH TYR A 75 -2.618 24.179 -61.864 1.00 47.68 H
ATOM 351 N SER A 76 -3.375 31.264 -55.985 1.00 41.44 N
ATOM 352 CA SER A 76 -2.945 32.282 -55.034 1.00 44.48 C
ATOM 353 C SER A 76 -3.661 32.135 -53.696 1.00 45.16 C
ATOM 354 O SER A 76 -3.026 32.203 -52.638 1.00 43.85 O
ATOM 355 CB SER A 76 -3.177 33.675 -55.618 1.00 48.53 C

```
ATOM    356  OG  SER A  76      -2.407  33.867 -56.791  1.00 70.85           O
ATOM    357  H   SER A  76      -3.896  31.560 -56.800  1.00 41.44           H
ATOM    358  HA  SER A  76      -1.873  32.170 -54.854  1.00 44.48           H
ATOM    359  HB2 SER A  76      -4.230  33.828 -55.860  1.00 48.53           H
ATOM    360  HB3 SER A  76      -2.899  34.446 -54.898  1.00 48.53           H
ATOM    361  HG  SER A  76      -2.811  33.362 -57.503  1.00 70.85           H
ATOM    362  N   ILE A  77      -4.978  31.929 -53.723  1.00 45.99           N
ATOM    363  CA  ILE A  77      -5.746  31.840 -52.484  1.00 47.16           C
ATOM    364  C   ILE A  77      -5.347  30.600 -51.694  1.00 45.25           C
ATOM    365  O   ILE A  77      -5.115  30.665 -50.482  1.00 44.45           O
ATOM    366  CB  ILE A  77      -7.255  31.860 -52.789  1.00 58.94           C
ATOM    367  CG1 ILE A  77      -7.674  33.245 -53.290  1.00 65.91           C
ATOM    368  CG2 ILE A  77      -8.061  31.465 -51.561  1.00 57.35           C
ATOM    369  CD1 ILE A  77      -9.086  33.301 -53.825  1.00 68.01           C
ATOM    370  H   ILE A  77      -5.488  31.865 -54.596  1.00 45.99           H
ATOM    371  HA  ILE A  77      -5.510  32.711 -51.868  1.00 47.16           H
ATOM    372  HB  ILE A  77      -7.463  31.131 -53.574  1.00 58.94           H
ATOM    373 HG12 ILE A  77      -7.580  33.962 -52.474  1.00 65.91           H
ATOM    374 HG13 ILE A  77      -6.996  33.609 -54.061  1.00 65.91           H
ATOM    375 HG21 ILE A  77      -9.130  31.585 -51.734  1.00 57.35           H
ATOM    376 HG22 ILE A  77      -7.934  30.418 -51.283  1.00 57.35           H
ATOM    377 HG23 ILE A  77      -7.805  32.080 -50.697  1.00 57.35           H
ATOM    378 HD11 ILE A  77      -9.193  34.112 -54.546  1.00 68.01           H
ATOM    379 HD12 ILE A  77      -9.363  32.376 -54.332  1.00 68.01           H
ATOM    380 HD13 ILE A  77      -9.807  33.478 -53.027  1.00 68.01           H
ATOM    381  N   VAL A  78      -5.255  29.452 -52.370  1.00 37.62           N
ATOM    382  CA  VAL A  78      -4.893  28.215 -51.683  1.00 39.31           C
ATOM    383  C   VAL A  78      -3.495  28.323 -51.088  1.00 43.66           C
ATOM    384  O   VAL A  78      -3.239  27.855 -49.971  1.00 41.53           O
ATOM    385  CB  VAL A  78      -5.014  27.017 -52.644  1.00 41.14           C
ATOM    386  CG1 VAL A  78      -4.493  25.750 -51.989  1.00 35.97           C
ATOM    387  CG2 VAL A  78      -6.459  26.835 -53.074  1.00 41.50           C
ATOM    388  H   VAL A  78      -5.441  29.406 -53.364  1.00 37.62           H
ATOM    389  HA  VAL A  78      -5.592  28.065 -50.857  1.00 39.31           H
ATOM    390  HB  VAL A  78      -4.415  27.211 -53.536  1.00 41.14           H
ATOM    391 HG11 VAL A  78      -4.647  24.906 -52.659  1.00 35.97           H
ATOM    392 HG12 VAL A  78      -3.431  25.768 -51.753  1.00 35.97           H
ATOM    393 HG13 VAL A  78      -5.041  25.520 -51.078  1.00 35.97           H
ATOM    394 HG21 VAL A  78      -6.551  26.010 -53.778  1.00 41.50           H
ATOM    395 HG22 VAL A  78      -7.096  26.602 -52.220  1.00 41.50           H
```

ATOM	396	HG23	VAL	A	78	-6.877	27.718	-53.553	1.00	41.50	H
ATOM	397	N	CYS	A	79	-2.572	28.954	-51.816	1.00	44.10	N
ATOM	398	CA	CYS	A	79	-1.216	29.127	-51.307	1.00	42.55	C
ATOM	399	C	CYS	A	79	-1.201	30.011	-50.066	1.00	47.69	C
ATOM	400	O	CYS	A	79	-0.602	29.653	-49.045	1.00	45.61	O
ATOM	401	CB	CYS	A	79	-0.320	29.714	-52.397	1.00	39.85	C
ATOM	402	SG	CYS	A	79	1.361	30.072	-51.857	1.00	48.18	S
ATOM	403	H	CYS	A	79	-2.787	29.331	-52.730	1.00	44.10	H
ATOM	404	HA	CYS	A	79	-0.819	28.149	-51.037	1.00	42.55	H
ATOM	405	HB2	CYS	A	79	-0.276	29.042	-53.249	1.00	39.85	H
ATOM	406	HB3	CYS	A	79	-0.744	30.645	-52.774	1.00	39.85	H
ATOM	407	HG	CYS	A	79	1.729	28.798	-51.686	1.00	48.18	H
ATOM	408	N	VAL	A	80	-1.862	31.169	-50.135	1.00	41.50	N
ATOM	409	CA	VAL	A	80	-1.849	32.110	-49.017	1.00	43.75	C
ATOM	410	C	VAL	A	80	-2.537	31.506	-47.798	1.00	45.31	C
ATOM	411	O	VAL	A	80	-2.028	31.587	-46.675	1.00	43.24	O
ATOM	412	CB	VAL	A	80	-2.499	33.443	-49.431	1.00	42.20	C
ATOM	413	CG1	VAL	A	80	-2.723	34.327	-48.215	1.00	51.14	C
ATOM	414	CG2	VAL	A	80	-1.632	34.159	-50.457	1.00	49.76	C
ATOM	415	H	VAL	A	80	-2.351	31.446	-50.977	1.00	41.50	H
ATOM	416	HA	VAL	A	80	-0.808	32.304	-48.747	1.00	43.75	H
ATOM	417	HB	VAL	A	80	-3.467	33.242	-49.894	1.00	42.20	H
ATOM	418	HG11	VAL	A	80	-3.016	35.331	-48.522	1.00	51.14	H
ATOM	419	HG12	VAL	A	80	-3.526	33.967	-47.571	1.00	51.14	H
ATOM	420	HG13	VAL	A	80	-1.818	34.426	-47.614	1.00	51.14	H
ATOM	421	HG21	VAL	A	80	-2.137	35.043	-50.846	1.00	49.76	H
ATOM	422	HG22	VAL	A	80	-0.692	34.488	-50.013	1.00	49.76	H
ATOM	423	HG23	VAL	A	80	-1.376	33.531	-51.308	1.00	49.76	H
ATOM	424	N	VAL	A	81	-3.707	30.895	-48.000	1.00	43.35	N
ATOM	425	CA	VAL	A	81	-4.433	30.293	-46.885	1.00	46.33	C
ATOM	426	C	VAL	A	81	-3.641	29.131	-46.298	1.00	48.79	C
ATOM	427	O	VAL	A	81	-3.562	28.969	-45.075	1.00	46.75	O
ATOM	428	CB	VAL	A	81	-5.839	29.855	-47.338	1.00	47.71	C
ATOM	429	CG1	VAL	A	81	-6.517	29.026	-46.256	1.00	50.80	C
ATOM	430	CG2	VAL	A	81	-6.684	31.072	-47.684	1.00	44.41	C
ATOM	431	H	VAL	A	81	-4.120	30.831	-48.923	1.00	43.35	H
ATOM	432	HA	VAL	A	81	-4.545	31.045	-46.101	1.00	46.33	H
ATOM	433	HB	VAL	A	81	-5.751	29.236	-48.233	1.00	47.71	H
ATOM	434	HG11	VAL	A	81	-7.544	28.793	-46.539	1.00	50.80	H
ATOM	435	HG12	VAL	A	81	-6.028	28.071	-46.077	1.00	50.80	H

```
ATOM    436 HG13 VAL A  81      -6.556  29.563 -45.308  1.00 50.80           H
ATOM    437 HG21 VAL A  81      -7.656  30.772 -48.077  1.00 44.41           H
ATOM    438 HG22 VAL A  81      -6.862  31.691 -46.804  1.00 44.41           H
ATOM    439 HG23 VAL A  81      -6.218  31.709 -48.434  1.00 44.41           H
ATOM    440  N   GLY A  82      -3.030  28.314 -47.159  1.00 42.80           N
ATOM    441  CA  GLY A  82      -2.248  27.191 -46.670  1.00 41.72           C
ATOM    442  C   GLY A  82      -1.018  27.621 -45.892  1.00 45.69           C
ATOM    443  O   GLY A  82      -0.716  27.061 -44.835  1.00 41.53           O
ATOM    444  H   GLY A  82      -3.110  28.444 -48.160  1.00 42.80           H
ATOM    445  HA2 GLY A  82      -2.862  26.506 -46.083  1.00 41.72           H
ATOM    446  HA3 GLY A  82      -1.918  26.666 -47.552  1.00 41.72           H
ATOM    447  N   LEU A  83      -0.292  28.617 -46.401  1.00 37.86           N
ATOM    448  CA  LEU A  83       0.906  29.076 -45.706  1.00 41.27           C
ATOM    449  C   LEU A  83       0.556  29.765 -44.393  1.00 45.51           C
ATOM    450  O   LEU A  83       1.214  29.535 -43.373  1.00 45.79           O
ATOM    451  CB  LEU A  83       1.716  30.008 -46.609  1.00 41.52           C
ATOM    452  CG  LEU A  83       2.402  29.330 -47.800  1.00 50.52           C
ATOM    453  CD1 LEU A  83       3.148  30.345 -48.658  1.00 48.07           C
ATOM    454  CD2 LEU A  83       3.343  28.233 -47.325  1.00 50.51           C
ATOM    455  H   LEU A  83      -0.533  29.057 -47.281  1.00 37.86           H
ATOM    456  HA  LEU A  83       1.517  28.211 -45.444  1.00 41.27           H
ATOM    457  HB2 LEU A  83       1.066  30.807 -46.971  1.00 41.52           H
ATOM    458  HB3 LEU A  83       2.486  30.504 -46.015  1.00 41.52           H
ATOM    459  HG  LEU A  83       1.651  28.856 -48.429  1.00 50.52           H
ATOM    460 HD11 LEU A  83       3.207  30.009 -49.692  1.00 48.07           H
ATOM    461 HD12 LEU A  83       2.661  31.321 -48.654  1.00 48.07           H
ATOM    462 HD13 LEU A  83       4.171  30.489 -48.309  1.00 48.07           H
ATOM    463 HD21 LEU A  83       4.112  28.012 -48.066  1.00 50.51           H
ATOM    464 HD22 LEU A  83       3.850  28.514 -46.403  1.00 50.51           H
ATOM    465 HD23 LEU A  83       2.812  27.306 -47.128  1.00 50.51           H
ATOM    466  N   PHE A  84      -0.485  30.600 -44.392  1.00 46.18           N
ATOM    467  CA  PHE A  84      -0.850  31.321 -43.176  1.00 51.28           C
ATOM    468  C   PHE A  84      -1.341  30.369 -42.092  1.00 49.44           C
ATOM    469  O   PHE A  84      -0.913  30.457 -40.936  1.00 52.38           O
ATOM    470  CB  PHE A  84      -1.915  32.374 -43.481  1.00 52.21           C
ATOM    471  CG  PHE A  84      -2.538  32.970 -42.251  1.00 63.43           C
ATOM    472  CD1 PHE A  84      -1.862  33.926 -41.513  1.00 71.04           C
ATOM    473  CD2 PHE A  84      -3.794  32.568 -41.827  1.00 66.16           C
ATOM    474  CE1 PHE A  84      -2.426  34.472 -40.377  1.00 73.24           C
ATOM    475  CE2 PHE A  84      -4.364  33.112 -40.692  1.00 69.35           C
```

ATOM 476 CZ PHE A 84 -3.679 34.067 -39.968 1.00 70.14 C
ATOM 477 H PHE A 84 -1.021 30.780 -45.232 1.00 46.18 H
ATOM 478 HA PHE A 84 0.033 31.837 -42.791 1.00 51.28 H
ATOM 479 HB2 PHE A 84 -1.476 33.177 -44.075 1.00 52.21 H
ATOM 480 HB3 PHE A 84 -2.704 31.942 -44.099 1.00 52.21 H
ATOM 481 HD1 PHE A 84 -0.879 34.249 -41.823 1.00 71.04 H
ATOM 482 HD2 PHE A 84 -4.338 31.818 -42.383 1.00 66.16 H
ATOM 483 HE1 PHE A 84 -1.886 35.216 -39.809 1.00 73.24 H
ATOM 484 HE2 PHE A 84 -5.343 32.789 -40.371 1.00 69.35 H
ATOM 485 HZ PHE A 84 -4.121 34.492 -39.079 1.00 70.14 H
ATOM 486 N GLY A 85 -2.248 29.458 -42.447 1.00 44.61 N
ATOM 487 CA GLY A 85 -2.815 28.569 -41.447 1.00 41.89 C
ATOM 488 C GLY A 85 -1.787 27.641 -40.830 1.00 45.37 C
ATOM 489 O GLY A 85 -1.828 27.368 -39.627 1.00 46.18 O
ATOM 490 H GLY A 85 -2.584 29.392 -43.400 1.00 44.61 H
ATOM 491 HA2 GLY A 85 -3.309 29.150 -40.666 1.00 41.89 H
ATOM 492 HA3 GLY A 85 -3.586 27.962 -41.922 1.00 41.89 H
ATOM 493 N ASN A 86 -0.845 27.154 -41.636 1.00 37.27 N
ATOM 494 CA ASN A 86 0.118 26.189 -41.118 1.00 35.97 C
ATOM 495 C ASN A 86 1.270 26.867 -40.387 1.00 40.23 C
ATOM 496 O ASN A 86 1.787 26.321 -39.407 1.00 40.16 O
ATOM 497 CB ASN A 86 0.632 25.305 -42.252 1.00 34.90 C
ATOM 498 CG ASN A 86 -0.409 24.311 -42.716 1.00 39.72 C
ATOM 499 ND2 ASN A 86 -0.952 24.530 -43.908 1.00 36.36 N
ATOM 500 OD1 ASN A 86 -0.732 23.361 -42.002 1.00 38.34 O
ATOM 501 H ASN A 86 -0.813 27.386 -42.620 1.00 37.27 H
ATOM 502 HA ASN A 86 -0.395 25.534 -40.408 1.00 35.97 H
ATOM 503 HB2 ASN A 86 1.007 25.906 -43.082 1.00 34.90 H
ATOM 504 HB3 ASN A 86 1.477 24.724 -41.890 1.00 34.90 H
ATOM 505 HD21 ASN A 86 -0.701 25.355 -44.441 1.00 36.36 H
ATOM 506 HD22 ASN A 86 -1.669 23.907 -44.258 1.00 36.36 H
ATOM 507 N PHE A 87 1.689 28.051 -40.840 1.00 39.11 N
ATOM 508 CA PHE A 87 2.687 28.795 -40.082 1.00 38.59 C
ATOM 509 C PHE A 87 2.126 29.275 -38.750 1.00 42.93 C
ATOM 510 O PHE A 87 2.870 29.385 -37.769 1.00 44.51 O
ATOM 511 CB PHE A 87 3.210 29.976 -40.899 1.00 44.99 C
ATOM 512 CG PHE A 87 4.423 29.648 -41.725 1.00 59.02 C
ATOM 513 CD1 PHE A 87 4.295 29.046 -42.965 1.00 56.30 C
ATOM 514 CD2 PHE A 87 5.694 29.940 -41.256 1.00 71.91 C
ATOM 515 CE1 PHE A 87 5.410 28.743 -43.725 1.00 58.43 C

ATOM	516	CE2	PHE	A	87	6.813	29.640	-42.011	1.00	76.67	C
ATOM	517	CZ	PHE	A	87	6.670	29.040	-43.246	1.00	69.33	C
ATOM	518	H	PHE	A	87	1.283	28.490	-41.657	1.00	39.11	H
ATOM	519	HA	PHE	A	87	3.527	28.150	-39.834	1.00	38.59	H
ATOM	520	HB2	PHE	A	87	2.432	30.425	-41.511	1.00	44.99	H
ATOM	521	HB3	PHE	A	87	3.508	30.786	-40.230	1.00	44.99	H
ATOM	522	HD1	PHE	A	87	3.343	28.744	-43.352	1.00	56.30	H
ATOM	523	HD2	PHE	A	87	5.821	30.405	-40.289	1.00	71.91	H
ATOM	524	HE1	PHE	A	87	5.302	28.258	-44.683	1.00	58.43	H
ATOM	525	HE2	PHE	A	87	7.798	29.871	-41.633	1.00	76.67	H
ATOM	526	HZ	PHE	A	87	7.543	28.800	-43.835	1.00	69.33	H
ATOM	527	N	LEU	A	88	0.823	29.562	-38.697	1.00	38.67	N
ATOM	528	CA	LEU	A	88	0.198	29.929	-37.432	1.00	44.99	C
ATOM	529	C	LEU	A	88	0.206	28.759	-36.456	1.00	45.56	C
ATOM	530	O	LEU	A	88	0.457	28.944	-35.259	1.00	48.49	O
ATOM	531	CB	LEU	A	88	-1.229	30.415	-37.679	1.00	41.93	C
ATOM	532	CG	LEU	A	88	-2.039	30.778	-36.434	1.00	47.36	C
ATOM	533	CD1	LEU	A	88	-1.340	31.877	-35.644	1.00	47.99	C
ATOM	534	CD2	LEU	A	88	-3.447	31.199	-36.820	1.00	48.52	C
ATOM	535	H	LEU	A	88	0.233	29.506	-39.517	1.00	38.67	H
ATOM	536	HA	LEU	A	88	0.774	30.745	-36.992	1.00	44.99	H
ATOM	537	HB2	LEU	A	88	-1.195	31.281	-38.343	1.00	41.93	H
ATOM	538	HB3	LEU	A	88	-1.770	29.646	-38.231	1.00	41.93	H
ATOM	539	HG	LEU	A	88	-2.144	29.902	-35.793	1.00	47.36	H
ATOM	540	HD11	LEU	A	88	-2.045	32.570	-35.185	1.00	47.99	H
ATOM	541	HD12	LEU	A	88	-0.748	31.454	-34.833	1.00	47.99	H
ATOM	542	HD13	LEU	A	88	-0.667	32.471	-36.263	1.00	47.99	H
ATOM	543	HD21	LEU	A	88	-4.180	30.794	-36.122	1.00	48.52	H
ATOM	544	HD22	LEU	A	88	-3.570	32.283	-36.824	1.00	48.52	H
ATOM	545	HD23	LEU	A	88	-3.724	30.852	-37.816	1.00	48.52	H
ATOM	546	N	VAL	A	89	-0.065	27.548	-36.950	1.00	39.78	N
ATOM	547	CA	VAL	A	89	-0.005	26.359	-36.104	1.00	39.03	C
ATOM	548	C	VAL	A	89	1.412	26.148	-35.588	1.00	46.81	C
ATOM	549	O	VAL	A	89	1.630	25.887	-34.398	1.00	44.00	O
ATOM	550	CB	VAL	A	89	-0.509	25.125	-36.874	1.00	36.04	C
ATOM	551	CG1	VAL	A	89	-0.251	23.861	-36.072	1.00	32.94	C
ATOM	552	CG2	VAL	A	89	-1.987	25.266	-37.193	1.00	34.65	C
ATOM	553	H	VAL	A	89	-0.292	27.420	-37.927	1.00	39.78	H
ATOM	554	HA	VAL	A	89	-0.656	26.522	-35.244	1.00	39.03	H
ATOM	555	HB	VAL	A	89	0.025	25.044	-37.821	1.00	36.04	H

ATOM 556 HG11 VAL A 89 -0.762 23.012 -36.527 1.00 32.94 H
ATOM 557 HG12 VAL A 89 0.804 23.588 -36.035 1.00 32.94 H
ATOM 558 HG13 VAL A 89 -0.620 23.946 -35.049 1.00 32.94 H
ATOM 559 HG21 VAL A 89 -2.302 24.427 -37.810 1.00 34.65 H
ATOM 560 HG22 VAL A 89 -2.577 25.224 -36.277 1.00 34.65 H
ATOM 561 HG23 VAL A 89 -2.257 26.197 -37.671 1.00 34.65 H
ATOM 562 N MET A 90 2.399 26.252 -36.481 1.00 38.82 N
ATOM 563 CA MET A 90 3.782 26.052 -36.068 1.00 44.65 C
ATOM 564 C MET A 90 4.234 27.131 -35.098 1.00 43.21 C
ATOM 565 O MET A 90 5.022 26.855 -34.187 1.00 42.84 O
ATOM 566 CB MET A 90 4.696 26.012 -37.292 1.00 41.83 C
ATOM 567 CG MET A 90 4.554 24.741 -38.115 1.00 45.54 C
ATOM 568 SD MET A 90 5.740 24.659 -39.469 1.00 48.39 S
ATOM 569 CE MET A 90 4.991 25.786 -40.641 1.00 42.55 C
ATOM 570 H MET A 90 2.213 26.448 -37.456 1.00 38.82 H
ATOM 571 HA MET A 90 3.863 25.094 -35.550 1.00 44.65 H
ATOM 572 HB2 MET A 90 4.506 26.883 -37.921 1.00 41.83 H
ATOM 573 HB3 MET A 90 5.738 26.096 -36.978 1.00 41.83 H
ATOM 574 HG2 MET A 90 4.707 23.865 -37.486 1.00 45.54 H
ATOM 575 HG3 MET A 90 3.549 24.652 -38.521 1.00 45.54 H
ATOM 576 HE1 MET A 90 5.488 25.694 -41.606 1.00 42.55 H
ATOM 577 HE2 MET A 90 3.935 25.564 -40.783 1.00 42.55 H
ATOM 578 HE3 MET A 90 5.118 26.811 -40.302 1.00 42.55 H
ATOM 579 N TYR A 91 3.741 28.359 -35.266 1.00 42.99 N
ATOM 580 CA TYR A 91 4.078 29.423 -34.328 1.00 46.23 C
ATOM 581 C TYR A 91 3.500 29.138 -32.947 1.00 48.28 C
ATOM 582 O TYR A 91 4.205 29.241 -31.937 1.00 46.72 O
ATOM 583 CB TYR A 91 3.578 30.769 -34.854 1.00 47.66 C
ATOM 584 CG TYR A 91 3.588 31.869 -33.814 1.00 54.06 C
ATOM 585 CD1 TYR A 91 4.770 32.507 -33.459 1.00 60.28 C
ATOM 586 CD2 TYR A 91 2.414 32.270 -33.187 1.00 56.57 C
ATOM 587 CE1 TYR A 91 4.783 33.512 -32.506 1.00 61.39 C
ATOM 588 CE2 TYR A 91 2.418 33.272 -32.232 1.00 54.24 C
ATOM 589 CZ TYR A 91 3.604 33.889 -31.897 1.00 59.64 C
ATOM 590 OH TYR A 91 3.611 34.888 -30.949 1.00 69.59 O
ATOM 591 H TYR A 91 3.125 28.582 -36.038 1.00 42.99 H
ATOM 592 HA TYR A 91 5.165 29.481 -34.239 1.00 46.23 H
ATOM 593 HB2 TYR A 91 4.169 31.078 -35.717 1.00 47.66 H
ATOM 594 HB3 TYR A 91 2.555 30.667 -35.218 1.00 47.66 H
ATOM 595 HD1 TYR A 91 5.697 32.215 -33.930 1.00 60.28 H

ATOM 596 HD2 TYR A 91 1.479 31.793 -33.442 1.00 56.57 H
ATOM 597 HE1 TYR A 91 5.712 33.995 -32.242 1.00 61.39 H
ATOM 598 HE2 TYR A 91 1.493 33.564 -31.757 1.00 54.24 H
ATOM 599 HH TYR A 91 2.735 35.106 -30.625 1.00 69.59 H
ATOM 600 N VAL A 92 2.215 28.781 -32.884 1.00 46.23 N
ATOM 601 CA VAL A 92 1.576 28.514 -31.597 1.00 52.27 C
ATOM 602 C VAL A 92 2.257 27.347 -30.894 1.00 50.69 C
ATOM 603 O VAL A 92 2.426 27.353 -29.668 1.00 50.12 O
ATOM 604 CB VAL A 92 0.068 28.262 -31.791 1.00 52.04 C
ATOM 605 CG1 VAL A 92 -0.559 27.730 -30.510 1.00 57.30 C
ATOM 606 CG2 VAL A 92 -0.628 29.541 -32.227 1.00 52.61 C
ATOM 607 H VAL A 92 1.647 28.706 -33.719 1.00 46.23 H
ATOM 608 HA VAL A 92 1.709 29.405 -30.985 1.00 52.27 H
ATOM 609 HB VAL A 92 -0.066 27.522 -32.581 1.00 52.04 H
ATOM 610 HG11 VAL A 92 -1.646 27.711 -30.597 1.00 57.30 H
ATOM 611 HG12 VAL A 92 -0.263 26.704 -30.289 1.00 57.30 H
ATOM 612 HG13 VAL A 92 -0.319 28.353 -29.648 1.00 57.30 H
ATOM 613 HG21 VAL A 92 -1.669 29.350 -32.490 1.00 52.61 H
ATOM 614 HG22 VAL A 92 -0.621 30.283 -31.428 1.00 52.61 H
ATOM 615 HG23 VAL A 92 -0.155 30.002 -33.090 1.00 52.61 H
ATOM 616 N ILE A 93 2.673 26.335 -31.657 1.00 43.19 N
ATOM 617 CA ILE A 93 3.352 25.188 -31.062 1.00 44.29 C
ATOM 618 C ILE A 93 4.719 25.595 -30.524 1.00 53.04 C
ATOM 619 O ILE A 93 5.080 25.269 -29.386 1.00 56.03 O
ATOM 620 CB ILE A 93 3.465 24.041 -32.082 1.00 47.47 C
ATOM 621 CG1 ILE A 93 2.103 23.377 -32.283 1.00 48.83 C
ATOM 622 CG2 ILE A 93 4.500 23.025 -31.630 1.00 53.89 C
ATOM 623 CD1 ILE A 93 2.122 22.246 -33.279 1.00 58.34 C
ATOM 624 H ILE A 93 2.517 26.329 -32.657 1.00 43.19 H
ATOM 625 HA ILE A 93 2.694 24.926 -30.268 1.00 44.29 H
ATOM 626 HB ILE A 93 3.792 24.448 -33.041 1.00 47.47 H
ATOM 627 HG12 ILE A 93 1.670 23.044 -31.339 1.00 48.83 H
ATOM 628 HG13 ILE A 93 1.476 24.150 -32.694 1.00 48.83 H
ATOM 629 HG21 ILE A 93 4.534 22.194 -32.326 1.00 53.89 H
ATOM 630 HG22 ILE A 93 5.521 23.406 -31.625 1.00 53.89 H
ATOM 631 HG23 ILE A 93 4.273 22.624 -30.641 1.00 53.89 H
ATOM 632 HD11 ILE A 93 1.111 22.096 -33.636 1.00 58.34 H
ATOM 633 HD12 ILE A 93 2.749 22.469 -34.142 1.00 58.34 H
ATOM 634 HD13 ILE A 93 2.450 21.310 -32.828 1.00 58.34 H
ATOM 635 N VAL A 94 5.497 26.318 -31.330 1.00 50.31 N

```
ATOM    636  CA  VAL A  94       6.842  26.705 -30.915  1.00 56.10           C
ATOM    637  C   VAL A  94       6.787  27.708 -29.767  1.00 62.86           C
ATOM    638  O   VAL A  94       7.618  27.668 -28.852  1.00 62.62           O
ATOM    639  CB  VAL A  94       7.626  27.252 -32.122  1.00 61.86           C
ATOM    640  CG1 VAL A  94       8.905  27.944 -31.674  1.00 66.14           C
ATOM    641  CG2 VAL A  94       7.945  26.125 -33.092  1.00 55.28           C
ATOM    642  H   VAL A  94       5.197  26.580 -32.261  1.00 50.31           H
ATOM    643  HA  VAL A  94       7.361  25.816 -30.548  1.00 56.10           H
ATOM    644  HB  VAL A  94       7.011  27.985 -32.647  1.00 61.86           H
ATOM    645 HG11 VAL A  94       9.533  28.178 -32.534  1.00 66.14           H
ATOM    646 HG12 VAL A  94       8.719  28.895 -31.174  1.00 66.14           H
ATOM    647 HG13 VAL A  94       9.496  27.313 -31.009  1.00 66.14           H
ATOM    648 HG21 VAL A  94       8.392  26.511 -34.009  1.00 55.28           H
ATOM    649 HG22 VAL A  94       8.649  25.416 -32.655  1.00 55.28           H
ATOM    650 HG23 VAL A  94       7.060  25.558 -33.375  1.00 55.28           H
ATOM    651  N   ARG A  95       5.798  28.601 -29.778  1.00 62.33           N
ATOM    652  CA  ARG A  95       5.728  29.674 -28.792  1.00 67.71           C
ATOM    653  C   ARG A  95       5.049  29.253 -27.492  1.00 67.29           C
ATOM    654  O   ARG A  95       5.502  29.641 -26.410  1.00 73.63           O
ATOM    655  CB  ARG A  95       4.996  30.883 -29.386  1.00 66.52           C
ATOM    656  CG  ARG A  95       4.871  32.079 -28.448  1.00 73.97           C
ATOM    657  CD  ARG A  95       6.220  32.724 -28.179  1.00 73.83           C
ATOM    658  NE  ARG A  95       6.097  33.925 -27.358  1.00 86.17           N
ATOM    659  CZ  ARG A  95       6.225  33.943 -26.034  1.00 93.15           C
ATOM    660  NH1 ARG A  95       6.482  32.822 -25.375  1.00 92.13           N1+
ATOM    661  NH2 ARG A  95       6.097  35.084 -25.369  1.00 94.95           N1+
ATOM    662  H   ARG A  95       5.129  28.624 -30.539  1.00 62.33           H
ATOM    663  HA  ARG A  95       6.745  29.985 -28.552  1.00 67.71           H
ATOM    664  HB2 ARG A  95       5.500  31.195 -30.303  1.00 66.52           H
ATOM    665  HB3 ARG A  95       3.990  30.585 -29.687  1.00 66.52           H
ATOM    666  HG2 ARG A  95       4.280  32.802 -29.011  1.00 73.97           H
ATOM    667  HG3 ARG A  95       4.305  31.901 -27.534  1.00 73.97           H
ATOM    668  HD2 ARG A  95       7.019  32.050 -27.875  1.00 73.83           H
ATOM    669  HD3 ARG A  95       6.548  33.137 -29.134  1.00 73.83           H
ATOM    670  HE  ARG A  95       5.852  34.777 -27.842  1.00 86.17           H
ATOM    671 HH11 ARG A  95       6.565  31.951 -25.880  1.00 92.13           H
ATOM    672 HH12 ARG A  95       6.568  32.825 -24.368  1.00 92.13           H
ATOM    673 HH21 ARG A  95       5.897  35.942 -25.863  1.00 94.95           H
ATOM    674 HH22 ARG A  95       6.195  35.108 -24.365  1.00 94.95           H
ATOM    675  N   TYR A  96       3.974  28.467 -27.564  1.00 66.56           N
```

ATOM 676 CA TYR A 96 3.150 28.198 -26.389 1.00 65.56 C
ATOM 677 C TYR A 96 3.161 26.733 -25.971 1.00 68.07 C
ATOM 678 O TYR A 96 3.553 26.431 -24.839 1.00 70.19 O
ATOM 679 CB TYR A 96 1.714 28.678 -26.644 1.00 63.12 C
ATOM 680 CG TYR A 96 1.607 30.169 -26.878 1.00 74.01 C
ATOM 681 CD1 TYR A 96 1.701 31.066 -25.819 1.00 77.33 C
ATOM 682 CD2 TYR A 96 1.411 30.681 -28.155 1.00 76.49 C
ATOM 683 CE1 TYR A 96 1.605 32.430 -26.026 1.00 77.55 C
ATOM 684 CE2 TYR A 96 1.312 32.045 -28.373 1.00 78.90 C
ATOM 685 CZ TYR A 96 1.410 32.914 -27.304 1.00 84.01 C
ATOM 686 OH TYR A 96 1.313 34.271 -27.513 1.00 84.67 O
ATOM 687 H TYR A 96 3.628 28.135 -28.456 1.00 66.56 H
ATOM 688 HA TYR A 96 3.495 28.752 -25.514 1.00 65.56 H
ATOM 689 HB2 TYR A 96 1.267 28.149 -27.486 1.00 63.12 H
ATOM 690 HB3 TYR A 96 1.087 28.438 -25.784 1.00 63.12 H
ATOM 691 HD1 TYR A 96 1.852 30.695 -24.816 1.00 77.33 H
ATOM 692 HD2 TYR A 96 1.327 30.005 -28.987 1.00 76.49 H
ATOM 693 HE1 TYR A 96 1.681 33.111 -25.191 1.00 77.55 H
ATOM 694 HE2 TYR A 96 1.160 32.419 -29.375 1.00 78.90 H
ATOM 695 HH TYR A 96 1.102 34.500 -28.419 1.00 84.67 H
ATOM 696 N THR A 97 2.743 25.807 -26.840 1.00 67.89 N
ATOM 697 CA THR A 97 2.534 24.429 -26.399 1.00 68.61 C
ATOM 698 C THR A 97 3.847 23.682 -26.184 1.00 70.65 C
ATOM 699 O THR A 97 3.906 22.780 -25.339 1.00 65.79 O
ATOM 700 CB THR A 97 1.654 23.678 -27.400 1.00 67.58 C
ATOM 701 CG2 THR A 97 0.374 24.457 -27.669 1.00 65.85 C
ATOM 702 OG1 THR A 97 2.366 23.496 -28.629 1.00 66.39 O
ATOM 703 H THR A 97 2.460 26.048 -27.780 1.00 67.89 H
ATOM 704 HA THR A 97 2.004 24.440 -25.443 1.00 68.61 H
ATOM 705 HB THR A 97 1.390 22.721 -26.965 1.00 67.58 H
ATOM 706 HG1 THR A 97 1.768 23.103 -29.270 1.00 66.39 H
ATOM 707 HG21 THR A 97 -0.352 23.845 -28.205 1.00 65.85 H
ATOM 708 HG22 THR A 97 -0.081 24.776 -26.738 1.00 65.85 H
ATOM 709 HG23 THR A 97 0.551 25.350 -28.271 1.00 65.85 H
ATOM 710 N LYS A 98 4.889 24.019 -26.945 1.00 74.95 N
ATOM 711 CA LYS A 98 6.286 23.602 -26.778 1.00 79.85 C
ATOM 712 C LYS A 98 6.598 22.185 -27.260 1.00 76.37 C
ATOM 713 O LYS A 98 7.750 21.752 -27.106 1.00 90.01 O
ATOM 714 CB LYS A 98 6.767 23.721 -25.322 1.00 79.28 C
ATOM 715 CG LYS A 98 6.857 25.148 -24.813 1.00 85.42 C

```
ATOM    716  CD  LYS A  98       7.794  25.976 -25.676  1.00 87.92           C
ATOM    717  CE  LYS A  98       8.091  27.321 -25.035  1.00 93.16           C
ATOM    718  NZ  LYS A  98       6.847  28.080 -24.727  1.00 94.56           N1+
ATOM    719  H   LYS A  98       4.742  24.702 -27.679  1.00 74.95           H
ATOM    720  HA  LYS A  98       6.875  24.237 -27.436  1.00 79.85           H
ATOM    721  HB2 LYS A  98       6.170  23.123 -24.638  1.00 79.28           H
ATOM    722  HB3 LYS A  98       7.774  23.314 -25.220  1.00 79.28           H
ATOM    723  HG2 LYS A  98       5.865  25.588 -24.789  1.00 85.42           H
ATOM    724  HG3 LYS A  98       7.212  25.133 -23.782  1.00 85.42           H
ATOM    725  HD2 LYS A  98       8.726  25.441 -25.866  1.00 87.92           H
ATOM    726  HD3 LYS A  98       7.339  26.175 -26.645  1.00 87.92           H
ATOM    727  HE2 LYS A  98       8.644  27.175 -24.107  1.00 93.16           H
ATOM    728  HE3 LYS A  98       8.721  27.923 -25.691  1.00 93.16           H
ATOM    729  HZ1 LYS A  98       6.366  28.331 -25.583  1.00 94.56           H
ATOM    730  HZ2 LYS A  98       6.236  27.516 -24.154  1.00 94.56           H
ATOM    731  HZ3 LYS A  98       7.084  28.926 -24.231  1.00 94.56           H
ATOM    732  N   MET A  99       5.636  21.448 -27.822  1.00 48.25           N
ATOM    733  CA  MET A  99       5.898  20.138 -28.429  1.00 58.94           C
ATOM    734  C   MET A  99       6.431  19.128 -27.412  1.00 59.86           C
ATOM    735  O   MET A  99       7.364  18.373 -27.692  1.00 54.45           O
ATOM    736  CB  MET A  99       6.861  20.259 -29.614  1.00 66.88           C
ATOM    737  CG  MET A  99       6.253  19.901 -30.958  1.00 65.96           C
ATOM    738  SD  MET A  99       7.507  19.743 -32.244  1.00 71.53           S
ATOM    739  CE  MET A  99       8.279  21.358 -32.162  1.00 66.71           C
ATOM    740  H   MET A  99       4.706  21.829 -27.924  1.00 48.25           H
ATOM    741  HA  MET A  99       4.939  19.757 -28.775  1.00 58.94           H
ATOM    742  HB2 MET A  99       7.166  21.300 -29.712  1.00 66.88           H
ATOM    743  HB3 MET A  99       7.814  19.743 -29.505  1.00 66.88           H
ATOM    744  HG2 MET A  99       5.746  18.940 -30.880  1.00 65.96           H
ATOM    745  HG3 MET A  99       5.499  20.618 -31.253  1.00 65.96           H
ATOM    746  HE1 MET A  99       8.956  21.484 -33.007  1.00 66.71           H
ATOM    747  HE2 MET A  99       7.528  22.146 -32.211  1.00 66.71           H
ATOM    748  HE3 MET A  99       8.859  21.474 -31.247  1.00 66.71           H
ATOM    749  N   LYS A 100       5.831  19.106 -26.224  1.00 56.26           N
ATOM    750  CA  LYS A 100       6.210  18.133 -25.208  1.00 57.17           C
ATOM    751  C   LYS A 100       5.427  16.832 -25.310  1.00 51.33           C
ATOM    752  O   LYS A 100       5.844  15.827 -24.724  1.00 53.76           O
ATOM    753  CB  LYS A 100       6.015  18.721 -23.807  1.00 60.34           C
ATOM    754  CG  LYS A 100       6.864  19.948 -23.515  1.00 67.29           C
ATOM    755  CD  LYS A 100       6.481  20.567 -22.181  1.00 71.75           C
```

ATOM 756 CE LYS A 100 7.309 21.806 -21.884 1.00 83.75 C
ATOM 757 NZ LYS A 100 8.754 21.483 -21.722 1.00 92.27 N1+
ATOM 758 H LYS A 100 5.090 19.755 -26.002 1.00 56.26 H
ATOM 759 HA LYS A 100 7.269 17.878 -25.294 1.00 57.17 H
ATOM 760 HB2 LYS A 100 4.961 18.976 -23.677 1.00 60.34 H
ATOM 761 HB3 LYS A 100 6.236 17.965 -23.051 1.00 60.34 H
ATOM 762 HG2 LYS A 100 7.916 19.661 -23.525 1.00 67.29 H
ATOM 763 HG3 LYS A 100 6.739 20.691 -24.303 1.00 67.29 H
ATOM 764 HD2 LYS A 100 5.425 20.840 -22.192 1.00 71.75 H
ATOM 765 HD3 LYS A 100 6.601 19.837 -21.379 1.00 71.75 H
ATOM 766 HE2 LYS A 100 7.196 22.549 -22.671 1.00 83.75 H
ATOM 767 HE3 LYS A 100 6.958 22.269 -20.962 1.00 83.75 H
ATOM 768 HZ1 LYS A 100 9.108 21.081 -22.578 1.00 92.27 H
ATOM 769 HZ2 LYS A 100 9.268 22.326 -21.510 1.00 92.27 H
ATOM 770 HZ3 LYS A 100 8.870 20.822 -20.967 1.00 92.27 H
ATOM 771 N THR A 101 4.315 16.826 -26.035 1.00 44.30 N
ATOM 772 CA THR A 101 3.418 15.683 -26.097 1.00 41.13 C
ATOM 773 C THR A 101 3.440 15.060 -27.485 1.00 44.80 C
ATOM 774 O THR A 101 3.807 15.699 -28.474 1.00 38.56 O
ATOM 775 CB THR A 101 1.986 16.092 -25.739 1.00 49.95 C
ATOM 776 CG2 THR A 101 1.935 16.676 -24.334 1.00 46.85 C
ATOM 777 OG1 THR A 101 1.523 17.074 -26.675 1.00 49.70 O
ATOM 778 H THR A 101 4.018 17.655 -26.528 1.00 44.30 H
ATOM 779 HA THR A 101 3.716 14.902 -25.394 1.00 41.13 H
ATOM 780 HB THR A 101 1.321 15.228 -25.788 1.00 49.95 H
ATOM 781 HG1 THR A 101 0.676 17.410 -26.370 1.00 49.70 H
ATOM 782 HG21 THR A 101 0.907 16.896 -24.044 1.00 46.85 H
ATOM 783 HG22 THR A 101 2.335 15.974 -23.602 1.00 46.85 H
ATOM 784 HG23 THR A 101 2.500 17.605 -24.249 1.00 46.85 H
ATOM 785 N ALA A 102 3.028 13.792 -27.542 1.00 42.68 N
ATOM 786 CA ALA A 102 2.952 13.092 -28.819 1.00 41.40 C
ATOM 787 C ALA A 102 2.004 13.795 -29.782 1.00 40.52 C
ATOM 788 O ALA A 102 2.279 13.881 -30.985 1.00 42.55 O
ATOM 789 CB ALA A 102 2.513 11.644 -28.596 1.00 35.31 C
ATOM 790 H ALA A 102 2.740 13.291 -26.714 1.00 42.68 H
ATOM 791 HA ALA A 102 3.945 13.083 -29.269 1.00 41.40 H
ATOM 792 HB1 ALA A 102 2.380 11.101 -29.531 1.00 35.31 H
ATOM 793 HB2 ALA A 102 3.229 11.107 -27.988 1.00 35.31 H
ATOM 794 HB3 ALA A 102 1.558 11.600 -28.070 1.00 35.31 H
ATOM 795 N THR A 103 0.884 14.312 -29.271 1.00 37.74 N

```
ATOM    796  CA  THR A 103      -0.106  14.943 -30.140  1.00 40.78           C
ATOM    797  C   THR A 103       0.461  16.193 -30.805  1.00 36.08           C
ATOM    798  O   THR A 103       0.288  16.400 -32.011  1.00 40.10           O
ATOM    799  CB  THR A 103      -1.368  15.280 -29.345  1.00 50.32           C
ATOM    800  CG2 THR A 103      -2.399  15.954 -30.241  1.00 50.62           C
ATOM    801  OG1 THR A 103      -1.927  14.076 -28.806  1.00 58.98           O
ATOM    802  H   THR A 103       0.661  14.227 -28.290  1.00 37.74           H
ATOM    803  HA  THR A 103      -0.371  14.234 -30.928  1.00 40.78           H
ATOM    804  HB  THR A 103      -1.131  15.938 -28.507  1.00 50.32           H
ATOM    805  HG1 THR A 103      -2.301  13.562 -29.526  1.00 58.98           H
ATOM    806 HG21 THR A 103      -3.360  16.015 -29.731  1.00 50.62           H
ATOM    807 HG22 THR A 103      -2.119  16.978 -30.485  1.00 50.62           H
ATOM    808 HG23 THR A 103      -2.556  15.409 -31.173  1.00 50.62           H
ATOM    809  N   ASN A 104       1.151  17.036 -30.033  1.00 37.62           N
ATOM    810  CA  ASN A 104       1.732  18.244 -30.610  1.00 39.54           C
ATOM    811  C   ASN A 104       2.881  17.921 -31.554  1.00 40.50           C
ATOM    812  O   ASN A 104       3.135  18.676 -32.500  1.00 40.82           O
ATOM    813  CB  ASN A 104       2.197  19.185 -29.501  1.00 37.80           C
ATOM    814  CG  ASN A 104       1.041  19.867 -28.812  1.00 46.39           C
ATOM    815  ND2 ASN A 104       1.266  20.341 -27.592  1.00 44.81           N
ATOM    816  OD1 ASN A 104      -0.049  19.965 -29.374  1.00 48.59           O
ATOM    817  H   ASN A 104       1.300  16.857 -29.049  1.00 37.62           H
ATOM    818  HA  ASN A 104       0.953  18.728 -31.202  1.00 39.54           H
ATOM    819  HB2 ASN A 104       2.824  18.656 -28.781  1.00 37.80           H
ATOM    820  HB3 ASN A 104       2.806  19.986 -29.925  1.00 37.80           H
ATOM    821 HD21 ASN A 104       2.170  20.237 -27.156  1.00 44.81           H
ATOM    822 HD22 ASN A 104       0.519  20.793 -27.085  1.00 44.81           H
ATOM    823  N   ILE A 105       3.580  16.812 -31.315  1.00 38.70           N
ATOM    824  CA  ILE A 105       4.627  16.381 -32.235  1.00 35.71           C
ATOM    825  C   ILE A 105       4.026  15.987 -33.581  1.00 37.17           C
ATOM    826  O   ILE A 105       4.590  16.291 -34.640  1.00 39.96           O
ATOM    827  CB  ILE A 105       5.439  15.236 -31.601  1.00 35.93           C
ATOM    828  CG1 ILE A 105       6.276  15.771 -30.435  1.00 37.32           C
ATOM    829  CG2 ILE A 105       6.322  14.552 -32.634  1.00 32.69           C
ATOM    830  CD1 ILE A 105       6.971  14.699 -29.631  1.00 39.06           C
ATOM    831  H   ILE A 105       3.372  16.218 -30.523  1.00 38.70           H
ATOM    832  HA  ILE A 105       5.304  17.218 -32.418  1.00 35.71           H
ATOM    833  HB  ILE A 105       4.755  14.487 -31.203  1.00 35.93           H
ATOM    834 HG12 ILE A 105       7.041  16.437 -30.835  1.00 37.32           H
ATOM    835 HG13 ILE A 105       5.698  16.407 -29.771  1.00 37.32           H
```

ATOM 836 HG21 ILE A 105 6.944 13.774 -32.202 1.00 32.69 H
ATOM 837 HG22 ILE A 105 5.736 14.023 -33.383 1.00 32.69 H
ATOM 838 HG23 ILE A 105 6.980 15.259 -33.141 1.00 32.69 H
ATOM 839 HD11 ILE A 105 7.545 15.139 -28.816 1.00 39.06 H
ATOM 840 HD12 ILE A 105 6.220 14.061 -29.172 1.00 39.06 H
ATOM 841 HD13 ILE A 105 7.612 14.060 -30.206 1.00 39.06 H
ATOM 842 N TYR A 106 2.867 15.320 -33.563 1.00 34.96 N
ATOM 843 CA TYR A 106 2.182 14.978 -34.807 1.00 34.32 C
ATOM 844 C TYR A 106 1.612 16.218 -35.486 1.00 40.41 C
ATOM 845 O TYR A 106 1.682 16.348 -36.715 1.00 33.25 O
ATOM 846 CB TYR A 106 1.065 13.968 -34.536 1.00 32.43 C
ATOM 847 CG TYR A 106 1.547 12.583 -34.155 1.00 38.02 C
ATOM 848 CD1 TYR A 106 2.618 11.994 -34.814 1.00 38.04 C
ATOM 849 CD2 TYR A 106 0.930 11.866 -33.135 1.00 32.34 C
ATOM 850 CE1 TYR A 106 3.061 10.730 -34.473 1.00 39.42 C
ATOM 851 CE2 TYR A 106 1.368 10.597 -32.781 1.00 31.03 C
ATOM 852 CZ TYR A 106 2.432 10.035 -33.456 1.00 34.94 C
ATOM 853 OH TYR A 106 2.878 8.780 -33.121 1.00 37.20 O
ATOM 854 H TYR A 106 2.427 15.054 -32.692 1.00 34.96 H
ATOM 855 HA TYR A 106 2.904 14.568 -35.513 1.00 34.32 H
ATOM 856 HB2 TYR A 106 0.383 14.346 -33.773 1.00 32.43 H
ATOM 857 HB3 TYR A 106 0.464 13.839 -35.438 1.00 32.43 H
ATOM 858 HD1 TYR A 106 3.160 12.514 -35.587 1.00 38.04 H
ATOM 859 HD2 TYR A 106 0.096 12.299 -32.602 1.00 32.34 H
ATOM 860 HE1 TYR A 106 4.022 10.487 -34.816 1.00 39.42 H
ATOM 861 HE2 TYR A 106 0.873 10.058 -31.986 1.00 31.03 H
ATOM 862 HH TYR A 106 2.377 8.378 -32.408 1.00 37.20 H
ATOM 863 N ILE A 107 1.036 17.134 -34.704 1.00 34.40 N
ATOM 864 CA ILE A 107 0.442 18.341 -35.273 1.00 40.03 C
ATOM 865 C ILE A 107 1.506 19.193 -35.953 1.00 42.24 C
ATOM 866 O ILE A 107 1.298 19.700 -37.062 1.00 37.11 O
ATOM 867 CB ILE A 107 -0.310 19.132 -34.187 1.00 41.04 C
ATOM 868 CG1 ILE A 107 -1.523 18.341 -33.690 1.00 41.09 C
ATOM 869 CG2 ILE A 107 -0.748 20.491 -34.717 1.00 38.68 C
ATOM 870 CD1 ILE A 107 -2.248 19.002 -32.521 1.00 41.14 C
ATOM 871 H ILE A 107 0.961 16.998 -33.704 1.00 34.40 H
ATOM 872 HA ILE A 107 -0.278 18.039 -36.037 1.00 40.03 H
ATOM 873 HB ILE A 107 0.367 19.299 -33.349 1.00 41.04 H
ATOM 874 HG12 ILE A 107 -2.233 18.263 -34.514 1.00 41.09 H
ATOM 875 HG13 ILE A 107 -1.301 17.307 -33.457 1.00 41.09 H

```
ATOM    876 HG21 ILE A 107      -1.195  21.100 -33.932  1.00 38.68           H
ATOM    877 HG22 ILE A 107       0.040  21.104 -35.142  1.00 38.68           H
ATOM    878 HG23 ILE A 107      -1.498  20.373 -35.499  1.00 38.68           H
ATOM    879 HD11 ILE A 107      -3.029  18.345 -32.138  1.00 41.14           H
ATOM    880 HD12 ILE A 107      -1.564  19.219 -31.701  1.00 41.14           H
ATOM    881 HD13 ILE A 107      -2.736  19.931 -32.806  1.00 41.14           H
ATOM    882  N   PHE A 108       2.664  19.361 -35.308  1.00 35.77           N
ATOM    883  CA  PHE A 108       3.728  20.150 -35.920  1.00 41.08           C
ATOM    884  C   PHE A 108       4.224  19.496 -37.203  1.00 41.68           C
ATOM    885  O   PHE A 108       4.457  20.180 -38.206  1.00 37.04           O
ATOM    886  CB  PHE A 108       4.887  20.347 -34.942  1.00 39.30           C
ATOM    887  CG  PHE A 108       5.969  21.251 -35.469  1.00 42.62           C
ATOM    888  CD1 PHE A 108       7.011  20.742 -36.229  1.00 46.55           C
ATOM    889  CD2 PHE A 108       5.933  22.615 -35.220  1.00 45.08           C
ATOM    890  CE1 PHE A 108       7.997  21.573 -36.726  1.00 43.28           C
ATOM    891  CE2 PHE A 108       6.920  23.452 -35.712  1.00 46.34           C
ATOM    892  CZ  PHE A 108       7.952  22.930 -36.466  1.00 44.51           C
ATOM    893  H   PHE A 108       2.830  18.956 -34.395  1.00 35.77           H
ATOM    894  HA  PHE A 108       3.326  21.134 -36.171  1.00 41.08           H
ATOM    895  HB2 PHE A 108       4.513  20.759 -34.008  1.00 39.30           H
ATOM    896  HB3 PHE A 108       5.325  19.383 -34.675  1.00 39.30           H
ATOM    897  HD1 PHE A 108       7.060  19.683 -36.439  1.00 46.55           H
ATOM    898  HD2 PHE A 108       5.127  23.037 -34.639  1.00 45.08           H
ATOM    899  HE1 PHE A 108       8.800  21.163 -37.320  1.00 43.28           H
ATOM    900  HE2 PHE A 108       6.878  24.512 -35.513  1.00 46.34           H
ATOM    901  HZ  PHE A 108       8.720  23.582 -36.857  1.00 44.51           H
ATOM    902  N   ASN A 109       4.399  18.172 -37.187  1.00 36.64           N
ATOM    903  CA  ASN A 109       4.825  17.463 -38.389  1.00 38.44           C
ATOM    904  C   ASN A 109       3.816  17.627 -39.516  1.00 41.26           C
ATOM    905  O   ASN A 109       4.199  17.738 -40.687  1.00 40.10           O
ATOM    906  CB  ASN A 109       5.035  15.980 -38.071  1.00 39.64           C
ATOM    907  CG  ASN A 109       5.900  15.279 -39.097  1.00 38.92           C
ATOM    908  ND2 ASN A 109       5.278  14.474 -39.949  1.00 39.15           N
ATOM    909  OD1 ASN A 109       7.117  15.445 -39.111  1.00 43.31           O
ATOM    910  H   ASN A 109       4.216  17.626 -36.356  1.00 36.64           H
ATOM    911  HA  ASN A 109       5.760  17.891 -38.760  1.00 38.44           H
ATOM    912  HB2 ASN A 109       5.571  15.896 -37.126  1.00 39.64           H
ATOM    913  HB3 ASN A 109       4.089  15.456 -37.923  1.00 39.64           H
ATOM    914 HD21 ASN A 109       4.275  14.338 -39.913  1.00 39.15           H
ATOM    915 HD22 ASN A 109       5.812  13.978 -40.646  1.00 39.15           H
```

```
ATOM 916 N LEU A 110 2.524 17.647 -39.183 1.00 40.63 N
ATOM 917 CA LEU A 110 1.499 17.839 -40.201 1.00 36.80 C
ATOM 918 C LEU A 110 1.527 19.260 -40.750 1.00 38.28 C
ATOM 919 O LEU A 110 1.437 19.466 -41.967 1.00 33.47 O
ATOM 920 CB LEU A 110 0.123 17.512 -39.620 1.00 34.33 C
ATOM 921 CG LEU A 110 -1.072 17.587 -40.575 1.00 42.10 C
ATOM 922 CD1 LEU A 110 -0.905 16.599 -41.722 1.00 37.24 C
ATOM 923 CD2 LEU A 110 -2.375 17.335 -39.831 1.00 36.08 C
ATOM 924 H LEU A 110 2.225 17.531 -38.223 1.00 40.63 H
ATOM 925 HA LEU A 110 1.704 17.153 -41.025 1.00 36.80 H
ATOM 926 HB2 LEU A 110 0.154 16.514 -39.186 1.00 34.33 H
ATOM 927 HB3 LEU A 110 -0.070 18.179 -38.779 1.00 34.33 H
ATOM 928 HG LEU A 110 -1.138 18.591 -40.993 1.00 42.10 H
ATOM 929 HD11 LEU A 110 -1.798 16.015 -41.941 1.00 37.24 H
ATOM 930 HD12 LEU A 110 -0.655 17.133 -42.639 1.00 37.24 H
ATOM 931 HD13 LEU A 110 -0.103 15.891 -41.546 1.00 37.24 H
ATOM 932 HD21 LEU A 110 -3.049 16.640 -40.332 1.00 36.08 H
ATOM 933 HD22 LEU A 110 -2.205 16.938 -38.830 1.00 36.08 H
ATOM 934 HD23 LEU A 110 -2.924 18.267 -39.703 1.00 36.08 H
ATOM 935 N ALA A 111 1.659 20.253 -39.867 1.00 37.27 N
ATOM 936 CA ALA A 111 1.675 21.642 -40.310 1.00 40.38 C
ATOM 937 C ALA A 111 2.900 21.936 -41.166 1.00 41.27 C
ATOM 938 O ALA A 111 2.807 22.661 -42.162 1.00 40.23 O
ATOM 939 CB ALA A 111 1.626 22.579 -39.103 1.00 31.94 C
ATOM 940 H ALA A 111 1.717 20.069 -38.873 1.00 37.27 H
ATOM 941 HA ALA A 111 0.784 21.805 -40.911 1.00 40.38 H
ATOM 942 HB1 ALA A 111 1.600 23.622 -39.417 1.00 31.94 H
ATOM 943 HB2 ALA A 111 0.729 22.397 -38.511 1.00 31.94 H
ATOM 944 HB3 ALA A 111 2.486 22.440 -38.447 1.00 31.94 H
ATOM 945 N LEU A 112 4.056 21.381 -40.794 1.00 36.20 N
ATOM 946 CA LEU A 112 5.262 21.585 -41.590 1.00 40.94 C
ATOM 947 C LEU A 112 5.106 20.995 -42.987 1.00 38.12 C
ATOM 948 O LEU A 112 5.461 21.634 -43.984 1.00 34.28 O
ATOM 949 CB LEU A 112 6.466 20.971 -40.877 1.00 40.03 C
ATOM 950 CG LEU A 112 7.811 21.040 -41.601 1.00 41.57 C
ATOM 951 CD1 LEU A 112 8.209 22.488 -41.865 1.00 41.38 C
ATOM 952 CD2 LEU A 112 8.885 20.323 -40.796 1.00 45.01 C
ATOM 953 H LEU A 112 4.124 20.808 -39.962 1.00 36.20 H
ATOM 954 HA LEU A 112 5.426 22.659 -41.695 1.00 40.94 H
ATOM 955 HB2 LEU A 112 6.565 21.444 -39.898 1.00 40.03 H
```

ATOM 956 HB3 LEU A 112 6.242 19.923 -40.667 1.00 40.03 H
ATOM 957 HG LEU A 112 7.742 20.523 -42.559 1.00 41.57 H
ATOM 958 HD11 LEU A 112 9.288 22.643 -41.838 1.00 41.38 H
ATOM 959 HD12 LEU A 112 7.877 22.799 -42.856 1.00 41.38 H
ATOM 960 HD13 LEU A 112 7.767 23.175 -41.144 1.00 41.38 H
ATOM 961 HD21 LEU A 112 9.340 19.527 -41.386 1.00 45.01 H
ATOM 962 HD22 LEU A 112 9.690 20.981 -40.468 1.00 45.01 H
ATOM 963 HD23 LEU A 112 8.484 19.857 -39.895 1.00 45.01 H
ATOM 964 N ALA A 113 4.571 19.776 -43.080 1.00 31.62 N
ATOM 965 CA ALA A 113 4.363 19.162 -44.388 1.00 32.47 C
ATOM 966 C ALA A 113 3.343 19.940 -45.208 1.00 36.70 C
ATOM 967 O ALA A 113 3.522 20.131 -46.417 1.00 37.48 O
ATOM 968 CB ALA A 113 3.924 17.710 -44.205 1.00 26.60 C
ATOM 969 H ALA A 113 4.286 19.258 -42.260 1.00 31.62 H
ATOM 970 HA ALA A 113 5.313 19.191 -44.904 1.00 32.47 H
ATOM 971 HB1 ALA A 113 3.834 17.203 -45.166 1.00 26.60 H
ATOM 972 HB2 ALA A 113 4.669 17.189 -43.612 1.00 26.60 H
ATOM 973 HB3 ALA A 113 2.966 17.635 -43.687 1.00 26.60 H
ATOM 974 N ASP A 114 2.268 20.401 -44.567 1.00 35.20 N
ATOM 975 CA ASP A 114 1.241 21.147 -45.286 1.00 38.59 C
ATOM 976 C ASP A 114 1.748 22.506 -45.751 1.00 41.43 C
ATOM 977 O ASP A 114 1.364 22.971 -46.830 1.00 38.49 O
ATOM 978 CB ASP A 114 -0.035 21.264 -44.426 1.00 38.51 C
ATOM 979 CG ASP A 114 -0.819 19.961 -44.314 1.00 51.63 C
ATOM 980 OD1 ASP A 114 -0.875 19.251 -45.339 1.00 54.84 O
ATOM 981 OD2 ASP A 114 -1.494 19.763 -43.286 1.00 56.10 O1-
ATOM 982 H ASP A 114 2.117 20.215 -43.583 1.00 35.20 H
ATOM 983 HA ASP A 114 0.984 20.604 -46.199 1.00 38.59 H
ATOM 984 HB2 ASP A 114 0.277 21.531 -43.417 1.00 38.51 H
ATOM 985 HB3 ASP A 114 -0.697 22.053 -44.766 1.00 38.51 H
ATOM 986 N ALA A 115 2.608 23.156 -44.962 1.00 39.13 N
ATOM 987 CA ALA A 115 3.194 24.418 -45.402 1.00 40.65 C
ATOM 988 C ALA A 115 4.135 24.206 -46.583 1.00 42.56 C
ATOM 989 O ALA A 115 4.138 25.000 -47.531 1.00 42.31 O
ATOM 990 CB ALA A 115 3.928 25.090 -44.244 1.00 40.00 C
ATOM 991 H ALA A 115 2.888 22.784 -44.062 1.00 39.13 H
ATOM 992 HA ALA A 115 2.393 25.091 -45.715 1.00 40.65 H
ATOM 993 HB1 ALA A 115 4.374 26.034 -44.556 1.00 40.00 H
ATOM 994 HB2 ALA A 115 3.254 25.309 -43.418 1.00 40.00 H
ATOM 995 HB3 ALA A 115 4.727 24.458 -43.854 1.00 40.00 H

ATOM 996 N LEU A 116 4.943 23.144 -46.544 1.00 39.38 N
ATOM 997 CA LEU A 116 5.849 22.868 -47.655 1.00 46.50 C
ATOM 998 C LEU A 116 5.086 22.490 -48.917 1.00 41.35 C
ATOM 999 O LEU A 116 5.501 22.856 -50.024 1.00 40.37 O
ATOM 1000 CB LEU A 116 6.832 21.760 -47.271 1.00 41.31 C
ATOM 1001 CG LEU A 116 7.938 22.164 -46.294 1.00 45.04 C
ATOM 1002 CD1 LEU A 116 8.667 20.941 -45.757 1.00 40.90 C
ATOM 1003 CD2 LEU A 116 8.916 23.119 -46.966 1.00 42.26 C
ATOM 1004 H LEU A 116 4.947 22.510 -45.755 1.00 39.38 H
ATOM 1005 HA LEU A 116 6.414 23.775 -47.872 1.00 46.50 H
ATOM 1006 HB2 LEU A 116 6.256 20.947 -46.830 1.00 41.31 H
ATOM 1007 HB3 LEU A 116 7.298 21.348 -48.167 1.00 41.31 H
ATOM 1008 HG LEU A 116 7.490 22.677 -45.443 1.00 45.04 H
ATOM 1009 HD11 LEU A 116 9.586 21.213 -45.236 1.00 40.90 H
ATOM 1010 HD12 LEU A 116 8.042 20.405 -45.042 1.00 40.90 H
ATOM 1011 HD13 LEU A 116 8.930 20.246 -46.554 1.00 40.90 H
ATOM 1012 HD21 LEU A 116 9.950 22.780 -46.891 1.00 42.26 H
ATOM 1013 HD22 LEU A 116 8.711 23.255 -48.029 1.00 42.26 H
ATOM 1014 HD23 LEU A 116 8.869 24.105 -46.502 1.00 42.26 H
ATOM 1015 N ALA A 117 3.970 21.771 -48.771 1.00 37.55 N
ATOM 1016 CA ALA A 117 3.159 21.406 -49.928 1.00 38.91 C
ATOM 1017 C ALA A 117 2.637 22.644 -50.645 1.00 40.28 C
ATOM 1018 O ALA A 117 2.754 22.764 -51.871 1.00 40.11 O
ATOM 1019 CB ALA A 117 2.010 20.487 -49.495 1.00 33.82 C
ATOM 1020 H ALA A 117 3.664 21.455 -47.859 1.00 37.55 H
ATOM 1021 HA ALA A 117 3.783 20.842 -50.625 1.00 38.91 H
ATOM 1022 HB1 ALA A 117 1.438 20.166 -50.365 1.00 33.82 H
ATOM 1023 HB2 ALA A 117 2.388 19.587 -49.008 1.00 33.82 H
ATOM 1024 HB3 ALA A 117 1.323 20.977 -48.805 1.00 33.82 H
ATOM 1025 N THR A 118 2.059 23.585 -49.894 1.00 37.59 N
ATOM 1026 CA THR A 118 1.531 24.790 -50.518 1.00 39.93 C
ATOM 1027 C THR A 118 2.634 25.737 -50.971 1.00 40.13 C
ATOM 1028 O THR A 118 2.367 26.629 -51.783 1.00 44.50 O
ATOM 1029 CB THR A 118 0.515 25.447 -49.546 1.00 49.41 C
ATOM 1030 CG2 THR A 118 -0.755 24.580 -49.450 1.00 47.20 C
ATOM 1031 OG1 THR A 118 1.019 25.570 -48.223 1.00 49.57 O
ATOM 1032 H THR A 118 1.968 23.480 -48.893 1.00 37.59 H
ATOM 1033 HA THR A 118 0.958 24.508 -51.403 1.00 39.93 H
ATOM 1034 HB THR A 118 0.248 26.445 -49.897 1.00 49.41 H
ATOM 1035 HG1 THR A 118 1.032 24.705 -47.789 1.00 49.57 H

ATOM 1036 HG21 THR A 118 -1.243 24.503 -50.420 1.00 47.20 H
ATOM 1037 HG22 THR A 118 -0.534 23.565 -49.119 1.00 47.20 H
ATOM 1038 HG23 THR A 118 -1.493 24.989 -48.766 1.00 47.20 H
ATOM 1039 N SER A 119 3.866 25.553 -50.486 1.00 38.77 N
ATOM 1040 CA SER A 119 4.972 26.390 -50.935 1.00 42.05 C
ATOM 1041 C SER A 119 5.354 26.124 -52.387 1.00 38.66 C
ATOM 1042 O SER A 119 6.067 26.938 -52.981 1.00 41.99 O
ATOM 1043 CB SER A 119 6.195 26.191 -50.037 1.00 43.09 C
ATOM 1044 OG SER A 119 6.815 24.941 -50.284 1.00 42.83 O
ATOM 1045 H SER A 119 4.058 24.836 -49.800 1.00 38.77 H
ATOM 1046 HA SER A 119 4.668 27.436 -50.849 1.00 42.05 H
ATOM 1047 HB2 SER A 119 6.930 26.974 -50.226 1.00 43.09 H
ATOM 1048 HB3 SER A 119 5.928 26.267 -48.983 1.00 43.09 H
ATOM 1049 HG SER A 119 6.187 24.224 -50.122 1.00 42.83 H
ATOM 1050 N THR A 120 4.902 25.013 -52.971 1.00 41.13 N
ATOM 1051 CA THR A 120 5.166 24.750 -54.381 1.00 44.69 C
ATOM 1052 C THR A 120 4.185 25.459 -55.307 1.00 48.12 C
ATOM 1053 O THR A 120 4.455 25.553 -56.510 1.00 48.32 O
ATOM 1054 CB THR A 120 5.054 23.218 -54.636 1.00 43.40 C
ATOM 1055 CG2 THR A 120 5.954 22.379 -53.713 1.00 41.11 C
ATOM 1056 OG1 THR A 120 3.723 22.735 -54.535 1.00 41.21 O
ATOM 1057 H THR A 120 4.328 24.347 -52.471 1.00 41.13 H
ATOM 1058 HA THR A 120 6.174 25.078 -54.634 1.00 44.69 H
ATOM 1059 HB THR A 120 5.362 23.024 -55.665 1.00 43.40 H
ATOM 1060 HG1 THR A 120 3.474 22.606 -53.604 1.00 41.21 H
ATOM 1061 HG21 THR A 120 5.911 21.324 -53.975 1.00 41.11 H
ATOM 1062 HG22 THR A 120 6.996 22.689 -53.792 1.00 41.11 H
ATOM 1063 HG23 THR A 120 5.665 22.462 -52.666 1.00 41.11 H
ATOM 1064 N LEU A 121 3.068 25.957 -54.777 1.00 45.47 N
ATOM 1065 CA LEU A 121 2.035 26.541 -55.629 1.00 42.50 C
ATOM 1066 C LEU A 121 2.479 27.785 -56.391 1.00 42.33 C
ATOM 1067 O LEU A 121 2.042 27.945 -57.545 1.00 48.67 O
ATOM 1068 CB LEU A 121 0.737 26.805 -54.840 1.00 41.62 C
ATOM 1069 CG LEU A 121 0.018 25.544 -54.325 1.00 47.59 C
ATOM 1070 CD1 LEU A 121 -1.185 25.927 -53.442 1.00 46.36 C
ATOM 1071 CD2 LEU A 121 -0.390 24.592 -55.466 1.00 48.72 C
ATOM 1072 H LEU A 121 2.880 25.885 -53.786 1.00 45.47 H
ATOM 1073 HA LEU A 121 1.813 25.800 -56.394 1.00 42.50 H
ATOM 1074 HB2 LEU A 121 0.978 27.439 -53.991 1.00 41.62 H
ATOM 1075 HB3 LEU A 121 0.041 27.375 -55.460 1.00 41.62 H

ATOM 1076 HG LEU A 121 0.716 24.992 -53.694 1.00 47.59 H
ATOM 1077 HD11 LEU A 121 -2.036 25.262 -53.586 1.00 46.36 H
ATOM 1078 HD12 LEU A 121 -0.921 25.880 -52.386 1.00 46.36 H
ATOM 1079 HD13 LEU A 121 -1.536 26.939 -53.644 1.00 46.36 H
ATOM 1080 HD21 LEU A 121 0.142 23.647 -55.365 1.00 48.72 H
ATOM 1081 HD22 LEU A 121 -1.455 24.365 -55.480 1.00 48.72 H
ATOM 1082 HD23 LEU A 121 -0.153 24.969 -56.458 1.00 48.72 H
ATOM 1083 N PRO A 122 3.290 28.700 -55.839 1.00 44.44 N
ATOM 1084 CA PRO A 122 3.761 29.817 -56.676 1.00 48.60 C
ATOM 1085 C PRO A 122 4.522 29.355 -57.906 1.00 46.99 C
ATOM 1086 O PRO A 122 4.412 29.980 -58.968 1.00 46.49 O
ATOM 1087 CB PRO A 122 4.647 30.625 -55.719 1.00 44.16 C
ATOM 1088 CG PRO A 122 4.096 30.321 -54.361 1.00 41.67 C
ATOM 1089 CD PRO A 122 3.683 28.879 -54.426 1.00 40.84 C
ATOM 1090 HA PRO A 122 2.899 30.425 -56.951 1.00 48.60 H
ATOM 1091 HB2 PRO A 122 5.685 30.289 -55.767 1.00 44.16 H
ATOM 1092 HB3 PRO A 122 4.637 31.693 -55.939 1.00 44.16 H
ATOM 1093 HG2 PRO A 122 4.800 30.527 -53.554 1.00 41.67 H
ATOM 1094 HG3 PRO A 122 3.212 30.938 -54.192 1.00 41.67 H
ATOM 1095 HD2 PRO A 122 4.548 28.251 -54.258 1.00 40.84 H
ATOM 1096 HD3 PRO A 122 2.914 28.621 -53.704 1.00 40.84 H
ATOM 1097 N PHE A 123 5.288 28.267 -57.795 1.00 43.45 N
ATOM 1098 CA PHE A 123 5.943 27.704 -58.971 1.00 46.19 C
ATOM 1099 C PHE A 123 4.920 27.166 -59.963 1.00 48.77 C
ATOM 1100 O PHE A 123 5.046 27.380 -61.174 1.00 47.05 O
ATOM 1101 CB PHE A 123 6.928 26.587 -58.577 1.00 45.53 C
ATOM 1102 CG PHE A 123 8.063 27.020 -57.672 1.00 52.69 C
ATOM 1103 CD1 PHE A 123 9.258 27.526 -58.225 1.00 52.11 C
ATOM 1104 CD2 PHE A 123 7.880 27.073 -56.275 1.00 54.02 C
ATOM 1105 CE1 PHE A 123 10.259 28.003 -57.393 1.00 55.66 C
ATOM 1106 CE2 PHE A 123 8.895 27.548 -55.458 1.00 52.63 C
ATOM 1107 CZ PHE A 123 10.083 28.005 -56.015 1.00 54.62 C
ATOM 1108 H PHE A 123 5.375 27.767 -56.922 1.00 43.45 H
ATOM 1109 HA PHE A 123 6.515 28.491 -59.467 1.00 46.19 H
ATOM 1110 HB2 PHE A 123 6.404 25.758 -58.103 1.00 45.53 H
ATOM 1111 HB3 PHE A 123 7.367 26.172 -59.484 1.00 45.53 H
ATOM 1112 HD1 PHE A 123 9.403 27.543 -59.294 1.00 52.11 H
ATOM 1113 HD2 PHE A 123 6.954 26.749 -55.829 1.00 54.02 H
ATOM 1114 HE1 PHE A 123 11.173 28.389 -57.819 1.00 55.66 H
ATOM 1115 HE2 PHE A 123 8.753 27.573 -54.386 1.00 52.63 H

ATOM 1116 HZ PHE A 123 10.868 28.381 -55.374 1.00 54.62 H
ATOM 1117 N GLN A 124 3.893 26.472 -59.464 1.00 47.06 N
ATOM 1118 CA GLN A 124 2.889 25.892 -60.348 1.00 45.94 C
ATOM 1119 C GLN A 124 2.046 26.969 -61.019 1.00 47.60 C
ATOM 1120 O GLN A 124 1.646 26.818 -62.180 1.00 46.26 O
ATOM 1121 CB GLN A 124 1.974 24.946 -59.551 1.00 38.61 C
ATOM 1122 CG GLN A 124 2.715 23.749 -58.935 1.00 36.71 C
ATOM 1123 CD GLN A 124 1.764 22.831 -58.179 1.00 39.59 C
ATOM 1124 NE2 GLN A 124 2.112 22.436 -56.965 1.00 37.08 N
ATOM 1125 OE1 GLN A 124 0.728 22.439 -58.703 1.00 44.76 O
ATOM 1126 H GLN A 124 3.803 26.308 -58.470 1.00 47.06 H
ATOM 1127 HA GLN A 124 3.395 25.326 -61.132 1.00 45.94 H
ATOM 1128 HB2 GLN A 124 1.453 25.498 -58.767 1.00 38.61 H
ATOM 1129 HB3 GLN A 124 1.187 24.574 -60.212 1.00 38.61 H
ATOM 1130 HG2 GLN A 124 3.187 23.162 -59.724 1.00 36.71 H
ATOM 1131 HG3 GLN A 124 3.515 24.073 -58.275 1.00 36.71 H
ATOM 1132 HE21 GLN A 124 2.928 22.748 -56.451 1.00 37.08 H
ATOM 1133 HE22 GLN A 124 1.561 21.698 -56.519 1.00 37.08 H
ATOM 1134 N SER A 125 1.761 28.060 -60.309 1.00 44.75 N
ATOM 1135 CA SER A 125 1.011 29.156 -60.911 1.00 48.84 C
ATOM 1136 C SER A 125 1.775 29.750 -62.088 1.00 53.07 C
ATOM 1137 O SER A 125 1.260 29.815 -63.210 1.00 56.61 O
ATOM 1138 CB SER A 125 0.627 30.167 -59.808 1.00 58.34 C
ATOM 1139 OG SER A 125 1.699 30.948 -59.304 1.00 77.79 O
ATOM 1140 H SER A 125 2.051 28.147 -59.343 1.00 44.75 H
ATOM 1141 HA SER A 125 0.058 28.769 -61.279 1.00 48.84 H
ATOM 1142 HB2 SER A 125 -0.128 30.849 -60.200 1.00 58.34 H
ATOM 1143 HB3 SER A 125 0.161 29.645 -58.976 1.00 58.34 H
ATOM 1144 HG SER A 125 2.421 30.375 -59.014 1.00 77.79 H
ATOM 1145 N VAL A 126 3.020 30.176 -61.847 1.00 51.16 N
ATOM 1146 CA VAL A 126 3.857 30.732 -62.912 1.00 51.42 C
ATOM 1147 C VAL A 126 3.974 29.747 -64.066 1.00 53.81 C
ATOM 1148 O VAL A 126 3.909 30.129 -65.241 1.00 51.45 O
ATOM 1149 CB VAL A 126 5.243 31.114 -62.359 1.00 56.39 C
ATOM 1150 CG1 VAL A 126 6.167 31.547 -63.490 1.00 56.26 C
ATOM 1151 CG2 VAL A 126 5.114 32.215 -61.317 1.00 56.66 C
ATOM 1152 H VAL A 126 3.411 30.134 -60.915 1.00 51.16 H
ATOM 1153 HA VAL A 126 3.478 31.623 -63.366 1.00 51.42 H
ATOM 1154 HB VAL A 126 5.679 30.242 -61.869 1.00 56.39 H
ATOM 1155 HG11 VAL A 126 7.095 31.957 -63.092 1.00 56.26 H

ATOM	1156	HG12	VAL	A	126	6.459	30.718	-64.135	1.00	56.26	H
ATOM	1157	HG13	VAL	A	126	5.713	32.321	-64.111	1.00	56.26	H
ATOM	1158	HG21	VAL	A	126	6.079	32.435	-60.860	1.00	56.66	H
ATOM	1159	HG22	VAL	A	126	4.747	33.138	-61.768	1.00	56.66	H
ATOM	1160	HG23	VAL	A	126	4.428	31.960	-60.512	1.00	56.66	H
ATOM	1161	N	ASN	A	127	4.147	28.462	-63.747	1.00	49.91	N
ATOM	1162	CA	ASN	A	127	4.192	27.440	-64.785	1.00	53.68	C
ATOM	1163	C	ASN	A	127	2.919	27.439	-65.622	1.00	60.12	C
ATOM	1164	O	ASN	A	127	2.970	27.165	-66.827	1.00	66.23	O
ATOM	1165	CB	ASN	A	127	4.452	26.061	-64.133	1.00	52.67	C
ATOM	1166	CG	ASN	A	127	4.574	24.910	-65.136	1.00	59.71	C
ATOM	1167	ND2	ASN	A	127	3.559	24.056	-65.232	1.00	65.68	N
ATOM	1168	OD1	ASN	A	127	5.588	24.773	-65.806	1.00	63.30	O
ATOM	1169	H	ASN	A	127	4.225	28.163	-62.783	1.00	49.91	H
ATOM	1170	HA	ASN	A	127	5.037	27.673	-65.431	1.00	53.68	H
ATOM	1171	HB2	ASN	A	127	5.382	26.098	-63.566	1.00	52.67	H
ATOM	1172	HB3	ASN	A	127	3.668	25.825	-63.418	1.00	52.67	H
ATOM	1173	HD21	ASN	A	127	2.718	24.169	-64.688	1.00	65.68	H
ATOM	1174	HD22	ASN	A	127	3.607	23.303	-65.902	1.00	65.68	H
ATOM	1175	N	TYR	A	128	1.775	27.763	-65.013	1.00	61.86	N
ATOM	1176	CA	TYR	A	128	0.530	27.833	-65.769	1.00	64.74	C
ATOM	1177	C	TYR	A	128	0.438	29.116	-66.589	1.00	68.82	C
ATOM	1178	O	TYR	A	128	0.053	29.078	-67.762	1.00	72.91	O
ATOM	1179	CB	TYR	A	128	-0.686	27.658	-64.812	1.00	52.59	C
ATOM	1180	CG	TYR	A	128	-2.024	28.095	-65.394	1.00	62.72	C
ATOM	1181	CD1	TYR	A	128	-2.648	27.308	-66.384	1.00	72.45	C
ATOM	1182	CD2	TYR	A	128	-2.587	29.340	-65.033	1.00	61.54	C
ATOM	1183	CE1	TYR	A	128	-3.796	27.782	-67.046	1.00	75.42	C
ATOM	1184	CE2	TYR	A	128	-3.735	29.814	-65.697	1.00	68.38	C
ATOM	1185	CZ	TYR	A	128	-4.326	29.044	-66.717	1.00	74.70	C
ATOM	1186	OH	TYR	A	128	-5.414	29.523	-67.384	1.00	82.90	O
ATOM	1187	H	TYR	A	128	1.747	28.001	-64.030	1.00	61.86	H
ATOM	1188	HA	TYR	A	128	0.478	26.988	-66.460	1.00	64.74	H
ATOM	1189	HB2	TYR	A	128	-0.756	26.619	-64.486	1.00	52.59	H
ATOM	1190	HB3	TYR	A	128	-0.535	28.231	-63.900	1.00	52.59	H
ATOM	1191	HD1	TYR	A	128	-2.220	26.360	-66.674	1.00	72.45	H
ATOM	1192	HD2	TYR	A	128	-2.117	29.959	-64.281	1.00	61.54	H
ATOM	1193	HE1	TYR	A	128	-4.246	27.179	-67.820	1.00	75.42	H
ATOM	1194	HE2	TYR	A	128	-4.150	30.777	-65.435	1.00	68.38	H
ATOM	1195	HH	TYR	A	128	-5.731	28.929	-68.070	1.00	82.90	H

ATOM 1196 N LEU A 129 0.760 30.262 -65.983 1.00 67.11 N
ATOM 1197 CA LEU A 129 0.747 31.545 -66.683 1.00 76.46 C
ATOM 1198 C LEU A 129 1.506 31.471 -67.997 1.00 75.70 C
ATOM 1199 O LEU A 129 0.930 31.610 -69.081 1.00 81.95 O
ATOM 1200 CB LEU A 129 1.390 32.646 -65.839 1.00 84.84 C
ATOM 1201 CG LEU A 129 0.688 33.430 -64.739 1.00 93.76 C
ATOM 1202 CD1 LEU A 129 0.942 32.770 -63.432 1.00 93.40 C
ATOM 1203 CD2 LEU A 129 1.258 34.828 -64.705 1.00104.03 C
ATOM 1204 H LEU A 129 1.053 30.262 -65.014 1.00 67.11 H
ATOM 1205 HA LEU A 129 -0.289 31.815 -66.897 1.00 76.46 H
ATOM 1206 HB2 LEU A 129 2.358 32.307 -65.467 1.00 84.84 H
ATOM 1207 HB3 LEU A 129 1.662 33.420 -66.562 1.00 84.84 H
ATOM 1208 HG LEU A 129 -0.384 33.482 -64.934 1.00 93.76 H
ATOM 1209 HD11 LEU A 129 0.525 33.407 -62.656 1.00 93.40 H
ATOM 1210 HD12 LEU A 129 0.370 31.861 -63.356 1.00 93.40 H
ATOM 1211 HD13 LEU A 129 1.966 32.731 -63.132 1.00 93.40 H
ATOM 1212 HD21 LEU A 129 0.985 35.353 -63.789 1.00104.03 H
ATOM 1213 HD22 LEU A 129 2.345 34.831 -64.763 1.00104.03 H
ATOM 1214 HD23 LEU A 129 0.876 35.425 -65.534 1.00104.03 H
ATOM 1215 N MET A 130 2.818 31.266 -67.891 1.00 69.65 N
ATOM 1216 CA MET A 130 3.706 31.271 -69.041 1.00 76.07 C
ATOM 1217 C MET A 130 3.542 30.042 -69.919 1.00 67.04 C
ATOM 1218 O MET A 130 4.119 30.006 -71.011 1.00 66.30 O
ATOM 1219 CB MET A 130 5.152 31.380 -68.559 1.00 78.91 C
ATOM 1220 CG MET A 130 5.346 32.404 -67.450 1.00 79.21 C
ATOM 1221 SD MET A 130 4.766 34.046 -67.921 1.00 91.71 S
ATOM 1222 CE MET A 130 4.922 34.917 -66.365 1.00 94.83 C
ATOM 1223 H MET A 130 3.240 31.121 -66.984 1.00 69.65 H
ATOM 1224 HA MET A 130 3.475 32.143 -69.655 1.00 76.07 H
ATOM 1225 HB2 MET A 130 5.490 30.413 -68.182 1.00 78.91 H
ATOM 1226 HB3 MET A 130 5.808 31.619 -69.397 1.00 78.91 H
ATOM 1227 HG2 MET A 130 4.850 32.104 -66.529 1.00 79.21 H
ATOM 1228 HG3 MET A 130 6.406 32.476 -67.205 1.00 79.21 H
ATOM 1229 HE1 MET A 130 4.488 35.914 -66.442 1.00 94.83 H
ATOM 1230 HE2 MET A 130 4.412 34.371 -65.573 1.00 94.83 H
ATOM 1231 HE3 MET A 130 5.973 35.014 -66.091 1.00 94.83 H
ATOM 1232 N GLY A 131 2.771 29.050 -69.478 1.00 57.77 N
ATOM 1233 CA GLY A 131 2.672 27.801 -70.212 1.00 66.69 C
ATOM 1234 C GLY A 131 4.003 27.109 -70.389 1.00 70.85 C
ATOM 1235 O GLY A 131 4.221 26.443 -71.406 1.00 68.92 O

ATOM 1236 H GLY A 131 2.282 29.128 -68.597 1.00 57.77 H
ATOM 1237 HA2 GLY A 131 2.004 27.134 -69.667 1.00 66.69 H
ATOM 1238 HA3 GLY A 131 2.213 27.981 -71.186 1.00 66.69 H
ATOM 1239 N THR A 132 4.907 27.258 -69.422 1.00 71.11 N
ATOM 1240 CA THR A 132 6.283 26.796 -69.540 1.00 68.12 C
ATOM 1241 C THR A 132 6.927 26.854 -68.163 1.00 65.02 C
ATOM 1242 O THR A 132 6.625 27.755 -67.376 1.00 64.28 O
ATOM 1243 CB THR A 132 7.073 27.661 -70.537 1.00 74.02 C
ATOM 1244 CG2 THR A 132 8.532 27.247 -70.598 1.00 65.17 C
ATOM 1245 OG1 THR A 132 6.500 27.534 -71.844 1.00 87.22 O
ATOM 1246 H THR A 132 4.678 27.778 -68.586 1.00 71.11 H
ATOM 1247 HA THR A 132 6.272 25.755 -69.873 1.00 68.12 H
ATOM 1248 HB THR A 132 7.009 28.715 -70.260 1.00 74.02 H
ATOM 1249 HG1 THR A 132 5.545 27.631 -71.770 1.00 87.22 H
ATOM 1250 HG21 THR A 132 9.019 27.589 -71.512 1.00 65.17 H
ATOM 1251 HG22 THR A 132 9.107 27.635 -69.757 1.00 65.17 H
ATOM 1252 HG23 THR A 132 8.568 26.174 -70.565 1.00 65.17 H
ATOM 1253 N TRP A 133 7.801 25.886 -67.875 1.00 62.25 N
ATOM 1254 CA TRP A 133 8.572 25.890 -66.639 1.00 59.33 C
ATOM 1255 C TRP A 133 9.813 26.749 -66.835 1.00 60.73 C
ATOM 1256 O TRP A 133 10.702 26.361 -67.612 1.00 60.73 O
ATOM 1257 CB TRP A 133 8.972 24.448 -66.283 1.00 55.70 C
ATOM 1258 CG TRP A 133 9.801 24.342 -65.039 1.00 51.04 C
ATOM 1259 CD1 TRP A 133 11.152 24.318 -64.987 1.00 46.45 C
ATOM 1260 CD2 TRP A 133 9.346 24.346 -63.656 1.00 48.16 C
ATOM 1261 CE2 TRP A 133 10.489 24.289 -62.802 1.00 45.74 C
ATOM 1262 CE3 TRP A 133 8.076 24.385 -63.036 1.00 48.43 C
ATOM 1263 NE1 TRP A 133 11.564 24.278 -63.671 1.00 50.15 N
ATOM 1264 CZ2 TRP A 133 10.368 24.271 -61.401 1.00 43.95 C
ATOM 1265 CZ3 TRP A 133 7.945 24.363 -61.634 1.00 46.50 C
ATOM 1266 CH2 TRP A 133 9.090 24.306 -60.817 1.00 44.59 C
ATOM 1267 H TRP A 133 7.981 25.137 -68.531 1.00 62.25 H
ATOM 1268 HA TRP A 133 7.952 26.260 -65.822 1.00 59.33 H
ATOM 1269 HB2 TRP A 133 8.076 23.843 -66.141 1.00 55.70 H
ATOM 1270 HB3 TRP A 133 9.518 23.984 -67.103 1.00 55.70 H
ATOM 1271 HD1 TRP A 133 11.791 24.326 -65.859 1.00 46.45 H
ATOM 1272 HE1 TRP A 133 12.553 24.186 -63.423 1.00 50.15 H
ATOM 1273 HE3 TRP A 133 7.192 24.415 -63.655 1.00 48.43 H
ATOM 1274 HZ2 TRP A 133 11.242 24.239 -60.771 1.00 43.95 H
ATOM 1275 HZ3 TRP A 133 6.961 24.382 -61.188 1.00 46.50 H

ATOM	1276	HH2	TRP	A	133	8.991	24.286	-59.743	1.00	44.59	H
ATOM	1277	N	PRO	A	134	9.931	27.900	-66.175	1.00	59.31	N
ATOM	1278	CA	PRO	A	134	11.081	28.789	-66.385	1.00	61.82	C
ATOM	1279	C	PRO	A	134	12.171	28.717	-65.321	1.00	52.54	C
ATOM	1280	O	PRO	A	134	13.133	29.486	-65.414	1.00	59.88	O
ATOM	1281	CB	PRO	A	134	10.413	30.166	-66.351	1.00	65.76	C
ATOM	1282	CG	PRO	A	134	9.362	29.996	-65.274	1.00	60.39	C
ATOM	1283	CD	PRO	A	134	8.895	28.548	-65.348	1.00	59.94	C
ATOM	1284	HA	PRO	A	134	11.556	28.658	-67.359	1.00	61.82	H
ATOM	1285	HB2	PRO	A	134	11.088	31.000	-66.153	1.00	65.76	H
ATOM	1286	HB3	PRO	A	134	9.931	30.358	-67.311	1.00	65.76	H
ATOM	1287	HG2	PRO	A	134	9.816	30.171	-64.297	1.00	60.39	H
ATOM	1288	HG3	PRO	A	134	8.545	30.711	-65.376	1.00	60.39	H
ATOM	1289	HD2	PRO	A	134	8.819	28.075	-64.377	1.00	59.94	H
ATOM	1290	HD3	PRO	A	134	7.906	28.562	-65.758	1.00	59.94	H
ATOM	1291	N	PHE	A	135	12.049	27.839	-64.329	1.00	55.12	N
ATOM	1292	CA	PHE	A	135	12.913	27.877	-63.157	1.00	58.88	C
ATOM	1293	C	PHE	A	135	14.155	27.002	-63.283	1.00	60.51	C
ATOM	1294	O	PHE	A	135	14.977	26.984	-62.359	1.00	60.28	O
ATOM	1295	CB	PHE	A	135	12.116	27.471	-61.912	1.00	55.16	C
ATOM	1296	CG	PHE	A	135	10.916	28.339	-61.654	1.00	54.43	C
ATOM	1297	CD1	PHE	A	135	11.062	29.603	-61.103	1.00	57.42	C
ATOM	1298	CD2	PHE	A	135	9.641	27.892	-61.964	1.00	48.71	C
ATOM	1299	CE1	PHE	A	135	9.960	30.405	-60.868	1.00	56.04	C
ATOM	1300	CE2	PHE	A	135	8.535	28.687	-61.729	1.00	53.70	C
ATOM	1301	CZ	PHE	A	135	8.695	29.946	-61.181	1.00	61.05	C
ATOM	1302	H	PHE	A	135	11.259	27.210	-64.295	1.00	55.12	H
ATOM	1303	HA	PHE	A	135	13.268	28.896	-62.987	1.00	58.88	H
ATOM	1304	HB2	PHE	A	135	11.796	26.438	-61.987	1.00	55.16	H
ATOM	1305	HB3	PHE	A	135	12.750	27.503	-61.041	1.00	55.16	H
ATOM	1306	HD1	PHE	A	135	12.047	29.970	-60.854	1.00	57.42	H
ATOM	1307	HD2	PHE	A	135	9.500	26.914	-62.395	1.00	48.71	H
ATOM	1308	HE1	PHE	A	135	10.088	31.387	-60.438	1.00	56.04	H
ATOM	1309	HE2	PHE	A	135	7.547	28.325	-61.975	1.00	53.70	H
ATOM	1310	HZ	PHE	A	135	7.835	30.569	-60.990	1.00	61.05	H
ATOM	1311	N	GLY	A	136	14.316	26.279	-64.386	1.00	60.91	N
ATOM	1312	CA	GLY	A	136	15.516	25.497	-64.601	1.00	60.06	C
ATOM	1313	C	GLY	A	136	15.411	24.074	-64.089	1.00	59.72	C
ATOM	1314	O	GLY	A	136	14.414	23.642	-63.503	1.00	56.41	O
ATOM	1315	H	GLY	A	136	13.624	26.309	-65.121	1.00	60.91	H

ATOM 1316 HA2 GLY A 136 15.683 25.455 -65.678 1.00 60.06 H
ATOM 1317 HA3 GLY A 136 16.401 25.979 -64.180 1.00 60.06 H
ATOM 1318 N THR A 137 16.500 23.334 -64.312 1.00 60.77 N
ATOM 1319 CA THR A 137 16.530 21.908 -64.004 1.00 58.22 C
ATOM 1320 C THR A 137 16.656 21.649 -62.506 1.00 56.97 C
ATOM 1321 O THR A 137 16.021 20.727 -61.979 1.00 56.23 O
ATOM 1322 CB THR A 137 17.692 21.238 -64.760 1.00 0.00 C
ATOM 1323 CG2 THR A 137 17.316 20.782 -66.175 1.00 0.00 C
ATOM 1324 OG1 THR A 137 18.785 22.159 -64.847 1.00 0.00 O
ATOM 1325 H THR A 137 17.314 23.709 -64.778 1.00 60.77 H
ATOM 1326 HA THR A 137 15.598 21.451 -64.335 1.00 58.22 H
ATOM 1327 HB THR A 137 18.043 20.360 -64.213 1.00 0.00 H
ATOM 1328 HG1 THR A 137 19.460 21.780 -65.416 1.00 0.00 H
ATOM 1329 HG21 THR A 137 18.130 20.184 -66.586 1.00 0.00 H
ATOM 1330 HG22 THR A 137 16.477 20.094 -66.100 1.00 0.00 H
ATOM 1331 HG23 THR A 137 17.288 21.671 -66.802 1.00 0.00 H
ATOM 1332 N ILE A 138 17.472 22.442 -61.808 1.00 55.50 N
ATOM 1333 CA ILE A 138 17.691 22.214 -60.382 1.00 54.19 C
ATOM 1334 C ILE A 138 16.412 22.471 -59.595 1.00 53.99 C
ATOM 1335 O ILE A 138 16.027 21.676 -58.729 1.00 50.32 O
ATOM 1336 CB ILE A 138 18.854 23.085 -59.872 1.00 62.95 C
ATOM 1337 CG1 ILE A 138 20.164 22.678 -60.550 1.00 66.39 C
ATOM 1338 CG2 ILE A 138 18.974 22.983 -58.360 1.00 64.64 C
ATOM 1339 CD1 ILE A 138 21.350 23.522 -60.139 1.00 72.99 C
ATOM 1340 H ILE A 138 17.991 23.183 -62.257 1.00 55.50 H
ATOM 1341 HA ILE A 138 17.962 21.165 -60.238 1.00 54.19 H
ATOM 1342 HB ILE A 138 18.653 24.127 -60.126 1.00 62.95 H
ATOM 1343 HG12 ILE A 138 20.381 21.633 -60.325 1.00 66.39 H
ATOM 1344 HG13 ILE A 138 20.068 22.734 -61.635 1.00 66.39 H
ATOM 1345 HG21 ILE A 138 19.842 23.522 -57.982 1.00 64.64 H
ATOM 1346 HG22 ILE A 138 18.124 23.425 -57.840 1.00 64.64 H
ATOM 1347 HG23 ILE A 138 19.069 21.946 -58.034 1.00 64.64 H
ATOM 1348 HD11 ILE A 138 22.101 23.543 -60.930 1.00 72.99 H
ATOM 1349 HD12 ILE A 138 21.064 24.554 -59.935 1.00 72.99 H
ATOM 1350 HD13 ILE A 138 21.831 23.122 -59.246 1.00 72.99 H
ATOM 1351 N LEU A 139 15.729 23.581 -59.889 1.00 52.91 N
ATOM 1352 CA LEU A 139 14.492 23.892 -59.181 1.00 50.40 C
ATOM 1353 C LEU A 139 13.379 22.920 -59.543 1.00 50.50 C
ATOM 1354 O LEU A 139 12.495 22.660 -58.719 1.00 48.57 O
ATOM 1355 CB LEU A 139 14.062 25.329 -59.473 1.00 52.50 C

ATOM 1356 CG LEU A 139 14.508 26.380 -58.452 1.00 63.26 C
ATOM 1357 CD1 LEU A 139 16.005 26.301 -58.194 1.00 76.19 C
ATOM 1358 CD2 LEU A 139 14.122 27.775 -58.917 1.00 61.59 C
ATOM 1359 H LEU A 139 16.046 24.226 -60.598 1.00 52.91 H
ATOM 1360 HA LEU A 139 14.660 23.796 -58.107 1.00 50.40 H
ATOM 1361 HB2 LEU A 139 14.398 25.597 -60.469 1.00 52.50 H
ATOM 1362 HB3 LEU A 139 12.973 25.395 -59.521 1.00 52.50 H
ATOM 1363 HG LEU A 139 13.994 26.188 -57.509 1.00 63.26 H
ATOM 1364 HD11 LEU A 139 16.420 27.269 -57.913 1.00 76.19 H
ATOM 1365 HD12 LEU A 139 16.226 25.613 -57.378 1.00 76.19 H
ATOM 1366 HD13 LEU A 139 16.560 25.970 -59.072 1.00 76.19 H
ATOM 1367 HD21 LEU A 139 14.475 28.529 -58.213 1.00 61.59 H
ATOM 1368 HD22 LEU A 139 14.558 28.018 -59.886 1.00 61.59 H
ATOM 1369 HD23 LEU A 139 13.041 27.888 -58.963 1.00 61.59 H
ATOM 1370 N CYS A 140 13.400 22.377 -60.762 1.00 47.94 N
ATOM 1371 CA CYS A 140 12.425 21.356 -61.128 1.00 45.04 C
ATOM 1372 C CYS A 140 12.571 20.123 -60.244 1.00 43.39 C
ATOM 1373 O CYS A 140 11.575 19.587 -59.746 1.00 43.25 O
ATOM 1374 CB CYS A 140 12.587 20.995 -62.623 1.00 51.30 C
ATOM 1375 SG CYS A 140 11.594 19.612 -63.242 1.00 59.60 S
ATOM 1376 H CYS A 140 14.120 22.610 -61.432 1.00 47.94 H
ATOM 1377 HA CYS A 140 11.421 21.755 -60.979 1.00 45.04 H
ATOM 1378 HB2 CYS A 140 12.356 21.857 -63.242 1.00 51.30 H
ATOM 1379 HB3 CYS A 140 13.624 20.742 -62.834 1.00 51.30 H
ATOM 1380 N LYS A 141 13.808 19.673 -60.022 1.00 46.82 N
ATOM 1381 CA LYS A 141 14.033 18.534 -59.136 1.00 48.70 C
ATOM 1382 C LYS A 141 13.633 18.859 -57.701 1.00 47.55 C
ATOM 1383 O LYS A 141 13.055 18.016 -57.006 1.00 41.87 O
ATOM 1384 CB LYS A 141 15.500 18.103 -59.194 1.00 46.54 C
ATOM 1385 CG LYS A 141 15.930 17.512 -60.527 1.00 50.52 C
ATOM 1386 CD LYS A 141 17.414 17.184 -60.528 1.00 56.71 C
ATOM 1387 CE LYS A 141 17.794 16.337 -61.731 1.00 57.81 C
ATOM 1388 NZ LYS A 141 17.124 15.004 -61.692 1.00 55.81 N1+
ATOM 1389 H LYS A 141 14.615 20.119 -60.437 1.00 46.82 H
ATOM 1390 HA LYS A 141 13.411 17.704 -59.469 1.00 48.70 H
ATOM 1391 HB2 LYS A 141 16.142 18.950 -58.945 1.00 46.54 H
ATOM 1392 HB3 LYS A 141 15.681 17.352 -58.423 1.00 46.54 H
ATOM 1393 HG2 LYS A 141 15.337 16.614 -60.681 1.00 50.52 H
ATOM 1394 HG3 LYS A 141 15.694 18.184 -61.352 1.00 50.52 H
ATOM 1395 HD2 LYS A 141 17.988 18.111 -60.545 1.00 56.71 H

ATOM	1396	HD3	LYS	A	141	17.694	16.669	-59.608	1.00	56.71	H
ATOM	1397	HE2	LYS	A	141	17.552	16.842	-62.667	1.00	57.81	H
ATOM	1398	HE3	LYS	A	141	18.872	16.173	-61.724	1.00	57.81	H
ATOM	1399	HZ1	LYS	A	141	16.143	15.131	-61.921	1.00	55.81	H
ATOM	1400	HZ2	LYS	A	141	17.532	14.383	-62.376	1.00	55.81	H
ATOM	1401	HZ3	LYS	A	141	17.200	14.600	-60.767	1.00	55.81	H
ATOM	1402	N	ILE	A	142	13.929	20.076	-57.244	1.00	45.82	N
ATOM	1403	CA	ILE	A	142	13.671	20.433	-55.852	1.00	44.45	C
ATOM	1404	C	ILE	A	142	12.173	20.535	-55.592	1.00	42.99	C
ATOM	1405	O	ILE	A	142	11.661	19.997	-54.603	1.00	42.95	O
ATOM	1406	CB	ILE	A	142	14.397	21.742	-55.495	1.00	47.55	C
ATOM	1407	CG1	ILE	A	142	15.910	21.517	-55.468	1.00	49.83	C
ATOM	1408	CG2	ILE	A	142	13.906	22.285	-54.160	1.00	45.00	C
ATOM	1409	CD1	ILE	A	142	16.711	22.768	-55.178	1.00	49.99	C
ATOM	1410	H	ILE	A	142	14.410	20.751	-57.824	1.00	45.82	H
ATOM	1411	HA	ILE	A	142	14.062	19.641	-55.209	1.00	44.45	H
ATOM	1412	HB	ILE	A	142	14.178	22.487	-56.261	1.00	47.55	H
ATOM	1413	HG12	ILE	A	142	16.139	20.784	-54.693	1.00	49.83	H
ATOM	1414	HG13	ILE	A	142	16.268	21.063	-56.388	1.00	49.83	H
ATOM	1415	HG21	ILE	A	142	14.469	23.164	-53.849	1.00	45.00	H
ATOM	1416	HG22	ILE	A	142	12.869	22.621	-54.188	1.00	45.00	H
ATOM	1417	HG23	ILE	A	142	14.003	21.542	-53.367	1.00	45.00	H
ATOM	1418	HD11	ILE	A	142	17.731	22.664	-55.549	1.00	49.99	H
ATOM	1419	HD12	ILE	A	142	16.276	23.645	-55.657	1.00	49.99	H
ATOM	1420	HD13	ILE	A	142	16.777	22.962	-54.107	1.00	49.99	H
ATOM	1421	N	VAL	A	143	11.448	21.224	-56.475	1.00	40.26	N
ATOM	1422	CA	VAL	A	143	10.024	21.445	-56.245	1.00	39.82	C
ATOM	1423	C	VAL	A	143	9.245	20.141	-56.377	1.00	39.12	C
ATOM	1424	O	VAL	A	143	8.340	19.863	-55.581	1.00	40.68	O
ATOM	1425	CB	VAL	A	143	9.473	22.537	-57.212	1.00	44.86	C
ATOM	1426	CG1	VAL	A	143	7.938	22.672	-57.250	1.00	44.25	C
ATOM	1427	CG2	VAL	A	143	10.085	23.911	-56.871	1.00	42.80	C
ATOM	1428	H	VAL	A	143	11.871	21.659	-57.287	1.00	40.26	H
ATOM	1429	HA	VAL	A	143	9.889	21.804	-55.222	1.00	39.82	H
ATOM	1430	HB	VAL	A	143	9.793	22.281	-58.224	1.00	44.86	H
ATOM	1431	HG11	VAL	A	143	7.626	23.515	-57.866	1.00	44.25	H
ATOM	1432	HG12	VAL	A	143	7.453	21.785	-57.661	1.00	44.25	H
ATOM	1433	HG13	VAL	A	143	7.547	22.833	-56.247	1.00	44.25	H
ATOM	1434	HG21	VAL	A	143	9.788	24.672	-57.592	1.00	42.80	H
ATOM	1435	HG22	VAL	A	143	9.765	24.255	-55.887	1.00	42.80	H

ATOM 1436 HG23 VAL A 143 11.174 23.889 -56.865 1.00 42.80 H
ATOM 1437 N ILE A 144 9.581 19.318 -57.374 1.00 38.20 N
ATOM 1438 CA ILE A 144 8.886 18.043 -57.536 1.00 37.51 C
ATOM 1439 C ILE A 144 9.134 17.142 -56.333 1.00 36.92 C
ATOM 1440 O ILE A 144 8.205 16.517 -55.806 1.00 38.64 O
ATOM 1441 CB ILE A 144 9.236 17.303 -58.876 1.00 45.46 C
ATOM 1442 CG1 ILE A 144 8.491 17.991 -60.050 1.00 55.47 C
ATOM 1443 CG2 ILE A 144 8.937 15.782 -58.886 1.00 41.03 C
ATOM 1444 CD1 ILE A 144 8.647 17.323 -61.429 1.00 71.81 C
ATOM 1445 H ILE A 144 10.318 19.545 -58.029 1.00 38.20 H
ATOM 1446 HA ILE A 144 7.812 18.239 -57.567 1.00 37.51 H
ATOM 1447 HB ILE A 144 10.308 17.410 -59.052 1.00 45.46 H
ATOM 1448 HG12 ILE A 144 7.424 18.032 -59.827 1.00 55.47 H
ATOM 1449 HG13 ILE A 144 8.815 19.031 -60.125 1.00 55.47 H
ATOM 1450 HG21 ILE A 144 9.227 15.317 -59.824 1.00 41.03 H
ATOM 1451 HG22 ILE A 144 9.488 15.239 -58.120 1.00 41.03 H
ATOM 1452 HG23 ILE A 144 7.873 15.601 -58.741 1.00 41.03 H
ATOM 1453 HD11 ILE A 144 8.202 17.942 -62.207 1.00 71.81 H
ATOM 1454 HD12 ILE A 144 9.694 17.173 -61.689 1.00 71.81 H
ATOM 1455 HD13 ILE A 144 8.134 16.363 -61.483 1.00 71.81 H
ATOM 1456 N SER A 145 10.384 17.070 -55.869 1.00 41.49 N
ATOM 1457 CA SER A 145 10.693 16.222 -54.723 1.00 41.47 C
ATOM 1458 C SER A 145 9.984 16.714 -53.467 1.00 37.02 C
ATOM 1459 O SER A 145 9.398 15.918 -52.724 1.00 40.57 O
ATOM 1460 CB SER A 145 12.205 16.162 -54.504 1.00 47.02 C
ATOM 1461 OG SER A 145 12.531 15.246 -53.471 1.00 52.17 O
ATOM 1462 H SER A 145 11.142 17.584 -56.299 1.00 41.49 H
ATOM 1463 HA SER A 145 10.333 15.214 -54.929 1.00 41.47 H
ATOM 1464 HB2 SER A 145 12.683 15.819 -55.421 1.00 47.02 H
ATOM 1465 HB3 SER A 145 12.610 17.146 -54.263 1.00 47.02 H
ATOM 1466 HG SER A 145 12.391 14.347 -53.788 1.00 52.17 H
ATOM 1467 N ILE A 146 10.026 18.025 -53.215 1.00 39.50 N
ATOM 1468 CA ILE A 146 9.304 18.590 -52.077 1.00 38.64 C
ATOM 1469 C ILE A 146 7.816 18.292 -52.195 1.00 38.73 C
ATOM 1470 O ILE A 146 7.149 17.966 -51.203 1.00 33.92 O
ATOM 1471 CB ILE A 146 9.530 20.137 -51.976 1.00 42.12 C
ATOM 1472 CG1 ILE A 146 10.956 20.426 -51.454 1.00 41.64 C
ATOM 1473 CG2 ILE A 146 8.501 20.948 -51.145 1.00 40.43 C
ATOM 1474 CD1 ILE A 146 11.388 21.892 -51.599 1.00 50.00 C
ATOM 1475 H ILE A 146 10.525 18.665 -53.821 1.00 39.50 H

ATOM 1476 HA ILE A 146 9.666 18.106 -51.168 1.00 38.64 H
ATOM 1477 HB ILE A 146 9.468 20.529 -52.993 1.00 42.12 H
ATOM 1478 HG12 ILE A 146 11.024 20.137 -50.404 1.00 41.64 H
ATOM 1479 HG13 ILE A 146 11.683 19.800 -51.974 1.00 41.64 H
ATOM 1480 HG21 ILE A 146 8.706 22.017 -51.190 1.00 40.43 H
ATOM 1481 HG22 ILE A 146 7.477 20.839 -51.503 1.00 40.43 H
ATOM 1482 HG23 ILE A 146 8.526 20.664 -50.095 1.00 40.43 H
ATOM 1483 HD11 ILE A 146 12.473 21.982 -51.555 1.00 50.00 H
ATOM 1484 HD12 ILE A 146 11.059 22.314 -52.549 1.00 50.00 H
ATOM 1485 HD13 ILE A 146 10.981 22.508 -50.797 1.00 50.00 H
ATOM 1486 N ASP A 147 7.277 18.373 -53.413 1.00 37.12 N
ATOM 1487 CA ASP A 147 5.846 18.169 -53.603 1.00 37.29 C
ATOM 1488 C ASP A 147 5.441 16.730 -53.303 1.00 38.62 C
ATOM 1489 O ASP A 147 4.434 16.494 -52.624 1.00 36.94 O
ATOM 1490 CB ASP A 147 5.442 18.560 -55.042 1.00 36.68 C
ATOM 1491 CG ASP A 147 3.944 18.700 -55.270 1.00 40.30 C
ATOM 1492 OD1 ASP A 147 3.218 19.019 -54.305 1.00 38.20 O
ATOM 1493 OD2 ASP A 147 3.561 18.607 -56.452 1.00 40.06 O1-
ATOM 1494 H ASP A 147 7.826 18.649 -54.218 1.00 37.12 H
ATOM 1495 HA ASP A 147 5.330 18.821 -52.893 1.00 37.29 H
ATOM 1496 HB2 ASP A 147 5.887 19.512 -55.321 1.00 36.68 H
ATOM 1497 HB3 ASP A 147 5.820 17.811 -55.739 1.00 36.68 H
ATOM 1498 N TYR A 148 6.206 15.751 -53.799 1.00 33.20 N
ATOM 1499 CA TYR A 148 5.888 14.360 -53.486 1.00 31.34 C
ATOM 1500 C TYR A 148 6.174 14.041 -52.022 1.00 37.64 C
ATOM 1501 O TYR A 148 5.376 13.359 -51.368 1.00 38.27 O
ATOM 1502 CB TYR A 148 6.673 13.398 -54.402 1.00 32.20 C
ATOM 1503 CG TYR A 148 6.039 13.123 -55.755 1.00 31.77 C
ATOM 1504 CD1 TYR A 148 6.634 13.601 -56.940 1.00 32.62 C
ATOM 1505 CD2 TYR A 148 4.863 12.345 -55.834 1.00 34.94 C
ATOM 1506 CE1 TYR A 148 6.090 13.265 -58.195 1.00 40.74 C
ATOM 1507 CE2 TYR A 148 4.308 12.025 -57.087 1.00 43.26 C
ATOM 1508 CZ TYR A 148 4.932 12.467 -58.269 1.00 41.39 C
ATOM 1509 OH TYR A 148 4.410 12.116 -59.479 1.00 39.52 O
ATOM 1510 H TYR A 148 7.017 15.948 -54.372 1.00 33.20 H
ATOM 1511 HA TYR A 148 4.822 14.191 -53.659 1.00 31.34 H
ATOM 1512 HB2 TYR A 148 7.704 13.733 -54.514 1.00 32.20 H
ATOM 1513 HB3 TYR A 148 6.741 12.420 -53.922 1.00 32.20 H
ATOM 1514 HD1 TYR A 148 7.522 14.213 -56.889 1.00 32.62 H
ATOM 1515 HD2 TYR A 148 4.379 11.987 -54.936 1.00 34.94 H

ATOM 1516 HE1 TYR A 148 6.570 13.622 -59.095 1.00 40.74 H
ATOM 1517 HE2 TYR A 148 3.405 11.435 -57.140 1.00 43.26 H
ATOM 1518 HH TYR A 148 4.930 12.431 -60.222 1.00 39.52 H
ATOM 1519 N TYR A 149 7.301 14.526 -51.491 1.00 35.85 N
ATOM 1520 CA TYR A 149 7.659 14.238 -50.103 1.00 37.55 C
ATOM 1521 C TYR A 149 6.558 14.668 -49.145 1.00 38.10 C
ATOM 1522 O TYR A 149 6.199 13.931 -48.221 1.00 36.32 O
ATOM 1523 CB TYR A 149 8.976 14.933 -49.677 1.00 33.32 C
ATOM 1524 CG TYR A 149 10.167 14.013 -49.510 1.00 43.06 C
ATOM 1525 CD1 TYR A 149 10.165 13.057 -48.472 1.00 40.48 C
ATOM 1526 CD2 TYR A 149 11.301 14.148 -50.336 1.00 41.57 C
ATOM 1527 CE1 TYR A 149 11.296 12.255 -48.250 1.00 39.96 C
ATOM 1528 CE2 TYR A 149 12.435 13.346 -50.110 1.00 42.24 C
ATOM 1529 CZ TYR A 149 12.437 12.409 -49.061 1.00 43.41 C
ATOM 1530 OH TYR A 149 13.550 11.663 -48.828 1.00 46.71 O
ATOM 1531 H TYR A 149 7.948 15.083 -52.037 1.00 35.85 H
ATOM 1532 HA TYR A 149 7.781 13.156 -50.010 1.00 37.55 H
ATOM 1533 HB2 TYR A 149 9.213 15.784 -50.310 1.00 33.32 H
ATOM 1534 HB3 TYR A 149 8.865 15.388 -48.689 1.00 33.32 H
ATOM 1535 HD1 TYR A 149 9.309 12.959 -47.818 1.00 40.48 H
ATOM 1536 HD2 TYR A 149 11.320 14.884 -51.128 1.00 41.57 H
ATOM 1537 HE1 TYR A 149 11.291 11.554 -47.427 1.00 39.96 H
ATOM 1538 HE2 TYR A 149 13.309 13.467 -50.735 1.00 42.24 H
ATOM 1539 HH TYR A 149 13.396 10.949 -48.177 1.00 46.71 H
ATOM 1540 N ASN A 150 6.008 15.859 -49.352 1.00 34.61 N
ATOM 1541 CA ASN A 150 5.089 16.429 -48.380 1.00 33.44 C
ATOM 1542 C ASN A 150 3.638 16.073 -48.645 1.00 35.99 C
ATOM 1543 O ASN A 150 2.818 16.172 -47.727 1.00 39.73 O
ATOM 1544 CB ASN A 150 5.308 17.944 -48.362 1.00 31.56 C
ATOM 1545 CG ASN A 150 6.670 18.246 -47.753 1.00 41.16 C
ATOM 1546 ND2 ASN A 150 7.632 18.679 -48.552 1.00 35.91 N
ATOM 1547 OD1 ASN A 150 6.878 18.014 -46.572 1.00 41.42 O
ATOM 1548 H ASN A 150 6.301 16.444 -50.124 1.00 34.61 H
ATOM 1549 HA ASN A 150 5.348 16.058 -47.394 1.00 33.44 H
ATOM 1550 HB2 ASN A 150 5.190 18.390 -49.351 1.00 31.56 H
ATOM 1551 HB3 ASN A 150 4.557 18.404 -47.727 1.00 31.56 H
ATOM 1552 HD21 ASN A 150 7.467 18.712 -49.557 1.00 35.91 H
ATOM 1553 HD22 ASN A 150 8.547 18.866 -48.175 1.00 35.91 H
ATOM 1554 N MET A 151 3.297 15.653 -49.862 1.00 32.39 N
ATOM 1555 CA MET A 151 2.027 14.963 -50.052 1.00 31.58 C

```
ATOM   1556  C   MET A 151       1.959  13.727 -49.164  1.00 36.94           C
ATOM   1557  O   MET A 151       0.981  13.520 -48.438  1.00 33.47           O
ATOM   1558  CB  MET A 151       1.993  14.510 -51.535  1.00 30.72           C
ATOM   1559  CG  MET A 151       0.770  13.724 -52.031  1.00 30.96           C
ATOM   1560  SD  MET A 151       1.078  12.919 -53.628  1.00 39.03           S
ATOM   1561  CE  MET A 151      -0.626  12.646 -54.176  1.00 51.80           C
ATOM   1562  H   MET A 151       3.968  15.625 -50.618  1.00 32.39           H
ATOM   1563  HA  MET A 151       1.213  15.634 -49.770  1.00 31.58           H
ATOM   1564  HB2 MET A 151       2.115  15.377 -52.169  1.00 30.72           H
ATOM   1565  HB3 MET A 151       2.873  13.904 -51.748  1.00 30.72           H
ATOM   1566  HG2 MET A 151       0.472  12.947 -51.328  1.00 30.96           H
ATOM   1567  HG3 MET A 151      -0.073  14.399 -52.144  1.00 30.96           H
ATOM   1568  HE1 MET A 151      -0.638  12.042 -55.082  1.00 51.80           H
ATOM   1569  HE2 MET A 151      -1.209  12.131 -53.413  1.00 51.80           H
ATOM   1570  HE3 MET A 151      -1.108  13.598 -54.397  1.00 51.80           H
ATOM   1571  N   PHE A 152       3.006  12.903 -49.191  1.00 31.61           N
ATOM   1572  CA  PHE A 152       2.995  11.671 -48.413  1.00 34.82           C
ATOM   1573  C   PHE A 152       3.178  11.940 -46.923  1.00 30.28           C
ATOM   1574  O   PHE A 152       2.533  11.290 -46.094  1.00 33.97           O
ATOM   1575  CB  PHE A 152       4.066  10.683 -48.917  1.00 30.42           C
ATOM   1576  CG  PHE A 152       3.748  10.015 -50.242  1.00 36.72           C
ATOM   1577  CD1 PHE A 152       2.652   9.131 -50.346  1.00 36.23           C
ATOM   1578  CD2 PHE A 152       4.500  10.314 -51.395  1.00 38.00           C
ATOM   1579  CE1 PHE A 152       2.339   8.563 -51.572  1.00 41.86           C
ATOM   1580  CE2 PHE A 152       4.147   9.764 -52.617  1.00 37.87           C
ATOM   1581  CZ  PHE A 152       3.070   8.893 -52.704  1.00 36.08           C
ATOM   1582  H   PHE A 152       3.805  13.082 -49.785  1.00 31.61           H
ATOM   1583  HA  PHE A 152       2.026  11.179 -48.526  1.00 34.82           H
ATOM   1584  HB2 PHE A 152       5.036  11.178 -48.977  1.00 30.42           H
ATOM   1585  HB3 PHE A 152       4.190   9.886 -48.185  1.00 30.42           H
ATOM   1586  HD1 PHE A 152       2.053   8.895 -49.478  1.00 36.23           H
ATOM   1587  HD2 PHE A 152       5.355  10.968 -51.337  1.00 38.00           H
ATOM   1588  HE1 PHE A 152       1.507   7.882 -51.651  1.00 41.86           H
ATOM   1589  HE2 PHE A 152       4.713  10.012 -53.501  1.00 37.87           H
ATOM   1590  HZ  PHE A 152       2.805   8.467 -53.658  1.00 36.08           H
ATOM   1591  N   THR A 153       4.047  12.889 -46.563  1.00 29.43           N
ATOM   1592  CA  THR A 153       4.245  13.211 -45.152  1.00 32.64           C
ATOM   1593  C   THR A 153       2.968  13.756 -44.525  1.00 40.17           C
ATOM   1594  O   THR A 153       2.606  13.376 -43.405  1.00 37.00           O
ATOM   1595  CB  THR A 153       5.393  14.209 -44.993  1.00 31.99           C
```

```
ATOM   1596  CG2 THR A 153       5.558  14.611 -43.527  1.00 39.47           C
ATOM   1597  OG1 THR A 153       6.611  13.610 -45.450  1.00 42.03           O
ATOM   1598  H   THR A 153       4.590  13.396 -47.250  1.00 29.43           H
ATOM   1599  HA  THR A 153       4.513  12.291 -44.626  1.00 32.64           H
ATOM   1600  HB  THR A 153       5.196  15.108 -45.566  1.00 31.99           H
ATOM   1601  HG1 THR A 153       6.547  13.481 -46.402  1.00 42.03           H
ATOM   1602 HG21 THR A 153       6.394  15.302 -43.415  1.00 39.47           H
ATOM   1603 HG22 THR A 153       4.690  15.099 -43.088  1.00 39.47           H
ATOM   1604 HG23 THR A 153       5.779  13.739 -42.910  1.00 39.47           H
ATOM   1605  N   SER A 154       2.269  14.648 -45.232  1.00 32.83           N
ATOM   1606  CA  SER A 154       0.988  15.145 -44.741  1.00 32.13           C
ATOM   1607  C   SER A 154      -0.010  14.006 -44.580  1.00 35.52           C
ATOM   1608  O   SER A 154      -0.648  13.862 -43.530  1.00 30.51           O
ATOM   1609  CB  SER A 154       0.450  16.215 -45.718  1.00 30.48           C
ATOM   1610  OG  SER A 154      -0.867  16.619 -45.403  1.00 40.32           O
ATOM   1611  H   SER A 154       2.574  14.963 -46.144  1.00 32.83           H
ATOM   1612  HA  SER A 154       1.140  15.609 -43.764  1.00 32.13           H
ATOM   1613  HB2 SER A 154       1.100  17.092 -45.724  1.00 30.48           H
ATOM   1614  HB3 SER A 154       0.433  15.822 -46.735  1.00 30.48           H
ATOM   1615  HG  SER A 154      -0.883  17.588 -45.336  1.00 40.32           H
ATOM   1616  N   ILE A 155      -0.138  13.172 -45.610  1.00 29.67           N
ATOM   1617  CA  ILE A 155      -1.135  12.110 -45.593  1.00 28.67           C
ATOM   1618  C   ILE A 155      -0.782  11.044 -44.558  1.00 31.40           C
ATOM   1619  O   ILE A 155      -1.655  10.559 -43.829  1.00 30.86           O
ATOM   1620  CB  ILE A 155      -1.284  11.508 -47.018  1.00 35.93           C
ATOM   1621  CG1 ILE A 155      -1.933  12.542 -47.974  1.00 34.60           C
ATOM   1622  CG2 ILE A 155      -2.057  10.183 -47.096  1.00 31.50           C
ATOM   1623  CD1 ILE A 155      -1.774  12.209 -49.464  1.00 40.15           C
ATOM   1624  H   ILE A 155       0.395  13.301 -46.460  1.00 29.67           H
ATOM   1625  HA  ILE A 155      -2.095  12.544 -45.313  1.00 28.67           H
ATOM   1626  HB  ILE A 155      -0.277  11.303 -47.388  1.00 35.93           H
ATOM   1627 HG12 ILE A 155      -2.992  12.642 -47.749  1.00 34.60           H
ATOM   1628 HG13 ILE A 155      -1.522  13.538 -47.805  1.00 34.60           H
ATOM   1629 HG21 ILE A 155      -2.135   9.867 -48.134  1.00 31.50           H
ATOM   1630 HG22 ILE A 155      -1.562   9.373 -46.560  1.00 31.50           H
ATOM   1631 HG23 ILE A 155      -3.068  10.289 -46.703  1.00 31.50           H
ATOM   1632 HD11 ILE A 155      -1.485  13.094 -50.027  1.00 40.15           H
ATOM   1633 HD12 ILE A 155      -1.012  11.449 -49.636  1.00 40.15           H
ATOM   1634 HD13 ILE A 155      -2.714  11.853 -49.886  1.00 40.15           H
ATOM   1635  N   PHE A 156       0.495  10.659 -44.476  1.00 27.48           N
```

ATOM	1636	CA	PHE	A	156	0.881	9.635	-43.509	1.00	34.73	C
ATOM	1637	C	PHE	A	156	0.725	10.129	-42.076	1.00	36.96	C
ATOM	1638	O	PHE	A	156	0.428	9.332	-41.179	1.00	32.87	O
ATOM	1639	CB	PHE	A	156	2.321	9.176	-43.753	1.00	35.29	C
ATOM	1640	CG	PHE	A	156	2.521	8.448	-45.059	1.00	35.90	C
ATOM	1641	CD1	PHE	A	156	1.441	7.961	-45.778	1.00	39.73	C
ATOM	1642	CD2	PHE	A	156	3.797	8.247	-45.560	1.00	34.82	C
ATOM	1643	CE1	PHE	A	156	1.630	7.293	-46.978	1.00	37.95	C
ATOM	1644	CE2	PHE	A	156	3.994	7.582	-46.758	1.00	39.17	C
ATOM	1645	CZ	PHE	A	156	2.909	7.104	-47.465	1.00	36.27	C
ATOM	1646	H	PHE	A	156	1.213	11.032	-45.088	1.00	27.48	H
ATOM	1647	HA	PHE	A	156	0.205	8.781	-43.564	1.00	34.73	H
ATOM	1648	HB2	PHE	A	156	3.001	10.029	-43.716	1.00	35.29	H
ATOM	1649	HB3	PHE	A	156	2.635	8.499	-42.958	1.00	35.29	H
ATOM	1650	HD1	PHE	A	156	0.418	8.068	-45.461	1.00	39.73	H
ATOM	1651	HD2	PHE	A	156	4.652	8.616	-45.013	1.00	34.82	H
ATOM	1652	HE1	PHE	A	156	0.780	6.920	-47.521	1.00	37.95	H
ATOM	1653	HE2	PHE	A	156	4.995	7.441	-47.138	1.00	39.17	H
ATOM	1654	HZ	PHE	A	156	3.060	6.583	-48.399	1.00	36.27	H
ATOM	1655	N	THR	A	157	0.912	11.431	-41.840	1.00	36.14	N
ATOM	1656	CA	THR	A	157	0.754	11.963	-40.490	1.00	29.55	C
ATOM	1657	C	THR	A	157	-0.707	11.959	-40.060	1.00	36.33	C
ATOM	1658	O	THR	A	157	-1.019	11.629	-38.911	1.00	37.69	O
ATOM	1659	CB	THR	A	157	1.336	13.374	-40.404	1.00	28.36	C
ATOM	1660	CG2	THR	A	157	1.236	13.909	-38.973	1.00	31.15	C
ATOM	1661	OG1	THR	A	157	2.713	13.341	-40.796	1.00	31.50	O
ATOM	1662	H	THR	A	157	1.152	12.075	-42.582	1.00	36.14	H
ATOM	1663	HA	THR	A	157	1.312	11.328	-39.798	1.00	29.55	H
ATOM	1664	HB	THR	A	157	0.818	14.035	-41.093	1.00	28.36	H
ATOM	1665	HG1	THR	A	157	2.749	13.224	-41.754	1.00	31.50	H
ATOM	1666	HG21	THR	A	157	1.804	14.833	-38.881	1.00	31.15	H
ATOM	1667	HG22	THR	A	157	0.211	14.137	-38.678	1.00	31.15	H
ATOM	1668	HG23	THR	A	157	1.649	13.206	-38.248	1.00	31.15	H
ATOM	1669	N	LEU	A	158	-1.623	12.314	-40.964	1.00	32.63	N
ATOM	1670	CA	LEU	A	158	-3.029	12.302	-40.580	1.00	37.38	C
ATOM	1671	C	LEU	A	158	-3.557	10.876	-40.452	1.00	34.87	C
ATOM	1672	O	LEU	A	158	-4.493	10.635	-39.682	1.00	39.21	O
ATOM	1673	CB	LEU	A	158	-3.859	13.128	-41.568	1.00	43.55	C
ATOM	1674	CG	LEU	A	158	-4.079	12.665	-43.005	1.00	41.78	C
ATOM	1675	CD1	LEU	A	158	-5.303	11.773	-43.095	1.00	34.21	C

```
ATOM   1676  CD2 LEU A 158      -4.220  13.866 -43.935  1.00 39.05           C
ATOM   1677  H   LEU A 158      -1.377  12.598 -41.904  1.00 32.63           H
ATOM   1678  HA  LEU A 158      -3.138  12.786 -39.606  1.00 37.38           H
ATOM   1679  HB2 LEU A 158      -4.823  13.359 -41.112  1.00 43.55           H
ATOM   1680  HB3 LEU A 158      -3.366  14.101 -41.604  1.00 43.55           H
ATOM   1681  HG  LEU A 158      -3.208  12.132 -43.328  1.00 41.78           H
ATOM   1682 HD11 LEU A 158      -6.042  12.153 -43.801  1.00 34.21           H
ATOM   1683 HD12 LEU A 158      -5.021  10.776 -43.437  1.00 34.21           H
ATOM   1684 HD13 LEU A 158      -5.820  11.646 -42.143  1.00 34.21           H
ATOM   1685 HD21 LEU A 158      -4.396  13.556 -44.964  1.00 39.05           H
ATOM   1686 HD22 LEU A 158      -5.048  14.510 -43.635  1.00 39.05           H
ATOM   1687 HD23 LEU A 158      -3.315  14.474 -43.938  1.00 39.05           H
ATOM   1688  N   CYS A 159      -2.972   9.921 -41.184  1.00 35.03           N
ATOM   1689  CA  CYS A 159      -3.260   8.515 -40.912  1.00 34.02           C
ATOM   1690  C   CYS A 159      -2.764   8.118 -39.529  1.00 29.94           C
ATOM   1691  O   CYS A 159      -3.438   7.370 -38.811  1.00 37.46           O
ATOM   1692  CB  CYS A 159      -2.617   7.615 -41.968  1.00 33.73           C
ATOM   1693  SG  CYS A 159      -3.343   7.719 -43.613  1.00 42.02           S
ATOM   1694  H   CYS A 159      -2.242  10.139 -41.849  1.00 35.03           H
ATOM   1695  HA  CYS A 159      -4.340   8.356 -40.943  1.00 34.02           H
ATOM   1696  HB2 CYS A 159      -1.551   7.823 -42.056  1.00 33.73           H
ATOM   1697  HB3 CYS A 159      -2.694   6.571 -41.664  1.00 33.73           H
ATOM   1698  HG  CYS A 159      -2.909   8.957 -43.874  1.00 42.02           H
ATOM   1699  N   THR A 160      -1.574   8.595 -39.153  1.00 35.02           N
ATOM   1700  CA  THR A 160      -1.020   8.286 -37.840  1.00 39.51           C
ATOM   1701  C   THR A 160      -1.908   8.825 -36.727  1.00 37.17           C
ATOM   1702  O   THR A 160      -2.106   8.161 -35.703  1.00 39.82           O
ATOM   1703  CB  THR A 160       0.393   8.858 -37.723  1.00 37.94           C
ATOM   1704  CG2 THR A 160       0.972   8.574 -36.345  1.00 31.21           C
ATOM   1705  OG1 THR A 160       1.232   8.260 -38.719  1.00 35.71           O
ATOM   1706  H   THR A 160      -1.034   9.192 -39.764  1.00 35.02           H
ATOM   1707  HA  THR A 160      -0.969   7.199 -37.738  1.00 39.51           H
ATOM   1708  HB  THR A 160       0.411   9.933 -37.884  1.00 37.94           H
ATOM   1709  HG1 THR A 160       0.904   8.517 -39.588  1.00 35.71           H
ATOM   1710 HG21 THR A 160       2.016   8.821 -36.388  1.00 31.21           H
ATOM   1711 HG22 THR A 160       0.533   9.208 -35.574  1.00 31.21           H
ATOM   1712 HG23 THR A 160       0.836   7.538 -36.042  1.00 31.21           H
ATOM   1713  N   MET A 161      -2.456  10.027 -36.912  1.00 35.47           N
ATOM   1714  CA  MET A 161      -3.374  10.575 -35.922  1.00 35.47           C
ATOM   1715  C   MET A 161      -4.644   9.738 -35.815  1.00 34.19           C
```

ATOM 1716 O MET A 161 -5.222 9.631 -34.729 1.00 35.82 O
ATOM 1717 CB MET A 161 -3.703 12.028 -36.265 1.00 40.25 C
ATOM 1718 CG MET A 161 -2.470 12.924 -36.312 1.00 49.70 C
ATOM 1719 SD MET A 161 -2.828 14.667 -36.605 1.00 53.76 S
ATOM 1720 CE MET A 161 -3.570 15.122 -35.040 1.00 66.13 C
ATOM 1721 H MET A 161 -2.263 10.573 -37.742 1.00 35.47 H
ATOM 1722 HA MET A 161 -2.881 10.566 -34.948 1.00 35.47 H
ATOM 1723 HB2 MET A 161 -4.215 12.078 -37.228 1.00 40.25 H
ATOM 1724 HB3 MET A 161 -4.409 12.416 -35.530 1.00 40.25 H
ATOM 1725 HG2 MET A 161 -1.954 12.860 -35.353 1.00 49.70 H
ATOM 1726 HG3 MET A 161 -1.738 12.616 -37.030 1.00 49.70 H
ATOM 1727 HE1 MET A 161 -3.718 16.199 -34.983 1.00 66.13 H
ATOM 1728 HE2 MET A 161 -4.536 14.636 -34.908 1.00 66.13 H
ATOM 1729 HE3 MET A 161 -2.920 14.833 -34.214 1.00 66.13 H
ATOM 1730 N SER A 162 -5.083 9.125 -36.916 1.00 28.80 N
ATOM 1731 CA SER A 162 -6.237 8.233 -36.841 1.00 30.96 C
ATOM 1732 C SER A 162 -5.893 6.949 -36.097 1.00 27.50 C
ATOM 1733 O SER A 162 -6.664 6.490 -35.246 1.00 33.85 O
ATOM 1734 CB SER A 162 -6.751 7.912 -38.245 1.00 33.77 C
ATOM 1735 OG SER A 162 -7.884 7.067 -38.177 1.00 47.77 O
ATOM 1736 H SER A 162 -4.607 9.219 -37.803 1.00 28.80 H
ATOM 1737 HA SER A 162 -7.038 8.733 -36.294 1.00 30.96 H
ATOM 1738 HB2 SER A 162 -7.028 8.829 -38.767 1.00 33.77 H
ATOM 1739 HB3 SER A 162 -5.992 7.417 -38.850 1.00 33.77 H
ATOM 1740 HG SER A 162 -8.627 7.572 -37.836 1.00 47.77 H
ATOM 1741 N VAL A 163 -4.747 6.345 -36.421 1.00 30.31 N
ATOM 1742 CA VAL A 163 -4.311 5.136 -35.728 1.00 30.40 C
ATOM 1743 C VAL A 163 -4.081 5.426 -34.250 1.00 34.14 C
ATOM 1744 O VAL A 163 -4.410 4.611 -33.379 1.00 37.21 O
ATOM 1745 CB VAL A 163 -3.044 4.572 -36.397 1.00 31.52 C
ATOM 1746 CG1 VAL A 163 -2.474 3.417 -35.586 1.00 32.66 C
ATOM 1747 CG2 VAL A 163 -3.347 4.134 -37.825 1.00 26.59 C
ATOM 1748 H VAL A 163 -4.143 6.720 -37.142 1.00 30.31 H
ATOM 1749 HA VAL A 163 -5.103 4.390 -35.807 1.00 30.40 H
ATOM 1750 HB VAL A 163 -2.289 5.358 -36.448 1.00 31.52 H
ATOM 1751 HG11 VAL A 163 -1.684 2.910 -36.141 1.00 32.66 H
ATOM 1752 HG12 VAL A 163 -2.021 3.747 -34.651 1.00 32.66 H
ATOM 1753 HG13 VAL A 163 -3.236 2.672 -35.353 1.00 32.66 H
ATOM 1754 HG21 VAL A 163 -2.430 3.861 -38.348 1.00 26.59 H
ATOM 1755 HG22 VAL A 163 -3.996 3.259 -37.837 1.00 26.59 H

ATOM 1756 HG23 VAL A 163 -3.835 4.906 -38.416 1.00 26.59 H
ATOM 1757 N ASP A 164 -3.512 6.593 -33.948 1.00 33.76 N
ATOM 1758 CA ASP A 164 -3.257 6.973 -32.564 1.00 30.01 C
ATOM 1759 C ASP A 164 -4.549 7.039 -31.758 1.00 34.12 C
ATOM 1760 O ASP A 164 -4.601 6.574 -30.613 1.00 35.72 O
ATOM 1761 CB ASP A 164 -2.532 8.317 -32.535 1.00 34.22 C
ATOM 1762 CG ASP A 164 -2.344 8.849 -31.135 1.00 38.67 C
ATOM 1763 OD1 ASP A 164 -1.590 8.222 -30.361 1.00 41.22 O
ATOM 1764 OD2 ASP A 164 -2.941 9.900 -30.816 1.00 42.20 O1-
ATOM 1765 H ASP A 164 -3.210 7.234 -34.673 1.00 33.76 H
ATOM 1766 HA ASP A 164 -2.637 6.200 -32.111 1.00 30.01 H
ATOM 1767 HB2 ASP A 164 -1.555 8.257 -33.016 1.00 34.22 H
ATOM 1768 HB3 ASP A 164 -3.087 9.057 -33.111 1.00 34.22 H
ATOM 1769 N ARG A 165 -5.604 7.614 -32.340 1.00 31.74 N
ATOM 1770 CA ARG A 165 -6.882 7.694 -31.643 1.00 36.70 C
ATOM 1771 C ARG A 165 -7.556 6.331 -31.555 1.00 38.55 C
ATOM 1772 O ARG A 165 -8.209 6.025 -30.549 1.00 34.10 O
ATOM 1773 CB ARG A 165 -7.790 8.707 -32.343 1.00 37.46 C
ATOM 1774 CG ARG A 165 -7.262 10.130 -32.279 1.00 39.84 C
ATOM 1775 CD ARG A 165 -7.852 11.001 -33.378 1.00 47.40 C
ATOM 1776 NE ARG A 165 -7.454 12.398 -33.230 1.00 52.02 N
ATOM 1777 CZ ARG A 165 -7.756 13.359 -34.096 1.00 56.13 C
ATOM 1778 NH1 ARG A 165 -8.460 13.076 -35.186 1.00 47.41 N1+
ATOM 1779 NH2 ARG A 165 -7.350 14.601 -33.872 1.00 58.79 N1+
ATOM 1780 H ARG A 165 -5.543 8.003 -33.271 1.00 31.74 H
ATOM 1781 HA ARG A 165 -6.711 8.050 -30.624 1.00 36.70 H
ATOM 1782 HB2 ARG A 165 -7.902 8.403 -33.386 1.00 37.46 H
ATOM 1783 HB3 ARG A 165 -8.792 8.690 -31.913 1.00 37.46 H
ATOM 1784 HG2 ARG A 165 -7.380 10.569 -31.287 1.00 39.84 H
ATOM 1785 HG3 ARG A 165 -6.187 10.112 -32.452 1.00 39.84 H
ATOM 1786 HD2 ARG A 165 -7.461 10.679 -34.342 1.00 47.40 H
ATOM 1787 HD3 ARG A 165 -8.936 10.898 -33.425 1.00 47.40 H
ATOM 1788 HE ARG A 165 -6.926 12.631 -32.401 1.00 52.02 H
ATOM 1789 HH11 ARG A 165 -8.711 12.119 -35.403 1.00 47.41 H
ATOM 1790 HH12 ARG A 165 -8.712 13.806 -35.835 1.00 47.41 H
ATOM 1791 HH21 ARG A 165 -6.820 14.825 -33.042 1.00 58.79 H
ATOM 1792 HH22 ARG A 165 -7.591 15.343 -34.513 1.00 58.79 H
ATOM 1793 N TYR A 166 -7.414 5.504 -32.593 1.00 35.25 N
ATOM 1794 CA TYR A 166 -7.940 4.146 -32.529 1.00 33.77 C
ATOM 1795 C TYR A 166 -7.273 3.350 -31.412 1.00 28.93 C

ATOM 1796 O TYR A 166 -7.945 2.634 -30.661 1.00 36.35 O
ATOM 1797 CB TYR A 166 -7.754 3.451 -33.880 1.00 32.15 C
ATOM 1798 CG TYR A 166 -7.803 1.944 -33.799 1.00 32.95 C
ATOM 1799 CD1 TYR A 166 -9.014 1.275 -33.710 1.00 33.23 C
ATOM 1800 CD2 TYR A 166 -6.634 1.190 -33.815 1.00 34.57 C
ATOM 1801 CE1 TYR A 166 -9.065 -0.103 -33.633 1.00 37.25 C
ATOM 1802 CE2 TYR A 166 -6.674 -0.189 -33.739 1.00 31.86 C
ATOM 1803 CZ TYR A 166 -7.894 -0.830 -33.647 1.00 36.03 C
ATOM 1804 OH TYR A 166 -7.946 -2.203 -33.569 1.00 44.80 O
ATOM 1805 H TYR A 166 -6.903 5.779 -33.422 1.00 35.25 H
ATOM 1806 HA TYR A 166 -9.010 4.197 -32.317 1.00 33.77 H
ATOM 1807 HB2 TYR A 166 -8.536 3.800 -34.549 1.00 32.15 H
ATOM 1808 HB3 TYR A 166 -6.803 3.733 -34.331 1.00 32.15 H
ATOM 1809 HD1 TYR A 166 -9.936 1.838 -33.691 1.00 33.23 H
ATOM 1810 HD2 TYR A 166 -5.677 1.686 -33.888 1.00 34.57 H
ATOM 1811 HE1 TYR A 166 -10.015 -0.607 -33.544 1.00 37.25 H
ATOM 1812 HE2 TYR A 166 -5.755 -0.756 -33.749 1.00 31.86 H
ATOM 1813 HH TYR A 166 -7.083 -2.606 -33.450 1.00 44.80 H
ATOM 1814 N ILE A 167 -5.950 3.475 -31.281 1.00 30.53 N
ATOM 1815 CA ILE A 167 -5.230 2.763 -30.229 1.00 33.93 C
ATOM 1816 C ILE A 167 -5.670 3.249 -28.852 1.00 37.15 C
ATOM 1817 O ILE A 167 -5.831 2.451 -27.920 1.00 34.00 O
ATOM 1818 CB ILE A 167 -3.712 2.917 -30.436 1.00 39.01 C
ATOM 1819 CG1 ILE A 167 -3.264 2.111 -31.659 1.00 38.10 C
ATOM 1820 CG2 ILE A 167 -2.934 2.495 -29.188 1.00 35.40 C
ATOM 1821 CD1 ILE A 167 -1.782 2.220 -31.953 1.00 35.60 C
ATOM 1822 H ILE A 167 -5.411 4.051 -31.916 1.00 30.53 H
ATOM 1823 HA ILE A 167 -5.483 1.702 -30.296 1.00 33.93 H
ATOM 1824 HB ILE A 167 -3.487 3.968 -30.628 1.00 39.01 H
ATOM 1825 HG12 ILE A 167 -3.497 1.059 -31.490 1.00 38.10 H
ATOM 1826 HG13 ILE A 167 -3.826 2.382 -32.548 1.00 38.10 H
ATOM 1827 HG21 ILE A 167 -1.857 2.534 -29.345 1.00 35.40 H
ATOM 1828 HG22 ILE A 167 -3.107 3.165 -28.349 1.00 35.40 H
ATOM 1829 HG23 ILE A 167 -3.178 1.481 -28.875 1.00 35.40 H
ATOM 1830 HD11 ILE A 167 -1.573 1.927 -32.982 1.00 35.60 H
ATOM 1831 HD12 ILE A 167 -1.429 3.243 -31.831 1.00 35.60 H
ATOM 1832 HD13 ILE A 167 -1.189 1.571 -31.308 1.00 35.60 H
ATOM 1833 N ALA A 168 -5.895 4.557 -28.708 1.00 34.16 N
ATOM 1834 CA ALA A 168 -6.291 5.111 -27.416 1.00 33.98 C
ATOM 1835 C ALA A 168 -7.602 4.509 -26.925 1.00 36.35 C

ATOM	1836	O	ALA	A	168	-7.765	4.248	-25.726	1.00	39.08	O
ATOM	1837	CB	ALA	A	168	-6.409	6.631	-27.516	1.00	33.75	C
ATOM	1838	H	ALA	A	168	-5.755	5.200	-29.476	1.00	34.16	H
ATOM	1839	HA	ALA	A	168	-5.514	4.881	-26.688	1.00	33.98	H
ATOM	1840	HB1	ALA	A	168	-6.584	7.077	-26.537	1.00	33.75	H
ATOM	1841	HB2	ALA	A	168	-5.498	7.064	-27.928	1.00	33.75	H
ATOM	1842	HB3	ALA	A	168	-7.230	6.931	-28.168	1.00	33.75	H
ATOM	1843	N	VAL	A	169	-8.544	4.275	-27.831	1.00	33.09	N
ATOM	1844	CA	VAL	A	169	-9.868	3.792	-27.450	1.00	33.26	C
ATOM	1845	C	VAL	A	169	-9.922	2.272	-27.423	1.00	36.39	C
ATOM	1846	O	VAL	A	169	-10.458	1.676	-26.485	1.00	35.47	O
ATOM	1847	CB	VAL	A	169	-10.933	4.374	-28.404	1.00	37.14	C
ATOM	1848	CG1	VAL	A	169	-12.293	3.754	-28.131	1.00	36.50	C
ATOM	1849	CG2	VAL	A	169	-10.996	5.882	-28.262	1.00	35.69	C
ATOM	1850	H	VAL	A	169	-8.388	4.494	-28.806	1.00	33.09	H
ATOM	1851	HA	VAL	A	169	-10.120	4.137	-26.444	1.00	33.26	H
ATOM	1852	HB	VAL	A	169	-10.650	4.128	-29.428	1.00	37.14	H
ATOM	1853	HG11	VAL	A	169	-13.035	4.223	-28.770	1.00	36.50	H
ATOM	1854	HG12	VAL	A	169	-12.329	2.698	-28.386	1.00	36.50	H
ATOM	1855	HG13	VAL	A	169	-12.607	3.878	-27.094	1.00	36.50	H
ATOM	1856	HG21	VAL	A	169	-11.814	6.310	-28.841	1.00	35.69	H
ATOM	1857	HG22	VAL	A	169	-11.158	6.175	-27.224	1.00	35.69	H
ATOM	1858	HG23	VAL	A	169	-10.077	6.361	-28.600	1.00	35.69	H
ATOM	1859	N	CYS	A	170	-9.369	1.620	-28.447	1.00	33.56	N
ATOM	1860	CA	CYS	A	170	-9.541	0.182	-28.605	1.00	38.11	C
ATOM	1861	C	CYS	A	170	-8.427	-0.636	-27.969	1.00	36.89	C
ATOM	1862	O	CYS	A	170	-8.627	-1.830	-27.718	1.00	40.09	O
ATOM	1863	CB	CYS	A	170	-9.658	-0.175	-30.092	1.00	34.39	C
ATOM	1864	SG	CYS	A	170	-11.144	0.511	-30.883	1.00	40.02	S
ATOM	1865	H	CYS	A	170	-8.913	2.118	-29.202	1.00	33.56	H
ATOM	1866	HA	CYS	A	170	-10.477	-0.135	-28.147	1.00	38.11	H
ATOM	1867	HB2	CYS	A	170	-8.779	0.161	-30.642	1.00	34.39	H
ATOM	1868	HB3	CYS	A	170	-9.699	-1.257	-30.223	1.00	34.39	H
ATOM	1869	HG	CYS	A	170	-12.021	-0.249	-30.221	1.00	40.02	H
ATOM	1870	N	HIS	A	171	-7.269	-0.036	-27.701	1.00	32.66	N
ATOM	1871	CA	HIS	A	171	-6.164	-0.713	-27.019	1.00	33.10	C
ATOM	1872	C	HIS	A	171	-5.583	0.209	-25.953	1.00	34.78	C
ATOM	1873	O	HIS	A	171	-4.414	0.605	-26.023	1.00	36.09	O
ATOM	1874	CB	HIS	A	171	-5.092	-1.142	-28.021	1.00	39.44	C
ATOM	1875	CG	HIS	A	171	-5.622	-1.984	-29.141	1.00	44.90	C

ATOM	1876	CD2	HIS	A	171	-5.968	-1.663	-30.410	1.00	44.59	C
ATOM	1877	ND1	HIS	A	171	-5.866	-3.334	-29.007	1.00	46.20	N
ATOM	1878	CE1	HIS	A	171	-6.331	-3.810	-30.149	1.00	46.37	C
ATOM	1879	NE2	HIS	A	171	-6.402	-2.817	-31.017	1.00	43.11	N
ATOM	1880	H	HIS	A	171	-7.133	0.944	-27.916	1.00	32.66	H
ATOM	1881	HA	HIS	A	171	-6.503	-1.614	-26.505	1.00	33.10	H
ATOM	1882	HB2	HIS	A	171	-4.611	-0.272	-28.470	1.00	39.44	H
ATOM	1883	HB3	HIS	A	171	-4.314	-1.707	-27.510	1.00	39.44	H
ATOM	1884	HD1	HIS	A	171	-5.737	-3.871	-28.162	1.00	46.20	H
ATOM	1885	HD2	HIS	A	171	-5.944	-0.713	-30.924	1.00	44.59	H
ATOM	1886	HE1	HIS	A	171	-6.618	-4.834	-30.339	1.00	46.37	H
ATOM	1887	N	PRO	A	172	-6.378	0.562	-24.938	1.00	35.33	N
ATOM	1888	CA	PRO	A	172	-5.914	1.566	-23.963	1.00	34.03	C
ATOM	1889	C	PRO	A	172	-4.682	1.147	-23.181	1.00	42.17	C
ATOM	1890	O	PRO	A	172	-3.905	2.015	-22.763	1.00	42.46	O
ATOM	1891	CB	PRO	A	172	-7.132	1.746	-23.046	1.00	32.29	C
ATOM	1892	CG	PRO	A	172	-7.886	0.461	-23.163	1.00	33.23	C
ATOM	1893	CD	PRO	A	172	-7.709	0.031	-24.593	1.00	37.37	C
ATOM	1894	HA	PRO	A	172	-5.699	2.509	-24.470	1.00	34.03	H
ATOM	1895	HB2	PRO	A	172	-6.879	1.988	-22.012	1.00	32.29	H
ATOM	1896	HB3	PRO	A	172	-7.747	2.563	-23.426	1.00	32.29	H
ATOM	1897	HG2	PRO	A	172	-7.430	-0.281	-22.506	1.00	33.23	H
ATOM	1898	HG3	PRO	A	172	-8.933	0.553	-22.874	1.00	33.23	H
ATOM	1899	HD2	PRO	A	172	-7.794	-1.051	-24.701	1.00	37.37	H
ATOM	1900	HD3	PRO	A	172	-8.456	0.508	-25.225	1.00	37.37	H
ATOM	1901	N	VAL	A	173	-4.473	-0.153	-22.963	1.00	35.97	N
ATOM	1902	CA	VAL	A	173	-3.271	-0.592	-22.260	1.00	38.72	C
ATOM	1903	C	VAL	A	173	-2.040	-0.387	-23.136	1.00	38.98	C
ATOM	1904	O	VAL	A	173	-1.029	0.172	-22.695	1.00	40.23	O
ATOM	1905	CB	VAL	A	173	-3.410	-2.057	-21.813	1.00	45.97	C
ATOM	1906	CG1	VAL	A	173	-2.090	-2.561	-21.248	1.00	47.22	C
ATOM	1907	CG2	VAL	A	173	-4.515	-2.186	-20.774	1.00	45.55	C
ATOM	1908	H	VAL	A	173	-5.121	-0.856	-23.286	1.00	35.97	H
ATOM	1909	HA	VAL	A	173	-3.142	0.019	-21.363	1.00	38.72	H
ATOM	1910	HB	VAL	A	173	-3.677	-2.680	-22.668	1.00	45.97	H
ATOM	1911	HG11	VAL	A	173	-2.220	-3.535	-20.776	1.00	47.22	H
ATOM	1912	HG12	VAL	A	173	-1.326	-2.700	-22.014	1.00	47.22	H
ATOM	1913	HG13	VAL	A	173	-1.693	-1.887	-20.488	1.00	47.22	H
ATOM	1914	HG21	VAL	A	173	-4.616	-3.218	-20.436	1.00	45.55	H
ATOM	1915	HG22	VAL	A	173	-4.312	-1.571	-19.897	1.00	45.55	H

ATOM 1916 HG23 VAL A 173 -5.484 -1.885 -21.173 1.00 45.55 H
ATOM 1917 N LYS A 174 -2.108 -0.831 -24.392 1.00 37.75 N
ATOM 1918 CA LYS A 174 -1.011 -0.593 -25.322 1.00 41.25 C
ATOM 1919 C LYS A 174 -0.820 0.891 -25.610 1.00 42.02 C
ATOM 1920 O LYS A 174 0.294 1.308 -25.942 1.00 40.81 O
ATOM 1921 CB LYS A 174 -1.253 -1.363 -26.623 1.00 45.68 C
ATOM 1922 CG LYS A 174 -1.259 -2.879 -26.447 1.00 60.42 C
ATOM 1923 CD LYS A 174 -1.661 -3.594 -27.726 1.00 66.87 C
ATOM 1924 CE LYS A 174 -1.685 -5.103 -27.528 1.00 70.70 C
ATOM 1925 NZ LYS A 174 -2.132 -5.817 -28.757 1.00 73.90 N1+
ATOM 1926 H LYS A 174 -2.941 -1.277 -24.747 1.00 37.75 H
ATOM 1927 HA LYS A 174 -0.086 -0.966 -24.874 1.00 41.25 H
ATOM 1928 HB2 LYS A 174 -2.194 -1.033 -27.063 1.00 45.68 H
ATOM 1929 HB3 LYS A 174 -0.479 -1.107 -27.349 1.00 45.68 H
ATOM 1930 HG2 LYS A 174 -0.269 -3.211 -26.130 1.00 60.42 H
ATOM 1931 HG3 LYS A 174 -1.948 -3.162 -25.650 1.00 60.42 H
ATOM 1932 HD2 LYS A 174 -2.643 -3.251 -28.053 1.00 66.87 H
ATOM 1933 HD3 LYS A 174 -0.963 -3.338 -28.524 1.00 66.87 H
ATOM 1934 HE2 LYS A 174 -0.694 -5.466 -27.252 1.00 70.70 H
ATOM 1935 HE3 LYS A 174 -2.362 -5.367 -26.714 1.00 70.70 H
ATOM 1936 HZ1 LYS A 174 -3.061 -5.512 -29.009 1.00 73.90 H
ATOM 1937 HZ2 LYS A 174 -1.496 -5.614 -29.515 1.00 73.90 H
ATOM 1938 HZ3 LYS A 174 -2.139 -6.812 -28.585 1.00 73.90 H
ATOM 1939 N ALA A 175 -1.878 1.696 -25.475 1.00 36.08 N
ATOM 1940 CA ALA A 175 -1.752 3.135 -25.676 1.00 31.90 C
ATOM 1941 C ALA A 175 -0.753 3.754 -24.709 1.00 34.40 C
ATOM 1942 O ALA A 175 -0.133 4.775 -25.027 1.00 37.29 O
ATOM 1943 CB ALA A 175 -3.118 3.808 -25.525 1.00 31.50 C
ATOM 1944 H ALA A 175 -2.791 1.329 -25.238 1.00 36.08 H
ATOM 1945 HA ALA A 175 -1.392 3.309 -26.691 1.00 31.90 H
ATOM 1946 HB1 ALA A 175 -3.058 4.867 -25.744 1.00 31.50 H
ATOM 1947 HB2 ALA A 175 -3.857 3.361 -26.188 1.00 31.50 H
ATOM 1948 HB3 ALA A 175 -3.498 3.731 -24.510 1.00 31.50 H
ATOM 1949 N LEU A 176 -0.583 3.153 -23.527 1.00 33.86 N
ATOM 1950 CA LEU A 176 0.374 3.675 -22.557 1.00 37.50 C
ATOM 1951 C LEU A 176 1.800 3.627 -23.093 1.00 46.13 C
ATOM 1952 O LEU A 176 2.621 4.487 -22.750 1.00 42.85 O
ATOM 1953 CB LEU A 176 0.269 2.890 -21.245 1.00 33.54 C
ATOM 1954 CG LEU A 176 -1.062 2.977 -20.494 1.00 38.83 C
ATOM 1955 CD1 LEU A 176 -1.088 2.017 -19.304 1.00 39.67 C

ATOM	1956	CD2	LEU	A	176	-1.326	4.404	-20.030	1.00	34.89	C
ATOM	1957	H	LEU	A	176	-1.091	2.314	-23.283	1.00	33.86	H
ATOM	1958	HA	LEU	A	176	0.134	4.722	-22.363	1.00	37.50	H
ATOM	1959	HB2	LEU	A	176	0.494	1.844	-21.455	1.00	33.54	H
ATOM	1960	HB3	LEU	A	176	1.063	3.216	-20.570	1.00	33.54	H
ATOM	1961	HG	LEU	A	176	-1.863	2.677	-21.169	1.00	38.83	H
ATOM	1962	HD11	LEU	A	176	-1.990	1.404	-19.327	1.00	39.67	H
ATOM	1963	HD12	LEU	A	176	-0.241	1.330	-19.314	1.00	39.67	H
ATOM	1964	HD13	LEU	A	176	-1.064	2.523	-18.338	1.00	39.67	H
ATOM	1965	HD21	LEU	A	176	-1.788	4.457	-19.044	1.00	34.89	H
ATOM	1966	HD22	LEU	A	176	-0.416	5.003	-19.990	1.00	34.89	H
ATOM	1967	HD23	LEU	A	176	-2.010	4.902	-20.718	1.00	34.89	H
ATOM	1968	N	ASP	A	177	2.111	2.637	-23.931	1.00	45.43	N
ATOM	1969	CA	ASP	A	177	3.427	2.532	-24.548	1.00	43.87	C
ATOM	1970	C	ASP	A	177	3.520	3.286	-25.868	1.00	44.03	C
ATOM	1971	O	ASP	A	177	4.584	3.828	-26.188	1.00	45.83	O
ATOM	1972	CB	ASP	A	177	3.784	1.061	-24.786	1.00	51.38	C
ATOM	1973	CG	ASP	A	177	3.799	0.247	-23.502	1.00	66.86	C
ATOM	1974	OD1	ASP	A	177	4.270	0.768	-22.470	1.00	72.14	O
ATOM	1975	OD2	ASP	A	177	3.335	-0.913	-23.527	1.00	67.38	O1-
ATOM	1976	H	ASP	A	177	1.422	1.944	-24.189	1.00	45.43	H
ATOM	1977	HA	ASP	A	177	4.191	2.954	-23.890	1.00	43.87	H
ATOM	1978	HB2	ASP	A	177	3.049	0.619	-25.462	1.00	51.38	H
ATOM	1979	HB3	ASP	A	177	4.758	0.962	-25.266	1.00	51.38	H
ATOM	1980	N	PHE	A	178	2.432	3.342	-26.637	1.00	39.67	N
ATOM	1981	CA	PHE	A	178	2.500	3.931	-27.971	1.00	36.59	C
ATOM	1982	C	PHE	A	178	2.442	5.454	-27.939	1.00	42.67	C
ATOM	1983	O	PHE	A	178	3.086	6.115	-28.761	1.00	41.21	O
ATOM	1984	CB	PHE	A	178	1.373	3.382	-28.848	1.00	39.40	C
ATOM	1985	CG	PHE	A	178	1.272	4.052	-30.193	1.00	50.00	C
ATOM	1986	CD1	PHE	A	178	2.169	3.746	-31.204	1.00	54.30	C
ATOM	1987	CD2	PHE	A	178	0.280	4.988	-30.446	1.00	48.54	C
ATOM	1988	CE1	PHE	A	178	2.079	4.360	-32.442	1.00	50.61	C
ATOM	1989	CE2	PHE	A	178	0.186	5.606	-31.682	1.00	52.43	C
ATOM	1990	CZ	PHE	A	178	1.087	5.290	-32.680	1.00	47.01	C
ATOM	1991	H	PHE	A	178	1.572	2.887	-26.358	1.00	39.67	H
ATOM	1992	HA	PHE	A	178	3.439	3.647	-28.450	1.00	36.59	H
ATOM	1993	HB2	PHE	A	178	1.525	2.314	-29.009	1.00	39.40	H
ATOM	1994	HB3	PHE	A	178	0.417	3.471	-28.329	1.00	39.40	H
ATOM	1995	HD1	PHE	A	178	2.951	3.022	-31.028	1.00	54.30	H

ATOM 1996 HD2 PHE A 178 -0.435 5.237 -29.678 1.00 48.54 H
ATOM 1997 HE1 PHE A 178 2.791 4.098 -33.207 1.00 50.61 H
ATOM 1998 HE2 PHE A 178 -0.563 6.354 -31.862 1.00 52.43 H
ATOM 1999 HZ PHE A 178 1.005 5.773 -33.642 1.00 47.01 H
ATOM 2000 N ARG A 179 1.681 6.034 -27.016 1.00 36.86 N
ATOM 2001 CA ARG A 179 1.359 7.460 -27.103 1.00 35.56 C
ATOM 2002 C ARG A 179 2.346 8.327 -26.328 1.00 37.85 C
ATOM 2003 O ARG A 179 1.969 9.254 -25.611 1.00 45.57 O
ATOM 2004 CB ARG A 179 -0.069 7.695 -26.633 1.00 38.55 C
ATOM 2005 CG ARG A 179 -1.093 6.859 -27.382 1.00 40.42 C
ATOM 2006 CD ARG A 179 -2.502 7.285 -27.030 1.00 40.38 C
ATOM 2007 NE ARG A 179 -2.949 8.418 -27.834 1.00 35.37 N
ATOM 2008 CZ ARG A 179 -3.931 9.239 -27.479 1.00 42.06 C
ATOM 2009 NH1 ARG A 179 -4.557 9.066 -26.321 1.00 37.45 N1+
ATOM 2010 NH2 ARG A 179 -4.280 10.239 -28.277 1.00 39.24 N1+
ATOM 2011 H ARG A 179 1.185 5.487 -26.323 1.00 36.86 H
ATOM 2012 HA ARG A 179 1.399 7.803 -28.139 1.00 35.56 H
ATOM 2013 HB2 ARG A 179 -0.150 7.492 -25.564 1.00 38.55 H
ATOM 2014 HB3 ARG A 179 -0.317 8.750 -26.768 1.00 38.55 H
ATOM 2015 HG2 ARG A 179 -0.916 7.023 -28.437 1.00 40.42 H
ATOM 2016 HG3 ARG A 179 -0.971 5.787 -27.236 1.00 40.42 H
ATOM 2017 HD2 ARG A 179 -3.218 6.470 -27.094 1.00 40.38 H
ATOM 2018 HD3 ARG A 179 -2.490 7.625 -25.994 1.00 40.38 H
ATOM 2019 HE ARG A 179 -2.519 8.517 -28.753 1.00 35.37 H
ATOM 2020 HH11 ARG A 179 -4.291 8.305 -25.714 1.00 37.45 H
ATOM 2021 HH12 ARG A 179 -5.296 9.692 -26.038 1.00 37.45 H
ATOM 2022 HH21 ARG A 179 -3.817 10.331 -29.179 1.00 39.24 H
ATOM 2023 HH22 ARG A 179 -5.012 10.884 -28.022 1.00 39.24 H
ATOM 2024 N THR A 180 3.613 8.037 -26.485 1.00 36.81 N
ATOM 2025 CA THR A 180 4.678 8.812 -25.876 1.00 39.33 C
ATOM 2026 C THR A 180 5.278 9.777 -26.890 1.00 38.70 C
ATOM 2027 O THR A 180 5.229 9.524 -28.098 1.00 35.56 O
ATOM 2028 CB THR A 180 5.784 7.895 -25.348 1.00 44.70 C
ATOM 2029 CG2 THR A 180 5.215 6.872 -24.379 1.00 47.87 C
ATOM 2030 OG1 THR A 180 6.400 7.211 -26.447 1.00 42.00 O
ATOM 2031 H THR A 180 3.884 7.293 -27.112 1.00 36.81 H
ATOM 2032 HA THR A 180 4.290 9.366 -25.022 1.00 39.33 H
ATOM 2033 HB THR A 180 6.553 8.478 -24.839 1.00 44.70 H
ATOM 2034 HG1 THR A 180 5.849 6.458 -26.681 1.00 42.00 H
ATOM 2035 HG21 THR A 180 6.009 6.250 -23.964 1.00 47.87 H

```
ATOM   2036 HG22 THR A 180       4.714   7.360 -23.542  1.00 47.87           H
ATOM   2037 HG23 THR A 180       4.495   6.200 -24.847  1.00 47.87           H
ATOM   2038  N   PRO A 181       5.845  10.895 -26.429  1.00 40.61           N
ATOM   2039  CA  PRO A 181       6.572  11.772 -27.360  1.00 39.23           C
ATOM   2040  C   PRO A 181       7.720  11.071 -28.062  1.00 42.47           C
ATOM   2041  O   PRO A 181       7.998  11.372 -29.230  1.00 36.97           O
ATOM   2042  CB  PRO A 181       7.067  12.916 -26.458  1.00 41.18           C
ATOM   2043  CG  PRO A 181       6.937  12.409 -25.049  1.00 43.69           C
ATOM   2044  CD  PRO A 181       5.788  11.451 -25.065  1.00 42.41           C
ATOM   2045  HA  PRO A 181       5.880  12.152 -28.111  1.00 39.23           H
ATOM   2046  HB2 PRO A 181       8.078  13.259 -26.682  1.00 41.18           H
ATOM   2047  HB3 PRO A 181       6.406  13.773 -26.592  1.00 41.18           H
ATOM   2048  HG2 PRO A 181       7.845  11.862 -24.791  1.00 43.69           H
ATOM   2049  HG3 PRO A 181       6.819  13.206 -24.315  1.00 43.69           H
ATOM   2050  HD2 PRO A 181       5.893  10.712 -24.271  1.00 42.41           H
ATOM   2051  HD3 PRO A 181       4.839  11.974 -24.936  1.00 42.41           H
ATOM   2052  N   ARG A 182       8.391  10.132 -27.389  1.00 40.41           N
ATOM   2053  CA  ARG A 182       9.486   9.408 -28.026  1.00 39.02           C
ATOM   2054  C   ARG A 182       8.993   8.599 -29.221  1.00 39.17           C
ATOM   2055  O   ARG A 182       9.622   8.604 -30.286  1.00 38.52           O
ATOM   2056  CB  ARG A 182      10.172   8.500 -27.007  1.00 46.89           C
ATOM   2057  CG  ARG A 182      11.445   7.845 -27.514  1.00 49.21           C
ATOM   2058  CD  ARG A 182      12.062   6.972 -26.433  1.00 61.23           C
ATOM   2059  NE  ARG A 182      13.430   6.576 -26.753  1.00 70.36           N
ATOM   2060  CZ  ARG A 182      13.754   5.473 -27.418  1.00 68.95           C
ATOM   2061  NH1 ARG A 182      12.805   4.647 -27.839  1.00 62.21           N1+
ATOM   2062  NH2 ARG A 182      15.027   5.194 -27.662  1.00 72.71           N1+
ATOM   2063  H   ARG A 182       8.152   9.891 -26.438  1.00 40.41           H
ATOM   2064  HA  ARG A 182      10.217  10.136 -28.385  1.00 39.02           H
ATOM   2065  HB2 ARG A 182      10.422   9.101 -26.131  1.00 46.89           H
ATOM   2066  HB3 ARG A 182       9.475   7.736 -26.656  1.00 46.89           H
ATOM   2067  HG2 ARG A 182      11.207   7.208 -28.366  1.00 49.21           H
ATOM   2068  HG3 ARG A 182      12.161   8.586 -27.872  1.00 49.21           H
ATOM   2069  HD2 ARG A 182      12.054   7.503 -25.481  1.00 61.23           H
ATOM   2070  HD3 ARG A 182      11.458   6.081 -26.264  1.00 61.23           H
ATOM   2071  HE  ARG A 182      14.161   7.217 -26.479  1.00 70.36           H
ATOM   2072 HH11 ARG A 182      11.834   4.851 -27.651  1.00 62.21           H
ATOM   2073 HH12 ARG A 182      13.043   3.805 -28.343  1.00 62.21           H
ATOM   2074 HH21 ARG A 182      15.758   5.812 -27.340  1.00 72.71           H
ATOM   2075 HH22 ARG A 182      15.280   4.360 -28.172  1.00 72.71           H
```

ATOM	2076	N	ASN	A	183	7.871	7.894	-29.064	1.00	36.92	N
ATOM	2077	CA	ASN	A	183	7.355	7.091	-30.166	1.00	41.23	C
ATOM	2078	C	ASN	A	183	6.777	7.955	-31.278	1.00	40.20	C
ATOM	2079	O	ASN	A	183	6.824	7.563	-32.450	1.00	35.89	O
ATOM	2080	CB	ASN	A	183	6.303	6.108	-29.658	1.00	36.80	C
ATOM	2081	CG	ASN	A	183	6.917	4.848	-29.092	1.00	51.14	C
ATOM	2082	ND2	ASN	A	183	6.236	4.238	-28.133	1.00	45.86	N
ATOM	2083	OD1	ASN	A	183	7.993	4.425	-29.516	1.00	51.81	O
ATOM	2084	H	ASN	A	183	7.362	7.888	-28.189	1.00	36.92	H
ATOM	2085	HA	ASN	A	183	8.211	6.553	-30.576	1.00	41.23	H
ATOM	2086	HB2	ASN	A	183	5.629	6.590	-28.950	1.00	36.80	H
ATOM	2087	HB3	ASN	A	183	5.678	5.765	-30.485	1.00	36.80	H
ATOM	2088	HD21	ASN	A	183	5.347	4.587	-27.802	1.00	45.86	H
ATOM	2089	HD22	ASN	A	183	6.603	3.394	-27.718	1.00	45.86	H
ATOM	2090	N	ALA	A	184	6.229	9.124	-30.939	1.00	40.21	N
ATOM	2091	CA	ALA	A	184	5.719	10.020	-31.971	1.00	38.93	C
ATOM	2092	C	ALA	A	184	6.839	10.493	-32.886	1.00	39.26	C
ATOM	2093	O	ALA	A	184	6.653	10.599	-34.104	1.00	40.27	O
ATOM	2094	CB	ALA	A	184	5.006	11.209	-31.330	1.00	35.93	C
ATOM	2095	H	ALA	A	184	6.167	9.420	-29.974	1.00	40.21	H
ATOM	2096	HA	ALA	A	184	5.037	9.465	-32.596	1.00	38.93	H
ATOM	2097	HB1	ALA	A	184	4.601	11.888	-32.082	1.00	35.93	H
ATOM	2098	HB2	ALA	A	184	4.174	10.856	-30.728	1.00	35.93	H
ATOM	2099	HB3	ALA	A	184	5.669	11.784	-30.683	1.00	35.93	H
ATOM	2100	N	LYS	A	185	8.014	10.778	-32.319	1.00	36.41	N
ATOM	2101	CA	LYS	A	185	9.151	11.164	-33.147	1.00	38.91	C
ATOM	2102	C	LYS	A	185	9.661	9.986	-33.966	1.00	44.38	C
ATOM	2103	O	LYS	A	185	10.055	10.155	-35.126	1.00	39.25	O
ATOM	2104	CB	LYS	A	185	10.265	11.745	-32.276	1.00	38.84	C
ATOM	2105	CG	LYS	A	185	9.939	13.122	-31.710	1.00	51.01	C
ATOM	2106	CD	LYS	A	185	10.919	13.538	-30.628	1.00	62.35	C
ATOM	2107	CE	LYS	A	185	10.579	14.918	-30.086	1.00	78.25	C
ATOM	2108	NZ	LYS	A	185	11.375	15.266	-28.875	1.00	87.36	N1+
ATOM	2109	H	LYS	A	185	8.152	10.700	-31.320	1.00	36.41	H
ATOM	2110	HA	LYS	A	185	8.834	11.943	-33.845	1.00	38.91	H
ATOM	2111	HB2	LYS	A	185	10.488	11.048	-31.466	1.00	38.84	H
ATOM	2112	HB3	LYS	A	185	11.185	11.834	-32.857	1.00	38.84	H
ATOM	2113	HG2	LYS	A	185	9.908	13.862	-32.511	1.00	51.01	H
ATOM	2114	HG3	LYS	A	185	8.945	13.059	-31.298	1.00	51.01	H
ATOM	2115	HD2	LYS	A	185	10.893	12.808	-29.817	1.00	62.35	H

ATOM 2116 HD3 LYS A 185 11.936 13.531 -31.022 1.00 62.35 H
ATOM 2117 HE2 LYS A 185 10.741 15.677 -30.852 1.00 78.25 H
ATOM 2118 HE3 LYS A 185 9.539 14.978 -29.800 1.00 78.25 H
ATOM 2119 HZ1 LYS A 185 12.359 15.262 -29.104 1.00 87.36 H
ATOM 2120 HZ2 LYS A 185 11.110 16.185 -28.551 1.00 87.36 H
ATOM 2121 HZ3 LYS A 185 11.197 14.590 -28.147 1.00 87.36 H
ATOM 2122 N ILE A 186 9.658 8.784 -33.387 1.00 38.40 N
ATOM 2123 CA ILE A 186 10.059 7.603 -34.144 1.00 41.58 C
ATOM 2124 C ILE A 186 9.111 7.373 -35.317 1.00 41.00 C
ATOM 2125 O ILE A 186 9.546 7.066 -36.434 1.00 38.58 O
ATOM 2126 CB ILE A 186 10.132 6.373 -33.220 1.00 40.47 C
ATOM 2127 CG1 ILE A 186 11.267 6.539 -32.207 1.00 44.84 C
ATOM 2128 CG2 ILE A 186 10.323 5.100 -34.032 1.00 36.96 C
ATOM 2129 CD1 ILE A 186 11.359 5.416 -31.191 1.00 41.00 C
ATOM 2130 H ILE A 186 9.351 8.657 -32.432 1.00 38.40 H
ATOM 2131 HA ILE A 186 11.055 7.781 -34.556 1.00 41.58 H
ATOM 2132 HB ILE A 186 9.195 6.284 -32.671 1.00 40.47 H
ATOM 2133 HG12 ILE A 186 12.212 6.577 -32.751 1.00 44.84 H
ATOM 2134 HG13 ILE A 186 11.216 7.492 -31.691 1.00 44.84 H
ATOM 2135 HG21 ILE A 186 10.468 4.231 -33.391 1.00 36.96 H
ATOM 2136 HG22 ILE A 186 9.457 4.853 -34.646 1.00 36.96 H
ATOM 2137 HG23 ILE A 186 11.193 5.168 -34.686 1.00 36.96 H
ATOM 2138 HD11 ILE A 186 11.823 5.772 -30.272 1.00 41.00 H
ATOM 2139 HD12 ILE A 186 10.375 5.025 -30.930 1.00 41.00 H
ATOM 2140 HD13 ILE A 186 11.965 4.589 -31.563 1.00 41.00 H
ATOM 2141 N ILE A 187 7.805 7.531 -35.087 1.00 36.61 N
ATOM 2142 CA ILE A 187 6.828 7.309 -36.150 1.00 39.38 C
ATOM 2143 C ILE A 187 7.016 8.323 -37.273 1.00 42.51 C
ATOM 2144 O ILE A 187 6.954 7.973 -38.459 1.00 35.92 O
ATOM 2145 CB ILE A 187 5.399 7.346 -35.578 1.00 0.00 C
ATOM 2146 CG1 ILE A 187 4.634 6.046 -35.899 1.00 0.00 C
ATOM 2147 CG2 ILE A 187 4.634 8.585 -36.078 1.00 0.00 C
ATOM 2148 CD1 ILE A 187 3.187 5.912 -35.398 1.00 0.00 C
ATOM 2149 H ILE A 187 7.456 7.762 -34.164 1.00 36.61 H
ATOM 2150 HA ILE A 187 7.020 6.320 -36.572 1.00 39.38 H
ATOM 2151 HB ILE A 187 5.440 7.429 -34.494 1.00 0.00 H
ATOM 2152 HG12 ILE A 187 4.624 5.913 -36.982 1.00 0.00 H
ATOM 2153 HG13 ILE A 187 5.204 5.201 -35.511 1.00 0.00 H
ATOM 2154 HG21 ILE A 187 3.745 8.568 -35.480 1.00 0.00 H
ATOM 2155 HG22 ILE A 187 5.155 9.512 -35.926 1.00 0.00 H

ATOM	2156	HG23	ILE	A	187	4.328	8.511	-37.123	1.00	0.00	H
ATOM	2157	HD11	ILE	A	187	2.946	4.869	-35.206	1.00	0.00	H
ATOM	2158	HD12	ILE	A	187	3.014	6.491	-34.493	1.00	0.00	H
ATOM	2159	HD13	ILE	A	187	2.504	6.244	-36.174	1.00	0.00	H
ATOM	2160	N	ASN	A	188	7.253	9.591	-36.921	1.00	33.47	N
ATOM	2161	CA	ASN	A	188	7.520	10.608	-37.934	1.00	38.81	C
ATOM	2162	C	ASN	A	188	8.732	10.239	-38.784	1.00	38.65	C
ATOM	2163	O	ASN	A	188	8.740	10.471	-39.999	1.00	39.18	O
ATOM	2164	CB	ASN	A	188	7.723	11.973	-37.271	1.00	32.78	C
ATOM	2165	CG	ASN	A	188	6.423	12.582	-36.770	1.00	33.24	C
ATOM	2166	ND2	ASN	A	188	6.534	13.564	-35.881	1.00	37.05	N
ATOM	2167	OD1	ASN	A	188	5.334	12.185	-37.185	1.00	37.65	O
ATOM	2168	H	ASN	A	188	7.275	9.870	-35.949	1.00	33.47	H
ATOM	2169	HA	ASN	A	188	6.644	10.630	-38.586	1.00	38.81	H
ATOM	2170	HB2	ASN	A	188	8.454	11.912	-36.464	1.00	32.78	H
ATOM	2171	HB3	ASN	A	188	8.122	12.681	-38.000	1.00	32.78	H
ATOM	2172	HD21	ASN	A	188	7.443	13.873	-35.570	1.00	37.05	H
ATOM	2173	HD22	ASN	A	188	5.706	14.017	-35.520	1.00	37.05	H
ATOM	2174	N	VAL	A	189	9.762	9.655	-38.167	1.00	36.58	N
ATOM	2175	CA	VAL	A	189	10.945	9.251	-38.924	1.00	37.30	C
ATOM	2176	C	VAL	A	189	10.618	8.080	-39.842	1.00	37.57	C
ATOM	2177	O	VAL	A	189	11.006	8.067	-41.017	1.00	35.01	O
ATOM	2178	CB	VAL	A	189	12.106	8.912	-37.970	1.00	43.19	C
ATOM	2179	CG1	VAL	A	189	13.262	8.275	-38.735	1.00	39.82	C
ATOM	2180	CG2	VAL	A	189	12.579	10.160	-37.239	1.00	39.82	C
ATOM	2181	H	VAL	A	189	9.750	9.477	-37.171	1.00	36.58	H
ATOM	2182	HA	VAL	A	189	11.261	10.087	-39.553	1.00	37.30	H
ATOM	2183	HB	VAL	A	189	11.763	8.202	-37.217	1.00	43.19	H
ATOM	2184	HG11	VAL	A	189	14.147	8.201	-38.102	1.00	39.82	H
ATOM	2185	HG12	VAL	A	189	13.039	7.258	-39.059	1.00	39.82	H
ATOM	2186	HG13	VAL	A	189	13.540	8.861	-39.612	1.00	39.82	H
ATOM	2187	HG21	VAL	A	189	13.289	9.906	-36.451	1.00	39.82	H
ATOM	2188	HG22	VAL	A	189	13.081	10.845	-37.924	1.00	39.82	H
ATOM	2189	HG23	VAL	A	189	11.768	10.719	-36.781	1.00	39.82	H
ATOM	2190	N	CYS	A	190	9.907	7.075	-39.322	1.00	34.33	N
ATOM	2191	CA	CYS	A	190	9.513	5.938	-40.148	1.00	34.82	C
ATOM	2192	C	CYS	A	190	8.655	6.382	-41.327	1.00	40.20	C
ATOM	2193	O	CYS	A	190	8.840	5.906	-42.453	1.00	37.01	O
ATOM	2194	CB	CYS	A	190	8.768	4.907	-39.301	1.00	35.54	C
ATOM	2195	SG	CYS	A	190	9.794	4.069	-38.061	1.00	41.55	S

ATOM 2196 H CYS A 190 9.608 7.089 -38.355 1.00 34.33 H
ATOM 2197 HA CYS A 190 10.415 5.469 -40.547 1.00 34.82 H
ATOM 2198 HB2 CYS A 190 7.922 5.368 -38.790 1.00 35.54 H
ATOM 2199 HB3 CYS A 190 8.351 4.129 -39.943 1.00 35.54 H
ATOM 2200 HG CYS A 190 9.969 5.141 -37.281 1.00 41.55 H
ATOM 2201 N ASN A 191 7.707 7.291 -41.085 1.00 32.76 N
ATOM 2202 CA ASN A 191 6.882 7.808 -42.171 1.00 34.97 C
ATOM 2203 C ASN A 191 7.715 8.577 -43.186 1.00 38.08 C
ATOM 2204 O ASN A 191 7.430 8.528 -44.389 1.00 37.26 O
ATOM 2205 CB ASN A 191 5.774 8.701 -41.613 1.00 31.04 C
ATOM 2206 CG ASN A 191 4.679 7.908 -40.934 1.00 38.93 C
ATOM 2207 ND2 ASN A 191 3.895 8.577 -40.097 1.00 35.56 N
ATOM 2208 OD1 ASN A 191 4.532 6.709 -41.168 1.00 42.78 O
ATOM 2209 H ASN A 191 7.544 7.649 -40.151 1.00 32.76 H
ATOM 2210 HA ASN A 191 6.448 6.945 -42.682 1.00 34.97 H
ATOM 2211 HB2 ASN A 191 6.183 9.454 -40.936 1.00 31.04 H
ATOM 2212 HB3 ASN A 191 5.290 9.244 -42.427 1.00 31.04 H
ATOM 2213 HD21 ASN A 191 4.048 9.560 -39.925 1.00 35.56 H
ATOM 2214 HD22 ASN A 191 3.143 8.105 -39.611 1.00 35.56 H
ATOM 2215 N TRP A 192 8.742 9.291 -42.728 1.00 33.83 N
ATOM 2216 CA TRP A 192 9.609 9.999 -43.660 1.00 36.79 C
ATOM 2217 C TRP A 192 10.428 9.023 -44.495 1.00 44.90 C
ATOM 2218 O TRP A 192 10.631 9.242 -45.696 1.00 38.01 O
ATOM 2219 CB TRP A 192 10.519 10.962 -42.901 1.00 35.05 C
ATOM 2220 CG TRP A 192 11.408 11.747 -43.808 1.00 45.30 C
ATOM 2221 CD1 TRP A 192 11.106 12.918 -44.441 1.00 48.31 C
ATOM 2222 CD2 TRP A 192 12.744 11.413 -44.196 1.00 46.40 C
ATOM 2223 CE2 TRP A 192 13.194 12.430 -45.061 1.00 50.31 C
ATOM 2224 CE3 TRP A 192 13.606 10.353 -43.894 1.00 50.00 C
ATOM 2225 NE1 TRP A 192 12.175 13.336 -45.195 1.00 50.57 N
ATOM 2226 CZ2 TRP A 192 14.466 12.419 -45.629 1.00 50.26 C
ATOM 2227 CZ3 TRP A 192 14.868 10.344 -44.458 1.00 58.63 C
ATOM 2228 CH2 TRP A 192 15.286 11.371 -45.314 1.00 56.32 C
ATOM 2229 H TRP A 192 8.953 9.348 -41.740 1.00 33.83 H
ATOM 2230 HA TRP A 192 8.990 10.593 -44.336 1.00 36.79 H
ATOM 2231 HB2 TRP A 192 9.916 11.665 -42.325 1.00 35.05 H
ATOM 2232 HB3 TRP A 192 11.138 10.430 -42.178 1.00 35.05 H
ATOM 2233 HD1 TRP A 192 10.157 13.428 -44.354 1.00 48.31 H
ATOM 2234 HE1 TRP A 192 12.180 14.178 -45.753 1.00 50.57 H
ATOM 2235 HE3 TRP A 192 13.293 9.558 -43.234 1.00 50.00 H

ATOM 2236 HZ2 TRP A 192 14.791 13.205 -46.295 1.00 50.26 H
ATOM 2237 HZ3 TRP A 192 15.543 9.530 -44.235 1.00 58.63 H
ATOM 2238 HH2 TRP A 192 16.278 11.332 -45.740 1.00 56.32 H
ATOM 2239 N ILE A 193 10.906 7.939 -43.878 1.00 35.98 N
ATOM 2240 CA ILE A 193 11.613 6.905 -44.628 1.00 37.96 C
ATOM 2241 C ILE A 193 10.703 6.311 -45.693 1.00 42.04 C
ATOM 2242 O ILE A 193 11.120 6.086 -46.837 1.00 40.87 O
ATOM 2243 CB ILE A 193 12.148 5.823 -43.672 1.00 37.67 C
ATOM 2244 CG1 ILE A 193 13.199 6.417 -42.731 1.00 38.83 C
ATOM 2245 CG2 ILE A 193 12.716 4.644 -44.456 1.00 38.06 C
ATOM 2246 CD1 ILE A 193 13.717 5.443 -41.690 1.00 45.19 C
ATOM 2247 H ILE A 193 10.758 7.790 -42.887 1.00 35.98 H
ATOM 2248 HA ILE A 193 12.462 7.371 -45.135 1.00 37.96 H
ATOM 2249 HB ILE A 193 11.327 5.451 -43.059 1.00 37.67 H
ATOM 2250 HG12 ILE A 193 14.049 6.741 -43.333 1.00 38.83 H
ATOM 2251 HG13 ILE A 193 12.866 7.323 -42.242 1.00 38.83 H
ATOM 2252 HG21 ILE A 193 13.172 3.906 -43.798 1.00 38.06 H
ATOM 2253 HG22 ILE A 193 11.955 4.095 -45.011 1.00 38.06 H
ATOM 2254 HG23 ILE A 193 13.483 4.966 -45.162 1.00 38.06 H
ATOM 2255 HD11 ILE A 193 14.122 5.978 -40.831 1.00 45.19 H
ATOM 2256 HD12 ILE A 193 12.927 4.787 -41.324 1.00 45.19 H
ATOM 2257 HD13 ILE A 193 14.518 4.820 -42.089 1.00 45.19 H
ATOM 2258 N LEU A 194 9.442 6.055 -45.336 1.00 38.37 N
ATOM 2259 CA LEU A 194 8.486 5.529 -46.303 1.00 45.61 C
ATOM 2260 C LEU A 194 8.228 6.532 -47.421 1.00 47.31 C
ATOM 2261 O LEU A 194 8.177 6.159 -48.599 1.00 47.61 O
ATOM 2262 CB LEU A 194 7.183 5.158 -45.595 1.00 38.86 C
ATOM 2263 CG LEU A 194 6.083 4.503 -46.429 1.00 46.77 C
ATOM 2264 CD1 LEU A 194 6.568 3.183 -47.014 1.00 43.05 C
ATOM 2265 CD2 LEU A 194 4.830 4.297 -45.588 1.00 48.90 C
ATOM 2266 H LEU A 194 9.121 6.218 -44.390 1.00 38.37 H
ATOM 2267 HA LEU A 194 8.914 4.627 -46.744 1.00 45.61 H
ATOM 2268 HB2 LEU A 194 7.421 4.498 -44.758 1.00 38.86 H
ATOM 2269 HB3 LEU A 194 6.774 6.059 -45.136 1.00 38.86 H
ATOM 2270 HG LEU A 194 5.811 5.163 -47.253 1.00 46.77 H
ATOM 2271 HD11 LEU A 194 5.773 2.441 -47.091 1.00 43.05 H
ATOM 2272 HD12 LEU A 194 6.951 3.334 -48.024 1.00 43.05 H
ATOM 2273 HD13 LEU A 194 7.370 2.738 -46.424 1.00 43.05 H
ATOM 2274 HD21 LEU A 194 3.947 4.661 -46.114 1.00 48.90 H
ATOM 2275 HD22 LEU A 194 4.650 3.249 -45.346 1.00 48.90 H

```
ATOM   2276 HD23 LEU A 194       4.874   4.832 -44.638  1.00 48.90           H
ATOM   2277  N   SER A 195       8.069   7.811 -47.070  1.00 40.15           N
ATOM   2278  CA  SER A 195       7.892   8.842 -48.087  1.00 43.64           C
ATOM   2279  C   SER A 195       9.107   8.942 -49.002  1.00 43.33           C
ATOM   2280  O   SER A 195       8.975   9.329 -50.168  1.00 41.40           O
ATOM   2281  CB  SER A 195       7.614  10.191 -47.422  1.00 36.06           C
ATOM   2282  OG  SER A 195       6.424  10.147 -46.658  1.00 44.98           O
ATOM   2283  H   SER A 195       8.092   8.097 -46.099  1.00 40.15           H
ATOM   2284  HA  SER A 195       7.029   8.580 -48.703  1.00 43.64           H
ATOM   2285  HB2 SER A 195       8.439  10.490 -46.774  1.00 36.06           H
ATOM   2286  HB3 SER A 195       7.503  10.975 -48.173  1.00 36.06           H
ATOM   2287  HG  SER A 195       6.589   9.622 -45.869  1.00 44.98           H
ATOM   2288  N   SER A 196      10.292   8.592 -48.496  1.00 38.09           N
ATOM   2289  CA  SER A 196      11.506   8.664 -49.300  1.00 41.59           C
ATOM   2290  C   SER A 196      11.518   7.650 -50.438  1.00 42.19           C
ATOM   2291  O   SER A 196      12.251   7.847 -51.413  1.00 46.33           O
ATOM   2292  CB  SER A 196      12.734   8.461 -48.412  1.00 43.43           C
ATOM   2293  OG  SER A 196      12.835   9.486 -47.439  1.00 45.74           O
ATOM   2294  H   SER A 196      10.378   8.280 -47.538  1.00 38.09           H
ATOM   2295  HA  SER A 196      11.565   9.653 -49.757  1.00 41.59           H
ATOM   2296  HB2 SER A 196      12.746   7.491 -47.925  1.00 43.43           H
ATOM   2297  HB3 SER A 196      13.638   8.497 -49.017  1.00 43.43           H
ATOM   2298  HG  SER A 196      12.127   9.370 -46.794  1.00 45.74           H
ATOM   2299  N   ALA A 197      10.731   6.572 -50.337  1.00 42.37           N
ATOM   2300  CA  ALA A 197      10.681   5.576 -51.404  1.00 47.80           C
ATOM   2301  C   ALA A 197      10.208   6.171 -52.722  1.00 49.56           C
ATOM   2302  O   ALA A 197      10.492   5.609 -53.784  1.00 50.31           O
ATOM   2303  CB  ALA A 197       9.772   4.412 -51.005  1.00 47.45           C
ATOM   2304  H   ALA A 197      10.135   6.430 -49.532  1.00 42.37           H
ATOM   2305  HA  ALA A 197      11.690   5.185 -51.553  1.00 47.80           H
ATOM   2306  HB1 ALA A 197       9.783   3.623 -51.758  1.00 47.45           H
ATOM   2307  HB2 ALA A 197      10.096   3.968 -50.063  1.00 47.45           H
ATOM   2308  HB3 ALA A 197       8.738   4.735 -50.879  1.00 47.45           H
ATOM   2309  N   ILE A 198       9.494   7.292 -52.676  1.00 42.68           N
ATOM   2310  CA  ILE A 198       9.100   8.026 -53.865  1.00 48.64           C
ATOM   2311  C   ILE A 198       9.753   9.403 -53.914  1.00 52.39           C
ATOM   2312  O   ILE A 198      10.140   9.870 -54.987  1.00 47.98           O
ATOM   2313  CB  ILE A 198       7.543   8.146 -53.995  1.00 50.56           C
ATOM   2314  CG1 ILE A 198       6.925   6.747 -54.254  1.00 54.38           C
ATOM   2315  CG2 ILE A 198       7.056   9.135 -55.086  1.00 48.54           C
```

ATOM   2316 CD1 ILE A 198   5.409   6.654 -54.023 1.00 56.06   C
ATOM   2317 H   ILE A 198   9.264   7.706 -51.784 1.00 42.68   H
ATOM   2318 HA ILE A 198   9.437   7.522 -54.773 1.00 48.64   H
ATOM   2319 HB ILE A 198   7.157   8.504 -53.038 1.00 50.56   H
ATOM   2320 HG12 ILE A 198   7.156   6.429 -55.271 1.00 54.38   H
ATOM   2321 HG13 ILE A 198   7.391   6.004 -53.607 1.00 54.38   H
ATOM   2322 HG21 ILE A 198   5.973   9.121 -55.202 1.00 48.54   H
ATOM   2323 HG22 ILE A 198   7.322 10.168 -54.861 1.00 48.54   H
ATOM   2324 HG23 ILE A 198   7.484   8.891 -56.058 1.00 48.54   H
ATOM   2325 HD11 ILE A 198   5.076   5.617 -54.065 1.00 56.06   H
ATOM   2326 HD12 ILE A 198   5.139   7.039 -53.040 1.00 56.06   H
ATOM   2327 HD13 ILE A 198   4.844   7.203 -54.776 1.00 56.06   H
ATOM   2328 N   GLY A 199   9.923 10.051 -52.755 1.00 44.98   N
ATOM   2329 CA GLY A 199   10.474 11.397 -52.744 1.00 43.62   C
ATOM   2330 C   GLY A 199   11.904 11.476 -53.244 1.00 47.58   C
ATOM   2331 O   GLY A 199   12.292 12.467 -53.866 1.00 53.22   O
ATOM   2332 H   GLY A 199   9.606   9.657 -51.878 1.00 44.98   H
ATOM   2333 HA2 GLY A 199   9.836 12.068 -53.323 1.00 43.62   H
ATOM   2334 HA3 GLY A 199   10.440 11.750 -51.716 1.00 43.62   H
ATOM   2335 N   LEU A 200   12.712 10.452 -52.968 1.00 46.67   N
ATOM   2336 CA LEU A 200   14.099 10.437 -53.423 1.00 52.06   C
ATOM   2337 C   LEU A 200   14.217 10.048 -54.896 1.00 51.35   C
ATOM   2338 O   LEU A 200   14.953 10.714 -55.637 1.00 42.98   O
ATOM   2339 CB LEU A 200   14.943   9.496 -52.557 1.00 57.90   C
ATOM   2340 CG LEU A 200   15.347 10.002 -51.175 1.00 62.45   C
ATOM   2341 CD1 LEU A 200   16.181   8.959 -50.451 1.00 66.58   C
ATOM   2342 CD2 LEU A 200   16.107 11.312 -51.293 1.00 66.77   C
ATOM   2343 H   LEU A 200   12.381   9.647 -52.452 1.00 46.67   H
ATOM   2344 HA LEU A 200   14.511 11.444 -53.343 1.00 52.06   H
ATOM   2345 HB2 LEU A 200   14.454   8.544 -52.405 1.00 57.90   H
ATOM   2346 HB3 LEU A 200   15.865   9.258 -53.092 1.00 57.90   H
ATOM   2347 HG LEU A 200   14.426 10.135 -50.626 1.00 62.45   H
ATOM   2348 HD11 LEU A 200   15.891   8.892 -49.403 1.00 66.58   H
ATOM   2349 HD12 LEU A 200   16.066   7.962 -50.879 1.00 66.58   H
ATOM   2350 HD13 LEU A 200   17.247   9.187 -50.474 1.00 66.58   H
ATOM   2351 HD21 LEU A 200   16.880 11.412 -50.531 1.00 66.77   H
ATOM   2352 HD22 LEU A 200   16.595 11.429 -52.261 1.00 66.77   H
ATOM   2353 HD23 LEU A 200   15.432 12.158 -51.160 1.00 66.77   H
ATOM   2354 N   PRO A 201   13.545   8.983 -55.367 1.00 42.72   N
ATOM   2355 CA PRO A 201   13.636   8.668 -56.805 1.00 45.63   C

ATOM 2356 C PRO A 201 13.171 9.792 -57.717 1.00 49.30 C
ATOM 2357 O PRO A 201 13.757 9.980 -58.791 1.00 53.76 O
ATOM 2358 CB PRO A 201 12.751 7.423 -56.945 1.00 43.87 C
ATOM 2359 CG PRO A 201 12.821 6.777 -55.620 1.00 46.80 C
ATOM 2360 CD PRO A 201 12.840 7.910 -54.636 1.00 42.90 C
ATOM 2361 HA PRO A 201 14.671 8.407 -57.036 1.00 45.63 H
ATOM 2362 HB2 PRO A 201 11.715 7.698 -57.155 1.00 43.87 H
ATOM 2363 HB3 PRO A 201 13.083 6.762 -57.746 1.00 43.87 H
ATOM 2364 HG2 PRO A 201 12.030 6.049 -55.491 1.00 46.80 H
ATOM 2365 HG3 PRO A 201 13.764 6.233 -55.548 1.00 46.80 H
ATOM 2366 HD2 PRO A 201 11.844 8.186 -54.372 1.00 42.90 H
ATOM 2367 HD3 PRO A 201 13.337 7.552 -53.745 1.00 42.90 H
ATOM 2368 N VAL A 202 12.136 10.547 -57.334 1.00 41.26 N
ATOM 2369 CA VAL A 202 11.673 11.613 -58.220 1.00 46.30 C
ATOM 2370 C VAL A 202 12.703 12.729 -58.304 1.00 50.30 C
ATOM 2371 O VAL A 202 12.744 13.466 -59.294 1.00 51.45 O
ATOM 2372 CB VAL A 202 10.297 12.157 -57.787 1.00 40.72 C
ATOM 2373 CG1 VAL A 202 9.244 11.057 -57.841 1.00 36.87 C
ATOM 2374 CG2 VAL A 202 10.368 12.796 -56.410 1.00 44.68 C
ATOM 2375 H VAL A 202 11.635 10.385 -56.470 1.00 41.26 H
ATOM 2376 HA VAL A 202 11.540 11.201 -59.217 1.00 46.30 H
ATOM 2377 HB VAL A 202 9.988 12.935 -58.486 1.00 40.72 H
ATOM 2378 HG11 VAL A 202 8.300 11.391 -57.410 1.00 36.87 H
ATOM 2379 HG12 VAL A 202 9.039 10.780 -58.875 1.00 36.87 H
ATOM 2380 HG13 VAL A 202 9.538 10.144 -57.331 1.00 36.87 H
ATOM 2381 HG21 VAL A 202 9.386 13.147 -56.097 1.00 44.68 H
ATOM 2382 HG22 VAL A 202 10.710 12.094 -55.669 1.00 44.68 H
ATOM 2383 HG23 VAL A 202 11.030 13.660 -56.389 1.00 44.68 H
ATOM 2384 N MET A 203 13.552 12.875 -57.284 1.00 50.01 N
ATOM 2385 CA MET A 203 14.636 13.849 -57.369 1.00 56.63 C
ATOM 2386 C MET A 203 15.615 13.485 -58.477 1.00 53.71 C
ATOM 2387 O MET A 203 16.162 14.369 -59.148 1.00 52.82 O
ATOM 2388 CB MET A 203 15.351 13.956 -56.024 1.00 64.51 C
ATOM 2389 CG MET A 203 16.660 14.719 -56.066 1.00 79.41 C
ATOM 2390 SD MET A 203 16.906 15.735 -54.602 1.00 93.94 S
ATOM 2391 CE MET A 203 15.716 17.039 -54.903 1.00 88.64 C
ATOM 2392 H MET A 203 13.518 12.268 -56.476 1.00 50.01 H
ATOM 2393 HA MET A 203 14.216 14.830 -57.602 1.00 56.63 H
ATOM 2394 HB2 MET A 203 14.668 14.372 -55.285 1.00 64.51 H
ATOM 2395 HB3 MET A 203 15.598 12.971 -55.647 1.00 64.51 H

ATOM 2396 HG2 MET A 203 17.502 14.034 -56.175 1.00 79.41 H
ATOM 2397 HG3 MET A 203 16.703 15.416 -56.902 1.00 79.41 H
ATOM 2398 HE1 MET A 203 15.677 17.722 -54.055 1.00 88.64 H
ATOM 2399 HE2 MET A 203 15.995 17.609 -55.789 1.00 88.64 H
ATOM 2400 HE3 MET A 203 14.727 16.631 -55.065 1.00 88.64 H
ATOM 2401 N PHE A 204 15.836 12.187 -58.698 1.00 50.90 N
ATOM 2402 CA PHE A 204 16.662 11.753 -59.819 1.00 49.90 C
ATOM 2403 C PHE A 204 15.880 11.759 -61.125 1.00 49.62 C
ATOM 2404 O PHE A 204 16.419 12.136 -62.172 1.00 52.96 O
ATOM 2405 CB PHE A 204 17.229 10.359 -59.547 1.00 58.38 C
ATOM 2406 CG PHE A 204 18.332 10.342 -58.530 1.00 61.45 C
ATOM 2407 CD1 PHE A 204 19.652 10.472 -58.924 1.00 66.61 C
ATOM 2408 CD2 PHE A 204 18.051 10.197 -57.181 1.00 63.92 C
ATOM 2409 CE1 PHE A 204 20.673 10.460 -57.994 1.00 72.05 C
ATOM 2410 CE2 PHE A 204 19.069 10.183 -56.244 1.00 64.58 C
ATOM 2411 CZ PHE A 204 20.382 10.315 -56.653 1.00 66.27 C
ATOM 2412 H PHE A 204 15.378 11.476 -58.144 1.00 50.90 H
ATOM 2413 HA PHE A 204 17.512 12.426 -59.948 1.00 49.90 H
ATOM 2414 HB2 PHE A 204 16.437 9.684 -59.218 1.00 58.38 H
ATOM 2415 HB3 PHE A 204 17.618 9.923 -60.469 1.00 58.38 H
ATOM 2416 HD1 PHE A 204 19.893 10.588 -59.971 1.00 66.61 H
ATOM 2417 HD2 PHE A 204 17.030 10.084 -56.856 1.00 63.92 H
ATOM 2418 HE1 PHE A 204 21.699 10.563 -58.316 1.00 72.05 H
ATOM 2419 HE2 PHE A 204 18.836 10.068 -55.195 1.00 64.58 H
ATOM 2420 HZ PHE A 204 21.179 10.305 -55.924 1.00 66.27 H
ATOM 2421 N MET A 205 14.609 11.351 -61.084 1.00 41.16 N
ATOM 2422 CA MET A 205 13.825 11.236 -62.310 1.00 48.71 C
ATOM 2423 C MET A 205 13.426 12.595 -62.871 1.00 49.31 C
ATOM 2424 O MET A 205 13.302 12.745 -64.092 1.00 50.17 O
ATOM 2425 CB MET A 205 12.550 10.415 -62.048 1.00 44.99 C
ATOM 2426 CG MET A 205 12.786 8.942 -61.679 1.00 58.86 C
ATOM 2427 SD MET A 205 11.496 8.212 -60.629 1.00 63.02 S
ATOM 2428 CE MET A 205 10.012 8.747 -61.515 1.00 68.95 C
ATOM 2429 H MET A 205 14.189 11.026 -60.221 1.00 41.16 H
ATOM 2430 HA MET A 205 14.417 10.726 -63.074 1.00 48.71 H
ATOM 2431 HB2 MET A 205 11.953 10.907 -61.285 1.00 44.99 H
ATOM 2432 HB3 MET A 205 11.933 10.434 -62.948 1.00 44.99 H
ATOM 2433 HG2 MET A 205 12.867 8.346 -62.589 1.00 58.86 H
ATOM 2434 HG3 MET A 205 13.735 8.816 -61.160 1.00 58.86 H
ATOM 2435 HE1 MET A 205 9.157 8.168 -61.174 1.00 68.95 H

ATOM	2436	HE2	MET	A	205	9.805	9.802	-61.335	1.00	68.95	H
ATOM	2437	HE3	MET	A	205	10.139	8.596	-62.587	1.00	68.95	H
ATOM	2438	N	ALA	A	206	13.203	13.583	-62.009	1.00	39.97	N
ATOM	2439	CA	ALA	A	206	12.709	14.871	-62.474	1.00	46.95	C
ATOM	2440	C	ALA	A	206	13.765	15.586	-63.303	1.00	51.63	C
ATOM	2441	O	ALA	A	206	14.952	15.582	-62.969	1.00	55.63	O
ATOM	2442	CB	ALA	A	206	12.291	15.750	-61.296	1.00	37.78	C
ATOM	2443	H	ALA	A	206	13.282	13.444	-61.009	1.00	39.97	H
ATOM	2444	HA	ALA	A	206	11.823	14.674	-63.063	1.00	46.95	H
ATOM	2445	HB1	ALA	A	206	12.035	16.762	-61.612	1.00	37.78	H
ATOM	2446	HB2	ALA	A	206	11.426	15.334	-60.784	1.00	37.78	H
ATOM	2447	HB3	ALA	A	206	13.095	15.821	-60.565	1.00	37.78	H
ATOM	2448	N	THR	A	207	13.322	16.197	-64.396	1.00	53.48	N
ATOM	2449	CA	THR	A	207	14.208	16.977	-65.245	1.00	54.00	C
ATOM	2450	C	THR	A	207	13.359	17.872	-66.130	1.00	52.23	C
ATOM	2451	O	THR	A	207	12.152	17.666	-66.279	1.00	55.37	O
ATOM	2452	CB	THR	A	207	15.115	16.081	-66.099	1.00	60.06	C
ATOM	2453	CG2	THR	A	207	14.308	15.373	-67.175	1.00	51.07	C
ATOM	2454	OG1	THR	A	207	16.126	16.880	-66.724	1.00	69.31	O
ATOM	2455	H	THR	A	207	12.342	16.168	-64.653	1.00	53.48	H
ATOM	2456	HA	THR	A	207	14.819	17.627	-64.613	1.00	54.00	H
ATOM	2457	HB	THR	A	207	15.618	15.337	-65.481	1.00	60.06	H
ATOM	2458	HG1	THR	A	207	16.588	16.339	-67.370	1.00	69.31	H
ATOM	2459	HG21	THR	A	207	14.918	14.606	-67.653	1.00	51.07	H
ATOM	2460	HG22	THR	A	207	13.438	14.869	-66.754	1.00	51.07	H
ATOM	2461	HG23	THR	A	207	13.966	16.035	-67.972	1.00	51.07	H
ATOM	2462	N	THR	A	208	14.006	18.878	-66.704	1.00	60.05	N
ATOM	2463	CA	THR	A	208	13.395	19.723	-67.718	1.00	61.26	C
ATOM	2464	C	THR	A	208	13.792	19.207	-69.094	1.00	63.88	C
ATOM	2465	O	THR	A	208	14.971	18.932	-69.343	1.00	78.15	O
ATOM	2466	CB	THR	A	208	13.828	21.180	-67.559	1.00	61.63	C
ATOM	2467	CG2	THR	A	208	13.307	21.756	-66.247	1.00	54.71	C
ATOM	2468	OG1	THR	A	208	15.260	21.255	-67.580	1.00	58.74	O
ATOM	2469	H	THR	A	208	14.999	18.996	-66.559	1.00	60.05	H
ATOM	2470	HA	THR	A	208	12.310	19.703	-67.632	1.00	61.26	H
ATOM	2471	HB	THR	A	208	13.447	21.780	-68.386	1.00	61.63	H
ATOM	2472	HG1	THR	A	208	15.572	20.880	-68.409	1.00	58.74	H
ATOM	2473	HG21	THR	A	208	13.634	22.788	-66.120	1.00	54.71	H
ATOM	2474	HG22	THR	A	208	12.217	21.756	-66.227	1.00	54.71	H
ATOM	2475	HG23	THR	A	208	13.646	21.188	-65.383	1.00	54.71	H

ATOM 2476 N LYS A 209 12.807 19.062 -69.975 1.00 61.86 N
ATOM 2477 CA LYS A 209 13.060 18.699 -71.360 1.00 74.00 C
ATOM 2478 C LYS A 209 12.163 19.536 -72.258 1.00 80.79 C
ATOM 2479 O LYS A 209 11.102 20.007 -71.838 1.00 76.57 O
ATOM 2480 CB LYS A 209 12.824 17.204 -71.615 1.00 76.15 C
ATOM 2481 CG LYS A 209 11.414 16.735 -71.312 1.00 78.90 C
ATOM 2482 CD LYS A 209 11.107 15.431 -72.025 1.00 80.46 C
ATOM 2483 CE LYS A 209 11.183 15.610 -73.532 1.00 92.30 C
ATOM 2484 NZ LYS A 209 10.842 14.360 -74.266 1.00 96.81 N1+
ATOM 2485 H LYS A 209 11.851 19.282 -69.727 1.00 61.86 H
ATOM 2486 HA LYS A 209 14.089 18.939 -71.639 1.00 74.00 H
ATOM 2487 HB2 LYS A 209 13.149 17.040 -72.635 1.00 76.15 H
ATOM 2488 HB3 LYS A 209 13.520 16.621 -71.010 1.00 76.15 H
ATOM 2489 HG2 LYS A 209 11.312 16.593 -70.236 1.00 78.90 H
ATOM 2490 HG3 LYS A 209 10.650 17.461 -71.587 1.00 78.90 H
ATOM 2491 HD2 LYS A 209 11.804 14.657 -71.699 1.00 80.46 H
ATOM 2492 HD3 LYS A 209 10.111 15.091 -71.739 1.00 80.46 H
ATOM 2493 HE2 LYS A 209 10.495 16.393 -73.851 1.00 92.30 H
ATOM 2494 HE3 LYS A 209 12.168 15.867 -73.909 1.00 92.30 H
ATOM 2495 HZ1 LYS A 209 9.908 14.068 -74.020 1.00 96.81 H
ATOM 2496 HZ2 LYS A 209 10.893 14.530 -75.260 1.00 96.81 H
ATOM 2497 HZ3 LYS A 209 11.499 13.634 -74.017 1.00 96.81 H
ATOM 2498 N TYR A 210 12.600 19.719 -73.500 1.00 83.61 N
ATOM 2499 CA TYR A 210 11.867 20.557 -74.436 1.00 91.20 C
ATOM 2500 C TYR A 210 10.630 19.841 -74.959 1.00 92.18 C
ATOM 2501 O TYR A 210 10.657 18.639 -75.240 1.00 92.79 O
ATOM 2502 CB TYR A 210 12.765 20.977 -75.598 1.00 96.10 C
ATOM 2503 CG TYR A 210 13.582 22.209 -75.294 1.00106.77 C
ATOM 2504 CD1 TYR A 210 12.993 23.467 -75.287 1.00114.30 C
ATOM 2505 CD2 TYR A 210 14.938 22.118 -75.009 1.00109.50 C
ATOM 2506 CE1 TYR A 210 13.730 24.599 -75.006 1.00120.46 C
ATOM 2507 CE2 TYR A 210 15.685 23.247 -74.727 1.00115.42 C
ATOM 2508 CZ TYR A 210 15.075 24.485 -74.727 1.00122.00 C
ATOM 2509 OH TYR A 210 15.812 25.613 -74.448 1.00128.00 O
ATOM 2510 H TYR A 210 13.470 19.317 -73.815 1.00 83.61 H
ATOM 2511 HA TYR A 210 11.542 21.460 -73.912 1.00 91.20 H
ATOM 2512 HB2 TYR A 210 13.401 20.153 -75.928 1.00 96.10 H
ATOM 2513 HB3 TYR A 210 12.158 21.241 -76.466 1.00 96.10 H
ATOM 2514 HD1 TYR A 210 11.938 23.566 -75.500 1.00114.30 H
ATOM 2515 HD2 TYR A 210 15.423 21.153 -75.004 1.00109.50 H

```
ATOM   2516  HE1 TYR A 210      13.247  25.565 -75.004  1.00120.46           H
ATOM   2517  HE2 TYR A 210      16.739  23.158 -74.508  1.00115.42           H
ATOM   2518  HH  TYR A 210      15.293  26.420 -74.474  1.00128.00           H
ATOM   2519  N   ARG A 211       9.539  20.597 -75.087  1.00 90.65           N
ATOM   2520  CA  ARG A 211       8.251  20.057 -75.525  1.00 95.11           C
ATOM   2521  C   ARG A 211       7.568  21.141 -76.362  1.00104.28           C
ATOM   2522  O   ARG A 211       6.895  22.023 -75.824  1.00109.68           O
ATOM   2523  CB  ARG A 211       7.397  19.636 -74.338  1.00101.43           C
ATOM   2524  CG  ARG A 211       6.046  19.060 -74.718  1.00107.03           C
ATOM   2525  CD  ARG A 211       6.198  17.725 -75.419  1.00113.27           C
ATOM   2526  NE  ARG A 211       4.974  17.335 -76.111  1.00120.37           N
ATOM   2527  CZ  ARG A 211       4.824  16.193 -76.772  1.00120.52           C
ATOM   2528  NH1 ARG A 211       5.821  15.321 -76.826  1.00117.65           N1+
ATOM   2529  NH2 ARG A 211       3.676  15.920 -77.376  1.00122.28           N1+
ATOM   2530  H   ARG A 211       9.549  21.575 -74.817  1.00 90.65           H
ATOM   2531  HA  ARG A 211       8.436  19.213 -76.188  1.00 95.11           H
ATOM   2532  HB2 ARG A 211       7.943  18.899 -73.746  1.00101.43           H
ATOM   2533  HB3 ARG A 211       7.252  20.489 -73.673  1.00101.43           H
ATOM   2534  HG2 ARG A 211       5.528  18.864 -73.779  1.00107.03           H
ATOM   2535  HG3 ARG A 211       5.408  19.753 -75.269  1.00107.03           H
ATOM   2536  HD2 ARG A 211       7.072  17.637 -76.059  1.00113.27           H
ATOM   2537  HD3 ARG A 211       6.312  16.964 -74.646  1.00113.27           H
ATOM   2538  HE  ARG A 211       4.223  18.013 -76.123  1.00120.37           H
ATOM   2539 HH11 ARG A 211       6.704  15.537 -76.388  1.00117.65           H
ATOM   2540 HH12 ARG A 211       5.713  14.452 -77.330  1.00117.65           H
ATOM   2541 HH21 ARG A 211       2.918  16.589 -77.350  1.00122.28           H
ATOM   2542 HH22 ARG A 211       3.561  15.064 -77.903  1.00122.28           H
ATOM   2543  N   GLN A 212       7.759  21.061 -77.682  1.00100.09           N
ATOM   2544  CA  GLN A 212       7.173  22.008 -78.633  1.00100.97           C
ATOM   2545  C   GLN A 212       7.605  23.441 -78.321  1.00106.86           C
ATOM   2546  O   GLN A 212       6.787  24.360 -78.237  1.00103.67           O
ATOM   2547  CB  GLN A 212       5.647  21.891 -78.659  1.00101.41           C
ATOM   2548  CG  GLN A 212       5.132  20.468 -78.789  1.00103.31           C
ATOM   2549  CD  GLN A 212       3.618  20.401 -78.829  1.00105.89           C
ATOM   2550  NE2 GLN A 212       2.979  21.033 -79.669  1.00102.10           N
ATOM   2551  OE1 GLN A 212       3.035  19.636 -77.912  1.00111.06           O
ATOM   2552  H   GLN A 212       8.327  20.322 -78.070  1.00100.09           H
ATOM   2553  HA  GLN A 212       7.560  21.755 -79.621  1.00100.97           H
ATOM   2554  HB2 GLN A 212       5.211  22.329 -77.759  1.00101.41           H
ATOM   2555  HB3 GLN A 212       5.272  22.493 -79.489  1.00101.41           H
```

```
ATOM   2556  HG2 GLN A 212       5.524  20.010 -79.697  1.00103.31           H
ATOM   2557  HG3 GLN A 212       5.472  19.849 -77.959  1.00103.31           H
ATOM   2558 HE21 GLN A 212       3.452  21.610 -80.349  1.00102.10           H
ATOM   2559 HE22 GLN A 212       1.970  20.984 -79.677  1.00102.10           H
ATOM   2560  N   GLY A 213       8.906  23.627 -78.149  1.00112.07           N
ATOM   2561  CA  GLY A 213       9.430  24.938 -77.765  1.00119.50           C
ATOM   2562  C   GLY A 213       9.297  25.261 -76.294  1.00120.52           C
ATOM   2563  O   GLY A 213      10.246  25.756 -75.679  1.00126.75           O
ATOM   2564  H   GLY A 213       9.553  22.856 -78.224  1.00112.07           H
ATOM   2565  HA2 GLY A 213      10.489  24.953 -78.026  1.00119.50           H
ATOM   2566  HA3 GLY A 213       8.969  25.735 -78.351  1.00119.50           H
ATOM   2567  N   SER A 214       8.131  24.992 -75.709  1.00113.95           N
ATOM   2568  CA  SER A 214       7.926  25.199 -74.285  1.00101.89           C
ATOM   2569  C   SER A 214       8.679  24.139 -73.481  1.00 93.27           C
ATOM   2570  O   SER A 214       9.149  23.129 -74.011  1.00 91.08           O
ATOM   2571  CB  SER A 214       6.435  25.169 -73.952  1.00103.78           C
ATOM   2572  OG  SER A 214       5.847  23.950 -74.365  1.00110.89           O
ATOM   2573  H   SER A 214       7.370  24.602 -76.247  1.00113.95           H
ATOM   2574  HA  SER A 214       8.311  26.181 -74.001  1.00101.89           H
ATOM   2575  HB2 SER A 214       6.265  25.255 -72.880  1.00103.78           H
ATOM   2576  HB3 SER A 214       5.914  25.999 -74.430  1.00103.78           H
ATOM   2577  HG  SER A 214       6.066  23.776 -75.284  1.00110.89           H
ATOM   2578  N   ILE A 215       8.788  24.378 -72.177  1.00 86.52           N
ATOM   2579  CA  ILE A 215       9.570  23.536 -71.280  1.00 74.91           C
ATOM   2580  C   ILE A 215       8.628  22.844 -70.305  1.00 73.44           C
ATOM   2581  O   ILE A 215       7.796  23.495 -69.659  1.00 68.89           O
ATOM   2582  CB  ILE A 215      10.636  24.349 -70.527  1.00 74.06           C
ATOM   2583  CG1 ILE A 215      11.606  24.994 -71.518  1.00 74.93           C
ATOM   2584  CG2 ILE A 215      11.377  23.467 -69.533  1.00 70.75           C
ATOM   2585  CD1 ILE A 215      12.652  25.870 -70.865  1.00 73.96           C
ATOM   2586  H   ILE A 215       8.360  25.198 -71.773  1.00 86.52           H
ATOM   2587  HA  ILE A 215      10.101  22.758 -71.834  1.00 74.91           H
ATOM   2588  HB  ILE A 215      10.182  25.127 -69.924  1.00 74.06           H
ATOM   2589 HG12 ILE A 215      12.104  24.218 -72.101  1.00 74.93           H
ATOM   2590 HG13 ILE A 215      11.068  25.608 -72.241  1.00 74.93           H
ATOM   2591 HG21 ILE A 215      12.158  24.019 -69.011  1.00 70.75           H
ATOM   2592 HG22 ILE A 215      10.740  23.064 -68.747  1.00 70.75           H
ATOM   2593 HG23 ILE A 215      11.853  22.624 -70.035  1.00 70.75           H
ATOM   2594 HD11 ILE A 215      13.024  26.615 -71.569  1.00 73.96           H
ATOM   2595 HD12 ILE A 215      12.252  26.406 -70.004  1.00 73.96           H
```

ATOM 2596 HD13 ILE A 215 13.508 25.283 -70.531 1.00 73.96 H
ATOM 2597 N ASP A 216 8.767 21.528 -70.196 1.00 63.22 N
ATOM 2598 CA ASP A 216 7.994 20.717 -69.267 1.00 60.23 C
ATOM 2599 C ASP A 216 8.923 20.226 -68.166 1.00 67.54 C
ATOM 2600 O ASP A 216 9.946 19.593 -68.451 1.00 71.64 O
ATOM 2601 CB ASP A 216 7.354 19.506 -70.004 1.00 67.22 C
ATOM 2602 CG ASP A 216 6.342 18.669 -69.203 1.00 79.44 C
ATOM 2603 OD1 ASP A 216 6.007 19.043 -68.059 1.00 87.02 O
ATOM 2604 OD2 ASP A 216 5.864 17.665 -69.771 1.00 86.62 O1-
ATOM 2605 H ASP A 216 9.467 21.042 -70.741 1.00 63.22 H
ATOM 2606 HA ASP A 216 7.203 21.314 -68.808 1.00 60.23 H
ATOM 2607 HB2 ASP A 216 6.845 19.876 -70.891 1.00 67.22 H
ATOM 2608 HB3 ASP A 216 8.146 18.834 -70.340 1.00 67.22 H
ATOM 2609 N CYS A 217 8.579 20.534 -66.917 1.00 67.20 N
ATOM 2610 CA CYS A 217 9.239 19.928 -65.763 1.00 53.84 C
ATOM 2611 C CYS A 217 8.536 18.603 -65.500 1.00 58.60 C
ATOM 2612 O CYS A 217 7.434 18.567 -64.949 1.00 64.11 O
ATOM 2613 CB CYS A 217 9.185 20.905 -64.573 1.00 53.94 C
ATOM 2614 SG CYS A 217 9.688 20.258 -62.958 1.00 63.09 S
ATOM 2615 H CYS A 217 7.734 21.058 -66.737 1.00 67.20 H
ATOM 2616 HA CYS A 217 10.293 19.740 -65.977 1.00 53.84 H
ATOM 2617 HB2 CYS A 217 9.841 21.741 -64.792 1.00 53.94 H
ATOM 2618 HB3 CYS A 217 8.181 21.313 -64.455 1.00 53.94 H
ATOM 2619 N THR A 218 9.162 17.507 -65.919 1.00 59.73 N
ATOM 2620 CA THR A 218 8.511 16.207 -65.947 1.00 58.65 C
ATOM 2621 C THR A 218 9.435 15.157 -65.340 1.00 53.89 C
ATOM 2622 O THR A 218 10.508 15.466 -64.813 1.00 56.41 O
ATOM 2623 CB THR A 218 8.056 15.871 -67.407 1.00 60.96 C
ATOM 2624 CG2 THR A 218 9.204 15.798 -68.428 1.00 52.90 C
ATOM 2625 OG1 THR A 218 7.315 14.663 -67.525 1.00 66.56 O
ATOM 2626 H THR A 218 10.090 17.562 -66.317 1.00 59.73 H
ATOM 2627 HA THR A 218 7.619 16.192 -65.320 1.00 58.65 H
ATOM 2628 HB THR A 218 7.377 16.660 -67.716 1.00 60.96 H
ATOM 2629 HG1 THR A 218 6.936 14.600 -68.414 1.00 66.56 H
ATOM 2630 HG21 THR A 218 8.825 15.627 -69.436 1.00 52.90 H
ATOM 2631 HG22 THR A 218 9.764 16.733 -68.450 1.00 52.90 H
ATOM 2632 HG23 THR A 218 9.906 14.995 -68.196 1.00 52.90 H
ATOM 2633 N LEU A 219 9.000 13.904 -65.412 1.00 48.31 N
ATOM 2634 CA LEU A 219 9.744 12.764 -64.896 1.00 49.86 C
ATOM 2635 C LEU A 219 10.194 11.880 -66.051 1.00 53.06 C

ATOM	2636	O	LEU	A	219	9.433	11.641	-66.994	1.00	52.12	O
ATOM	2637	CB	LEU	A	219	8.840	11.954	-63.933	1.00	46.61	C
ATOM	2638	CG	LEU	A	219	8.373	12.711	-62.666	1.00	50.60	C
ATOM	2639	CD1	LEU	A	219	7.338	11.893	-61.865	1.00	47.08	C
ATOM	2640	CD2	LEU	A	219	9.551	13.170	-61.788	1.00	50.44	C
ATOM	2641	H	LEU	A	219	8.134	13.710	-65.900	1.00	48.31	H
ATOM	2642	HA	LEU	A	219	10.636	13.086	-64.360	1.00	49.86	H
ATOM	2643	HB2	LEU	A	219	7.959	11.623	-64.486	1.00	46.61	H
ATOM	2644	HB3	LEU	A	219	9.359	11.042	-63.632	1.00	46.61	H
ATOM	2645	HG	LEU	A	219	7.853	13.613	-62.997	1.00	50.60	H
ATOM	2646	HD11	LEU	A	219	7.661	11.661	-60.850	1.00	47.08	H
ATOM	2647	HD12	LEU	A	219	6.399	12.441	-61.789	1.00	47.08	H
ATOM	2648	HD13	LEU	A	219	7.111	10.942	-62.345	1.00	47.08	H
ATOM	2649	HD21	LEU	A	219	9.384	13.004	-60.726	1.00	50.44	H
ATOM	2650	HD22	LEU	A	219	10.479	12.664	-62.048	1.00	50.44	H
ATOM	2651	HD23	LEU	A	219	9.714	14.239	-61.922	1.00	50.44	H
ATOM	2652	N	THR	A	220	11.432	11.401	-65.976	1.00	47.93	N
ATOM	2653	CA	THR	A	220	11.973	10.459	-66.947	1.00	54.51	C
ATOM	2654	C	THR	A	220	12.137	9.101	-66.280	1.00	50.06	C
ATOM	2655	O	THR	A	220	12.628	9.014	-65.150	1.00	53.32	O
ATOM	2656	CB	THR	A	220	13.346	10.919	-67.498	1.00	64.09	C
ATOM	2657	CG2	THR	A	220	13.218	12.177	-68.366	1.00	68.94	C
ATOM	2658	OG1	THR	A	220	14.282	11.195	-66.472	1.00	76.32	O
ATOM	2659	H	THR	A	220	12.044	11.663	-65.214	1.00	47.93	H
ATOM	2660	HA	THR	A	220	11.297	10.328	-67.795	1.00	54.51	H
ATOM	2661	HB	THR	A	220	13.769	10.124	-68.116	1.00	64.09	H
ATOM	2662	HG1	THR	A	220	14.255	10.473	-65.833	1.00	76.32	H
ATOM	2663	HG21	THR	A	220	14.192	12.513	-68.723	1.00	68.94	H
ATOM	2664	HG22	THR	A	220	12.596	11.984	-69.240	1.00	68.94	H
ATOM	2665	HG23	THR	A	220	12.757	12.994	-67.815	1.00	68.94	H
ATOM	2666	N	PHE	A	221	11.726	8.049	-66.978	1.00	46.79	N
ATOM	2667	CA	PHE	A	221	11.647	6.718	-66.403	1.00	48.89	C
ATOM	2668	C	PHE	A	221	12.604	5.770	-67.112	1.00	53.99	C
ATOM	2669	O	PHE	A	221	13.079	6.039	-68.218	1.00	48.36	O
ATOM	2670	CB	PHE	A	221	10.200	6.204	-66.561	1.00	45.77	C
ATOM	2671	CG	PHE	A	221	9.164	6.982	-65.776	1.00	52.56	C
ATOM	2672	CD1	PHE	A	221	8.883	6.636	-64.439	1.00	47.57	C
ATOM	2673	CD2	PHE	A	221	8.593	8.154	-66.316	1.00	49.89	C
ATOM	2674	CE1	PHE	A	221	7.989	7.399	-63.702	1.00	54.42	C
ATOM	2675	CE2	PHE	A	221	7.712	8.910	-65.559	1.00	49.18	C

ATOM	2676	CZ	PHE	A	221	7.403	8.529	-64.261	1.00	45.75	C
ATOM	2677	H	PHE	A	221	11.366	8.160	-67.914	1.00	46.79	H
ATOM	2678	HA	PHE	A	221	11.929	6.718	-65.348	1.00	48.89	H
ATOM	2679	HB2	PHE	A	221	9.913	6.211	-67.613	1.00	45.77	H
ATOM	2680	HB3	PHE	A	221	10.143	5.161	-66.253	1.00	45.77	H
ATOM	2681	HD1	PHE	A	221	9.339	5.767	-63.986	1.00	47.57	H
ATOM	2682	HD2	PHE	A	221	8.825	8.471	-67.323	1.00	49.89	H
ATOM	2683	HE1	PHE	A	221	7.742	7.098	-62.696	1.00	54.42	H
ATOM	2684	HE2	PHE	A	221	7.262	9.794	-65.989	1.00	49.18	H
ATOM	2685	HZ	PHE	A	221	6.696	9.113	-63.692	1.00	45.75	H
ATOM	2686	N	SER	A	222	12.882	4.650	-66.451	1.00	54.95	N
ATOM	2687	CA	SER	A	222	13.674	3.595	-67.057	1.00	57.52	C
ATOM	2688	C	SER	A	222	12.869	2.895	-68.150	1.00	50.96	C
ATOM	2689	O	SER	A	222	11.653	3.067	-68.276	1.00	47.72	O
ATOM	2690	CB	SER	A	222	14.119	2.584	-66.000	1.00	59.56	C
ATOM	2691	OG	SER	A	222	14.819	3.217	-64.942	1.00	65.98	O
ATOM	2692	H	SER	A	222	12.504	4.472	-65.532	1.00	54.95	H
ATOM	2693	HA	SER	A	222	14.568	4.032	-67.508	1.00	57.52	H
ATOM	2694	HB2	SER	A	222	13.259	2.062	-65.578	1.00	59.56	H
ATOM	2695	HB3	SER	A	222	14.769	1.824	-66.435	1.00	59.56	H
ATOM	2696	HG	SER	A	222	15.082	2.549	-64.304	1.00	65.98	H
ATOM	2697	N	HIS	A	223	13.563	2.092	-68.941	1.00	51.15	N
ATOM	2698	CA	HIS	A	223	12.876	1.338	-69.979	1.00	54.24	C
ATOM	2699	C	HIS	A	223	12.220	0.096	-69.377	1.00	48.11	C
ATOM	2700	O	HIS	A	223	12.795	-0.541	-68.491	1.00	54.20	O
ATOM	2701	CB	HIS	A	223	13.852	0.927	-71.081	1.00	60.35	C
ATOM	2702	CG	HIS	A	223	14.639	2.068	-71.646	1.00	76.95	C
ATOM	2703	CD2	HIS	A	223	14.063	3.070	-72.397	1.00	82.30	C
ATOM	2704	ND1	HIS	A	223	15.958	2.363	-71.574	1.00	81.43	N
ATOM	2705	CE1	HIS	A	223	16.152	3.529	-72.275	1.00	85.70	C
ATOM	2706	NE2	HIS	A	223	14.993	3.934	-72.761	1.00	85.52	N
ATOM	2707	H	HIS	A	223	14.557	1.955	-68.830	1.00	51.15	H
ATOM	2708	HA	HIS	A	223	12.173	2.028	-70.440	1.00	54.24	H
ATOM	2709	HB2	HIS	A	223	14.562	0.188	-70.704	1.00	60.35	H
ATOM	2710	HB3	HIS	A	223	13.314	0.445	-71.899	1.00	60.35	H
ATOM	2711	HD2	HIS	A	223	13.032	3.210	-72.688	1.00	82.30	H
ATOM	2712	HE1	HIS	A	223	17.099	4.032	-72.406	1.00	85.70	H
ATOM	2713	HE2	HIS	A	223	14.845	4.765	-73.316	1.00	0.00	H
ATOM	2714	N	PRO	A	224	11.003	-0.257	-69.819	1.00	47.34	N
ATOM	2715	CA	PRO	A	224	10.189	0.546	-70.738	1.00	45.22	C

ATOM 2716 C PRO A 224 9.408 1.622 -69.990 1.00 44.62 C
ATOM 2717 O PRO A 224 8.883 1.352 -68.908 1.00 41.02 O
ATOM 2718 CB PRO A 224 9.252 -0.485 -71.359 1.00 46.82 C
ATOM 2719 CG PRO A 224 9.065 -1.497 -70.280 1.00 46.77 C
ATOM 2720 CD PRO A 224 10.376 -1.558 -69.526 1.00 46.51 C
ATOM 2721 HA PRO A 224 10.758 0.983 -71.559 1.00 45.22 H
ATOM 2722 HB2 PRO A 224 8.314 -0.041 -71.673 1.00 46.82 H
ATOM 2723 HB3 PRO A 224 9.722 -0.943 -72.230 1.00 46.82 H
ATOM 2724 HG2 PRO A 224 8.282 -1.148 -69.613 1.00 46.77 H
ATOM 2725 HG3 PRO A 224 8.746 -2.469 -70.658 1.00 46.77 H
ATOM 2726 HD2 PRO A 224 10.226 -1.719 -68.457 1.00 46.51 H
ATOM 2727 HD3 PRO A 224 11.010 -2.356 -69.915 1.00 46.51 H
ATOM 2728 N THR A 225 9.337 2.825 -70.565 1.00 44.25 N
ATOM 2729 CA THR A 225 8.732 3.950 -69.858 1.00 45.39 C
ATOM 2730 C THR A 225 7.260 3.696 -69.559 1.00 38.94 C
ATOM 2731 O THR A 225 6.763 4.085 -68.497 1.00 42.54 O
ATOM 2732 CB THR A 225 8.897 5.236 -70.669 1.00 43.86 C
ATOM 2733 CG2 THR A 225 10.368 5.597 -70.800 1.00 47.12 C
ATOM 2734 OG1 THR A 225 8.337 5.050 -71.974 1.00 48.48 O
ATOM 2735 H THR A 225 9.757 3.014 -71.464 1.00 44.25 H
ATOM 2736 HA THR A 225 9.243 4.074 -68.901 1.00 45.39 H
ATOM 2737 HB THR A 225 8.371 6.059 -70.181 1.00 43.86 H
ATOM 2738 HG1 THR A 225 7.384 4.972 -71.889 1.00 48.48 H
ATOM 2739 HG21 THR A 225 10.490 6.536 -71.341 1.00 47.12 H
ATOM 2740 HG22 THR A 225 10.829 5.724 -69.821 1.00 47.12 H
ATOM 2741 HG23 THR A 225 10.937 4.836 -71.336 1.00 47.12 H
ATOM 2742 N TRP A 226 6.548 3.032 -70.475 1.00 38.02 N
ATOM 2743 CA TRP A 226 5.122 2.803 -70.270 1.00 39.85 C
ATOM 2744 C TRP A 226 4.839 1.877 -69.093 1.00 47.19 C
ATOM 2745 O TRP A 226 3.725 1.900 -68.558 1.00 43.69 O
ATOM 2746 CB TRP A 226 4.476 2.249 -71.544 1.00 42.72 C
ATOM 2747 CG TRP A 226 5.272 1.189 -72.245 1.00 50.16 C
ATOM 2748 CD1 TRP A 226 6.125 1.366 -73.297 1.00 54.02 C
ATOM 2749 CD2 TRP A 226 5.277 -0.214 -71.957 1.00 46.58 C
ATOM 2750 CE2 TRP A 226 6.161 -0.825 -72.869 1.00 52.52 C
ATOM 2751 CE3 TRP A 226 4.624 -1.013 -71.013 1.00 44.50 C
ATOM 2752 NE1 TRP A 226 6.665 0.161 -73.676 1.00 52.01 N
ATOM 2753 CZ2 TRP A 226 6.409 -2.197 -72.865 1.00 54.11 C
ATOM 2754 CZ3 TRP A 226 4.871 -2.376 -71.011 1.00 40.82 C
ATOM 2755 CH2 TRP A 226 5.756 -2.953 -71.930 1.00 38.28 C

ATOM 2756 H TRP A 226 6.969 2.691 -71.327 1.00 38.02 H
ATOM 2757 HA TRP A 226 4.632 3.751 -70.036 1.00 39.85 H
ATOM 2758 HB2 TRP A 226 3.468 1.878 -71.351 1.00 42.72 H
ATOM 2759 HB3 TRP A 226 4.350 3.070 -72.251 1.00 42.72 H
ATOM 2760 HD1 TRP A 226 6.335 2.322 -73.755 1.00 54.02 H
ATOM 2761 HE1 TRP A 226 7.319 0.048 -74.437 1.00 52.01 H
ATOM 2762 HE3 TRP A 226 3.935 -0.582 -70.302 1.00 44.50 H
ATOM 2763 HZ2 TRP A 226 7.092 -2.647 -73.571 1.00 54.11 H
ATOM 2764 HZ3 TRP A 226 4.373 -3.004 -70.287 1.00 40.82 H
ATOM 2765 HH2 TRP A 226 5.928 -4.019 -71.900 1.00 38.28 H
ATOM 2766 N TYR A 227 5.810 1.070 -68.670 1.00 36.79 N
ATOM 2767 CA TYR A 227 5.608 0.270 -67.467 1.00 34.92 C
ATOM 2768 C TYR A 227 5.864 1.099 -66.213 1.00 35.02 C
ATOM 2769 O TYR A 227 4.987 1.237 -65.353 1.00 36.23 O
ATOM 2770 CB TYR A 227 6.510 -0.970 -67.489 1.00 34.39 C
ATOM 2771 CG TYR A 227 6.290 -1.906 -66.316 1.00 37.16 C
ATOM 2772 CD1 TYR A 227 6.934 -1.694 -65.099 1.00 38.71 C
ATOM 2773 CD2 TYR A 227 5.434 -2.998 -66.422 1.00 34.42 C
ATOM 2774 CE1 TYR A 227 6.731 -2.540 -64.025 1.00 38.49 C
ATOM 2775 CE2 TYR A 227 5.224 -3.849 -65.351 1.00 36.70 C
ATOM 2776 CZ TYR A 227 5.879 -3.615 -64.156 1.00 36.69 C
ATOM 2777 OH TYR A 227 5.682 -4.458 -63.089 1.00 38.13 O
ATOM 2778 H TYR A 227 6.725 1.067 -69.100 1.00 36.79 H
ATOM 2779 HA TYR A 227 4.581 -0.100 -67.424 1.00 34.92 H
ATOM 2780 HB2 TYR A 227 6.331 -1.530 -68.407 1.00 34.39 H
ATOM 2781 HB3 TYR A 227 7.562 -0.682 -67.506 1.00 34.39 H
ATOM 2782 HD1 TYR A 227 7.607 -0.860 -64.988 1.00 38.71 H
ATOM 2783 HD2 TYR A 227 4.918 -3.187 -67.352 1.00 34.42 H
ATOM 2784 HE1 TYR A 227 7.244 -2.357 -63.092 1.00 38.49 H
ATOM 2785 HE2 TYR A 227 4.555 -4.691 -65.451 1.00 36.70 H
ATOM 2786 HH TYR A 227 6.185 -4.207 -62.311 1.00 38.13 H
ATOM 2787 N TRP A 228 7.066 1.668 -66.098 1.00 35.69 N
ATOM 2788 CA TRP A 228 7.461 2.303 -64.845 1.00 39.72 C
ATOM 2789 C TRP A 228 6.696 3.596 -64.585 1.00 35.87 C
ATOM 2790 O TRP A 228 6.458 3.944 -63.423 1.00 36.20 O
ATOM 2791 CB TRP A 228 8.971 2.545 -64.841 1.00 39.16 C
ATOM 2792 CG TRP A 228 9.737 1.263 -64.944 1.00 40.87 C
ATOM 2793 CD1 TRP A 228 10.457 0.817 -66.016 1.00 40.68 C
ATOM 2794 CD2 TRP A 228 9.825 0.238 -63.948 1.00 40.69 C
ATOM 2795 CE2 TRP A 228 10.626 -0.793 -64.478 1.00 44.15 C

```
ATOM   2796  CE3 TRP A 228       9.311   0.096 -62.654  1.00 42.68           C
ATOM   2797  NE1 TRP A 228      11.000  -0.414 -65.741  1.00 43.77           N
ATOM   2798  CZ2 TRP A 228      10.926  -1.948 -63.761  1.00 45.24           C
ATOM   2799  CZ3 TRP A 228       9.608  -1.055 -61.944  1.00 43.77           C
ATOM   2800  CH2 TRP A 228      10.408  -2.061 -62.500  1.00 43.51           C
ATOM   2801  H   TRP A 228       7.766   1.572 -66.821  1.00 35.69           H
ATOM   2802  HA  TRP A 228       7.226   1.625 -64.022  1.00 39.72           H
ATOM   2803  HB2 TRP A 228       9.251   3.185 -65.677  1.00 39.16           H
ATOM   2804  HB3 TRP A 228       9.279   3.058 -63.929  1.00 39.16           H
ATOM   2805  HD1 TRP A 228      10.563   1.355 -66.941  1.00 40.68           H
ATOM   2806  HE1 TRP A 228      11.578  -0.930 -66.391  1.00 43.77           H
ATOM   2807  HE3 TRP A 228       8.701   0.873 -62.216  1.00 42.68           H
ATOM   2808  HZ2 TRP A 228      11.543  -2.727 -64.185  1.00 45.24           H
ATOM   2809  HZ3 TRP A 228       9.224  -1.178 -60.942  1.00 43.77           H
ATOM   2810  HH2 TRP A 228      10.623  -2.946 -61.919  1.00 43.51           H
ATOM   2811  N   GLU A 229       6.300   4.318 -65.636  1.00 35.95           N
ATOM   2812  CA  GLU A 229       5.499   5.520 -65.421  1.00 42.81           C
ATOM   2813  C   GLU A 229       4.133   5.172 -64.847  1.00 36.50           C
ATOM   2814  O   GLU A 229       3.654   5.833 -63.918  1.00 39.47           O
ATOM   2815  CB  GLU A 229       5.395   6.293 -66.760  1.00 39.70           C
ATOM   2816  CG  GLU A 229       4.515   7.561 -66.732  1.00 61.33           C
ATOM   2817  CD  GLU A 229       4.480   8.340 -68.050  1.00 69.54           C
ATOM   2818  OE1 GLU A 229       5.418   8.191 -68.862  1.00 68.17           O
ATOM   2819  OE2 GLU A 229       3.495   9.089 -68.228  1.00 74.45           O1-
ATOM   2820  H   GLU A 229       6.506   4.044 -66.589  1.00 35.95           H
ATOM   2821  HA  GLU A 229       5.999   6.158 -64.698  1.00 42.81           H
ATOM   2822  HB2 GLU A 229       6.401   6.557 -67.086  1.00 39.70           H
ATOM   2823  HB3 GLU A 229       4.995   5.631 -67.528  1.00 39.70           H
ATOM   2824  HG2 GLU A 229       3.492   7.272 -66.502  1.00 61.33           H
ATOM   2825  HG3 GLU A 229       4.847   8.234 -65.942  1.00 61.33           H
ATOM   2826  N   ASN A 230       3.493   4.133 -65.381  1.00 35.07           N
ATOM   2827  CA  ASN A 230       2.183   3.750 -64.876  1.00 41.13           C
ATOM   2828  C   ASN A 230       2.279   3.089 -63.507  1.00 42.78           C
ATOM   2829  O   ASN A 230       1.373   3.249 -62.682  1.00 40.17           O
ATOM   2830  CB  ASN A 230       1.488   2.847 -65.890  1.00 41.06           C
ATOM   2831  CG  ASN A 230       1.051   3.608 -67.124  1.00 42.59           C
ATOM   2832  ND2 ASN A 230       1.450   3.129 -68.297  1.00 41.24           N
ATOM   2833  OD1 ASN A 230       0.380   4.633 -67.021  1.00 44.14           O
ATOM   2834  H   ASN A 230       3.896   3.590 -66.131  1.00 35.07           H
ATOM   2835  HA  ASN A 230       1.592   4.663 -64.766  1.00 41.13           H
```

ATOM 2836 HB2 ASN A 230 2.121 1.996 -66.151 1.00 41.06 H
ATOM 2837 HB3 ASN A 230 0.580 2.429 -65.452 1.00 41.06 H
ATOM 2838 HD21 ASN A 230 2.085 2.340 -68.345 1.00 41.24 H
ATOM 2839 HD22 ASN A 230 1.181 3.597 -69.150 1.00 41.24 H
ATOM 2840 N LEU A 231 3.369 2.367 -63.237 1.00 41.11 N
ATOM 2841 CA LEU A 231 3.573 1.831 -61.894 1.00 38.53 C
ATOM 2842 C LEU A 231 3.663 2.951 -60.865 1.00 40.74 C
ATOM 2843 O LEU A 231 3.099 2.844 -59.769 1.00 42.65 O
ATOM 2844 CB LEU A 231 4.832 0.966 -61.849 1.00 36.91 C
ATOM 2845 CG LEU A 231 5.149 0.389 -60.464 1.00 48.84 C
ATOM 2846 CD1 LEU A 231 4.047 -0.561 -60.005 1.00 43.51 C
ATOM 2847 CD2 LEU A 231 6.500 -0.304 -60.448 1.00 48.08 C
ATOM 2848 H LEU A 231 4.090 2.212 -63.930 1.00 41.11 H
ATOM 2849 HA LEU A 231 2.711 1.208 -61.647 1.00 38.53 H
ATOM 2850 HB2 LEU A 231 4.744 0.156 -62.576 1.00 36.91 H
ATOM 2851 HB3 LEU A 231 5.682 1.565 -62.178 1.00 36.91 H
ATOM 2852 HG LEU A 231 5.232 1.183 -59.722 1.00 48.84 H
ATOM 2853 HD11 LEU A 231 4.415 -1.415 -59.437 1.00 43.51 H
ATOM 2854 HD12 LEU A 231 3.340 -0.042 -59.358 1.00 43.51 H
ATOM 2855 HD13 LEU A 231 3.476 -0.961 -60.844 1.00 43.51 H
ATOM 2856 HD21 LEU A 231 6.504 -1.253 -59.913 1.00 48.08 H
ATOM 2857 HD22 LEU A 231 6.840 -0.518 -61.460 1.00 48.08 H
ATOM 2858 HD23 LEU A 231 7.254 0.328 -59.976 1.00 48.08 H
ATOM 2859 N LEU A 232 4.368 4.037 -61.198 1.00 36.08 N
ATOM 2860 CA LEU A 232 4.448 5.171 -60.282 1.00 37.60 C
ATOM 2861 C LEU A 232 3.067 5.753 -60.014 1.00 33.96 C
ATOM 2862 O LEU A 232 2.700 6.004 -58.860 1.00 37.61 O
ATOM 2863 CB LEU A 232 5.425 6.268 -60.786 1.00 40.68 C
ATOM 2864 CG LEU A 232 5.660 7.428 -59.769 1.00 38.72 C
ATOM 2865 CD1 LEU A 232 6.409 6.960 -58.502 1.00 40.78 C
ATOM 2866 CD2 LEU A 232 6.318 8.664 -60.416 1.00 43.48 C
ATOM 2867 H LEU A 232 4.843 4.109 -62.088 1.00 36.08 H
ATOM 2868 HA LEU A 232 4.853 4.808 -59.336 1.00 37.60 H
ATOM 2869 HB2 LEU A 232 6.383 5.816 -61.045 1.00 40.68 H
ATOM 2870 HB3 LEU A 232 5.033 6.679 -61.718 1.00 40.68 H
ATOM 2871 HG LEU A 232 4.688 7.792 -59.434 1.00 38.72 H
ATOM 2872 HD11 LEU A 232 7.256 7.596 -58.246 1.00 40.78 H
ATOM 2873 HD12 LEU A 232 5.743 6.963 -57.638 1.00 40.78 H
ATOM 2874 HD13 LEU A 232 6.799 5.947 -58.605 1.00 40.78 H
ATOM 2875 HD21 LEU A 232 5.695 9.549 -60.272 1.00 43.48 H

ATOM 2876 HD22 LEU A 232 7.294 8.900 -59.996 1.00 43.48 H
ATOM 2877 HD23 LEU A 232 6.445 8.544 -61.488 1.00 43.48 H
ATOM 2878 N LYS A 233 2.286 5.974 -61.075 1.00 31.01 N
ATOM 2879 CA LYS A 233 0.939 6.509 -60.906 1.00 35.83 C
ATOM 2880 C LYS A 233 0.087 5.592 -60.039 1.00 38.98 C
ATOM 2881 O LYS A 233 -0.673 6.062 -59.184 1.00 35.70 O
ATOM 2882 CB LYS A 233 0.272 6.710 -62.282 1.00 32.70 C
ATOM 2883 CG LYS A 233 0.927 7.804 -63.143 1.00 33.94 C
ATOM 2884 CD LYS A 233 0.415 7.780 -64.592 1.00 34.02 C
ATOM 2885 CE LYS A 233 1.151 8.774 -65.500 1.00 41.13 C
ATOM 2886 NZ LYS A 233 0.859 8.509 -66.928 1.00 48.12 N1+
ATOM 2887 H LYS A 233 2.604 5.771 -62.013 1.00 31.01 H
ATOM 2888 HA LYS A 233 1.015 7.474 -60.401 1.00 35.83 H
ATOM 2889 HB2 LYS A 233 0.274 5.761 -62.821 1.00 32.70 H
ATOM 2890 HB3 LYS A 233 -0.780 6.970 -62.144 1.00 32.70 H
ATOM 2891 HG2 LYS A 233 0.734 8.781 -62.698 1.00 33.94 H
ATOM 2892 HG3 LYS A 233 2.010 7.683 -63.141 1.00 33.94 H
ATOM 2893 HD2 LYS A 233 0.520 6.774 -64.999 1.00 34.02 H
ATOM 2894 HD3 LYS A 233 -0.652 8.003 -64.602 1.00 34.02 H
ATOM 2895 HE2 LYS A 233 0.885 9.803 -65.256 1.00 41.13 H
ATOM 2896 HE3 LYS A 233 2.226 8.687 -65.351 1.00 41.13 H
ATOM 2897 HZ1 LYS A 233 1.022 7.532 -67.126 1.00 48.12 H
ATOM 2898 HZ2 LYS A 233 -0.101 8.739 -67.136 1.00 48.12 H
ATOM 2899 HZ3 LYS A 233 1.482 9.061 -67.502 1.00 48.12 H
ATOM 2900 N ILE A 234 0.213 4.278 -60.237 1.00 37.77 N
ATOM 2901 CA ILE A 234 -0.554 3.322 -59.446 1.00 35.96 C
ATOM 2902 C ILE A 234 -0.105 3.342 -57.991 1.00 33.24 C
ATOM 2903 O ILE A 234 -0.934 3.321 -57.074 1.00 42.00 O
ATOM 2904 CB ILE A 234 -0.488 1.881 -60.042 1.00 41.35 C
ATOM 2905 CG1 ILE A 234 -1.329 1.782 -61.338 1.00 49.39 C
ATOM 2906 CG2 ILE A 234 -0.840 0.722 -59.079 1.00 41.05 C
ATOM 2907 CD1 ILE A 234 -2.842 2.011 -61.164 1.00 63.19 C
ATOM 2908 H ILE A 234 0.826 3.911 -60.954 1.00 37.77 H
ATOM 2909 HA ILE A 234 -1.596 3.642 -59.427 1.00 35.96 H
ATOM 2910 HB ILE A 234 0.547 1.709 -60.343 1.00 41.35 H
ATOM 2911 HG12 ILE A 234 -0.967 2.515 -62.059 1.00 49.39 H
ATOM 2912 HG13 ILE A 234 -1.159 0.814 -61.811 1.00 49.39 H
ATOM 2913 HG21 ILE A 234 -0.866 -0.232 -59.607 1.00 41.05 H
ATOM 2914 HG22 ILE A 234 -0.101 0.615 -58.285 1.00 41.05 H
ATOM 2915 HG23 ILE A 234 -1.811 0.864 -58.604 1.00 41.05 H

ATOM 2916 HD11 ILE A 234 -3.409 1.392 -61.860 1.00 63.19 H
ATOM 2917 HD12 ILE A 234 -3.194 1.777 -60.160 1.00 63.19 H
ATOM 2918 HD13 ILE A 234 -3.102 3.050 -61.368 1.00 63.19 H
ATOM 2919 N CYS A 235 1.210 3.378 -57.754 1.00 39.51 N
ATOM 2920 CA CYS A 235 1.711 3.399 -56.383 1.00 42.53 C
ATOM 2921 C CYS A 235 1.305 4.678 -55.665 1.00 44.32 C
ATOM 2922 O CYS A 235 0.994 4.653 -54.469 1.00 40.71 O
ATOM 2923 CB CYS A 235 3.247 3.288 -56.333 1.00 47.98 C
ATOM 2924 SG CYS A 235 3.779 1.654 -56.913 1.00 59.39 S
ATOM 2925 H CYS A 235 1.885 3.374 -58.510 1.00 39.51 H
ATOM 2926 HA CYS A 235 1.322 2.536 -55.852 1.00 42.53 H
ATOM 2927 HB2 CYS A 235 3.719 4.061 -56.944 1.00 47.98 H
ATOM 2928 HB3 CYS A 235 3.612 3.420 -55.313 1.00 47.98 H
ATOM 2929 HG CYS A 235 3.499 1.891 -58.205 1.00 59.39 H
ATOM 2930 N VAL A 236 1.313 5.809 -56.374 1.00 39.48 N
ATOM 2931 CA VAL A 236 0.850 7.054 -55.771 1.00 40.98 C
ATOM 2932 C VAL A 236 -0.627 6.950 -55.422 1.00 33.44 C
ATOM 2933 O VAL A 236 -1.052 7.349 -54.332 1.00 39.94 O
ATOM 2934 CB VAL A 236 1.134 8.290 -56.675 1.00 40.85 C
ATOM 2935 CG1 VAL A 236 0.475 9.601 -56.196 1.00 34.49 C
ATOM 2936 CG2 VAL A 236 2.651 8.535 -56.819 1.00 37.62 C
ATOM 2937 H VAL A 236 1.605 5.827 -57.344 1.00 39.48 H
ATOM 2938 HA VAL A 236 1.398 7.211 -54.841 1.00 40.98 H
ATOM 2939 HB VAL A 236 0.746 8.070 -57.671 1.00 40.85 H
ATOM 2940 HG11 VAL A 236 0.789 10.449 -56.806 1.00 34.49 H
ATOM 2941 HG12 VAL A 236 -0.614 9.564 -56.247 1.00 34.49 H
ATOM 2942 HG13 VAL A 236 0.746 9.829 -55.164 1.00 34.49 H
ATOM 2943 HG21 VAL A 236 2.856 9.224 -57.639 1.00 37.62 H
ATOM 2944 HG22 VAL A 236 3.075 8.969 -55.913 1.00 37.62 H
ATOM 2945 HG23 VAL A 236 3.207 7.623 -57.024 1.00 37.62 H
ATOM 2946 N PHE A 237 -1.427 6.392 -56.333 1.00 34.16 N
ATOM 2947 CA PHE A 237 -2.862 6.272 -56.098 1.00 38.59 C
ATOM 2948 C PHE A 237 -3.168 5.402 -54.884 1.00 38.19 C
ATOM 2949 O PHE A 237 -4.100 5.696 -54.127 1.00 42.09 O
ATOM 2950 CB PHE A 237 -3.562 5.772 -57.381 1.00 35.21 C
ATOM 2951 CG PHE A 237 -5.075 5.591 -57.323 1.00 41.21 C
ATOM 2952 CD1 PHE A 237 -5.902 6.541 -56.681 1.00 41.72 C
ATOM 2953 CD2 PHE A 237 -5.684 4.560 -58.069 1.00 40.94 C
ATOM 2954 CE1 PHE A 237 -7.283 6.421 -56.738 1.00 45.53 C
ATOM 2955 CE2 PHE A 237 -7.068 4.454 -58.110 1.00 37.81 C

```
ATOM   2956  CZ  PHE A 237      -7.864   5.378 -57.445  1.00 41.50           C
ATOM   2957  H   PHE A 237      -1.066   6.069 -57.222  1.00 34.16           H
ATOM   2958  HA  PHE A 237      -3.254   7.271 -55.901  1.00 38.59           H
ATOM   2959  HB2 PHE A 237      -3.357   6.467 -58.197  1.00 35.21           H
ATOM   2960  HB3 PHE A 237      -3.114   4.824 -57.678  1.00 35.21           H
ATOM   2961  HD1 PHE A 237      -5.473   7.365 -56.132  1.00 41.72           H
ATOM   2962  HD2 PHE A 237      -5.080   3.848 -58.613  1.00 40.94           H
ATOM   2963  HE1 PHE A 237      -7.909   7.145 -56.236  1.00 45.53           H
ATOM   2964  HE2 PHE A 237      -7.529   3.655 -58.674  1.00 37.81           H
ATOM   2965  HZ  PHE A 237      -8.940   5.291 -57.487  1.00 41.50           H
ATOM   2966  N   ILE A 238      -2.384   4.350 -54.658  0.57 38.27           N
ATOM   2967  CA  ILE A 238      -2.703   3.419 -53.581  0.57 40.87           C
ATOM   2968  C   ILE A 238      -2.078   3.821 -52.245  0.57 39.50           C
ATOM   2969  O   ILE A 238      -2.669   3.566 -51.192  0.57 41.36           O
ATOM   2970  CB  ILE A 238      -2.335   1.942 -53.902  0.57 47.23           C
ATOM   2971  CG1 ILE A 238      -0.818   1.683 -54.023  0.57 47.57           C
ATOM   2972  CG2 ILE A 238      -3.128   1.456 -55.135  0.57 45.13           C
ATOM   2973  CD1 ILE A 238      -0.427   0.272 -54.483  0.57 46.50           C
ATOM   2974  H   ILE A 238      -1.627   4.112 -55.286  0.57 38.27           H
ATOM   2975  HA  ILE A 238      -3.775   3.411 -53.469  0.57 40.87           H
ATOM   2976  HB  ILE A 238      -2.683   1.340 -53.060  0.57 47.23           H
ATOM   2977 HG12 ILE A 238      -0.423   2.405 -54.725  0.57 47.57           H
ATOM   2978 HG13 ILE A 238      -0.315   1.879 -53.076  0.57 47.57           H
ATOM   2979 HG21 ILE A 238      -3.013   0.384 -55.294  0.57 45.13           H
ATOM   2980 HG22 ILE A 238      -4.196   1.642 -55.014  0.57 45.13           H
ATOM   2981 HG23 ILE A 238      -2.814   1.959 -56.048  0.57 45.13           H
ATOM   2982 HD11 ILE A 238       0.612   0.059 -54.231  0.57 46.50           H
ATOM   2983 HD12 ILE A 238      -1.045  -0.490 -54.007  0.57 46.50           H
ATOM   2984 HD13 ILE A 238      -0.530   0.168 -55.564  0.57 46.50           H
ATOM   2985  N   PHE A 239      -0.898   4.446 -52.251  1.00 35.93           N
ATOM   2986  CA  PHE A 239      -0.277   4.879 -51.005  1.00 39.60           C
ATOM   2987  C   PHE A 239      -0.761   6.248 -50.553  1.00 39.94           C
ATOM   2988  O   PHE A 239      -0.740   6.539 -49.353  1.00 39.70           O
ATOM   2989  CB  PHE A 239       1.244   4.892 -51.145  1.00 45.10           C
ATOM   2990  CG  PHE A 239       1.878   3.549 -50.960  1.00 51.51           C
ATOM   2991  CD1 PHE A 239       2.095   3.041 -49.688  1.00 54.40           C
ATOM   2992  CD2 PHE A 239       2.259   2.793 -52.053  1.00 52.60           C
ATOM   2993  CE1 PHE A 239       2.678   1.803 -49.513  1.00 51.55           C
ATOM   2994  CE2 PHE A 239       2.844   1.557 -51.885  1.00 57.00           C
ATOM   2995  CZ  PHE A 239       3.053   1.060 -50.613  1.00 54.76           C
```

ATOM 2996 H PHE A 239 -0.403 4.631 -53.114 1.00 35.93 H
ATOM 2997 HA PHE A 239 -0.518 4.189 -50.193 1.00 39.60 H
ATOM 2998 HB2 PHE A 239 1.547 5.323 -52.099 1.00 45.10 H
ATOM 2999 HB3 PHE A 239 1.688 5.545 -50.391 1.00 45.10 H
ATOM 3000 HD1 PHE A 239 1.803 3.616 -48.821 1.00 54.40 H
ATOM 3001 HD2 PHE A 239 2.121 3.155 -53.055 1.00 52.60 H
ATOM 3002 HE1 PHE A 239 2.840 1.418 -48.517 1.00 51.55 H
ATOM 3003 HE2 PHE A 239 3.139 0.978 -52.748 1.00 57.00 H
ATOM 3004 HZ PHE A 239 3.510 0.091 -50.480 1.00 54.76 H
ATOM 3005 N ALA A 240 -1.185 7.102 -51.478 1.00 39.12 N
ATOM 3006 CA ALA A 240 -1.645 8.429 -51.102 1.00 41.39 C
ATOM 3007 C ALA A 240 -3.157 8.533 -51.005 1.00 40.28 C
ATOM 3008 O ALA A 240 -3.657 9.479 -50.389 1.00 43.92 O
ATOM 3009 CB ALA A 240 -1.152 9.475 -52.116 1.00 41.15 C
ATOM 3010 H ALA A 240 -1.179 6.870 -52.463 1.00 39.12 H
ATOM 3011 HA ALA A 240 -1.243 8.730 -50.133 1.00 41.39 H
ATOM 3012 HB1 ALA A 240 -1.351 10.482 -51.753 1.00 41.15 H
ATOM 3013 HB2 ALA A 240 -0.084 9.410 -52.305 1.00 41.15 H
ATOM 3014 HB3 ALA A 240 -1.663 9.389 -53.073 1.00 41.15 H
ATOM 3015 N PHE A 241 -3.899 7.589 -51.579 1.00 36.85 N
ATOM 3016 CA PHE A 241 -5.349 7.694 -51.526 1.00 37.48 C
ATOM 3017 C PHE A 241 -6.003 6.433 -50.973 1.00 36.07 C
ATOM 3018 O PHE A 241 -6.689 6.493 -49.949 1.00 38.51 O
ATOM 3019 CB PHE A 241 -5.917 8.019 -52.908 1.00 39.16 C
ATOM 3020 CG PHE A 241 -7.392 8.253 -52.898 1.00 37.29 C
ATOM 3021 CD1 PHE A 241 -7.914 9.420 -52.364 1.00 38.43 C
ATOM 3022 CD2 PHE A 241 -8.261 7.295 -53.389 1.00 47.36 C
ATOM 3023 CE1 PHE A 241 -9.273 9.632 -52.336 1.00 47.74 C
ATOM 3024 CE2 PHE A 241 -9.622 7.502 -53.365 1.00 45.98 C
ATOM 3025 CZ PHE A 241 -10.130 8.669 -52.840 1.00 45.33 C
ATOM 3026 H PHE A 241 -3.489 6.816 -52.084 1.00 36.85 H
ATOM 3027 HA PHE A 241 -5.691 8.486 -50.857 1.00 37.48 H
ATOM 3028 HB2 PHE A 241 -5.443 8.928 -53.270 1.00 39.16 H
ATOM 3029 HB3 PHE A 241 -5.709 7.267 -53.656 1.00 39.16 H
ATOM 3030 HD1 PHE A 241 -7.251 10.169 -51.956 1.00 38.43 H
ATOM 3031 HD2 PHE A 241 -7.861 6.394 -53.824 1.00 47.36 H
ATOM 3032 HE1 PHE A 241 -9.663 10.540 -51.900 1.00 47.74 H
ATOM 3033 HE2 PHE A 241 -10.290 6.747 -53.753 1.00 45.98 H
ATOM 3034 HZ PHE A 241 -11.200 8.788 -52.794 1.00 45.33 H
ATOM 3035 N ILE A 242 -5.796 5.290 -51.634 1.00 41.27 N

ATOM 3036 CA ILE A 242 -6.531 4.076 -51.276 1.00 42.31 C
ATOM 3037 C ILE A 242 -6.198 3.641 -49.852 1.00 41.45 C
ATOM 3038 O ILE A 242 -7.088 3.469 -49.013 1.00 46.71 O
ATOM 3039 CB ILE A 242 -6.242 2.948 -52.279 1.00 44.97 C
ATOM 3040 CG1 ILE A 242 -6.722 3.334 -53.681 1.00 52.29 C
ATOM 3041 CG2 ILE A 242 -6.896 1.658 -51.806 1.00 44.32 C
ATOM 3042 CD1 ILE A 242 -8.200 3.646 -53.763 1.00 53.12 C
ATOM 3043 H ILE A 242 -5.224 5.266 -52.468 1.00 41.27 H
ATOM 3044 HA ILE A 242 -7.595 4.308 -51.260 1.00 42.31 H
ATOM 3045 HB ILE A 242 -5.185 2.725 -52.277 1.00 44.97 H
ATOM 3046 HG12 ILE A 242 -6.168 4.194 -54.051 1.00 52.29 H
ATOM 3047 HG13 ILE A 242 -6.489 2.528 -54.378 1.00 52.29 H
ATOM 3048 HG21 ILE A 242 -6.861 0.901 -52.589 1.00 44.32 H
ATOM 3049 HG22 ILE A 242 -6.385 1.219 -50.949 1.00 44.32 H
ATOM 3050 HG23 ILE A 242 -7.943 1.799 -51.535 1.00 44.32 H
ATOM 3051 HD11 ILE A 242 -8.488 3.878 -54.785 1.00 53.12 H
ATOM 3052 HD12 ILE A 242 -8.803 2.787 -53.469 1.00 53.12 H
ATOM 3053 HD13 ILE A 242 -8.514 4.483 -53.148 1.00 53.12 H
ATOM 3054 N MET A 243 -4.912 3.424 -49.573 1.00 40.13 N
ATOM 3055 CA MET A 243 -4.505 3.021 -48.227 1.00 44.25 C
ATOM 3056 C MET A 243 -4.921 4.030 -47.164 1.00 43.89 C
ATOM 3057 O MET A 243 -5.448 3.608 -46.120 1.00 39.59 O
ATOM 3058 CB MET A 243 -2.993 2.773 -48.189 1.00 49.27 C
ATOM 3059 CG MET A 243 -2.449 2.433 -46.806 1.00 58.03 C
ATOM 3060 SD MET A 243 -0.653 2.584 -46.669 1.00 70.56 S
ATOM 3061 CE MET A 243 -0.450 4.363 -46.702 1.00 73.55 C
ATOM 3062 H MET A 243 -4.185 3.549 -50.268 1.00 40.13 H
ATOM 3063 HA MET A 243 -5.002 2.076 -47.996 1.00 44.25 H
ATOM 3064 HB2 MET A 243 -2.790 1.916 -48.833 1.00 49.27 H
ATOM 3065 HB3 MET A 243 -2.414 3.567 -48.640 1.00 49.27 H
ATOM 3066 HG2 MET A 243 -2.839 3.083 -46.030 1.00 58.03 H
ATOM 3067 HG3 MET A 243 -2.737 1.417 -46.534 1.00 58.03 H
ATOM 3068 HE1 MET A 243 0.611 4.611 -46.692 1.00 73.55 H
ATOM 3069 HE2 MET A 243 -0.894 4.790 -47.594 1.00 73.55 H
ATOM 3070 HE3 MET A 243 -0.920 4.818 -45.830 1.00 73.55 H
ATOM 3071 N PRO A 244 -4.716 5.341 -47.338 1.00 38.63 N
ATOM 3072 CA PRO A 244 -5.170 6.283 -46.300 1.00 38.57 C
ATOM 3073 C PRO A 244 -6.671 6.266 -46.069 1.00 36.85 C
ATOM 3074 O PRO A 244 -7.111 6.363 -44.917 1.00 38.08 O
ATOM 3075 CB PRO A 244 -4.690 7.637 -46.835 1.00 38.89 C

ATOM 3076 CG PRO A 244 -3.541 7.294 -47.709 1.00 37.15 C
ATOM 3077 CD PRO A 244 -3.901 6.020 -48.362 1.00 40.27 C
ATOM 3078 HA PRO A 244 -4.651 6.030 -45.376 1.00 38.57 H
ATOM 3079 HB2 PRO A 244 -5.462 8.101 -47.452 1.00 38.89 H
ATOM 3080 HB3 PRO A 244 -4.438 8.359 -46.063 1.00 38.89 H
ATOM 3081 HG2 PRO A 244 -3.293 8.076 -48.407 1.00 37.15 H
ATOM 3082 HG3 PRO A 244 -2.662 7.135 -47.082 1.00 37.15 H
ATOM 3083 HD2 PRO A 244 -4.388 6.180 -49.277 1.00 40.27 H
ATOM 3084 HD3 PRO A 244 -2.986 5.501 -48.603 1.00 40.27 H
ATOM 3085 N VAL A 245 -7.475 6.155 -47.129 1.00 34.21 N
ATOM 3086 CA VAL A 245 -8.923 6.085 -46.943 1.00 39.52 C
ATOM 3087 C VAL A 245 -9.295 4.834 -46.156 1.00 39.58 C
ATOM 3088 O VAL A 245 -10.163 4.873 -45.277 1.00 39.65 O
ATOM 3089 CB VAL A 245 -9.651 6.142 -48.300 1.00 42.28 C
ATOM 3090 CG1 VAL A 245 -11.121 5.780 -48.131 1.00 39.81 C
ATOM 3091 CG2 VAL A 245 -9.526 7.528 -48.910 1.00 44.96 C
ATOM 3092 H VAL A 245 -7.114 6.102 -48.073 1.00 34.21 H
ATOM 3093 HA VAL A 245 -9.237 6.953 -46.359 1.00 39.52 H
ATOM 3094 HB VAL A 245 -9.194 5.428 -48.987 1.00 42.28 H
ATOM 3095 HG11 VAL A 245 -11.673 5.990 -49.048 1.00 39.81 H
ATOM 3096 HG12 VAL A 245 -11.277 4.720 -47.927 1.00 39.81 H
ATOM 3097 HG13 VAL A 245 -11.592 6.358 -47.335 1.00 39.81 H
ATOM 3098 HG21 VAL A 245 -9.929 7.549 -49.923 1.00 44.96 H
ATOM 3099 HG22 VAL A 245 -10.071 8.269 -48.324 1.00 44.96 H
ATOM 3100 HG23 VAL A 245 -8.495 7.864 -48.964 1.00 44.96 H
ATOM 3101 N LEU A 246 -8.633 3.711 -46.442 1.00 34.41 N
ATOM 3102 CA LEU A 246 -8.915 2.488 -45.695 1.00 35.91 C
ATOM 3103 C LEU A 246 -8.481 2.614 -44.239 1.00 39.03 C
ATOM 3104 O LEU A 246 -9.201 2.179 -43.333 1.00 39.66 O
ATOM 3105 CB LEU A 246 -8.228 1.294 -46.357 1.00 37.16 C
ATOM 3106 CG LEU A 246 -8.816 0.843 -47.697 1.00 50.15 C
ATOM 3107 CD1 LEU A 246 -7.984 -0.276 -48.303 1.00 47.65 C
ATOM 3108 CD2 LEU A 246 -10.262 0.404 -47.520 1.00 48.16 C
ATOM 3109 H LEU A 246 -7.930 3.678 -47.169 1.00 34.41 H
ATOM 3110 HA LEU A 246 -9.992 2.317 -45.692 1.00 35.91 H
ATOM 3111 HB2 LEU A 246 -7.171 1.529 -46.491 1.00 37.16 H
ATOM 3112 HB3 LEU A 246 -8.250 0.441 -45.675 1.00 37.16 H
ATOM 3113 HG LEU A 246 -8.804 1.682 -48.392 1.00 50.15 H
ATOM 3114 HD11 LEU A 246 -7.696 -0.028 -49.324 1.00 47.65 H
ATOM 3115 HD12 LEU A 246 -7.061 -0.450 -47.749 1.00 47.65 H

ATOM 3116 HD13 LEU A 246 -8.507 -1.232 -48.340 1.00 47.65 H
ATOM 3117 HD21 LEU A 246 -10.536 -0.429 -48.168 1.00 48.16 H
ATOM 3118 HD22 LEU A 246 -10.483 0.100 -46.496 1.00 48.16 H
ATOM 3119 HD23 LEU A 246 -10.939 1.222 -47.768 1.00 48.16 H
ATOM 3120 N ILE A 247 -7.312 3.210 -43.994 1.00 34.52 N
ATOM 3121 CA ILE A 247 -6.798 3.305 -42.629 1.00 38.88 C
ATOM 3122 C ILE A 247 -7.724 4.153 -41.763 1.00 36.89 C
ATOM 3123 O ILE A 247 -8.126 3.741 -40.668 1.00 37.49 O
ATOM 3124 CB ILE A 247 -5.361 3.859 -42.628 1.00 39.10 C
ATOM 3125 CG1 ILE A 247 -4.383 2.814 -43.170 1.00 43.11 C
ATOM 3126 CG2 ILE A 247 -4.953 4.286 -41.223 1.00 38.28 C
ATOM 3127 CD1 ILE A 247 -2.954 3.313 -43.274 1.00 44.54 C
ATOM 3128 H ILE A 247 -6.727 3.552 -44.747 1.00 34.52 H
ATOM 3129 HA ILE A 247 -6.779 2.300 -42.201 1.00 38.88 H
ATOM 3130 HB ILE A 247 -5.316 4.736 -43.277 1.00 39.10 H
ATOM 3131 HG12 ILE A 247 -4.391 1.949 -42.506 1.00 43.11 H
ATOM 3132 HG13 ILE A 247 -4.701 2.424 -44.133 1.00 43.11 H
ATOM 3133 HG21 ILE A 247 -3.912 4.596 -41.173 1.00 38.28 H
ATOM 3134 HG22 ILE A 247 -5.503 5.154 -40.858 1.00 38.28 H
ATOM 3135 HG23 ILE A 247 -5.080 3.476 -40.504 1.00 38.28 H
ATOM 3136 HD11 ILE A 247 -2.353 2.630 -43.873 1.00 44.54 H
ATOM 3137 HD12 ILE A 247 -2.904 4.299 -43.737 1.00 44.54 H
ATOM 3138 HD13 ILE A 247 -2.469 3.367 -42.300 1.00 44.54 H
ATOM 3139 N ILE A 248 -8.086 5.345 -42.243 1.00 34.92 N
ATOM 3140 CA ILE A 248 -8.834 6.267 -41.392 1.00 35.13 C
ATOM 3141 C ILE A 248 -10.285 5.825 -41.231 1.00 39.95 C
ATOM 3142 O ILE A 248 -10.882 6.032 -40.168 1.00 40.16 O
ATOM 3143 CB ILE A 248 -8.739 7.710 -41.925 1.00 34.18 C
ATOM 3144 CG1 ILE A 248 -9.462 7.855 -43.265 1.00 41.24 C
ATOM 3145 CG2 ILE A 248 -7.275 8.142 -42.036 1.00 34.38 C
ATOM 3146 CD1 ILE A 248 -9.547 9.284 -43.766 1.00 42.90 C
ATOM 3147 H ILE A 248 -7.771 5.675 -43.147 1.00 34.92 H
ATOM 3148 HA ILE A 248 -8.381 6.253 -40.406 1.00 35.13 H
ATOM 3149 HB ILE A 248 -9.224 8.366 -41.200 1.00 34.18 H
ATOM 3150 HG12 ILE A 248 -9.070 7.206 -44.032 1.00 41.24 H
ATOM 3151 HG13 ILE A 248 -10.505 7.564 -43.171 1.00 41.24 H
ATOM 3152 HG21 ILE A 248 -7.189 9.195 -42.303 1.00 34.38 H
ATOM 3153 HG22 ILE A 248 -6.762 8.017 -41.085 1.00 34.38 H
ATOM 3154 HG23 ILE A 248 -6.714 7.575 -42.775 1.00 34.38 H
ATOM 3155 HD11 ILE A 248 -10.364 9.400 -44.479 1.00 42.90 H

ATOM 3156 HD12 ILE A 248 -9.716 9.991 -42.953 1.00 42.90 H
ATOM 3157 HD13 ILE A 248 -8.629 9.575 -44.278 1.00 42.90 H
ATOM 3158 N THR A 249 -10.882 5.211 -42.255 1.00 33.10 N
ATOM 3159 CA THR A 249 -12.263 4.761 -42.103 1.00 37.62 C
ATOM 3160 C THR A 249 -12.352 3.561 -41.164 1.00 37.73 C
ATOM 3161 O THR A 249 -13.276 3.474 -40.347 1.00 39.51 O
ATOM 3162 CB THR A 249 -12.882 4.431 -43.464 1.00 39.02 C
ATOM 3163 CG2 THR A 249 -13.026 5.695 -44.310 1.00 39.43 C
ATOM 3164 OG1 THR A 249 -12.066 3.477 -44.155 1.00 46.06 O
ATOM 3165 H THR A 249 -10.406 5.032 -43.128 1.00 33.10 H
ATOM 3166 HA THR A 249 -12.858 5.566 -41.665 1.00 37.62 H
ATOM 3167 HB THR A 249 -13.869 3.985 -43.330 1.00 39.02 H
ATOM 3168 HG1 THR A 249 -11.334 3.947 -44.574 1.00 46.06 H
ATOM 3169 HG21 THR A 249 -13.387 5.457 -45.311 1.00 39.43 H
ATOM 3170 HG22 THR A 249 -13.752 6.368 -43.855 1.00 39.43 H
ATOM 3171 HG23 THR A 249 -12.097 6.251 -44.418 1.00 39.43 H
ATOM 3172 N VAL A 250 -11.397 2.634 -41.252 1.00 39.37 N
ATOM 3173 CA VAL A 250 -11.436 1.460 -40.383 1.00 38.80 C
ATOM 3174 C VAL A 250 -11.073 1.838 -38.952 1.00 39.21 C
ATOM 3175 O VAL A 250 -11.734 1.406 -38.000 1.00 39.16 O
ATOM 3176 CB VAL A 250 -10.518 0.352 -40.930 1.00 41.51 C
ATOM 3177 CG1 VAL A 250 -10.369 -0.772 -39.911 1.00 42.18 C
ATOM 3178 CG2 VAL A 250 -11.074 -0.193 -42.236 1.00 43.79 C
ATOM 3179 H VAL A 250 -10.653 2.698 -41.935 1.00 39.37 H
ATOM 3180 HA VAL A 250 -12.454 1.064 -40.367 1.00 38.80 H
ATOM 3181 HB VAL A 250 -9.527 0.764 -41.125 1.00 41.51 H
ATOM 3182 HG11 VAL A 250 -9.859 -1.627 -40.356 1.00 42.18 H
ATOM 3183 HG12 VAL A 250 -9.769 -0.484 -39.047 1.00 42.18 H
ATOM 3184 HG13 VAL A 250 -11.338 -1.124 -39.555 1.00 42.18 H
ATOM 3185 HG21 VAL A 250 -10.399 -0.931 -42.670 1.00 43.79 H
ATOM 3186 HG22 VAL A 250 -12.039 -0.678 -42.084 1.00 43.79 H
ATOM 3187 HG23 VAL A 250 -11.219 0.586 -42.983 1.00 43.79 H
ATOM 3188 N CYS A 251 -10.028 2.648 -38.772 1.00 29.75 N
ATOM 3189 CA CYS A 251 -9.617 3.031 -37.423 1.00 33.57 C
ATOM 3190 C CYS A 251 -10.692 3.862 -36.735 1.00 37.57 C
ATOM 3191 O CYS A 251 -11.067 3.581 -35.591 1.00 37.09 O
ATOM 3192 CB CYS A 251 -8.288 3.789 -37.469 1.00 28.43 C
ATOM 3193 SG CYS A 251 -6.864 2.730 -37.803 1.00 41.82 S
ATOM 3194 H CYS A 251 -9.486 3.000 -39.552 1.00 29.75 H
ATOM 3195 HA CYS A 251 -9.470 2.127 -36.827 1.00 33.57 H

ATOM 3196 HB2 CYS A 251 -8.329 4.551 -38.244 1.00 28.43 H
ATOM 3197 HB3 CYS A 251 -8.088 4.332 -36.549 1.00 28.43 H
ATOM 3198 HG CYS A 251 -7.177 2.487 -39.079 1.00 41.82 H
ATOM 3199 N TYR A 252 -11.209 4.885 -37.421 1.00 33.61 N
ATOM 3200 CA TYR A 252 -12.259 5.708 -36.829 1.00 34.00 C
ATOM 3201 C TYR A 252 -13.556 4.925 -36.672 1.00 42.08 C
ATOM 3202 O TYR A 252 -14.289 5.117 -35.694 1.00 37.40 O
ATOM 3203 CB TYR A 252 -12.486 6.959 -37.674 1.00 30.89 C
ATOM 3204 CG TYR A 252 -11.427 8.023 -37.495 1.00 37.96 C
ATOM 3205 CD1 TYR A 252 -10.905 8.308 -36.238 1.00 37.64 C
ATOM 3206 CD2 TYR A 252 -10.950 8.745 -38.583 1.00 33.81 C
ATOM 3207 CE1 TYR A 252 -9.938 9.283 -36.070 1.00 39.15 C
ATOM 3208 CE2 TYR A 252 -9.982 9.719 -38.426 1.00 38.63 C
ATOM 3209 CZ TYR A 252 -9.482 9.985 -37.169 1.00 40.56 C
ATOM 3210 OH TYR A 252 -8.519 10.954 -37.009 1.00 36.30 O
ATOM 3211 H TYR A 252 -10.903 5.110 -38.359 1.00 33.61 H
ATOM 3212 HA TYR A 252 -11.962 5.965 -35.817 1.00 34.00 H
ATOM 3213 HB2 TYR A 252 -12.692 6.731 -38.718 1.00 30.89 H
ATOM 3214 HB3 TYR A 252 -13.390 7.435 -37.303 1.00 30.89 H
ATOM 3215 HD1 TYR A 252 -11.251 7.809 -35.349 1.00 37.64 H
ATOM 3216 HD2 TYR A 252 -11.343 8.553 -39.571 1.00 33.81 H
ATOM 3217 HE1 TYR A 252 -9.548 9.483 -35.085 1.00 39.15 H
ATOM 3218 HE2 TYR A 252 -9.627 10.267 -39.287 1.00 38.63 H
ATOM 3219 HH TYR A 252 -8.240 11.340 -37.844 1.00 36.30 H
ATOM 3220 N GLY A 253 -13.857 4.042 -37.626 1.00 34.67 N
ATOM 3221 CA GLY A 253 -15.051 3.221 -37.506 1.00 38.78 C
ATOM 3222 C GLY A 253 -14.990 2.284 -36.315 1.00 39.48 C
ATOM 3223 O GLY A 253 -15.953 2.160 -35.557 1.00 39.96 O
ATOM 3224 H GLY A 253 -13.264 3.910 -38.435 1.00 34.67 H
ATOM 3225 HA2 GLY A 253 -15.939 3.853 -37.442 1.00 38.78 H
ATOM 3226 HA3 GLY A 253 -15.156 2.624 -38.412 1.00 38.78 H
ATOM 3227 N LEU A 254 -13.853 1.607 -36.137 1.00 36.56 N
ATOM 3228 CA LEU A 254 -13.692 0.735 -34.979 1.00 40.39 C
ATOM 3229 C LEU A 254 -13.660 1.532 -33.681 1.00 43.46 C
ATOM 3230 O LEU A 254 -14.106 1.037 -32.640 1.00 39.76 O
ATOM 3231 CB LEU A 254 -12.422 -0.101 -35.122 1.00 32.00 C
ATOM 3232 CG LEU A 254 -12.453 -1.159 -36.229 1.00 41.11 C
ATOM 3233 CD1 LEU A 254 -11.169 -1.976 -36.235 1.00 42.60 C
ATOM 3234 CD2 LEU A 254 -13.672 -2.057 -36.072 1.00 41.99 C
ATOM 3235 H LEU A 254 -13.074 1.693 -36.779 1.00 36.56 H

ATOM 3236 HA LEU A 254 -14.560 0.079 -34.911 1.00 40.39 H
ATOM 3237 HB2 LEU A 254 -11.568 0.561 -35.276 1.00 32.00 H
ATOM 3238 HB3 LEU A 254 -12.236 -0.614 -34.177 1.00 32.00 H
ATOM 3239 HG LEU A 254 -12.548 -0.684 -37.201 1.00 41.11 H
ATOM 3240 HD11 LEU A 254 -10.779 -2.073 -37.248 1.00 42.60 H
ATOM 3241 HD12 LEU A 254 -10.381 -1.515 -35.643 1.00 42.60 H
ATOM 3242 HD13 LEU A 254 -11.309 -2.984 -35.844 1.00 42.60 H
ATOM 3243 HD21 LEU A 254 -13.487 -3.068 -36.437 1.00 41.99 H
ATOM 3244 HD22 LEU A 254 -13.992 -2.148 -35.033 1.00 41.99 H
ATOM 3245 HD23 LEU A 254 -14.514 -1.670 -36.647 1.00 41.99 H
ATOM 3246 N MET A 255 -13.138 2.760 -33.727 1.00 35.93 N
ATOM 3247 CA MET A 255 -13.140 3.616 -32.546 1.00 38.38 C
ATOM 3248 C MET A 255 -14.564 3.970 -32.131 1.00 42.89 C
ATOM 3249 O MET A 255 -14.911 3.906 -30.945 1.00 42.02 O
ATOM 3250 CB MET A 255 -12.322 4.877 -32.831 1.00 37.59 C
ATOM 3251 CG MET A 255 -12.210 5.852 -31.674 1.00 45.32 C
ATOM 3252 SD MET A 255 -11.495 7.429 -32.185 1.00 43.07 S
ATOM 3253 CE MET A 255 -12.892 8.181 -33.019 1.00 38.56 C
ATOM 3254 H MET A 255 -12.742 3.131 -34.581 1.00 35.93 H
ATOM 3255 HA MET A 255 -12.664 3.082 -31.721 1.00 38.38 H
ATOM 3256 HB2 MET A 255 -11.313 4.582 -33.121 1.00 37.59 H
ATOM 3257 HB3 MET A 255 -12.734 5.390 -33.695 1.00 37.59 H
ATOM 3258 HG2 MET A 255 -13.164 6.054 -31.192 1.00 45.32 H
ATOM 3259 HG3 MET A 255 -11.554 5.420 -30.928 1.00 45.32 H
ATOM 3260 HE1 MET A 255 -12.644 9.197 -33.327 1.00 38.56 H
ATOM 3261 HE2 MET A 255 -13.174 7.616 -33.907 1.00 38.56 H
ATOM 3262 HE3 MET A 255 -13.753 8.222 -32.352 1.00 38.56 H
ATOM 3263 N ILE A 256 -15.405 4.334 -33.100 1.00 37.56 N
ATOM 3264 CA ILE A 256 -16.792 4.677 -32.803 1.00 44.45 C
ATOM 3265 C ILE A 256 -17.558 3.450 -32.320 1.00 48.12 C
ATOM 3266 O ILE A 256 -18.368 3.538 -31.390 1.00 44.25 O
ATOM 3267 CB ILE A 256 -17.452 5.318 -34.037 1.00 44.45 C
ATOM 3268 CG1 ILE A 256 -16.907 6.730 -34.247 1.00 47.29 C
ATOM 3269 CG2 ILE A 256 -18.969 5.334 -33.899 1.00 44.34 C
ATOM 3270 CD1 ILE A 256 -17.409 7.392 -35.512 1.00 58.01 C
ATOM 3271 H ILE A 256 -15.100 4.393 -34.064 1.00 37.56 H
ATOM 3272 HA ILE A 256 -16.802 5.408 -31.991 1.00 44.45 H
ATOM 3273 HB ILE A 256 -17.201 4.724 -34.918 1.00 44.45 H
ATOM 3274 HG12 ILE A 256 -17.177 7.357 -33.395 1.00 47.29 H
ATOM 3275 HG13 ILE A 256 -15.818 6.723 -34.270 1.00 47.29 H

ATOM 3276 HG21 ILE A 256 -19.451 5.836 -34.736 1.00 44.34 H
ATOM 3277 HG22 ILE A 256 -19.413 4.338 -33.899 1.00 44.34 H
ATOM 3278 HG23 ILE A 256 -19.284 5.849 -32.990 1.00 44.34 H
ATOM 3279 HD11 ILE A 256 -16.896 8.327 -35.693 1.00 58.01 H
ATOM 3280 HD12 ILE A 256 -17.259 6.755 -36.384 1.00 58.01 H
ATOM 3281 HD13 ILE A 256 -18.466 7.629 -35.441 1.00 58.01 H
ATOM 3282 N LEU A 257 -17.306 2.288 -32.930 1.00 40.65 N
ATOM 3283 CA LEU A 257 -17.991 1.069 -32.508 1.00 50.46 C
ATOM 3284 C LEU A 257 -17.675 0.728 -31.057 1.00 39.24 C
ATOM 3285 O LEU A 257 -18.563 0.311 -30.304 1.00 47.40 O
ATOM 3286 CB LEU A 257 -17.615 -0.093 -33.429 1.00 53.32 C
ATOM 3287 CG LEU A 257 -18.181 -0.034 -34.850 1.00 64.57 C
ATOM 3288 CD1 LEU A 257 -17.673 -1.203 -35.685 1.00 67.76 C
ATOM 3289 CD2 LEU A 257 -19.702 -0.008 -34.824 1.00 62.78 C
ATOM 3290 H LEU A 257 -16.654 2.226 -33.702 1.00 40.65 H
ATOM 3291 HA LEU A 257 -19.066 1.243 -32.565 1.00 50.46 H
ATOM 3292 HB2 LEU A 257 -16.527 -0.164 -33.479 1.00 53.32 H
ATOM 3293 HB3 LEU A 257 -17.945 -1.030 -32.976 1.00 53.32 H
ATOM 3294 HG LEU A 257 -17.888 0.885 -35.341 1.00 64.57 H
ATOM 3295 HD11 LEU A 257 -17.113 -0.840 -36.547 1.00 67.76 H
ATOM 3296 HD12 LEU A 257 -16.999 -1.850 -35.122 1.00 67.76 H
ATOM 3297 HD13 LEU A 257 -18.472 -1.840 -36.065 1.00 67.76 H
ATOM 3298 HD21 LEU A 257 -20.141 -0.407 -35.739 1.00 62.78 H
ATOM 3299 HD22 LEU A 257 -20.093 -0.594 -33.994 1.00 62.78 H
ATOM 3300 HD23 LEU A 257 -20.072 1.013 -34.720 1.00 62.78 H
ATOM 3301 N ARG A 258 -16.414 0.890 -30.647 0.65 45.62 N
ATOM 3302 CA ARG A 258 -16.061 0.639 -29.252 0.65 46.59 C
ATOM 3303 C ARG A 258 -16.746 1.636 -28.327 0.65 47.31 C
ATOM 3304 O ARG A 258 -17.241 1.262 -27.256 0.65 52.72 O
ATOM 3305 CB ARG A 258 -14.542 0.694 -29.072 0.65 43.30 C
ATOM 3306 CG ARG A 258 -14.073 0.510 -27.631 0.65 42.45 C
ATOM 3307 CD ARG A 258 -14.350 -0.902 -27.123 0.65 46.50 C
ATOM 3308 NE ARG A 258 -13.640 -1.910 -27.907 0.65 48.61 N
ATOM 3309 CZ ARG A 258 -12.408 -2.333 -27.642 0.65 48.89 C
ATOM 3310 NH1 ARG A 258 -11.741 -1.838 -26.607 0.65 49.73 N1+
ATOM 3311 NH2 ARG A 258 -11.842 -3.251 -28.413 0.65 49.03 N1+
ATOM 3312 H ARG A 258 -15.689 1.211 -31.275 0.65 45.62 H
ATOM 3313 HA ARG A 258 -16.415 -0.364 -29.011 0.65 46.59 H
ATOM 3314 HB2 ARG A 258 -14.077 -0.047 -29.723 0.65 43.30 H
ATOM 3315 HB3 ARG A 258 -14.177 1.657 -29.432 0.65 43.30 H

ATOM 3316 HG2 ARG A 258 -12.989 0.625 -27.663 0.65 42.45 H
ATOM 3317 HG3 ARG A 258 -14.417 1.272 -26.932 0.65 42.45 H
ATOM 3318 HD2 ARG A 258 -14.228 -1.000 -26.044 0.65 46.50 H
ATOM 3319 HD3 ARG A 258 -15.398 -1.139 -27.292 0.65 46.50 H
ATOM 3320 HE ARG A 258 -14.122 -2.271 -28.718 0.65 48.61 H
ATOM 3321 HH11 ARG A 258 -12.163 -1.132 -26.022 0.65 49.73 H
ATOM 3322 HH12 ARG A 258 -10.796 -2.140 -26.414 0.65 49.73 H
ATOM 3323 HH21 ARG A 258 -12.339 -3.632 -29.206 0.65 49.03 H
ATOM 3324 HH22 ARG A 258 -10.904 -3.574 -28.217 0.65 49.03 H
ATOM 3325 N LEU A 259 -16.794 2.909 -28.727 1.00 42.90 N
ATOM 3326 CA LEU A 259 -17.423 3.922 -27.887 1.00 51.48 C
ATOM 3327 C LEU A 259 -18.933 3.736 -27.801 1.00 58.39 C
ATOM 3328 O LEU A 259 -19.540 4.099 -26.788 1.00 57.30 O
ATOM 3329 CB LEU A 259 -17.089 5.320 -28.409 1.00 46.17 C
ATOM 3330 CG LEU A 259 -15.641 5.769 -28.207 1.00 43.80 C
ATOM 3331 CD1 LEU A 259 -15.375 7.083 -28.926 1.00 41.89 C
ATOM 3332 CD2 LEU A 259 -15.329 5.894 -26.720 1.00 38.49 C
ATOM 3333 H LEU A 259 -16.380 3.206 -29.601 1.00 42.90 H
ATOM 3334 HA LEU A 259 -17.037 3.825 -26.870 1.00 51.48 H
ATOM 3335 HB2 LEU A 259 -17.334 5.357 -29.472 1.00 46.17 H
ATOM 3336 HB3 LEU A 259 -17.741 6.056 -27.934 1.00 46.17 H
ATOM 3337 HG LEU A 259 -14.993 5.014 -28.639 1.00 43.80 H
ATOM 3338 HD11 LEU A 259 -14.497 7.000 -29.565 1.00 41.89 H
ATOM 3339 HD12 LEU A 259 -16.205 7.381 -29.568 1.00 41.89 H
ATOM 3340 HD13 LEU A 259 -15.197 7.916 -28.247 1.00 41.89 H
ATOM 3341 HD21 LEU A 259 -14.674 6.735 -26.490 1.00 38.49 H
ATOM 3342 HD22 LEU A 259 -16.227 6.012 -26.113 1.00 38.49 H
ATOM 3343 HD23 LEU A 259 -14.815 5.000 -26.366 1.00 38.49 H
ATOM 3344 N LYS A 260 -19.559 3.183 -28.841 1.00 45.85 N
ATOM 3345 CA LYS A 260 -20.995 2.944 -28.786 1.00 53.40 C
ATOM 3346 C LYS A 260 -21.353 1.681 -28.012 1.00 56.66 C
ATOM 3347 O LYS A 260 -22.532 1.480 -27.698 1.00 58.41 O
ATOM 3348 CB LYS A 260 -21.582 2.877 -30.203 1.00 65.77 C
ATOM 3349 CG LYS A 260 -21.754 1.475 -30.770 1.00 76.08 C
ATOM 3350 CD LYS A 260 -22.435 1.523 -32.133 1.00 85.40 C
ATOM 3351 CE LYS A 260 -22.787 0.133 -32.643 1.00 91.86 C
ATOM 3352 NZ LYS A 260 -23.407 0.172 -33.999 1.00 94.60 N1+
ATOM 3353 H LYS A 260 -19.062 2.932 -29.687 1.00 45.85 H
ATOM 3354 HA LYS A 260 -21.511 3.743 -28.267 1.00 53.40 H
ATOM 3355 HB2 LYS A 260 -22.579 3.318 -30.154 1.00 65.77 H

ATOM 3356 HB3 LYS A 260 -21.024 3.518 -30.887 1.00 65.77 H
ATOM 3357 HG2 LYS A 260 -20.800 0.971 -30.862 1.00 76.08 H
ATOM 3358 HG3 LYS A 260 -22.396 0.842 -30.159 1.00 76.08 H
ATOM 3359 HD2 LYS A 260 -23.339 2.131 -32.079 1.00 85.40 H
ATOM 3360 HD3 LYS A 260 -21.773 2.017 -32.846 1.00 85.40 H
ATOM 3361 HE2 LYS A 260 -21.900 -0.498 -32.675 1.00 91.86 H
ATOM 3362 HE3 LYS A 260 -23.489 -0.349 -31.961 1.00 91.86 H
ATOM 3363 HZ1 LYS A 260 -22.760 0.586 -34.656 1.00 94.60 H
ATOM 3364 HZ2 LYS A 260 -23.630 -0.767 -34.296 1.00 94.60 H
ATOM 3365 HZ3 LYS A 260 -24.253 0.723 -33.969 1.00 94.60 H
ATOM 3366 N SER A 261 -20.371 0.845 -27.676 1.00 54.91 N
ATOM 3367 CA SER A 261 -20.635 -0.396 -26.961 1.00 60.10 C
ATOM 3368 C SER A 261 -20.632 -0.234 -25.448 1.00 63.52 C
ATOM 3369 O SER A 261 -21.109 -1.132 -24.747 1.00 69.84 O
ATOM 3370 CB SER A 261 -19.604 -1.459 -27.351 1.00 59.03 C
ATOM 3371 OG SER A 261 -18.314 -1.104 -26.881 1.00 60.20 O
ATOM 3372 H SER A 261 -19.416 1.040 -27.943 1.00 54.91 H
ATOM 3373 HA SER A 261 -21.612 -0.789 -27.248 1.00 60.10 H
ATOM 3374 HB2 SER A 261 -19.871 -2.430 -26.933 1.00 59.03 H
ATOM 3375 HB3 SER A 261 -19.569 -1.583 -28.434 1.00 59.03 H
ATOM 3376 HG SER A 261 -18.064 -0.242 -27.232 1.00 60.20 H
ATOM 3377 N VAL A 262 -20.108 0.874 -24.924 1.00 64.66 N
ATOM 3378 CA VAL A 262 -20.054 1.058 -23.479 1.00 69.71 C
ATOM 3379 C VAL A 262 -21.449 1.371 -22.953 1.00 78.62 C
ATOM 3380 O VAL A 262 -22.229 2.091 -23.590 1.00 75.83 O
ATOM 3381 CB VAL A 262 -19.039 2.156 -23.113 1.00 72.15 C
ATOM 3382 CG1 VAL A 262 -19.406 3.473 -23.753 1.00 71.18 C
ATOM 3383 CG2 VAL A 262 -18.939 2.310 -21.602 1.00 72.08 C
ATOM 3384 H VAL A 262 -19.719 1.600 -25.508 1.00 64.66 H
ATOM 3385 HA VAL A 262 -19.698 0.128 -23.028 1.00 69.71 H
ATOM 3386 HB VAL A 262 -18.067 1.845 -23.499 1.00 72.15 H
ATOM 3387 HG11 VAL A 262 -18.538 4.125 -23.856 1.00 71.18 H
ATOM 3388 HG12 VAL A 262 -19.821 3.299 -24.722 1.00 71.18 H
ATOM 3389 HG13 VAL A 262 -20.159 4.012 -23.177 1.00 71.18 H
ATOM 3390 HG21 VAL A 262 -18.132 2.996 -21.342 1.00 72.08 H
ATOM 3391 HG22 VAL A 262 -19.848 2.709 -21.151 1.00 72.08 H
ATOM 3392 HG23 VAL A 262 -18.718 1.357 -21.120 1.00 72.08 H
ATOM 3393 N ARG A 263 -21.763 0.750 -21.842 1.00 88.59 N
ATOM 3394 CA ARG A 263 -23.011 0.921 -21.181 1.00 98.61 C
ATOM 3395 C ARG A 263 -22.588 1.393 -19.866 1.00103.24 C

ATOM 3396 O ARG A 263 -21.887 0.716 -19.170 1.00106.40 O
ATOM 3397 CB ARG A 263 -23.671 -0.416 -20.997 1.00103.46 C
ATOM 3398 CG ARG A 263 -24.047 -1.077 -22.294 1.00108.12 C
ATOM 3399 CD ARG A 263 -25.009 -0.216 -23.092 1.00114.04 C
ATOM 3400 NE ARG A 263 -24.895 -0.506 -24.512 1.00116.98 N
ATOM 3401 CZ ARG A 263 -25.294 0.306 -25.475 1.00116.62 C
ATOM 3402 NH1 ARG A 263 -25.857 1.470 -25.179 1.00116.97 N1+
ATOM 3403 NH2 ARG A 263 -25.133 -0.053 -26.733 1.00115.34 N1+
ATOM 3404 H ARG A 263 -21.089 0.158 -21.378 1.00 88.59 H
ATOM 3405 HA ARG A 263 -23.652 1.684 -21.620 1.00 98.61 H
ATOM 3406 HB2 ARG A 263 -23.036 -1.115 -20.449 1.00103.46 H
ATOM 3407 HB3 ARG A 263 -24.574 -0.293 -20.395 1.00103.46 H
ATOM 3408 HG2 ARG A 263 -23.166 -1.343 -22.880 1.00108.12 H
ATOM 3409 HG3 ARG A 263 -24.540 -2.018 -22.047 1.00108.12 H
ATOM 3410 HD2 ARG A 263 -26.028 -0.514 -22.845 1.00114.04 H
ATOM 3411 HD3 ARG A 263 -24.969 0.846 -22.887 1.00114.04 H
ATOM 3412 HE ARG A 263 -24.502 -1.405 -24.755 1.00116.98 H
ATOM 3413 HH11 ARG A 263 -26.002 1.726 -24.213 1.00116.97 H
ATOM 3414 HH12 ARG A 263 -26.228 2.062 -25.908 1.00116.97 H
ATOM 3415 HH21 ARG A 263 -24.727 -0.951 -26.958 1.00115.34 H
ATOM 3416 HH22 ARG A 263 -25.409 0.563 -27.484 1.00115.34 H
ATOM 3417 N MET A 264 -22.976 2.590 -19.526 1.00107.41 N
ATOM 3418 CA MET A 264 -22.628 3.076 -18.230 1.00114.28 C
ATOM 3419 C MET A 264 -23.934 3.135 -17.475 1.00113.36 C
ATOM 3420 O MET A 264 -24.834 3.845 -17.864 1.00113.15 O
ATOM 3421 CB MET A 264 -21.983 4.448 -18.338 1.00121.50 C
ATOM 3422 CG MET A 264 -21.978 5.016 -19.747 1.00125.45 C
ATOM 3423 SD MET A 264 -23.574 4.681 -20.488 1.00131.50 S
ATOM 3424 CE MET A 264 -24.577 5.893 -19.647 1.00137.27 C
ATOM 3425 H MET A 264 -23.517 3.201 -20.127 1.00107.41 H
ATOM 3426 HA MET A 264 -21.937 2.459 -17.651 1.00114.28 H
ATOM 3427 HB2 MET A 264 -22.407 5.167 -17.635 1.00121.50 H
ATOM 3428 HB3 MET A 264 -20.942 4.355 -18.027 1.00121.50 H
ATOM 3429 HG2 MET A 264 -21.780 6.088 -19.744 1.00125.45 H
ATOM 3430 HG3 MET A 264 -21.202 4.547 -20.352 1.00125.45 H
ATOM 3431 HE1 MET A 264 -25.602 5.593 -19.608 1.00137.27 H
ATOM 3432 HE2 MET A 264 -24.220 6.080 -18.638 1.00137.27 H
ATOM 3433 HE3 MET A 264 -24.253 6.714 -20.259 1.00137.27 H
ATOM 3434 N LEU A 265 -24.012 2.399 -16.379 1.00114.26 N
ATOM 3435 CA LEU A 265 -25.219 2.324 -15.562 1.00115.58 C

ATOM 3436 C LEU A 265 -25.952 3.617 -15.265 1.00115.06 C
ATOM 3437 O LEU A 265 -27.097 3.577 -14.878 1.00114.43 O
ATOM 3438 CB LEU A 265 -24.943 1.612 -14.236 1.00115.68 C
ATOM 3439 CG LEU A 265 -25.208 0.120 -14.342 1.00115.15 C
ATOM 3440 CD1 LEU A 265 -23.996 -0.620 -14.863 1.00112.15 C
ATOM 3441 CD2 LEU A 265 -25.816 -0.516 -13.099 1.00119.72 C
ATOM 3442 H LEU A 265 -23.232 1.826 -16.092 1.00114.26 H
ATOM 3443 HA LEU A 265 -25.934 1.760 -16.162 1.00115.58 H
ATOM 3444 HB2 LEU A 265 -23.939 1.821 -13.863 1.00115.68 H
ATOM 3445 HB3 LEU A 265 -25.600 1.999 -13.455 1.00115.68 H
ATOM 3446 HG LEU A 265 -25.994 -0.027 -15.084 1.00115.15 H
ATOM 3447 HD11 LEU A 265 -23.541 -1.247 -14.096 1.00112.15 H
ATOM 3448 HD12 LEU A 265 -24.270 -1.275 -15.691 1.00112.15 H
ATOM 3449 HD13 LEU A 265 -23.214 0.045 -15.229 1.00112.15 H
ATOM 3450 HD21 LEU A 265 -26.393 -1.404 -13.359 1.00119.72 H
ATOM 3451 HD22 LEU A 265 -25.049 -0.820 -12.386 1.00119.72 H
ATOM 3452 HD23 LEU A 265 -26.493 0.161 -12.578 1.00119.72 H
ATOM 3453 N SER A 266 -25.354 4.757 -15.529 1.00114.06 N
ATOM 3454 CA SER A 266 -26.043 5.981 -15.133 1.00116.49 C
ATOM 3455 C SER A 266 -26.303 6.957 -16.279 1.00117.91 C
ATOM 3456 O SER A 266 -27.440 7.083 -16.751 1.00127.10 O
ATOM 3457 CB SER A 266 -25.253 6.694 -14.038 1.00111.09 C
ATOM 3458 OG SER A 266 -25.894 7.900 -13.667 1.00114.21 O
ATOM 3459 H SER A 266 -24.434 4.810 -15.939 1.00114.06 H
ATOM 3460 HA SER A 266 -27.033 5.835 -14.697 1.00116.49 H
ATOM 3461 HB2 SER A 266 -25.187 6.059 -13.154 1.00111.09 H
ATOM 3462 HB3 SER A 266 -24.228 6.905 -14.345 1.00111.09 H
ATOM 3463 HG SER A 266 -25.383 8.319 -12.970 1.00114.21 H
ATOM 3464 N GLY A 267 -25.269 7.685 -16.706 1.00110.19 N
ATOM 3465 CA GLY A 267 -25.465 8.844 -17.560 1.00108.81 C
ATOM 3466 C GLY A 267 -25.465 8.560 -19.047 1.00109.56 C
ATOM 3467 O GLY A 267 -24.526 8.944 -19.754 1.00106.95 O
ATOM 3468 H GLY A 267 -24.351 7.548 -16.310 1.00110.19 H
ATOM 3469 HA2 GLY A 267 -26.379 9.381 -17.296 1.00108.81 H
ATOM 3470 HA3 GLY A 267 -24.653 9.540 -17.347 1.00108.81 H
ATOM 3471 N SER A 268 -26.527 7.911 -19.537 1.00117.85 N
ATOM 3472 CA SER A 268 -26.609 7.581 -20.956 1.00117.10 C
ATOM 3473 C SER A 268 -26.714 8.826 -21.828 1.00110.17 C
ATOM 3474 O SER A 268 -26.297 8.798 -22.993 1.00110.31 O
ATOM 3475 CB SER A 268 -27.799 6.660 -21.213 1.00124.17 C

ATOM 3476 OG SER A 268 -27.548 5.350 -20.735 1.00128.64 O
ATOM 3477 H SER A 268 -27.266 7.583 -18.929 1.00117.85 H
ATOM 3478 HA SER A 268 -25.767 7.083 -21.413 1.00117.10 H
ATOM 3479 HB2 SER A 268 -28.705 7.047 -20.744 1.00124.17 H
ATOM 3480 HB3 SER A 268 -28.007 6.581 -22.282 1.00124.17 H
ATOM 3481 HG SER A 268 -28.332 4.815 -20.881 1.00128.64 H
ATOM 3482 N LYS A 269 -27.274 9.914 -21.295 1.00106.08 N
ATOM 3483 CA LYS A 269 -27.382 11.145 -22.070 1.00102.33 C
ATOM 3484 C LYS A 269 -26.012 11.758 -22.328 1.00102.78 C
ATOM 3485 O LYS A 269 -25.737 12.234 -23.437 1.00103.12 O
ATOM 3486 CB LYS A 269 -28.289 12.138 -21.349 1.00 0.00 C
ATOM 3487 CG LYS A 269 -28.655 13.356 -22.203 1.00 0.00 C
ATOM 3488 CD LYS A 269 -29.524 14.396 -21.485 1.00 0.00 C
ATOM 3489 CE LYS A 269 -29.902 15.624 -22.325 1.00 0.00 C
ATOM 3490 NZ LYS A 269 -30.736 16.591 -21.590 1.00 0.00 N1+
ATOM 3491 H LYS A 269 -27.618 9.916 -20.346 1.00106.08 H
ATOM 3492 HA LYS A 269 -27.838 10.909 -23.035 1.00102.33 H
ATOM 3493 HB2 LYS A 269 -29.216 11.634 -21.071 1.00 0.00 H
ATOM 3494 HB3 LYS A 269 -27.830 12.459 -20.412 1.00 0.00 H
ATOM 3495 HG2 LYS A 269 -27.753 13.861 -22.548 1.00 0.00 H
ATOM 3496 HG3 LYS A 269 -29.171 13.018 -23.102 1.00 0.00 H
ATOM 3497 HD2 LYS A 269 -30.437 13.910 -21.138 1.00 0.00 H
ATOM 3498 HD3 LYS A 269 -29.004 14.727 -20.586 1.00 0.00 H
ATOM 3499 HE2 LYS A 269 -28.999 16.135 -22.663 1.00 0.00 H
ATOM 3500 HE3 LYS A 269 -30.443 15.311 -23.218 1.00 0.00 H
ATOM 3501 HZ1 LYS A 269 -30.237 16.919 -20.775 1.00 0.00 H
ATOM 3502 HZ2 LYS A 269 -30.954 17.375 -22.188 1.00 0.00 H
ATOM 3503 HZ3 LYS A 269 -31.594 16.144 -21.298 1.00 0.00 H
ATOM 3504 N GLU A 270 -25.140 11.757 -21.315 1.00103.76 N
ATOM 3505 CA GLU A 270 -23.790 12.279 -21.494 1.00107.44 C
ATOM 3506 C GLU A 270 -22.981 11.423 -22.460 1.00104.43 C
ATOM 3507 O GLU A 270 -22.075 11.934 -23.130 1.00109.67 O
ATOM 3508 CB GLU A 270 -23.083 12.374 -20.145 1.00 0.00 C
ATOM 3509 CG GLU A 270 -22.197 13.608 -19.914 1.00 0.00 C
ATOM 3510 CD GLU A 270 -21.495 13.692 -18.562 1.00 0.00 C
ATOM 3511 OE1 GLU A 270 -20.358 13.180 -18.462 1.00 0.00 O
ATOM 3512 OE2 GLU A 270 -22.112 14.277 -17.648 1.00 0.00 O1-
ATOM 3513 H GLU A 270 -25.373 11.352 -20.420 1.00103.76 H
ATOM 3514 HA GLU A 270 -23.875 13.285 -21.910 1.00107.44 H
ATOM 3515 HB2 GLU A 270 -23.826 12.358 -19.345 1.00 0.00 H

ATOM 3516 HB3 GLU A 270 -22.482 11.480 -19.967 1.00 0.00 H
ATOM 3517 HG2 GLU A 270 -21.434 13.651 -20.693 1.00 0.00 H
ATOM 3518 HG3 GLU A 270 -22.798 14.510 -20.039 1.00 0.00 H
ATOM 3519 N LYS A 271 -23.287 10.127 -22.544 1.00100.41 N
ATOM 3520 CA LYS A 271 -22.598 9.261 -23.496 1.00 92.31 C
ATOM 3521 C LYS A 271 -23.097 9.482 -24.918 1.00 87.07 C
ATOM 3522 O LYS A 271 -22.294 9.551 -25.856 1.00 85.19 O
ATOM 3523 CB LYS A 271 -22.772 7.796 -23.102 1.00 89.83 C
ATOM 3524 CG LYS A 271 -22.185 6.818 -24.105 1.00 90.39 C
ATOM 3525 CD LYS A 271 -23.153 5.688 -24.411 1.00 93.35 C
ATOM 3526 CE LYS A 271 -22.565 4.714 -25.418 1.00 88.71 C
ATOM 3527 NZ LYS A 271 -23.491 3.581 -25.697 1.00 91.01 N1+
ATOM 3528 H LYS A 271 -24.016 9.722 -21.973 1.00100.41 H
ATOM 3529 HA LYS A 271 -21.527 9.476 -23.473 1.00 92.31 H
ATOM 3530 HB2 LYS A 271 -22.338 7.618 -22.117 1.00 89.83 H
ATOM 3531 HB3 LYS A 271 -23.838 7.600 -22.981 1.00 89.83 H
ATOM 3532 HG2 LYS A 271 -21.824 7.253 -25.034 1.00 90.39 H
ATOM 3533 HG3 LYS A 271 -21.289 6.386 -23.658 1.00 90.39 H
ATOM 3534 HD2 LYS A 271 -23.399 5.156 -23.491 1.00 93.35 H
ATOM 3535 HD3 LYS A 271 -24.089 6.096 -24.796 1.00 93.35 H
ATOM 3536 HE2 LYS A 271 -22.422 5.233 -26.346 1.00 88.71 H
ATOM 3537 HE3 LYS A 271 -21.608 4.335 -25.088 1.00 88.71 H
ATOM 3538 HZ1 LYS A 271 -23.102 2.991 -26.421 1.00 91.01 H
ATOM 3539 HZ2 LYS A 271 -23.596 3.034 -24.853 1.00 91.01 H
ATOM 3540 HZ3 LYS A 271 -24.386 3.943 -25.992 1.00 91.01 H
ATOM 3541 N ASP A 272 -24.416 9.585 -25.099 1.00 85.65 N
ATOM 3542 CA ASP A 272 -24.960 9.852 -26.426 1.00 85.08 C
ATOM 3543 C ASP A 272 -24.478 11.195 -26.958 1.00 79.45 C
ATOM 3544 O ASP A 272 -24.233 11.343 -28.161 1.00 71.98 O
ATOM 3545 CB ASP A 272 -26.489 9.808 -26.394 1.00 92.27 C
ATOM 3546 CG ASP A 272 -27.034 8.394 -26.363 1.00103.12 C
ATOM 3547 OD1 ASP A 272 -26.237 7.448 -26.199 1.00107.58 O
ATOM 3548 OD2 ASP A 272 -28.263 8.229 -26.512 1.00111.41 O1-
ATOM 3549 H ASP A 272 -25.068 9.474 -24.333 1.00 85.65 H
ATOM 3550 HA ASP A 272 -24.584 9.095 -27.119 1.00 85.08 H
ATOM 3551 HB2 ASP A 272 -26.862 10.348 -25.522 1.00 92.27 H
ATOM 3552 HB3 ASP A 272 -26.888 10.291 -27.288 1.00 92.27 H
ATOM 3553 N ARG A 273 -24.325 12.183 -26.073 1.00 80.33 N
ATOM 3554 CA ARG A 273 -23.901 13.510 -26.506 1.00 75.60 C
ATOM 3555 C ARG A 273 -22.468 13.489 -27.022 1.00 76.97 C

```
ATOM   3556  O   ARG A 273     -22.182  14.006 -28.108  1.00 71.69           O
ATOM   3557  CB  ARG A 273     -24.048  14.504 -25.358  1.00  0.00           C
ATOM   3558  CG  ARG A 273     -23.898  16.017 -25.635  1.00  0.00           C
ATOM   3559  CD  ARG A 273     -24.048  17.001 -24.474  1.00  0.00           C
ATOM   3560  NE  ARG A 273     -23.880  18.394 -24.900  1.00  0.00           N
ATOM   3561  CZ  ARG A 273     -22.701  19.040 -24.953  1.00  0.00           C
ATOM   3562  NH1 ARG A 273     -21.568  18.428 -24.546  1.00  0.00           N1+
ATOM   3563  NH2 ARG A 273     -22.685  20.308 -25.412  1.00  0.00           N1+
ATOM   3564  H   ARG A 273     -24.541  12.046 -25.094  1.00 80.33           H
ATOM   3565  HA  ARG A 273     -24.549  13.837 -27.321  1.00 75.60           H
ATOM   3566  HB2 ARG A 273     -25.036  14.368 -24.918  1.00  0.00           H
ATOM   3567  HB3 ARG A 273     -23.349  14.232 -24.565  1.00  0.00           H
ATOM   3568  HG2 ARG A 273     -22.973  16.233 -26.171  1.00  0.00           H
ATOM   3569  HG3 ARG A 273     -24.694  16.248 -26.343  1.00  0.00           H
ATOM   3570  HD2 ARG A 273     -25.094  16.956 -24.169  1.00  0.00           H
ATOM   3571  HD3 ARG A 273     -23.477  16.725 -23.587  1.00  0.00           H
ATOM   3572  HE  ARG A 273     -24.710  18.867 -25.225  1.00  0.00           H
ATOM   3573 HH11 ARG A 273     -20.687  18.922 -24.541  1.00  0.00           H
ATOM   3574 HH12 ARG A 273     -21.603  17.478 -24.206  1.00  0.00           H
ATOM   3575 HH21 ARG A 273     -23.542  20.764 -25.689  1.00  0.00           H
ATOM   3576 HH22 ARG A 273     -21.818  20.823 -25.460  1.00  0.00           H
ATOM   3577  N   ASN A 274     -21.550  12.892 -26.256  1.00 81.13           N
ATOM   3578  CA  ASN A 274     -20.161  12.809 -26.699  1.00 81.73           C
ATOM   3579  C   ASN A 274     -20.029  11.976 -27.966  1.00 72.25           C
ATOM   3580  O   ASN A 274     -19.188  12.275 -28.821  1.00 71.93           O
ATOM   3581  CB  ASN A 274     -19.282  12.230 -25.588  1.00 90.34           C
ATOM   3582  CG  ASN A 274     -18.566  13.301 -24.792  1.00 93.48           C
ATOM   3583  ND2 ASN A 274     -18.733  13.404 -23.576  1.00 88.75           N
ATOM   3584  OD1 ASN A 274     -17.762  14.107 -25.476  1.00 93.91           O
ATOM   3585  H   ASN A 274     -21.794  12.468 -25.371  1.00 81.13           H
ATOM   3586  HA  ASN A 274     -19.869  13.840 -26.916  1.00 81.73           H
ATOM   3587  HB2 ASN A 274     -19.839  11.543 -24.947  1.00 90.34           H
ATOM   3588  HB3 ASN A 274     -18.473  11.633 -26.015  1.00 90.34           H
ATOM   3589 HD21 ASN A 274     -19.355  12.776 -23.087  1.00 88.75           H
ATOM   3590 HD22 ASN A 274     -18.239  14.116 -23.057  1.00 88.75           H
ATOM   3591  N   LEU A 275     -20.847  10.931 -28.108  1.00 70.70           N
ATOM   3592  CA  LEU A 275     -20.783  10.110 -29.311  1.00 66.32           C
ATOM   3593  C   LEU A 275     -21.252  10.882 -30.537  1.00 62.48           C
ATOM   3594  O   LEU A 275     -20.720  10.689 -31.636  1.00 62.81           O
ATOM   3595  CB  LEU A 275     -21.610   8.841 -29.128  1.00 72.59           C
```

```
ATOM   3596  CG  LEU A 275     -20.820   7.591 -28.750  1.00 77.60           C
ATOM   3597  CD1 LEU A 275     -21.772   6.440 -28.509  1.00 88.31           C
ATOM   3598  CD2 LEU A 275     -19.821   7.244 -29.841  1.00 73.97           C
ATOM   3599  H   LEU A 275     -21.515  10.676 -27.392  1.00 70.70           H
ATOM   3600  HA  LEU A 275     -19.739   9.843 -29.487  1.00 66.32           H
ATOM   3601  HB2 LEU A 275     -22.401   9.022 -28.400  1.00 72.59           H
ATOM   3602  HB3 LEU A 275     -22.150   8.604 -30.048  1.00 72.59           H
ATOM   3603  HG  LEU A 275     -20.283   7.791 -27.821  1.00 77.60           H
ATOM   3604 HD11 LEU A 275     -21.222   5.750 -27.886  1.00 88.31           H
ATOM   3605 HD12 LEU A 275     -22.684   6.749 -27.997  1.00 88.31           H
ATOM   3606 HD13 LEU A 275     -22.051   5.926 -29.430  1.00 88.31           H
ATOM   3607 HD21 LEU A 275     -19.794   6.174 -30.054  1.00 73.97           H
ATOM   3608 HD22 LEU A 275     -20.033   7.749 -30.784  1.00 73.97           H
ATOM   3609 HD23 LEU A 275     -18.813   7.532 -29.544  1.00 73.97           H
ATOM   3610  N   ARG A 276     -22.251  11.754 -30.375  1.00 61.37           N
ATOM   3611  CA  ARG A 276     -22.689  12.576 -31.498  1.00 57.99           C
ATOM   3612  C   ARG A 276     -21.581  13.515 -31.952  1.00 52.50           C
ATOM   3613  O   ARG A 276     -21.376  13.709 -33.155  1.00 50.49           O
ATOM   3614  CB  ARG A 276     -23.939  13.376 -31.127  1.00 61.23           C
ATOM   3615  CG  ARG A 276     -25.203  12.552 -30.974  1.00 77.57           C
ATOM   3616  CD  ARG A 276     -26.442  13.435 -31.054  1.00 82.77           C
ATOM   3617  NE  ARG A 276     -26.309  14.652 -30.255  1.00 89.73           N
ATOM   3618  CZ  ARG A 276     -26.683  14.760 -28.984  1.00 93.08           C
ATOM   3619  NH1 ARG A 276     -27.218  13.722 -28.356  1.00 94.99           N1+
ATOM   3620  NH2 ARG A 276     -26.523  15.908 -28.339  1.00 92.99           N1+
ATOM   3621  H   ARG A 276     -22.703  11.883 -29.479  1.00 61.37           H
ATOM   3622  HA  ARG A 276     -22.935  11.919 -32.335  1.00 57.99           H
ATOM   3623  HB2 ARG A 276     -23.754  13.957 -30.223  1.00 61.23           H
ATOM   3624  HB3 ARG A 276     -24.114  14.103 -31.923  1.00 61.23           H
ATOM   3625  HG2 ARG A 276     -25.247  11.911 -31.855  1.00 77.57           H
ATOM   3626  HG3 ARG A 276     -25.245  11.865 -30.144  1.00 77.57           H
ATOM   3627  HD2 ARG A 276     -26.662  13.672 -32.096  1.00 82.77           H
ATOM   3628  HD3 ARG A 276     -27.314  12.886 -30.701  1.00 82.77           H
ATOM   3629  HE  ARG A 276     -25.871  15.440 -30.709  1.00 89.73           H
ATOM   3630 HH11 ARG A 276     -27.338  12.846 -28.843  1.00 94.99           H
ATOM   3631 HH12 ARG A 276     -27.506  13.790 -27.391  1.00 94.99           H
ATOM   3632 HH21 ARG A 276     -26.843  16.006 -27.385  1.00 92.99           H
ATOM   3633 HH22 ARG A 276     -26.114  16.704 -28.808  1.00 92.99           H
ATOM   3634  N   ARG A 277     -20.851  14.101 -31.000  1.00 51.28           N
ATOM   3635  CA  ARG A 277     -19.790  15.039 -31.353  1.00 55.03           C
```

ATOM 3636 C ARG A 277 -18.608 14.324 -31.997 1.00 54.69 C
ATOM 3637 O ARG A 277 -18.017 14.831 -32.958 1.00 43.51 O
ATOM 3638 CB ARG A 277 -19.345 15.809 -30.110 1.00 63.64 C
ATOM 3639 CG ARG A 277 -18.467 17.014 -30.400 1.00 74.64 C
ATOM 3640 CD ARG A 277 -18.324 17.891 -29.166 1.00 87.10 C
ATOM 3641 NE ARG A 277 -17.745 19.194 -29.482 1.00 94.98 N
ATOM 3642 CZ ARG A 277 -17.658 20.201 -28.619 1.00102.19 C
ATOM 3643 NH1 ARG A 277 -18.114 20.059 -27.381 1.00101.71 N1+
ATOM 3644 NH2 ARG A 277 -17.117 21.352 -28.993 1.00106.17 N1+
ATOM 3645 H ARG A 277 -21.026 13.923 -30.020 1.00 51.28 H
ATOM 3646 HA ARG A 277 -20.185 15.758 -32.074 1.00 55.03 H
ATOM 3647 HB2 ARG A 277 -20.253 16.166 -29.622 1.00 63.64 H
ATOM 3648 HB3 ARG A 277 -18.862 15.149 -29.386 1.00 63.64 H
ATOM 3649 HG2 ARG A 277 -17.484 16.645 -30.695 1.00 74.64 H
ATOM 3650 HG3 ARG A 277 -18.840 17.601 -31.238 1.00 74.64 H
ATOM 3651 HD2 ARG A 277 -19.267 17.975 -28.624 1.00 87.10 H
ATOM 3652 HD3 ARG A 277 -17.609 17.425 -28.486 1.00 87.10 H
ATOM 3653 HE ARG A 277 -17.339 19.297 -30.401 1.00 94.98 H
ATOM 3654 HH11 ARG A 277 -18.501 19.175 -27.087 1.00101.71 H
ATOM 3655 HH12 ARG A 277 -18.031 20.811 -26.713 1.00101.71 H
ATOM 3656 HH21 ARG A 277 -16.770 21.469 -29.934 1.00106.17 H
ATOM 3657 HH22 ARG A 277 -17.062 22.126 -28.346 1.00106.17 H
ATOM 3658 N ILE A 278 -18.249 13.145 -31.487 1.00 50.99 N
ATOM 3659 CA ILE A 278 -17.107 12.419 -32.036 1.00 49.06 C
ATOM 3660 C ILE A 278 -17.440 11.864 -33.415 1.00 48.06 C
ATOM 3661 O ILE A 278 -16.641 11.969 -34.354 1.00 53.28 O
ATOM 3662 CB ILE A 278 -16.662 11.307 -31.068 1.00 51.00 C
ATOM 3663 CG1 ILE A 278 -16.136 11.920 -29.770 1.00 57.34 C
ATOM 3664 CG2 ILE A 278 -15.596 10.432 -31.709 1.00 47.00 C
ATOM 3665 CD1 ILE A 278 -15.828 10.901 -28.697 1.00 65.15 C
ATOM 3666 H ILE A 278 -18.725 12.747 -30.687 1.00 50.99 H
ATOM 3667 HA ILE A 278 -16.271 13.113 -32.153 1.00 49.06 H
ATOM 3668 HB ILE A 278 -17.525 10.682 -30.828 1.00 51.00 H
ATOM 3669 HG12 ILE A 278 -15.224 12.477 -29.988 1.00 57.34 H
ATOM 3670 HG13 ILE A 278 -16.819 12.662 -29.363 1.00 57.34 H
ATOM 3671 HG21 ILE A 278 -15.208 9.698 -31.004 1.00 47.00 H
ATOM 3672 HG22 ILE A 278 -15.972 9.847 -32.549 1.00 47.00 H
ATOM 3673 HG23 ILE A 278 -14.749 11.022 -32.062 1.00 47.00 H
ATOM 3674 HD11 ILE A 278 -15.902 11.351 -27.707 1.00 65.15 H
ATOM 3675 HD12 ILE A 278 -16.526 10.063 -28.724 1.00 65.15 H

ATOM 3676 HD13 ILE A 278 -14.817 10.505 -28.800 1.00 65.15 H
ATOM 3677 N THR A 279 -18.625 11.266 -33.559 1.00 48.07 N
ATOM 3678 CA THR A 279 -19.038 10.744 -34.858 1.00 51.54 C
ATOM 3679 C THR A 279 -19.117 11.858 -35.894 1.00 47.86 C
ATOM 3680 O THR A 279 -18.729 11.669 -37.053 1.00 46.32 O
ATOM 3681 CB THR A 279 -20.385 10.031 -34.730 1.00 54.64 C
ATOM 3682 CG2 THR A 279 -20.781 9.386 -36.048 1.00 51.56 C
ATOM 3683 OG1 THR A 279 -20.292 9.018 -33.719 1.00 63.23 O
ATOM 3684 H THR A 279 -19.275 11.187 -32.788 1.00 48.07 H
ATOM 3685 HA THR A 279 -18.286 10.031 -35.190 1.00 51.54 H
ATOM 3686 HB THR A 279 -21.163 10.731 -34.421 1.00 54.64 H
ATOM 3687 HG1 THR A 279 -20.286 9.453 -32.860 1.00 63.23 H
ATOM 3688 HG21 THR A 279 -21.624 8.709 -35.909 1.00 51.56 H
ATOM 3689 HG22 THR A 279 -21.099 10.131 -36.777 1.00 51.56 H
ATOM 3690 HG23 THR A 279 -19.972 8.815 -36.499 1.00 51.56 H
ATOM 3691 N ARG A 280 -19.607 13.031 -35.489 1.00 46.07 N
ATOM 3692 CA ARG A 280 -19.668 14.169 -36.397 1.00 47.43 C
ATOM 3693 C ARG A 280 -18.275 14.585 -36.853 1.00 48.06 C
ATOM 3694 O ARG A 280 -18.056 14.850 -38.042 1.00 45.33 O
ATOM 3695 CB ARG A 280 -20.382 15.332 -35.711 1.00 48.92 C
ATOM 3696 CG ARG A 280 -20.939 16.389 -36.639 1.00 63.28 C
ATOM 3697 CD ARG A 280 -21.715 17.411 -35.829 1.00 78.29 C
ATOM 3698 NE ARG A 280 -20.849 18.104 -34.878 1.00 82.48 N
ATOM 3699 CZ ARG A 280 -21.228 18.495 -33.666 1.00 84.32 C
ATOM 3700 NH1 ARG A 280 -22.461 18.251 -33.242 1.00 86.38 N1+
ATOM 3701 NH2 ARG A 280 -20.371 19.122 -32.873 1.00 84.38 N1+
ATOM 3702 H ARG A 280 -19.936 13.166 -34.542 1.00 46.07 H
ATOM 3703 HA ARG A 280 -20.243 13.874 -37.278 1.00 47.43 H
ATOM 3704 HB2 ARG A 280 -21.254 14.920 -35.211 1.00 48.92 H
ATOM 3705 HB3 ARG A 280 -19.751 15.756 -34.929 1.00 48.92 H
ATOM 3706 HG2 ARG A 280 -20.118 16.881 -37.162 1.00 63.28 H
ATOM 3707 HG3 ARG A 280 -21.579 15.952 -37.406 1.00 63.28 H
ATOM 3708 HD2 ARG A 280 -22.243 18.122 -36.466 1.00 78.29 H
ATOM 3709 HD3 ARG A 280 -22.488 16.859 -35.296 1.00 78.29 H
ATOM 3710 HE ARG A 280 -19.922 18.338 -35.205 1.00 82.48 H
ATOM 3711 HH11 ARG A 280 -23.118 17.786 -33.851 1.00 86.38 H
ATOM 3712 HH12 ARG A 280 -22.759 18.540 -32.322 1.00 86.38 H
ATOM 3713 HH21 ARG A 280 -19.434 19.317 -33.194 1.00 84.38 H
ATOM 3714 HH22 ARG A 280 -20.658 19.438 -31.958 1.00 84.38 H
ATOM 3715 N MET A 281 -17.317 14.638 -35.923 1.00 41.46 N

ATOM	3716	CA	MET	A	281	-15.951	15.004	-36.287	1.00	49.30	C
ATOM	3717	C	MET	A	281	-15.314	13.951	-37.185	1.00	49.10	C
ATOM	3718	O	MET	A	281	-14.569	14.289	-38.112	1.00	41.14	O
ATOM	3719	CB	MET	A	281	-15.108	15.218	-35.031	1.00	49.22	C
ATOM	3720	CG	MET	A	281	-14.931	16.679	-34.656	1.00	69.05	C
ATOM	3721	SD	MET	A	281	-14.024	17.598	-35.916	1.00	74.17	S
ATOM	3722	CE	MET	A	281	-12.413	16.825	-35.799	1.00	79.13	C
ATOM	3723	H	MET	A	281	-17.509	14.421	-34.954	1.00	41.46	H
ATOM	3724	HA	MET	A	281	-15.993	15.925	-36.870	1.00	49.30	H
ATOM	3725	HB2	MET	A	281	-15.554	14.691	-34.186	1.00	49.22	H
ATOM	3726	HB3	MET	A	281	-14.124	14.758	-35.127	1.00	49.22	H
ATOM	3727	HG2	MET	A	281	-15.905	17.148	-34.513	1.00	69.05	H
ATOM	3728	HG3	MET	A	281	-14.399	16.763	-33.708	1.00	69.05	H
ATOM	3729	HE1	MET	A	281	-11.699	17.371	-36.416	1.00	79.13	H
ATOM	3730	HE2	MET	A	281	-12.054	16.837	-34.770	1.00	79.13	H
ATOM	3731	HE3	MET	A	281	-12.440	15.796	-36.153	1.00	79.13	H
ATOM	3732	N	VAL	A	282	-15.591	12.672	-36.922	1.00	50.89	N
ATOM	3733	CA	VAL	A	282	-15.078	11.607	-37.782	1.00	44.87	C
ATOM	3734	C	VAL	A	282	-15.591	11.785	-39.204	1.00	42.27	C
ATOM	3735	O	VAL	A	282	-14.828	11.704	-40.175	1.00	45.91	O
ATOM	3736	CB	VAL	A	282	-15.458	10.228	-37.216	1.00	43.21	C
ATOM	3737	CG1	VAL	A	282	-15.256	9.153	-38.272	1.00	42.99	C
ATOM	3738	CG2	VAL	A	282	-14.636	9.921	-35.974	1.00	39.05	C
ATOM	3739	H	VAL	A	282	-16.176	12.409	-36.138	1.00	50.89	H
ATOM	3740	HA	VAL	A	282	-13.989	11.683	-37.807	1.00	44.87	H
ATOM	3741	HB	VAL	A	282	-16.510	10.230	-36.934	1.00	43.21	H
ATOM	3742	HG11	VAL	A	282	-15.403	8.165	-37.836	1.00	42.99	H
ATOM	3743	HG12	VAL	A	282	-15.982	9.205	-39.084	1.00	42.99	H
ATOM	3744	HG13	VAL	A	282	-14.254	9.184	-38.703	1.00	42.99	H
ATOM	3745	HG21	VAL	A	282	-14.849	8.939	-35.558	1.00	39.05	H
ATOM	3746	HG22	VAL	A	282	-13.572	9.924	-36.212	1.00	39.05	H
ATOM	3747	HG23	VAL	A	282	-14.774	10.650	-35.182	1.00	39.05	H
ATOM	3748	N	LEU	A	283	-16.894	12.036	-39.349	1.00	43.45	N
ATOM	3749	CA	LEU	A	283	-17.464	12.209	-40.679	1.00	47.70	C
ATOM	3750	C	LEU	A	283	-16.869	13.417	-41.391	1.00	47.05	C
ATOM	3751	O	LEU	A	283	-16.700	13.390	-42.614	1.00	41.03	O
ATOM	3752	CB	LEU	A	283	-18.985	12.331	-40.587	1.00	49.45	C
ATOM	3753	CG	LEU	A	283	-19.710	11.086	-40.066	1.00	57.36	C
ATOM	3754	CD1	LEU	A	283	-21.192	11.360	-39.867	1.00	50.34	C
ATOM	3755	CD2	LEU	A	283	-19.501	9.905	-41.002	1.00	60.15	C

ATOM 3756 H LEU A 283 -17.515 12.091 -38.552 1.00 43.45 H
ATOM 3757 HA LEU A 283 -17.213 11.331 -41.277 1.00 47.70 H
ATOM 3758 HB2 LEU A 283 -19.223 13.177 -39.940 1.00 49.45 H
ATOM 3759 HB3 LEU A 283 -19.393 12.588 -41.567 1.00 49.45 H
ATOM 3760 HG LEU A 283 -19.298 10.780 -39.114 1.00 57.36 H
ATOM 3761 HD11 LEU A 283 -21.556 10.883 -38.957 1.00 50.34 H
ATOM 3762 HD12 LEU A 283 -21.402 12.426 -39.771 1.00 50.34 H
ATOM 3763 HD13 LEU A 283 -21.801 10.994 -40.694 1.00 50.34 H
ATOM 3764 HD21 LEU A 283 -20.398 9.295 -41.115 1.00 60.15 H
ATOM 3765 HD22 LEU A 283 -19.198 10.215 -42.003 1.00 60.15 H
ATOM 3766 HD23 LEU A 283 -18.722 9.245 -40.618 1.00 60.15 H
ATOM 3767 N VAL A 284 -16.524 14.470 -40.648 1.00 43.86 N
ATOM 3768 CA VAL A 284 -15.973 15.667 -41.277 1.00 41.94 C
ATOM 3769 C VAL A 284 -14.565 15.402 -41.803 1.00 40.71 C
ATOM 3770 O VAL A 284 -14.264 15.686 -42.968 1.00 41.57 O
ATOM 3771 CB VAL A 284 -15.998 16.856 -40.300 1.00 47.01 C
ATOM 3772 CG1 VAL A 284 -15.199 18.021 -40.868 1.00 41.31 C
ATOM 3773 CG2 VAL A 284 -17.431 17.288 -40.030 1.00 50.78 C
ATOM 3774 H VAL A 284 -16.671 14.481 -39.648 1.00 43.86 H
ATOM 3775 HA VAL A 284 -16.599 15.930 -42.134 1.00 41.94 H
ATOM 3776 HB VAL A 284 -15.553 16.553 -39.351 1.00 47.01 H
ATOM 3777 HG11 VAL A 284 -15.344 18.917 -40.263 1.00 41.31 H
ATOM 3778 HG12 VAL A 284 -14.127 17.828 -40.865 1.00 41.31 H
ATOM 3779 HG13 VAL A 284 -15.501 18.265 -41.887 1.00 41.31 H
ATOM 3780 HG21 VAL A 284 -17.482 17.955 -39.169 1.00 50.78 H
ATOM 3781 HG22 VAL A 284 -17.841 17.826 -40.885 1.00 50.78 H
ATOM 3782 HG23 VAL A 284 -18.104 16.456 -39.847 1.00 50.78 H
ATOM 3783 N VAL A 285 -13.683 14.852 -40.962 1.00 34.11 N
ATOM 3784 CA VAL A 285 -12.294 14.685 -41.388 1.00 37.45 C
ATOM 3785 C VAL A 285 -12.192 13.673 -42.524 1.00 38.88 C
ATOM 3786 O VAL A 285 -11.316 13.793 -43.390 1.00 37.18 O
ATOM 3787 CB VAL A 285 -11.383 14.300 -40.203 1.00 41.52 C
ATOM 3788 CG1 VAL A 285 -11.556 15.280 -39.048 1.00 47.21 C
ATOM 3789 CG2 VAL A 285 -11.631 12.866 -39.750 1.00 46.57 C
ATOM 3790 H VAL A 285 -13.930 14.605 -40.012 1.00 34.11 H
ATOM 3791 HA VAL A 285 -11.937 15.641 -41.775 1.00 37.45 H
ATOM 3792 HB VAL A 285 -10.349 14.376 -40.537 1.00 41.52 H
ATOM 3793 HG11 VAL A 285 -10.827 15.084 -38.261 1.00 47.21 H
ATOM 3794 HG12 VAL A 285 -11.391 16.299 -39.392 1.00 47.21 H
ATOM 3795 HG13 VAL A 285 -12.533 15.268 -38.592 1.00 47.21 H

ATOM 3796 HG21 VAL A 285 -11.040 12.646 -38.861 1.00 46.57 H
ATOM 3797 HG22 VAL A 285 -12.670 12.708 -39.479 1.00 46.57 H
ATOM 3798 HG23 VAL A 285 -11.342 12.122 -40.492 1.00 46.57 H
ATOM 3799 N VAL A 286 -13.080 12.678 -42.557 1.00 38.31 N
ATOM 3800 CA VAL A 286 -13.059 11.706 -43.646 1.00 41.29 C
ATOM 3801 C VAL A 286 -13.629 12.317 -44.919 1.00 43.62 C
ATOM 3802 O VAL A 286 -13.077 12.133 -46.011 1.00 42.84 O
ATOM 3803 CB VAL A 286 -13.819 10.429 -43.234 1.00 41.63 C
ATOM 3804 CG1 VAL A 286 -13.973 9.487 -44.422 1.00 40.61 C
ATOM 3805 CG2 VAL A 286 -13.095 9.726 -42.092 1.00 37.79 C
ATOM 3806 H VAL A 286 -13.786 12.575 -41.840 1.00 38.31 H
ATOM 3807 HA VAL A 286 -12.025 11.418 -43.853 1.00 41.29 H
ATOM 3808 HB VAL A 286 -14.816 10.702 -42.882 1.00 41.63 H
ATOM 3809 HG11 VAL A 286 -14.409 8.542 -44.100 1.00 40.61 H
ATOM 3810 HG12 VAL A 286 -14.645 9.874 -45.188 1.00 40.61 H
ATOM 3811 HG13 VAL A 286 -13.013 9.263 -44.889 1.00 40.61 H
ATOM 3812 HG21 VAL A 286 -13.662 8.862 -41.745 1.00 37.79 H
ATOM 3813 HG22 VAL A 286 -12.116 9.367 -42.406 1.00 37.79 H
ATOM 3814 HG23 VAL A 286 -12.938 10.371 -41.230 1.00 37.79 H
ATOM 3815 N ALA A 287 -14.736 13.055 -44.803 1.00 37.74 N
ATOM 3816 CA ALA A 287 -15.328 13.687 -45.976 1.00 41.01 C
ATOM 3817 C ALA A 287 -14.393 14.731 -46.574 1.00 38.64 C
ATOM 3818 O ALA A 287 -14.294 14.851 -47.800 1.00 42.37 O
ATOM 3819 CB ALA A 287 -16.674 14.316 -45.617 1.00 38.91 C
ATOM 3820 H ALA A 287 -15.190 13.198 -43.910 1.00 37.74 H
ATOM 3821 HA ALA A 287 -15.509 12.920 -46.732 1.00 41.01 H
ATOM 3822 HB1 ALA A 287 -17.127 14.801 -46.483 1.00 38.91 H
ATOM 3823 HB2 ALA A 287 -17.377 13.560 -45.265 1.00 38.91 H
ATOM 3824 HB3 ALA A 287 -16.571 15.067 -44.832 1.00 38.91 H
ATOM 3825 N VAL A 288 -13.700 15.495 -45.727 1.00 37.96 N
ATOM 3826 CA VAL A 288 -12.763 16.495 -46.233 1.00 41.92 C
ATOM 3827 C VAL A 288 -11.639 15.823 -47.013 1.00 41.35 C
ATOM 3828 O VAL A 288 -11.283 16.255 -48.116 1.00 37.23 O
ATOM 3829 CB VAL A 288 -12.218 17.358 -45.082 1.00 40.95 C
ATOM 3830 CG1 VAL A 288 -10.996 18.137 -45.542 1.00 42.10 C
ATOM 3831 CG2 VAL A 288 -13.294 18.311 -44.587 1.00 39.17 C
ATOM 3832 H VAL A 288 -13.810 15.409 -44.723 1.00 37.96 H
ATOM 3833 HA VAL A 288 -13.300 17.146 -46.927 1.00 41.92 H
ATOM 3834 HB VAL A 288 -11.927 16.713 -44.251 1.00 40.95 H
ATOM 3835 HG11 VAL A 288 -10.737 18.908 -44.817 1.00 42.10 H

ATOM 3836 HG12 VAL A 288 -10.109 17.511 -45.637 1.00 42.10 H
ATOM 3837 HG13 VAL A 288 -11.169 18.645 -46.492 1.00 42.10 H
ATOM 3838 HG21 VAL A 288 -12.961 18.852 -43.701 1.00 39.17 H
ATOM 3839 HG22 VAL A 288 -13.543 19.052 -45.348 1.00 39.17 H
ATOM 3840 HG23 VAL A 288 -14.219 17.800 -44.327 1.00 39.17 H
ATOM 3841 N PHE A 289 -11.069 14.749 -46.458 1.00 36.73 N
ATOM 3842 CA PHE A 289 -10.011 14.038 -47.169 1.00 38.18 C
ATOM 3843 C PHE A 289 -10.509 13.517 -48.511 1.00 37.36 C
ATOM 3844 O PHE A 289 -9.813 13.628 -49.528 1.00 36.96 O
ATOM 3845 CB PHE A 289 -9.472 12.885 -46.319 1.00 36.26 C
ATOM 3846 CG PHE A 289 -8.263 12.210 -46.916 1.00 35.27 C
ATOM 3847 CD1 PHE A 289 -8.405 11.201 -47.859 1.00 39.24 C
ATOM 3848 CD2 PHE A 289 -6.986 12.594 -46.544 1.00 33.71 C
ATOM 3849 CE1 PHE A 289 -7.297 10.590 -48.416 1.00 34.16 C
ATOM 3850 CE2 PHE A 289 -5.872 11.984 -47.098 1.00 43.23 C
ATOM 3851 CZ PHE A 289 -6.030 10.981 -48.037 1.00 33.37 C
ATOM 3852 H PHE A 289 -11.362 14.397 -45.556 1.00 36.73 H
ATOM 3853 HA PHE A 289 -9.194 14.736 -47.357 1.00 38.18 H
ATOM 3854 HB2 PHE A 289 -9.200 13.261 -45.332 1.00 36.26 H
ATOM 3855 HB3 PHE A 289 -10.249 12.139 -46.143 1.00 36.26 H
ATOM 3856 HD1 PHE A 289 -9.390 10.880 -48.166 1.00 39.24 H
ATOM 3857 HD2 PHE A 289 -6.849 13.380 -45.816 1.00 33.71 H
ATOM 3858 HE1 PHE A 289 -7.417 9.815 -49.158 1.00 34.16 H
ATOM 3859 HE2 PHE A 289 -4.890 12.282 -46.778 1.00 43.23 H
ATOM 3860 HZ PHE A 289 -5.162 10.504 -48.467 1.00 33.37 H
ATOM 3861 N ILE A 290 -11.718 12.954 -48.535 1.00 36.80 N
ATOM 3862 CA ILE A 290 -12.242 12.362 -49.760 1.00 40.36 C
ATOM 3863 C ILE A 290 -12.568 13.441 -50.787 1.00 43.77 C
ATOM 3864 O ILE A 290 -12.258 13.296 -51.974 1.00 42.23 O
ATOM 3865 CB ILE A 290 -13.466 11.485 -49.440 1.00 40.87 C
ATOM 3866 CG1 ILE A 290 -13.027 10.233 -48.680 1.00 45.01 C
ATOM 3867 CG2 ILE A 290 -14.216 11.112 -50.713 1.00 40.33 C
ATOM 3868 CD1 ILE A 290 -14.174 9.325 -48.289 1.00 51.52 C
ATOM 3869 H ILE A 290 -12.275 12.869 -47.694 1.00 36.80 H
ATOM 3870 HA ILE A 290 -11.470 11.721 -50.194 1.00 40.36 H
ATOM 3871 HB ILE A 290 -14.146 12.052 -48.801 1.00 40.87 H
ATOM 3872 HG12 ILE A 290 -12.338 9.661 -49.304 1.00 45.01 H
ATOM 3873 HG13 ILE A 290 -12.454 10.488 -47.790 1.00 45.01 H
ATOM 3874 HG21 ILE A 290 -15.087 10.499 -50.483 1.00 40.33 H
ATOM 3875 HG22 ILE A 290 -14.624 11.961 -51.262 1.00 40.33 H

ATOM 3876 HG23 ILE A 290 -13.597 10.522 -51.385 1.00 40.33 H
ATOM 3877 HD11 ILE A 290 -13.891 8.692 -47.448 1.00 51.52 H
ATOM 3878 HD12 ILE A 290 -15.054 9.894 -47.988 1.00 51.52 H
ATOM 3879 HD13 ILE A 290 -14.458 8.665 -49.109 1.00 51.52 H
ATOM 3880 N VAL A 291 -13.188 14.540 -50.352 1.00 37.21 N
ATOM 3881 CA VAL A 291 -13.561 15.599 -51.287 1.00 44.30 C
ATOM 3882 C VAL A 291 -12.322 16.260 -51.880 1.00 45.60 C
ATOM 3883 O VAL A 291 -12.290 16.599 -53.070 1.00 42.97 O
ATOM 3884 CB VAL A 291 -14.476 16.625 -50.590 1.00 45.98 C
ATOM 3885 CG1 VAL A 291 -14.599 17.892 -51.424 1.00 42.39 C
ATOM 3886 CG2 VAL A 291 -15.851 16.022 -50.334 1.00 45.71 C
ATOM 3887 H VAL A 291 -13.451 14.652 -49.380 1.00 37.21 H
ATOM 3888 HA VAL A 291 -14.119 15.150 -52.112 1.00 44.30 H
ATOM 3889 HB VAL A 291 -14.042 16.896 -49.626 1.00 45.98 H
ATOM 3890 HG11 VAL A 291 -15.377 18.540 -51.018 1.00 42.39 H
ATOM 3891 HG12 VAL A 291 -13.687 18.491 -51.426 1.00 42.39 H
ATOM 3892 HG13 VAL A 291 -14.872 17.674 -52.457 1.00 42.39 H
ATOM 3893 HG21 VAL A 291 -16.467 16.694 -49.737 1.00 45.71 H
ATOM 3894 HG22 VAL A 291 -16.379 15.838 -51.270 1.00 45.71 H
ATOM 3895 HG23 VAL A 291 -15.801 15.071 -49.806 1.00 45.71 H
ATOM 3896 N CYS A 292 -11.276 16.438 -51.070 1.00 42.73 N
ATOM 3897 CA CYS A 292 -10.102 17.178 -51.520 1.00 43.85 C
ATOM 3898 C CYS A 292 -9.166 16.339 -52.383 1.00 43.09 C
ATOM 3899 O CYS A 292 -8.506 16.886 -53.275 1.00 38.94 O
ATOM 3900 CB CYS A 292 -9.333 17.730 -50.318 1.00 40.86 C
ATOM 3901 SG CYS A 292 -10.142 19.125 -49.480 1.00 45.63 S
ATOM 3902 H CYS A 292 -11.310 16.152 -50.100 1.00 42.73 H
ATOM 3903 HA CYS A 292 -10.417 18.035 -52.120 1.00 43.85 H
ATOM 3904 HB2 CYS A 292 -9.153 16.940 -49.587 1.00 40.86 H
ATOM 3905 HB3 CYS A 292 -8.349 18.070 -50.626 1.00 40.86 H
ATOM 3906 HG CYS A 292 -11.159 18.413 -48.987 1.00 45.63 H
ATOM 3907 N TRP A 293 -9.086 15.030 -52.141 1.00 36.06 N
ATOM 3908 CA TRP A 293 -8.083 14.191 -52.785 1.00 38.31 C
ATOM 3909 C TRP A 293 -8.622 13.276 -53.875 1.00 40.47 C
ATOM 3910 O TRP A 293 -7.863 12.913 -54.777 1.00 39.22 O
ATOM 3911 CB TRP A 293 -7.363 13.307 -51.751 1.00 31.17 C
ATOM 3912 CG TRP A 293 -6.386 13.992 -50.857 1.00 35.04 C
ATOM 3913 CD1 TRP A 293 -6.552 14.262 -49.545 1.00 32.70 C
ATOM 3914 CD2 TRP A 293 -5.069 14.505 -51.210 1.00 37.45 C
ATOM 3915 CE2 TRP A 293 -4.448 14.989 -50.022 1.00 37.51 C

ATOM 3916 CE3 TRP A 293 -4.326 14.595 -52.411 1.00 38.20 C
ATOM 3917 NE1 TRP A 293 -5.400 14.831 -49.046 1.00 36.60 N
ATOM 3918 CZ2 TRP A 293 -3.140 15.486 -50.008 1.00 42.29 C
ATOM 3919 CZ3 TRP A 293 -3.011 15.098 -52.414 1.00 39.60 C
ATOM 3920 CH2 TRP A 293 -2.416 15.518 -51.210 1.00 44.36 C
ATOM 3921 H TRP A 293 -9.634 14.610 -51.402 1.00 36.06 H
ATOM 3922 HA TRP A 293 -7.338 14.794 -53.303 1.00 38.31 H
ATOM 3923 HB2 TRP A 293 -8.077 12.748 -51.146 1.00 31.17 H
ATOM 3924 HB3 TRP A 293 -6.773 12.544 -52.261 1.00 31.17 H
ATOM 3925 HD1 TRP A 293 -7.431 14.011 -48.973 1.00 32.70 H
ATOM 3926 HE1 TRP A 293 -5.285 15.114 -48.071 1.00 36.60 H
ATOM 3927 HE3 TRP A 293 -4.769 14.270 -53.340 1.00 38.20 H
ATOM 3928 HZ2 TRP A 293 -2.700 15.825 -49.083 1.00 42.29 H
ATOM 3929 HZ3 TRP A 293 -2.456 15.164 -53.340 1.00 39.60 H
ATOM 3930 HH2 TRP A 293 -1.402 15.879 -51.208 1.00 44.36 H
ATOM 3931 N THR A 294 -9.894 12.878 -53.818 1.00 35.53 N
ATOM 3932 CA THR A 294 -10.429 11.995 -54.855 1.00 38.74 C
ATOM 3933 C THR A 294 -10.381 12.610 -56.250 1.00 43.28 C
ATOM 3934 O THR A 294 -9.973 11.905 -57.190 1.00 37.41 O
ATOM 3935 CB THR A 294 -11.859 11.566 -54.504 1.00 39.95 C
ATOM 3936 CG2 THR A 294 -12.339 10.483 -55.462 1.00 39.76 C
ATOM 3937 OG1 THR A 294 -11.900 11.060 -53.165 1.00 42.89 O
ATOM 3938 H THR A 294 -10.517 13.183 -53.083 1.00 35.53 H
ATOM 3939 HA THR A 294 -9.805 11.098 -54.870 1.00 38.74 H
ATOM 3940 HB THR A 294 -12.559 12.398 -54.553 1.00 39.95 H
ATOM 3941 HG1 THR A 294 -11.919 11.817 -52.568 1.00 42.89 H
ATOM 3942 HG21 THR A 294 -13.298 10.080 -55.135 1.00 39.76 H
ATOM 3943 HG22 THR A 294 -12.489 10.861 -56.473 1.00 39.76 H
ATOM 3944 HG23 THR A 294 -11.637 9.650 -55.512 1.00 39.76 H
ATOM 3945 N PRO A 295 -10.776 13.874 -56.470 1.00 38.48 N
ATOM 3946 CA PRO A 295 -10.812 14.378 -57.857 1.00 41.16 C
ATOM 3947 C PRO A 295 -9.478 14.299 -58.581 1.00 40.37 C
ATOM 3948 O PRO A 295 -9.428 13.789 -59.706 1.00 37.29 O
ATOM 3949 CB PRO A 295 -11.291 15.826 -57.684 1.00 34.57 C
ATOM 3950 CG PRO A 295 -12.081 15.805 -56.429 1.00 39.83 C
ATOM 3951 CD PRO A 295 -11.336 14.865 -55.531 1.00 37.19 C
ATOM 3952 HA PRO A 295 -11.566 13.809 -58.406 1.00 41.16 H
ATOM 3953 HB2 PRO A 295 -10.456 16.520 -57.572 1.00 34.57 H
ATOM 3954 HB3 PRO A 295 -11.896 16.162 -58.525 1.00 34.57 H
ATOM 3955 HG2 PRO A 295 -12.227 16.789 -55.993 1.00 39.83 H

ATOM 3956 HG3 PRO A 295 -13.070 15.392 -56.636 1.00 39.83 H
ATOM 3957 HD2 PRO A 295 -10.513 15.386 -55.039 1.00 37.19 H
ATOM 3958 HD3 PRO A 295 -11.988 14.455 -54.763 1.00 37.19 H
ATOM 3959 N ILE A 296 -8.389 14.780 -57.974 1.00 33.55 N
ATOM 3960 CA ILE A 296 -7.124 14.784 -58.702 1.00 32.57 C
ATOM 3961 C ILE A 296 -6.616 13.361 -58.916 1.00 45.92 C
ATOM 3962 O ILE A 296 -6.043 13.054 -59.969 1.00 40.89 O
ATOM 3963 CB ILE A 296 -6.050 15.633 -57.954 1.00 33.96 C
ATOM 3964 CG1 ILE A 296 -4.732 15.828 -58.733 1.00 35.80 C
ATOM 3965 CG2 ILE A 296 -5.743 15.166 -56.513 1.00 39.02 C
ATOM 3966 CD1 ILE A 296 -4.928 16.407 -60.135 1.00 35.41 C
ATOM 3967 H ILE A 296 -8.420 15.191 -57.052 1.00 33.55 H
ATOM 3968 HA ILE A 296 -7.333 15.207 -59.685 1.00 32.57 H
ATOM 3969 HB ILE A 296 -6.478 16.625 -57.855 1.00 33.96 H
ATOM 3970 HG12 ILE A 296 -4.084 16.505 -58.176 1.00 35.80 H
ATOM 3971 HG13 ILE A 296 -4.186 14.886 -58.804 1.00 35.80 H
ATOM 3972 HG21 ILE A 296 -5.077 15.872 -56.013 1.00 39.02 H
ATOM 3973 HG22 ILE A 296 -6.643 15.102 -55.903 1.00 39.02 H
ATOM 3974 HG23 ILE A 296 -5.247 14.195 -56.489 1.00 39.02 H
ATOM 3975 HD11 ILE A 296 -3.979 16.752 -60.548 1.00 35.41 H
ATOM 3976 HD12 ILE A 296 -5.322 15.649 -60.807 1.00 35.41 H
ATOM 3977 HD13 ILE A 296 -5.618 17.250 -60.134 1.00 35.41 H
ATOM 3978 N HIS A 297 -6.844 12.460 -57.956 1.00 35.20 N
ATOM 3979 CA HIS A 297 -6.381 11.086 -58.130 1.00 37.71 C
ATOM 3980 C HIS A 297 -7.130 10.381 -59.252 1.00 38.29 C
ATOM 3981 O HIS A 297 -6.526 9.644 -60.039 1.00 40.38 O
ATOM 3982 CB HIS A 297 -6.518 10.291 -56.821 1.00 31.05 C
ATOM 3983 CG HIS A 297 -5.428 10.554 -55.816 1.00 37.83 C
ATOM 3984 CD2 HIS A 297 -4.162 10.032 -55.695 1.00 38.14 C
ATOM 3985 ND1 HIS A 297 -5.569 11.424 -54.753 1.00 39.40 N
ATOM 3986 CE1 HIS A 297 -4.440 11.383 -54.039 1.00 43.11 C
ATOM 3987 NE2 HIS A 297 -3.539 10.565 -54.568 1.00 42.96 N
ATOM 3988 H HIS A 297 -7.328 12.700 -57.102 1.00 35.20 H
ATOM 3989 HA HIS A 297 -5.329 11.096 -58.426 1.00 37.71 H
ATOM 3990 HB2 HIS A 297 -7.491 10.469 -56.359 1.00 31.05 H
ATOM 3991 HB3 HIS A 297 -6.492 9.225 -57.040 1.00 31.05 H
ATOM 3992 HD1 HIS A 297 -6.397 11.997 -54.568 1.00 39.40 H
ATOM 3993 HD2 HIS A 297 -3.649 9.334 -56.338 1.00 38.14 H
ATOM 3994 HE1 HIS A 297 -4.270 11.941 -53.128 1.00 43.11 H
ATOM 3995 N ILE A 298 -8.443 10.593 -59.346 1.00 37.36 N

```
ATOM   3996  CA  ILE A 298      -9.211   9.972 -60.419  1.00 40.86           C
ATOM   3997  C   ILE A 298      -8.865  10.613 -61.757  1.00 43.37           C
ATOM   3998  O   ILE A 298      -8.766   9.928 -62.782  1.00 46.82           O
ATOM   3999  CB  ILE A 298     -10.737  10.044 -60.117  1.00 46.71           C
ATOM   4000  CG1 ILE A 298     -11.104   9.194 -58.874  1.00 49.51           C
ATOM   4001  CG2 ILE A 298     -11.661   9.696 -61.303  1.00 41.76           C
ATOM   4002  CD1 ILE A 298     -10.780   7.694 -58.979  1.00 48.01           C
ATOM   4003  H   ILE A 298      -8.935  11.187 -58.690  1.00 37.36           H
ATOM   4004  HA  ILE A 298      -8.924   8.922 -60.507  1.00 40.86           H
ATOM   4005  HB  ILE A 298     -10.964  11.079 -59.854  1.00 46.71           H
ATOM   4006 HG12 ILE A 298     -10.577   9.579 -58.002  1.00 49.51           H
ATOM   4007 HG13 ILE A 298     -12.162   9.321 -58.641  1.00 49.51           H
ATOM   4008 HG21 ILE A 298     -12.706   9.666 -60.994  1.00 41.76           H
ATOM   4009 HG22 ILE A 298     -11.592  10.443 -62.094  1.00 41.76           H
ATOM   4010 HG23 ILE A 298     -11.414   8.728 -61.740  1.00 41.76           H
ATOM   4011 HD11 ILE A 298     -11.372   7.122 -58.264  1.00 48.01           H
ATOM   4012 HD12 ILE A 298     -10.989   7.292 -59.970  1.00 48.01           H
ATOM   4013 HD13 ILE A 298      -9.730   7.502 -58.756  1.00 48.01           H
ATOM   4014  N   TYR A 299      -8.663  11.933 -61.765  1.00 39.57           N
ATOM   4015  CA  TYR A 299      -8.295  12.629 -62.994  1.00 44.17           C
ATOM   4016  C   TYR A 299      -6.982  12.097 -63.557  1.00 43.45           C
ATOM   4017  O   TYR A 299      -6.859  11.879 -64.769  1.00 45.05           O
ATOM   4018  CB  TYR A 299      -8.197  14.130 -62.721  1.00 46.44           C
ATOM   4019  CG  TYR A 299      -8.604  15.017 -63.877  1.00 47.76           C
ATOM   4020  CD1 TYR A 299      -8.254  14.704 -65.182  1.00 52.71           C
ATOM   4021  CD2 TYR A 299      -9.340  16.173 -63.655  1.00 50.45           C
ATOM   4022  CE1 TYR A 299      -8.625  15.520 -66.235  1.00 60.66           C
ATOM   4023  CE2 TYR A 299      -9.718  16.991 -64.698  1.00 52.74           C
ATOM   4024  CZ  TYR A 299      -9.357  16.662 -65.985  1.00 58.62           C
ATOM   4025  OH  TYR A 299      -9.734  17.481 -67.021  1.00 65.76           O
ATOM   4026  H   TYR A 299      -8.769  12.486 -60.923  1.00 39.57           H
ATOM   4027  HA  TYR A 299      -9.085  12.445 -63.725  1.00 44.17           H
ATOM   4028  HB2 TYR A 299      -8.907  14.388 -61.943  1.00 46.44           H
ATOM   4029  HB3 TYR A 299      -7.221  14.424 -62.334  1.00 46.44           H
ATOM   4030  HD1 TYR A 299      -7.700  13.837 -65.432  1.00 52.71           H
ATOM   4031  HD2 TYR A 299      -9.623  16.441 -62.651  1.00 50.45           H
ATOM   4032  HE1 TYR A 299      -8.343  15.262 -67.243  1.00 60.66           H
ATOM   4033  HE2 TYR A 299     -10.295  17.880 -64.496  1.00 52.74           H
ATOM   4034  HH  TYR A 299     -10.211  18.262 -66.723  1.00 65.76           H
ATOM   4035  N   VAL A 300      -5.992  11.875 -62.689  1.00 39.62           N
```

```
ATOM   4036  CA  VAL A 300      -4.701  11.364 -63.143  1.00 41.83           C
ATOM   4037  C   VAL A 300      -4.859   9.974 -63.750  1.00 41.73           C
ATOM   4038  O   VAL A 300      -4.248   9.655 -64.779  1.00 39.04           O
ATOM   4039  CB  VAL A 300      -3.673  11.345 -61.971  1.00 39.45           C
ATOM   4040  CG1 VAL A 300      -2.426  10.456 -62.178  1.00 45.05           C
ATOM   4041  CG2 VAL A 300      -3.221  12.779 -61.637  1.00 33.69           C
ATOM   4042  H   VAL A 300      -6.105  12.067 -61.701  1.00 39.62           H
ATOM   4043  HA  VAL A 300      -4.330  12.025 -63.930  1.00 41.83           H
ATOM   4044  HB  VAL A 300      -4.185  10.963 -61.086  1.00 39.45           H
ATOM   4045 HG11 VAL A 300      -1.710  10.590 -61.368  1.00 45.05           H
ATOM   4046 HG12 VAL A 300      -2.675   9.394 -62.196  1.00 45.05           H
ATOM   4047 HG13 VAL A 300      -1.914  10.698 -63.110  1.00 45.05           H
ATOM   4048 HG21 VAL A 300      -2.656  12.809 -60.706  1.00 33.69           H
ATOM   4049 HG22 VAL A 300      -2.581  13.179 -62.423  1.00 33.69           H
ATOM   4050 HG23 VAL A 300      -4.061  13.460 -61.517  1.00 33.69           H
ATOM   4051  N   ILE A 301      -5.694   9.131 -63.137  1.00 40.23           N
ATOM   4052  CA  ILE A 301      -5.902   7.782 -63.657  1.00 44.41           C
ATOM   4053  C   ILE A 301      -6.592   7.829 -65.015  1.00 45.55           C
ATOM   4054  O   ILE A 301      -6.216   7.102 -65.943  1.00 50.58           O
ATOM   4055  CB  ILE A 301      -6.701   6.906 -62.641  1.00 44.34           C
ATOM   4056  CG1 ILE A 301      -5.885   6.641 -61.349  1.00 41.87           C
ATOM   4057  CG2 ILE A 301      -7.282   5.585 -63.194  1.00 44.72           C
ATOM   4058  CD1 ILE A 301      -4.787   5.571 -61.473  1.00 54.72           C
ATOM   4059  H   ILE A 301      -6.193   9.398 -62.299  1.00 40.23           H
ATOM   4060  HA  ILE A 301      -4.928   7.321 -63.832  1.00 44.41           H
ATOM   4061  HB  ILE A 301      -7.563   7.499 -62.329  1.00 44.34           H
ATOM   4062 HG12 ILE A 301      -5.428   7.562 -60.990  1.00 41.87           H
ATOM   4063 HG13 ILE A 301      -6.571   6.340 -60.556  1.00 41.87           H
ATOM   4064 HG21 ILE A 301      -7.758   5.006 -62.402  1.00 44.72           H
ATOM   4065 HG22 ILE A 301      -8.044   5.758 -63.955  1.00 44.72           H
ATOM   4066 HG23 ILE A 301      -6.506   4.962 -63.640  1.00 44.72           H
ATOM   4067 HD11 ILE A 301      -4.033   5.702 -60.696  1.00 54.72           H
ATOM   4068 HD12 ILE A 301      -5.203   4.570 -61.357  1.00 54.72           H
ATOM   4069 HD13 ILE A 301      -4.275   5.611 -62.434  1.00 54.72           H
ATOM   4070  N   ILE A 302      -7.611   8.680 -65.154  1.00 45.67           N
ATOM   4071  CA  ILE A 302      -8.360   8.740 -66.406  1.00 48.98           C
ATOM   4072  C   ILE A 302      -7.469   9.226 -67.541  1.00 51.01           C
ATOM   4073  O   ILE A 302      -7.524   8.698 -68.658  1.00 50.19           O
ATOM   4074  CB  ILE A 302      -9.609   9.624 -66.240  1.00 48.51           C
ATOM   4075  CG1 ILE A 302     -10.564   8.993 -65.226  1.00 55.47           C
```

ATOM 4076 CG2 ILE A 302 -10.313 9.817 -67.575 1.00 48.96 C
ATOM 4077 CD1 ILE A 302 -11.818 9.790 -64.998 1.00 50.89 C
ATOM 4078 H ILE A 302 -7.922 9.261 -64.385 1.00 45.67 H
ATOM 4079 HA ILE A 302 -8.688 7.728 -66.657 1.00 48.98 H
ATOM 4080 HB ILE A 302 -9.305 10.602 -65.863 1.00 48.51 H
ATOM 4081 HG12 ILE A 302 -10.869 8.020 -65.615 1.00 55.47 H
ATOM 4082 HG13 ILE A 302 -10.099 8.750 -64.280 1.00 55.47 H
ATOM 4083 HG21 ILE A 302 -11.216 10.417 -67.480 1.00 48.96 H
ATOM 4084 HG22 ILE A 302 -9.708 10.358 -68.303 1.00 48.96 H
ATOM 4085 HG23 ILE A 302 -10.597 8.863 -68.020 1.00 48.96 H
ATOM 4086 HD11 ILE A 302 -12.337 9.439 -64.107 1.00 50.89 H
ATOM 4087 HD12 ILE A 302 -11.602 10.850 -64.862 1.00 50.89 H
ATOM 4088 HD13 ILE A 302 -12.524 9.684 -65.822 1.00 50.89 H
ATOM 4089 N LYS A 303 -6.624 10.226 -67.279 1.00 50.46 N
ATOM 4090 CA LYS A 303 -5.708 10.689 -68.315 1.00 54.03 C
ATOM 4091 C LYS A 303 -4.666 9.640 -68.677 1.00 50.57 C
ATOM 4092 O LYS A 303 -4.126 9.681 -69.789 1.00 51.07 O
ATOM 4093 CB LYS A 303 -5.035 12.007 -67.878 1.00 51.95 C
ATOM 4094 CG LYS A 303 -5.947 13.232 -68.074 1.00 61.59 C
ATOM 4095 CD LYS A 303 -5.249 14.603 -67.961 1.00 75.40 C
ATOM 4096 CE LYS A 303 -4.711 14.964 -66.568 1.00 81.93 C
ATOM 4097 NZ LYS A 303 -3.459 14.247 -66.233 1.00 87.87 N1+
ATOM 4098 H LYS A 303 -6.586 10.665 -66.368 1.00 50.46 H
ATOM 4099 HA LYS A 303 -6.282 10.897 -69.221 1.00 54.03 H
ATOM 4100 HB2 LYS A 303 -4.682 11.922 -66.850 1.00 51.95 H
ATOM 4101 HB3 LYS A 303 -4.145 12.164 -68.489 1.00 51.95 H
ATOM 4102 HG2 LYS A 303 -6.398 13.176 -69.066 1.00 61.59 H
ATOM 4103 HG3 LYS A 303 -6.781 13.176 -67.376 1.00 61.59 H
ATOM 4104 HD2 LYS A 303 -4.442 14.665 -68.691 1.00 75.40 H
ATOM 4105 HD3 LYS A 303 -5.960 15.375 -68.258 1.00 75.40 H
ATOM 4106 HE2 LYS A 303 -4.496 16.032 -66.529 1.00 81.93 H
ATOM 4107 HE3 LYS A 303 -5.463 14.761 -65.806 1.00 81.93 H
ATOM 4108 HZ1 LYS A 303 -2.751 14.461 -66.920 1.00 87.87 H
ATOM 4109 HZ2 LYS A 303 -3.140 14.544 -65.322 1.00 87.87 H
ATOM 4110 HZ3 LYS A 303 -3.631 13.252 -66.223 1.00 87.87 H
ATOM 4111 N ALA A 304 -4.367 8.704 -67.774 1.00 46.29 N
ATOM 4112 CA ALA A 304 -3.456 7.624 -68.129 1.00 53.13 C
ATOM 4113 C ALA A 304 -4.120 6.590 -69.029 1.00 55.64 C
ATOM 4114 O ALA A 304 -3.423 5.897 -69.778 1.00 57.36 O
ATOM 4115 CB ALA A 304 -2.911 6.951 -66.871 1.00 47.34 C

ATOM 4116 H ALA A 304 -4.812 8.676 -66.866 1.00 46.29 H
ATOM 4117 HA ALA A 304 -2.596 8.033 -68.664 1.00 53.13 H
ATOM 4118 HB1 ALA A 304 -2.152 6.207 -67.117 1.00 47.34 H
ATOM 4119 HB2 ALA A 304 -2.449 7.681 -66.206 1.00 47.34 H
ATOM 4120 HB3 ALA A 304 -3.695 6.445 -66.307 1.00 47.34 H
ATOM 4121 N LEU A 305 -5.447 6.489 -68.993 1.00 55.18 N
ATOM 4122 CA LEU A 305 -6.178 5.465 -69.728 1.00 55.74 C
ATOM 4123 C LEU A 305 -6.718 5.945 -71.067 1.00 64.03 C
ATOM 4124 O LEU A 305 -6.627 5.215 -72.057 1.00 65.97 O
ATOM 4125 CB LEU A 305 -7.339 4.937 -68.880 1.00 49.70 C
ATOM 4126 CG LEU A 305 -6.953 4.135 -67.637 1.00 54.60 C
ATOM 4127 CD1 LEU A 305 -8.169 3.896 -66.759 1.00 50.28 C
ATOM 4128 CD2 LEU A 305 -6.312 2.813 -68.041 1.00 56.37 C
ATOM 4129 H LEU A 305 -5.986 7.091 -68.386 1.00 55.18 H
ATOM 4130 HA LEU A 305 -5.524 4.619 -69.945 1.00 55.74 H
ATOM 4131 HB2 LEU A 305 -7.944 5.794 -68.576 1.00 49.70 H
ATOM 4132 HB3 LEU A 305 -8.003 4.322 -69.492 1.00 49.70 H
ATOM 4133 HG LEU A 305 -6.233 4.709 -67.054 1.00 54.60 H
ATOM 4134 HD11 LEU A 305 -7.872 3.781 -65.716 1.00 50.28 H
ATOM 4135 HD12 LEU A 305 -8.874 4.727 -66.798 1.00 50.28 H
ATOM 4136 HD13 LEU A 305 -8.715 2.997 -67.048 1.00 50.28 H
ATOM 4137 HD21 LEU A 305 -6.698 1.970 -67.467 1.00 56.37 H
ATOM 4138 HD22 LEU A 305 -6.462 2.575 -69.095 1.00 56.37 H
ATOM 4139 HD23 LEU A 305 -5.236 2.845 -67.868 1.00 56.37 H
ATOM 4140 N VAL A 306 -7.290 7.150 -71.124 1.00 58.11 N
ATOM 4141 CA VAL A 306 -7.920 7.655 -72.335 1.00 67.25 C
ATOM 4142 C VAL A 306 -7.352 9.028 -72.669 1.00 72.12 C
ATOM 4143 O VAL A 306 -6.737 9.696 -71.836 1.00 72.87 O
ATOM 4144 CB VAL A 306 -9.452 7.736 -72.206 1.00 0.00 C
ATOM 4145 CG1 VAL A 306 -9.997 8.652 -73.304 1.00 0.00 C
ATOM 4146 CG2 VAL A 306 -10.120 6.359 -72.332 1.00 0.00 C
ATOM 4147 H VAL A 306 -7.330 7.744 -70.305 1.00 58.11 H
ATOM 4148 HA VAL A 306 -7.689 7.027 -73.198 1.00 67.25 H
ATOM 4149 HB VAL A 306 -9.719 8.156 -71.235 1.00 0.00 H
ATOM 4150 HG11 VAL A 306 -11.082 8.542 -73.319 1.00 0.00 H
ATOM 4151 HG12 VAL A 306 -9.891 9.709 -73.068 1.00 0.00 H
ATOM 4152 HG13 VAL A 306 -9.707 8.383 -74.320 1.00 0.00 H
ATOM 4153 HG21 VAL A 306 -11.203 6.431 -72.230 1.00 0.00 H
ATOM 4154 HG22 VAL A 306 -9.905 5.898 -73.297 1.00 0.00 H
ATOM 4155 HG23 VAL A 306 -9.766 5.680 -71.556 1.00 0.00 H

```
ATOM   4156  N   THR A 307      -7.567   9.438 -73.918  1.00 64.55           N
ATOM   4157  CA  THR A 307      -7.230  10.774 -74.393  1.00 70.31           C
ATOM   4158  C   THR A 307      -8.504  11.612 -74.415  1.00 81.35           C
ATOM   4159  O   THR A 307      -9.489  11.228 -75.052  1.00 93.09           O
ATOM   4160  CB  THR A 307      -6.599  10.718 -75.786  1.00 84.71           C
ATOM   4161  CG2 THR A 307      -6.506  12.111 -76.394  1.00 83.65           C
ATOM   4162  OG1 THR A 307      -5.287  10.145 -75.702  1.00 93.33           O
ATOM   4163  H   THR A 307      -8.079   8.851 -74.560  1.00 64.55           H
ATOM   4164  HA  THR A 307      -6.511  11.254 -73.725  1.00 70.31           H
ATOM   4165  HB  THR A 307      -7.192  10.082 -76.446  1.00 84.71           H
ATOM   4166  HG1 THR A 307      -5.368   9.245 -75.376  1.00 93.33           H
ATOM   4167 HG21 THR A 307      -5.890  12.091 -77.294  1.00 83.65           H
ATOM   4168 HG22 THR A 307      -7.474  12.508 -76.702  1.00 83.65           H
ATOM   4169 HG23 THR A 307      -6.046  12.823 -75.707  1.00 83.65           H
ATOM   4170  N   ILE A 308      -8.485  12.747 -73.722  1.00 82.28           N
ATOM   4171  CA  ILE A 308      -9.685  13.563 -73.538  1.00 92.90           C
ATOM   4172  C   ILE A 308      -9.582  14.841 -74.365  1.00100.78           C
ATOM   4173  O   ILE A 308      -8.471  15.349 -74.575  1.00100.35           O
ATOM   4174  CB  ILE A 308      -9.914  13.868 -72.048  1.00 95.81           C
ATOM   4175  CG1 ILE A 308      -8.669  14.496 -71.420  1.00 95.56           C
ATOM   4176  CG2 ILE A 308     -10.292  12.599 -71.299  1.00 94.07           C
ATOM   4177  CD1 ILE A 308      -8.806  14.752 -69.929  1.00 93.49           C
ATOM   4178  H   ILE A 308      -7.650  13.048 -73.241  1.00 82.28           H
ATOM   4179  HA  ILE A 308     -10.562  13.014 -73.879  1.00 92.90           H
ATOM   4180  HB  ILE A 308     -10.740  14.574 -71.951  1.00 95.81           H
ATOM   4181 HG12 ILE A 308      -7.824  13.815 -71.520  1.00 95.56           H
ATOM   4182 HG13 ILE A 308      -8.349  15.389 -71.940  1.00 95.56           H
ATOM   4183 HG21 ILE A 308     -10.613  12.805 -70.278  1.00 94.07           H
ATOM   4184 HG22 ILE A 308     -11.126  12.092 -71.785  1.00 94.07           H
ATOM   4185 HG23 ILE A 308      -9.464  11.890 -71.252  1.00 94.07           H
ATOM   4186 HD11 ILE A 308      -8.018  15.420 -69.583  1.00 93.49           H
ATOM   4187 HD12 ILE A 308      -9.761  15.223 -69.692  1.00 93.49           H
ATOM   4188 HD13 ILE A 308      -8.734  13.832 -69.350  1.00 93.49           H
ATOM   4189  N   PRO A 309     -10.697  15.388 -74.858  1.00107.03           N
ATOM   4190  CA  PRO A 309     -10.630  16.560 -75.750  1.00112.90           C
ATOM   4191  C   PRO A 309     -10.254  17.823 -74.990  1.00108.59           C
ATOM   4192  O   PRO A 309     -10.976  18.266 -74.095  1.00108.73           O
ATOM   4193  CB  PRO A 309     -12.049  16.648 -76.323  1.00115.55           C
ATOM   4194  CG  PRO A 309     -12.903  16.028 -75.274  1.00111.10           C
ATOM   4195  CD  PRO A 309     -12.079  14.902 -74.701  1.00106.47           C
```

```
ATOM   4196  HA  PRO A 309      -9.923  16.391 -76.566  1.00112.90           H
ATOM   4197  HB2 PRO A 309     -12.365  17.662 -76.575  1.00115.55           H
ATOM   4198  HB3 PRO A 309     -12.105  16.056 -77.237  1.00115.55           H
ATOM   4199  HG2 PRO A 309     -13.109  16.759 -74.491  1.00111.10           H
ATOM   4200  HG3 PRO A 309     -13.867  15.692 -75.657  1.00111.10           H
ATOM   4201  HD2 PRO A 309     -12.351  14.691 -73.667  1.00106.47           H
ATOM   4202  HD3 PRO A 309     -12.207  13.993 -75.291  1.00106.47           H
ATOM   4203  N   GLU A 310      -9.131  18.418 -75.379  1.00107.44           N
ATOM   4204  CA  GLU A 310      -8.588  19.606 -74.723  1.00105.77           C
ATOM   4205  C   GLU A 310      -9.529  20.782 -74.941  1.00101.57           C
ATOM   4206  O   GLU A 310      -9.535  21.402 -76.006  1.00110.79           O
ATOM   4207  CB  GLU A 310      -7.199  19.913 -75.267  1.00114.60           C
ATOM   4208  CG  GLU A 310      -6.524  18.720 -75.921  1.00119.89           C
ATOM   4209  CD  GLU A 310      -5.102  19.006 -76.361  1.00126.69           C
ATOM   4210  OE1 GLU A 310      -4.620  20.139 -76.152  1.00130.51           O
ATOM   4211  OE2 GLU A 310      -4.464  18.089 -76.918  1.00127.18           O1-
ATOM   4212  H   GLU A 310      -8.578  18.029 -76.129  1.00107.44           H
ATOM   4213  HA  GLU A 310      -8.474  19.438 -73.658  1.00105.77           H
ATOM   4214  HB2 GLU A 310      -7.194  20.740 -75.979  1.00114.60           H
ATOM   4215  HB3 GLU A 310      -6.585  20.257 -74.432  1.00114.60           H
ATOM   4216  HG2 GLU A 310      -6.494  17.873 -75.234  1.00119.89           H
ATOM   4217  HG3 GLU A 310      -7.059  18.404 -76.817  1.00119.89           H
ATOM   4218  N   THR A 311     -10.338  21.088 -73.931  1.00 94.81           N
ATOM   4219  CA  THR A 311     -11.302  22.180 -73.979  1.00 94.75           C
ATOM   4220  C   THR A 311     -11.094  23.084 -72.767  1.00 96.99           C
ATOM   4221  O   THR A 311     -10.239  22.830 -71.912  1.00 90.79           O
ATOM   4222  CB  THR A 311     -12.745  21.657 -74.016  1.00 94.98           C
ATOM   4223  CG2 THR A 311     -12.964  20.795 -75.247  1.00 90.62           C
ATOM   4224  OG1 THR A 311     -13.003  20.876 -72.840  1.00 95.98           O
ATOM   4225  H   THR A 311     -10.297  20.560 -73.072  1.00 94.81           H
ATOM   4226  HA  THR A 311     -11.145  22.816 -74.852  1.00 94.75           H
ATOM   4227  HB  THR A 311     -13.458  22.482 -74.044  1.00 94.98           H
ATOM   4228  HG1 THR A 311     -12.612  20.007 -72.969  1.00 95.98           H
ATOM   4229 HG21 THR A 311     -13.999  20.456 -75.298  1.00 90.62           H
ATOM   4230 HG22 THR A 311     -12.768  21.363 -76.157  1.00 90.62           H
ATOM   4231 HG23 THR A 311     -12.339  19.910 -75.280  1.00 90.62           H
ATOM   4232  N   THR A 312     -11.897  24.150 -72.692  1.00 82.29           N
ATOM   4233  CA  THR A 312     -11.830  25.053 -71.548  1.00 79.96           C
ATOM   4234  C   THR A 312     -12.268  24.359 -70.264  1.00 80.22           C
ATOM   4235  O   THR A 312     -11.645  24.537 -69.210  1.00 78.83           O
```

ATOM 4236 CB THR A 312 -12.694 26.285 -71.812 1.00 78.08 C
ATOM 4237 CG2 THR A 312 -12.725 27.195 -70.594 1.00 74.44 C
ATOM 4238 OG1 THR A 312 -12.166 27.007 -72.931 1.00 86.91 O
ATOM 4239 H THR A 312 -12.563 24.366 -73.419 1.00 82.29 H
ATOM 4240 HA THR A 312 -10.794 25.377 -71.422 1.00 79.96 H
ATOM 4241 HB THR A 312 -13.714 25.989 -72.064 1.00 78.08 H
ATOM 4242 HG1 THR A 312 -12.639 27.838 -73.014 1.00 86.91 H
ATOM 4243 HG21 THR A 312 -13.186 28.150 -70.847 1.00 74.44 H
ATOM 4244 HG22 THR A 312 -13.309 26.784 -69.770 1.00 74.44 H
ATOM 4245 HG23 THR A 312 -11.717 27.397 -70.230 1.00 74.44 H
ATOM 4246 N PHE A 313 -13.334 23.557 -70.332 1.00 80.17 N
ATOM 4247 CA PHE A 313 -13.826 22.892 -69.130 1.00 77.78 C
ATOM 4248 C PHE A 313 -12.825 21.867 -68.612 1.00 71.21 C
ATOM 4249 O PHE A 313 -12.668 21.711 -67.397 1.00 67.17 O
ATOM 4250 CB PHE A 313 -15.178 22.232 -69.399 1.00 80.51 C
ATOM 4251 CG PHE A 313 -15.814 21.640 -68.170 1.00 83.53 C
ATOM 4252 CD1 PHE A 313 -16.499 22.445 -67.273 1.00 88.19 C
ATOM 4253 CD2 PHE A 313 -15.726 20.281 -67.911 1.00 82.08 C
ATOM 4254 CE1 PHE A 313 -17.085 21.906 -66.142 1.00 91.16 C
ATOM 4255 CE2 PHE A 313 -16.309 19.736 -66.781 1.00 86.33 C
ATOM 4256 CZ PHE A 313 -16.990 20.550 -65.895 1.00 90.23 C
ATOM 4257 H PHE A 313 -13.825 23.399 -71.200 1.00 80.17 H
ATOM 4258 HA PHE A 313 -13.969 23.649 -68.355 1.00 77.78 H
ATOM 4259 HB2 PHE A 313 -15.867 22.974 -69.804 1.00 80.51 H
ATOM 4260 HB3 PHE A 313 -15.082 21.465 -70.170 1.00 80.51 H
ATOM 4261 HD1 PHE A 313 -16.578 23.507 -67.454 1.00 88.19 H
ATOM 4262 HD2 PHE A 313 -15.193 19.635 -68.594 1.00 82.08 H
ATOM 4263 HE1 PHE A 313 -17.615 22.545 -65.451 1.00 91.16 H
ATOM 4264 HE2 PHE A 313 -16.230 18.676 -66.590 1.00 86.33 H
ATOM 4265 HZ PHE A 313 -17.446 20.127 -65.012 1.00 90.23 H
ATOM 4266 N GLN A 314 -12.137 21.159 -69.511 1.00 67.12 N
ATOM 4267 CA GLN A 314 -11.115 20.213 -69.072 1.00 68.30 C
ATOM 4268 C GLN A 314 -9.958 20.935 -68.395 1.00 62.26 C
ATOM 4269 O GLN A 314 -9.480 20.515 -67.335 1.00 55.39 O
ATOM 4270 CB GLN A 314 -10.598 19.392 -70.251 1.00 71.69 C
ATOM 4271 CG GLN A 314 -9.351 18.594 -69.893 1.00 88.41 C
ATOM 4272 CD GLN A 314 -8.632 18.015 -71.089 1.00103.89 C
ATOM 4273 NE2 GLN A 314 -9.244 17.714 -72.111 1.00108.31 N
ATOM 4274 OE1 GLN A 314 -7.322 17.846 -70.963 1.00115.45 O
ATOM 4275 H GLN A 314 -12.277 21.287 -70.504 1.00 67.12 H

```
ATOM   4276  HA  GLN A 314     -11.560  19.516 -68.361  1.00 68.30           H
ATOM   4277  HB2 GLN A 314     -11.385  18.730 -70.616  1.00 71.69           H
ATOM   4278  HB3 GLN A 314     -10.366  20.073 -71.072  1.00 71.69           H
ATOM   4279  HG2 GLN A 314      -8.575  19.106 -69.328  1.00 88.41           H
ATOM   4280  HG3 GLN A 314      -9.661  17.723 -69.331  1.00 88.41           H
ATOM   4281 HE21 GLN A 314     -10.240  17.864 -72.193  1.00108.31           H
ATOM   4282 HE22 GLN A 314      -8.742  17.324 -72.897  1.00108.31           H
ATOM   4283  N   THR A 315      -9.489  22.024 -69.006  1.00 57.52           N
ATOM   4284  CA  THR A 315      -8.383  22.785 -68.437  1.00 56.67           C
ATOM   4285  C   THR A 315      -8.737  23.320 -67.056  1.00 54.16           C
ATOM   4286  O   THR A 315      -7.980  23.139 -66.094  1.00 56.24           O
ATOM   4287  CB  THR A 315      -8.006  23.931 -69.376  1.00 58.78           C
ATOM   4288  CG2 THR A 315      -7.144  24.957 -68.654  1.00 56.49           C
ATOM   4289  OG1 THR A 315      -7.302  23.410 -70.511  1.00 63.55           O
ATOM   4290  H   THR A 315      -9.890  22.352 -69.875  1.00 57.52           H
ATOM   4291  HA  THR A 315      -7.524  22.117 -68.330  1.00 56.67           H
ATOM   4292  HB  THR A 315      -8.902  24.428 -69.752  1.00 58.78           H
ATOM   4293  HG1 THR A 315      -7.912  22.884 -71.036  1.00 63.55           H
ATOM   4294 HG21 THR A 315      -6.728  25.663 -69.374  1.00 56.49           H
ATOM   4295 HG22 THR A 315      -7.695  25.566 -67.936  1.00 56.49           H
ATOM   4296 HG23 THR A 315      -6.297  24.491 -68.149  1.00 56.49           H
ATOM   4297  N   VAL A 316      -9.892  23.979 -66.940  1.00 51.39           N
ATOM   4298  CA  VAL A 316     -10.287  24.570 -65.666  1.00 55.78           C
ATOM   4299  C   VAL A 316     -10.496  23.490 -64.612  1.00 47.82           C
ATOM   4300  O   VAL A 316     -10.053  23.631 -63.466  1.00 50.50           O
ATOM   4301  CB  VAL A 316     -11.545  25.439 -65.847  1.00 53.30           C
ATOM   4302  CG1 VAL A 316     -12.050  25.928 -64.498  1.00 54.04           C
ATOM   4303  CG2 VAL A 316     -11.243  26.615 -66.761  1.00 54.96           C
ATOM   4304  H   VAL A 316     -10.506  24.115 -67.734  1.00 51.39           H
ATOM   4305  HA  VAL A 316      -9.474  25.214 -65.322  1.00 55.78           H
ATOM   4306  HB  VAL A 316     -12.330  24.843 -66.317  1.00 53.30           H
ATOM   4307 HG11 VAL A 316     -12.836  26.672 -64.630  1.00 54.04           H
ATOM   4308 HG12 VAL A 316     -12.488  25.131 -63.896  1.00 54.04           H
ATOM   4309 HG13 VAL A 316     -11.256  26.398 -63.916  1.00 54.04           H
ATOM   4310 HG21 VAL A 316     -12.151  27.174 -66.987  1.00 54.96           H
ATOM   4311 HG22 VAL A 316     -10.544  27.306 -66.287  1.00 54.96           H
ATOM   4312 HG23 VAL A 316     -10.800  26.325 -67.711  1.00 54.96           H
ATOM   4313  N   SER A 317     -11.163  22.393 -64.979  1.00 50.47           N
ATOM   4314  CA  SER A 317     -11.410  21.328 -64.012  1.00 49.23           C
ATOM   4315  C   SER A 317     -10.113  20.673 -63.555  1.00 44.14           C
```

ATOM 4316 O SER A 317 -10.006 20.259 -62.396 1.00 43.44 O
ATOM 4317 CB SER A 317 -12.355 20.283 -64.605 1.00 50.53 C
ATOM 4318 OG SER A 317 -11.782 19.666 -65.742 1.00 58.12 O
ATOM 4319 H SER A 317 -11.530 22.281 -65.915 1.00 50.47 H
ATOM 4320 HA SER A 317 -11.901 21.760 -63.138 1.00 49.23 H
ATOM 4321 HB2 SER A 317 -12.586 19.512 -63.869 1.00 50.53 H
ATOM 4322 HB3 SER A 317 -13.304 20.740 -64.887 1.00 50.53 H
ATOM 4323 HG SER A 317 -11.892 20.268 -66.485 1.00 58.12 H
ATOM 4324 N TRP A 318 -9.119 20.582 -64.442 1.00 45.31 N
ATOM 4325 CA TRP A 318 -7.839 19.984 -64.073 1.00 46.89 C
ATOM 4326 C TRP A 318 -7.142 20.799 -62.993 1.00 57.36 C
ATOM 4327 O TRP A 318 -6.685 20.253 -61.981 1.00 38.72 O
ATOM 4328 CB TRP A 318 -6.986 19.794 -65.344 1.00 48.57 C
ATOM 4329 CG TRP A 318 -5.553 19.406 -65.145 1.00 51.15 C
ATOM 4330 CD1 TRP A 318 -4.491 20.169 -65.482 1.00 48.66 C
ATOM 4331 CD2 TRP A 318 -5.003 18.200 -64.532 1.00 48.60 C
ATOM 4332 CE2 TRP A 318 -3.578 18.304 -64.536 1.00 48.21 C
ATOM 4333 CE3 TRP A 318 -5.557 17.032 -63.959 1.00 47.40 C
ATOM 4334 NE1 TRP A 318 -3.326 19.518 -65.135 1.00 48.89 N
ATOM 4335 CZ2 TRP A 318 -2.749 17.306 -63.997 1.00 49.10 C
ATOM 4336 CZ3 TRP A 318 -4.735 16.016 -63.430 1.00 42.95 C
ATOM 4337 CH2 TRP A 318 -3.334 16.157 -63.437 1.00 46.58 C
ATOM 4338 H TRP A 318 -9.233 20.905 -65.394 1.00 45.31 H
ATOM 4339 HA TRP A 318 -8.005 18.985 -63.665 1.00 46.89 H
ATOM 4340 HB2 TRP A 318 -7.448 19.039 -65.981 1.00 48.57 H
ATOM 4341 HB3 TRP A 318 -6.990 20.714 -65.928 1.00 48.57 H
ATOM 4342 HD1 TRP A 318 -4.560 21.146 -65.940 1.00 48.66 H
ATOM 4343 HE1 TRP A 318 -2.411 19.934 -65.269 1.00 48.89 H
ATOM 4344 HE3 TRP A 318 -6.630 16.924 -63.934 1.00 47.40 H
ATOM 4345 HZ2 TRP A 318 -1.675 17.425 -64.006 1.00 49.10 H
ATOM 4346 HZ3 TRP A 318 -5.177 15.127 -63.008 1.00 42.95 H
ATOM 4347 HH2 TRP A 318 -2.704 15.393 -63.009 1.00 46.58 H
ATOM 4348 N HIS A 319 -7.051 22.115 -63.188 1.00 40.89 N
ATOM 4349 CA HIS A 319 -6.388 22.947 -62.193 1.00 48.50 C
ATOM 4350 C HIS A 319 -7.219 23.072 -60.924 1.00 46.14 C
ATOM 4351 O HIS A 319 -6.659 23.242 -59.836 1.00 43.27 O
ATOM 4352 CB HIS A 319 -6.039 24.317 -62.805 1.00 41.28 C
ATOM 4353 CG HIS A 319 -4.897 24.242 -63.783 1.00 47.80 C
ATOM 4354 CD2 HIS A 319 -4.925 24.104 -65.154 1.00 44.70 C
ATOM 4355 ND1 HIS A 319 -3.561 24.252 -63.370 1.00 42.39 N

```
ATOM   4356  CE1 HIS A 319      -2.849  24.084 -64.477  1.00 53.88           C
ATOM   4357  NE2 HIS A 319      -3.608  23.984 -65.566  1.00 45.55           N
ATOM   4358  H   HIS A 319      -7.431  22.557 -64.015  1.00 40.89           H
ATOM   4359  HA  HIS A 319      -5.438  22.488 -61.910  1.00 48.50           H
ATOM   4360  HB2 HIS A 319      -6.910  24.755 -63.294  1.00 41.28           H
ATOM   4361  HB3 HIS A 319      -5.749  25.010 -62.014  1.00 41.28           H
ATOM   4362  HD2 HIS A 319      -5.753  24.047 -65.841  1.00 44.70           H
ATOM   4363  HE1 HIS A 319      -1.770  24.020 -64.494  1.00 53.88           H
ATOM   4364  HE2 HIS A 319      -3.270  23.810 -66.504  1.00  0.00           H
ATOM   4365  N   PHE A 320      -8.546  22.973 -61.036  1.00 44.49           N
ATOM   4366  CA  PHE A 320      -9.380  22.972 -59.839  1.00 46.14           C
ATOM   4367  C   PHE A 320      -9.158  21.708 -59.019  1.00 45.94           C
ATOM   4368  O   PHE A 320      -9.114  21.762 -57.784  1.00 43.91           O
ATOM   4369  CB  PHE A 320     -10.853  23.114 -60.220  1.00 48.18           C
ATOM   4370  CG  PHE A 320     -11.761  23.325 -59.042  1.00 66.95           C
ATOM   4371  CD1 PHE A 320     -11.960  24.594 -58.524  1.00 72.86           C
ATOM   4372  CD2 PHE A 320     -12.413  22.256 -58.450  1.00 70.49           C
ATOM   4373  CE1 PHE A 320     -12.791  24.793 -57.438  1.00 72.24           C
ATOM   4374  CE2 PHE A 320     -13.247  22.449 -57.364  1.00 73.40           C
ATOM   4375  CZ  PHE A 320     -13.436  23.719 -56.858  1.00 71.48           C
ATOM   4376  H   PHE A 320      -8.997  22.867 -61.936  1.00 44.49           H
ATOM   4377  HA  PHE A 320      -9.097  23.816 -59.215  1.00 46.14           H
ATOM   4378  HB2 PHE A 320     -10.972  23.970 -60.886  1.00 48.18           H
ATOM   4379  HB3 PHE A 320     -11.189  22.247 -60.791  1.00 48.18           H
ATOM   4380  HD1 PHE A 320     -11.484  25.446 -58.980  1.00 72.86           H
ATOM   4381  HD2 PHE A 320     -12.273  21.257 -58.836  1.00 70.49           H
ATOM   4382  HE1 PHE A 320     -12.940  25.789 -57.047  1.00 72.24           H
ATOM   4383  HE2 PHE A 320     -13.765  21.612 -56.923  1.00 73.40           H
ATOM   4384  HZ  PHE A 320     -14.090  23.873 -56.012  1.00 71.48           H
ATOM   4385  N   CYS A 321      -9.018  20.560 -59.686  1.00 38.53           N
ATOM   4386  CA  CYS A 321      -8.755  19.318 -58.970  1.00 37.83           C
ATOM   4387  C   CYS A 321      -7.378  19.340 -58.319  1.00 38.04           C
ATOM   4388  O   CYS A 321      -7.210  18.854 -57.194  1.00 40.50           O
ATOM   4389  CB  CYS A 321      -8.869  18.098 -59.898  1.00 42.59           C
ATOM   4390  SG  CYS A 321     -10.583  17.919 -60.470  1.00 47.33           S
ATOM   4391  H   CYS A 321      -9.081  20.524 -60.696  1.00 38.53           H
ATOM   4392  HA  CYS A 321      -9.498  19.200 -58.178  1.00 37.83           H
ATOM   4393  HB2 CYS A 321      -8.203  18.187 -60.759  1.00 42.59           H
ATOM   4394  HB3 CYS A 321      -8.589  17.190 -59.367  1.00 42.59           H
ATOM   4395  HG  CYS A 321     -10.533  18.961 -61.319  1.00 47.33           H
```

```
ATOM   4396  N   ILE A 322      -6.383  19.902 -59.011  1.00 35.14           N
ATOM   4397  CA  ILE A 322      -5.076  20.117 -58.396  1.00 35.71           C
ATOM   4398  C   ILE A 322      -5.217  20.979 -57.148  1.00 39.64           C
ATOM   4399  O   ILE A 322      -4.694  20.647 -56.076  1.00 37.75           O
ATOM   4400  CB  ILE A 322      -4.058  20.761 -59.392  1.00 39.56           C
ATOM   4401  CG1 ILE A 322      -3.748  19.816 -60.576  1.00 42.32           C
ATOM   4402  CG2 ILE A 322      -2.721  21.204 -58.757  1.00 41.06           C
ATOM   4403  CD1 ILE A 322      -3.195  20.537 -61.816  1.00 43.31           C
ATOM   4404  H   ILE A 322      -6.531  20.266 -59.943  1.00 35.14           H
ATOM   4405  HA  ILE A 322      -4.678  19.147 -58.091  1.00 35.71           H
ATOM   4406  HB  ILE A 322      -4.524  21.659 -59.801  1.00 39.56           H
ATOM   4407 HG12 ILE A 322      -3.048  19.042 -60.259  1.00 42.32           H
ATOM   4408 HG13 ILE A 322      -4.642  19.278 -60.882  1.00 42.32           H
ATOM   4409 HG21 ILE A 322      -2.044  21.639 -59.492  1.00 41.06           H
ATOM   4410 HG22 ILE A 322      -2.849  21.964 -57.987  1.00 41.06           H
ATOM   4411 HG23 ILE A 322      -2.211  20.347 -58.319  1.00 41.06           H
ATOM   4412 HD11 ILE A 322      -2.331  20.012 -62.222  1.00 43.31           H
ATOM   4413 HD12 ILE A 322      -3.948  20.583 -62.601  1.00 43.31           H
ATOM   4414 HD13 ILE A 322      -2.889  21.562 -61.610  1.00 43.31           H
ATOM   4415  N   ALA A 323      -5.934  22.099 -57.271  1.00 40.13           N
ATOM   4416  CA  ALA A 323      -6.091  23.009 -56.141  1.00 40.25           C
ATOM   4417  C   ALA A 323      -6.810  22.333 -54.982  1.00 41.82           C
ATOM   4418  O   ALA A 323      -6.485  22.580 -53.815  1.00 40.83           O
ATOM   4419  CB  ALA A 323      -6.844  24.266 -56.604  1.00 38.09           C
ATOM   4420  H   ALA A 323      -6.344  22.369 -58.156  1.00 40.13           H
ATOM   4421  HA  ALA A 323      -5.099  23.309 -55.797  1.00 40.25           H
ATOM   4422  HB1 ALA A 323      -6.959  24.980 -55.789  1.00 38.09           H
ATOM   4423  HB2 ALA A 323      -6.307  24.773 -57.407  1.00 38.09           H
ATOM   4424  HB3 ALA A 323      -7.842  24.027 -56.972  1.00 38.09           H
ATOM   4425  N   LEU A 324      -7.786  21.470 -55.282  1.00 39.75           N
ATOM   4426  CA  LEU A 324      -8.499  20.763 -54.222  1.00 45.08           C
ATOM   4427  C   LEU A 324      -7.551  19.913 -53.386  1.00 42.26           C
ATOM   4428  O   LEU A 324      -7.698  19.832 -52.160  1.00 39.12           O
ATOM   4429  CB  LEU A 324      -9.655  19.909 -54.777  1.00 43.49           C
ATOM   4430  CG  LEU A 324     -10.943  20.693 -55.110  1.00 48.74           C
ATOM   4431  CD1 LEU A 324     -11.973  19.733 -55.725  1.00 48.40           C
ATOM   4432  CD2 LEU A 324     -11.526  21.426 -53.880  1.00 45.58           C
ATOM   4433  H   LEU A 324      -8.068  21.304 -56.240  1.00 39.75           H
ATOM   4434  HA  LEU A 324      -8.919  21.512 -53.552  1.00 45.08           H
ATOM   4435  HB2 LEU A 324      -9.313  19.355 -55.652  1.00 43.49           H
```

```
ATOM   4436  HB3 LEU A 324      -9.916  19.145 -54.045  1.00 43.49           H
ATOM   4437  HG  LEU A 324     -10.713  21.452 -55.857  1.00 48.74           H
ATOM   4438 HD11 LEU A 324     -13.003  20.017 -55.515  1.00 48.40           H
ATOM   4439 HD12 LEU A 324     -11.861  19.676 -56.808  1.00 48.40           H
ATOM   4440 HD13 LEU A 324     -11.842  18.734 -55.319  1.00 48.40           H
ATOM   4441 HD21 LEU A 324     -12.604  21.295 -53.779  1.00 45.58           H
ATOM   4442 HD22 LEU A 324     -11.085  21.080 -52.945  1.00 45.58           H
ATOM   4443 HD23 LEU A 324     -11.344  22.499 -53.951  1.00 45.58           H
ATOM   4444  N   GLY A 325      -6.572  19.273 -54.030  1.00 40.68           N
ATOM   4445  CA  GLY A 325      -5.598  18.492 -53.285  1.00 42.40           C
ATOM   4446  C   GLY A 325      -4.819  19.327 -52.287  1.00 43.10           C
ATOM   4447  O   GLY A 325      -4.540  18.880 -51.171  1.00 38.59           O
ATOM   4448  H   GLY A 325      -6.474  19.345 -55.034  1.00 40.68           H
ATOM   4449  HA2 GLY A 325      -6.083  17.665 -52.765  1.00 42.40           H
ATOM   4450  HA3 GLY A 325      -4.896  18.050 -53.992  1.00 42.40           H
ATOM   4451  N   TYR A 326      -4.461  20.553 -52.670  1.00 36.00           N
ATOM   4452  CA  TYR A 326      -3.732  21.426 -51.761  1.00 35.84           C
ATOM   4453  C   TYR A 326      -4.643  22.100 -50.743  1.00 40.18           C
ATOM   4454  O   TYR A 326      -4.145  22.602 -49.729  1.00 43.17           O
ATOM   4455  CB  TYR A 326      -2.951  22.491 -52.554  1.00 36.58           C
ATOM   4456  CG  TYR A 326      -1.816  21.937 -53.386  1.00 39.48           C
ATOM   4457  CD1 TYR A 326      -0.588  21.582 -52.787  1.00 34.77           C
ATOM   4458  CD2 TYR A 326      -1.999  21.757 -54.770  1.00 34.82           C
ATOM   4459  CE1 TYR A 326       0.433  21.003 -53.567  1.00 35.29           C
ATOM   4460  CE2 TYR A 326      -0.980  21.182 -55.544  1.00 33.92           C
ATOM   4461  CZ  TYR A 326       0.220  20.783 -54.941  1.00 34.96           C
ATOM   4462  OH  TYR A 326       1.157  20.184 -55.721  1.00 36.28           O
ATOM   4463  H   TYR A 326      -4.696  20.908 -53.587  1.00 36.00           H
ATOM   4464  HA  TYR A 326      -3.003  20.842 -51.193  1.00 35.84           H
ATOM   4465  HB2 TYR A 326      -3.630  23.040 -53.205  1.00 36.58           H
ATOM   4466  HB3 TYR A 326      -2.523  23.226 -51.872  1.00 36.58           H
ATOM   4467  HD1 TYR A 326      -0.431  21.720 -51.727  1.00 34.77           H
ATOM   4468  HD2 TYR A 326      -2.916  22.059 -55.247  1.00 34.82           H
ATOM   4469  HE1 TYR A 326       1.361  20.699 -53.103  1.00 35.29           H
ATOM   4470  HE2 TYR A 326      -1.110  21.036 -56.602  1.00 33.92           H
ATOM   4471  HH  TYR A 326       1.848  19.703 -55.230  1.00 36.28           H
ATOM   4472  N   THR A 327      -5.957  22.108 -50.988  0.77 34.47           N
ATOM   4473  CA  THR A 327      -6.899  22.616 -49.996  0.77 39.51           C
ATOM   4474  C   THR A 327      -6.949  21.714 -48.769  0.77 39.47           C
ATOM   4475  O   THR A 327      -7.284  22.174 -47.670  0.77 38.93           O
```

```
ATOM   4476  CB  THR A 327      -8.311  22.762 -50.623  0.77 37.33           C
ATOM   4477  CG2 THR A 327      -9.381  23.375 -49.706  0.77 36.73           C
ATOM   4478  OG1 THR A 327      -8.211  23.600 -51.766  0.77 39.40           O
ATOM   4479  H   THR A 327      -6.332  21.688 -51.827  0.77 34.47           H
ATOM   4480  HA  THR A 327      -6.566  23.605 -49.679  0.77 39.51           H
ATOM   4481  HB  THR A 327      -8.677  21.788 -50.947  0.77 37.33           H
ATOM   4482  HG1 THR A 327      -7.573  23.224 -52.393  0.77 39.40           H
ATOM   4483 HG21 THR A 327     -10.308  23.550 -50.252  0.77 36.73           H
ATOM   4484 HG22 THR A 327      -9.625  22.714 -48.874  0.77 36.73           H
ATOM   4485 HG23 THR A 327      -9.053  24.329 -49.293  0.77 36.73           H
ATOM   4486  N   ASN A 328      -6.613  20.433 -48.934  1.00 37.95           N
ATOM   4487  CA  ASN A 328      -6.510  19.542 -47.787  1.00 34.67           C
ATOM   4488  C   ASN A 328      -5.457  20.049 -46.812  1.00 42.24           C
ATOM   4489  O   ASN A 328      -5.640  19.978 -45.592  1.00 41.57           O
ATOM   4490  CB  ASN A 328      -6.189  18.085 -48.202  1.00 33.38           C
ATOM   4491  CG  ASN A 328      -6.246  17.100 -47.023  1.00 37.08           C
ATOM   4492  ND2 ASN A 328      -7.413  16.897 -46.424  1.00 35.00           N
ATOM   4493  OD1 ASN A 328      -5.241  16.498 -46.657  1.00 41.71           O
ATOM   4494  H   ASN A 328      -6.354  20.070 -49.841  1.00 37.95           H
ATOM   4495  HA  ASN A 328      -7.459  19.539 -47.245  1.00 34.67           H
ATOM   4496  HB2 ASN A 328      -6.881  17.733 -48.961  1.00 33.38           H
ATOM   4497  HB3 ASN A 328      -5.197  18.026 -48.655  1.00 33.38           H
ATOM   4498 HD21 ASN A 328      -8.255  17.359 -46.725  1.00 35.00           H
ATOM   4499 HD22 ASN A 328      -7.416  16.340 -45.581  1.00 35.00           H
ATOM   4500  N   SER A 329      -4.354  20.589 -47.338  1.00 39.43           N
ATOM   4501  CA  SER A 329      -3.346  21.212 -46.493  1.00 39.61           C
ATOM   4502  C   SER A 329      -3.847  22.506 -45.865  1.00 47.08           C
ATOM   4503  O   SER A 329      -3.344  22.904 -44.808  1.00 48.88           O
ATOM   4504  CB  SER A 329      -2.109  21.552 -47.349  1.00 43.26           C
ATOM   4505  OG  SER A 329      -1.513  20.380 -47.838  1.00 42.58           O
ATOM   4506  H   SER A 329      -4.211  20.628 -48.337  1.00 39.43           H
ATOM   4507  HA  SER A 329      -3.107  20.525 -45.681  1.00 39.61           H
ATOM   4508  HB2 SER A 329      -2.356  22.201 -48.184  1.00 43.26           H
ATOM   4509  HB3 SER A 329      -1.366  22.105 -46.780  1.00 43.26           H
ATOM   4510  HG  SER A 329      -1.247  19.850 -47.069  1.00 42.58           H
ATOM   4511  N   CYS A 330      -4.828  23.164 -46.487  1.00 40.13           N
ATOM   4512  CA  CYS A 330      -5.420  24.363 -45.905  1.00 42.73           C
ATOM   4513  C   CYS A 330      -6.389  24.035 -44.778  1.00 49.84           C
ATOM   4514  O   CYS A 330      -6.523  24.822 -43.834  1.00 41.74           O
ATOM   4515  CB  CYS A 330      -6.154  25.173 -46.976  1.00 44.61           C
```

ATOM	4516	SG	CYS	A	330	-5.111	25.842	-48.283	1.00	47.63	S
ATOM	4517	H	CYS	A	330	-5.228	22.812	-47.345	1.00	40.13	H
ATOM	4518	HA	CYS	A	330	-4.633	24.998	-45.492	1.00	42.73	H
ATOM	4519	HB2	CYS	A	330	-6.944	24.593	-47.445	1.00	44.61	H
ATOM	4520	HB3	CYS	A	330	-6.667	26.014	-46.512	1.00	44.61	H
ATOM	4521	HG	CYS	A	330	-4.765	24.663	-48.809	1.00	47.63	H
ATOM	4522	N	LEU	A	331	-7.075	22.896	-44.860	1.00	40.93	N
ATOM	4523	CA	LEU	A	331	-8.082	22.543	-43.869	1.00	42.24	C
ATOM	4524	C	LEU	A	331	-7.528	21.710	-42.721	1.00	42.25	C
ATOM	4525	O	LEU	A	331	-8.116	21.716	-41.634	1.00	42.05	O
ATOM	4526	CB	LEU	A	331	-9.237	21.793	-44.541	1.00	38.65	C
ATOM	4527	CG	LEU	A	331	-10.006	22.599	-45.593	1.00	45.28	C
ATOM	4528	CD1	LEU	A	331	-11.029	21.733	-46.312	1.00	43.97	C
ATOM	4529	CD2	LEU	A	331	-10.680	23.809	-44.960	1.00	50.98	C
ATOM	4530	H	LEU	A	331	-6.962	22.280	-45.654	1.00	40.93	H
ATOM	4531	HA	LEU	A	331	-8.496	23.442	-43.410	1.00	42.24	H
ATOM	4532	HB2	LEU	A	331	-8.841	20.891	-45.012	1.00	38.65	H
ATOM	4533	HB3	LEU	A	331	-9.941	21.442	-43.784	1.00	38.65	H
ATOM	4534	HG	LEU	A	331	-9.314	22.969	-46.348	1.00	45.28	H
ATOM	4535	HD11	LEU	A	331	-11.740	22.328	-46.886	1.00	43.97	H
ATOM	4536	HD12	LEU	A	331	-10.534	21.065	-47.017	1.00	43.97	H
ATOM	4537	HD13	LEU	A	331	-11.597	21.118	-45.614	1.00	43.97	H
ATOM	4538	HD21	LEU	A	331	-11.706	23.946	-45.303	1.00	50.98	H
ATOM	4539	HD22	LEU	A	331	-10.726	23.742	-43.872	1.00	50.98	H
ATOM	4540	HD23	LEU	A	331	-10.138	24.723	-45.207	1.00	50.98	H
ATOM	4541	N	ASN	A	332	-6.419	21.002	-42.936	1.00	33.22	N
ATOM	4542	CA	ASN	A	332	-5.813	20.227	-41.855	1.00	40.13	C
ATOM	4543	C	ASN	A	332	-5.482	21.051	-40.613	1.00	42.34	C
ATOM	4544	O	ASN	A	332	-5.663	20.523	-39.502	1.00	44.41	O
ATOM	4545	CB	ASN	A	332	-4.564	19.503	-42.372	1.00	35.44	C
ATOM	4546	CG	ASN	A	332	-4.904	18.285	-43.206	1.00	38.05	C
ATOM	4547	ND2	ASN	A	332	-3.923	17.777	-43.941	1.00	39.09	N
ATOM	4548	OD1	ASN	A	332	-6.037	17.803	-43.186	1.00	44.48	O
ATOM	4549	H	ASN	A	332	-5.973	20.978	-43.844	1.00	33.22	H
ATOM	4550	HA	ASN	A	332	-6.584	19.508	-41.570	1.00	40.13	H
ATOM	4551	HB2	ASN	A	332	-3.944	20.185	-42.951	1.00	35.44	H
ATOM	4552	HB3	ASN	A	332	-3.956	19.146	-41.542	1.00	35.44	H
ATOM	4553	HD21	ASN	A	332	-3.005	18.206	-43.925	1.00	39.09	H
ATOM	4554	HD22	ASN	A	332	-4.090	16.980	-44.539	1.00	39.09	H
ATOM	4555	N	PRO	A	333	-4.996	22.298	-40.703	1.00	38.90	N

ATOM 4556 CA PRO A 333 -4.837 23.093 -39.473 1.00 44.34 C
ATOM 4557 C PRO A 333 -6.130 23.262 -38.701 1.00 49.44 C
ATOM 4558 O PRO A 333 -6.105 23.296 -37.465 1.00 56.97 O
ATOM 4559 CB PRO A 333 -4.314 24.442 -39.985 1.00 50.31 C
ATOM 4560 CG PRO A 333 -3.669 24.132 -41.264 1.00 44.88 C
ATOM 4561 CD PRO A 333 -4.449 23.008 -41.873 1.00 36.54 C
ATOM 4562 HA PRO A 333 -4.080 22.619 -38.845 1.00 44.34 H
ATOM 4563 HB2 PRO A 333 -5.146 25.120 -40.187 1.00 50.31 H
ATOM 4564 HB3 PRO A 333 -3.661 24.970 -39.300 1.00 50.31 H
ATOM 4565 HG2 PRO A 333 -3.521 24.989 -41.922 1.00 44.88 H
ATOM 4566 HG3 PRO A 333 -2.706 23.754 -40.960 1.00 44.88 H
ATOM 4567 HD2 PRO A 333 -5.269 23.384 -42.456 1.00 36.54 H
ATOM 4568 HD3 PRO A 333 -3.815 22.365 -42.477 1.00 36.54 H
ATOM 4569 N VAL A 334 -7.265 23.369 -39.392 1.00 43.96 N
ATOM 4570 CA VAL A 334 -8.540 23.488 -38.696 1.00 42.00 C
ATOM 4571 C VAL A 334 -8.925 22.157 -38.067 1.00 49.70 C
ATOM 4572 O VAL A 334 -9.261 22.087 -36.879 1.00 50.19 O
ATOM 4573 CB VAL A 334 -9.633 23.992 -39.654 1.00 47.12 C
ATOM 4574 CG1 VAL A 334 -10.961 24.102 -38.921 1.00 51.15 C
ATOM 4575 CG2 VAL A 334 -9.237 25.331 -40.259 1.00 54.94 C
ATOM 4576 H VAL A 334 -7.280 23.320 -40.401 1.00 43.96 H
ATOM 4577 HA VAL A 334 -8.462 24.207 -37.885 1.00 42.00 H
ATOM 4578 HB VAL A 334 -9.770 23.288 -40.474 1.00 47.12 H
ATOM 4579 HG11 VAL A 334 -11.710 24.588 -39.547 1.00 51.15 H
ATOM 4580 HG12 VAL A 334 -11.373 23.128 -38.657 1.00 51.15 H
ATOM 4581 HG13 VAL A 334 -10.870 24.690 -38.007 1.00 51.15 H
ATOM 4582 HG21 VAL A 334 -10.033 25.725 -40.891 1.00 54.94 H
ATOM 4583 HG22 VAL A 334 -9.031 26.073 -39.486 1.00 54.94 H
ATOM 4584 HG23 VAL A 334 -8.350 25.248 -40.888 1.00 54.94 H
ATOM 4585 N LEU A 335 -8.859 21.078 -38.851 1.00 42.29 N
ATOM 4586 CA LEU A 335 -9.373 19.791 -38.395 1.00 43.08 C
ATOM 4587 C LEU A 335 -8.516 19.198 -37.285 1.00 40.27 C
ATOM 4588 O LEU A 335 -9.045 18.612 -36.335 1.00 46.11 O
ATOM 4589 CB LEU A 335 -9.459 18.815 -39.569 1.00 44.55 C
ATOM 4590 CG LEU A 335 -10.340 19.216 -40.752 1.00 53.16 C
ATOM 4591 CD1 LEU A 335 -10.269 18.161 -41.846 1.00 53.81 C
ATOM 4592 CD2 LEU A 335 -11.774 19.426 -40.299 1.00 54.61 C
ATOM 4593 H LEU A 335 -8.558 21.153 -39.815 1.00 42.29 H
ATOM 4594 HA LEU A 335 -10.376 19.937 -37.988 1.00 43.08 H
ATOM 4595 HB2 LEU A 335 -8.450 18.652 -39.952 1.00 44.55 H

ATOM 4596 HB3 LEU A 335 -9.784 17.845 -39.192 1.00 44.55 H
ATOM 4597 HG LEU A 335 -9.992 20.155 -41.174 1.00 53.16 H
ATOM 4598 HD11 LEU A 335 -11.252 17.838 -42.186 1.00 53.81 H
ATOM 4599 HD12 LEU A 335 -9.738 18.551 -42.714 1.00 53.81 H
ATOM 4600 HD13 LEU A 335 -9.735 17.265 -41.527 1.00 53.81 H
ATOM 4601 HD21 LEU A 335 -12.472 19.387 -41.135 1.00 54.61 H
ATOM 4602 HD22 LEU A 335 -12.086 18.685 -39.562 1.00 54.61 H
ATOM 4603 HD23 LEU A 335 -11.894 20.412 -39.848 1.00 54.61 H
ATOM 4604 N TYR A 336 -7.193 19.337 -37.383 1.00 38.13 N
ATOM 4605 CA TYR A 336 -6.286 18.592 -36.523 1.00 36.77 C
ATOM 4606 C TYR A 336 -5.520 19.447 -35.524 1.00 41.70 C
ATOM 4607 O TYR A 336 -4.908 18.889 -34.606 1.00 47.50 O
ATOM 4608 CB TYR A 336 -5.287 17.801 -37.378 1.00 40.31 C
ATOM 4609 CG TYR A 336 -5.948 16.732 -38.216 1.00 36.37 C
ATOM 4610 CD1 TYR A 336 -6.325 15.520 -37.651 1.00 44.65 C
ATOM 4611 CD2 TYR A 336 -6.205 16.937 -39.566 1.00 38.09 C
ATOM 4612 CE1 TYR A 336 -6.934 14.539 -38.405 1.00 41.75 C
ATOM 4613 CE2 TYR A 336 -6.813 15.959 -40.332 1.00 37.63 C
ATOM 4614 CZ TYR A 336 -7.174 14.762 -39.744 1.00 37.99 C
ATOM 4615 OH TYR A 336 -7.780 13.785 -40.493 1.00 41.06 O
ATOM 4616 H TYR A 336 -6.782 19.838 -38.161 1.00 38.13 H
ATOM 4617 HA TYR A 336 -6.818 17.857 -35.916 1.00 36.77 H
ATOM 4618 HB2 TYR A 336 -4.723 18.475 -38.024 1.00 40.31 H
ATOM 4619 HB3 TYR A 336 -4.543 17.310 -36.753 1.00 40.31 H
ATOM 4620 HD1 TYR A 336 -6.138 15.338 -36.604 1.00 44.65 H
ATOM 4621 HD2 TYR A 336 -5.920 17.868 -40.031 1.00 38.09 H
ATOM 4622 HE1 TYR A 336 -7.214 13.601 -37.949 1.00 41.75 H
ATOM 4623 HE2 TYR A 336 -7.004 16.135 -41.380 1.00 37.63 H
ATOM 4624 HH TYR A 336 -7.887 14.031 -41.415 1.00 41.06 H
ATOM 4625 N ALA A 337 -5.528 20.769 -35.667 1.00 41.62 N
ATOM 4626 CA ALA A 337 -4.845 21.652 -34.725 1.00 40.93 C
ATOM 4627 C ALA A 337 -5.840 22.510 -33.971 1.00 42.77 C
ATOM 4628 O ALA A 337 -6.023 22.330 -32.762 1.00 47.21 O
ATOM 4629 CB ALA A 337 -3.805 22.511 -35.452 1.00 40.85 C
ATOM 4630 H ALA A 337 -6.031 21.205 -36.427 1.00 41.62 H
ATOM 4631 HA ALA A 337 -4.294 21.091 -33.970 1.00 40.93 H
ATOM 4632 HB1 ALA A 337 -3.074 22.896 -34.740 1.00 40.85 H
ATOM 4633 HB2 ALA A 337 -3.253 21.926 -36.188 1.00 40.85 H
ATOM 4634 HB3 ALA A 337 -4.214 23.378 -35.963 1.00 40.85 H
ATOM 4635 N PHE A 338 -6.516 23.446 -34.648 1.00 42.79 N

ATOM 4636 CA PHE A 338 -7.403 24.377 -33.961 1.00 51.49 C
ATOM 4637 C PHE A 338 -8.590 23.685 -33.300 1.00 53.22 C
ATOM 4638 O PHE A 338 -9.168 24.243 -32.363 1.00 52.31 O
ATOM 4639 CB PHE A 338 -7.895 25.450 -34.936 1.00 50.83 C
ATOM 4640 CG PHE A 338 -6.794 26.313 -35.493 1.00 62.73 C
ATOM 4641 CD1 PHE A 338 -6.030 27.110 -34.655 1.00 74.94 C
ATOM 4642 CD2 PHE A 338 -6.529 26.334 -36.853 1.00 68.50 C
ATOM 4643 CE1 PHE A 338 -5.018 27.905 -35.163 1.00 77.44 C
ATOM 4644 CE2 PHE A 338 -5.520 27.129 -37.368 1.00 65.34 C
ATOM 4645 CZ PHE A 338 -4.763 27.913 -36.521 1.00 71.54 C
ATOM 4646 H PHE A 338 -6.374 23.575 -35.643 1.00 42.79 H
ATOM 4647 HA PHE A 338 -6.839 24.863 -33.164 1.00 51.49 H
ATOM 4648 HB2 PHE A 338 -8.443 24.981 -35.753 1.00 50.83 H
ATOM 4649 HB3 PHE A 338 -8.610 26.109 -34.440 1.00 50.83 H
ATOM 4650 HD1 PHE A 338 -6.223 27.117 -33.592 1.00 74.94 H
ATOM 4651 HD2 PHE A 338 -7.117 25.752 -37.539 1.00 68.50 H
ATOM 4652 HE1 PHE A 338 -4.424 28.516 -34.499 1.00 77.44 H
ATOM 4653 HE2 PHE A 338 -5.322 27.133 -38.430 1.00 65.34 H
ATOM 4654 HZ PHE A 338 -3.963 28.515 -36.924 1.00 71.54 H
ATOM 4655 N LEU A 339 -8.963 22.491 -33.753 1.00 52.02 N
ATOM 4656 CA LEU A 339 -10.037 21.734 -33.123 1.00 52.69 C
ATOM 4657 C LEU A 339 -9.527 20.643 -32.189 1.00 53.87 C
ATOM 4658 O LEU A 339 -10.338 19.926 -31.597 1.00 58.78 O
ATOM 4659 CB LEU A 339 -10.954 21.126 -34.189 1.00 53.30 C
ATOM 4660 CG LEU A 339 -11.822 22.138 -34.945 1.00 59.15 C
ATOM 4661 CD1 LEU A 339 -12.700 21.444 -35.975 1.00 61.20 C
ATOM 4662 CD2 LEU A 339 -12.667 22.960 -33.980 1.00 55.62 C
ATOM 4663 H LEU A 339 -8.496 22.068 -34.543 1.00 52.02 H
ATOM 4664 HA LEU A 339 -10.648 22.376 -32.504 1.00 52.69 H
ATOM 4665 HB2 LEU A 339 -10.353 20.554 -34.897 1.00 53.30 H
ATOM 4666 HB3 LEU A 339 -11.631 20.401 -33.733 1.00 53.30 H
ATOM 4667 HG LEU A 339 -11.176 22.838 -35.474 1.00 59.15 H
ATOM 4668 HD11 LEU A 339 -12.548 21.877 -36.964 1.00 61.20 H
ATOM 4669 HD12 LEU A 339 -12.468 20.382 -36.061 1.00 61.20 H
ATOM 4670 HD13 LEU A 339 -13.765 21.513 -35.751 1.00 61.20 H
ATOM 4671 HD21 LEU A 339 -13.614 23.275 -34.419 1.00 55.62 H
ATOM 4672 HD22 LEU A 339 -12.903 22.412 -33.067 1.00 55.62 H
ATOM 4673 HD23 LEU A 339 -12.143 23.873 -33.693 1.00 55.62 H
ATOM 4674 N ASP A 340 -8.211 20.499 -32.044 1.00 51.25 N
ATOM 4675 CA ASP A 340 -7.663 19.574 -31.062 1.00 50.20 C

ATOM 4676 C ASP A 340 -8.004 20.050 -29.655 1.00 55.64 C
ATOM 4677 O ASP A 340 -8.012 21.251 -29.377 1.00 59.44 O
ATOM 4678 CB ASP A 340 -6.148 19.454 -31.230 1.00 50.12 C
ATOM 4679 CG ASP A 340 -5.505 18.603 -30.149 1.00 54.70 C
ATOM 4680 OD1 ASP A 340 -5.577 17.359 -30.244 1.00 54.79 O
ATOM 4681 OD2 ASP A 340 -4.931 19.180 -29.202 1.00 55.29 O1-
ATOM 4682 H ASP A 340 -7.561 21.113 -32.517 1.00 51.25 H
ATOM 4683 HA ASP A 340 -8.105 18.589 -31.228 1.00 50.20 H
ATOM 4684 HB2 ASP A 340 -5.913 18.996 -32.190 1.00 50.12 H
ATOM 4685 HB3 ASP A 340 -5.646 20.405 -31.276 1.00 50.12 H
ATOM 4686 N GLU A 341 -8.279 19.096 -28.762 1.00 61.54 N
ATOM 4687 CA GLU A 341 -8.791 19.438 -27.436 1.00 69.20 C
ATOM 4688 C GLU A 341 -7.788 20.271 -26.645 1.00 65.00 C
ATOM 4689 O GLU A 341 -8.147 21.289 -26.043 1.00 66.97 O
ATOM 4690 CB GLU A 341 -9.154 18.164 -26.672 1.00 87.63 C
ATOM 4691 CG GLU A 341 -10.373 18.308 -25.775 1.00102.93 C
ATOM 4692 CD GLU A 341 -11.676 18.131 -26.533 1.00110.21 C
ATOM 4693 OE1 GLU A 341 -12.287 17.047 -26.420 1.00112.37 O
ATOM 4694 OE2 GLU A 341 -12.083 19.069 -27.251 1.00111.51 O1-
ATOM 4695 H GLU A 341 -8.234 18.119 -29.012 1.00 61.54 H
ATOM 4696 HA GLU A 341 -9.687 20.042 -27.591 1.00 69.20 H
ATOM 4697 HB2 GLU A 341 -9.319 17.339 -27.368 1.00 87.63 H
ATOM 4698 HB3 GLU A 341 -8.316 17.831 -26.058 1.00 87.63 H
ATOM 4699 HG2 GLU A 341 -10.334 17.548 -24.994 1.00102.93 H
ATOM 4700 HG3 GLU A 341 -10.372 19.270 -25.262 1.00102.93 H
ATOM 4701 N ASN A 342 -6.521 19.849 -26.626 1.00 55.81 N
ATOM 4702 CA ASN A 342 -5.515 20.580 -25.863 1.00 59.45 C
ATOM 4703 C ASN A 342 -5.040 21.825 -26.603 1.00 64.98 C
ATOM 4704 O ASN A 342 -4.878 22.888 -25.991 1.00 68.28 O
ATOM 4705 CB ASN A 342 -4.329 19.668 -25.541 1.00 60.27 C
ATOM 4706 CG ASN A 342 -4.690 18.561 -24.573 1.00 58.49 C
ATOM 4707 ND2 ASN A 342 -4.775 17.334 -25.077 1.00 58.27 N
ATOM 4708 OD1 ASN A 342 -4.885 18.805 -23.385 1.00 60.81 O
ATOM 4709 H ASN A 342 -6.223 19.048 -27.166 1.00 55.81 H
ATOM 4710 HA ASN A 342 -5.933 20.871 -24.914 1.00 59.45 H
ATOM 4711 HB2 ASN A 342 -3.900 19.253 -26.456 1.00 60.27 H
ATOM 4712 HB3 ASN A 342 -3.533 20.248 -25.071 1.00 60.27 H
ATOM 4713 HD21 ASN A 342 -4.606 17.168 -26.059 1.00 58.27 H
ATOM 4714 HD22 ASN A 342 -5.012 16.558 -24.477 1.00 58.27 H
ATOM 4715 N PHE A 343 -4.806 21.707 -27.915 1.00 58.15 N

ATOM 4716 CA PHE A 343 -4.370 22.853 -28.710 1.00 67.35 C
ATOM 4717 C PHE A 343 -5.390 23.983 -28.650 1.00 75.79 C
ATOM 4718 O PHE A 343 -5.022 25.164 -28.658 1.00 79.57 O
ATOM 4719 CB PHE A 343 -4.130 22.409 -30.155 1.00 62.08 C
ATOM 4720 CG PHE A 343 -3.437 23.437 -31.014 1.00 61.49 C
ATOM 4721 CD1 PHE A 343 -4.151 24.468 -31.603 1.00 65.05 C
ATOM 4722 CD2 PHE A 343 -2.076 23.349 -31.261 1.00 59.92 C
ATOM 4723 CE1 PHE A 343 -3.523 25.403 -32.401 1.00 63.36 C
ATOM 4724 CE2 PHE A 343 -1.440 24.282 -32.061 1.00 59.42 C
ATOM 4725 CZ PHE A 343 -2.166 25.310 -32.633 1.00 48.89 C
ATOM 4726 H PHE A 343 -4.923 20.817 -28.392 1.00 58.15 H
ATOM 4727 HA PHE A 343 -3.426 23.211 -28.294 1.00 67.35 H
ATOM 4728 HB2 PHE A 343 -3.572 21.472 -30.189 1.00 62.08 H
ATOM 4729 HB3 PHE A 343 -5.106 22.221 -30.576 1.00 62.08 H
ATOM 4730 HD1 PHE A 343 -5.215 24.553 -31.438 1.00 65.05 H
ATOM 4731 HD2 PHE A 343 -1.499 22.547 -30.823 1.00 59.92 H
ATOM 4732 HE1 PHE A 343 -4.092 26.204 -32.846 1.00 63.36 H
ATOM 4733 HE2 PHE A 343 -0.395 24.210 -32.232 1.00 59.42 H
ATOM 4734 HZ PHE A 343 -1.677 26.038 -33.260 1.00 48.89 H
ATOM 4735 N LYS A 344 -6.677 23.636 -28.578 1.00 78.56 N
ATOM 4736 CA LYS A 344 -7.730 24.645 -28.510 1.00 83.34 C
ATOM 4737 C LYS A 344 -7.545 25.549 -27.298 1.00 86.07 C
ATOM 4738 O LYS A 344 -7.657 26.776 -27.401 1.00 93.20 O
ATOM 4739 CB LYS A 344 -9.096 23.959 -28.461 1.00 84.70 C
ATOM 4740 CG LYS A 344 -10.252 24.779 -29.006 1.00 88.19 C
ATOM 4741 CD LYS A 344 -11.568 24.025 -28.864 1.00 91.41 C
ATOM 4742 CE LYS A 344 -11.520 22.677 -29.569 1.00 91.48 C
ATOM 4743 NZ LYS A 344 -12.789 21.914 -29.394 1.00 95.26 N1+
ATOM 4744 H LYS A 344 -6.961 22.665 -28.603 1.00 78.56 H
ATOM 4745 HA LYS A 344 -7.670 25.257 -29.413 1.00 83.34 H
ATOM 4746 HB2 LYS A 344 -9.032 23.205 -29.219 1.00 84.70 H
ATOM 4747 HB3 LYS A 344 -9.304 23.480 -27.502 1.00 84.70 H
ATOM 4748 HG2 LYS A 344 -10.321 25.723 -28.465 1.00 88.19 H
ATOM 4749 HG3 LYS A 344 -10.079 25.040 -30.050 1.00 88.19 H
ATOM 4750 HD2 LYS A 344 -11.798 23.887 -27.807 1.00 91.41 H
ATOM 4751 HD3 LYS A 344 -12.375 24.630 -29.279 1.00 91.41 H
ATOM 4752 HE2 LYS A 344 -11.347 22.832 -30.626 1.00 91.48 H
ATOM 4753 HE3 LYS A 344 -10.743 22.018 -29.185 1.00 91.48 H
ATOM 4754 HZ1 LYS A 344 -13.563 22.448 -29.760 1.00 95.26 H
ATOM 4755 HZ2 LYS A 344 -12.715 21.035 -29.887 1.00 95.26 H

ATOM 4756 HZ3 LYS A 344 -12.933 21.724 -28.412 1.00 95.26 H
ATOM 4757 N ARG A 345 -7.240 24.955 -26.141 1.00 93.57 N
ATOM 4758 CA ARG A 345 -7.148 25.717 -24.899 1.00 93.28 C
ATOM 4759 C ARG A 345 -6.035 26.755 -24.955 1.00 98.22 C
ATOM 4760 O ARG A 345 -6.197 27.877 -24.459 1.00 99.54 O
ATOM 4761 CB ARG A 345 -6.925 24.766 -23.724 1.00 92.68 C
ATOM 4762 CG ARG A 345 -8.018 23.723 -23.555 1.00 94.35 C
ATOM 4763 CD ARG A 345 -7.499 22.444 -22.900 1.00 96.66 C
ATOM 4764 NE ARG A 345 -6.785 22.694 -21.651 1.00104.45 N
ATOM 4765 CZ ARG A 345 -5.473 22.546 -21.496 1.00104.25 C
ATOM 4766 NH1 ARG A 345 -4.723 22.142 -22.512 1.00 98.59 N1+
ATOM 4767 NH2 ARG A 345 -4.910 22.797 -20.321 1.00112.71 N1+
ATOM 4768 H ARG A 345 -7.114 23.954 -26.091 1.00 93.57 H
ATOM 4769 HA ARG A 345 -8.094 26.241 -24.745 1.00 93.28 H
ATOM 4770 HB2 ARG A 345 -5.973 24.259 -23.890 1.00 92.68 H
ATOM 4771 HB3 ARG A 345 -6.810 25.322 -22.792 1.00 92.68 H
ATOM 4772 HG2 ARG A 345 -8.925 24.115 -23.093 1.00 94.35 H
ATOM 4773 HG3 ARG A 345 -8.323 23.398 -24.547 1.00 94.35 H
ATOM 4774 HD2 ARG A 345 -8.400 21.971 -22.509 1.00 96.66 H
ATOM 4775 HD3 ARG A 345 -7.105 21.685 -23.552 1.00 96.66 H
ATOM 4776 HE ARG A 345 -7.340 23.021 -20.873 1.00104.45 H
ATOM 4777 HH11 ARG A 345 -3.725 22.037 -22.408 1.00 98.59 H
ATOM 4778 HH12 ARG A 345 -5.153 21.911 -23.396 1.00 98.59 H
ATOM 4779 HH21 ARG A 345 -5.476 23.098 -19.541 1.00112.71 H
ATOM 4780 HH22 ARG A 345 -3.914 22.691 -20.194 1.00112.71 H
ATOM 4781 N CYS A 346 -4.898 26.402 -25.555 1.00103.09 N
ATOM 4782 CA CYS A 346 -3.748 27.298 -25.548 1.00113.60 C
ATOM 4783 C CYS A 346 -3.944 28.511 -26.448 1.00119.03 C
ATOM 4784 O CYS A 346 -3.238 29.509 -26.266 1.00122.36 O
ATOM 4785 CB CYS A 346 -2.494 26.538 -25.975 1.00113.30 C
ATOM 4786 SG CYS A 346 -2.175 25.035 -25.028 1.00115.03 S
ATOM 4787 H CYS A 346 -4.791 25.492 -25.981 1.00103.09 H
ATOM 4788 HA CYS A 346 -3.588 27.658 -24.529 1.00113.60 H
ATOM 4789 HB2 CYS A 346 -2.570 26.247 -27.024 1.00113.30 H
ATOM 4790 HB3 CYS A 346 -1.615 27.179 -25.896 1.00113.30 H
ATOM 4791 HG CYS A 346 -3.207 24.336 -25.511 1.00115.03 H
ATOM 4792 N PHE A 347 -4.909 28.453 -27.369 1.00118.10 N
ATOM 4793 CA PHE A 347 -5.073 29.387 -28.498 1.00119.97 C
ATOM 4794 C PHE A 347 -3.869 30.297 -28.764 1.00125.27 C
ATOM 4795 O PHE A 347 -3.939 31.516 -28.608 1.00130.00 O

```
ATOM   4796  CB  PHE A 347      -6.346  30.243 -28.334  1.00118.65           C
ATOM   4797  CG  PHE A 347      -6.597  30.750 -26.936  1.00121.96           C
ATOM   4798  CD1 PHE A 347      -7.522  30.125 -26.116  1.00121.92           C
ATOM   4799  CD2 PHE A 347      -5.950  31.880 -26.461  1.00123.35           C
ATOM   4800  CE1 PHE A 347      -7.771  30.596 -24.840  1.00124.65           C
ATOM   4801  CE2 PHE A 347      -6.193  32.354 -25.187  1.00124.88           C
ATOM   4802  CZ  PHE A 347      -7.104  31.712 -24.377  1.00127.32           C
ATOM   4803  H   PHE A 347      -5.571  27.689 -27.335  1.00118.10           H
ATOM   4804  HA  PHE A 347      -5.189  28.774 -29.393  1.00119.97           H
ATOM   4805  HB2 PHE A 347      -6.404  31.068 -29.047  1.00118.65           H
ATOM   4806  HB3 PHE A 347      -7.196  29.611 -28.597  1.00118.65           H
ATOM   4807  HD1 PHE A 347      -8.051  29.250 -26.463  1.00121.92           H
ATOM   4808  HD2 PHE A 347      -5.287  32.468 -27.066  1.00123.35           H
ATOM   4809  HE1 PHE A 347      -8.486  30.090 -24.208  1.00124.65           H
ATOM   4810  HE2 PHE A 347      -5.684  33.239 -24.833  1.00124.88           H
ATOM   4811  HZ  PHE A 347      -7.298  32.083 -23.382  1.00127.32           H
ATOM   4812  HXT PHE A 347      -2.952  29.794 -29.071  1.00125.27           H
TER    4813      PHE A 347
HETATM 4814  C1  UNK     0       3.421  15.181 -60.379  1.00  0.00           C
HETATM 4815  N1  UNK     0       2.871  17.197 -58.892  1.00  0.00           N1+
HETATM 4816  O1  UNK     0       0.113  17.950 -51.199  1.00  0.00           O
HETATM 4817  C2  UNK     0       3.961  16.209 -59.375  1.00  0.00           C
HETATM 4818  N2  UNK     0      -0.508  15.450 -58.148  1.00  0.00           N
HETATM 4819  C3  UNK     0       2.462  18.245 -59.955  1.00  0.00           C
HETATM 4820  N3  UNK     0      -2.167  15.035 -56.676  1.00  0.00           N
HETATM 4821  C4  UNK     0       1.770  19.444 -59.288  1.00  0.00           C
HETATM 4822  C5  UNK     0       1.716  16.546 -58.086  1.00  0.00           C
HETATM 4823  C6  UNK     0       0.476  16.190 -58.938  1.00  0.00           C
HETATM 4824  C7  UNK     0      -0.780  14.098 -58.251  1.00  0.00           C
HETATM 4825  C8  UNK     0      -0.243  13.057 -59.022  1.00  0.00           C
HETATM 4826  C9  UNK     0      -0.817  11.793 -58.852  1.00  0.00           C
HETATM 4827  C10 UNK     0      -1.888  11.578 -57.984  1.00  0.00           C
HETATM 4828  C11 UNK     0      -2.418  12.618 -57.216  1.00  0.00           C
HETATM 4829  C12 UNK     0      -1.844  13.887 -57.339  1.00  0.00           C
HETATM 4830  C13 UNK     0      -1.356  15.942 -57.165  1.00  0.00           C
HETATM 4831  C14 UNK     0      -1.376  17.383 -56.680  1.00  0.00           C
HETATM 4832  C15 UNK     0      -1.002  17.535 -55.209  1.00  0.00           C
HETATM 4833  C16 UNK     0       0.255  17.134 -54.750  1.00  0.00           C
HETATM 4834  C17 UNK     0       0.612  17.345 -53.424  1.00  0.00           C
HETATM 4835  C18 UNK     0      -0.295  17.881 -52.518  1.00  0.00           C
```

HETATM 4836 C19 UNK   0   -0.807 18.356 -50.186 1.00 0.00   C
HETATM 4837 C20 UNK   0   -0.401 17.660 -48.884 1.00 0.00   C
HETATM 4838 C21 UNK   0   -1.545 18.297 -52.987 1.00 0.00   C
HETATM 4839 C22 UNK   0   -1.895 18.136 -54.324 1.00 0.00   C
HETATM 4840 H1 UNK   0   4.242 14.642 -60.850 1.00 0.00   H
HETATM 4841 H2 UNK   0   2.853 15.651 -61.180 1.00 0.00   H
HETATM 4842 H3 UNK   0   2.786 14.443 -59.890 1.00 0.00   H
HETATM 4843 H4 UNK   0   4.353 15.742 -58.470 1.00 0.00   H
HETATM 4844 H5 UNK   0   4.751 16.830 -59.800 1.00 0.00   H
HETATM 4845 H6 UNK   0   3.392 18.563 -60.429 1.00 0.00   H
HETATM 4846 H7 UNK   0   1.843 17.740 -60.698 1.00 0.00   H
HETATM 4847 H8 UNK   0   1.528 20.214 -60.021 1.00 0.00   H
HETATM 4848 H9 UNK   0   0.841 19.181 -58.785 1.00 0.00   H
HETATM 4849 H10 UNK   0   2.414 19.903 -58.536 1.00 0.00   H
HETATM 4850 H11 UNK   0   2.147 15.696 -57.553 1.00 0.00   H
HETATM 4851 H12 UNK   0   1.484 17.325 -57.364 1.00 0.00   H
HETATM 4852 H13 UNK   0   -0.017 17.078 -59.331 1.00 0.00   H
HETATM 4853 H14 UNK   0   0.747 15.585 -59.801 1.00 0.00   H
HETATM 4854 H15 UNK   0   0.581 13.211 -59.702 1.00 0.00   H
HETATM 4855 H16 UNK   0   -0.421 10.955 -59.409 1.00 0.00   H
HETATM 4856 H17 UNK   0   -2.298 10.582 -57.892 1.00 0.00   H
HETATM 4857 H18 UNK   0   -3.228 12.454 -56.523 1.00 0.00   H
HETATM 4858 H19 UNK   0   -0.699 18.018 -57.248 1.00 0.00   H
HETATM 4859 H20 UNK   0   -2.363 17.811 -56.859 1.00 0.00   H
HETATM 4860 H21 UNK   0   0.976 16.680 -55.410 1.00 0.00   H
HETATM 4861 H22 UNK   0   1.611 17.107 -53.102 1.00 0.00   H
HETATM 4862 H23 UNK   0   -0.775 19.441 -50.095 1.00 0.00   H
HETATM 4863 H24 UNK   0   -1.843 18.085 -50.387 1.00 0.00   H
HETATM 4864 H25 UNK   0   -1.051 17.940 -48.058 1.00 0.00   H
HETATM 4865 H26 UNK   0   -0.500 16.581 -48.975 1.00 0.00   H
HETATM 4866 H27 UNK   0   0.627 17.891 -48.607 1.00 0.00   H
HETATM 4867 H28 UNK   0   -2.238 18.777 -52.327 1.00 0.00   H
HETATM 4868 H29 UNK   0   -2.845 18.503 -54.679 1.00 0.00   H
HETATM 4869 H30 UNK   0   3.350 17.747 -58.186 1.00 0.00   H
CONECT 4814 4817 4840 4841 4842
CONECT 4815 4817 4819 4822 4869
CONECT 4816 4835 4836
CONECT 4817 4814 4815 4843 4844
CONECT 4818 4823
CONECT 4818 4824

CONECT 4818 4824
CONECT 4818 4824
CONECT 4818 4824
CONECT 4818 4830
CONECT 4818 4830
CONECT 4818 4830
CONECT 4818 4830
CONECT 4819 4815 4821 4845 4846
CONECT 4820 4829
CONECT 4820 4829
CONECT 4820 4829
CONECT 4820 4829
CONECT 4820 4830
CONECT 4820 4830
CONECT 4820 4830
CONECT 4820 4830
CONECT 4821 4819 4847 4848 4849
CONECT 4822 4815 4823 4850 4851
CONECT 4823 4818 4822 4852 4853
CONECT 4824 4818
CONECT 4824 4818
CONECT 4824 4818
CONECT 4824 4818
CONECT 4824 4825
CONECT 4824 4825
CONECT 4824 4825
CONECT 4824 4825
CONECT 4824 4829
CONECT 4824 4829
CONECT 4824 4829
CONECT 4824 4829
CONECT 4825 4824
CONECT 4825 4824
CONECT 4825 4824
CONECT 4825 4824
CONECT 4825 4826
CONECT 4825 4826
CONECT 4825 4826
CONECT 4825 4826
CONECT 4825 4854

CONECT 4826 4825
CONECT 4826 4825
CONECT 4826 4825
CONECT 4826 4825
CONECT 4826 4827
CONECT 4826 4827
CONECT 4826 4827
CONECT 4826 4827
CONECT 4826 4855
CONECT 4827 4826
CONECT 4827 4826
CONECT 4827 4826
CONECT 4827 4826
CONECT 4827 4828
CONECT 4827 4828
CONECT 4827 4828
CONECT 4827 4828
CONECT 4827 4856
CONECT 4828 4827
CONECT 4828 4827
CONECT 4828 4827
CONECT 4828 4827
CONECT 4828 4829
CONECT 4828 4829
CONECT 4828 4829
CONECT 4828 4829
CONECT 4828 4857
CONECT 4829 4820
CONECT 4829 4820
CONECT 4829 4820
CONECT 4829 4820
CONECT 4829 4824
CONECT 4829 4824
CONECT 4829 4824
CONECT 4829 4824
CONECT 4829 4828
CONECT 4829 4828
CONECT 4829 4828
CONECT 4829 4828
CONECT 4830 4818

CONECT 4830 4818
CONECT 4830 4818
CONECT 4830 4818
CONECT 4830 4820
CONECT 4830 4820
CONECT 4830 4820
CONECT 4830 4820
CONECT 4830 4831
CONECT 4831 4830 4832 4858 4859
CONECT 4832 4831
CONECT 4832 4833
CONECT 4832 4833
CONECT 4832 4833
CONECT 4832 4833
CONECT 4832 4839
CONECT 4832 4839
CONECT 4832 4839
CONECT 4832 4839
CONECT 4833 4832
CONECT 4833 4832
CONECT 4833 4832
CONECT 4833 4832
CONECT 4833 4834
CONECT 4833 4834
CONECT 4833 4834
CONECT 4833 4834
CONECT 4833 4860
CONECT 4834 4833
CONECT 4834 4833
CONECT 4834 4833
CONECT 4834 4833
CONECT 4834 4835
CONECT 4834 4835
CONECT 4834 4835
CONECT 4834 4835
CONECT 4834 4861
CONECT 4835 4816
CONECT 4835 4834
CONECT 4835 4834
CONECT 4835 4834

CONECT 4835 4834
CONECT 4835 4838
CONECT 4835 4838
CONECT 4835 4838
CONECT 4835 4838
CONECT 4836 4816 4837 4862 4863
CONECT 4837 4836 4864 4865 4866
CONECT 4838 4835
CONECT 4838 4835
CONECT 4838 4835
CONECT 4838 4835
CONECT 4838 4839
CONECT 4838 4839
CONECT 4838 4839
CONECT 4838 4839
CONECT 4838 4867
CONECT 4839 4832
CONECT 4839 4832
CONECT 4839 4832
CONECT 4839 4832
CONECT 4839 4838
CONECT 4839 4838
CONECT 4839 4838
CONECT 4839 4838
CONECT 4839 4868
CONECT 4840 4814
CONECT 4841 4814
CONECT 4842 4814
CONECT 4843 4817
CONECT 4844 4817
CONECT 4845 4819
CONECT 4846 4819
CONECT 4847 4821
CONECT 4848 4821
CONECT 4849 4821
CONECT 4850 4822
CONECT 4851 4822
CONECT 4852 4823
CONECT 4853 4823
CONECT 4854 4825

CONECT 4855 4826
CONECT 4856 4827
CONECT 4857 4828
CONECT 4858 4831
CONECT 4859 4831
CONECT 4860 4833
CONECT 4861 4834
CONECT 4862 4836
CONECT 4863 4836
CONECT 4864 4837
CONECT 4865 4837
CONECT 4866 4837
CONECT 4867 4838
CONECT 4868 4839
CONECT 4869 4815
END

Table S3. The 3D coordinates of the minimized structure of MOR with M6.

ATOM 1 N GLY A 52 5.641 11.638 -72.150 1.00110.27 N1+
ATOM 2 CA GLY A 52 4.612 11.828 -71.143 1.00107.88 C
ATOM 3 C GLY A 52 4.546 13.235 -70.580 1.00106.83 C
ATOM 4 O GLY A 52 5.549 13.777 -70.112 1.00102.57 O
ATOM 5 HA2 GLY A 52 3.684 11.499 -71.610 1.00107.88 H
ATOM 6 HA3 GLY A 52 4.887 11.120 -70.360 1.00107.88 H
ATOM 7 H1 GLY A 52 5.666 10.675 -72.456 1.00110.27 H
ATOM 8 H2 GLY A 52 5.476 12.217 -72.960 1.00110.27 H
ATOM 9 H3 GLY A 52 6.551 11.862 -71.773 1.00110.27 H
ATOM 10 N ARG A 53 3.355 13.827 -70.628 1.00107.33 N
ATOM 11 CA ARG A 53 3.135 15.162 -70.092 1.00107.30 C
ATOM 12 C ARG A 53 2.948 15.108 -68.582 1.00 97.40 C
ATOM 13 O ARG A 53 2.361 14.167 -68.040 1.00101.33 O
ATOM 14 CB ARG A 53 1.869 15.738 -70.784 1.00 0.00 C
ATOM 15 CG ARG A 53 0.541 15.023 -70.422 1.00 0.00 C
ATOM 16 CD ARG A 53 -0.657 15.440 -71.285 1.00 0.00 C
ATOM 17 NE ARG A 53 -1.850 14.622 -71.007 1.00 0.00 N
ATOM 18 CZ ARG A 53 -2.077 13.365 -71.424 1.00 0.00 C
ATOM 19 NH1 ARG A 53 -1.200 12.725 -72.204 1.00 0.00 N1+
ATOM 20 NH2 ARG A 53 -3.198 12.740 -71.048 1.00 0.00 N1+
ATOM 21 H ARG A 53 2.550 13.344 -70.998 1.00107.33 H
ATOM 22 HA ARG A 53 3.997 15.793 -70.321 1.00107.30 H
ATOM 23 HB2 ARG A 53 1.775 16.799 -70.545 1.00 0.00 H
ATOM 24 HB3 ARG A 53 2.023 15.696 -71.863 1.00 0.00 H
ATOM 25 HG2 ARG A 53 0.635 13.937 -70.421 1.00 0.00 H
ATOM 26 HG3 ARG A 53 0.309 15.298 -69.392 1.00 0.00 H
ATOM 27 HD2 ARG A 53 -0.956 16.430 -70.946 1.00 0.00 H
ATOM 28 HD3 ARG A 53 -0.422 15.534 -72.346 1.00 0.00 H
ATOM 29 HE ARG A 53 -2.535 15.065 -70.412 1.00 0.00 H
ATOM 30 HH11 ARG A 53 -0.361 13.190 -72.515 1.00 0.00 H
ATOM 31 HH12 ARG A 53 -1.372 11.781 -72.518 1.00 0.00 H
ATOM 32 HH21 ARG A 53 -3.895 13.214 -70.495 1.00 0.00 H
ATOM 33 HH22 ARG A 53 -3.369 11.773 -71.291 1.00 0.00 H
ATOM 34 N ASP A 54 3.451 16.137 -67.902 1.00 90.90 N
ATOM 35 CA ASP A 54 3.338 16.212 -66.450 1.00 84.22 C

```
ATOM     36  C   ASP A  54       2.840  17.587 -66.021  1.00 72.09           C
ATOM     37  O   ASP A  54       1.631  17.799 -65.885  1.00 72.61           O
ATOM     38  CB  ASP A  54       4.670  15.778 -65.774  1.00  0.00           C
ATOM     39  CG  ASP A  54       4.638  15.629 -64.246  1.00  0.00           C
ATOM     40  OD1 ASP A  54       3.536  15.683 -63.659  1.00  0.00           O
ATOM     41  OD2 ASP A  54       5.731  15.398 -63.687  1.00  0.00           O1-
ATOM     42  H   ASP A  54       3.955  16.879 -68.369  1.00 90.90           H
ATOM     43  HA  ASP A  54       2.571  15.525 -66.090  1.00 84.22           H
ATOM     44  HB2 ASP A  54       4.956  14.806 -66.177  1.00  0.00           H
ATOM     45  HB3 ASP A  54       5.455  16.486 -66.036  1.00  0.00           H
ATOM     46  N   SER A  55       3.764  18.526 -65.803  1.00 72.40           N
ATOM     47  CA  SER A  55       3.367  19.877 -65.424  1.00 79.19           C
ATOM     48  C   SER A  55       2.828  20.654 -66.618  1.00 85.33           C
ATOM     49  O   SER A  55       1.866  21.419 -66.481  1.00 86.66           O
ATOM     50  CB  SER A  55       4.560  20.561 -64.715  1.00 81.52           C
ATOM     51  OG  SER A  55       5.734  20.624 -65.510  1.00 87.70           O
ATOM     52  H   SER A  55       4.749  18.335 -65.926  1.00 72.40           H
ATOM     53  HA  SER A  55       2.584  19.819 -64.664  1.00 79.19           H
ATOM     54  HB2 SER A  55       4.286  21.570 -64.407  1.00 81.52           H
ATOM     55  HB3 SER A  55       4.797  20.023 -63.795  1.00 81.52           H
ATOM     56  HG  SER A  55       6.255  19.822 -65.320  1.00 87.70           H
ATOM     57  N   LEU A  56       3.432  20.473 -67.787  1.00 84.34           N
ATOM     58  CA  LEU A  56       2.998  21.175 -68.987  1.00 84.78           C
ATOM     59  C   LEU A  56       1.677  20.625 -69.503  1.00 85.67           C
ATOM     60  O   LEU A  56       1.599  19.481 -69.951  1.00 83.26           O
ATOM     61  CB  LEU A  56       4.092  21.083 -70.070  1.00 85.66           C
ATOM     62  CG  LEU A  56       3.814  21.862 -71.374  1.00 87.52           C
ATOM     63  CD1 LEU A  56       3.682  23.376 -71.149  1.00 87.52           C
ATOM     64  CD2 LEU A  56       4.895  21.551 -72.419  1.00 90.95           C
ATOM     65  H   LEU A  56       4.237  19.862 -67.869  1.00 84.34           H
ATOM     66  HA  LEU A  56       2.874  22.229 -68.730  1.00 84.78           H
ATOM     67  HB2 LEU A  56       5.038  21.430 -69.653  1.00 85.66           H
ATOM     68  HB3 LEU A  56       4.228  20.031 -70.322  1.00 85.66           H
ATOM     69  HG  LEU A  56       2.876  21.505 -71.802  1.00 87.52           H
ATOM     70 HD11 LEU A  56       4.213  23.937 -71.919  1.00 87.52           H
ATOM     71 HD12 LEU A  56       2.639  23.691 -71.193  1.00 87.52           H
ATOM     72 HD13 LEU A  56       4.087  23.694 -70.188  1.00 87.52           H
ATOM     73 HD21 LEU A  56       4.507  21.656 -73.433  1.00 90.95           H
ATOM     74 HD22 LEU A  56       5.755  22.216 -72.323  1.00 90.95           H
ATOM     75 HD23 LEU A  56       5.260  20.534 -72.316  1.00 90.95           H
```

```
ATOM     76  N   CYS A  57       0.642  21.453 -69.436  1.00 90.27           N
ATOM     77  CA  CYS A  57      -0.660  21.086 -69.924  1.00100.97           C
ATOM     78  C   CYS A  57      -0.870  21.460 -71.386  1.00113.61           C
ATOM     79  O   CYS A  57      -0.693  22.610 -71.800  1.00110.96           O
ATOM     80  CB  CYS A  57      -1.740  21.856 -69.129  1.00  0.00           C
ATOM     81  SG  CYS A  57      -1.666  21.488 -67.353  1.00  0.00           S
ATOM     82  H   CYS A  57       0.749  22.382 -69.056  1.00 90.27           H
ATOM     83  HA  CYS A  57      -0.790  20.022 -69.745  1.00100.97           H
ATOM     84  HB2 CYS A  57      -1.634  22.935 -69.262  1.00  0.00           H
ATOM     85  HB3 CYS A  57      -2.738  21.594 -69.487  1.00  0.00           H
ATOM     86  HG  CYS A  57      -2.083  20.221 -67.456  1.00  0.00           H
ATOM     87  N   PRO A  58      -1.243  20.464 -72.205  1.00124.15           N
ATOM     88  CA  PRO A  58      -1.641  20.732 -73.590  1.00135.47           C
ATOM     89  C   PRO A  58      -2.849  21.662 -73.630  1.00143.48           C
ATOM     90  O   PRO A  58      -3.984  21.211 -73.479  1.00143.53           O
ATOM     91  CB  PRO A  58      -1.985  19.342 -74.136  1.00134.79           C
ATOM     92  CG  PRO A  58      -1.220  18.397 -73.270  1.00129.71           C
ATOM     93  CD  PRO A  58      -1.214  19.021 -71.907  1.00126.49           C
ATOM     94  HA  PRO A  58      -0.791  21.141 -74.140  1.00135.47           H
ATOM     95  HB2 PRO A  58      -3.041  19.086 -74.045  1.00134.79           H
ATOM     96  HB3 PRO A  58      -1.726  19.234 -75.190  1.00134.79           H
ATOM     97  HG2 PRO A  58      -1.668  17.406 -73.297  1.00129.71           H
ATOM     98  HG3 PRO A  58      -0.196  18.320 -73.639  1.00129.71           H
ATOM     99  HD2 PRO A  58      -2.097  18.746 -71.328  1.00126.49           H
ATOM    100  HD3 PRO A  58      -0.305  18.735 -71.384  1.00126.49           H
ATOM    101  N   PRO A  59      -2.596  22.956 -73.821  1.00149.23           N
ATOM    102  CA  PRO A  59      -3.615  23.983 -73.663  1.00155.25           C
ATOM    103  C   PRO A  59      -4.064  24.617 -74.970  1.00153.72           C
ATOM    104  O   PRO A  59      -4.914  25.514 -74.933  1.00158.07           O
ATOM    105  CB  PRO A  59      -3.107  25.090 -72.721  1.00  0.00           C
ATOM    106  CG  PRO A  59      -1.645  25.187 -73.004  1.00  0.00           C
ATOM    107  CD  PRO A  59      -1.432  23.865 -73.762  1.00  0.00           C
ATOM    108  HA  PRO A  59      -4.526  23.582 -73.215  1.00155.25           H
ATOM    109  HB2 PRO A  59      -3.613  26.053 -72.810  1.00  0.00           H
ATOM    110  HB3 PRO A  59      -3.250  24.757 -71.692  1.00  0.00           H
ATOM    111  HG2 PRO A  59      -1.397  26.046 -73.630  1.00  0.00           H
ATOM    112  HG3 PRO A  59      -1.065  25.248 -72.083  1.00  0.00           H
ATOM    113  HD2 PRO A  59      -1.368  24.203 -74.796  1.00  0.00           H
ATOM    114  HD3 PRO A  59      -0.420  23.462 -73.799  1.00  0.00           H
ATOM    115  N   THR A  60      -3.526  24.173 -76.110  1.00149.99           N
```

ATOM 116 CA THR A 60 -3.749 24.778 -77.423 1.00148.17 C
ATOM 117 C THR A 60 -5.177 25.279 -77.601 1.00148.90 C
ATOM 118 O THR A 60 -6.143 24.544 -77.371 1.00148.23 O
ATOM 119 CB THR A 60 -3.399 23.784 -78.533 1.00146.14 C
ATOM 120 CG2 THR A 60 -3.132 24.522 -79.837 1.00147.10 C
ATOM 121 OG1 THR A 60 -2.220 23.059 -78.165 1.00142.32 O
ATOM 122 H THR A 60 -2.854 23.422 -76.079 1.00149.99 H
ATOM 123 HA THR A 60 -3.068 25.630 -77.477 1.00148.17 H
ATOM 124 HB THR A 60 -4.201 23.057 -78.676 1.00146.14 H
ATOM 125 HG1 THR A 60 -2.472 22.361 -77.555 1.00142.32 H
ATOM 126 HG21 THR A 60 -2.835 23.824 -80.620 1.00147.10 H
ATOM 127 HG22 THR A 60 -4.017 25.046 -80.197 1.00147.10 H
ATOM 128 HG23 THR A 60 -2.330 25.253 -79.729 1.00147.10 H
ATOM 129 N GLY A 61 -5.310 26.535 -78.012 1.00149.88 N
ATOM 130 CA GLY A 61 -6.559 27.252 -77.869 1.00150.23 C
ATOM 131 C GLY A 61 -6.469 28.159 -76.659 1.00148.79 C
ATOM 132 O GLY A 61 -7.088 27.891 -75.625 1.00147.77 O
ATOM 133 H GLY A 61 -4.488 27.079 -78.228 1.00149.88 H
ATOM 134 HA2 GLY A 61 -6.717 27.858 -78.762 1.00150.23 H
ATOM 135 HA3 GLY A 61 -7.432 26.602 -77.784 1.00150.23 H
ATOM 136 N SER A 62 -5.682 29.236 -76.790 1.00147.63 N
ATOM 137 CA SER A 62 -5.277 30.147 -75.721 1.00142.05 C
ATOM 138 C SER A 62 -6.404 30.450 -74.741 1.00135.94 C
ATOM 139 O SER A 62 -7.563 30.616 -75.147 1.00137.86 O
ATOM 140 CB SER A 62 -4.742 31.451 -76.317 1.00144.61 C
ATOM 141 OG SER A 62 -5.694 32.049 -77.178 1.00148.40 O
ATOM 142 H SER A 62 -5.256 29.419 -77.686 1.00147.63 H
ATOM 143 HA SER A 62 -4.462 29.637 -75.208 1.00142.05 H
ATOM 144 HB2 SER A 62 -4.471 32.165 -75.538 1.00144.61 H
ATOM 145 HB3 SER A 62 -3.835 31.257 -76.890 1.00144.61 H
ATOM 146 HG SER A 62 -6.474 32.274 -76.663 1.00148.40 H
ATOM 147 N PRO A 63 -6.098 30.545 -73.450 1.00128.05 N
ATOM 148 CA PRO A 63 -7.155 30.699 -72.446 1.00118.94 C
ATOM 149 C PRO A 63 -7.864 32.039 -72.573 1.00113.02 C
ATOM 150 O PRO A 63 -7.345 33.006 -73.134 1.00114.91 O
ATOM 151 CB PRO A 63 -6.397 30.598 -71.119 1.00115.15 C
ATOM 152 CG PRO A 63 -5.015 31.064 -71.451 1.00117.95 C
ATOM 153 CD PRO A 63 -4.753 30.575 -72.848 1.00123.25 C
ATOM 154 HA PRO A 63 -7.885 29.890 -72.524 1.00118.94 H
ATOM 155 HB2 PRO A 63 -6.846 31.167 -70.303 1.00115.15 H

ATOM 156 HB3 PRO A 63 -6.365 29.554 -70.803 1.00115.15 H
ATOM 157 HG2 PRO A 63 -5.000 32.155 -71.446 1.00117.95 H
ATOM 158 HG3 PRO A 63 -4.266 30.727 -70.733 1.00117.95 H
ATOM 159 HD2 PRO A 63 -4.053 31.224 -73.374 1.00123.25 H
ATOM 160 HD3 PRO A 63 -4.349 29.562 -72.830 1.00123.25 H
ATOM 161 N SER A 64 -9.079 32.079 -72.036 1.00107.37 N
ATOM 162 CA SER A 64 -9.865 33.300 -72.012 1.00106.28 C
ATOM 163 C SER A 64 -9.489 34.146 -70.796 1.00122.18 C
ATOM 164 O SER A 64 -8.639 33.777 -69.981 1.00114.78 O
ATOM 165 CB SER A 64 -11.357 32.975 -72.013 1.00106.54 C
ATOM 166 OG SER A 64 -11.723 32.270 -70.841 1.00103.81 O
ATOM 167 H SER A 64 -9.469 31.271 -71.572 1.00107.37 H
ATOM 168 HA SER A 64 -9.666 33.893 -72.907 1.00106.28 H
ATOM 169 HB2 SER A 64 -11.963 33.879 -72.088 1.00106.54 H
ATOM 170 HB3 SER A 64 -11.608 32.365 -72.881 1.00106.54 H
ATOM 171 HG SER A 64 -12.653 32.037 -70.908 1.00103.81 H
ATOM 172 N MET A 65 -10.142 35.302 -70.672 1.00 86.88 N
ATOM 173 CA MET A 65 -9.847 36.213 -69.572 1.00 87.64 C
ATOM 174 C MET A 65 -10.326 35.647 -68.241 1.00 82.86 C
ATOM 175 O MET A 65 -9.552 35.550 -67.281 1.00 79.45 O
ATOM 176 CB MET A 65 -10.492 37.573 -69.839 1.00 96.03 C
ATOM 177 CG MET A 65 -10.392 38.547 -68.679 1.00105.14 C
ATOM 178 SD MET A 65 -8.705 39.117 -68.427 1.00112.18 S
ATOM 179 CE MET A 65 -8.374 39.884 -70.011 1.00103.68 C
ATOM 180 H MET A 65 -10.829 35.593 -71.352 1.00 86.88 H
ATOM 181 HA MET A 65 -8.766 36.355 -69.512 1.00 87.64 H
ATOM 182 HB2 MET A 65 -10.093 38.001 -70.757 1.00 96.03 H
ATOM 183 HB3 MET A 65 -11.553 37.426 -70.050 1.00 96.03 H
ATOM 184 HG2 MET A 65 -10.996 39.425 -68.909 1.00105.14 H
ATOM 185 HG3 MET A 65 -10.796 38.165 -67.742 1.00105.14 H
ATOM 186 HE1 MET A 65 -7.480 40.503 -69.937 1.00103.68 H
ATOM 187 HE2 MET A 65 -8.193 39.136 -70.782 1.00103.68 H
ATOM 188 HE3 MET A 65 -9.204 40.521 -70.317 1.00103.68 H
ATOM 189 N ILE A 66 -11.603 35.264 -68.168 1.00 83.31 N
ATOM 190 CA ILE A 66 -12.193 34.856 -66.897 1.00 87.12 C
ATOM 191 C ILE A 66 -11.557 33.571 -66.381 1.00 81.04 C
ATOM 192 O ILE A 66 -11.418 33.385 -65.165 1.00 78.67 O
ATOM 193 CB ILE A 66 -13.719 34.713 -67.051 1.00 0.00 C
ATOM 194 CG1 ILE A 66 -14.133 33.232 -67.169 1.00 0.00 C
ATOM 195 CG2 ILE A 66 -14.234 35.536 -68.245 1.00 0.00 C

ATOM 196 CD1 ILE A 66 -15.627 32.906 -67.327 1.00 0.00 C
ATOM 197 H ILE A 66 -12.217 35.337 -68.965 1.00 83.31 H
ATOM 198 HA ILE A 66 -11.998 35.642 -66.163 1.00 87.12 H
ATOM 199 HB ILE A 66 -14.180 35.119 -66.150 1.00 0.00 H
ATOM 200 HG12 ILE A 66 -13.643 32.793 -68.036 1.00 0.00 H
ATOM 201 HG13 ILE A 66 -13.859 32.650 -66.288 1.00 0.00 H
ATOM 202 HG21 ILE A 66 -15.323 35.565 -68.242 1.00 0.00 H
ATOM 203 HG22 ILE A 66 -13.901 36.574 -68.197 1.00 0.00 H
ATOM 204 HG23 ILE A 66 -13.940 35.116 -69.208 1.00 0.00 H
ATOM 205 HD11 ILE A 66 -15.827 31.860 -67.091 1.00 0.00 H
ATOM 206 HD12 ILE A 66 -16.235 33.519 -66.662 1.00 0.00 H
ATOM 207 HD13 ILE A 66 -15.963 33.084 -68.348 1.00 0.00 H
ATOM 208 N THR A 67 -11.152 32.671 -67.279 1.00 80.92 N
ATOM 209 CA THR A 67 -10.598 31.394 -66.838 1.00 73.47 C
ATOM 210 C THR A 67 -9.184 31.556 -66.292 1.00 64.70 C
ATOM 211 O THR A 67 -8.847 30.976 -65.253 1.00 57.65 O
ATOM 212 CB THR A 67 -10.617 30.382 -67.985 1.00 72.78 C
ATOM 213 CG2 THR A 67 -12.048 30.098 -68.417 1.00 57.52 C
ATOM 214 OG1 THR A 67 -9.875 30.899 -69.096 1.00 85.80 O
ATOM 215 H THR A 67 -11.243 32.836 -68.271 1.00 80.92 H
ATOM 216 HA THR A 67 -11.219 30.989 -66.035 1.00 73.47 H
ATOM 217 HB THR A 67 -10.147 29.448 -67.672 1.00 72.78 H
ATOM 218 HG1 THR A 67 -10.447 31.484 -69.604 1.00 85.80 H
ATOM 219 HG21 THR A 67 -12.055 29.415 -69.259 1.00 57.52 H
ATOM 220 HG22 THR A 67 -12.618 29.636 -67.611 1.00 57.52 H
ATOM 221 HG23 THR A 67 -12.585 30.989 -68.731 1.00 57.52 H
ATOM 222 N ALA A 68 -8.344 32.337 -66.976 1.00 65.22 N
ATOM 223 CA ALA A 68 -6.972 32.525 -66.513 1.00 65.95 C
ATOM 224 C ALA A 68 -6.937 33.226 -65.160 1.00 66.66 C
ATOM 225 O ALA A 68 -6.132 32.873 -64.290 1.00 62.13 O
ATOM 226 CB ALA A 68 -6.169 33.310 -67.549 1.00 70.78 C
ATOM 227 H ALA A 68 -8.623 32.791 -67.835 1.00 65.22 H
ATOM 228 HA ALA A 68 -6.511 31.552 -66.374 1.00 65.95 H
ATOM 229 HB1 ALA A 68 -5.141 33.466 -67.219 1.00 70.78 H
ATOM 230 HB2 ALA A 68 -6.128 32.773 -68.497 1.00 70.78 H
ATOM 231 HB3 ALA A 68 -6.611 34.288 -67.744 1.00 70.78 H
ATOM 232 N ILE A 69 -7.807 34.219 -64.962 1.00 69.34 N
ATOM 233 CA ILE A 69 -7.883 34.889 -63.667 1.00 67.41 C
ATOM 234 C ILE A 69 -8.378 33.925 -62.598 1.00 64.05 C
ATOM 235 O ILE A 69 -7.874 33.919 -61.467 1.00 61.98 O

ATOM 236 CB ILE A 69 -8.780 36.137 -63.758 1.00 66.30 C
ATOM 237 CG1 ILE A 69 -8.198 37.145 -64.747 1.00 66.14 C
ATOM 238 CG2 ILE A 69 -8.948 36.778 -62.388 1.00 66.51 C
ATOM 239 CD1 ILE A 69 -9.021 38.408 -64.871 1.00 71.79 C
ATOM 240 H ILE A 69 -8.448 34.511 -65.688 1.00 69.34 H
ATOM 241 HA ILE A 69 -6.880 35.218 -63.388 1.00 67.41 H
ATOM 242 HB ILE A 69 -9.766 35.837 -64.120 1.00 66.30 H
ATOM 243 HG12 ILE A 69 -7.197 37.426 -64.417 1.00 66.14 H
ATOM 244 HG13 ILE A 69 -8.055 36.714 -65.734 1.00 66.14 H
ATOM 245 HG21 ILE A 69 -9.588 37.658 -62.419 1.00 66.51 H
ATOM 246 HG22 ILE A 69 -9.436 36.125 -61.665 1.00 66.51 H
ATOM 247 HG23 ILE A 69 -7.988 37.082 -61.968 1.00 66.51 H
ATOM 248 HD11 ILE A 69 -8.663 39.015 -65.697 1.00 71.79 H
ATOM 249 HD12 ILE A 69 -10.074 38.187 -65.047 1.00 71.79 H
ATOM 250 HD13 ILE A 69 -8.941 39.041 -63.988 1.00 71.79 H
ATOM 251 N THR A 70 -9.367 33.095 -62.936 1.00 59.61 N
ATOM 252 CA THR A 70 -9.900 32.141 -61.968 1.00 62.79 C
ATOM 253 C THR A 70 -8.822 31.165 -61.510 1.00 53.25 C
ATOM 254 O THR A 70 -8.626 30.958 -60.308 1.00 54.69 O
ATOM 255 CB THR A 70 -11.089 31.389 -62.570 1.00 66.88 C
ATOM 256 CG2 THR A 70 -11.650 30.389 -61.571 1.00 63.88 C
ATOM 257 OG1 THR A 70 -12.114 32.323 -62.929 1.00 74.48 O
ATOM 258 H THR A 70 -9.771 33.107 -63.862 1.00 59.61 H
ATOM 259 HA THR A 70 -10.246 32.699 -61.095 1.00 62.79 H
ATOM 260 HB THR A 70 -10.796 30.860 -63.477 1.00 66.88 H
ATOM 261 HG1 THR A 70 -11.824 32.818 -63.706 1.00 74.48 H
ATOM 262 HG21 THR A 70 -12.584 29.965 -61.940 1.00 63.88 H
ATOM 263 HG22 THR A 70 -10.979 29.547 -61.396 1.00 63.88 H
ATOM 264 HG23 THR A 70 -11.864 30.858 -60.610 1.00 63.88 H
ATOM 265 N ILE A 71 -8.106 30.562 -62.462 1.00 53.94 N
ATOM 266 CA ILE A 71 -7.037 29.630 -62.114 1.00 58.39 C
ATOM 267 C ILE A 71 -5.945 30.344 -61.329 1.00 59.04 C
ATOM 268 O ILE A 71 -5.357 29.781 -60.395 1.00 54.06 O
ATOM 269 CB ILE A 71 -6.495 28.866 -63.361 1.00 57.59 C
ATOM 270 CG1 ILE A 71 -7.612 27.940 -63.917 1.00 51.97 C
ATOM 271 CG2 ILE A 71 -5.217 28.040 -63.081 1.00 51.59 C
ATOM 272 CD1 ILE A 71 -7.316 27.296 -65.281 1.00 53.42 C
ATOM 273 H ILE A 71 -8.275 30.742 -63.444 1.00 53.94 H
ATOM 274 HA ILE A 71 -7.461 28.848 -61.482 1.00 58.39 H
ATOM 275 HB ILE A 71 -6.255 29.602 -64.131 1.00 57.59 H

ATOM 276 HG12 ILE A 71 -7.822 27.153 -63.192 1.00 51.97 H
ATOM 277 HG13 ILE A 71 -8.543 28.498 -64.015 1.00 51.97 H
ATOM 278 HG21 ILE A 71 -4.907 27.454 -63.946 1.00 51.59 H
ATOM 279 HG22 ILE A 71 -4.369 28.675 -62.827 1.00 51.59 H
ATOM 280 HG23 ILE A 71 -5.368 27.347 -62.253 1.00 51.59 H
ATOM 281 HD11 ILE A 71 -7.244 26.211 -65.200 1.00 53.42 H
ATOM 282 HD12 ILE A 71 -8.102 27.523 -66.001 1.00 53.42 H
ATOM 283 HD13 ILE A 71 -6.382 27.650 -65.706 1.00 53.42 H
ATOM 284 N MET A 72 -5.667 31.600 -61.683 1.00 52.90 N
ATOM 285 CA MET A 72 -4.676 32.377 -60.947 1.00 57.12 C
ATOM 286 C MET A 72 -5.130 32.638 -59.518 1.00 55.10 C
ATOM 287 O MET A 72 -4.369 32.426 -58.567 1.00 49.99 O
ATOM 288 CB MET A 72 -4.395 33.694 -61.669 1.00 66.43 C
ATOM 289 CG MET A 72 -3.244 33.580 -62.628 1.00 88.45 C
ATOM 290 SD MET A 72 -1.977 32.566 -61.852 1.00103.04 S
ATOM 291 CE MET A 72 -1.334 33.696 -60.618 1.00106.69 C
ATOM 292 H MET A 72 -6.135 32.046 -62.461 1.00 52.90 H
ATOM 293 HA MET A 72 -3.877 31.678 -60.743 1.00 57.12 H
ATOM 294 HB2 MET A 72 -5.281 34.057 -62.184 1.00 66.43 H
ATOM 295 HB3 MET A 72 -4.150 34.483 -60.956 1.00 66.43 H
ATOM 296 HG2 MET A 72 -3.560 33.085 -63.547 1.00 88.45 H
ATOM 297 HG3 MET A 72 -2.845 34.557 -62.901 1.00 88.45 H
ATOM 298 HE1 MET A 72 -0.467 33.265 -60.119 1.00106.69 H
ATOM 299 HE2 MET A 72 -1.030 34.634 -61.082 1.00106.69 H
ATOM 300 HE3 MET A 72 -2.087 33.911 -59.861 1.00106.69 H
ATOM 301 N ALA A 73 -6.366 33.114 -59.351 1.00 58.28 N
ATOM 302 CA ALA A 73 -6.901 33.318 -58.011 1.00 56.01 C
ATOM 303 C ALA A 73 -6.957 32.013 -57.232 1.00 53.49 C
ATOM 304 O ALA A 73 -6.779 32.013 -56.009 1.00 55.46 O
ATOM 305 CB ALA A 73 -8.288 33.956 -58.090 1.00 56.84 C
ATOM 306 H ALA A 73 -6.971 33.309 -60.141 1.00 58.28 H
ATOM 307 HA ALA A 73 -6.243 34.006 -57.476 1.00 56.01 H
ATOM 308 HB1 ALA A 73 -8.702 34.130 -57.096 1.00 56.84 H
ATOM 309 HB2 ALA A 73 -8.245 34.919 -58.600 1.00 56.84 H
ATOM 310 HB3 ALA A 73 -8.989 33.325 -58.638 1.00 56.84 H
ATOM 311 N LEU A 74 -7.191 30.893 -57.920 1.00 52.68 N
ATOM 312 CA LEU A 74 -7.207 29.593 -57.257 1.00 54.76 C
ATOM 313 C LEU A 74 -5.863 29.295 -56.604 1.00 44.00 C
ATOM 314 O LEU A 74 -5.784 29.059 -55.393 1.00 46.36 O
ATOM 315 CB LEU A 74 -7.575 28.498 -58.259 1.00 59.23 C

ATOM 316 CG LEU A 74 -8.996 27.937 -58.197 1.00 63.81 C
ATOM 317 CD1 LEU A 74 -9.197 26.878 -59.271 1.00 60.68 C
ATOM 318 CD2 LEU A 74 -9.279 27.363 -56.819 1.00 60.26 C
ATOM 319 H LEU A 74 -7.377 30.919 -58.915 1.00 52.68 H
ATOM 320 HA LEU A 74 -7.955 29.635 -56.463 1.00 54.76 H
ATOM 321 HB2 LEU A 74 -7.393 28.852 -59.267 1.00 59.23 H
ATOM 322 HB3 LEU A 74 -6.902 27.644 -58.151 1.00 59.23 H
ATOM 323 HG LEU A 74 -9.694 28.754 -58.388 1.00 63.81 H
ATOM 324 HD11 LEU A 74 -10.095 27.083 -59.855 1.00 60.68 H
ATOM 325 HD12 LEU A 74 -8.366 26.839 -59.977 1.00 60.68 H
ATOM 326 HD13 LEU A 74 -9.286 25.882 -58.853 1.00 60.68 H
ATOM 327 HD21 LEU A 74 -9.838 26.427 -56.845 1.00 60.26 H
ATOM 328 HD22 LEU A 74 -8.367 27.171 -56.251 1.00 60.26 H
ATOM 329 HD23 LEU A 74 -9.880 28.060 -56.235 1.00 60.26 H
ATOM 330 N TYR A 75 -4.789 29.304 -57.398 1.00 45.87 N
ATOM 331 CA TYR A 75 -3.469 28.984 -56.864 1.00 46.23 C
ATOM 332 C TYR A 75 -3.040 29.985 -55.799 1.00 44.98 C
ATOM 333 O TYR A 75 -2.413 29.609 -54.802 1.00 43.70 O
ATOM 334 CB TYR A 75 -2.421 28.952 -57.992 1.00 46.16 C
ATOM 335 CG TYR A 75 -2.387 27.674 -58.805 1.00 52.53 C
ATOM 336 CD1 TYR A 75 -1.999 26.473 -58.177 1.00 52.84 C
ATOM 337 CD2 TYR A 75 -2.704 27.674 -60.179 1.00 61.70 C
ATOM 338 CE1 TYR A 75 -1.925 25.277 -58.913 1.00 59.53 C
ATOM 339 CE2 TYR A 75 -2.637 26.475 -60.915 1.00 61.15 C
ATOM 340 CZ TYR A 75 -2.249 25.277 -60.280 1.00 56.42 C
ATOM 341 OH TYR A 75 -2.188 24.109 -60.972 1.00 47.68 O
ATOM 342 H TYR A 75 -4.869 29.500 -58.388 1.00 45.87 H
ATOM 343 HA TYR A 75 -3.521 28.002 -56.389 1.00 46.23 H
ATOM 344 HB2 TYR A 75 -2.519 29.824 -58.641 1.00 46.16 H
ATOM 345 HB3 TYR A 75 -1.429 29.025 -57.542 1.00 46.16 H
ATOM 346 HD1 TYR A 75 -1.762 26.470 -57.125 1.00 52.84 H
ATOM 347 HD2 TYR A 75 -3.003 28.587 -60.675 1.00 61.70 H
ATOM 348 HE1 TYR A 75 -1.627 24.355 -58.433 1.00 59.53 H
ATOM 349 HE2 TYR A 75 -2.883 26.482 -61.967 1.00 61.15 H
ATOM 350 HH TYR A 75 -2.627 24.153 -61.842 1.00 47.68 H
ATOM 351 N SER A 76 -3.375 31.264 -55.985 1.00 41.44 N
ATOM 352 CA SER A 76 -2.945 32.282 -55.034 1.00 44.48 C
ATOM 353 C SER A 76 -3.661 32.135 -53.696 1.00 45.16 C
ATOM 354 O SER A 76 -3.026 32.203 -52.638 1.00 43.85 O
ATOM 355 CB SER A 76 -3.177 33.675 -55.618 1.00 48.53 C

ATOM 356 OG SER A 76 -2.407 33.867 -56.791 1.00 70.85 O
ATOM 357 H SER A 76 -3.896 31.560 -56.800 1.00 41.44 H
ATOM 358 HA SER A 76 -1.873 32.170 -54.854 1.00 44.48 H
ATOM 359 HB2 SER A 76 -4.230 33.828 -55.860 1.00 48.53 H
ATOM 360 HB3 SER A 76 -2.899 34.446 -54.898 1.00 48.53 H
ATOM 361 HG SER A 76 -2.811 33.362 -57.503 1.00 70.85 H
ATOM 362 N ILE A 77 -4.978 31.929 -53.723 1.00 45.99 N
ATOM 363 CA ILE A 77 -5.746 31.840 -52.484 1.00 47.16 C
ATOM 364 C ILE A 77 -5.347 30.600 -51.694 1.00 45.25 C
ATOM 365 O ILE A 77 -5.115 30.665 -50.482 1.00 44.45 O
ATOM 366 CB ILE A 77 -7.255 31.860 -52.789 1.00 58.94 C
ATOM 367 CG1 ILE A 77 -7.674 33.245 -53.290 1.00 65.91 C
ATOM 368 CG2 ILE A 77 -8.061 31.465 -51.561 1.00 57.35 C
ATOM 369 CD1 ILE A 77 -9.086 33.301 -53.825 1.00 68.01 C
ATOM 370 H ILE A 77 -5.488 31.865 -54.596 1.00 45.99 H
ATOM 371 HA ILE A 77 -5.510 32.711 -51.868 1.00 47.16 H
ATOM 372 HB ILE A 77 -7.463 31.131 -53.574 1.00 58.94 H
ATOM 373 HG12 ILE A 77 -7.580 33.962 -52.474 1.00 65.91 H
ATOM 374 HG13 ILE A 77 -6.996 33.609 -54.061 1.00 65.91 H
ATOM 375 HG21 ILE A 77 -9.130 31.585 -51.734 1.00 57.35 H
ATOM 376 HG22 ILE A 77 -7.934 30.418 -51.283 1.00 57.35 H
ATOM 377 HG23 ILE A 77 -7.805 32.080 -50.697 1.00 57.35 H
ATOM 378 HD11 ILE A 77 -9.193 34.112 -54.546 1.00 68.01 H
ATOM 379 HD12 ILE A 77 -9.363 32.376 -54.332 1.00 68.01 H
ATOM 380 HD13 ILE A 77 -9.807 33.478 -53.027 1.00 68.01 H
ATOM 381 N VAL A 78 -5.255 29.452 -52.370 1.00 37.62 N
ATOM 382 CA VAL A 78 -4.893 28.215 -51.683 1.00 39.31 C
ATOM 383 C VAL A 78 -3.495 28.323 -51.088 1.00 43.66 C
ATOM 384 O VAL A 78 -3.239 27.855 -49.971 1.00 41.53 O
ATOM 385 CB VAL A 78 -5.014 27.017 -52.644 1.00 41.14 C
ATOM 386 CG1 VAL A 78 -4.493 25.750 -51.989 1.00 35.97 C
ATOM 387 CG2 VAL A 78 -6.459 26.835 -53.074 1.00 41.50 C
ATOM 388 H VAL A 78 -5.441 29.406 -53.364 1.00 37.62 H
ATOM 389 HA VAL A 78 -5.592 28.065 -50.857 1.00 39.31 H
ATOM 390 HB VAL A 78 -4.415 27.211 -53.536 1.00 41.14 H
ATOM 391 HG11 VAL A 78 -4.647 24.906 -52.659 1.00 35.97 H
ATOM 392 HG12 VAL A 78 -3.431 25.768 -51.753 1.00 35.97 H
ATOM 393 HG13 VAL A 78 -5.041 25.520 -51.078 1.00 35.97 H
ATOM 394 HG21 VAL A 78 -6.551 26.010 -53.778 1.00 41.50 H
ATOM 395 HG22 VAL A 78 -7.096 26.602 -52.220 1.00 41.50 H

```
ATOM    396 HG23 VAL A  78      -6.877  27.718 -53.553  1.00 41.50           H
ATOM    397  N   CYS A  79      -2.572  28.954 -51.816  1.00 44.10           N
ATOM    398  CA  CYS A  79      -1.216  29.127 -51.307  1.00 42.55           C
ATOM    399  C   CYS A  79      -1.201  30.011 -50.066  1.00 47.69           C
ATOM    400  O   CYS A  79      -0.602  29.653 -49.045  1.00 45.61           O
ATOM    401  CB  CYS A  79      -0.320  29.714 -52.397  1.00 39.85           C
ATOM    402  SG  CYS A  79       1.361  30.072 -51.857  1.00 48.18           S
ATOM    403  H   CYS A  79      -2.787  29.331 -52.730  1.00 44.10           H
ATOM    404  HA  CYS A  79      -0.819  28.149 -51.037  1.00 42.55           H
ATOM    405  HB2 CYS A  79      -0.276  29.042 -53.249  1.00 39.85           H
ATOM    406  HB3 CYS A  79      -0.744  30.645 -52.774  1.00 39.85           H
ATOM    407  HG  CYS A  79       1.729  28.798 -51.686  1.00 48.18           H
ATOM    408  N   VAL A  80      -1.862  31.169 -50.135  1.00 41.50           N
ATOM    409  CA  VAL A  80      -1.849  32.110 -49.017  1.00 43.75           C
ATOM    410  C   VAL A  80      -2.537  31.506 -47.798  1.00 45.31           C
ATOM    411  O   VAL A  80      -2.028  31.587 -46.675  1.00 43.24           O
ATOM    412  CB  VAL A  80      -2.499  33.443 -49.431  1.00 42.20           C
ATOM    413  CG1 VAL A  80      -2.723  34.327 -48.215  1.00 51.14           C
ATOM    414  CG2 VAL A  80      -1.632  34.159 -50.457  1.00 49.76           C
ATOM    415  H   VAL A  80      -2.351  31.446 -50.977  1.00 41.50           H
ATOM    416  HA  VAL A  80      -0.808  32.304 -48.747  1.00 43.75           H
ATOM    417  HB  VAL A  80      -3.467  33.242 -49.894  1.00 42.20           H
ATOM    418 HG11 VAL A  80      -3.016  35.331 -48.522  1.00 51.14           H
ATOM    419 HG12 VAL A  80      -3.526  33.967 -47.571  1.00 51.14           H
ATOM    420 HG13 VAL A  80      -1.818  34.426 -47.614  1.00 51.14           H
ATOM    421 HG21 VAL A  80      -2.137  35.043 -50.846  1.00 49.76           H
ATOM    422 HG22 VAL A  80      -0.692  34.488 -50.013  1.00 49.76           H
ATOM    423 HG23 VAL A  80      -1.376  33.531 -51.308  1.00 49.76           H
ATOM    424  N   VAL A  81      -3.707  30.895 -48.000  1.00 43.35           N
ATOM    425  CA  VAL A  81      -4.433  30.293 -46.885  1.00 46.33           C
ATOM    426  C   VAL A  81      -3.641  29.131 -46.298  1.00 48.79           C
ATOM    427  O   VAL A  81      -3.562  28.969 -45.075  1.00 46.75           O
ATOM    428  CB  VAL A  81      -5.839  29.855 -47.338  1.00 47.71           C
ATOM    429  CG1 VAL A  81      -6.517  29.026 -46.256  1.00 50.80           C
ATOM    430  CG2 VAL A  81      -6.684  31.072 -47.684  1.00 44.41           C
ATOM    431  H   VAL A  81      -4.120  30.831 -48.923  1.00 43.35           H
ATOM    432  HA  VAL A  81      -4.545  31.045 -46.101  1.00 46.33           H
ATOM    433  HB  VAL A  81      -5.751  29.236 -48.233  1.00 47.71           H
ATOM    434 HG11 VAL A  81      -7.544  28.793 -46.539  1.00 50.80           H
ATOM    435 HG12 VAL A  81      -6.028  28.071 -46.077  1.00 50.80           H
```

ATOM 436 HG13 VAL A 81 -6.556 29.563 -45.308 1.00 50.80 H
ATOM 437 HG21 VAL A 81 -7.656 30.772 -48.077 1.00 44.41 H
ATOM 438 HG22 VAL A 81 -6.862 31.691 -46.804 1.00 44.41 H
ATOM 439 HG23 VAL A 81 -6.218 31.709 -48.434 1.00 44.41 H
ATOM 440 N GLY A 82 -3.030 28.314 -47.159 1.00 42.80 N
ATOM 441 CA GLY A 82 -2.248 27.191 -46.670 1.00 41.72 C
ATOM 442 C GLY A 82 -1.018 27.621 -45.892 1.00 45.69 C
ATOM 443 O GLY A 82 -0.716 27.061 -44.835 1.00 41.53 O
ATOM 444 H GLY A 82 -3.110 28.444 -48.160 1.00 42.80 H
ATOM 445 HA2 GLY A 82 -2.862 26.506 -46.083 1.00 41.72 H
ATOM 446 HA3 GLY A 82 -1.918 26.666 -47.552 1.00 41.72 H
ATOM 447 N LEU A 83 -0.292 28.617 -46.401 1.00 37.86 N
ATOM 448 CA LEU A 83 0.906 29.076 -45.706 1.00 41.27 C
ATOM 449 C LEU A 83 0.556 29.765 -44.393 1.00 45.51 C
ATOM 450 O LEU A 83 1.214 29.535 -43.373 1.00 45.79 O
ATOM 451 CB LEU A 83 1.716 30.008 -46.609 1.00 41.52 C
ATOM 452 CG LEU A 83 2.402 29.330 -47.800 1.00 50.52 C
ATOM 453 CD1 LEU A 83 3.148 30.345 -48.658 1.00 48.07 C
ATOM 454 CD2 LEU A 83 3.343 28.233 -47.325 1.00 50.51 C
ATOM 455 H LEU A 83 -0.533 29.057 -47.281 1.00 37.86 H
ATOM 456 HA LEU A 83 1.517 28.211 -45.444 1.00 41.27 H
ATOM 457 HB2 LEU A 83 1.066 30.807 -46.971 1.00 41.52 H
ATOM 458 HB3 LEU A 83 2.486 30.504 -46.015 1.00 41.52 H
ATOM 459 HG LEU A 83 1.651 28.856 -48.429 1.00 50.52 H
ATOM 460 HD11 LEU A 83 3.207 30.009 -49.692 1.00 48.07 H
ATOM 461 HD12 LEU A 83 2.661 31.321 -48.654 1.00 48.07 H
ATOM 462 HD13 LEU A 83 4.171 30.489 -48.309 1.00 48.07 H
ATOM 463 HD21 LEU A 83 4.112 28.012 -48.066 1.00 50.51 H
ATOM 464 HD22 LEU A 83 3.850 28.514 -46.403 1.00 50.51 H
ATOM 465 HD23 LEU A 83 2.812 27.306 -47.128 1.00 50.51 H
ATOM 466 N PHE A 84 -0.485 30.600 -44.392 1.00 46.18 N
ATOM 467 CA PHE A 84 -0.850 31.321 -43.176 1.00 51.28 C
ATOM 468 C PHE A 84 -1.341 30.369 -42.092 1.00 49.44 C
ATOM 469 O PHE A 84 -0.913 30.457 -40.936 1.00 52.38 O
ATOM 470 CB PHE A 84 -1.915 32.374 -43.481 1.00 52.21 C
ATOM 471 CG PHE A 84 -2.538 32.970 -42.251 1.00 63.43 C
ATOM 472 CD1 PHE A 84 -1.862 33.926 -41.513 1.00 71.04 C
ATOM 473 CD2 PHE A 84 -3.794 32.568 -41.827 1.00 66.16 C
ATOM 474 CE1 PHE A 84 -2.426 34.472 -40.377 1.00 73.24 C
ATOM 475 CE2 PHE A 84 -4.364 33.112 -40.692 1.00 69.35 C

ATOM	476	CZ	PHE	A	84	-3.679	34.067	-39.968	1.00	70.14	C
ATOM	477	H	PHE	A	84	-1.021	30.780	-45.232	1.00	46.18	H
ATOM	478	HA	PHE	A	84	0.033	31.837	-42.791	1.00	51.28	H
ATOM	479	HB2	PHE	A	84	-1.476	33.177	-44.075	1.00	52.21	H
ATOM	480	HB3	PHE	A	84	-2.704	31.942	-44.099	1.00	52.21	H
ATOM	481	HD1	PHE	A	84	-0.879	34.249	-41.823	1.00	71.04	H
ATOM	482	HD2	PHE	A	84	-4.338	31.818	-42.383	1.00	66.16	H
ATOM	483	HE1	PHE	A	84	-1.886	35.216	-39.809	1.00	73.24	H
ATOM	484	HE2	PHE	A	84	-5.343	32.789	-40.371	1.00	69.35	H
ATOM	485	HZ	PHE	A	84	-4.121	34.492	-39.079	1.00	70.14	H
ATOM	486	N	GLY	A	85	-2.248	29.458	-42.447	1.00	44.61	N
ATOM	487	CA	GLY	A	85	-2.815	28.569	-41.447	1.00	41.89	C
ATOM	488	C	GLY	A	85	-1.787	27.641	-40.830	1.00	45.37	C
ATOM	489	O	GLY	A	85	-1.828	27.368	-39.627	1.00	46.18	O
ATOM	490	H	GLY	A	85	-2.584	29.392	-43.400	1.00	44.61	H
ATOM	491	HA2	GLY	A	85	-3.309	29.150	-40.666	1.00	41.89	H
ATOM	492	HA3	GLY	A	85	-3.586	27.962	-41.922	1.00	41.89	H
ATOM	493	N	ASN	A	86	-0.845	27.154	-41.636	1.00	37.27	N
ATOM	494	CA	ASN	A	86	0.118	26.189	-41.118	1.00	35.97	C
ATOM	495	C	ASN	A	86	1.270	26.867	-40.387	1.00	40.23	C
ATOM	496	O	ASN	A	86	1.787	26.321	-39.407	1.00	40.16	O
ATOM	497	CB	ASN	A	86	0.632	25.305	-42.252	1.00	34.90	C
ATOM	498	CG	ASN	A	86	-0.409	24.311	-42.716	1.00	39.72	C
ATOM	499	ND2	ASN	A	86	-0.952	24.530	-43.908	1.00	36.36	N
ATOM	500	OD1	ASN	A	86	-0.732	23.361	-42.002	1.00	38.34	O
ATOM	501	H	ASN	A	86	-0.813	27.386	-42.620	1.00	37.27	H
ATOM	502	HA	ASN	A	86	-0.395	25.534	-40.408	1.00	35.97	H
ATOM	503	HB2	ASN	A	86	1.007	25.906	-43.082	1.00	34.90	H
ATOM	504	HB3	ASN	A	86	1.477	24.724	-41.890	1.00	34.90	H
ATOM	505	HD21	ASN	A	86	-0.701	25.355	-44.441	1.00	36.36	H
ATOM	506	HD22	ASN	A	86	-1.669	23.907	-44.258	1.00	36.36	H
ATOM	507	N	PHE	A	87	1.689	28.051	-40.840	1.00	39.11	N
ATOM	508	CA	PHE	A	87	2.687	28.795	-40.082	1.00	38.59	C
ATOM	509	C	PHE	A	87	2.126	29.275	-38.750	1.00	42.93	C
ATOM	510	O	PHE	A	87	2.870	29.385	-37.769	1.00	44.51	O
ATOM	511	CB	PHE	A	87	3.210	29.976	-40.899	1.00	44.99	C
ATOM	512	CG	PHE	A	87	4.423	29.648	-41.725	1.00	59.02	C
ATOM	513	CD1	PHE	A	87	4.295	29.046	-42.965	1.00	56.30	C
ATOM	514	CD2	PHE	A	87	5.694	29.940	-41.256	1.00	71.91	C
ATOM	515	CE1	PHE	A	87	5.410	28.743	-43.725	1.00	58.43	C

ATOM	516	CE2	PHE	A	87	6.813	29.640	-42.011	1.00	76.67	C
ATOM	517	CZ	PHE	A	87	6.670	29.040	-43.246	1.00	69.33	C
ATOM	518	H	PHE	A	87	1.283	28.490	-41.657	1.00	39.11	H
ATOM	519	HA	PHE	A	87	3.527	28.150	-39.834	1.00	38.59	H
ATOM	520	HB2	PHE	A	87	2.432	30.425	-41.511	1.00	44.99	H
ATOM	521	HB3	PHE	A	87	3.508	30.786	-40.230	1.00	44.99	H
ATOM	522	HD1	PHE	A	87	3.343	28.744	-43.352	1.00	56.30	H
ATOM	523	HD2	PHE	A	87	5.821	30.405	-40.289	1.00	71.91	H
ATOM	524	HE1	PHE	A	87	5.302	28.258	-44.683	1.00	58.43	H
ATOM	525	HE2	PHE	A	87	7.798	29.871	-41.633	1.00	76.67	H
ATOM	526	HZ	PHE	A	87	7.543	28.800	-43.835	1.00	69.33	H
ATOM	527	N	LEU	A	88	0.823	29.562	-38.697	1.00	38.67	N
ATOM	528	CA	LEU	A	88	0.198	29.929	-37.432	1.00	44.99	C
ATOM	529	C	LEU	A	88	0.206	28.759	-36.456	1.00	45.56	C
ATOM	530	O	LEU	A	88	0.457	28.944	-35.259	1.00	48.49	O
ATOM	531	CB	LEU	A	88	-1.229	30.415	-37.679	1.00	41.93	C
ATOM	532	CG	LEU	A	88	-2.039	30.778	-36.434	1.00	47.36	C
ATOM	533	CD1	LEU	A	88	-1.340	31.877	-35.644	1.00	47.99	C
ATOM	534	CD2	LEU	A	88	-3.447	31.199	-36.820	1.00	48.52	C
ATOM	535	H	LEU	A	88	0.233	29.506	-39.517	1.00	38.67	H
ATOM	536	HA	LEU	A	88	0.774	30.745	-36.992	1.00	44.99	H
ATOM	537	HB2	LEU	A	88	-1.195	31.281	-38.343	1.00	41.93	H
ATOM	538	HB3	LEU	A	88	-1.770	29.646	-38.231	1.00	41.93	H
ATOM	539	HG	LEU	A	88	-2.144	29.902	-35.793	1.00	47.36	H
ATOM	540	HD11	LEU	A	88	-2.045	32.570	-35.185	1.00	47.99	H
ATOM	541	HD12	LEU	A	88	-0.748	31.454	-34.833	1.00	47.99	H
ATOM	542	HD13	LEU	A	88	-0.667	32.471	-36.263	1.00	47.99	H
ATOM	543	HD21	LEU	A	88	-4.180	30.794	-36.122	1.00	48.52	H
ATOM	544	HD22	LEU	A	88	-3.570	32.283	-36.824	1.00	48.52	H
ATOM	545	HD23	LEU	A	88	-3.724	30.852	-37.816	1.00	48.52	H
ATOM	546	N	VAL	A	89	-0.065	27.548	-36.950	1.00	39.78	N
ATOM	547	CA	VAL	A	89	-0.005	26.359	-36.104	1.00	39.03	C
ATOM	548	C	VAL	A	89	1.412	26.148	-35.588	1.00	46.81	C
ATOM	549	O	VAL	A	89	1.630	25.887	-34.398	1.00	44.00	O
ATOM	550	CB	VAL	A	89	-0.509	25.125	-36.874	1.00	36.04	C
ATOM	551	CG1	VAL	A	89	-0.251	23.861	-36.072	1.00	32.94	C
ATOM	552	CG2	VAL	A	89	-1.987	25.266	-37.193	1.00	34.65	C
ATOM	553	H	VAL	A	89	-0.292	27.420	-37.927	1.00	39.78	H
ATOM	554	HA	VAL	A	89	-0.656	26.522	-35.244	1.00	39.03	H
ATOM	555	HB	VAL	A	89	0.025	25.044	-37.821	1.00	36.04	H

ATOM 556 HG11 VAL A 89 -0.762 23.012 -36.527 1.00 32.94 H
ATOM 557 HG12 VAL A 89 0.804 23.588 -36.035 1.00 32.94 H
ATOM 558 HG13 VAL A 89 -0.620 23.946 -35.049 1.00 32.94 H
ATOM 559 HG21 VAL A 89 -2.302 24.427 -37.810 1.00 34.65 H
ATOM 560 HG22 VAL A 89 -2.577 25.224 -36.277 1.00 34.65 H
ATOM 561 HG23 VAL A 89 -2.257 26.197 -37.671 1.00 34.65 H
ATOM 562 N MET A 90 2.399 26.252 -36.481 1.00 38.82 N
ATOM 563 CA MET A 90 3.782 26.052 -36.068 1.00 44.65 C
ATOM 564 C MET A 90 4.234 27.131 -35.098 1.00 43.21 C
ATOM 565 O MET A 90 5.022 26.855 -34.187 1.00 42.84 O
ATOM 566 CB MET A 90 4.696 26.012 -37.292 1.00 41.83 C
ATOM 567 CG MET A 90 4.554 24.741 -38.115 1.00 45.54 C
ATOM 568 SD MET A 90 5.740 24.659 -39.469 1.00 48.39 S
ATOM 569 CE MET A 90 4.991 25.786 -40.641 1.00 42.55 C
ATOM 570 H MET A 90 2.213 26.448 -37.456 1.00 38.82 H
ATOM 571 HA MET A 90 3.863 25.094 -35.550 1.00 44.65 H
ATOM 572 HB2 MET A 90 4.506 26.883 -37.921 1.00 41.83 H
ATOM 573 HB3 MET A 90 5.738 26.096 -36.978 1.00 41.83 H
ATOM 574 HG2 MET A 90 4.707 23.865 -37.486 1.00 45.54 H
ATOM 575 HG3 MET A 90 3.549 24.652 -38.521 1.00 45.54 H
ATOM 576 HE1 MET A 90 5.488 25.694 -41.606 1.00 42.55 H
ATOM 577 HE2 MET A 90 3.935 25.564 -40.783 1.00 42.55 H
ATOM 578 HE3 MET A 90 5.118 26.811 -40.302 1.00 42.55 H
ATOM 579 N TYR A 91 3.741 28.359 -35.266 1.00 42.99 N
ATOM 580 CA TYR A 91 4.078 29.423 -34.328 1.00 46.23 C
ATOM 581 C TYR A 91 3.500 29.138 -32.947 1.00 48.28 C
ATOM 582 O TYR A 91 4.205 29.241 -31.937 1.00 46.72 O
ATOM 583 CB TYR A 91 3.578 30.769 -34.854 1.00 47.66 C
ATOM 584 CG TYR A 91 3.588 31.869 -33.814 1.00 54.06 C
ATOM 585 CD1 TYR A 91 4.770 32.507 -33.459 1.00 60.28 C
ATOM 586 CD2 TYR A 91 2.414 32.270 -33.187 1.00 56.57 C
ATOM 587 CE1 TYR A 91 4.783 33.512 -32.506 1.00 61.39 C
ATOM 588 CE2 TYR A 91 2.418 33.272 -32.232 1.00 54.24 C
ATOM 589 CZ TYR A 91 3.604 33.889 -31.897 1.00 59.64 C
ATOM 590 OH TYR A 91 3.611 34.888 -30.949 1.00 69.59 O
ATOM 591 H TYR A 91 3.125 28.582 -36.038 1.00 42.99 H
ATOM 592 HA TYR A 91 5.165 29.481 -34.239 1.00 46.23 H
ATOM 593 HB2 TYR A 91 4.169 31.078 -35.717 1.00 47.66 H
ATOM 594 HB3 TYR A 91 2.555 30.667 -35.218 1.00 47.66 H
ATOM 595 HD1 TYR A 91 5.697 32.215 -33.930 1.00 60.28 H

```
ATOM    596  HD2 TYR A  91       1.479  31.793 -33.442  1.00 56.57           H
ATOM    597  HE1 TYR A  91       5.712  33.995 -32.242  1.00 61.39           H
ATOM    598  HE2 TYR A  91       1.493  33.564 -31.757  1.00 54.24           H
ATOM    599  HH  TYR A  91       2.735  35.106 -30.625  1.00 69.59           H
ATOM    600  N   VAL A  92       2.215  28.781 -32.884  1.00 46.23           N
ATOM    601  CA  VAL A  92       1.576  28.514 -31.597  1.00 52.27           C
ATOM    602  C   VAL A  92       2.257  27.347 -30.894  1.00 50.69           C
ATOM    603  O   VAL A  92       2.426  27.353 -29.668  1.00 50.12           O
ATOM    604  CB  VAL A  92       0.068  28.262 -31.791  1.00 52.04           C
ATOM    605  CG1 VAL A  92      -0.559  27.730 -30.510  1.00 57.30           C
ATOM    606  CG2 VAL A  92      -0.628  29.541 -32.227  1.00 52.61           C
ATOM    607  H   VAL A  92       1.647  28.706 -33.719  1.00 46.23           H
ATOM    608  HA  VAL A  92       1.709  29.405 -30.985  1.00 52.27           H
ATOM    609  HB  VAL A  92      -0.066  27.522 -32.581  1.00 52.04           H
ATOM    610 HG11 VAL A  92      -1.646  27.711 -30.597  1.00 57.30           H
ATOM    611 HG12 VAL A  92      -0.263  26.704 -30.289  1.00 57.30           H
ATOM    612 HG13 VAL A  92      -0.319  28.353 -29.648  1.00 57.30           H
ATOM    613 HG21 VAL A  92      -1.669  29.350 -32.490  1.00 52.61           H
ATOM    614 HG22 VAL A  92      -0.621  30.283 -31.428  1.00 52.61           H
ATOM    615 HG23 VAL A  92      -0.155  30.002 -33.090  1.00 52.61           H
ATOM    616  N   ILE A  93       2.673  26.335 -31.657  1.00 43.19           N
ATOM    617  CA  ILE A  93       3.352  25.188 -31.062  1.00 44.29           C
ATOM    618  C   ILE A  93       4.719  25.595 -30.524  1.00 53.04           C
ATOM    619  O   ILE A  93       5.080  25.269 -29.386  1.00 56.03           O
ATOM    620  CB  ILE A  93       3.465  24.041 -32.082  1.00 47.47           C
ATOM    621  CG1 ILE A  93       2.103  23.377 -32.283  1.00 48.83           C
ATOM    622  CG2 ILE A  93       4.500  23.025 -31.630  1.00 53.89           C
ATOM    623  CD1 ILE A  93       2.122  22.246 -33.279  1.00 58.34           C
ATOM    624  H   ILE A  93       2.517  26.329 -32.657  1.00 43.19           H
ATOM    625  HA  ILE A  93       2.694  24.926 -30.268  1.00 44.29           H
ATOM    626  HB  ILE A  93       3.792  24.448 -33.041  1.00 47.47           H
ATOM    627 HG12 ILE A  93       1.670  23.044 -31.339  1.00 48.83           H
ATOM    628 HG13 ILE A  93       1.476  24.150 -32.694  1.00 48.83           H
ATOM    629 HG21 ILE A  93       4.534  22.194 -32.326  1.00 53.89           H
ATOM    630 HG22 ILE A  93       5.521  23.406 -31.625  1.00 53.89           H
ATOM    631 HG23 ILE A  93       4.273  22.624 -30.641  1.00 53.89           H
ATOM    632 HD11 ILE A  93       1.111  22.096 -33.636  1.00 58.34           H
ATOM    633 HD12 ILE A  93       2.749  22.469 -34.142  1.00 58.34           H
ATOM    634 HD13 ILE A  93       2.450  21.310 -32.828  1.00 58.34           H
ATOM    635  N   VAL A  94       5.497  26.318 -31.330  1.00 50.31           N
```

ATOM 636 CA VAL A 94 6.842 26.705 -30.915 1.00 56.10 C
ATOM 637 C VAL A 94 6.787 27.708 -29.767 1.00 62.86 C
ATOM 638 O VAL A 94 7.618 27.668 -28.852 1.00 62.62 O
ATOM 639 CB VAL A 94 7.626 27.252 -32.122 1.00 61.86 C
ATOM 640 CG1 VAL A 94 8.905 27.944 -31.674 1.00 66.14 C
ATOM 641 CG2 VAL A 94 7.945 26.125 -33.092 1.00 55.28 C
ATOM 642 H VAL A 94 5.197 26.580 -32.261 1.00 50.31 H
ATOM 643 HA VAL A 94 7.361 25.816 -30.548 1.00 56.10 H
ATOM 644 HB VAL A 94 7.011 27.985 -32.647 1.00 61.86 H
ATOM 645 HG11 VAL A 94 9.533 28.178 -32.534 1.00 66.14 H
ATOM 646 HG12 VAL A 94 8.719 28.895 -31.174 1.00 66.14 H
ATOM 647 HG13 VAL A 94 9.496 27.313 -31.009 1.00 66.14 H
ATOM 648 HG21 VAL A 94 8.392 26.511 -34.009 1.00 55.28 H
ATOM 649 HG22 VAL A 94 8.649 25.416 -32.655 1.00 55.28 H
ATOM 650 HG23 VAL A 94 7.060 25.558 -33.375 1.00 55.28 H
ATOM 651 N ARG A 95 5.798 28.601 -29.778 1.00 62.33 N
ATOM 652 CA ARG A 95 5.728 29.674 -28.792 1.00 67.71 C
ATOM 653 C ARG A 95 5.049 29.253 -27.492 1.00 67.29 C
ATOM 654 O ARG A 95 5.502 29.641 -26.410 1.00 73.63 O
ATOM 655 CB ARG A 95 4.996 30.883 -29.386 1.00 66.52 C
ATOM 656 CG ARG A 95 4.871 32.079 -28.448 1.00 73.97 C
ATOM 657 CD ARG A 95 6.220 32.724 -28.179 1.00 73.83 C
ATOM 658 NE ARG A 95 6.097 33.925 -27.358 1.00 86.17 N
ATOM 659 CZ ARG A 95 6.225 33.943 -26.034 1.00 93.15 C
ATOM 660 NH1 ARG A 95 6.482 32.822 -25.375 1.00 92.13 N1+
ATOM 661 NH2 ARG A 95 6.097 35.084 -25.369 1.00 94.95 N1+
ATOM 662 H ARG A 95 5.129 28.624 -30.539 1.00 62.33 H
ATOM 663 HA ARG A 95 6.745 29.985 -28.552 1.00 67.71 H
ATOM 664 HB2 ARG A 95 5.500 31.195 -30.303 1.00 66.52 H
ATOM 665 HB3 ARG A 95 3.990 30.585 -29.687 1.00 66.52 H
ATOM 666 HG2 ARG A 95 4.280 32.802 -29.011 1.00 73.97 H
ATOM 667 HG3 ARG A 95 4.305 31.901 -27.534 1.00 73.97 H
ATOM 668 HD2 ARG A 95 7.019 32.050 -27.875 1.00 73.83 H
ATOM 669 HD3 ARG A 95 6.548 33.137 -29.134 1.00 73.83 H
ATOM 670 HE ARG A 95 5.852 34.777 -27.842 1.00 86.17 H
ATOM 671 HH11 ARG A 95 6.565 31.951 -25.880 1.00 92.13 H
ATOM 672 HH12 ARG A 95 6.568 32.825 -24.368 1.00 92.13 H
ATOM 673 HH21 ARG A 95 5.897 35.942 -25.863 1.00 94.95 H
ATOM 674 HH22 ARG A 95 6.195 35.108 -24.365 1.00 94.95 H
ATOM 675 N TYR A 96 3.974 28.467 -27.564 1.00 66.56 N

ATOM 676 CA TYR A 96 3.150 28.198 -26.389 1.00 65.56 C
ATOM 677 C TYR A 96 3.161 26.733 -25.971 1.00 68.07 C
ATOM 678 O TYR A 96 3.553 26.431 -24.839 1.00 70.19 O
ATOM 679 CB TYR A 96 1.714 28.678 -26.644 1.00 63.12 C
ATOM 680 CG TYR A 96 1.607 30.169 -26.878 1.00 74.01 C
ATOM 681 CD1 TYR A 96 1.701 31.066 -25.819 1.00 77.33 C
ATOM 682 CD2 TYR A 96 1.411 30.681 -28.155 1.00 76.49 C
ATOM 683 CE1 TYR A 96 1.605 32.430 -26.026 1.00 77.55 C
ATOM 684 CE2 TYR A 96 1.312 32.045 -28.373 1.00 78.90 C
ATOM 685 CZ TYR A 96 1.410 32.914 -27.304 1.00 84.01 C
ATOM 686 OH TYR A 96 1.313 34.271 -27.513 1.00 84.67 O
ATOM 687 H TYR A 96 3.628 28.135 -28.456 1.00 66.56 H
ATOM 688 HA TYR A 96 3.495 28.752 -25.514 1.00 65.56 H
ATOM 689 HB2 TYR A 96 1.267 28.149 -27.486 1.00 63.12 H
ATOM 690 HB3 TYR A 96 1.087 28.438 -25.784 1.00 63.12 H
ATOM 691 HD1 TYR A 96 1.852 30.695 -24.816 1.00 77.33 H
ATOM 692 HD2 TYR A 96 1.327 30.005 -28.987 1.00 76.49 H
ATOM 693 HE1 TYR A 96 1.681 33.111 -25.191 1.00 77.55 H
ATOM 694 HE2 TYR A 96 1.160 32.419 -29.375 1.00 78.90 H
ATOM 695 HH TYR A 96 1.102 34.500 -28.419 1.00 84.67 H
ATOM 696 N THR A 97 2.743 25.807 -26.840 1.00 67.89 N
ATOM 697 CA THR A 97 2.534 24.429 -26.399 1.00 68.61 C
ATOM 698 C THR A 97 3.847 23.682 -26.184 1.00 70.65 C
ATOM 699 O THR A 97 3.906 22.780 -25.339 1.00 65.79 O
ATOM 700 CB THR A 97 1.654 23.678 -27.400 1.00 67.58 C
ATOM 701 CG2 THR A 97 0.374 24.457 -27.669 1.00 65.85 C
ATOM 702 OG1 THR A 97 2.366 23.496 -28.629 1.00 66.39 O
ATOM 703 H THR A 97 2.460 26.048 -27.780 1.00 67.89 H
ATOM 704 HA THR A 97 2.004 24.440 -25.443 1.00 68.61 H
ATOM 705 HB THR A 97 1.390 22.721 -26.965 1.00 67.58 H
ATOM 706 HG1 THR A 97 1.768 23.103 -29.270 1.00 66.39 H
ATOM 707 HG21 THR A 97 -0.352 23.845 -28.205 1.00 65.85 H
ATOM 708 HG22 THR A 97 -0.081 24.776 -26.738 1.00 65.85 H
ATOM 709 HG23 THR A 97 0.551 25.350 -28.271 1.00 65.85 H
ATOM 710 N LYS A 98 4.889 24.019 -26.945 1.00 74.95 N
ATOM 711 CA LYS A 98 6.286 23.602 -26.778 1.00 79.85 C
ATOM 712 C LYS A 98 6.598 22.185 -27.260 1.00 76.37 C
ATOM 713 O LYS A 98 7.750 21.752 -27.106 1.00 90.01 O
ATOM 714 CB LYS A 98 6.767 23.721 -25.322 1.00 79.28 C
ATOM 715 CG LYS A 98 6.857 25.148 -24.813 1.00 85.42 C

```
ATOM    716  CD  LYS A  98       7.794  25.976 -25.676  1.00 87.92           C
ATOM    717  CE  LYS A  98       8.091  27.321 -25.035  1.00 93.16           C
ATOM    718  NZ  LYS A  98       6.847  28.080 -24.727  1.00 94.56           N1+
ATOM    719  H   LYS A  98       4.742  24.702 -27.679  1.00 74.95           H
ATOM    720  HA  LYS A  98       6.875  24.237 -27.436  1.00 79.85           H
ATOM    721  HB2 LYS A  98       6.170  23.123 -24.638  1.00 79.28           H
ATOM    722  HB3 LYS A  98       7.774  23.314 -25.220  1.00 79.28           H
ATOM    723  HG2 LYS A  98       5.865  25.588 -24.789  1.00 85.42           H
ATOM    724  HG3 LYS A  98       7.212  25.133 -23.782  1.00 85.42           H
ATOM    725  HD2 LYS A  98       8.726  25.441 -25.866  1.00 87.92           H
ATOM    726  HD3 LYS A  98       7.339  26.175 -26.645  1.00 87.92           H
ATOM    727  HE2 LYS A  98       8.644  27.175 -24.107  1.00 93.16           H
ATOM    728  HE3 LYS A  98       8.721  27.923 -25.691  1.00 93.16           H
ATOM    729  HZ1 LYS A  98       6.366  28.331 -25.583  1.00 94.56           H
ATOM    730  HZ2 LYS A  98       6.236  27.516 -24.154  1.00 94.56           H
ATOM    731  HZ3 LYS A  98       7.084  28.926 -24.231  1.00 94.56           H
ATOM    732  N   MET A  99       5.636  21.448 -27.822  1.00 48.25           N
ATOM    733  CA  MET A  99       5.898  20.138 -28.429  1.00 58.94           C
ATOM    734  C   MET A  99       6.431  19.128 -27.412  1.00 59.86           C
ATOM    735  O   MET A  99       7.364  18.373 -27.692  1.00 54.45           O
ATOM    736  CB  MET A  99       6.861  20.259 -29.614  1.00 66.88           C
ATOM    737  CG  MET A  99       6.253  19.901 -30.958  1.00 65.96           C
ATOM    738  SD  MET A  99       7.507  19.743 -32.244  1.00 71.53           S
ATOM    739  CE  MET A  99       8.279  21.358 -32.162  1.00 66.71           C
ATOM    740  H   MET A  99       4.706  21.829 -27.924  1.00 48.25           H
ATOM    741  HA  MET A  99       4.939  19.757 -28.775  1.00 58.94           H
ATOM    742  HB2 MET A  99       7.166  21.300 -29.712  1.00 66.88           H
ATOM    743  HB3 MET A  99       7.814  19.743 -29.505  1.00 66.88           H
ATOM    744  HG2 MET A  99       5.746  18.940 -30.880  1.00 65.96           H
ATOM    745  HG3 MET A  99       5.499  20.618 -31.253  1.00 65.96           H
ATOM    746  HE1 MET A  99       8.956  21.484 -33.007  1.00 66.71           H
ATOM    747  HE2 MET A  99       7.528  22.146 -32.211  1.00 66.71           H
ATOM    748  HE3 MET A  99       8.859  21.474 -31.247  1.00 66.71           H
ATOM    749  N   LYS A 100       5.831  19.106 -26.224  1.00 56.26           N
ATOM    750  CA  LYS A 100       6.210  18.133 -25.208  1.00 57.17           C
ATOM    751  C   LYS A 100       5.427  16.832 -25.310  1.00 51.33           C
ATOM    752  O   LYS A 100       5.844  15.827 -24.724  1.00 53.76           O
ATOM    753  CB  LYS A 100       6.015  18.721 -23.807  1.00 60.34           C
ATOM    754  CG  LYS A 100       6.864  19.948 -23.515  1.00 67.29           C
ATOM    755  CD  LYS A 100       6.481  20.567 -22.181  1.00 71.75           C
```

ATOM 756 CE LYS A 100 7.309 21.806 -21.884 1.00 83.75 C
ATOM 757 NZ LYS A 100 8.754 21.483 -21.722 1.00 92.27 N1+
ATOM 758 H LYS A 100 5.090 19.755 -26.002 1.00 56.26 H
ATOM 759 HA LYS A 100 7.269 17.878 -25.294 1.00 57.17 H
ATOM 760 HB2 LYS A 100 4.961 18.976 -23.677 1.00 60.34 H
ATOM 761 HB3 LYS A 100 6.236 17.965 -23.051 1.00 60.34 H
ATOM 762 HG2 LYS A 100 7.916 19.661 -23.525 1.00 67.29 H
ATOM 763 HG3 LYS A 100 6.739 20.691 -24.303 1.00 67.29 H
ATOM 764 HD2 LYS A 100 5.425 20.840 -22.192 1.00 71.75 H
ATOM 765 HD3 LYS A 100 6.601 19.837 -21.379 1.00 71.75 H
ATOM 766 HE2 LYS A 100 7.196 22.549 -22.671 1.00 83.75 H
ATOM 767 HE3 LYS A 100 6.958 22.269 -20.962 1.00 83.75 H
ATOM 768 HZ1 LYS A 100 9.108 21.081 -22.578 1.00 92.27 H
ATOM 769 HZ2 LYS A 100 9.268 22.326 -21.510 1.00 92.27 H
ATOM 770 HZ3 LYS A 100 8.870 20.822 -20.967 1.00 92.27 H
ATOM 771 N THR A 101 4.315 16.826 -26.035 1.00 44.30 N
ATOM 772 CA THR A 101 3.418 15.683 -26.097 1.00 41.13 C
ATOM 773 C THR A 101 3.440 15.060 -27.485 1.00 44.80 C
ATOM 774 O THR A 101 3.807 15.699 -28.474 1.00 38.56 O
ATOM 775 CB THR A 101 1.986 16.092 -25.739 1.00 49.95 C
ATOM 776 CG2 THR A 101 1.935 16.676 -24.334 1.00 46.85 C
ATOM 777 OG1 THR A 101 1.523 17.074 -26.675 1.00 49.70 O
ATOM 778 H THR A 101 4.018 17.655 -26.528 1.00 44.30 H
ATOM 779 HA THR A 101 3.716 14.902 -25.394 1.00 41.13 H
ATOM 780 HB THR A 101 1.321 15.228 -25.788 1.00 49.95 H
ATOM 781 HG1 THR A 101 0.676 17.410 -26.370 1.00 49.70 H
ATOM 782 HG21 THR A 101 0.907 16.896 -24.044 1.00 46.85 H
ATOM 783 HG22 THR A 101 2.335 15.974 -23.602 1.00 46.85 H
ATOM 784 HG23 THR A 101 2.500 17.605 -24.249 1.00 46.85 H
ATOM 785 N ALA A 102 3.028 13.792 -27.542 1.00 42.68 N
ATOM 786 CA ALA A 102 2.952 13.092 -28.819 1.00 41.40 C
ATOM 787 C ALA A 102 2.004 13.795 -29.782 1.00 40.52 C
ATOM 788 O ALA A 102 2.279 13.881 -30.985 1.00 42.55 O
ATOM 789 CB ALA A 102 2.513 11.644 -28.596 1.00 35.31 C
ATOM 790 H ALA A 102 2.740 13.291 -26.714 1.00 42.68 H
ATOM 791 HA ALA A 102 3.945 13.083 -29.269 1.00 41.40 H
ATOM 792 HB1 ALA A 102 2.380 11.101 -29.531 1.00 35.31 H
ATOM 793 HB2 ALA A 102 3.229 11.107 -27.988 1.00 35.31 H
ATOM 794 HB3 ALA A 102 1.558 11.600 -28.070 1.00 35.31 H
ATOM 795 N THR A 103 0.884 14.312 -29.271 1.00 37.74 N

ATOM 796 CA THR A 103 -0.106 14.943 -30.140 1.00 40.78 C
ATOM 797 C THR A 103 0.461 16.193 -30.805 1.00 36.08 C
ATOM 798 O THR A 103 0.288 16.400 -32.011 1.00 40.10 O
ATOM 799 CB THR A 103 -1.368 15.280 -29.345 1.00 50.32 C
ATOM 800 CG2 THR A 103 -2.399 15.954 -30.241 1.00 50.62 C
ATOM 801 OG1 THR A 103 -1.927 14.076 -28.806 1.00 58.98 O
ATOM 802 H THR A 103 0.661 14.227 -28.290 1.00 37.74 H
ATOM 803 HA THR A 103 -0.371 14.234 -30.928 1.00 40.78 H
ATOM 804 HB THR A 103 -1.131 15.938 -28.507 1.00 50.32 H
ATOM 805 HG1 THR A 103 -2.301 13.562 -29.526 1.00 58.98 H
ATOM 806 HG21 THR A 103 -3.360 16.015 -29.731 1.00 50.62 H
ATOM 807 HG22 THR A 103 -2.119 16.978 -30.485 1.00 50.62 H
ATOM 808 HG23 THR A 103 -2.556 15.409 -31.173 1.00 50.62 H
ATOM 809 N ASN A 104 1.151 17.036 -30.033 1.00 37.62 N
ATOM 810 CA ASN A 104 1.732 18.244 -30.610 1.00 39.54 C
ATOM 811 C ASN A 104 2.881 17.921 -31.554 1.00 40.50 C
ATOM 812 O ASN A 104 3.135 18.676 -32.500 1.00 40.82 O
ATOM 813 CB ASN A 104 2.197 19.185 -29.501 1.00 37.80 C
ATOM 814 CG ASN A 104 1.041 19.867 -28.812 1.00 46.39 C
ATOM 815 ND2 ASN A 104 1.266 20.341 -27.592 1.00 44.81 N
ATOM 816 OD1 ASN A 104 -0.049 19.965 -29.374 1.00 48.59 O
ATOM 817 H ASN A 104 1.300 16.857 -29.049 1.00 37.62 H
ATOM 818 HA ASN A 104 0.953 18.728 -31.202 1.00 39.54 H
ATOM 819 HB2 ASN A 104 2.824 18.656 -28.781 1.00 37.80 H
ATOM 820 HB3 ASN A 104 2.806 19.986 -29.925 1.00 37.80 H
ATOM 821 HD21 ASN A 104 2.170 20.237 -27.156 1.00 44.81 H
ATOM 822 HD22 ASN A 104 0.519 20.793 -27.085 1.00 44.81 H
ATOM 823 N ILE A 105 3.580 16.812 -31.315 1.00 38.70 N
ATOM 824 CA ILE A 105 4.627 16.381 -32.235 1.00 35.71 C
ATOM 825 C ILE A 105 4.026 15.987 -33.581 1.00 37.17 C
ATOM 826 O ILE A 105 4.590 16.291 -34.640 1.00 39.96 O
ATOM 827 CB ILE A 105 5.439 15.236 -31.601 1.00 35.93 C
ATOM 828 CG1 ILE A 105 6.276 15.771 -30.435 1.00 37.32 C
ATOM 829 CG2 ILE A 105 6.322 14.552 -32.634 1.00 32.69 C
ATOM 830 CD1 ILE A 105 6.971 14.699 -29.631 1.00 39.06 C
ATOM 831 H ILE A 105 3.372 16.218 -30.523 1.00 38.70 H
ATOM 832 HA ILE A 105 5.304 17.218 -32.418 1.00 35.71 H
ATOM 833 HB ILE A 105 4.755 14.487 -31.203 1.00 35.93 H
ATOM 834 HG12 ILE A 105 7.041 16.437 -30.835 1.00 37.32 H
ATOM 835 HG13 ILE A 105 5.698 16.407 -29.771 1.00 37.32 H

ATOM 836 HG21 ILE A 105 6.944 13.774 -32.202 1.00 32.69 H
ATOM 837 HG22 ILE A 105 5.736 14.023 -33.383 1.00 32.69 H
ATOM 838 HG23 ILE A 105 6.980 15.259 -33.141 1.00 32.69 H
ATOM 839 HD11 ILE A 105 7.545 15.139 -28.816 1.00 39.06 H
ATOM 840 HD12 ILE A 105 6.220 14.061 -29.172 1.00 39.06 H
ATOM 841 HD13 ILE A 105 7.612 14.060 -30.206 1.00 39.06 H
ATOM 842 N TYR A 106 2.867 15.320 -33.563 1.00 34.96 N
ATOM 843 CA TYR A 106 2.182 14.978 -34.807 1.00 34.32 C
ATOM 844 C TYR A 106 1.612 16.218 -35.486 1.00 40.41 C
ATOM 845 O TYR A 106 1.682 16.348 -36.715 1.00 33.25 O
ATOM 846 CB TYR A 106 1.065 13.968 -34.536 1.00 32.43 C
ATOM 847 CG TYR A 106 1.547 12.583 -34.155 1.00 38.02 C
ATOM 848 CD1 TYR A 106 2.618 11.994 -34.814 1.00 38.04 C
ATOM 849 CD2 TYR A 106 0.930 11.866 -33.135 1.00 32.34 C
ATOM 850 CE1 TYR A 106 3.061 10.730 -34.473 1.00 39.42 C
ATOM 851 CE2 TYR A 106 1.368 10.597 -32.781 1.00 31.03 C
ATOM 852 CZ TYR A 106 2.432 10.035 -33.456 1.00 34.94 C
ATOM 853 OH TYR A 106 2.878 8.780 -33.121 1.00 37.20 O
ATOM 854 H TYR A 106 2.427 15.054 -32.692 1.00 34.96 H
ATOM 855 HA TYR A 106 2.904 14.568 -35.513 1.00 34.32 H
ATOM 856 HB2 TYR A 106 0.383 14.346 -33.773 1.00 32.43 H
ATOM 857 HB3 TYR A 106 0.464 13.839 -35.438 1.00 32.43 H
ATOM 858 HD1 TYR A 106 3.160 12.514 -35.587 1.00 38.04 H
ATOM 859 HD2 TYR A 106 0.096 12.299 -32.602 1.00 32.34 H
ATOM 860 HE1 TYR A 106 4.022 10.487 -34.816 1.00 39.42 H
ATOM 861 HE2 TYR A 106 0.873 10.058 -31.986 1.00 31.03 H
ATOM 862 HH TYR A 106 2.377 8.378 -32.408 1.00 37.20 H
ATOM 863 N ILE A 107 1.036 17.134 -34.704 1.00 34.40 N
ATOM 864 CA ILE A 107 0.442 18.341 -35.273 1.00 40.03 C
ATOM 865 C ILE A 107 1.506 19.193 -35.953 1.00 42.24 C
ATOM 866 O ILE A 107 1.298 19.700 -37.062 1.00 37.11 O
ATOM 867 CB ILE A 107 -0.310 19.132 -34.187 1.00 41.04 C
ATOM 868 CG1 ILE A 107 -1.523 18.341 -33.690 1.00 41.09 C
ATOM 869 CG2 ILE A 107 -0.748 20.491 -34.717 1.00 38.68 C
ATOM 870 CD1 ILE A 107 -2.248 19.002 -32.521 1.00 41.14 C
ATOM 871 H ILE A 107 0.961 16.998 -33.704 1.00 34.40 H
ATOM 872 HA ILE A 107 -0.278 18.039 -36.037 1.00 40.03 H
ATOM 873 HB ILE A 107 0.367 19.299 -33.349 1.00 41.04 H
ATOM 874 HG12 ILE A 107 -2.233 18.263 -34.514 1.00 41.09 H
ATOM 875 HG13 ILE A 107 -1.301 17.307 -33.457 1.00 41.09 H

ATOM 876 HG21 ILE A 107 -1.195 21.100 -33.932 1.00 38.68 H
ATOM 877 HG22 ILE A 107 0.040 21.104 -35.142 1.00 38.68 H
ATOM 878 HG23 ILE A 107 -1.498 20.373 -35.499 1.00 38.68 H
ATOM 879 HD11 ILE A 107 -3.029 18.345 -32.138 1.00 41.14 H
ATOM 880 HD12 ILE A 107 -1.564 19.219 -31.701 1.00 41.14 H
ATOM 881 HD13 ILE A 107 -2.736 19.931 -32.806 1.00 41.14 H
ATOM 882 N PHE A 108 2.664 19.361 -35.308 1.00 35.77 N
ATOM 883 CA PHE A 108 3.728 20.150 -35.920 1.00 41.08 C
ATOM 884 C PHE A 108 4.224 19.496 -37.203 1.00 41.68 C
ATOM 885 O PHE A 108 4.457 20.180 -38.206 1.00 37.04 O
ATOM 886 CB PHE A 108 4.887 20.347 -34.942 1.00 39.30 C
ATOM 887 CG PHE A 108 5.969 21.251 -35.469 1.00 42.62 C
ATOM 888 CD1 PHE A 108 7.011 20.742 -36.229 1.00 46.55 C
ATOM 889 CD2 PHE A 108 5.933 22.615 -35.220 1.00 45.08 C
ATOM 890 CE1 PHE A 108 7.997 21.573 -36.726 1.00 43.28 C
ATOM 891 CE2 PHE A 108 6.920 23.452 -35.712 1.00 46.34 C
ATOM 892 CZ PHE A 108 7.952 22.930 -36.466 1.00 44.51 C
ATOM 893 H PHE A 108 2.830 18.956 -34.395 1.00 35.77 H
ATOM 894 HA PHE A 108 3.326 21.134 -36.171 1.00 41.08 H
ATOM 895 HB2 PHE A 108 4.513 20.759 -34.008 1.00 39.30 H
ATOM 896 HB3 PHE A 108 5.325 19.383 -34.675 1.00 39.30 H
ATOM 897 HD1 PHE A 108 7.060 19.683 -36.439 1.00 46.55 H
ATOM 898 HD2 PHE A 108 5.127 23.037 -34.639 1.00 45.08 H
ATOM 899 HE1 PHE A 108 8.800 21.163 -37.320 1.00 43.28 H
ATOM 900 HE2 PHE A 108 6.878 24.512 -35.513 1.00 46.34 H
ATOM 901 HZ PHE A 108 8.720 23.582 -36.857 1.00 44.51 H
ATOM 902 N ASN A 109 4.399 18.172 -37.187 1.00 36.64 N
ATOM 903 CA ASN A 109 4.825 17.463 -38.389 1.00 38.44 C
ATOM 904 C ASN A 109 3.816 17.627 -39.516 1.00 41.26 C
ATOM 905 O ASN A 109 4.199 17.738 -40.687 1.00 40.10 O
ATOM 906 CB ASN A 109 5.035 15.980 -38.071 1.00 39.64 C
ATOM 907 CG ASN A 109 5.900 15.279 -39.097 1.00 38.92 C
ATOM 908 ND2 ASN A 109 5.278 14.474 -39.949 1.00 39.15 N
ATOM 909 OD1 ASN A 109 7.117 15.445 -39.111 1.00 43.31 O
ATOM 910 H ASN A 109 4.216 17.626 -36.356 1.00 36.64 H
ATOM 911 HA ASN A 109 5.760 17.891 -38.760 1.00 38.44 H
ATOM 912 HB2 ASN A 109 5.571 15.896 -37.126 1.00 39.64 H
ATOM 913 HB3 ASN A 109 4.089 15.456 -37.923 1.00 39.64 H
ATOM 914 HD21 ASN A 109 4.275 14.338 -39.913 1.00 39.15 H
ATOM 915 HD22 ASN A 109 5.812 13.978 -40.646 1.00 39.15 H

ATOM 916 N LEU A 110 2.524 17.647 -39.183 1.00 40.63 N
ATOM 917 CA LEU A 110 1.499 17.839 -40.201 1.00 36.80 C
ATOM 918 C LEU A 110 1.527 19.260 -40.750 1.00 38.28 C
ATOM 919 O LEU A 110 1.437 19.466 -41.967 1.00 33.47 O
ATOM 920 CB LEU A 110 0.123 17.512 -39.620 1.00 34.33 C
ATOM 921 CG LEU A 110 -1.072 17.587 -40.575 1.00 42.10 C
ATOM 922 CD1 LEU A 110 -0.905 16.599 -41.722 1.00 37.24 C
ATOM 923 CD2 LEU A 110 -2.375 17.335 -39.831 1.00 36.08 C
ATOM 924 H LEU A 110 2.225 17.531 -38.223 1.00 40.63 H
ATOM 925 HA LEU A 110 1.704 17.153 -41.025 1.00 36.80 H
ATOM 926 HB2 LEU A 110 0.154 16.514 -39.186 1.00 34.33 H
ATOM 927 HB3 LEU A 110 -0.070 18.179 -38.779 1.00 34.33 H
ATOM 928 HG LEU A 110 -1.138 18.591 -40.993 1.00 42.10 H
ATOM 929 HD11 LEU A 110 -1.798 16.015 -41.941 1.00 37.24 H
ATOM 930 HD12 LEU A 110 -0.655 17.133 -42.639 1.00 37.24 H
ATOM 931 HD13 LEU A 110 -0.103 15.891 -41.546 1.00 37.24 H
ATOM 932 HD21 LEU A 110 -3.049 16.640 -40.332 1.00 36.08 H
ATOM 933 HD22 LEU A 110 -2.205 16.938 -38.830 1.00 36.08 H
ATOM 934 HD23 LEU A 110 -2.924 18.267 -39.703 1.00 36.08 H
ATOM 935 N ALA A 111 1.659 20.253 -39.867 1.00 37.27 N
ATOM 936 CA ALA A 111 1.675 21.642 -40.310 1.00 40.38 C
ATOM 937 C ALA A 111 2.900 21.936 -41.166 1.00 41.27 C
ATOM 938 O ALA A 111 2.807 22.661 -42.162 1.00 40.23 O
ATOM 939 CB ALA A 111 1.626 22.579 -39.103 1.00 31.94 C
ATOM 940 H ALA A 111 1.717 20.069 -38.873 1.00 37.27 H
ATOM 941 HA ALA A 111 0.784 21.805 -40.911 1.00 40.38 H
ATOM 942 HB1 ALA A 111 1.600 23.622 -39.417 1.00 31.94 H
ATOM 943 HB2 ALA A 111 0.729 22.397 -38.511 1.00 31.94 H
ATOM 944 HB3 ALA A 111 2.486 22.440 -38.447 1.00 31.94 H
ATOM 945 N LEU A 112 4.056 21.381 -40.794 1.00 36.20 N
ATOM 946 CA LEU A 112 5.262 21.585 -41.590 1.00 40.94 C
ATOM 947 C LEU A 112 5.106 20.995 -42.987 1.00 38.12 C
ATOM 948 O LEU A 112 5.461 21.634 -43.984 1.00 34.28 O
ATOM 949 CB LEU A 112 6.466 20.971 -40.877 1.00 40.03 C
ATOM 950 CG LEU A 112 7.811 21.040 -41.601 1.00 41.57 C
ATOM 951 CD1 LEU A 112 8.209 22.488 -41.865 1.00 41.38 C
ATOM 952 CD2 LEU A 112 8.885 20.323 -40.796 1.00 45.01 C
ATOM 953 H LEU A 112 4.124 20.808 -39.962 1.00 36.20 H
ATOM 954 HA LEU A 112 5.426 22.659 -41.695 1.00 40.94 H
ATOM 955 HB2 LEU A 112 6.565 21.444 -39.898 1.00 40.03 H

```
ATOM    956  HB3 LEU A 112       6.242  19.923 -40.667  1.00 40.03           H
ATOM    957  HG  LEU A 112       7.742  20.523 -42.559  1.00 41.57           H
ATOM    958 HD11 LEU A 112       9.288  22.643 -41.838  1.00 41.38           H
ATOM    959 HD12 LEU A 112       7.877  22.799 -42.856  1.00 41.38           H
ATOM    960 HD13 LEU A 112       7.767  23.175 -41.144  1.00 41.38           H
ATOM    961 HD21 LEU A 112       9.340  19.527 -41.386  1.00 45.01           H
ATOM    962 HD22 LEU A 112       9.690  20.981 -40.468  1.00 45.01           H
ATOM    963 HD23 LEU A 112       8.484  19.857 -39.895  1.00 45.01           H
ATOM    964  N   ALA A 113       4.571  19.776 -43.080  1.00 31.62           N
ATOM    965  CA  ALA A 113       4.363  19.162 -44.388  1.00 32.47           C
ATOM    966  C   ALA A 113       3.343  19.940 -45.208  1.00 36.70           C
ATOM    967  O   ALA A 113       3.522  20.131 -46.417  1.00 37.48           O
ATOM    968  CB  ALA A 113       3.924  17.710 -44.205  1.00 26.60           C
ATOM    969  H   ALA A 113       4.286  19.258 -42.260  1.00 31.62           H
ATOM    970  HA  ALA A 113       5.313  19.191 -44.904  1.00 32.47           H
ATOM    971  HB1 ALA A 113       3.835  17.203 -45.165  1.00 26.60           H
ATOM    972  HB2 ALA A 113       4.670  17.189 -43.611  1.00 26.60           H
ATOM    973  HB3 ALA A 113       2.967  17.634 -43.686  1.00 26.60           H
ATOM    974  N   ASP A 114       2.268  20.401 -44.567  1.00 35.20           N
ATOM    975  CA  ASP A 114       1.241  21.147 -45.286  1.00 38.59           C
ATOM    976  C   ASP A 114       1.748  22.506 -45.751  1.00 41.43           C
ATOM    977  O   ASP A 114       1.364  22.971 -46.830  1.00 38.49           O
ATOM    978  CB  ASP A 114      -0.036  21.263 -44.428  1.00 38.51           C
ATOM    979  CG  ASP A 114      -0.823  19.960 -44.326  1.00 51.63           C
ATOM    980  OD1 ASP A 114      -0.886  19.261 -45.359  1.00 54.84           O
ATOM    981  OD2 ASP A 114      -1.494  19.752 -43.297  1.00 56.10           O1-
ATOM    982  H   ASP A 114       2.117  20.215 -43.583  1.00 35.20           H
ATOM    983  HA  ASP A 114       0.984  20.604 -46.199  1.00 38.59           H
ATOM    984  HB2 ASP A 114       0.273  21.525 -43.417  1.00 38.51           H
ATOM    985  HB3 ASP A 114      -0.696  22.054 -44.766  1.00 38.51           H
ATOM    986  N   ALA A 115       2.608  23.156 -44.962  1.00 39.13           N
ATOM    987  CA  ALA A 115       3.194  24.418 -45.402  1.00 40.65           C
ATOM    988  C   ALA A 115       4.135  24.206 -46.583  1.00 42.56           C
ATOM    989  O   ALA A 115       4.138  25.000 -47.531  1.00 42.31           O
ATOM    990  CB  ALA A 115       3.928  25.090 -44.244  1.00 40.00           C
ATOM    991  H   ALA A 115       2.888  22.784 -44.062  1.00 39.13           H
ATOM    992  HA  ALA A 115       2.393  25.091 -45.715  1.00 40.65           H
ATOM    993  HB1 ALA A 115       4.374  26.034 -44.556  1.00 40.00           H
ATOM    994  HB2 ALA A 115       3.254  25.309 -43.418  1.00 40.00           H
ATOM    995  HB3 ALA A 115       4.727  24.458 -43.854  1.00 40.00           H
```

ATOM 996 N LEU A 116 4.943 23.144 -46.544 1.00 39.38 N
ATOM 997 CA LEU A 116 5.849 22.868 -47.655 1.00 46.50 C
ATOM 998 C LEU A 116 5.086 22.490 -48.917 1.00 41.35 C
ATOM 999 O LEU A 116 5.501 22.856 -50.024 1.00 40.37 O
ATOM 1000 CB LEU A 116 6.832 21.760 -47.271 1.00 41.31 C
ATOM 1001 CG LEU A 116 7.938 22.164 -46.294 1.00 45.04 C
ATOM 1002 CD1 LEU A 116 8.667 20.941 -45.757 1.00 40.90 C
ATOM 1003 CD2 LEU A 116 8.916 23.119 -46.966 1.00 42.26 C
ATOM 1004 H LEU A 116 4.947 22.510 -45.755 1.00 39.38 H
ATOM 1005 HA LEU A 116 6.414 23.775 -47.872 1.00 46.50 H
ATOM 1006 HB2 LEU A 116 6.256 20.947 -46.830 1.00 41.31 H
ATOM 1007 HB3 LEU A 116 7.298 21.348 -48.167 1.00 41.31 H
ATOM 1008 HG LEU A 116 7.490 22.677 -45.443 1.00 45.04 H
ATOM 1009 HD11 LEU A 116 9.586 21.213 -45.236 1.00 40.90 H
ATOM 1010 HD12 LEU A 116 8.042 20.405 -45.042 1.00 40.90 H
ATOM 1011 HD13 LEU A 116 8.930 20.246 -46.554 1.00 40.90 H
ATOM 1012 HD21 LEU A 116 9.950 22.780 -46.891 1.00 42.26 H
ATOM 1013 HD22 LEU A 116 8.711 23.255 -48.029 1.00 42.26 H
ATOM 1014 HD23 LEU A 116 8.869 24.105 -46.502 1.00 42.26 H
ATOM 1015 N ALA A 117 3.970 21.771 -48.771 1.00 37.55 N
ATOM 1016 CA ALA A 117 3.159 21.406 -49.928 1.00 38.91 C
ATOM 1017 C ALA A 117 2.637 22.644 -50.645 1.00 40.28 C
ATOM 1018 O ALA A 117 2.754 22.764 -51.871 1.00 40.11 O
ATOM 1019 CB ALA A 117 2.004 20.492 -49.485 1.00 33.82 C
ATOM 1020 H ALA A 117 3.664 21.455 -47.859 1.00 37.55 H
ATOM 1021 HA ALA A 117 3.783 20.842 -50.625 1.00 38.91 H
ATOM 1022 HB1 ALA A 117 1.396 20.186 -50.337 1.00 33.82 H
ATOM 1023 HB2 ALA A 117 2.380 19.583 -49.013 1.00 33.82 H
ATOM 1024 HB3 ALA A 117 1.342 20.984 -48.772 1.00 33.82 H
ATOM 1025 N THR A 118 2.059 23.585 -49.894 1.00 37.59 N
ATOM 1026 CA THR A 118 1.531 24.790 -50.518 1.00 39.93 C
ATOM 1027 C THR A 118 2.634 25.737 -50.971 1.00 40.13 C
ATOM 1028 O THR A 118 2.367 26.629 -51.783 1.00 44.50 O
ATOM 1029 CB THR A 118 0.514 25.447 -49.547 1.00 49.41 C
ATOM 1030 CG2 THR A 118 -0.754 24.577 -49.448 1.00 47.20 C
ATOM 1031 OG1 THR A 118 1.019 25.575 -48.226 1.00 49.57 O
ATOM 1032 H THR A 118 1.968 23.480 -48.893 1.00 37.59 H
ATOM 1033 HA THR A 118 0.958 24.508 -51.403 1.00 39.93 H
ATOM 1034 HB THR A 118 0.244 26.443 -49.902 1.00 49.41 H
ATOM 1035 HG1 THR A 118 1.031 24.712 -47.787 1.00 49.57 H

ATOM 1036 HG21 THR A 118 -1.236 24.483 -50.419 1.00 47.20 H
ATOM 1037 HG22 THR A 118 -0.531 23.568 -49.099 1.00 47.20 H
ATOM 1038 HG23 THR A 118 -1.497 24.994 -48.775 1.00 47.20 H
ATOM 1039 N SER A 119 3.866 25.553 -50.486 1.00 38.77 N
ATOM 1040 CA SER A 119 4.972 26.390 -50.935 1.00 42.05 C
ATOM 1041 C SER A 119 5.354 26.124 -52.387 1.00 38.66 C
ATOM 1042 O SER A 119 6.067 26.938 -52.981 1.00 41.99 O
ATOM 1043 CB SER A 119 6.195 26.191 -50.037 1.00 43.09 C
ATOM 1044 OG SER A 119 6.815 24.941 -50.284 1.00 42.83 O
ATOM 1045 H SER A 119 4.058 24.836 -49.800 1.00 38.77 H
ATOM 1046 HA SER A 119 4.668 27.436 -50.849 1.00 42.05 H
ATOM 1047 HB2 SER A 119 6.930 26.974 -50.226 1.00 43.09 H
ATOM 1048 HB3 SER A 119 5.928 26.267 -48.983 1.00 43.09 H
ATOM 1049 HG SER A 119 6.187 24.224 -50.122 1.00 42.83 H
ATOM 1050 N THR A 120 4.902 25.013 -52.971 1.00 41.13 N
ATOM 1051 CA THR A 120 5.166 24.750 -54.381 1.00 44.69 C
ATOM 1052 C THR A 120 4.185 25.459 -55.307 1.00 48.12 C
ATOM 1053 O THR A 120 4.455 25.553 -56.510 1.00 48.32 O
ATOM 1054 CB THR A 120 5.049 23.218 -54.635 1.00 43.40 C
ATOM 1055 CG2 THR A 120 5.950 22.379 -53.712 1.00 41.11 C
ATOM 1056 OG1 THR A 120 3.716 22.735 -54.531 1.00 41.21 O
ATOM 1057 H THR A 120 4.328 24.347 -52.471 1.00 41.13 H
ATOM 1058 HA THR A 120 6.174 25.078 -54.634 1.00 44.69 H
ATOM 1059 HB THR A 120 5.356 23.022 -55.663 1.00 43.40 H
ATOM 1060 HG1 THR A 120 3.459 22.643 -53.598 1.00 41.21 H
ATOM 1061 HG21 THR A 120 5.906 21.323 -53.972 1.00 41.11 H
ATOM 1062 HG22 THR A 120 6.992 22.689 -53.792 1.00 41.11 H
ATOM 1063 HG23 THR A 120 5.663 22.463 -52.664 1.00 41.11 H
ATOM 1064 N LEU A 121 3.068 25.957 -54.777 1.00 45.47 N
ATOM 1065 CA LEU A 121 2.035 26.541 -55.629 1.00 42.50 C
ATOM 1066 C LEU A 121 2.479 27.785 -56.391 1.00 42.33 C
ATOM 1067 O LEU A 121 2.042 27.945 -57.545 1.00 48.67 O
ATOM 1068 CB LEU A 121 0.737 26.804 -54.839 1.00 41.62 C
ATOM 1069 CG LEU A 121 0.006 25.535 -54.364 1.00 47.59 C
ATOM 1070 CD1 LEU A 121 -1.219 25.901 -53.502 1.00 46.36 C
ATOM 1071 CD2 LEU A 121 -0.366 24.613 -55.542 1.00 48.72 C
ATOM 1072 H LEU A 121 2.880 25.885 -53.786 1.00 45.47 H
ATOM 1073 HA LEU A 121 1.813 25.800 -56.394 1.00 42.50 H
ATOM 1074 HB2 LEU A 121 0.978 27.418 -53.973 1.00 41.62 H
ATOM 1075 HB3 LEU A 121 0.050 27.394 -55.450 1.00 41.62 H

ATOM 1076 HG LEU A 121 0.688 24.970 -53.728 1.00 47.59 H
ATOM 1077 HD11 LEU A 121 -2.096 25.298 -53.736 1.00 46.36 H
ATOM 1078 HD12 LEU A 121 -1.004 25.751 -52.445 1.00 46.36 H
ATOM 1079 HD13 LEU A 121 -1.513 26.943 -53.626 1.00 46.36 H
ATOM 1080 HD21 LEU A 121 0.239 23.709 -55.505 1.00 48.72 H
ATOM 1081 HD22 LEU A 121 -1.413 24.314 -55.543 1.00 48.72 H
ATOM 1082 HD23 LEU A 121 -0.182 25.057 -56.516 1.00 48.72 H
ATOM 1083 N PRO A 122 3.290 28.700 -55.839 1.00 44.44 N
ATOM 1084 CA PRO A 122 3.761 29.817 -56.676 1.00 48.60 C
ATOM 1085 C PRO A 122 4.522 29.355 -57.906 1.00 46.99 C
ATOM 1086 O PRO A 122 4.412 29.980 -58.968 1.00 46.49 O
ATOM 1087 CB PRO A 122 4.647 30.625 -55.719 1.00 44.16 C
ATOM 1088 CG PRO A 122 4.096 30.321 -54.361 1.00 41.67 C
ATOM 1089 CD PRO A 122 3.683 28.879 -54.426 1.00 40.84 C
ATOM 1090 HA PRO A 122 2.899 30.425 -56.951 1.00 48.60 H
ATOM 1091 HB2 PRO A 122 5.685 30.289 -55.767 1.00 44.16 H
ATOM 1092 HB3 PRO A 122 4.637 31.693 -55.939 1.00 44.16 H
ATOM 1093 HG2 PRO A 122 4.800 30.527 -53.554 1.00 41.67 H
ATOM 1094 HG3 PRO A 122 3.212 30.938 -54.192 1.00 41.67 H
ATOM 1095 HD2 PRO A 122 4.548 28.251 -54.258 1.00 40.84 H
ATOM 1096 HD3 PRO A 122 2.914 28.621 -53.704 1.00 40.84 H
ATOM 1097 N PHE A 123 5.288 28.267 -57.795 1.00 43.45 N
ATOM 1098 CA PHE A 123 5.943 27.704 -58.971 1.00 46.19 C
ATOM 1099 C PHE A 123 4.920 27.166 -59.963 1.00 48.77 C
ATOM 1100 O PHE A 123 5.046 27.380 -61.174 1.00 47.05 O
ATOM 1101 CB PHE A 123 6.928 26.587 -58.577 1.00 45.53 C
ATOM 1102 CG PHE A 123 8.063 27.020 -57.672 1.00 52.69 C
ATOM 1103 CD1 PHE A 123 9.258 27.525 -58.225 1.00 52.11 C
ATOM 1104 CD2 PHE A 123 7.880 27.072 -56.275 1.00 54.02 C
ATOM 1105 CE1 PHE A 123 10.260 28.001 -57.392 1.00 55.66 C
ATOM 1106 CE2 PHE A 123 8.895 27.547 -55.458 1.00 52.63 C
ATOM 1107 CZ PHE A 123 10.083 28.003 -56.014 1.00 54.62 C
ATOM 1108 H PHE A 123 5.375 27.767 -56.922 1.00 43.45 H
ATOM 1109 HA PHE A 123 6.515 28.491 -59.467 1.00 46.19 H
ATOM 1110 HB2 PHE A 123 6.404 25.757 -58.104 1.00 45.53 H
ATOM 1111 HB3 PHE A 123 7.367 26.172 -59.484 1.00 45.53 H
ATOM 1112 HD1 PHE A 123 9.404 27.542 -59.293 1.00 52.11 H
ATOM 1113 HD2 PHE A 123 6.953 26.749 -55.829 1.00 54.02 H
ATOM 1114 HE1 PHE A 123 11.173 28.386 -57.817 1.00 55.66 H
ATOM 1115 HE2 PHE A 123 8.753 27.572 -54.386 1.00 52.63 H

ATOM 1116 HZ PHE A 123 10.868 28.378 -55.373 1.00 54.62 H
ATOM 1117 N GLN A 124 3.893 26.472 -59.464 1.00 47.06 N
ATOM 1118 CA GLN A 124 2.889 25.892 -60.348 1.00 45.94 C
ATOM 1119 C GLN A 124 2.046 26.969 -61.019 1.00 47.60 C
ATOM 1120 O GLN A 124 1.646 26.818 -62.180 1.00 46.26 O
ATOM 1121 CB GLN A 124 2.013 24.889 -59.581 1.00 38.61 C
ATOM 1122 CG GLN A 124 2.816 23.649 -59.161 1.00 36.71 C
ATOM 1123 CD GLN A 124 1.951 22.616 -58.456 1.00 39.59 C
ATOM 1124 NE2 GLN A 124 2.240 22.371 -57.190 1.00 37.08 N
ATOM 1125 OE1 GLN A 124 1.053 22.024 -59.054 1.00 44.76 O
ATOM 1126 H GLN A 124 3.803 26.308 -58.470 1.00 47.06 H
ATOM 1127 HA GLN A 124 3.395 25.326 -61.132 1.00 45.94 H
ATOM 1128 HB2 GLN A 124 1.558 25.363 -58.711 1.00 38.61 H
ATOM 1129 HB3 GLN A 124 1.180 24.574 -60.215 1.00 38.61 H
ATOM 1130 HG2 GLN A 124 3.248 23.170 -60.040 1.00 36.71 H
ATOM 1131 HG3 GLN A 124 3.655 23.929 -58.522 1.00 36.71 H
ATOM 1132 HE21 GLN A 124 2.978 22.827 -56.672 1.00 37.08 H
ATOM 1133 HE22 GLN A 124 1.756 21.617 -56.689 1.00 37.08 H
ATOM 1134 N SER A 125 1.761 28.060 -60.309 1.00 44.75 N
ATOM 1135 CA SER A 125 1.011 29.156 -60.911 1.00 48.84 C
ATOM 1136 C SER A 125 1.775 29.750 -62.088 1.00 53.07 C
ATOM 1137 O SER A 125 1.260 29.815 -63.210 1.00 56.61 O
ATOM 1138 CB SER A 125 0.627 30.168 -59.808 1.00 58.34 C
ATOM 1139 OG SER A 125 1.699 30.949 -59.304 1.00 77.79 O
ATOM 1140 H SER A 125 2.051 28.147 -59.343 1.00 44.75 H
ATOM 1141 HA SER A 125 0.058 28.769 -61.279 1.00 48.84 H
ATOM 1142 HB2 SER A 125 -0.128 30.849 -60.201 1.00 58.34 H
ATOM 1143 HB3 SER A 125 0.161 29.646 -58.976 1.00 58.34 H
ATOM 1144 HG SER A 125 2.422 30.377 -59.016 1.00 77.79 H
ATOM 1145 N VAL A 126 3.020 30.176 -61.847 1.00 51.16 N
ATOM 1146 CA VAL A 126 3.857 30.732 -62.912 1.00 51.42 C
ATOM 1147 C VAL A 126 3.974 29.747 -64.066 1.00 53.81 C
ATOM 1148 O VAL A 126 3.909 30.129 -65.241 1.00 51.45 O
ATOM 1149 CB VAL A 126 5.243 31.114 -62.359 1.00 56.39 C
ATOM 1150 CG1 VAL A 126 6.167 31.547 -63.490 1.00 56.26 C
ATOM 1151 CG2 VAL A 126 5.114 32.215 -61.317 1.00 56.66 C
ATOM 1152 H VAL A 126 3.411 30.134 -60.915 1.00 51.16 H
ATOM 1153 HA VAL A 126 3.478 31.623 -63.366 1.00 51.42 H
ATOM 1154 HB VAL A 126 5.679 30.242 -61.869 1.00 56.39 H
ATOM 1155 HG11 VAL A 126 7.095 31.957 -63.092 1.00 56.26 H

ATOM 1156 HG12 VAL A 126 6.459 30.718 -64.135 1.00 56.26 H
ATOM 1157 HG13 VAL A 126 5.713 32.321 -64.111 1.00 56.26 H
ATOM 1158 HG21 VAL A 126 6.079 32.435 -60.860 1.00 56.66 H
ATOM 1159 HG22 VAL A 126 4.747 33.138 -61.768 1.00 56.66 H
ATOM 1160 HG23 VAL A 126 4.428 31.960 -60.512 1.00 56.66 H
ATOM 1161 N ASN A 127 4.147 28.462 -63.747 1.00 49.91 N
ATOM 1162 CA ASN A 127 4.192 27.440 -64.785 1.00 53.68 C
ATOM 1163 C ASN A 127 2.919 27.439 -65.622 1.00 60.12 C
ATOM 1164 O ASN A 127 2.970 27.165 -66.827 1.00 66.23 O
ATOM 1165 CB ASN A 127 4.452 26.061 -64.133 1.00 52.67 C
ATOM 1166 CG ASN A 127 4.572 24.909 -65.136 1.00 59.71 C
ATOM 1167 ND2 ASN A 127 3.560 24.053 -65.227 1.00 65.68 N
ATOM 1168 OD1 ASN A 127 5.585 24.776 -65.811 1.00 63.30 O
ATOM 1169 H ASN A 127 4.225 28.163 -62.783 1.00 49.91 H
ATOM 1170 HA ASN A 127 5.037 27.673 -65.431 1.00 53.68 H
ATOM 1171 HB2 ASN A 127 5.382 26.098 -63.566 1.00 52.67 H
ATOM 1172 HB3 ASN A 127 3.667 25.826 -63.418 1.00 52.67 H
ATOM 1173 HD21 ASN A 127 2.722 24.161 -64.676 1.00 65.68 H
ATOM 1174 HD22 ASN A 127 3.606 23.298 -65.895 1.00 65.68 H
ATOM 1175 N TYR A 128 1.775 27.763 -65.013 1.00 61.86 N
ATOM 1176 CA TYR A 128 0.530 27.833 -65.769 1.00 64.74 C
ATOM 1177 C TYR A 128 0.438 29.116 -66.589 1.00 68.82 C
ATOM 1178 O TYR A 128 0.053 29.078 -67.762 1.00 72.91 O
ATOM 1179 CB TYR A 128 -0.685 27.657 -64.812 1.00 52.59 C
ATOM 1180 CG TYR A 128 -2.024 28.093 -65.393 1.00 62.72 C
ATOM 1181 CD1 TYR A 128 -2.650 27.305 -66.380 1.00 72.45 C
ATOM 1182 CD2 TYR A 128 -2.585 29.340 -65.034 1.00 61.54 C
ATOM 1183 CE1 TYR A 128 -3.798 27.778 -67.042 1.00 75.42 C
ATOM 1184 CE2 TYR A 128 -3.734 29.814 -65.698 1.00 68.38 C
ATOM 1185 CZ TYR A 128 -4.327 29.042 -66.716 1.00 74.70 C
ATOM 1186 OH TYR A 128 -5.415 29.520 -67.383 1.00 82.90 O
ATOM 1187 H TYR A 128 1.747 28.001 -64.030 1.00 61.86 H
ATOM 1188 HA TYR A 128 0.478 26.988 -66.460 1.00 64.74 H
ATOM 1189 HB2 TYR A 128 -0.755 26.618 -64.486 1.00 52.59 H
ATOM 1190 HB3 TYR A 128 -0.534 28.230 -63.899 1.00 52.59 H
ATOM 1191 HD1 TYR A 128 -2.223 26.356 -66.668 1.00 72.45 H
ATOM 1192 HD2 TYR A 128 -2.114 29.960 -64.284 1.00 61.54 H
ATOM 1193 HE1 TYR A 128 -4.250 27.174 -67.814 1.00 75.42 H
ATOM 1194 HE2 TYR A 128 -4.147 30.777 -65.437 1.00 68.38 H
ATOM 1195 HH TYR A 128 -5.733 28.926 -68.067 1.00 82.90 H

ATOM 1196 N LEU A 129 0.760 30.262 -65.983 1.00 67.11 N
ATOM 1197 CA LEU A 129 0.747 31.545 -66.683 1.00 76.46 C
ATOM 1198 C LEU A 129 1.506 31.471 -67.997 1.00 75.70 C
ATOM 1199 O LEU A 129 0.930 31.610 -69.081 1.00 81.95 O
ATOM 1200 CB LEU A 129 1.390 32.646 -65.839 1.00 84.84 C
ATOM 1201 CG LEU A 129 0.688 33.430 -64.739 1.00 93.76 C
ATOM 1202 CD1 LEU A 129 0.942 32.770 -63.432 1.00 93.40 C
ATOM 1203 CD2 LEU A 129 1.258 34.828 -64.705 1.00104.03 C
ATOM 1204 H LEU A 129 1.053 30.262 -65.014 1.00 67.11 H
ATOM 1205 HA LEU A 129 -0.289 31.815 -66.897 1.00 76.46 H
ATOM 1206 HB2 LEU A 129 2.358 32.307 -65.467 1.00 84.84 H
ATOM 1207 HB3 LEU A 129 1.662 33.420 -66.562 1.00 84.84 H
ATOM 1208 HG LEU A 129 -0.384 33.482 -64.934 1.00 93.76 H
ATOM 1209 HD11 LEU A 129 0.525 33.407 -62.656 1.00 93.40 H
ATOM 1210 HD12 LEU A 129 0.370 31.861 -63.356 1.00 93.40 H
ATOM 1211 HD13 LEU A 129 1.966 32.731 -63.132 1.00 93.40 H
ATOM 1212 HD21 LEU A 129 0.985 35.353 -63.789 1.00104.03 H
ATOM 1213 HD22 LEU A 129 2.345 34.831 -64.763 1.00104.03 H
ATOM 1214 HD23 LEU A 129 0.876 35.425 -65.534 1.00104.03 H
ATOM 1215 N MET A 130 2.818 31.266 -67.891 1.00 69.65 N
ATOM 1216 CA MET A 130 3.706 31.271 -69.041 1.00 76.07 C
ATOM 1217 C MET A 130 3.542 30.042 -69.919 1.00 67.04 C
ATOM 1218 O MET A 130 4.119 30.006 -71.011 1.00 66.30 O
ATOM 1219 CB MET A 130 5.152 31.380 -68.559 1.00 78.91 C
ATOM 1220 CG MET A 130 5.346 32.404 -67.450 1.00 79.21 C
ATOM 1221 SD MET A 130 4.766 34.046 -67.921 1.00 91.71 S
ATOM 1222 CE MET A 130 4.922 34.917 -66.365 1.00 94.83 C
ATOM 1223 H MET A 130 3.240 31.121 -66.984 1.00 69.65 H
ATOM 1224 HA MET A 130 3.475 32.143 -69.655 1.00 76.07 H
ATOM 1225 HB2 MET A 130 5.490 30.413 -68.182 1.00 78.91 H
ATOM 1226 HB3 MET A 130 5.808 31.619 -69.397 1.00 78.91 H
ATOM 1227 HG2 MET A 130 4.850 32.104 -66.529 1.00 79.21 H
ATOM 1228 HG3 MET A 130 6.406 32.476 -67.205 1.00 79.21 H
ATOM 1229 HE1 MET A 130 4.488 35.914 -66.442 1.00 94.83 H
ATOM 1230 HE2 MET A 130 4.412 34.371 -65.573 1.00 94.83 H
ATOM 1231 HE3 MET A 130 5.973 35.014 -66.091 1.00 94.83 H
ATOM 1232 N GLY A 131 2.771 29.050 -69.478 1.00 57.77 N
ATOM 1233 CA GLY A 131 2.672 27.801 -70.212 1.00 66.69 C
ATOM 1234 C GLY A 131 4.003 27.109 -70.389 1.00 70.85 C
ATOM 1235 O GLY A 131 4.221 26.443 -71.406 1.00 68.92 O

```
ATOM   1236  H   GLY A 131       2.282  29.128 -68.597  1.00 57.77           H
ATOM   1237  HA2 GLY A 131       2.004  27.134 -69.667  1.00 66.69           H
ATOM   1238  HA3 GLY A 131       2.213  27.981 -71.186  1.00 66.69           H
ATOM   1239  N   THR A 132       4.907  27.258 -69.422  1.00 71.11           N
ATOM   1240  CA  THR A 132       6.283  26.796 -69.540  1.00 68.12           C
ATOM   1241  C   THR A 132       6.927  26.854 -68.163  1.00 65.02           C
ATOM   1242  O   THR A 132       6.625  27.755 -67.376  1.00 64.28           O
ATOM   1243  CB  THR A 132       7.073  27.661 -70.537  1.00 74.02           C
ATOM   1244  CG2 THR A 132       8.532  27.247 -70.598  1.00 65.17           C
ATOM   1245  OG1 THR A 132       6.500  27.534 -71.844  1.00 87.22           O
ATOM   1246  H   THR A 132       4.678  27.778 -68.586  1.00 71.11           H
ATOM   1247  HA  THR A 132       6.272  25.755 -69.873  1.00 68.12           H
ATOM   1248  HB  THR A 132       7.009  28.715 -70.260  1.00 74.02           H
ATOM   1249  HG1 THR A 132       5.545  27.631 -71.770  1.00 87.22           H
ATOM   1250 HG21 THR A 132       9.019  27.589 -71.512  1.00 65.17           H
ATOM   1251 HG22 THR A 132       9.107  27.635 -69.757  1.00 65.17           H
ATOM   1252 HG23 THR A 132       8.568  26.174 -70.565  1.00 65.17           H
ATOM   1253  N   TRP A 133       7.801  25.886 -67.875  1.00 62.25           N
ATOM   1254  CA  TRP A 133       8.572  25.890 -66.639  1.00 59.33           C
ATOM   1255  C   TRP A 133       9.813  26.749 -66.835  1.00 60.73           C
ATOM   1256  O   TRP A 133      10.702  26.361 -67.612  1.00 60.73           O
ATOM   1257  CB  TRP A 133       8.972  24.448 -66.283  1.00 55.70           C
ATOM   1258  CG  TRP A 133       9.801  24.342 -65.039  1.00 51.04           C
ATOM   1259  CD1 TRP A 133      11.152  24.319 -64.986  1.00 46.45           C
ATOM   1260  CD2 TRP A 133       9.346  24.347 -63.655  1.00 48.16           C
ATOM   1261  CE2 TRP A 133      10.488  24.290 -62.802  1.00 45.74           C
ATOM   1262  CE3 TRP A 133       8.076  24.385 -63.036  1.00 48.43           C
ATOM   1263  NE1 TRP A 133      11.564  24.279 -63.671  1.00 50.15           N
ATOM   1264  CZ2 TRP A 133      10.368  24.271 -61.401  1.00 43.95           C
ATOM   1265  CZ3 TRP A 133       7.944  24.363 -61.634  1.00 46.50           C
ATOM   1266  CH2 TRP A 133       9.090  24.306 -60.817  1.00 44.59           C
ATOM   1267  H   TRP A 133       7.981  25.137 -68.531  1.00 62.25           H
ATOM   1268  HA  TRP A 133       7.952  26.260 -65.822  1.00 59.33           H
ATOM   1269  HB2 TRP A 133       8.075  23.844 -66.140  1.00 55.70           H
ATOM   1270  HB3 TRP A 133       9.518  23.984 -67.103  1.00 55.70           H
ATOM   1271  HD1 TRP A 133      11.791  24.327 -65.858  1.00 46.45           H
ATOM   1272  HE1 TRP A 133      12.553  24.186 -63.422  1.00 50.15           H
ATOM   1273  HE3 TRP A 133       7.191  24.415 -63.655  1.00 48.43           H
ATOM   1274  HZ2 TRP A 133      11.241  24.239 -60.771  1.00 43.95           H
ATOM   1275  HZ3 TRP A 133       6.960  24.381 -61.188  1.00 46.50           H
```

ATOM 1276 HH2 TRP A 133 8.990 24.286 -59.743 1.00 44.59 H
ATOM 1277 N PRO A 134 9.931 27.900 -66.175 1.00 59.31 N
ATOM 1278 CA PRO A 134 11.081 28.789 -66.385 1.00 61.82 C
ATOM 1279 C PRO A 134 12.171 28.717 -65.321 1.00 52.54 C
ATOM 1280 O PRO A 134 13.133 29.486 -65.414 1.00 59.88 O
ATOM 1281 CB PRO A 134 10.413 30.166 -66.351 1.00 65.76 C
ATOM 1282 CG PRO A 134 9.362 29.996 -65.274 1.00 60.39 C
ATOM 1283 CD PRO A 134 8.895 28.548 -65.348 1.00 59.94 C
ATOM 1284 HA PRO A 134 11.556 28.658 -67.359 1.00 61.82 H
ATOM 1285 HB2 PRO A 134 11.088 31.000 -66.153 1.00 65.76 H
ATOM 1286 HB3 PRO A 134 9.931 30.358 -67.311 1.00 65.76 H
ATOM 1287 HG2 PRO A 134 9.816 30.171 -64.297 1.00 60.39 H
ATOM 1288 HG3 PRO A 134 8.545 30.711 -65.376 1.00 60.39 H
ATOM 1289 HD2 PRO A 134 8.819 28.075 -64.377 1.00 59.94 H
ATOM 1290 HD3 PRO A 134 7.906 28.562 -65.758 1.00 59.94 H
ATOM 1291 N PHE A 135 12.049 27.839 -64.329 1.00 55.12 N
ATOM 1292 CA PHE A 135 12.913 27.877 -63.157 1.00 58.88 C
ATOM 1293 C PHE A 135 14.155 27.002 -63.283 1.00 60.51 C
ATOM 1294 O PHE A 135 14.977 26.984 -62.359 1.00 60.28 O
ATOM 1295 CB PHE A 135 12.116 27.471 -61.912 1.00 55.16 C
ATOM 1296 CG PHE A 135 10.916 28.339 -61.654 1.00 54.43 C
ATOM 1297 CD1 PHE A 135 11.062 29.603 -61.103 1.00 57.42 C
ATOM 1298 CD2 PHE A 135 9.641 27.892 -61.964 1.00 48.71 C
ATOM 1299 CE1 PHE A 135 9.960 30.405 -60.868 1.00 56.04 C
ATOM 1300 CE2 PHE A 135 8.535 28.687 -61.729 1.00 53.70 C
ATOM 1301 CZ PHE A 135 8.695 29.946 -61.181 1.00 61.05 C
ATOM 1302 H PHE A 135 11.259 27.210 -64.295 1.00 55.12 H
ATOM 1303 HA PHE A 135 13.268 28.896 -62.987 1.00 58.88 H
ATOM 1304 HB2 PHE A 135 11.796 26.438 -61.987 1.00 55.16 H
ATOM 1305 HB3 PHE A 135 12.750 27.503 -61.041 1.00 55.16 H
ATOM 1306 HD1 PHE A 135 12.047 29.970 -60.854 1.00 57.42 H
ATOM 1307 HD2 PHE A 135 9.500 26.914 -62.395 1.00 48.71 H
ATOM 1308 HE1 PHE A 135 10.088 31.387 -60.438 1.00 56.04 H
ATOM 1309 HE2 PHE A 135 7.547 28.325 -61.975 1.00 53.70 H
ATOM 1310 HZ PHE A 135 7.835 30.569 -60.990 1.00 61.05 H
ATOM 1311 N GLY A 136 14.316 26.279 -64.386 1.00 60.91 N
ATOM 1312 CA GLY A 136 15.516 25.497 -64.601 1.00 60.06 C
ATOM 1313 C GLY A 136 15.411 24.074 -64.089 1.00 59.72 C
ATOM 1314 O GLY A 136 14.414 23.642 -63.503 1.00 56.41 O
ATOM 1315 H GLY A 136 13.624 26.309 -65.121 1.00 60.91 H

```
ATOM   1316  HA2 GLY A 136      15.683  25.455 -65.678  1.00 60.06           H
ATOM   1317  HA3 GLY A 136      16.401  25.979 -64.180  1.00 60.06           H
ATOM   1318  N   THR A 137      16.500  23.334 -64.312  1.00 60.77           N
ATOM   1319  CA  THR A 137      16.530  21.908 -64.004  1.00 58.22           C
ATOM   1320  C   THR A 137      16.656  21.649 -62.506  1.00 56.97           C
ATOM   1321  O   THR A 137      16.021  20.727 -61.979  1.00 56.23           O
ATOM   1322  CB  THR A 137      17.692  21.238 -64.760  1.00  0.00           C
ATOM   1323  CG2 THR A 137      17.316  20.782 -66.175  1.00  0.00           C
ATOM   1324  OG1 THR A 137      18.785  22.159 -64.847  1.00  0.00           O
ATOM   1325  H   THR A 137      17.314  23.709 -64.778  1.00 60.77           H
ATOM   1326  HA  THR A 137      15.598  21.451 -64.335  1.00 58.22           H
ATOM   1327  HB  THR A 137      18.043  20.360 -64.213  1.00  0.00           H
ATOM   1328  HG1 THR A 137      19.460  21.780 -65.416  1.00  0.00           H
ATOM   1329 HG21 THR A 137      18.130  20.184 -66.586  1.00  0.00           H
ATOM   1330 HG22 THR A 137      16.477  20.094 -66.100  1.00  0.00           H
ATOM   1331 HG23 THR A 137      17.288  21.671 -66.802  1.00  0.00           H
ATOM   1332  N   ILE A 138      17.472  22.442 -61.808  1.00 55.50           N
ATOM   1333  CA  ILE A 138      17.691  22.214 -60.382  1.00 54.19           C
ATOM   1334  C   ILE A 138      16.412  22.471 -59.595  1.00 53.99           C
ATOM   1335  O   ILE A 138      16.027  21.676 -58.729  1.00 50.32           O
ATOM   1336  CB  ILE A 138      18.854  23.085 -59.872  1.00 62.95           C
ATOM   1337  CG1 ILE A 138      20.164  22.678 -60.550  1.00 66.39           C
ATOM   1338  CG2 ILE A 138      18.974  22.983 -58.360  1.00 64.64           C
ATOM   1339  CD1 ILE A 138      21.350  23.522 -60.139  1.00 72.99           C
ATOM   1340  H   ILE A 138      17.991  23.183 -62.257  1.00 55.50           H
ATOM   1341  HA  ILE A 138      17.962  21.165 -60.238  1.00 54.19           H
ATOM   1342  HB  ILE A 138      18.653  24.127 -60.126  1.00 62.95           H
ATOM   1343 HG12 ILE A 138      20.381  21.633 -60.325  1.00 66.39           H
ATOM   1344 HG13 ILE A 138      20.068  22.734 -61.635  1.00 66.39           H
ATOM   1345 HG21 ILE A 138      19.842  23.522 -57.982  1.00 64.64           H
ATOM   1346 HG22 ILE A 138      18.124  23.425 -57.840  1.00 64.64           H
ATOM   1347 HG23 ILE A 138      19.069  21.946 -58.034  1.00 64.64           H
ATOM   1348 HD11 ILE A 138      22.101  23.543 -60.930  1.00 72.99           H
ATOM   1349 HD12 ILE A 138      21.064  24.554 -59.935  1.00 72.99           H
ATOM   1350 HD13 ILE A 138      21.831  23.122 -59.246  1.00 72.99           H
ATOM   1351  N   LEU A 139      15.729  23.581 -59.889  1.00 52.91           N
ATOM   1352  CA  LEU A 139      14.492  23.892 -59.181  1.00 50.40           C
ATOM   1353  C   LEU A 139      13.379  22.920 -59.543  1.00 50.50           C
ATOM   1354  O   LEU A 139      12.495  22.660 -58.719  1.00 48.57           O
ATOM   1355  CB  LEU A 139      14.062  25.329 -59.473  1.00 52.50           C
```

ATOM 1356 CG LEU A 139 14.508 26.380 -58.452 1.00 63.26 C
ATOM 1357 CD1 LEU A 139 16.005 26.301 -58.194 1.00 76.19 C
ATOM 1358 CD2 LEU A 139 14.122 27.775 -58.917 1.00 61.59 C
ATOM 1359 H LEU A 139 16.046 24.226 -60.598 1.00 52.91 H
ATOM 1360 HA LEU A 139 14.660 23.796 -58.107 1.00 50.40 H
ATOM 1361 HB2 LEU A 139 14.398 25.597 -60.469 1.00 52.50 H
ATOM 1362 HB3 LEU A 139 12.973 25.395 -59.521 1.00 52.50 H
ATOM 1363 HG LEU A 139 13.994 26.188 -57.509 1.00 63.26 H
ATOM 1364 HD11 LEU A 139 16.420 27.269 -57.913 1.00 76.19 H
ATOM 1365 HD12 LEU A 139 16.226 25.613 -57.378 1.00 76.19 H
ATOM 1366 HD13 LEU A 139 16.560 25.970 -59.072 1.00 76.19 H
ATOM 1367 HD21 LEU A 139 14.475 28.529 -58.213 1.00 61.59 H
ATOM 1368 HD22 LEU A 139 14.558 28.018 -59.886 1.00 61.59 H
ATOM 1369 HD23 LEU A 139 13.041 27.888 -58.963 1.00 61.59 H
ATOM 1370 N CYS A 140 13.400 22.377 -60.762 1.00 47.94 N
ATOM 1371 CA CYS A 140 12.425 21.356 -61.128 1.00 45.04 C
ATOM 1372 C CYS A 140 12.571 20.123 -60.244 1.00 43.39 C
ATOM 1373 O CYS A 140 11.575 19.587 -59.746 1.00 43.25 O
ATOM 1374 CB CYS A 140 12.588 20.996 -62.623 1.00 51.30 C
ATOM 1375 SG CYS A 140 11.594 19.614 -63.244 1.00 59.60 S
ATOM 1376 H CYS A 140 14.120 22.610 -61.432 1.00 47.94 H
ATOM 1377 HA CYS A 140 11.421 21.755 -60.979 1.00 45.04 H
ATOM 1378 HB2 CYS A 140 12.357 21.858 -63.242 1.00 51.30 H
ATOM 1379 HB3 CYS A 140 13.625 20.742 -62.834 1.00 51.30 H
ATOM 1380 N LYS A 141 13.808 19.673 -60.022 1.00 46.82 N
ATOM 1381 CA LYS A 141 14.033 18.534 -59.136 1.00 48.70 C
ATOM 1382 C LYS A 141 13.633 18.859 -57.701 1.00 47.55 C
ATOM 1383 O LYS A 141 13.055 18.016 -57.006 1.00 41.87 O
ATOM 1384 CB LYS A 141 15.500 18.103 -59.194 1.00 46.54 C
ATOM 1385 CG LYS A 141 15.930 17.512 -60.527 1.00 50.52 C
ATOM 1386 CD LYS A 141 17.414 17.184 -60.528 1.00 56.71 C
ATOM 1387 CE LYS A 141 17.794 16.337 -61.731 1.00 57.81 C
ATOM 1388 NZ LYS A 141 17.124 15.004 -61.692 1.00 55.81 N1+
ATOM 1389 H LYS A 141 14.615 20.119 -60.437 1.00 46.82 H
ATOM 1390 HA LYS A 141 13.411 17.704 -59.469 1.00 48.70 H
ATOM 1391 HB2 LYS A 141 16.142 18.950 -58.945 1.00 46.54 H
ATOM 1392 HB3 LYS A 141 15.681 17.352 -58.423 1.00 46.54 H
ATOM 1393 HG2 LYS A 141 15.337 16.614 -60.681 1.00 50.52 H
ATOM 1394 HG3 LYS A 141 15.694 18.184 -61.352 1.00 50.52 H
ATOM 1395 HD2 LYS A 141 17.988 18.111 -60.545 1.00 56.71 H

ATOM 1396 HD3 LYS A 141 17.694 16.669 -59.608 1.00 56.71 H
ATOM 1397 HE2 LYS A 141 17.552 16.842 -62.667 1.00 57.81 H
ATOM 1398 HE3 LYS A 141 18.872 16.173 -61.724 1.00 57.81 H
ATOM 1399 HZ1 LYS A 141 16.143 15.131 -61.921 1.00 55.81 H
ATOM 1400 HZ2 LYS A 141 17.532 14.383 -62.376 1.00 55.81 H
ATOM 1401 HZ3 LYS A 141 17.200 14.600 -60.767 1.00 55.81 H
ATOM 1402 N ILE A 142 13.929 20.076 -57.244 1.00 45.82 N
ATOM 1403 CA ILE A 142 13.671 20.433 -55.852 1.00 44.45 C
ATOM 1404 C ILE A 142 12.173 20.535 -55.592 1.00 42.99 C
ATOM 1405 O ILE A 142 11.661 19.997 -54.603 1.00 42.95 O
ATOM 1406 CB ILE A 142 14.397 21.742 -55.495 1.00 47.55 C
ATOM 1407 CG1 ILE A 142 15.910 21.517 -55.468 1.00 49.83 C
ATOM 1408 CG2 ILE A 142 13.906 22.285 -54.160 1.00 45.00 C
ATOM 1409 CD1 ILE A 142 16.711 22.768 -55.178 1.00 49.99 C
ATOM 1410 H ILE A 142 14.410 20.751 -57.824 1.00 45.82 H
ATOM 1411 HA ILE A 142 14.062 19.641 -55.209 1.00 44.45 H
ATOM 1412 HB ILE A 142 14.178 22.487 -56.261 1.00 47.55 H
ATOM 1413 HG12 ILE A 142 16.139 20.784 -54.693 1.00 49.83 H
ATOM 1414 HG13 ILE A 142 16.268 21.063 -56.388 1.00 49.83 H
ATOM 1415 HG21 ILE A 142 14.469 23.164 -53.849 1.00 45.00 H
ATOM 1416 HG22 ILE A 142 12.869 22.621 -54.188 1.00 45.00 H
ATOM 1417 HG23 ILE A 142 14.003 21.542 -53.367 1.00 45.00 H
ATOM 1418 HD11 ILE A 142 17.731 22.664 -55.549 1.00 49.99 H
ATOM 1419 HD12 ILE A 142 16.276 23.645 -55.657 1.00 49.99 H
ATOM 1420 HD13 ILE A 142 16.777 22.962 -54.107 1.00 49.99 H
ATOM 1421 N VAL A 143 11.448 21.224 -56.475 1.00 40.26 N
ATOM 1422 CA VAL A 143 10.024 21.445 -56.245 1.00 39.82 C
ATOM 1423 C VAL A 143 9.245 20.141 -56.377 1.00 39.12 C
ATOM 1424 O VAL A 143 8.340 19.863 -55.581 1.00 40.68 O
ATOM 1425 CB VAL A 143 9.473 22.537 -57.212 1.00 44.86 C
ATOM 1426 CG1 VAL A 143 7.937 22.672 -57.249 1.00 44.25 C
ATOM 1427 CG2 VAL A 143 10.085 23.912 -56.870 1.00 42.80 C
ATOM 1428 H VAL A 143 11.871 21.659 -57.287 1.00 40.26 H
ATOM 1429 HA VAL A 143 9.889 21.804 -55.222 1.00 39.82 H
ATOM 1430 HB VAL A 143 9.792 22.281 -58.224 1.00 44.86 H
ATOM 1431 HG11 VAL A 143 7.626 23.515 -57.865 1.00 44.25 H
ATOM 1432 HG12 VAL A 143 7.452 21.786 -57.660 1.00 44.25 H
ATOM 1433 HG13 VAL A 143 7.547 22.834 -56.246 1.00 44.25 H
ATOM 1434 HG21 VAL A 143 9.789 24.672 -57.592 1.00 42.80 H
ATOM 1435 HG22 VAL A 143 9.765 24.255 -55.886 1.00 42.80 H

ATOM 1436 HG23 VAL A 143 11.174 23.889 -56.865 1.00 42.80 H
ATOM 1437 N ILE A 144 9.581 19.318 -57.374 1.00 38.20 N
ATOM 1438 CA ILE A 144 8.886 18.043 -57.536 1.00 37.51 C
ATOM 1439 C ILE A 144 9.134 17.142 -56.333 1.00 36.92 C
ATOM 1440 O ILE A 144 8.205 16.517 -55.806 1.00 38.64 O
ATOM 1441 CB ILE A 144 9.247 17.300 -58.872 1.00 45.46 C
ATOM 1442 CG1 ILE A 144 8.516 17.990 -60.054 1.00 55.47 C
ATOM 1443 CG2 ILE A 144 8.946 15.779 -58.883 1.00 41.03 C
ATOM 1444 CD1 ILE A 144 8.685 17.323 -61.432 1.00 71.81 C
ATOM 1445 H ILE A 144 10.318 19.545 -58.029 1.00 38.20 H
ATOM 1446 HA ILE A 144 7.812 18.239 -57.567 1.00 37.51 H
ATOM 1447 HB ILE A 144 10.320 17.405 -59.038 1.00 45.46 H
ATOM 1448 HG12 ILE A 144 7.447 18.033 -59.841 1.00 55.47 H
ATOM 1449 HG13 ILE A 144 8.842 19.029 -60.125 1.00 55.47 H
ATOM 1450 HG21 ILE A 144 9.248 15.313 -59.817 1.00 41.03 H
ATOM 1451 HG22 ILE A 144 9.488 15.235 -58.111 1.00 41.03 H
ATOM 1452 HG23 ILE A 144 7.881 15.594 -58.752 1.00 41.03 H
ATOM 1453 HD11 ILE A 144 8.231 17.934 -62.211 1.00 71.81 H
ATOM 1454 HD12 ILE A 144 9.735 17.191 -61.691 1.00 71.81 H
ATOM 1455 HD13 ILE A 144 8.188 16.355 -61.487 1.00 71.81 H
ATOM 1456 N SER A 145 10.384 17.070 -55.869 1.00 41.49 N
ATOM 1457 CA SER A 145 10.693 16.222 -54.723 1.00 41.47 C
ATOM 1458 C SER A 145 9.984 16.714 -53.467 1.00 37.02 C
ATOM 1459 O SER A 145 9.398 15.918 -52.724 1.00 40.57 O
ATOM 1460 CB SER A 145 12.205 16.162 -54.504 1.00 47.02 C
ATOM 1461 OG SER A 145 12.531 15.246 -53.471 1.00 52.17 O
ATOM 1462 H SER A 145 11.142 17.584 -56.299 1.00 41.49 H
ATOM 1463 HA SER A 145 10.333 15.214 -54.929 1.00 41.47 H
ATOM 1464 HB2 SER A 145 12.683 15.819 -55.421 1.00 47.02 H
ATOM 1465 HB3 SER A 145 12.610 17.146 -54.263 1.00 47.02 H
ATOM 1466 HG SER A 145 12.391 14.347 -53.788 1.00 52.17 H
ATOM 1467 N ILE A 146 10.026 18.025 -53.215 1.00 39.50 N
ATOM 1468 CA ILE A 146 9.304 18.590 -52.077 1.00 38.64 C
ATOM 1469 C ILE A 146 7.816 18.292 -52.195 1.00 38.73 C
ATOM 1470 O ILE A 146 7.149 17.966 -51.203 1.00 33.92 O
ATOM 1471 CB ILE A 146 9.530 20.137 -51.975 1.00 42.12 C
ATOM 1472 CG1 ILE A 146 10.956 20.426 -51.454 1.00 41.64 C
ATOM 1473 CG2 ILE A 146 8.500 20.947 -51.144 1.00 40.43 C
ATOM 1474 CD1 ILE A 146 11.388 21.892 -51.599 1.00 50.00 C
ATOM 1475 H ILE A 146 10.525 18.665 -53.821 1.00 39.50 H

ATOM 1476 HA ILE A 146 9.666 18.106 -51.168 1.00 38.64 H
ATOM 1477 HB ILE A 146 9.468 20.529 -52.992 1.00 42.12 H
ATOM 1478 HG12 ILE A 146 11.024 20.137 -50.404 1.00 41.64 H
ATOM 1479 HG13 ILE A 146 11.683 19.800 -51.974 1.00 41.64 H
ATOM 1480 HG21 ILE A 146 8.707 22.016 -51.189 1.00 40.43 H
ATOM 1481 HG22 ILE A 146 7.477 20.839 -51.503 1.00 40.43 H
ATOM 1482 HG23 ILE A 146 8.525 20.663 -50.095 1.00 40.43 H
ATOM 1483 HD11 ILE A 146 12.473 21.982 -51.555 1.00 50.00 H
ATOM 1484 HD12 ILE A 146 11.059 22.314 -52.549 1.00 50.00 H
ATOM 1485 HD13 ILE A 146 10.981 22.508 -50.797 1.00 50.00 H
ATOM 1486 N ASP A 147 7.277 18.373 -53.413 1.00 37.12 N
ATOM 1487 CA ASP A 147 5.846 18.169 -53.603 1.00 37.29 C
ATOM 1488 C ASP A 147 5.441 16.730 -53.303 1.00 38.62 C
ATOM 1489 O ASP A 147 4.434 16.494 -52.624 1.00 36.94 O
ATOM 1490 CB ASP A 147 5.428 18.566 -55.037 1.00 36.68 C
ATOM 1491 CG ASP A 147 3.924 18.697 -55.247 1.00 40.30 C
ATOM 1492 OD1 ASP A 147 3.193 18.918 -54.258 1.00 38.20 O
ATOM 1493 OD2 ASP A 147 3.521 18.659 -56.426 1.00 40.06 O1-
ATOM 1494 H ASP A 147 7.826 18.649 -54.218 1.00 37.12 H
ATOM 1495 HA ASP A 147 5.330 18.821 -52.893 1.00 37.29 H
ATOM 1496 HB2 ASP A 147 5.867 19.521 -55.314 1.00 36.68 H
ATOM 1497 HB3 ASP A 147 5.803 17.822 -55.741 1.00 36.68 H
ATOM 1498 N TYR A 148 6.206 15.751 -53.799 1.00 33.20 N
ATOM 1499 CA TYR A 148 5.888 14.360 -53.486 1.00 31.34 C
ATOM 1500 C TYR A 148 6.174 14.041 -52.022 1.00 37.64 C
ATOM 1501 O TYR A 148 5.376 13.359 -51.368 1.00 38.27 O
ATOM 1502 CB TYR A 148 6.674 13.396 -54.399 1.00 32.20 C
ATOM 1503 CG TYR A 148 6.013 13.097 -55.732 1.00 31.77 C
ATOM 1504 CD1 TYR A 148 6.550 13.606 -56.932 1.00 32.62 C
ATOM 1505 CD2 TYR A 148 4.861 12.283 -55.775 1.00 34.94 C
ATOM 1506 CE1 TYR A 148 5.954 13.287 -58.168 1.00 40.74 C
ATOM 1507 CE2 TYR A 148 4.259 11.973 -57.009 1.00 43.26 C
ATOM 1508 CZ TYR A 148 4.808 12.467 -58.206 1.00 41.39 C
ATOM 1509 OH TYR A 148 4.215 12.160 -59.394 1.00 39.52 O
ATOM 1510 H TYR A 148 7.017 15.948 -54.372 1.00 33.20 H
ATOM 1511 HA TYR A 148 4.822 14.191 -53.659 1.00 31.34 H
ATOM 1512 HB2 TYR A 148 7.698 13.745 -54.537 1.00 32.20 H
ATOM 1513 HB3 TYR A 148 6.767 12.425 -53.909 1.00 32.20 H
ATOM 1514 HD1 TYR A 148 7.426 14.237 -56.908 1.00 32.62 H
ATOM 1515 HD2 TYR A 148 4.425 11.899 -54.864 1.00 34.94 H

```
ATOM   1516  HE1 TYR A 148       6.380  13.677 -59.081  1.00 40.74           H
ATOM   1517  HE2 TYR A 148       3.367  11.366 -57.034  1.00 43.26           H
ATOM   1518  HH  TYR A 148       4.603  12.616 -60.146  1.00 39.52           H
ATOM   1519  N   TYR A 149       7.301  14.526 -51.491  1.00 35.85           N
ATOM   1520  CA  TYR A 149       7.659  14.238 -50.103  1.00 37.55           C
ATOM   1521  C   TYR A 149       6.558  14.668 -49.145  1.00 38.10           C
ATOM   1522  O   TYR A 149       6.199  13.931 -48.221  1.00 36.32           O
ATOM   1523  CB  TYR A 149       8.976  14.933 -49.677  1.00 33.32           C
ATOM   1524  CG  TYR A 149      10.167  14.013 -49.511  1.00 43.06           C
ATOM   1525  CD1 TYR A 149      10.165  13.057 -48.472  1.00 40.48           C
ATOM   1526  CD2 TYR A 149      11.301  14.148 -50.336  1.00 41.57           C
ATOM   1527  CE1 TYR A 149      11.296  12.255 -48.250  1.00 39.96           C
ATOM   1528  CE2 TYR A 149      12.435  13.346 -50.111  1.00 42.24           C
ATOM   1529  CZ  TYR A 149      12.437  12.409 -49.061  1.00 43.41           C
ATOM   1530  OH  TYR A 149      13.550  11.663 -48.828  1.00 46.71           O
ATOM   1531  H   TYR A 149       7.948  15.083 -52.037  1.00 35.85           H
ATOM   1532  HA  TYR A 149       7.781  13.156 -50.010  1.00 37.55           H
ATOM   1533  HB2 TYR A 149       9.214  15.784 -50.310  1.00 33.32           H
ATOM   1534  HB3 TYR A 149       8.865  15.388 -48.689  1.00 33.32           H
ATOM   1535  HD1 TYR A 149       9.309  12.959 -47.818  1.00 40.48           H
ATOM   1536  HD2 TYR A 149      11.320  14.884 -51.129  1.00 41.57           H
ATOM   1537  HE1 TYR A 149      11.291  11.554 -47.427  1.00 39.96           H
ATOM   1538  HE2 TYR A 149      13.309  13.466 -50.735  1.00 42.24           H
ATOM   1539  HH  TYR A 149      13.396  10.949 -48.177  1.00 46.71           H
ATOM   1540  N   ASN A 150       6.008  15.859 -49.352  1.00 34.61           N
ATOM   1541  CA  ASN A 150       5.089  16.429 -48.380  1.00 33.44           C
ATOM   1542  C   ASN A 150       3.638  16.073 -48.645  1.00 35.99           C
ATOM   1543  O   ASN A 150       2.818  16.172 -47.727  1.00 39.73           O
ATOM   1544  CB  ASN A 150       5.310  17.944 -48.360  1.00 31.56           C
ATOM   1545  CG  ASN A 150       6.673  18.244 -47.750  1.00 41.16           C
ATOM   1546  ND2 ASN A 150       7.637  18.675 -48.549  1.00 35.91           N
ATOM   1547  OD1 ASN A 150       6.880  18.011 -46.570  1.00 41.42           O
ATOM   1548  H   ASN A 150       6.301  16.444 -50.124  1.00 34.61           H
ATOM   1549  HA  ASN A 150       5.348  16.058 -47.394  1.00 33.44           H
ATOM   1550  HB2 ASN A 150       5.194  18.391 -49.349  1.00 31.56           H
ATOM   1551  HB3 ASN A 150       4.561  18.404 -47.725  1.00 31.56           H
ATOM   1552 HD21 ASN A 150       7.474  18.710 -49.554  1.00 35.91           H
ATOM   1553 HD22 ASN A 150       8.552  18.860 -48.170  1.00 35.91           H
ATOM   1554  N   MET A 151       3.297  15.653 -49.862  1.00 32.39           N
ATOM   1555  CA  MET A 151       2.027  14.963 -50.052  1.00 31.58           C
```

ATOM 1556 C MET A 151 1.959 13.727 -49.164 1.00 36.94 C
ATOM 1557 O MET A 151 0.981 13.520 -48.438 1.00 33.47 O
ATOM 1558 CB MET A 151 1.931 14.548 -51.542 1.00 30.72 C
ATOM 1559 CG MET A 151 0.704 13.713 -51.971 1.00 30.96 C
ATOM 1560 SD MET A 151 0.909 12.953 -53.610 1.00 39.03 S
ATOM 1561 CE MET A 151 -0.819 12.648 -54.071 1.00 51.80 C
ATOM 1562 H MET A 151 3.968 15.625 -50.618 1.00 32.39 H
ATOM 1563 HA MET A 151 1.213 15.634 -49.770 1.00 31.58 H
ATOM 1564 HB2 MET A 151 1.976 15.452 -52.145 1.00 30.72 H
ATOM 1565 HB3 MET A 151 2.824 13.987 -51.819 1.00 30.72 H
ATOM 1566 HG2 MET A 151 0.507 12.896 -51.276 1.00 30.96 H
ATOM 1567 HG3 MET A 151 -0.188 14.340 -51.971 1.00 30.96 H
ATOM 1568 HE1 MET A 151 -1.364 13.584 -54.183 1.00 51.80 H
ATOM 1569 HE2 MET A 151 -0.871 12.108 -55.018 1.00 51.80 H
ATOM 1570 HE3 MET A 151 -1.324 12.054 -53.313 1.00 51.80 H
ATOM 1571 N PHE A 152 3.006 12.903 -49.191 1.00 31.61 N
ATOM 1572 CA PHE A 152 2.995 11.671 -48.413 1.00 34.82 C
ATOM 1573 C PHE A 152 3.178 11.940 -46.923 1.00 30.28 C
ATOM 1574 O PHE A 152 2.533 11.290 -46.094 1.00 33.97 O
ATOM 1575 CB PHE A 152 4.067 10.685 -48.916 1.00 30.42 C
ATOM 1576 CG PHE A 152 3.750 10.016 -50.241 1.00 36.72 C
ATOM 1577 CD1 PHE A 152 2.652 9.135 -50.346 1.00 36.23 C
ATOM 1578 CD2 PHE A 152 4.511 10.305 -51.391 1.00 38.00 C
ATOM 1579 CE1 PHE A 152 2.344 8.560 -51.570 1.00 41.86 C
ATOM 1580 CE2 PHE A 152 4.164 9.745 -52.611 1.00 37.87 C
ATOM 1581 CZ PHE A 152 3.084 8.879 -52.699 1.00 36.08 C
ATOM 1582 H PHE A 152 3.805 13.082 -49.785 1.00 31.61 H
ATOM 1583 HA PHE A 152 2.026 11.179 -48.526 1.00 34.82 H
ATOM 1584 HB2 PHE A 152 5.036 11.181 -48.977 1.00 30.42 H
ATOM 1585 HB3 PHE A 152 4.193 9.888 -48.184 1.00 30.42 H
ATOM 1586 HD1 PHE A 152 2.047 8.905 -49.480 1.00 36.23 H
ATOM 1587 HD2 PHE A 152 5.368 10.956 -51.331 1.00 38.00 H
ATOM 1588 HE1 PHE A 152 1.511 7.880 -51.650 1.00 41.86 H
ATOM 1589 HE2 PHE A 152 4.741 9.980 -53.492 1.00 37.87 H
ATOM 1590 HZ PHE A 152 2.826 8.444 -53.651 1.00 36.08 H
ATOM 1591 N THR A 153 4.047 12.889 -46.563 1.00 29.43 N
ATOM 1592 CA THR A 153 4.245 13.211 -45.152 1.00 32.64 C
ATOM 1593 C THR A 153 2.968 13.756 -44.525 1.00 40.17 C
ATOM 1594 O THR A 153 2.606 13.376 -43.405 1.00 37.00 O
ATOM 1595 CB THR A 153 5.393 14.209 -44.993 1.00 31.99 C

```
ATOM   1596  CG2 THR A 153       5.558  14.611 -43.527  1.00 39.47           C
ATOM   1597  OG1 THR A 153       6.611  13.610 -45.450  1.00 42.03           O
ATOM   1598  H   THR A 153       4.590  13.396 -47.250  1.00 29.43           H
ATOM   1599  HA  THR A 153       4.513  12.291 -44.626  1.00 32.64           H
ATOM   1600  HB  THR A 153       5.196  15.108 -45.566  1.00 31.99           H
ATOM   1601  HG1 THR A 153       6.547  13.481 -46.402  1.00 42.03           H
ATOM   1602 HG21 THR A 153       6.394  15.302 -43.415  1.00 39.47           H
ATOM   1603 HG22 THR A 153       4.690  15.099 -43.088  1.00 39.47           H
ATOM   1604 HG23 THR A 153       5.779  13.739 -42.910  1.00 39.47           H
ATOM   1605  N   SER A 154       2.269  14.648 -45.232  1.00 32.83           N
ATOM   1606  CA  SER A 154       0.988  15.145 -44.741  1.00 32.13           C
ATOM   1607  C   SER A 154      -0.010  14.006 -44.580  1.00 35.52           C
ATOM   1608  O   SER A 154      -0.648  13.862 -43.530  1.00 30.51           O
ATOM   1609  CB  SER A 154       0.448  16.222 -45.710  1.00 30.48           C
ATOM   1610  OG  SER A 154      -0.864  16.626 -45.377  1.00 40.32           O
ATOM   1611  H   SER A 154       2.574  14.963 -46.144  1.00 32.83           H
ATOM   1612  HA  SER A 154       1.140  15.609 -43.764  1.00 32.13           H
ATOM   1613  HB2 SER A 154       1.100  17.097 -45.713  1.00 30.48           H
ATOM   1614  HB3 SER A 154       0.421  15.840 -46.732  1.00 30.48           H
ATOM   1615  HG  SER A 154      -0.890  17.598 -45.358  1.00 40.32           H
ATOM   1616  N   ILE A 155      -0.138  13.172 -45.610  1.00 29.67           N
ATOM   1617  CA  ILE A 155      -1.135  12.110 -45.593  1.00 28.67           C
ATOM   1618  C   ILE A 155      -0.782  11.044 -44.558  1.00 31.40           C
ATOM   1619  O   ILE A 155      -1.655  10.559 -43.829  1.00 30.86           O
ATOM   1620  CB  ILE A 155      -1.295  11.526 -47.025  1.00 35.93           C
ATOM   1621  CG1 ILE A 155      -1.950  12.582 -47.957  1.00 34.60           C
ATOM   1622  CG2 ILE A 155      -2.072  10.205 -47.112  1.00 31.50           C
ATOM   1623  CD1 ILE A 155      -1.833  12.276 -49.456  1.00 40.15           C
ATOM   1624  H   ILE A 155       0.395  13.301 -46.460  1.00 29.67           H
ATOM   1625  HA  ILE A 155      -2.095  12.544 -45.313  1.00 28.67           H
ATOM   1626  HB  ILE A 155      -0.293  11.323 -47.407  1.00 35.93           H
ATOM   1627 HG12 ILE A 155      -3.002  12.694 -47.706  1.00 34.60           H
ATOM   1628 HG13 ILE A 155      -1.523  13.570 -47.783  1.00 34.60           H
ATOM   1629 HG21 ILE A 155      -2.159   9.900 -48.152  1.00 31.50           H
ATOM   1630 HG22 ILE A 155      -1.575   9.390 -46.587  1.00 31.50           H
ATOM   1631 HG23 ILE A 155      -3.080  10.309 -46.710  1.00 31.50           H
ATOM   1632 HD11 ILE A 155      -1.663  13.191 -50.023  1.00 40.15           H
ATOM   1633 HD12 ILE A 155      -1.009  11.596 -49.669  1.00 40.15           H
ATOM   1634 HD13 ILE A 155      -2.752  11.831 -49.835  1.00 40.15           H
ATOM   1635  N   PHE A 156       0.495  10.659 -44.476  1.00 27.48           N
```

ATOM 1636 CA PHE A 156 0.881 9.635 -43.509 1.00 34.73 C
ATOM 1637 C PHE A 156 0.725 10.129 -42.076 1.00 36.96 C
ATOM 1638 O PHE A 156 0.428 9.332 -41.179 1.00 32.87 O
ATOM 1639 CB PHE A 156 2.321 9.176 -43.753 1.00 35.29 C
ATOM 1640 CG PHE A 156 2.521 8.448 -45.059 1.00 35.90 C
ATOM 1641 CD1 PHE A 156 1.441 7.961 -45.778 1.00 39.73 C
ATOM 1642 CD2 PHE A 156 3.797 8.247 -45.560 1.00 34.82 C
ATOM 1643 CE1 PHE A 156 1.630 7.293 -46.978 1.00 37.95 C
ATOM 1644 CE2 PHE A 156 3.994 7.582 -46.758 1.00 39.17 C
ATOM 1645 CZ PHE A 156 2.909 7.104 -47.465 1.00 36.27 C
ATOM 1646 H PHE A 156 1.213 11.032 -45.088 1.00 27.48 H
ATOM 1647 HA PHE A 156 0.205 8.781 -43.564 1.00 34.73 H
ATOM 1648 HB2 PHE A 156 3.001 10.029 -43.716 1.00 35.29 H
ATOM 1649 HB3 PHE A 156 2.635 8.499 -42.958 1.00 35.29 H
ATOM 1650 HD1 PHE A 156 0.418 8.068 -45.461 1.00 39.73 H
ATOM 1651 HD2 PHE A 156 4.652 8.616 -45.013 1.00 34.82 H
ATOM 1652 HE1 PHE A 156 0.780 6.920 -47.521 1.00 37.95 H
ATOM 1653 HE2 PHE A 156 4.995 7.441 -47.138 1.00 39.17 H
ATOM 1654 HZ PHE A 156 3.060 6.583 -48.399 1.00 36.27 H
ATOM 1655 N THR A 157 0.912 11.431 -41.840 1.00 36.14 N
ATOM 1656 CA THR A 157 0.754 11.963 -40.490 1.00 29.55 C
ATOM 1657 C THR A 157 -0.707 11.959 -40.060 1.00 36.33 C
ATOM 1658 O THR A 157 -1.019 11.629 -38.911 1.00 37.69 O
ATOM 1659 CB THR A 157 1.336 13.374 -40.404 1.00 28.36 C
ATOM 1660 CG2 THR A 157 1.236 13.909 -38.973 1.00 31.15 C
ATOM 1661 OG1 THR A 157 2.713 13.341 -40.796 1.00 31.50 O
ATOM 1662 H THR A 157 1.152 12.075 -42.582 1.00 36.14 H
ATOM 1663 HA THR A 157 1.312 11.328 -39.798 1.00 29.55 H
ATOM 1664 HB THR A 157 0.818 14.035 -41.093 1.00 28.36 H
ATOM 1665 HG1 THR A 157 2.749 13.224 -41.754 1.00 31.50 H
ATOM 1666 HG21 THR A 157 1.804 14.833 -38.881 1.00 31.15 H
ATOM 1667 HG22 THR A 157 0.211 14.137 -38.678 1.00 31.15 H
ATOM 1668 HG23 THR A 157 1.649 13.206 -38.248 1.00 31.15 H
ATOM 1669 N LEU A 158 -1.623 12.314 -40.964 1.00 32.63 N
ATOM 1670 CA LEU A 158 -3.029 12.302 -40.580 1.00 37.38 C
ATOM 1671 C LEU A 158 -3.557 10.876 -40.452 1.00 34.87 C
ATOM 1672 O LEU A 158 -4.493 10.635 -39.682 1.00 39.21 O
ATOM 1673 CB LEU A 158 -3.859 13.128 -41.568 1.00 43.55 C
ATOM 1674 CG LEU A 158 -4.079 12.665 -43.005 1.00 41.78 C
ATOM 1675 CD1 LEU A 158 -5.303 11.773 -43.095 1.00 34.21 C

ATOM 1676 CD2 LEU A 158 -4.220 13.866 -43.935 1.00 39.05 C
ATOM 1677 H LEU A 158 -1.377 12.598 -41.904 1.00 32.63 H
ATOM 1678 HA LEU A 158 -3.138 12.786 -39.606 1.00 37.38 H
ATOM 1679 HB2 LEU A 158 -4.823 13.359 -41.112 1.00 43.55 H
ATOM 1680 HB3 LEU A 158 -3.366 14.101 -41.604 1.00 43.55 H
ATOM 1681 HG LEU A 158 -3.208 12.132 -43.328 1.00 41.78 H
ATOM 1682 HD11 LEU A 158 -6.042 12.153 -43.801 1.00 34.21 H
ATOM 1683 HD12 LEU A 158 -5.021 10.776 -43.437 1.00 34.21 H
ATOM 1684 HD13 LEU A 158 -5.820 11.646 -42.143 1.00 34.21 H
ATOM 1685 HD21 LEU A 158 -4.396 13.556 -44.964 1.00 39.05 H
ATOM 1686 HD22 LEU A 158 -5.048 14.510 -43.635 1.00 39.05 H
ATOM 1687 HD23 LEU A 158 -3.315 14.474 -43.938 1.00 39.05 H
ATOM 1688 N CYS A 159 -2.972 9.921 -41.184 1.00 35.03 N
ATOM 1689 CA CYS A 159 -3.260 8.515 -40.912 1.00 34.02 C
ATOM 1690 C CYS A 159 -2.764 8.118 -39.529 1.00 29.94 C
ATOM 1691 O CYS A 159 -3.438 7.370 -38.811 1.00 37.46 O
ATOM 1692 CB CYS A 159 -2.617 7.615 -41.968 1.00 33.73 C
ATOM 1693 SG CYS A 159 -3.343 7.719 -43.613 1.00 42.02 S
ATOM 1694 H CYS A 159 -2.242 10.139 -41.849 1.00 35.03 H
ATOM 1695 HA CYS A 159 -4.340 8.356 -40.943 1.00 34.02 H
ATOM 1696 HB2 CYS A 159 -1.551 7.823 -42.056 1.00 33.73 H
ATOM 1697 HB3 CYS A 159 -2.694 6.571 -41.664 1.00 33.73 H
ATOM 1698 HG CYS A 159 -2.909 8.957 -43.874 1.00 42.02 H
ATOM 1699 N THR A 160 -1.574 8.595 -39.153 1.00 35.02 N
ATOM 1700 CA THR A 160 -1.020 8.286 -37.840 1.00 39.51 C
ATOM 1701 C THR A 160 -1.908 8.825 -36.727 1.00 37.17 C
ATOM 1702 O THR A 160 -2.106 8.161 -35.703 1.00 39.82 O
ATOM 1703 CB THR A 160 0.393 8.858 -37.723 1.00 37.94 C
ATOM 1704 CG2 THR A 160 0.972 8.574 -36.345 1.00 31.21 C
ATOM 1705 OG1 THR A 160 1.232 8.260 -38.719 1.00 35.71 O
ATOM 1706 H THR A 160 -1.034 9.192 -39.764 1.00 35.02 H
ATOM 1707 HA THR A 160 -0.969 7.199 -37.738 1.00 39.51 H
ATOM 1708 HB THR A 160 0.411 9.933 -37.884 1.00 37.94 H
ATOM 1709 HG1 THR A 160 0.904 8.517 -39.588 1.00 35.71 H
ATOM 1710 HG21 THR A 160 2.016 8.821 -36.388 1.00 31.21 H
ATOM 1711 HG22 THR A 160 0.533 9.208 -35.574 1.00 31.21 H
ATOM 1712 HG23 THR A 160 0.836 7.538 -36.042 1.00 31.21 H
ATOM 1713 N MET A 161 -2.456 10.027 -36.912 1.00 35.47 N
ATOM 1714 CA MET A 161 -3.374 10.575 -35.922 1.00 35.47 C
ATOM 1715 C MET A 161 -4.644 9.738 -35.815 1.00 34.19 C

ATOM 1716 O MET A 161 -5.222 9.631 -34.729 1.00 35.82 O
ATOM 1717 CB MET A 161 -3.703 12.028 -36.265 1.00 40.25 C
ATOM 1718 CG MET A 161 -2.470 12.924 -36.312 1.00 49.70 C
ATOM 1719 SD MET A 161 -2.828 14.667 -36.605 1.00 53.76 S
ATOM 1720 CE MET A 161 -3.570 15.122 -35.040 1.00 66.13 C
ATOM 1721 H MET A 161 -2.263 10.573 -37.742 1.00 35.47 H
ATOM 1722 HA MET A 161 -2.881 10.566 -34.948 1.00 35.47 H
ATOM 1723 HB2 MET A 161 -4.215 12.078 -37.228 1.00 40.25 H
ATOM 1724 HB3 MET A 161 -4.409 12.416 -35.530 1.00 40.25 H
ATOM 1725 HG2 MET A 161 -1.954 12.860 -35.353 1.00 49.70 H
ATOM 1726 HG3 MET A 161 -1.738 12.616 -37.030 1.00 49.70 H
ATOM 1727 HE1 MET A 161 -3.718 16.199 -34.983 1.00 66.13 H
ATOM 1728 HE2 MET A 161 -4.536 14.636 -34.908 1.00 66.13 H
ATOM 1729 HE3 MET A 161 -2.920 14.833 -34.214 1.00 66.13 H
ATOM 1730 N SER A 162 -5.083 9.125 -36.916 1.00 28.80 N
ATOM 1731 CA SER A 162 -6.237 8.233 -36.841 1.00 30.96 C
ATOM 1732 C SER A 162 -5.893 6.949 -36.097 1.00 27.50 C
ATOM 1733 O SER A 162 -6.664 6.490 -35.246 1.00 33.85 O
ATOM 1734 CB SER A 162 -6.751 7.912 -38.245 1.00 33.77 C
ATOM 1735 OG SER A 162 -7.884 7.067 -38.177 1.00 47.77 O
ATOM 1736 H SER A 162 -4.607 9.219 -37.803 1.00 28.80 H
ATOM 1737 HA SER A 162 -7.038 8.733 -36.294 1.00 30.96 H
ATOM 1738 HB2 SER A 162 -7.028 8.829 -38.767 1.00 33.77 H
ATOM 1739 HB3 SER A 162 -5.992 7.417 -38.850 1.00 33.77 H
ATOM 1740 HG SER A 162 -8.627 7.572 -37.836 1.00 47.77 H
ATOM 1741 N VAL A 163 -4.747 6.345 -36.421 1.00 30.31 N
ATOM 1742 CA VAL A 163 -4.311 5.136 -35.728 1.00 30.40 C
ATOM 1743 C VAL A 163 -4.081 5.426 -34.250 1.00 34.14 C
ATOM 1744 O VAL A 163 -4.410 4.611 -33.379 1.00 37.21 O
ATOM 1745 CB VAL A 163 -3.044 4.572 -36.397 1.00 31.52 C
ATOM 1746 CG1 VAL A 163 -2.474 3.417 -35.586 1.00 32.66 C
ATOM 1747 CG2 VAL A 163 -3.347 4.134 -37.825 1.00 26.59 C
ATOM 1748 H VAL A 163 -4.143 6.720 -37.142 1.00 30.31 H
ATOM 1749 HA VAL A 163 -5.103 4.390 -35.807 1.00 30.40 H
ATOM 1750 HB VAL A 163 -2.289 5.358 -36.448 1.00 31.52 H
ATOM 1751 HG11 VAL A 163 -1.684 2.910 -36.141 1.00 32.66 H
ATOM 1752 HG12 VAL A 163 -2.021 3.747 -34.651 1.00 32.66 H
ATOM 1753 HG13 VAL A 163 -3.236 2.672 -35.353 1.00 32.66 H
ATOM 1754 HG21 VAL A 163 -2.430 3.861 -38.348 1.00 26.59 H
ATOM 1755 HG22 VAL A 163 -3.996 3.259 -37.837 1.00 26.59 H

ATOM 1756 HG23 VAL A 163 -3.835 4.906 -38.416 1.00 26.59 H
ATOM 1757 N ASP A 164 -3.512 6.593 -33.948 1.00 33.76 N
ATOM 1758 CA ASP A 164 -3.257 6.973 -32.564 1.00 30.01 C
ATOM 1759 C ASP A 164 -4.549 7.039 -31.758 1.00 34.12 C
ATOM 1760 O ASP A 164 -4.601 6.574 -30.613 1.00 35.72 O
ATOM 1761 CB ASP A 164 -2.532 8.317 -32.535 1.00 34.22 C
ATOM 1762 CG ASP A 164 -2.344 8.849 -31.135 1.00 38.67 C
ATOM 1763 OD1 ASP A 164 -1.590 8.222 -30.361 1.00 41.22 O
ATOM 1764 OD2 ASP A 164 -2.941 9.900 -30.816 1.00 42.20 O1-
ATOM 1765 H ASP A 164 -3.210 7.234 -34.673 1.00 33.76 H
ATOM 1766 HA ASP A 164 -2.637 6.200 -32.111 1.00 30.01 H
ATOM 1767 HB2 ASP A 164 -1.555 8.257 -33.016 1.00 34.22 H
ATOM 1768 HB3 ASP A 164 -3.087 9.057 -33.111 1.00 34.22 H
ATOM 1769 N ARG A 165 -5.604 7.614 -32.340 1.00 31.74 N
ATOM 1770 CA ARG A 165 -6.882 7.694 -31.643 1.00 36.70 C
ATOM 1771 C ARG A 165 -7.556 6.331 -31.555 1.00 38.55 C
ATOM 1772 O ARG A 165 -8.209 6.025 -30.549 1.00 34.10 O
ATOM 1773 CB ARG A 165 -7.790 8.707 -32.343 1.00 37.46 C
ATOM 1774 CG ARG A 165 -7.262 10.130 -32.279 1.00 39.84 C
ATOM 1775 CD ARG A 165 -7.852 11.001 -33.378 1.00 47.40 C
ATOM 1776 NE ARG A 165 -7.454 12.398 -33.230 1.00 52.02 N
ATOM 1777 CZ ARG A 165 -7.756 13.359 -34.096 1.00 56.13 C
ATOM 1778 NH1 ARG A 165 -8.460 13.076 -35.186 1.00 47.41 N1+
ATOM 1779 NH2 ARG A 165 -7.350 14.601 -33.872 1.00 58.79 N1+
ATOM 1780 H ARG A 165 -5.543 8.003 -33.271 1.00 31.74 H
ATOM 1781 HA ARG A 165 -6.711 8.050 -30.624 1.00 36.70 H
ATOM 1782 HB2 ARG A 165 -7.902 8.403 -33.386 1.00 37.46 H
ATOM 1783 HB3 ARG A 165 -8.792 8.690 -31.913 1.00 37.46 H
ATOM 1784 HG2 ARG A 165 -7.380 10.569 -31.287 1.00 39.84 H
ATOM 1785 HG3 ARG A 165 -6.187 10.112 -32.452 1.00 39.84 H
ATOM 1786 HD2 ARG A 165 -7.461 10.679 -34.342 1.00 47.40 H
ATOM 1787 HD3 ARG A 165 -8.936 10.898 -33.425 1.00 47.40 H
ATOM 1788 HE ARG A 165 -6.926 12.631 -32.401 1.00 52.02 H
ATOM 1789 HH11 ARG A 165 -8.711 12.119 -35.403 1.00 47.41 H
ATOM 1790 HH12 ARG A 165 -8.712 13.806 -35.835 1.00 47.41 H
ATOM 1791 HH21 ARG A 165 -6.820 14.825 -33.042 1.00 58.79 H
ATOM 1792 HH22 ARG A 165 -7.591 15.343 -34.513 1.00 58.79 H
ATOM 1793 N TYR A 166 -7.414 5.504 -32.593 1.00 35.25 N
ATOM 1794 CA TYR A 166 -7.940 4.146 -32.529 1.00 33.77 C
ATOM 1795 C TYR A 166 -7.273 3.350 -31.412 1.00 28.93 C

ATOM 1796 O TYR A 166 -7.945 2.634 -30.661 1.00 36.35 O
ATOM 1797 CB TYR A 166 -7.754 3.451 -33.880 1.00 32.15 C
ATOM 1798 CG TYR A 166 -7.803 1.944 -33.799 1.00 32.95 C
ATOM 1799 CD1 TYR A 166 -9.014 1.275 -33.710 1.00 33.23 C
ATOM 1800 CD2 TYR A 166 -6.634 1.190 -33.815 1.00 34.57 C
ATOM 1801 CE1 TYR A 166 -9.065 -0.103 -33.633 1.00 37.25 C
ATOM 1802 CE2 TYR A 166 -6.674 -0.189 -33.739 1.00 31.86 C
ATOM 1803 CZ TYR A 166 -7.894 -0.830 -33.647 1.00 36.03 C
ATOM 1804 OH TYR A 166 -7.946 -2.203 -33.569 1.00 44.80 O
ATOM 1805 H TYR A 166 -6.903 5.779 -33.422 1.00 35.25 H
ATOM 1806 HA TYR A 166 -9.010 4.197 -32.317 1.00 33.77 H
ATOM 1807 HB2 TYR A 166 -8.536 3.800 -34.549 1.00 32.15 H
ATOM 1808 HB3 TYR A 166 -6.803 3.733 -34.331 1.00 32.15 H
ATOM 1809 HD1 TYR A 166 -9.936 1.838 -33.691 1.00 33.23 H
ATOM 1810 HD2 TYR A 166 -5.677 1.686 -33.888 1.00 34.57 H
ATOM 1811 HE1 TYR A 166 -10.015 -0.607 -33.544 1.00 37.25 H
ATOM 1812 HE2 TYR A 166 -5.755 -0.756 -33.749 1.00 31.86 H
ATOM 1813 HH TYR A 166 -7.083 -2.606 -33.450 1.00 44.80 H
ATOM 1814 N ILE A 167 -5.950 3.475 -31.281 1.00 30.53 N
ATOM 1815 CA ILE A 167 -5.230 2.763 -30.229 1.00 33.93 C
ATOM 1816 C ILE A 167 -5.670 3.249 -28.852 1.00 37.15 C
ATOM 1817 O ILE A 167 -5.831 2.451 -27.920 1.00 34.00 O
ATOM 1818 CB ILE A 167 -3.712 2.917 -30.436 1.00 39.01 C
ATOM 1819 CG1 ILE A 167 -3.264 2.111 -31.659 1.00 38.10 C
ATOM 1820 CG2 ILE A 167 -2.934 2.495 -29.188 1.00 35.40 C
ATOM 1821 CD1 ILE A 167 -1.782 2.220 -31.953 1.00 35.60 C
ATOM 1822 H ILE A 167 -5.411 4.051 -31.916 1.00 30.53 H
ATOM 1823 HA ILE A 167 -5.483 1.702 -30.296 1.00 33.93 H
ATOM 1824 HB ILE A 167 -3.487 3.968 -30.628 1.00 39.01 H
ATOM 1825 HG12 ILE A 167 -3.497 1.059 -31.490 1.00 38.10 H
ATOM 1826 HG13 ILE A 167 -3.826 2.382 -32.548 1.00 38.10 H
ATOM 1827 HG21 ILE A 167 -1.857 2.534 -29.345 1.00 35.40 H
ATOM 1828 HG22 ILE A 167 -3.107 3.165 -28.349 1.00 35.40 H
ATOM 1829 HG23 ILE A 167 -3.178 1.481 -28.875 1.00 35.40 H
ATOM 1830 HD11 ILE A 167 -1.573 1.927 -32.982 1.00 35.60 H
ATOM 1831 HD12 ILE A 167 -1.429 3.243 -31.831 1.00 35.60 H
ATOM 1832 HD13 ILE A 167 -1.189 1.571 -31.308 1.00 35.60 H
ATOM 1833 N ALA A 168 -5.895 4.557 -28.708 1.00 34.16 N
ATOM 1834 CA ALA A 168 -6.291 5.111 -27.416 1.00 33.98 C
ATOM 1835 C ALA A 168 -7.602 4.509 -26.925 1.00 36.35 C

ATOM 1836 O ALA A 168 -7.765 4.248 -25.726 1.00 39.08 O
ATOM 1837 CB ALA A 168 -6.409 6.631 -27.516 1.00 33.75 C
ATOM 1838 H ALA A 168 -5.755 5.200 -29.476 1.00 34.16 H
ATOM 1839 HA ALA A 168 -5.514 4.881 -26.688 1.00 33.98 H
ATOM 1840 HB1 ALA A 168 -6.584 7.077 -26.537 1.00 33.75 H
ATOM 1841 HB2 ALA A 168 -5.498 7.064 -27.928 1.00 33.75 H
ATOM 1842 HB3 ALA A 168 -7.230 6.931 -28.168 1.00 33.75 H
ATOM 1843 N VAL A 169 -8.544 4.275 -27.831 1.00 33.09 N
ATOM 1844 CA VAL A 169 -9.868 3.792 -27.450 1.00 33.26 C
ATOM 1845 C VAL A 169 -9.922 2.272 -27.423 1.00 36.39 C
ATOM 1846 O VAL A 169 -10.458 1.676 -26.485 1.00 35.47 O
ATOM 1847 CB VAL A 169 -10.933 4.374 -28.404 1.00 37.14 C
ATOM 1848 CG1 VAL A 169 -12.293 3.754 -28.131 1.00 36.50 C
ATOM 1849 CG2 VAL A 169 -10.996 5.882 -28.262 1.00 35.69 C
ATOM 1850 H VAL A 169 -8.388 4.494 -28.806 1.00 33.09 H
ATOM 1851 HA VAL A 169 -10.120 4.137 -26.444 1.00 33.26 H
ATOM 1852 HB VAL A 169 -10.650 4.128 -29.428 1.00 37.14 H
ATOM 1853 HG11 VAL A 169 -13.035 4.223 -28.770 1.00 36.50 H
ATOM 1854 HG12 VAL A 169 -12.329 2.698 -28.386 1.00 36.50 H
ATOM 1855 HG13 VAL A 169 -12.607 3.878 -27.094 1.00 36.50 H
ATOM 1856 HG21 VAL A 169 -11.814 6.310 -28.841 1.00 35.69 H
ATOM 1857 HG22 VAL A 169 -11.158 6.175 -27.224 1.00 35.69 H
ATOM 1858 HG23 VAL A 169 -10.077 6.361 -28.600 1.00 35.69 H
ATOM 1859 N CYS A 170 -9.369 1.620 -28.447 1.00 33.56 N
ATOM 1860 CA CYS A 170 -9.541 0.182 -28.605 1.00 38.11 C
ATOM 1861 C CYS A 170 -8.427 -0.636 -27.969 1.00 36.89 C
ATOM 1862 O CYS A 170 -8.627 -1.830 -27.718 1.00 40.09 O
ATOM 1863 CB CYS A 170 -9.658 -0.175 -30.092 1.00 34.39 C
ATOM 1864 SG CYS A 170 -11.144 0.511 -30.883 1.00 40.02 S
ATOM 1865 H CYS A 170 -8.913 2.118 -29.202 1.00 33.56 H
ATOM 1866 HA CYS A 170 -10.477 -0.135 -28.147 1.00 38.11 H
ATOM 1867 HB2 CYS A 170 -8.779 0.161 -30.642 1.00 34.39 H
ATOM 1868 HB3 CYS A 170 -9.699 -1.257 -30.223 1.00 34.39 H
ATOM 1869 HG CYS A 170 -12.021 -0.249 -30.221 1.00 40.02 H
ATOM 1870 N HIS A 171 -7.269 -0.036 -27.701 1.00 32.66 N
ATOM 1871 CA HIS A 171 -6.164 -0.713 -27.019 1.00 33.10 C
ATOM 1872 C HIS A 171 -5.583 0.209 -25.953 1.00 34.78 C
ATOM 1873 O HIS A 171 -4.414 0.605 -26.023 1.00 36.09 O
ATOM 1874 CB HIS A 171 -5.092 -1.142 -28.021 1.00 39.44 C
ATOM 1875 CG HIS A 171 -5.622 -1.984 -29.141 1.00 44.90 C

ATOM 1876 CD2 HIS A 171 -5.968 -1.663 -30.410 1.00 44.59 C
ATOM 1877 ND1 HIS A 171 -5.866 -3.334 -29.007 1.00 46.20 N
ATOM 1878 CE1 HIS A 171 -6.331 -3.810 -30.149 1.00 46.37 C
ATOM 1879 NE2 HIS A 171 -6.402 -2.817 -31.017 1.00 43.11 N
ATOM 1880 H HIS A 171 -7.133 0.944 -27.916 1.00 32.66 H
ATOM 1881 HA HIS A 171 -6.503 -1.614 -26.505 1.00 33.10 H
ATOM 1882 HB2 HIS A 171 -4.611 -0.272 -28.470 1.00 39.44 H
ATOM 1883 HB3 HIS A 171 -4.314 -1.707 -27.510 1.00 39.44 H
ATOM 1884 HD1 HIS A 171 -5.737 -3.871 -28.162 1.00 46.20 H
ATOM 1885 HD2 HIS A 171 -5.944 -0.713 -30.924 1.00 44.59 H
ATOM 1886 HE1 HIS A 171 -6.618 -4.834 -30.339 1.00 46.37 H
ATOM 1887 N PRO A 172 -6.378 0.562 -24.938 1.00 35.33 N
ATOM 1888 CA PRO A 172 -5.914 1.566 -23.963 1.00 34.03 C
ATOM 1889 C PRO A 172 -4.682 1.147 -23.181 1.00 42.17 C
ATOM 1890 O PRO A 172 -3.905 2.015 -22.763 1.00 42.46 O
ATOM 1891 CB PRO A 172 -7.132 1.746 -23.046 1.00 32.29 C
ATOM 1892 CG PRO A 172 -7.886 0.461 -23.163 1.00 33.23 C
ATOM 1893 CD PRO A 172 -7.709 0.031 -24.593 1.00 37.37 C
ATOM 1894 HA PRO A 172 -5.699 2.509 -24.470 1.00 34.03 H
ATOM 1895 HB2 PRO A 172 -6.879 1.988 -22.012 1.00 32.29 H
ATOM 1896 HB3 PRO A 172 -7.747 2.563 -23.426 1.00 32.29 H
ATOM 1897 HG2 PRO A 172 -7.430 -0.281 -22.506 1.00 33.23 H
ATOM 1898 HG3 PRO A 172 -8.933 0.553 -22.874 1.00 33.23 H
ATOM 1899 HD2 PRO A 172 -7.794 -1.051 -24.701 1.00 37.37 H
ATOM 1900 HD3 PRO A 172 -8.456 0.508 -25.225 1.00 37.37 H
ATOM 1901 N VAL A 173 -4.473 -0.153 -22.963 1.00 35.97 N
ATOM 1902 CA VAL A 173 -3.271 -0.592 -22.260 1.00 38.72 C
ATOM 1903 C VAL A 173 -2.040 -0.387 -23.136 1.00 38.98 C
ATOM 1904 O VAL A 173 -1.029 0.172 -22.695 1.00 40.23 O
ATOM 1905 CB VAL A 173 -3.410 -2.057 -21.813 1.00 45.97 C
ATOM 1906 CG1 VAL A 173 -2.090 -2.561 -21.248 1.00 47.22 C
ATOM 1907 CG2 VAL A 173 -4.515 -2.186 -20.774 1.00 45.55 C
ATOM 1908 H VAL A 173 -5.121 -0.856 -23.286 1.00 35.97 H
ATOM 1909 HA VAL A 173 -3.142 0.019 -21.363 1.00 38.72 H
ATOM 1910 HB VAL A 173 -3.677 -2.680 -22.668 1.00 45.97 H
ATOM 1911 HG11 VAL A 173 -2.220 -3.535 -20.776 1.00 47.22 H
ATOM 1912 HG12 VAL A 173 -1.326 -2.700 -22.014 1.00 47.22 H
ATOM 1913 HG13 VAL A 173 -1.693 -1.887 -20.488 1.00 47.22 H
ATOM 1914 HG21 VAL A 173 -4.616 -3.218 -20.436 1.00 45.55 H
ATOM 1915 HG22 VAL A 173 -4.312 -1.571 -19.897 1.00 45.55 H

```
ATOM   1916 HG23 VAL A 173      -5.484  -1.885 -21.173  1.00 45.55           H
ATOM   1917  N   LYS A 174      -2.108  -0.831 -24.392  1.00 37.75           N
ATOM   1918  CA  LYS A 174      -1.011  -0.593 -25.322  1.00 41.25           C
ATOM   1919  C   LYS A 174      -0.820   0.891 -25.610  1.00 42.02           C
ATOM   1920  O   LYS A 174       0.294   1.308 -25.942  1.00 40.81           O
ATOM   1921  CB  LYS A 174      -1.253  -1.363 -26.623  1.00 45.68           C
ATOM   1922  CG  LYS A 174      -1.259  -2.879 -26.447  1.00 60.42           C
ATOM   1923  CD  LYS A 174      -1.661  -3.594 -27.726  1.00 66.87           C
ATOM   1924  CE  LYS A 174      -1.685  -5.103 -27.528  1.00 70.70           C
ATOM   1925  NZ  LYS A 174      -2.132  -5.817 -28.757  1.00 73.90           N1+
ATOM   1926  H   LYS A 174      -2.941  -1.277 -24.747  1.00 37.75           H
ATOM   1927  HA  LYS A 174      -0.086  -0.966 -24.874  1.00 41.25           H
ATOM   1928  HB2 LYS A 174      -2.194  -1.033 -27.063  1.00 45.68           H
ATOM   1929  HB3 LYS A 174      -0.479  -1.107 -27.349  1.00 45.68           H
ATOM   1930  HG2 LYS A 174      -0.269  -3.211 -26.130  1.00 60.42           H
ATOM   1931  HG3 LYS A 174      -1.948  -3.162 -25.650  1.00 60.42           H
ATOM   1932  HD2 LYS A 174      -2.643  -3.251 -28.053  1.00 66.87           H
ATOM   1933  HD3 LYS A 174      -0.963  -3.338 -28.524  1.00 66.87           H
ATOM   1934  HE2 LYS A 174      -0.694  -5.466 -27.252  1.00 70.70           H
ATOM   1935  HE3 LYS A 174      -2.362  -5.367 -26.714  1.00 70.70           H
ATOM   1936  HZ1 LYS A 174      -3.061  -5.512 -29.009  1.00 73.90           H
ATOM   1937  HZ2 LYS A 174      -1.496  -5.614 -29.515  1.00 73.90           H
ATOM   1938  HZ3 LYS A 174      -2.139  -6.812 -28.585  1.00 73.90           H
ATOM   1939  N   ALA A 175      -1.878   1.696 -25.475  1.00 36.08           N
ATOM   1940  CA  ALA A 175      -1.752   3.135 -25.676  1.00 31.90           C
ATOM   1941  C   ALA A 175      -0.753   3.754 -24.709  1.00 34.40           C
ATOM   1942  O   ALA A 175      -0.133   4.775 -25.027  1.00 37.29           O
ATOM   1943  CB  ALA A 175      -3.118   3.808 -25.525  1.00 31.50           C
ATOM   1944  H   ALA A 175      -2.791   1.329 -25.238  1.00 36.08           H
ATOM   1945  HA  ALA A 175      -1.392   3.309 -26.691  1.00 31.90           H
ATOM   1946  HB1 ALA A 175      -3.058   4.867 -25.744  1.00 31.50           H
ATOM   1947  HB2 ALA A 175      -3.857   3.361 -26.188  1.00 31.50           H
ATOM   1948  HB3 ALA A 175      -3.498   3.731 -24.510  1.00 31.50           H
ATOM   1949  N   LEU A 176      -0.583   3.153 -23.527  1.00 33.86           N
ATOM   1950  CA  LEU A 176       0.374   3.675 -22.557  1.00 37.50           C
ATOM   1951  C   LEU A 176       1.800   3.627 -23.093  1.00 46.13           C
ATOM   1952  O   LEU A 176       2.621   4.487 -22.750  1.00 42.85           O
ATOM   1953  CB  LEU A 176       0.269   2.890 -21.245  1.00 33.54           C
ATOM   1954  CG  LEU A 176      -1.062   2.977 -20.494  1.00 38.83           C
ATOM   1955  CD1 LEU A 176      -1.088   2.017 -19.304  1.00 39.67           C
```

ATOM 1956 CD2 LEU A 176 -1.326 4.404 -20.030 1.00 34.89 C
ATOM 1957 H LEU A 176 -1.091 2.314 -23.283 1.00 33.86 H
ATOM 1958 HA LEU A 176 0.134 4.722 -22.363 1.00 37.50 H
ATOM 1959 HB2 LEU A 176 0.494 1.844 -21.455 1.00 33.54 H
ATOM 1960 HB3 LEU A 176 1.063 3.216 -20.570 1.00 33.54 H
ATOM 1961 HG LEU A 176 -1.863 2.677 -21.169 1.00 38.83 H
ATOM 1962 HD11 LEU A 176 -1.990 1.404 -19.327 1.00 39.67 H
ATOM 1963 HD12 LEU A 176 -0.241 1.330 -19.314 1.00 39.67 H
ATOM 1964 HD13 LEU A 176 -1.064 2.523 -18.338 1.00 39.67 H
ATOM 1965 HD21 LEU A 176 -1.788 4.457 -19.044 1.00 34.89 H
ATOM 1966 HD22 LEU A 176 -0.416 5.003 -19.990 1.00 34.89 H
ATOM 1967 HD23 LEU A 176 -2.010 4.902 -20.718 1.00 34.89 H
ATOM 1968 N ASP A 177 2.111 2.637 -23.931 1.00 45.43 N
ATOM 1969 CA ASP A 177 3.427 2.532 -24.548 1.00 43.87 C
ATOM 1970 C ASP A 177 3.520 3.286 -25.868 1.00 44.03 C
ATOM 1971 O ASP A 177 4.584 3.828 -26.188 1.00 45.83 O
ATOM 1972 CB ASP A 177 3.784 1.061 -24.786 1.00 51.38 C
ATOM 1973 CG ASP A 177 3.799 0.247 -23.502 1.00 66.86 C
ATOM 1974 OD1 ASP A 177 4.270 0.768 -22.470 1.00 72.14 O
ATOM 1975 OD2 ASP A 177 3.335 -0.913 -23.527 1.00 67.38 O1-
ATOM 1976 H ASP A 177 1.422 1.944 -24.189 1.00 45.43 H
ATOM 1977 HA ASP A 177 4.191 2.954 -23.890 1.00 43.87 H
ATOM 1978 HB2 ASP A 177 3.049 0.619 -25.462 1.00 51.38 H
ATOM 1979 HB3 ASP A 177 4.758 0.962 -25.266 1.00 51.38 H
ATOM 1980 N PHE A 178 2.432 3.342 -26.637 1.00 39.67 N
ATOM 1981 CA PHE A 178 2.500 3.931 -27.971 1.00 36.59 C
ATOM 1982 C PHE A 178 2.442 5.454 -27.939 1.00 42.67 C
ATOM 1983 O PHE A 178 3.086 6.115 -28.761 1.00 41.21 O
ATOM 1984 CB PHE A 178 1.373 3.382 -28.848 1.00 39.40 C
ATOM 1985 CG PHE A 178 1.272 4.052 -30.193 1.00 50.00 C
ATOM 1986 CD1 PHE A 178 2.169 3.746 -31.204 1.00 54.30 C
ATOM 1987 CD2 PHE A 178 0.280 4.988 -30.446 1.00 48.54 C
ATOM 1988 CE1 PHE A 178 2.079 4.360 -32.442 1.00 50.61 C
ATOM 1989 CE2 PHE A 178 0.186 5.606 -31.682 1.00 52.43 C
ATOM 1990 CZ PHE A 178 1.087 5.290 -32.680 1.00 47.01 C
ATOM 1991 H PHE A 178 1.572 2.887 -26.358 1.00 39.67 H
ATOM 1992 HA PHE A 178 3.439 3.647 -28.450 1.00 36.59 H
ATOM 1993 HB2 PHE A 178 1.525 2.314 -29.009 1.00 39.40 H
ATOM 1994 HB3 PHE A 178 0.417 3.471 -28.329 1.00 39.40 H
ATOM 1995 HD1 PHE A 178 2.951 3.022 -31.028 1.00 54.30 H

ATOM 1996 HD2 PHE A 178 -0.435 5.237 -29.678 1.00 48.54 H
ATOM 1997 HE1 PHE A 178 2.791 4.098 -33.207 1.00 50.61 H
ATOM 1998 HE2 PHE A 178 -0.563 6.354 -31.862 1.00 52.43 H
ATOM 1999 HZ PHE A 178 1.005 5.773 -33.642 1.00 47.01 H
ATOM 2000 N ARG A 179 1.681 6.034 -27.016 1.00 36.86 N
ATOM 2001 CA ARG A 179 1.359 7.460 -27.103 1.00 35.56 C
ATOM 2002 C ARG A 179 2.346 8.327 -26.328 1.00 37.85 C
ATOM 2003 O ARG A 179 1.969 9.254 -25.611 1.00 45.57 O
ATOM 2004 CB ARG A 179 -0.069 7.695 -26.633 1.00 38.55 C
ATOM 2005 CG ARG A 179 -1.093 6.859 -27.382 1.00 40.42 C
ATOM 2006 CD ARG A 179 -2.502 7.285 -27.030 1.00 40.38 C
ATOM 2007 NE ARG A 179 -2.949 8.418 -27.834 1.00 35.37 N
ATOM 2008 CZ ARG A 179 -3.931 9.239 -27.479 1.00 42.06 C
ATOM 2009 NH1 ARG A 179 -4.557 9.066 -26.321 1.00 37.45 N1+
ATOM 2010 NH2 ARG A 179 -4.280 10.239 -28.277 1.00 39.24 N1+
ATOM 2011 H ARG A 179 1.185 5.487 -26.323 1.00 36.86 H
ATOM 2012 HA ARG A 179 1.399 7.803 -28.139 1.00 35.56 H
ATOM 2013 HB2 ARG A 179 -0.150 7.492 -25.564 1.00 38.55 H
ATOM 2014 HB3 ARG A 179 -0.317 8.750 -26.768 1.00 38.55 H
ATOM 2015 HG2 ARG A 179 -0.916 7.023 -28.437 1.00 40.42 H
ATOM 2016 HG3 ARG A 179 -0.971 5.787 -27.236 1.00 40.42 H
ATOM 2017 HD2 ARG A 179 -3.218 6.470 -27.094 1.00 40.38 H
ATOM 2018 HD3 ARG A 179 -2.490 7.625 -25.994 1.00 40.38 H
ATOM 2019 HE ARG A 179 -2.519 8.517 -28.753 1.00 35.37 H
ATOM 2020 HH11 ARG A 179 -4.291 8.305 -25.714 1.00 37.45 H
ATOM 2021 HH12 ARG A 179 -5.296 9.692 -26.038 1.00 37.45 H
ATOM 2022 HH21 ARG A 179 -3.817 10.331 -29.179 1.00 39.24 H
ATOM 2023 HH22 ARG A 179 -5.012 10.884 -28.022 1.00 39.24 H
ATOM 2024 N THR A 180 3.613 8.037 -26.485 1.00 36.81 N
ATOM 2025 CA THR A 180 4.678 8.812 -25.876 1.00 39.33 C
ATOM 2026 C THR A 180 5.278 9.777 -26.890 1.00 38.70 C
ATOM 2027 O THR A 180 5.229 9.524 -28.098 1.00 35.56 O
ATOM 2028 CB THR A 180 5.784 7.895 -25.348 1.00 44.70 C
ATOM 2029 CG2 THR A 180 5.215 6.872 -24.379 1.00 47.87 C
ATOM 2030 OG1 THR A 180 6.400 7.211 -26.447 1.00 42.00 O
ATOM 2031 H THR A 180 3.884 7.293 -27.112 1.00 36.81 H
ATOM 2032 HA THR A 180 4.290 9.366 -25.022 1.00 39.33 H
ATOM 2033 HB THR A 180 6.553 8.478 -24.839 1.00 44.70 H
ATOM 2034 HG1 THR A 180 5.849 6.458 -26.681 1.00 42.00 H
ATOM 2035 HG21 THR A 180 6.009 6.250 -23.964 1.00 47.87 H

ATOM 2036 HG22 THR A 180 4.714 7.360 -23.542 1.00 47.87 H
ATOM 2037 HG23 THR A 180 4.495 6.200 -24.847 1.00 47.87 H
ATOM 2038 N PRO A 181 5.845 10.895 -26.429 1.00 40.61 N
ATOM 2039 CA PRO A 181 6.572 11.772 -27.360 1.00 39.23 C
ATOM 2040 C PRO A 181 7.720 11.071 -28.062 1.00 42.47 C
ATOM 2041 O PRO A 181 7.998 11.372 -29.230 1.00 36.97 O
ATOM 2042 CB PRO A 181 7.067 12.916 -26.458 1.00 41.18 C
ATOM 2043 CG PRO A 181 6.937 12.409 -25.049 1.00 43.69 C
ATOM 2044 CD PRO A 181 5.788 11.451 -25.065 1.00 42.41 C
ATOM 2045 HA PRO A 181 5.880 12.152 -28.111 1.00 39.23 H
ATOM 2046 HB2 PRO A 181 8.078 13.259 -26.682 1.00 41.18 H
ATOM 2047 HB3 PRO A 181 6.406 13.773 -26.592 1.00 41.18 H
ATOM 2048 HG2 PRO A 181 7.845 11.862 -24.791 1.00 43.69 H
ATOM 2049 HG3 PRO A 181 6.819 13.206 -24.315 1.00 43.69 H
ATOM 2050 HD2 PRO A 181 5.893 10.712 -24.271 1.00 42.41 H
ATOM 2051 HD3 PRO A 181 4.839 11.974 -24.936 1.00 42.41 H
ATOM 2052 N ARG A 182 8.391 10.132 -27.389 1.00 40.41 N
ATOM 2053 CA ARG A 182 9.486 9.408 -28.026 1.00 39.02 C
ATOM 2054 C ARG A 182 8.993 8.599 -29.221 1.00 39.17 C
ATOM 2055 O ARG A 182 9.622 8.604 -30.286 1.00 38.52 O
ATOM 2056 CB ARG A 182 10.172 8.500 -27.007 1.00 46.89 C
ATOM 2057 CG ARG A 182 11.445 7.845 -27.514 1.00 49.21 C
ATOM 2058 CD ARG A 182 12.062 6.972 -26.433 1.00 61.23 C
ATOM 2059 NE ARG A 182 13.430 6.576 -26.753 1.00 70.36 N
ATOM 2060 CZ ARG A 182 13.754 5.473 -27.418 1.00 68.95 C
ATOM 2061 NH1 ARG A 182 12.805 4.647 -27.839 1.00 62.21 N1+
ATOM 2062 NH2 ARG A 182 15.027 5.194 -27.662 1.00 72.71 N1+
ATOM 2063 H ARG A 182 8.152 9.891 -26.438 1.00 40.41 H
ATOM 2064 HA ARG A 182 10.217 10.136 -28.385 1.00 39.02 H
ATOM 2065 HB2 ARG A 182 10.422 9.101 -26.131 1.00 46.89 H
ATOM 2066 HB3 ARG A 182 9.475 7.736 -26.656 1.00 46.89 H
ATOM 2067 HG2 ARG A 182 11.207 7.208 -28.366 1.00 49.21 H
ATOM 2068 HG3 ARG A 182 12.161 8.586 -27.872 1.00 49.21 H
ATOM 2069 HD2 ARG A 182 12.054 7.503 -25.481 1.00 61.23 H
ATOM 2070 HD3 ARG A 182 11.458 6.081 -26.264 1.00 61.23 H
ATOM 2071 HE ARG A 182 14.161 7.217 -26.479 1.00 70.36 H
ATOM 2072 HH11 ARG A 182 11.834 4.851 -27.651 1.00 62.21 H
ATOM 2073 HH12 ARG A 182 13.043 3.805 -28.343 1.00 62.21 H
ATOM 2074 HH21 ARG A 182 15.758 5.812 -27.340 1.00 72.71 H
ATOM 2075 HH22 ARG A 182 15.280 4.360 -28.172 1.00 72.71 H

ATOM 2076 N ASN A 183 7.871 7.894 -29.064 1.00 36.92 N
ATOM 2077 CA ASN A 183 7.355 7.091 -30.166 1.00 41.23 C
ATOM 2078 C ASN A 183 6.777 7.955 -31.278 1.00 40.20 C
ATOM 2079 O ASN A 183 6.824 7.563 -32.450 1.00 35.89 O
ATOM 2080 CB ASN A 183 6.303 6.108 -29.658 1.00 36.80 C
ATOM 2081 CG ASN A 183 6.917 4.848 -29.092 1.00 51.14 C
ATOM 2082 ND2 ASN A 183 6.236 4.238 -28.133 1.00 45.86 N
ATOM 2083 OD1 ASN A 183 7.993 4.425 -29.516 1.00 51.81 O
ATOM 2084 H ASN A 183 7.362 7.888 -28.189 1.00 36.92 H
ATOM 2085 HA ASN A 183 8.211 6.553 -30.576 1.00 41.23 H
ATOM 2086 HB2 ASN A 183 5.629 6.590 -28.950 1.00 36.80 H
ATOM 2087 HB3 ASN A 183 5.678 5.765 -30.485 1.00 36.80 H
ATOM 2088 HD21 ASN A 183 5.347 4.587 -27.802 1.00 45.86 H
ATOM 2089 HD22 ASN A 183 6.603 3.394 -27.718 1.00 45.86 H
ATOM 2090 N ALA A 184 6.229 9.124 -30.939 1.00 40.21 N
ATOM 2091 CA ALA A 184 5.719 10.020 -31.971 1.00 38.93 C
ATOM 2092 C ALA A 184 6.839 10.493 -32.886 1.00 39.26 C
ATOM 2093 O ALA A 184 6.653 10.599 -34.104 1.00 40.27 O
ATOM 2094 CB ALA A 184 5.006 11.209 -31.330 1.00 35.93 C
ATOM 2095 H ALA A 184 6.167 9.420 -29.974 1.00 40.21 H
ATOM 2096 HA ALA A 184 5.037 9.465 -32.596 1.00 38.93 H
ATOM 2097 HB1 ALA A 184 4.601 11.888 -32.082 1.00 35.93 H
ATOM 2098 HB2 ALA A 184 4.174 10.856 -30.728 1.00 35.93 H
ATOM 2099 HB3 ALA A 184 5.669 11.784 -30.683 1.00 35.93 H
ATOM 2100 N LYS A 185 8.014 10.778 -32.319 1.00 36.41 N
ATOM 2101 CA LYS A 185 9.151 11.164 -33.147 1.00 38.91 C
ATOM 2102 C LYS A 185 9.661 9.986 -33.966 1.00 44.38 C
ATOM 2103 O LYS A 185 10.055 10.155 -35.126 1.00 39.25 O
ATOM 2104 CB LYS A 185 10.265 11.745 -32.276 1.00 38.84 C
ATOM 2105 CG LYS A 185 9.939 13.122 -31.710 1.00 51.01 C
ATOM 2106 CD LYS A 185 10.919 13.538 -30.628 1.00 62.35 C
ATOM 2107 CE LYS A 185 10.579 14.918 -30.086 1.00 78.25 C
ATOM 2108 NZ LYS A 185 11.375 15.266 -28.875 1.00 87.36 N1+
ATOM 2109 H LYS A 185 8.152 10.700 -31.320 1.00 36.41 H
ATOM 2110 HA LYS A 185 8.834 11.943 -33.845 1.00 38.91 H
ATOM 2111 HB2 LYS A 185 10.488 11.048 -31.466 1.00 38.84 H
ATOM 2112 HB3 LYS A 185 11.185 11.834 -32.857 1.00 38.84 H
ATOM 2113 HG2 LYS A 185 9.908 13.862 -32.511 1.00 51.01 H
ATOM 2114 HG3 LYS A 185 8.945 13.059 -31.298 1.00 51.01 H
ATOM 2115 HD2 LYS A 185 10.893 12.808 -29.817 1.00 62.35 H

```
ATOM   2116  HD3 LYS A 185      11.936  13.531 -31.022  1.00 62.35           H
ATOM   2117  HE2 LYS A 185      10.741  15.677 -30.852  1.00 78.25           H
ATOM   2118  HE3 LYS A 185       9.539  14.978 -29.800  1.00 78.25           H
ATOM   2119  HZ1 LYS A 185      12.359  15.262 -29.104  1.00 87.36           H
ATOM   2120  HZ2 LYS A 185      11.110  16.185 -28.551  1.00 87.36           H
ATOM   2121  HZ3 LYS A 185      11.197  14.590 -28.147  1.00 87.36           H
ATOM   2122  N   ILE A 186       9.658   8.784 -33.387  1.00 38.40           N
ATOM   2123  CA  ILE A 186      10.059   7.603 -34.144  1.00 41.58           C
ATOM   2124  C   ILE A 186       9.111   7.373 -35.317  1.00 41.00           C
ATOM   2125  O   ILE A 186       9.546   7.066 -36.434  1.00 38.58           O
ATOM   2126  CB  ILE A 186      10.132   6.373 -33.220  1.00 40.47           C
ATOM   2127  CG1 ILE A 186      11.267   6.539 -32.207  1.00 44.84           C
ATOM   2128  CG2 ILE A 186      10.323   5.100 -34.032  1.00 36.96           C
ATOM   2129  CD1 ILE A 186      11.359   5.416 -31.191  1.00 41.00           C
ATOM   2130  H   ILE A 186       9.351   8.657 -32.432  1.00 38.40           H
ATOM   2131  HA  ILE A 186      11.055   7.781 -34.556  1.00 41.58           H
ATOM   2132  HB  ILE A 186       9.195   6.284 -32.671  1.00 40.47           H
ATOM   2133 HG12 ILE A 186      12.212   6.577 -32.751  1.00 44.84           H
ATOM   2134 HG13 ILE A 186      11.216   7.492 -31.691  1.00 44.84           H
ATOM   2135 HG21 ILE A 186      10.468   4.231 -33.391  1.00 36.96           H
ATOM   2136 HG22 ILE A 186       9.457   4.853 -34.646  1.00 36.96           H
ATOM   2137 HG23 ILE A 186      11.193   5.168 -34.686  1.00 36.96           H
ATOM   2138 HD11 ILE A 186      11.823   5.772 -30.272  1.00 41.00           H
ATOM   2139 HD12 ILE A 186      10.375   5.025 -30.930  1.00 41.00           H
ATOM   2140 HD13 ILE A 186      11.965   4.589 -31.563  1.00 41.00           H
ATOM   2141  N   ILE A 187       7.805   7.531 -35.087  1.00 36.61           N
ATOM   2142  CA  ILE A 187       6.828   7.309 -36.150  1.00 39.38           C
ATOM   2143  C   ILE A 187       7.016   8.323 -37.273  1.00 42.51           C
ATOM   2144  O   ILE A 187       6.954   7.973 -38.459  1.00 35.92           O
ATOM   2145  CB  ILE A 187       5.399   7.346 -35.578  1.00  0.00           C
ATOM   2146  CG1 ILE A 187       4.634   6.046 -35.899  1.00  0.00           C
ATOM   2147  CG2 ILE A 187       4.634   8.585 -36.078  1.00  0.00           C
ATOM   2148  CD1 ILE A 187       3.187   5.912 -35.398  1.00  0.00           C
ATOM   2149  H   ILE A 187       7.456   7.762 -34.164  1.00 36.61           H
ATOM   2150  HA  ILE A 187       7.020   6.320 -36.572  1.00 39.38           H
ATOM   2151  HB  ILE A 187       5.440   7.429 -34.494  1.00  0.00           H
ATOM   2152 HG12 ILE A 187       4.624   5.913 -36.982  1.00  0.00           H
ATOM   2153 HG13 ILE A 187       5.204   5.201 -35.511  1.00  0.00           H
ATOM   2154 HG21 ILE A 187       3.745   8.568 -35.480  1.00  0.00           H
ATOM   2155 HG22 ILE A 187       5.155   9.512 -35.926  1.00  0.00           H
```

ATOM	2156	HG23	ILE	A	187	4.328	8.511	-37.123	1.00	0.00	H
ATOM	2157	HD11	ILE	A	187	2.946	4.869	-35.206	1.00	0.00	H
ATOM	2158	HD12	ILE	A	187	3.014	6.491	-34.493	1.00	0.00	H
ATOM	2159	HD13	ILE	A	187	2.504	6.244	-36.174	1.00	0.00	H
ATOM	2160	N	ASN	A	188	7.253	9.591	-36.921	1.00	33.47	N
ATOM	2161	CA	ASN	A	188	7.520	10.608	-37.934	1.00	38.81	C
ATOM	2162	C	ASN	A	188	8.732	10.239	-38.784	1.00	38.65	C
ATOM	2163	O	ASN	A	188	8.740	10.471	-39.999	1.00	39.18	O
ATOM	2164	CB	ASN	A	188	7.723	11.973	-37.271	1.00	32.78	C
ATOM	2165	CG	ASN	A	188	6.423	12.582	-36.770	1.00	33.24	C
ATOM	2166	ND2	ASN	A	188	6.534	13.564	-35.881	1.00	37.05	N
ATOM	2167	OD1	ASN	A	188	5.334	12.185	-37.185	1.00	37.65	O
ATOM	2168	H	ASN	A	188	7.275	9.870	-35.949	1.00	33.47	H
ATOM	2169	HA	ASN	A	188	6.644	10.630	-38.586	1.00	38.81	H
ATOM	2170	HB2	ASN	A	188	8.454	11.912	-36.464	1.00	32.78	H
ATOM	2171	HB3	ASN	A	188	8.122	12.681	-38.000	1.00	32.78	H
ATOM	2172	HD21	ASN	A	188	7.443	13.873	-35.570	1.00	37.05	H
ATOM	2173	HD22	ASN	A	188	5.706	14.017	-35.520	1.00	37.05	H
ATOM	2174	N	VAL	A	189	9.762	9.655	-38.167	1.00	36.58	N
ATOM	2175	CA	VAL	A	189	10.945	9.251	-38.924	1.00	37.30	C
ATOM	2176	C	VAL	A	189	10.618	8.080	-39.842	1.00	37.57	C
ATOM	2177	O	VAL	A	189	11.006	8.067	-41.017	1.00	35.01	O
ATOM	2178	CB	VAL	A	189	12.106	8.912	-37.970	1.00	43.19	C
ATOM	2179	CG1	VAL	A	189	13.262	8.275	-38.735	1.00	39.82	C
ATOM	2180	CG2	VAL	A	189	12.579	10.160	-37.239	1.00	39.82	C
ATOM	2181	H	VAL	A	189	9.750	9.477	-37.171	1.00	36.58	H
ATOM	2182	HA	VAL	A	189	11.261	10.087	-39.553	1.00	37.30	H
ATOM	2183	HB	VAL	A	189	11.763	8.202	-37.217	1.00	43.19	H
ATOM	2184	HG11	VAL	A	189	14.147	8.201	-38.102	1.00	39.82	H
ATOM	2185	HG12	VAL	A	189	13.039	7.258	-39.059	1.00	39.82	H
ATOM	2186	HG13	VAL	A	189	13.540	8.861	-39.612	1.00	39.82	H
ATOM	2187	HG21	VAL	A	189	13.289	9.906	-36.451	1.00	39.82	H
ATOM	2188	HG22	VAL	A	189	13.081	10.845	-37.924	1.00	39.82	H
ATOM	2189	HG23	VAL	A	189	11.768	10.719	-36.781	1.00	39.82	H
ATOM	2190	N	CYS	A	190	9.907	7.075	-39.322	1.00	34.33	N
ATOM	2191	CA	CYS	A	190	9.513	5.938	-40.148	1.00	34.82	C
ATOM	2192	C	CYS	A	190	8.655	6.382	-41.327	1.00	40.20	C
ATOM	2193	O	CYS	A	190	8.840	5.906	-42.453	1.00	37.01	O
ATOM	2194	CB	CYS	A	190	8.768	4.907	-39.301	1.00	35.54	C
ATOM	2195	SG	CYS	A	190	9.794	4.069	-38.061	1.00	41.55	S

ATOM   2196 H   CYS A 190   9.608   7.089 -38.355 1.00 34.33   H
ATOM   2197 HA CYS A 190   10.415   5.469 -40.547 1.00 34.82   H
ATOM   2198 HB2 CYS A 190   7.922   5.368 -38.790 1.00 35.54   H
ATOM   2199 HB3 CYS A 190   8.351   4.129 -39.943 1.00 35.54   H
ATOM   2200 HG CYS A 190   9.969   5.141 -37.281 1.00 41.55   H
ATOM   2201 N   ASN A 191   7.707   7.291 -41.085 1.00 32.76   N
ATOM   2202 CA ASN A 191   6.882   7.808 -42.171 1.00 34.97   C
ATOM   2203 C   ASN A 191   7.715   8.577 -43.186 1.00 38.08   C
ATOM   2204 O   ASN A 191   7.430   8.528 -44.389 1.00 37.26   O
ATOM   2205 CB ASN A 191   5.774   8.701 -41.613 1.00 31.04   C
ATOM   2206 CG ASN A 191   4.679   7.908 -40.934 1.00 38.93   C
ATOM   2207 ND2 ASN A 191   3.895   8.577 -40.097 1.00 35.56   N
ATOM   2208 OD1 ASN A 191   4.532   6.709 -41.168 1.00 42.78   O
ATOM   2209 H   ASN A 191   7.544   7.649 -40.151 1.00 32.76   H
ATOM   2210 HA ASN A 191   6.448   6.945 -42.682 1.00 34.97   H
ATOM   2211 HB2 ASN A 191   6.183   9.454 -40.936 1.00 31.04   H
ATOM   2212 HB3 ASN A 191   5.290   9.244 -42.427 1.00 31.04   H
ATOM   2213 HD21 ASN A 191   4.048   9.560 -39.925 1.00 35.56   H
ATOM   2214 HD22 ASN A 191   3.143   8.105 -39.611 1.00 35.56   H
ATOM   2215 N   TRP A 192   8.742   9.291 -42.728 1.00 33.83   N
ATOM   2216 CA TRP A 192   9.609   9.999 -43.660 1.00 36.79   C
ATOM   2217 C   TRP A 192   10.428   9.023 -44.495 1.00 44.90   C
ATOM   2218 O   TRP A 192   10.631   9.242 -45.696 1.00 38.01   O
ATOM   2219 CB TRP A 192   10.519 10.962 -42.901 1.00 35.05   C
ATOM   2220 CG TRP A 192   11.408 11.747 -43.808 1.00 45.30   C
ATOM   2221 CD1 TRP A 192   11.106 12.918 -44.441 1.00 48.31   C
ATOM   2222 CD2 TRP A 192   12.744 11.413 -44.196 1.00 46.40   C
ATOM   2223 CE2 TRP A 192   13.194 12.430 -45.061 1.00 50.31   C
ATOM   2224 CE3 TRP A 192   13.606 10.353 -43.894 1.00 50.00   C
ATOM   2225 NE1 TRP A 192   12.175 13.336 -45.195 1.00 50.57   N
ATOM   2226 CZ2 TRP A 192   14.466 12.419 -45.629 1.00 50.26   C
ATOM   2227 CZ3 TRP A 192   14.868 10.344 -44.458 1.00 58.63   C
ATOM   2228 CH2 TRP A 192   15.286 11.371 -45.314 1.00 56.32   C
ATOM   2229 H   TRP A 192   8.953   9.348 -41.740 1.00 33.83   H
ATOM   2230 HA TRP A 192   8.990 10.593 -44.336 1.00 36.79   H
ATOM   2231 HB2 TRP A 192   9.916 11.665 -42.325 1.00 35.05   H
ATOM   2232 HB3 TRP A 192   11.138 10.430 -42.178 1.00 35.05   H
ATOM   2233 HD1 TRP A 192   10.157 13.428 -44.354 1.00 48.31   H
ATOM   2234 HE1 TRP A 192   12.180 14.178 -45.753 1.00 50.57   H
ATOM   2235 HE3 TRP A 192   13.293   9.558 -43.234 1.00 50.00   H

ATOM	2236	HZ2	TRP	A	192	14.791	13.205	-46.295	1.00	50.26	H
ATOM	2237	HZ3	TRP	A	192	15.543	9.530	-44.235	1.00	58.63	H
ATOM	2238	HH2	TRP	A	192	16.278	11.332	-45.740	1.00	56.32	H
ATOM	2239	N	ILE	A	193	10.906	7.939	-43.878	1.00	35.98	N
ATOM	2240	CA	ILE	A	193	11.613	6.905	-44.628	1.00	37.96	C
ATOM	2241	C	ILE	A	193	10.703	6.311	-45.693	1.00	42.04	C
ATOM	2242	O	ILE	A	193	11.120	6.086	-46.837	1.00	40.87	O
ATOM	2243	CB	ILE	A	193	12.148	5.823	-43.672	1.00	37.67	C
ATOM	2244	CG1	ILE	A	193	13.199	6.417	-42.731	1.00	38.83	C
ATOM	2245	CG2	ILE	A	193	12.716	4.644	-44.456	1.00	38.06	C
ATOM	2246	CD1	ILE	A	193	13.717	5.443	-41.690	1.00	45.19	C
ATOM	2247	H	ILE	A	193	10.758	7.790	-42.887	1.00	35.98	H
ATOM	2248	HA	ILE	A	193	12.462	7.371	-45.135	1.00	37.96	H
ATOM	2249	HB	ILE	A	193	11.327	5.451	-43.059	1.00	37.67	H
ATOM	2250	HG12	ILE	A	193	14.049	6.741	-43.333	1.00	38.83	H
ATOM	2251	HG13	ILE	A	193	12.866	7.323	-42.242	1.00	38.83	H
ATOM	2252	HG21	ILE	A	193	13.172	3.906	-43.798	1.00	38.06	H
ATOM	2253	HG22	ILE	A	193	11.955	4.095	-45.011	1.00	38.06	H
ATOM	2254	HG23	ILE	A	193	13.483	4.966	-45.162	1.00	38.06	H
ATOM	2255	HD11	ILE	A	193	14.122	5.978	-40.831	1.00	45.19	H
ATOM	2256	HD12	ILE	A	193	12.927	4.787	-41.324	1.00	45.19	H
ATOM	2257	HD13	ILE	A	193	14.518	4.820	-42.089	1.00	45.19	H
ATOM	2258	N	LEU	A	194	9.442	6.055	-45.336	1.00	38.37	N
ATOM	2259	CA	LEU	A	194	8.486	5.529	-46.303	1.00	45.61	C
ATOM	2260	C	LEU	A	194	8.228	6.532	-47.421	1.00	47.31	C
ATOM	2261	O	LEU	A	194	8.177	6.159	-48.599	1.00	47.61	O
ATOM	2262	CB	LEU	A	194	7.183	5.158	-45.595	1.00	38.86	C
ATOM	2263	CG	LEU	A	194	6.083	4.503	-46.429	1.00	46.77	C
ATOM	2264	CD1	LEU	A	194	6.568	3.183	-47.014	1.00	43.05	C
ATOM	2265	CD2	LEU	A	194	4.830	4.297	-45.588	1.00	48.90	C
ATOM	2266	H	LEU	A	194	9.121	6.218	-44.390	1.00	38.37	H
ATOM	2267	HA	LEU	A	194	8.914	4.627	-46.744	1.00	45.61	H
ATOM	2268	HB2	LEU	A	194	7.421	4.498	-44.758	1.00	38.86	H
ATOM	2269	HB3	LEU	A	194	6.774	6.059	-45.136	1.00	38.86	H
ATOM	2270	HG	LEU	A	194	5.811	5.163	-47.253	1.00	46.77	H
ATOM	2271	HD11	LEU	A	194	5.773	2.441	-47.091	1.00	43.05	H
ATOM	2272	HD12	LEU	A	194	6.951	3.334	-48.024	1.00	43.05	H
ATOM	2273	HD13	LEU	A	194	7.370	2.738	-46.424	1.00	43.05	H
ATOM	2274	HD21	LEU	A	194	3.947	4.661	-46.114	1.00	48.90	H
ATOM	2275	HD22	LEU	A	194	4.650	3.249	-45.346	1.00	48.90	H

ATOM 2276 HD23 LEU A 194 4.874 4.832 -44.638 1.00 48.90 H
ATOM 2277 N SER A 195 8.069 7.811 -47.070 1.00 40.15 N
ATOM 2278 CA SER A 195 7.892 8.842 -48.087 1.00 43.64 C
ATOM 2279 C SER A 195 9.107 8.942 -49.002 1.00 43.33 C
ATOM 2280 O SER A 195 8.975 9.329 -50.168 1.00 41.40 O
ATOM 2281 CB SER A 195 7.614 10.191 -47.422 1.00 36.06 C
ATOM 2282 OG SER A 195 6.424 10.147 -46.658 1.00 44.98 O
ATOM 2283 H SER A 195 8.092 8.097 -46.099 1.00 40.15 H
ATOM 2284 HA SER A 195 7.029 8.580 -48.703 1.00 43.64 H
ATOM 2285 HB2 SER A 195 8.439 10.490 -46.774 1.00 36.06 H
ATOM 2286 HB3 SER A 195 7.503 10.975 -48.173 1.00 36.06 H
ATOM 2287 HG SER A 195 6.589 9.622 -45.869 1.00 44.98 H
ATOM 2288 N SER A 196 10.292 8.592 -48.496 1.00 38.09 N
ATOM 2289 CA SER A 196 11.506 8.664 -49.300 1.00 41.59 C
ATOM 2290 C SER A 196 11.518 7.650 -50.438 1.00 42.19 C
ATOM 2291 O SER A 196 12.251 7.847 -51.413 1.00 46.33 O
ATOM 2292 CB SER A 196 12.734 8.461 -48.412 1.00 43.43 C
ATOM 2293 OG SER A 196 12.835 9.486 -47.439 1.00 45.74 O
ATOM 2294 H SER A 196 10.378 8.280 -47.538 1.00 38.09 H
ATOM 2295 HA SER A 196 11.565 9.653 -49.757 1.00 41.59 H
ATOM 2296 HB2 SER A 196 12.746 7.491 -47.925 1.00 43.43 H
ATOM 2297 HB3 SER A 196 13.638 8.497 -49.017 1.00 43.43 H
ATOM 2298 HG SER A 196 12.127 9.370 -46.794 1.00 45.74 H
ATOM 2299 N ALA A 197 10.731 6.572 -50.337 1.00 42.37 N
ATOM 2300 CA ALA A 197 10.681 5.576 -51.404 1.00 47.80 C
ATOM 2301 C ALA A 197 10.208 6.171 -52.722 1.00 49.56 C
ATOM 2302 O ALA A 197 10.492 5.609 -53.784 1.00 50.31 O
ATOM 2303 CB ALA A 197 9.772 4.412 -51.005 1.00 47.45 C
ATOM 2304 H ALA A 197 10.135 6.430 -49.532 1.00 42.37 H
ATOM 2305 HA ALA A 197 11.690 5.185 -51.553 1.00 47.80 H
ATOM 2306 HB1 ALA A 197 9.783 3.623 -51.758 1.00 47.45 H
ATOM 2307 HB2 ALA A 197 10.096 3.968 -50.063 1.00 47.45 H
ATOM 2308 HB3 ALA A 197 8.738 4.735 -50.879 1.00 47.45 H
ATOM 2309 N ILE A 198 9.494 7.292 -52.676 1.00 42.68 N
ATOM 2310 CA ILE A 198 9.100 8.026 -53.865 1.00 48.64 C
ATOM 2311 C ILE A 198 9.753 9.403 -53.914 1.00 52.39 C
ATOM 2312 O ILE A 198 10.140 9.870 -54.987 1.00 47.98 O
ATOM 2313 CB ILE A 198 7.543 8.144 -53.998 1.00 50.56 C
ATOM 2314 CG1 ILE A 198 6.929 6.743 -54.261 1.00 54.38 C
ATOM 2315 CG2 ILE A 198 7.058 9.133 -55.089 1.00 48.54 C

```
ATOM   2316  CD1 ILE A 198       5.413   6.645 -54.033  1.00 56.06           C
ATOM   2317  H   ILE A 198       9.264   7.706 -51.784  1.00 42.68           H
ATOM   2318  HA  ILE A 198       9.437   7.522 -54.773  1.00 48.64           H
ATOM   2319  HB  ILE A 198       7.154   8.499 -53.042  1.00 50.56           H
ATOM   2320 HG12 ILE A 198       7.163   6.428 -55.279  1.00 54.38           H
ATOM   2321 HG13 ILE A 198       7.397   6.000 -53.616  1.00 54.38           H
ATOM   2322 HG21 ILE A 198       5.976   9.115 -55.212  1.00 48.54           H
ATOM   2323 HG22 ILE A 198       7.318  10.166 -54.860  1.00 48.54           H
ATOM   2324 HG23 ILE A 198       7.493   8.893 -56.060  1.00 48.54           H
ATOM   2325 HD11 ILE A 198       5.083   5.607 -54.086  1.00 56.06           H
ATOM   2326 HD12 ILE A 198       5.142   7.019 -53.046  1.00 56.06           H
ATOM   2327 HD13 ILE A 198       4.848   7.200 -54.781  1.00 56.06           H
ATOM   2328  N   GLY A 199       9.923  10.051 -52.755  1.00 44.98           N
ATOM   2329  CA  GLY A 199      10.474  11.397 -52.744  1.00 43.62           C
ATOM   2330  C   GLY A 199      11.904  11.476 -53.244  1.00 47.58           C
ATOM   2331  O   GLY A 199      12.292  12.467 -53.866  1.00 53.22           O
ATOM   2332  H   GLY A 199       9.606   9.657 -51.878  1.00 44.98           H
ATOM   2333  HA2 GLY A 199       9.836  12.068 -53.323  1.00 43.62           H
ATOM   2334  HA3 GLY A 199      10.440  11.750 -51.716  1.00 43.62           H
ATOM   2335  N   LEU A 200      12.712  10.452 -52.968  1.00 46.67           N
ATOM   2336  CA  LEU A 200      14.099  10.437 -53.423  1.00 52.06           C
ATOM   2337  C   LEU A 200      14.217  10.048 -54.896  1.00 51.35           C
ATOM   2338  O   LEU A 200      14.953  10.714 -55.637  1.00 42.98           O
ATOM   2339  CB  LEU A 200      14.943   9.496 -52.557  1.00 57.90           C
ATOM   2340  CG  LEU A 200      15.347  10.002 -51.175  1.00 62.45           C
ATOM   2341  CD1 LEU A 200      16.181   8.959 -50.451  1.00 66.58           C
ATOM   2342  CD2 LEU A 200      16.107  11.312 -51.293  1.00 66.77           C
ATOM   2343  H   LEU A 200      12.381   9.647 -52.452  1.00 46.67           H
ATOM   2344  HA  LEU A 200      14.511  11.444 -53.343  1.00 52.06           H
ATOM   2345  HB2 LEU A 200      14.454   8.544 -52.405  1.00 57.90           H
ATOM   2346  HB3 LEU A 200      15.865   9.258 -53.092  1.00 57.90           H
ATOM   2347  HG  LEU A 200      14.426  10.135 -50.626  1.00 62.45           H
ATOM   2348 HD11 LEU A 200      15.891   8.892 -49.403  1.00 66.58           H
ATOM   2349 HD12 LEU A 200      16.066   7.962 -50.879  1.00 66.58           H
ATOM   2350 HD13 LEU A 200      17.247   9.187 -50.474  1.00 66.58           H
ATOM   2351 HD21 LEU A 200      16.880  11.412 -50.531  1.00 66.77           H
ATOM   2352 HD22 LEU A 200      16.595  11.429 -52.261  1.00 66.77           H
ATOM   2353 HD23 LEU A 200      15.432  12.158 -51.160  1.00 66.77           H
ATOM   2354  N   PRO A 201      13.545   8.983 -55.367  1.00 42.72           N
ATOM   2355  CA  PRO A 201      13.636   8.668 -56.805  1.00 45.63           C
```

ATOM 2356 C PRO A 201 13.171 9.792 -57.717 1.00 49.30 C
ATOM 2357 O PRO A 201 13.757 9.980 -58.791 1.00 53.76 O
ATOM 2358 CB PRO A 201 12.751 7.423 -56.945 1.00 43.87 C
ATOM 2359 CG PRO A 201 12.821 6.777 -55.620 1.00 46.80 C
ATOM 2360 CD PRO A 201 12.840 7.910 -54.636 1.00 42.90 C
ATOM 2361 HA PRO A 201 14.671 8.407 -57.036 1.00 45.63 H
ATOM 2362 HB2 PRO A 201 11.715 7.698 -57.155 1.00 43.87 H
ATOM 2363 HB3 PRO A 201 13.083 6.762 -57.746 1.00 43.87 H
ATOM 2364 HG2 PRO A 201 12.030 6.049 -55.491 1.00 46.80 H
ATOM 2365 HG3 PRO A 201 13.764 6.233 -55.548 1.00 46.80 H
ATOM 2366 HD2 PRO A 201 11.844 8.186 -54.372 1.00 42.90 H
ATOM 2367 HD3 PRO A 201 13.337 7.552 -53.745 1.00 42.90 H
ATOM 2368 N VAL A 202 12.136 10.547 -57.334 1.00 41.26 N
ATOM 2369 CA VAL A 202 11.673 11.613 -58.220 1.00 46.30 C
ATOM 2370 C VAL A 202 12.703 12.729 -58.304 1.00 50.30 C
ATOM 2371 O VAL A 202 12.744 13.466 -59.294 1.00 51.45 O
ATOM 2372 CB VAL A 202 10.297 12.157 -57.787 1.00 40.72 C
ATOM 2373 CG1 VAL A 202 9.244 11.057 -57.841 1.00 36.87 C
ATOM 2374 CG2 VAL A 202 10.368 12.796 -56.410 1.00 44.68 C
ATOM 2375 H VAL A 202 11.635 10.385 -56.470 1.00 41.26 H
ATOM 2376 HA VAL A 202 11.540 11.201 -59.217 1.00 46.30 H
ATOM 2377 HB VAL A 202 9.988 12.935 -58.486 1.00 40.72 H
ATOM 2378 HG11 VAL A 202 8.300 11.391 -57.410 1.00 36.87 H
ATOM 2379 HG12 VAL A 202 9.039 10.780 -58.875 1.00 36.87 H
ATOM 2380 HG13 VAL A 202 9.538 10.144 -57.331 1.00 36.87 H
ATOM 2381 HG21 VAL A 202 9.386 13.147 -56.097 1.00 44.68 H
ATOM 2382 HG22 VAL A 202 10.710 12.094 -55.669 1.00 44.68 H
ATOM 2383 HG23 VAL A 202 11.030 13.660 -56.389 1.00 44.68 H
ATOM 2384 N MET A 203 13.552 12.875 -57.284 1.00 50.01 N
ATOM 2385 CA MET A 203 14.636 13.849 -57.369 1.00 56.63 C
ATOM 2386 C MET A 203 15.615 13.485 -58.477 1.00 53.71 C
ATOM 2387 O MET A 203 16.162 14.369 -59.148 1.00 52.82 O
ATOM 2388 CB MET A 203 15.351 13.956 -56.024 1.00 64.51 C
ATOM 2389 CG MET A 203 16.660 14.719 -56.066 1.00 79.41 C
ATOM 2390 SD MET A 203 16.906 15.735 -54.602 1.00 93.94 S
ATOM 2391 CE MET A 203 15.716 17.039 -54.903 1.00 88.64 C
ATOM 2392 H MET A 203 13.518 12.268 -56.476 1.00 50.01 H
ATOM 2393 HA MET A 203 14.216 14.830 -57.602 1.00 56.63 H
ATOM 2394 HB2 MET A 203 14.668 14.372 -55.285 1.00 64.51 H
ATOM 2395 HB3 MET A 203 15.598 12.971 -55.647 1.00 64.51 H

ATOM 2396 HG2 MET A 203 17.502 14.034 -56.175 1.00 79.41 H
ATOM 2397 HG3 MET A 203 16.703 15.416 -56.902 1.00 79.41 H
ATOM 2398 HE1 MET A 203 15.677 17.722 -54.055 1.00 88.64 H
ATOM 2399 HE2 MET A 203 15.995 17.609 -55.789 1.00 88.64 H
ATOM 2400 HE3 MET A 203 14.727 16.631 -55.065 1.00 88.64 H
ATOM 2401 N PHE A 204 15.836 12.187 -58.698 1.00 50.90 N
ATOM 2402 CA PHE A 204 16.662 11.753 -59.819 1.00 49.90 C
ATOM 2403 C PHE A 204 15.880 11.759 -61.125 1.00 49.62 C
ATOM 2404 O PHE A 204 16.419 12.136 -62.172 1.00 52.96 O
ATOM 2405 CB PHE A 204 17.229 10.359 -59.547 1.00 58.38 C
ATOM 2406 CG PHE A 204 18.332 10.342 -58.530 1.00 61.45 C
ATOM 2407 CD1 PHE A 204 19.652 10.472 -58.924 1.00 66.61 C
ATOM 2408 CD2 PHE A 204 18.051 10.197 -57.181 1.00 63.92 C
ATOM 2409 CE1 PHE A 204 20.673 10.460 -57.994 1.00 72.05 C
ATOM 2410 CE2 PHE A 204 19.069 10.183 -56.244 1.00 64.58 C
ATOM 2411 CZ PHE A 204 20.382 10.315 -56.653 1.00 66.27 C
ATOM 2412 H PHE A 204 15.378 11.476 -58.144 1.00 50.90 H
ATOM 2413 HA PHE A 204 17.512 12.426 -59.948 1.00 49.90 H
ATOM 2414 HB2 PHE A 204 16.437 9.684 -59.218 1.00 58.38 H
ATOM 2415 HB3 PHE A 204 17.618 9.923 -60.469 1.00 58.38 H
ATOM 2416 HD1 PHE A 204 19.893 10.588 -59.971 1.00 66.61 H
ATOM 2417 HD2 PHE A 204 17.030 10.084 -56.856 1.00 63.92 H
ATOM 2418 HE1 PHE A 204 21.699 10.563 -58.316 1.00 72.05 H
ATOM 2419 HE2 PHE A 204 18.836 10.068 -55.195 1.00 64.58 H
ATOM 2420 HZ PHE A 204 21.179 10.305 -55.924 1.00 66.27 H
ATOM 2421 N MET A 205 14.609 11.351 -61.084 1.00 41.16 N
ATOM 2422 CA MET A 205 13.825 11.236 -62.310 1.00 48.71 C
ATOM 2423 C MET A 205 13.426 12.595 -62.871 1.00 49.31 C
ATOM 2424 O MET A 205 13.302 12.745 -64.092 1.00 50.17 O
ATOM 2425 CB MET A 205 12.550 10.415 -62.048 1.00 44.99 C
ATOM 2426 CG MET A 205 12.786 8.941 -61.680 1.00 58.86 C
ATOM 2427 SD MET A 205 11.497 8.210 -60.630 1.00 63.02 S
ATOM 2428 CE MET A 205 10.013 8.747 -61.513 1.00 68.95 C
ATOM 2429 H MET A 205 14.189 11.026 -60.221 1.00 41.16 H
ATOM 2430 HA MET A 205 14.417 10.726 -63.074 1.00 48.71 H
ATOM 2431 HB2 MET A 205 11.954 10.906 -61.284 1.00 44.99 H
ATOM 2432 HB3 MET A 205 11.932 10.434 -62.948 1.00 44.99 H
ATOM 2433 HG2 MET A 205 12.868 8.346 -62.590 1.00 58.86 H
ATOM 2434 HG3 MET A 205 13.736 8.815 -61.161 1.00 58.86 H
ATOM 2435 HE1 MET A 205 9.158 8.168 -61.171 1.00 68.95 H

```
ATOM   2436  HE2 MET A 205       9.806   9.802 -61.332  1.00 68.95           H
ATOM   2437  HE3 MET A 205      10.137   8.597 -62.585  1.00 68.95           H
ATOM   2438  N   ALA A 206      13.203  13.583 -62.009  1.00 39.97           N
ATOM   2439  CA  ALA A 206      12.709  14.871 -62.474  1.00 46.95           C
ATOM   2440  C   ALA A 206      13.765  15.586 -63.303  1.00 51.63           C
ATOM   2441  O   ALA A 206      14.952  15.582 -62.969  1.00 55.63           O
ATOM   2442  CB  ALA A 206      12.291  15.750 -61.296  1.00 37.78           C
ATOM   2443  H   ALA A 206      13.282  13.444 -61.009  1.00 39.97           H
ATOM   2444  HA  ALA A 206      11.823  14.674 -63.063  1.00 46.95           H
ATOM   2445  HB1 ALA A 206      12.035  16.762 -61.612  1.00 37.78           H
ATOM   2446  HB2 ALA A 206      11.426  15.334 -60.784  1.00 37.78           H
ATOM   2447  HB3 ALA A 206      13.095  15.821 -60.565  1.00 37.78           H
ATOM   2448  N   THR A 207      13.322  16.197 -64.396  1.00 53.48           N
ATOM   2449  CA  THR A 207      14.208  16.977 -65.245  1.00 54.00           C
ATOM   2450  C   THR A 207      13.359  17.872 -66.130  1.00 52.23           C
ATOM   2451  O   THR A 207      12.152  17.666 -66.279  1.00 55.37           O
ATOM   2452  CB  THR A 207      15.115  16.081 -66.099  1.00 60.06           C
ATOM   2453  CG2 THR A 207      14.308  15.373 -67.175  1.00 51.07           C
ATOM   2454  OG1 THR A 207      16.126  16.880 -66.724  1.00 69.31           O
ATOM   2455  H   THR A 207      12.342  16.168 -64.653  1.00 53.48           H
ATOM   2456  HA  THR A 207      14.819  17.627 -64.613  1.00 54.00           H
ATOM   2457  HB  THR A 207      15.618  15.337 -65.481  1.00 60.06           H
ATOM   2458  HG1 THR A 207      16.588  16.339 -67.370  1.00 69.31           H
ATOM   2459 HG21 THR A 207      14.918  14.606 -67.653  1.00 51.07           H
ATOM   2460 HG22 THR A 207      13.438  14.869 -66.754  1.00 51.07           H
ATOM   2461 HG23 THR A 207      13.966  16.035 -67.972  1.00 51.07           H
ATOM   2462  N   THR A 208      14.006  18.878 -66.704  1.00 60.05           N
ATOM   2463  CA  THR A 208      13.395  19.723 -67.718  1.00 61.26           C
ATOM   2464  C   THR A 208      13.792  19.207 -69.094  1.00 63.88           C
ATOM   2465  O   THR A 208      14.971  18.932 -69.343  1.00 78.15           O
ATOM   2466  CB  THR A 208      13.828  21.180 -67.559  1.00 61.63           C
ATOM   2467  CG2 THR A 208      13.307  21.756 -66.247  1.00 54.71           C
ATOM   2468  OG1 THR A 208      15.260  21.255 -67.580  1.00 58.74           O
ATOM   2469  H   THR A 208      14.999  18.996 -66.559  1.00 60.05           H
ATOM   2470  HA  THR A 208      12.310  19.703 -67.632  1.00 61.26           H
ATOM   2471  HB  THR A 208      13.447  21.780 -68.386  1.00 61.63           H
ATOM   2472  HG1 THR A 208      15.572  20.880 -68.409  1.00 58.74           H
ATOM   2473 HG21 THR A 208      13.634  22.788 -66.120  1.00 54.71           H
ATOM   2474 HG22 THR A 208      12.217  21.756 -66.227  1.00 54.71           H
ATOM   2475 HG23 THR A 208      13.646  21.188 -65.383  1.00 54.71           H
```

ATOM 2476 N LYS A 209 12.807 19.062 -69.975 1.00 61.86 N
ATOM 2477 CA LYS A 209 13.060 18.699 -71.360 1.00 74.00 C
ATOM 2478 C LYS A 209 12.163 19.536 -72.258 1.00 80.79 C
ATOM 2479 O LYS A 209 11.102 20.007 -71.838 1.00 76.57 O
ATOM 2480 CB LYS A 209 12.824 17.204 -71.615 1.00 76.15 C
ATOM 2481 CG LYS A 209 11.414 16.735 -71.312 1.00 78.90 C
ATOM 2482 CD LYS A 209 11.107 15.431 -72.025 1.00 80.46 C
ATOM 2483 CE LYS A 209 11.183 15.610 -73.532 1.00 92.30 C
ATOM 2484 NZ LYS A 209 10.842 14.360 -74.266 1.00 96.81 N1+
ATOM 2485 H LYS A 209 11.851 19.282 -69.727 1.00 61.86 H
ATOM 2486 HA LYS A 209 14.089 18.939 -71.639 1.00 74.00 H
ATOM 2487 HB2 LYS A 209 13.149 17.040 -72.635 1.00 76.15 H
ATOM 2488 HB3 LYS A 209 13.520 16.621 -71.010 1.00 76.15 H
ATOM 2489 HG2 LYS A 209 11.312 16.593 -70.236 1.00 78.90 H
ATOM 2490 HG3 LYS A 209 10.650 17.461 -71.587 1.00 78.90 H
ATOM 2491 HD2 LYS A 209 11.804 14.657 -71.699 1.00 80.46 H
ATOM 2492 HD3 LYS A 209 10.111 15.091 -71.739 1.00 80.46 H
ATOM 2493 HE2 LYS A 209 10.495 16.393 -73.851 1.00 92.30 H
ATOM 2494 HE3 LYS A 209 12.168 15.867 -73.909 1.00 92.30 H
ATOM 2495 HZ1 LYS A 209 9.908 14.068 -74.020 1.00 96.81 H
ATOM 2496 HZ2 LYS A 209 10.893 14.530 -75.260 1.00 96.81 H
ATOM 2497 HZ3 LYS A 209 11.499 13.634 -74.017 1.00 96.81 H
ATOM 2498 N TYR A 210 12.600 19.719 -73.500 1.00 83.61 N
ATOM 2499 CA TYR A 210 11.867 20.557 -74.436 1.00 91.20 C
ATOM 2500 C TYR A 210 10.630 19.841 -74.959 1.00 92.18 C
ATOM 2501 O TYR A 210 10.657 18.639 -75.240 1.00 92.79 O
ATOM 2502 CB TYR A 210 12.765 20.977 -75.598 1.00 96.10 C
ATOM 2503 CG TYR A 210 13.582 22.209 -75.294 1.00106.77 C
ATOM 2504 CD1 TYR A 210 12.993 23.467 -75.287 1.00114.30 C
ATOM 2505 CD2 TYR A 210 14.938 22.118 -75.009 1.00109.50 C
ATOM 2506 CE1 TYR A 210 13.730 24.599 -75.006 1.00120.46 C
ATOM 2507 CE2 TYR A 210 15.685 23.247 -74.727 1.00115.42 C
ATOM 2508 CZ TYR A 210 15.075 24.485 -74.727 1.00122.00 C
ATOM 2509 OH TYR A 210 15.812 25.613 -74.448 1.00128.00 O
ATOM 2510 H TYR A 210 13.470 19.317 -73.815 1.00 83.61 H
ATOM 2511 HA TYR A 210 11.542 21.460 -73.912 1.00 91.20 H
ATOM 2512 HB2 TYR A 210 13.401 20.153 -75.928 1.00 96.10 H
ATOM 2513 HB3 TYR A 210 12.158 21.241 -76.466 1.00 96.10 H
ATOM 2514 HD1 TYR A 210 11.938 23.566 -75.500 1.00114.30 H
ATOM 2515 HD2 TYR A 210 15.423 21.153 -75.004 1.00109.50 H

ATOM 2516 HE1 TYR A 210 13.247 25.565 -75.004 1.00120.46 H
ATOM 2517 HE2 TYR A 210 16.739 23.158 -74.508 1.00115.42 H
ATOM 2518 HH TYR A 210 15.293 26.420 -74.474 1.00128.00 H
ATOM 2519 N ARG A 211 9.539 20.597 -75.087 1.00 90.65 N
ATOM 2520 CA ARG A 211 8.251 20.057 -75.525 1.00 95.11 C
ATOM 2521 C ARG A 211 7.568 21.141 -76.362 1.00104.28 C
ATOM 2522 O ARG A 211 6.895 22.023 -75.824 1.00109.68 O
ATOM 2523 CB ARG A 211 7.397 19.636 -74.338 1.00101.43 C
ATOM 2524 CG ARG A 211 6.046 19.060 -74.718 1.00107.03 C
ATOM 2525 CD ARG A 211 6.198 17.725 -75.419 1.00113.27 C
ATOM 2526 NE ARG A 211 4.974 17.335 -76.111 1.00120.37 N
ATOM 2527 CZ ARG A 211 4.824 16.193 -76.772 1.00120.52 C
ATOM 2528 NH1 ARG A 211 5.821 15.321 -76.826 1.00117.65 N1+
ATOM 2529 NH2 ARG A 211 3.676 15.920 -77.376 1.00122.28 N1+
ATOM 2530 H ARG A 211 9.549 21.575 -74.817 1.00 90.65 H
ATOM 2531 HA ARG A 211 8.436 19.213 -76.188 1.00 95.11 H
ATOM 2532 HB2 ARG A 211 7.943 18.899 -73.746 1.00101.43 H
ATOM 2533 HB3 ARG A 211 7.252 20.489 -73.673 1.00101.43 H
ATOM 2534 HG2 ARG A 211 5.528 18.864 -73.779 1.00107.03 H
ATOM 2535 HG3 ARG A 211 5.408 19.753 -75.269 1.00107.03 H
ATOM 2536 HD2 ARG A 211 7.072 17.637 -76.059 1.00113.27 H
ATOM 2537 HD3 ARG A 211 6.312 16.964 -74.646 1.00113.27 H
ATOM 2538 HE ARG A 211 4.223 18.013 -76.123 1.00120.37 H
ATOM 2539 HH11 ARG A 211 6.704 15.537 -76.388 1.00117.65 H
ATOM 2540 HH12 ARG A 211 5.713 14.452 -77.330 1.00117.65 H
ATOM 2541 HH21 ARG A 211 2.918 16.589 -77.350 1.00122.28 H
ATOM 2542 HH22 ARG A 211 3.561 15.064 -77.903 1.00122.28 H
ATOM 2543 N GLN A 212 7.759 21.061 -77.682 1.00100.09 N
ATOM 2544 CA GLN A 212 7.173 22.008 -78.633 1.00100.97 C
ATOM 2545 C GLN A 212 7.605 23.441 -78.321 1.00106.86 C
ATOM 2546 O GLN A 212 6.787 24.360 -78.237 1.00103.67 O
ATOM 2547 CB GLN A 212 5.647 21.891 -78.659 1.00101.41 C
ATOM 2548 CG GLN A 212 5.132 20.468 -78.789 1.00103.31 C
ATOM 2549 CD GLN A 212 3.618 20.401 -78.829 1.00105.89 C
ATOM 2550 NE2 GLN A 212 2.979 21.033 -79.669 1.00102.10 N
ATOM 2551 OE1 GLN A 212 3.035 19.636 -77.912 1.00111.06 O
ATOM 2552 H GLN A 212 8.327 20.322 -78.070 1.00100.09 H
ATOM 2553 HA GLN A 212 7.560 21.755 -79.621 1.00100.97 H
ATOM 2554 HB2 GLN A 212 5.211 22.329 -77.759 1.00101.41 H
ATOM 2555 HB3 GLN A 212 5.272 22.493 -79.489 1.00101.41 H

ATOM 2556 HG2 GLN A 212 5.524 20.010 -79.697 1.00103.31 H
ATOM 2557 HG3 GLN A 212 5.472 19.849 -77.959 1.00103.31 H
ATOM 2558 HE21 GLN A 212 3.452 21.610 -80.349 1.00102.10 H
ATOM 2559 HE22 GLN A 212 1.970 20.984 -79.677 1.00102.10 H
ATOM 2560 N GLY A 213 8.906 23.627 -78.149 1.00112.07 N
ATOM 2561 CA GLY A 213 9.430 24.938 -77.765 1.00119.50 C
ATOM 2562 C GLY A 213 9.297 25.261 -76.294 1.00120.52 C
ATOM 2563 O GLY A 213 10.246 25.756 -75.679 1.00126.75 O
ATOM 2564 H GLY A 213 9.553 22.856 -78.224 1.00112.07 H
ATOM 2565 HA2 GLY A 213 10.489 24.953 -78.026 1.00119.50 H
ATOM 2566 HA3 GLY A 213 8.969 25.735 -78.351 1.00119.50 H
ATOM 2567 N SER A 214 8.131 24.992 -75.709 1.00113.95 N
ATOM 2568 CA SER A 214 7.926 25.199 -74.285 1.00101.89 C
ATOM 2569 C SER A 214 8.679 24.139 -73.481 1.00 93.27 C
ATOM 2570 O SER A 214 9.149 23.129 -74.011 1.00 91.08 O
ATOM 2571 CB SER A 214 6.435 25.169 -73.952 1.00103.78 C
ATOM 2572 OG SER A 214 5.847 23.950 -74.365 1.00110.89 O
ATOM 2573 H SER A 214 7.370 24.602 -76.247 1.00113.95 H
ATOM 2574 HA SER A 214 8.311 26.181 -74.001 1.00101.89 H
ATOM 2575 HB2 SER A 214 6.265 25.255 -72.880 1.00103.78 H
ATOM 2576 HB3 SER A 214 5.914 25.999 -74.430 1.00103.78 H
ATOM 2577 HG SER A 214 6.066 23.776 -75.284 1.00110.89 H
ATOM 2578 N ILE A 215 8.788 24.378 -72.177 1.00 86.52 N
ATOM 2579 CA ILE A 215 9.570 23.536 -71.280 1.00 74.91 C
ATOM 2580 C ILE A 215 8.628 22.844 -70.305 1.00 73.44 C
ATOM 2581 O ILE A 215 7.796 23.495 -69.659 1.00 68.89 O
ATOM 2582 CB ILE A 215 10.636 24.349 -70.527 1.00 74.06 C
ATOM 2583 CG1 ILE A 215 11.606 24.994 -71.518 1.00 74.93 C
ATOM 2584 CG2 ILE A 215 11.377 23.467 -69.533 1.00 70.75 C
ATOM 2585 CD1 ILE A 215 12.652 25.870 -70.865 1.00 73.96 C
ATOM 2586 H ILE A 215 8.360 25.198 -71.773 1.00 86.52 H
ATOM 2587 HA ILE A 215 10.101 22.758 -71.834 1.00 74.91 H
ATOM 2588 HB ILE A 215 10.182 25.127 -69.924 1.00 74.06 H
ATOM 2589 HG12 ILE A 215 12.104 24.218 -72.101 1.00 74.93 H
ATOM 2590 HG13 ILE A 215 11.068 25.608 -72.241 1.00 74.93 H
ATOM 2591 HG21 ILE A 215 12.158 24.019 -69.011 1.00 70.75 H
ATOM 2592 HG22 ILE A 215 10.740 23.064 -68.747 1.00 70.75 H
ATOM 2593 HG23 ILE A 215 11.853 22.624 -70.035 1.00 70.75 H
ATOM 2594 HD11 ILE A 215 13.024 26.615 -71.569 1.00 73.96 H
ATOM 2595 HD12 ILE A 215 12.252 26.406 -70.004 1.00 73.96 H

ATOM	2596	HD13	ILE	A	215	13.508	25.283	-70.531	1.00	73.96	H
ATOM	2597	N	ASP	A	216	8.767	21.528	-70.196	1.00	63.22	N
ATOM	2598	CA	ASP	A	216	7.994	20.717	-69.267	1.00	60.23	C
ATOM	2599	C	ASP	A	216	8.923	20.226	-68.166	1.00	67.54	C
ATOM	2600	O	ASP	A	216	9.946	19.593	-68.451	1.00	71.64	O
ATOM	2601	CB	ASP	A	216	7.354	19.506	-70.005	1.00	67.22	C
ATOM	2602	CG	ASP	A	216	6.343	18.668	-69.203	1.00	79.44	C
ATOM	2603	OD1	ASP	A	216	6.007	19.043	-68.060	1.00	87.02	O
ATOM	2604	OD2	ASP	A	216	5.867	17.663	-69.770	1.00	86.62	O1-
ATOM	2605	H	ASP	A	216	9.467	21.042	-70.741	1.00	63.22	H
ATOM	2606	HA	ASP	A	216	7.203	21.314	-68.808	1.00	60.23	H
ATOM	2607	HB2	ASP	A	216	6.845	19.876	-70.891	1.00	67.22	H
ATOM	2608	HB3	ASP	A	216	8.146	18.834	-70.341	1.00	67.22	H
ATOM	2609	N	CYS	A	217	8.579	20.534	-66.917	1.00	67.20	N
ATOM	2610	CA	CYS	A	217	9.239	19.928	-65.763	1.00	53.84	C
ATOM	2611	C	CYS	A	217	8.536	18.603	-65.500	1.00	58.60	C
ATOM	2612	O	CYS	A	217	7.434	18.567	-64.949	1.00	64.11	O
ATOM	2613	CB	CYS	A	217	9.184	20.906	-64.574	1.00	53.94	C
ATOM	2614	SG	CYS	A	217	9.689	20.262	-62.959	1.00	63.09	S
ATOM	2615	H	CYS	A	217	7.734	21.058	-66.737	1.00	67.20	H
ATOM	2616	HA	CYS	A	217	10.293	19.740	-65.977	1.00	53.84	H
ATOM	2617	HB2	CYS	A	217	9.839	21.743	-64.795	1.00	53.94	H
ATOM	2618	HB3	CYS	A	217	8.180	21.313	-64.456	1.00	53.94	H
ATOM	2619	N	THR	A	218	9.162	17.507	-65.919	1.00	59.73	N
ATOM	2620	CA	THR	A	218	8.511	16.207	-65.947	1.00	58.65	C
ATOM	2621	C	THR	A	218	9.435	15.157	-65.340	1.00	53.89	C
ATOM	2622	O	THR	A	218	10.508	15.466	-64.813	1.00	56.41	O
ATOM	2623	CB	THR	A	218	8.057	15.871	-67.408	1.00	60.96	C
ATOM	2624	CG2	THR	A	218	9.206	15.802	-68.428	1.00	52.90	C
ATOM	2625	OG1	THR	A	218	7.319	14.662	-67.528	1.00	66.56	O
ATOM	2626	H	THR	A	218	10.090	17.562	-66.317	1.00	59.73	H
ATOM	2627	HA	THR	A	218	7.619	16.192	-65.320	1.00	58.65	H
ATOM	2628	HB	THR	A	218	7.376	16.660	-67.715	1.00	60.96	H
ATOM	2629	HG1	THR	A	218	6.939	14.601	-68.417	1.00	66.56	H
ATOM	2630	HG21	THR	A	218	8.827	15.631	-69.436	1.00	52.90	H
ATOM	2631	HG22	THR	A	218	9.763	16.739	-68.450	1.00	52.90	H
ATOM	2632	HG23	THR	A	218	9.909	15.002	-68.196	1.00	52.90	H
ATOM	2633	N	LEU	A	219	9.000	13.904	-65.412	1.00	48.31	N
ATOM	2634	CA	LEU	A	219	9.744	12.764	-64.896	1.00	49.86	C
ATOM	2635	C	LEU	A	219	10.194	11.880	-66.051	1.00	53.06	C

ATOM 2636 O LEU A 219 9.433 11.641 -66.994 1.00 52.12 O
ATOM 2637 CB LEU A 219 8.842 11.951 -63.933 1.00 46.61 C
ATOM 2638 CG LEU A 219 8.382 12.701 -62.660 1.00 50.60 C
ATOM 2639 CD1 LEU A 219 7.366 11.868 -61.849 1.00 47.08 C
ATOM 2640 CD2 LEU A 219 9.564 13.169 -61.792 1.00 50.44 C
ATOM 2641 H LEU A 219 8.134 13.710 -65.900 1.00 48.31 H
ATOM 2642 HA LEU A 219 10.636 13.086 -64.360 1.00 49.86 H
ATOM 2643 HB2 LEU A 219 7.959 11.622 -64.484 1.00 46.61 H
ATOM 2644 HB3 LEU A 219 9.362 11.037 -63.639 1.00 46.61 H
ATOM 2645 HG LEU A 219 7.849 13.597 -62.984 1.00 50.60 H
ATOM 2646 HD11 LEU A 219 7.719 11.609 -60.851 1.00 47.08 H
ATOM 2647 HD12 LEU A 219 6.433 12.419 -61.726 1.00 47.08 H
ATOM 2648 HD13 LEU A 219 7.117 10.932 -62.346 1.00 47.08 H
ATOM 2649 HD21 LEU A 219 9.401 13.009 -60.728 1.00 50.44 H
ATOM 2650 HD22 LEU A 219 10.491 12.664 -62.054 1.00 50.44 H
ATOM 2651 HD23 LEU A 219 9.721 14.238 -61.934 1.00 50.44 H
ATOM 2652 N THR A 220 11.432 11.401 -65.976 1.00 47.93 N
ATOM 2653 CA THR A 220 11.973 10.459 -66.947 1.00 54.51 C
ATOM 2654 C THR A 220 12.137 9.101 -66.280 1.00 50.06 C
ATOM 2655 O THR A 220 12.628 9.014 -65.150 1.00 53.32 O
ATOM 2656 CB THR A 220 13.346 10.919 -67.498 1.00 64.09 C
ATOM 2657 CG2 THR A 220 13.219 12.177 -68.366 1.00 68.94 C
ATOM 2658 OG1 THR A 220 14.282 11.196 -66.472 1.00 76.32 O
ATOM 2659 H THR A 220 12.044 11.663 -65.214 1.00 47.93 H
ATOM 2660 HA THR A 220 11.297 10.328 -67.795 1.00 54.51 H
ATOM 2661 HB THR A 220 13.769 10.124 -68.116 1.00 64.09 H
ATOM 2662 HG1 THR A 220 14.255 10.473 -65.833 1.00 76.32 H
ATOM 2663 HG21 THR A 220 14.192 12.513 -68.723 1.00 68.94 H
ATOM 2664 HG22 THR A 220 12.596 11.984 -69.240 1.00 68.94 H
ATOM 2665 HG23 THR A 220 12.757 12.994 -67.815 1.00 68.94 H
ATOM 2666 N PHE A 221 11.726 8.049 -66.978 1.00 46.79 N
ATOM 2667 CA PHE A 221 11.647 6.718 -66.403 1.00 48.89 C
ATOM 2668 C PHE A 221 12.604 5.770 -67.112 1.00 53.99 C
ATOM 2669 O PHE A 221 13.079 6.039 -68.218 1.00 48.36 O
ATOM 2670 CB PHE A 221 10.201 6.204 -66.561 1.00 45.77 C
ATOM 2671 CG PHE A 221 9.164 6.982 -65.776 1.00 52.56 C
ATOM 2672 CD1 PHE A 221 8.883 6.636 -64.438 1.00 47.57 C
ATOM 2673 CD2 PHE A 221 8.593 8.154 -66.316 1.00 49.89 C
ATOM 2674 CE1 PHE A 221 7.989 7.399 -63.702 1.00 54.42 C
ATOM 2675 CE2 PHE A 221 7.712 8.910 -65.559 1.00 49.18 C

ATOM 2676 CZ PHE A 221 7.403 8.529 -64.261 1.00 45.75 C
ATOM 2677 H PHE A 221 11.366 8.160 -67.914 1.00 46.79 H
ATOM 2678 HA PHE A 221 11.929 6.718 -65.348 1.00 48.89 H
ATOM 2679 HB2 PHE A 221 9.913 6.211 -67.613 1.00 45.77 H
ATOM 2680 HB3 PHE A 221 10.144 5.161 -66.253 1.00 45.77 H
ATOM 2681 HD1 PHE A 221 9.339 5.767 -63.986 1.00 47.57 H
ATOM 2682 HD2 PHE A 221 8.826 8.470 -67.323 1.00 49.89 H
ATOM 2683 HE1 PHE A 221 7.742 7.099 -62.696 1.00 54.42 H
ATOM 2684 HE2 PHE A 221 7.262 9.794 -65.989 1.00 49.18 H
ATOM 2685 HZ PHE A 221 6.695 9.112 -63.692 1.00 45.75 H
ATOM 2686 N SER A 222 12.882 4.650 -66.451 1.00 54.95 N
ATOM 2687 CA SER A 222 13.674 3.595 -67.057 1.00 57.52 C
ATOM 2688 C SER A 222 12.869 2.895 -68.150 1.00 50.96 C
ATOM 2689 O SER A 222 11.653 3.067 -68.276 1.00 47.72 O
ATOM 2690 CB SER A 222 14.119 2.584 -66.000 1.00 59.56 C
ATOM 2691 OG SER A 222 14.819 3.217 -64.942 1.00 65.98 O
ATOM 2692 H SER A 222 12.504 4.472 -65.532 1.00 54.95 H
ATOM 2693 HA SER A 222 14.568 4.032 -67.508 1.00 57.52 H
ATOM 2694 HB2 SER A 222 13.259 2.062 -65.578 1.00 59.56 H
ATOM 2695 HB3 SER A 222 14.769 1.824 -66.435 1.00 59.56 H
ATOM 2696 HG SER A 222 15.082 2.549 -64.304 1.00 65.98 H
ATOM 2697 N HIS A 223 13.563 2.092 -68.941 1.00 51.15 N
ATOM 2698 CA HIS A 223 12.876 1.338 -69.979 1.00 54.24 C
ATOM 2699 C HIS A 223 12.220 0.096 -69.377 1.00 48.11 C
ATOM 2700 O HIS A 223 12.795 -0.541 -68.491 1.00 54.20 O
ATOM 2701 CB HIS A 223 13.852 0.927 -71.081 1.00 60.35 C
ATOM 2702 CG HIS A 223 14.639 2.068 -71.646 1.00 76.95 C
ATOM 2703 CD2 HIS A 223 14.063 3.070 -72.397 1.00 82.30 C
ATOM 2704 ND1 HIS A 223 15.958 2.363 -71.574 1.00 81.43 N
ATOM 2705 CE1 HIS A 223 16.152 3.529 -72.275 1.00 85.70 C
ATOM 2706 NE2 HIS A 223 14.993 3.934 -72.761 1.00 85.52 N
ATOM 2707 H HIS A 223 14.557 1.955 -68.830 1.00 51.15 H
ATOM 2708 HA HIS A 223 12.173 2.028 -70.440 1.00 54.24 H
ATOM 2709 HB2 HIS A 223 14.562 0.188 -70.704 1.00 60.35 H
ATOM 2710 HB3 HIS A 223 13.314 0.445 -71.899 1.00 60.35 H
ATOM 2711 HD2 HIS A 223 13.032 3.210 -72.688 1.00 82.30 H
ATOM 2712 HE1 HIS A 223 17.099 4.032 -72.406 1.00 85.70 H
ATOM 2713 HE2 HIS A 223 14.845 4.765 -73.316 1.00 0.00 H
ATOM 2714 N PRO A 224 11.003 -0.257 -69.819 1.00 47.34 N
ATOM 2715 CA PRO A 224 10.189 0.546 -70.738 1.00 45.22 C

ATOM 2716 C PRO A 224 9.408 1.622 -69.990 1.00 44.62 C
ATOM 2717 O PRO A 224 8.883 1.352 -68.908 1.00 41.02 O
ATOM 2718 CB PRO A 224 9.252 -0.485 -71.359 1.00 46.82 C
ATOM 2719 CG PRO A 224 9.065 -1.497 -70.280 1.00 46.77 C
ATOM 2720 CD PRO A 224 10.376 -1.558 -69.526 1.00 46.51 C
ATOM 2721 HA PRO A 224 10.758 0.983 -71.559 1.00 45.22 H
ATOM 2722 HB2 PRO A 224 8.314 -0.041 -71.673 1.00 46.82 H
ATOM 2723 HB3 PRO A 224 9.722 -0.943 -72.230 1.00 46.82 H
ATOM 2724 HG2 PRO A 224 8.282 -1.148 -69.613 1.00 46.77 H
ATOM 2725 HG3 PRO A 224 8.746 -2.469 -70.658 1.00 46.77 H
ATOM 2726 HD2 PRO A 224 10.226 -1.719 -68.457 1.00 46.51 H
ATOM 2727 HD3 PRO A 224 11.010 -2.356 -69.915 1.00 46.51 H
ATOM 2728 N THR A 225 9.337 2.825 -70.565 1.00 44.25 N
ATOM 2729 CA THR A 225 8.732 3.950 -69.858 1.00 45.39 C
ATOM 2730 C THR A 225 7.260 3.696 -69.559 1.00 38.94 C
ATOM 2731 O THR A 225 6.763 4.085 -68.497 1.00 42.54 O
ATOM 2732 CB THR A 225 8.897 5.236 -70.669 1.00 43.86 C
ATOM 2733 CG2 THR A 225 10.368 5.597 -70.800 1.00 47.12 C
ATOM 2734 OG1 THR A 225 8.337 5.050 -71.974 1.00 48.48 O
ATOM 2735 H THR A 225 9.757 3.014 -71.464 1.00 44.25 H
ATOM 2736 HA THR A 225 9.243 4.074 -68.901 1.00 45.39 H
ATOM 2737 HB THR A 225 8.371 6.059 -70.181 1.00 43.86 H
ATOM 2738 HG1 THR A 225 7.384 4.972 -71.889 1.00 48.48 H
ATOM 2739 HG21 THR A 225 10.490 6.536 -71.341 1.00 47.12 H
ATOM 2740 HG22 THR A 225 10.829 5.724 -69.821 1.00 47.12 H
ATOM 2741 HG23 THR A 225 10.937 4.836 -71.336 1.00 47.12 H
ATOM 2742 N TRP A 226 6.548 3.032 -70.475 1.00 38.02 N
ATOM 2743 CA TRP A 226 5.122 2.803 -70.270 1.00 39.85 C
ATOM 2744 C TRP A 226 4.839 1.877 -69.093 1.00 47.19 C
ATOM 2745 O TRP A 226 3.725 1.900 -68.558 1.00 43.69 O
ATOM 2746 CB TRP A 226 4.476 2.249 -71.544 1.00 42.72 C
ATOM 2747 CG TRP A 226 5.272 1.189 -72.245 1.00 50.16 C
ATOM 2748 CD1 TRP A 226 6.125 1.366 -73.297 1.00 54.02 C
ATOM 2749 CD2 TRP A 226 5.277 -0.214 -71.957 1.00 46.58 C
ATOM 2750 CE2 TRP A 226 6.161 -0.825 -72.869 1.00 52.52 C
ATOM 2751 CE3 TRP A 226 4.624 -1.013 -71.013 1.00 44.50 C
ATOM 2752 NE1 TRP A 226 6.665 0.161 -73.676 1.00 52.01 N
ATOM 2753 CZ2 TRP A 226 6.409 -2.197 -72.865 1.00 54.11 C
ATOM 2754 CZ3 TRP A 226 4.871 -2.376 -71.011 1.00 40.82 C
ATOM 2755 CH2 TRP A 226 5.756 -2.953 -71.930 1.00 38.28 C

ATOM 2756 H TRP A 226 6.969 2.691 -71.327 1.00 38.02 H
ATOM 2757 HA TRP A 226 4.632 3.751 -70.036 1.00 39.85 H
ATOM 2758 HB2 TRP A 226 3.468 1.878 -71.351 1.00 42.72 H
ATOM 2759 HB3 TRP A 226 4.350 3.070 -72.251 1.00 42.72 H
ATOM 2760 HD1 TRP A 226 6.335 2.322 -73.755 1.00 54.02 H
ATOM 2761 HE1 TRP A 226 7.319 0.048 -74.437 1.00 52.01 H
ATOM 2762 HE3 TRP A 226 3.935 -0.582 -70.302 1.00 44.50 H
ATOM 2763 HZ2 TRP A 226 7.092 -2.647 -73.571 1.00 54.11 H
ATOM 2764 HZ3 TRP A 226 4.373 -3.004 -70.287 1.00 40.82 H
ATOM 2765 HH2 TRP A 226 5.928 -4.019 -71.900 1.00 38.28 H
ATOM 2766 N TYR A 227 5.810 1.070 -68.670 1.00 36.79 N
ATOM 2767 CA TYR A 227 5.608 0.270 -67.467 1.00 34.92 C
ATOM 2768 C TYR A 227 5.864 1.099 -66.213 1.00 35.02 C
ATOM 2769 O TYR A 227 4.987 1.237 -65.353 1.00 36.23 O
ATOM 2770 CB TYR A 227 6.510 -0.970 -67.489 1.00 34.39 C
ATOM 2771 CG TYR A 227 6.290 -1.906 -66.316 1.00 37.16 C
ATOM 2772 CD1 TYR A 227 6.934 -1.694 -65.099 1.00 38.71 C
ATOM 2773 CD2 TYR A 227 5.434 -2.998 -66.422 1.00 34.42 C
ATOM 2774 CE1 TYR A 227 6.731 -2.540 -64.025 1.00 38.49 C
ATOM 2775 CE2 TYR A 227 5.224 -3.849 -65.351 1.00 36.70 C
ATOM 2776 CZ TYR A 227 5.879 -3.615 -64.156 1.00 36.69 C
ATOM 2777 OH TYR A 227 5.682 -4.458 -63.089 1.00 38.13 O
ATOM 2778 H TYR A 227 6.725 1.067 -69.100 1.00 36.79 H
ATOM 2779 HA TYR A 227 4.581 -0.100 -67.424 1.00 34.92 H
ATOM 2780 HB2 TYR A 227 6.331 -1.530 -68.407 1.00 34.39 H
ATOM 2781 HB3 TYR A 227 7.562 -0.682 -67.506 1.00 34.39 H
ATOM 2782 HD1 TYR A 227 7.607 -0.860 -64.988 1.00 38.71 H
ATOM 2783 HD2 TYR A 227 4.918 -3.187 -67.352 1.00 34.42 H
ATOM 2784 HE1 TYR A 227 7.244 -2.357 -63.092 1.00 38.49 H
ATOM 2785 HE2 TYR A 227 4.555 -4.691 -65.451 1.00 36.70 H
ATOM 2786 HH TYR A 227 6.185 -4.207 -62.311 1.00 38.13 H
ATOM 2787 N TRP A 228 7.066 1.668 -66.098 1.00 35.69 N
ATOM 2788 CA TRP A 228 7.461 2.303 -64.845 1.00 39.72 C
ATOM 2789 C TRP A 228 6.696 3.596 -64.585 1.00 35.87 C
ATOM 2790 O TRP A 228 6.458 3.944 -63.423 1.00 36.20 O
ATOM 2791 CB TRP A 228 8.971 2.545 -64.841 1.00 39.16 C
ATOM 2792 CG TRP A 228 9.737 1.263 -64.944 1.00 40.87 C
ATOM 2793 CD1 TRP A 228 10.457 0.817 -66.016 1.00 40.68 C
ATOM 2794 CD2 TRP A 228 9.825 0.238 -63.948 1.00 40.69 C
ATOM 2795 CE2 TRP A 228 10.626 -0.793 -64.478 1.00 44.15 C

ATOM	2796	CE3	TRP	A	228	9.311	0.096	-62.654	1.00	42.68	C
ATOM	2797	NE1	TRP	A	228	11.000	-0.414	-65.741	1.00	43.77	N
ATOM	2798	CZ2	TRP	A	228	10.926	-1.948	-63.761	1.00	45.24	C
ATOM	2799	CZ3	TRP	A	228	9.608	-1.055	-61.944	1.00	43.77	C
ATOM	2800	CH2	TRP	A	228	10.408	-2.061	-62.500	1.00	43.51	C
ATOM	2801	H	TRP	A	228	7.766	1.572	-66.821	1.00	35.69	H
ATOM	2802	HA	TRP	A	228	7.226	1.625	-64.022	1.00	39.72	H
ATOM	2803	HB2	TRP	A	228	9.251	3.185	-65.677	1.00	39.16	H
ATOM	2804	HB3	TRP	A	228	9.279	3.058	-63.929	1.00	39.16	H
ATOM	2805	HD1	TRP	A	228	10.563	1.355	-66.941	1.00	40.68	H
ATOM	2806	HE1	TRP	A	228	11.578	-0.930	-66.391	1.00	43.77	H
ATOM	2807	HE3	TRP	A	228	8.701	0.873	-62.216	1.00	42.68	H
ATOM	2808	HZ2	TRP	A	228	11.543	-2.727	-64.185	1.00	45.24	H
ATOM	2809	HZ3	TRP	A	228	9.224	-1.178	-60.942	1.00	43.77	H
ATOM	2810	HH2	TRP	A	228	10.623	-2.946	-61.919	1.00	43.51	H
ATOM	2811	N	GLU	A	229	6.300	4.318	-65.636	1.00	35.95	N
ATOM	2812	CA	GLU	A	229	5.499	5.520	-65.421	1.00	42.81	C
ATOM	2813	C	GLU	A	229	4.133	5.172	-64.847	1.00	36.50	C
ATOM	2814	O	GLU	A	229	3.654	5.833	-63.918	1.00	39.47	O
ATOM	2815	CB	GLU	A	229	5.394	6.295	-66.759	1.00	39.70	C
ATOM	2816	CG	GLU	A	229	4.518	7.566	-66.727	1.00	61.33	C
ATOM	2817	CD	GLU	A	229	4.485	8.349	-68.043	1.00	69.54	C
ATOM	2818	OE1	GLU	A	229	5.407	8.177	-68.869	1.00	68.17	O
ATOM	2819	OE2	GLU	A	229	3.519	9.125	-68.205	1.00	74.45	O1-
ATOM	2820	H	GLU	A	229	6.506	4.044	-66.589	1.00	35.95	H
ATOM	2821	HA	GLU	A	229	5.999	6.158	-64.698	1.00	42.81	H
ATOM	2822	HB2	GLU	A	229	6.401	6.557	-67.086	1.00	39.70	H
ATOM	2823	HB3	GLU	A	229	4.992	5.634	-67.527	1.00	39.70	H
ATOM	2824	HG2	GLU	A	229	3.493	7.283	-66.495	1.00	61.33	H
ATOM	2825	HG3	GLU	A	229	4.855	8.236	-65.936	1.00	61.33	H
ATOM	2826	N	ASN	A	230	3.493	4.133	-65.381	1.00	35.07	N
ATOM	2827	CA	ASN	A	230	2.183	3.750	-64.876	1.00	41.13	C
ATOM	2828	C	ASN	A	230	2.279	3.089	-63.507	1.00	42.78	C
ATOM	2829	O	ASN	A	230	1.373	3.249	-62.682	1.00	40.17	O
ATOM	2830	CB	ASN	A	230	1.488	2.847	-65.890	1.00	41.06	C
ATOM	2831	CG	ASN	A	230	1.051	3.608	-67.124	1.00	42.59	C
ATOM	2832	ND2	ASN	A	230	1.450	3.129	-68.297	1.00	41.24	N
ATOM	2833	OD1	ASN	A	230	0.380	4.633	-67.021	1.00	44.14	O
ATOM	2834	H	ASN	A	230	3.896	3.590	-66.131	1.00	35.07	H
ATOM	2835	HA	ASN	A	230	1.592	4.663	-64.766	1.00	41.13	H

ATOM 2836 HB2 ASN A 230 2.121 1.996 -66.151 1.00 41.06 H
ATOM 2837 HB3 ASN A 230 0.580 2.429 -65.452 1.00 41.06 H
ATOM 2838 HD21 ASN A 230 2.085 2.340 -68.345 1.00 41.24 H
ATOM 2839 HD22 ASN A 230 1.181 3.597 -69.150 1.00 41.24 H
ATOM 2840 N LEU A 231 3.369 2.367 -63.237 1.00 41.11 N
ATOM 2841 CA LEU A 231 3.573 1.831 -61.894 1.00 38.53 C
ATOM 2842 C LEU A 231 3.663 2.951 -60.865 1.00 40.74 C
ATOM 2843 O LEU A 231 3.099 2.844 -59.769 1.00 42.65 O
ATOM 2844 CB LEU A 231 4.832 0.966 -61.849 1.00 36.91 C
ATOM 2845 CG LEU A 231 5.149 0.389 -60.464 1.00 48.84 C
ATOM 2846 CD1 LEU A 231 4.047 -0.561 -60.005 1.00 43.51 C
ATOM 2847 CD2 LEU A 231 6.500 -0.304 -60.448 1.00 48.08 C
ATOM 2848 H LEU A 231 4.090 2.212 -63.930 1.00 41.11 H
ATOM 2849 HA LEU A 231 2.711 1.208 -61.647 1.00 38.53 H
ATOM 2850 HB2 LEU A 231 4.744 0.156 -62.576 1.00 36.91 H
ATOM 2851 HB3 LEU A 231 5.682 1.565 -62.178 1.00 36.91 H
ATOM 2852 HG LEU A 231 5.232 1.183 -59.722 1.00 48.84 H
ATOM 2853 HD11 LEU A 231 4.415 -1.415 -59.437 1.00 43.51 H
ATOM 2854 HD12 LEU A 231 3.340 -0.042 -59.358 1.00 43.51 H
ATOM 2855 HD13 LEU A 231 3.476 -0.961 -60.844 1.00 43.51 H
ATOM 2856 HD21 LEU A 231 6.504 -1.253 -59.913 1.00 48.08 H
ATOM 2857 HD22 LEU A 231 6.840 -0.518 -61.460 1.00 48.08 H
ATOM 2858 HD23 LEU A 231 7.254 0.328 -59.976 1.00 48.08 H
ATOM 2859 N LEU A 232 4.368 4.037 -61.198 1.00 36.08 N
ATOM 2860 CA LEU A 232 4.448 5.171 -60.282 1.00 37.60 C
ATOM 2861 C LEU A 232 3.067 5.753 -60.014 1.00 33.96 C
ATOM 2862 O LEU A 232 2.700 6.004 -58.860 1.00 37.61 O
ATOM 2863 CB LEU A 232 5.424 6.269 -60.785 1.00 40.68 C
ATOM 2864 CG LEU A 232 5.656 7.430 -59.769 1.00 38.72 C
ATOM 2865 CD1 LEU A 232 6.402 6.963 -58.499 1.00 40.78 C
ATOM 2866 CD2 LEU A 232 6.315 8.666 -60.415 1.00 43.48 C
ATOM 2867 H LEU A 232 4.843 4.109 -62.088 1.00 36.08 H
ATOM 2868 HA LEU A 232 4.853 4.808 -59.336 1.00 37.60 H
ATOM 2869 HB2 LEU A 232 6.384 5.818 -61.043 1.00 40.68 H
ATOM 2870 HB3 LEU A 232 5.032 6.679 -61.718 1.00 40.68 H
ATOM 2871 HG LEU A 232 4.683 7.794 -59.437 1.00 38.72 H
ATOM 2872 HD11 LEU A 232 7.247 7.602 -58.240 1.00 40.78 H
ATOM 2873 HD12 LEU A 232 5.732 6.966 -57.638 1.00 40.78 H
ATOM 2874 HD13 LEU A 232 6.795 5.951 -58.600 1.00 40.78 H
ATOM 2875 HD21 LEU A 232 5.693 9.551 -60.270 1.00 43.48 H

ATOM   2876 HD22 LEU A 232   7.293   8.901 -59.996 1.00 43.48   H
ATOM   2877 HD23 LEU A 232   6.442   8.546 -61.488 1.00 43.48   H
ATOM   2878 N   LYS A 233   2.286   5.974 -61.075 1.00 31.01   N
ATOM   2879 CA LYS A 233   0.939   6.509 -60.906 1.00 35.83   C
ATOM   2880 C   LYS A 233   0.087   5.592 -60.039 1.00 38.98   C
ATOM   2881 O   LYS A 233   -0.673   6.062 -59.184 1.00 35.70   O
ATOM   2882 CB LYS A 233   0.275   6.718 -62.282 1.00 32.70   C
ATOM   2883 CG LYS A 233   0.932   7.823 -63.124 1.00 33.94   C
ATOM   2884 CD LYS A 233   0.429   7.823 -64.575 1.00 34.02   C
ATOM   2885 CE LYS A 233   1.176   8.829 -65.461 1.00 41.13   C
ATOM   2886 NZ LYS A 233   0.878   8.602 -66.893 1.00 48.12   N1+
ATOM   2887 H   LYS A 233   2.604   5.771 -62.013 1.00 31.01   H
ATOM   2888 HA LYS A 233   1.015   7.474 -60.401 1.00 35.83   H
ATOM   2889 HB2 LYS A 233   0.279   5.774 -62.830 1.00 32.70   H
ATOM   2890 HB3 LYS A 233   -0.777   6.978 -62.142 1.00 32.70   H
ATOM   2891 HG2 LYS A 233   0.734   8.793 -62.663 1.00 33.94   H
ATOM   2892 HG3 LYS A 233   2.016   7.705 -63.116 1.00 33.94   H
ATOM   2893 HD2 LYS A 233   0.534   6.822 -64.997 1.00 34.02   H
ATOM   2894 HD3 LYS A 233   -0.637   8.050 -64.589 1.00 34.02   H
ATOM   2895 HE2 LYS A 233   0.924   9.854 -65.190 1.00 41.13   H
ATOM   2896 HE3 LYS A 233   2.250   8.723 -65.317 1.00 41.13   H
ATOM   2897 HZ1 LYS A 233   1.030   7.629 -67.116 1.00 48.12   H
ATOM   2898 HZ2 LYS A 233   -0.080   8.848 -67.093 1.00 48.12   H
ATOM   2899 HZ3 LYS A 233   1.507   9.161 -67.456 1.00 48.12   H
ATOM   2900 N   ILE A 234   0.213   4.278 -60.237 1.00 37.77   N
ATOM   2901 CA ILE A 234   -0.554   3.322 -59.446 1.00 35.96   C
ATOM   2902 C   ILE A 234   -0.105   3.342 -57.991 1.00 33.24   C
ATOM   2903 O   ILE A 234   -0.934   3.321 -57.074 1.00 42.00   O
ATOM   2904 CB ILE A 234   -0.488   1.881 -60.042 1.00 41.35   C
ATOM   2905 CG1 ILE A 234   -1.330   1.781 -61.338 1.00 49.39   C
ATOM   2906 CG2 ILE A 234   -0.837   0.722 -59.078 1.00 41.05   C
ATOM   2907 CD1 ILE A 234   -2.843   2.006 -61.162 1.00 63.19   C
ATOM   2908 H   ILE A 234   0.826   3.911 -60.954 1.00 37.77   H
ATOM   2909 HA ILE A 234   -1.596   3.642 -59.427 1.00 35.96   H
ATOM   2910 HB ILE A 234   0.547   1.710 -60.344 1.00 41.35   H
ATOM   2911 HG12 ILE A 234   -0.970   2.516 -62.057 1.00 49.39   H
ATOM   2912 HG13 ILE A 234   -1.158   0.814 -61.812 1.00 49.39   H
ATOM   2913 HG21 ILE A 234   -0.865 -0.232 -59.606 1.00 41.05   H
ATOM   2914 HG22 ILE A 234   -0.094   0.615 -58.287 1.00 41.05   H
ATOM   2915 HG23 ILE A 234   -1.806   0.864 -58.600 1.00 41.05   H

ATOM 2916 HD11 ILE A 234 -3.407 1.423 -61.890 1.00 63.19 H
ATOM 2917 HD12 ILE A 234 -3.201 1.724 -60.173 1.00 63.19 H
ATOM 2918 HD13 ILE A 234 -3.099 3.054 -61.317 1.00 63.19 H
ATOM 2919 N CYS A 235 1.210 3.378 -57.754 1.00 39.51 N
ATOM 2920 CA CYS A 235 1.711 3.399 -56.383 1.00 42.53 C
ATOM 2921 C CYS A 235 1.305 4.678 -55.665 1.00 44.32 C
ATOM 2922 O CYS A 235 0.994 4.653 -54.469 1.00 40.71 O
ATOM 2923 CB CYS A 235 3.247 3.289 -56.334 1.00 47.98 C
ATOM 2924 SG CYS A 235 3.780 1.654 -56.913 1.00 59.39 S
ATOM 2925 H CYS A 235 1.885 3.374 -58.510 1.00 39.51 H
ATOM 2926 HA CYS A 235 1.322 2.536 -55.852 1.00 42.53 H
ATOM 2927 HB2 CYS A 235 3.718 4.062 -56.945 1.00 47.98 H
ATOM 2928 HB3 CYS A 235 3.612 3.421 -55.314 1.00 47.98 H
ATOM 2929 HG CYS A 235 3.499 1.892 -58.205 1.00 59.39 H
ATOM 2930 N VAL A 236 1.313 5.809 -56.374 1.00 39.48 N
ATOM 2931 CA VAL A 236 0.850 7.054 -55.771 1.00 40.98 C
ATOM 2932 C VAL A 236 -0.627 6.950 -55.422 1.00 33.44 C
ATOM 2933 O VAL A 236 -1.052 7.349 -54.332 1.00 39.94 O
ATOM 2934 CB VAL A 236 1.143 8.307 -56.644 1.00 40.85 C
ATOM 2935 CG1 VAL A 236 0.516 9.611 -56.101 1.00 34.49 C
ATOM 2936 CG2 VAL A 236 2.661 8.531 -56.808 1.00 37.62 C
ATOM 2937 H VAL A 236 1.605 5.827 -57.344 1.00 39.48 H
ATOM 2938 HA VAL A 236 1.398 7.211 -54.841 1.00 40.98 H
ATOM 2939 HB VAL A 236 0.738 8.123 -57.640 1.00 40.85 H
ATOM 2940 HG11 VAL A 236 0.869 10.482 -56.651 1.00 34.49 H
ATOM 2941 HG12 VAL A 236 -0.572 9.612 -56.178 1.00 34.49 H
ATOM 2942 HG13 VAL A 236 0.771 9.771 -55.052 1.00 34.49 H
ATOM 2943 HG21 VAL A 236 2.861 9.216 -57.632 1.00 37.62 H
ATOM 2944 HG22 VAL A 236 3.105 8.957 -55.909 1.00 37.62 H
ATOM 2945 HG23 VAL A 236 3.201 7.612 -57.024 1.00 37.62 H
ATOM 2946 N PHE A 237 -1.427 6.392 -56.333 1.00 34.16 N
ATOM 2947 CA PHE A 237 -2.862 6.272 -56.098 1.00 38.59 C
ATOM 2948 C PHE A 237 -3.168 5.402 -54.884 1.00 38.19 C
ATOM 2949 O PHE A 237 -4.100 5.696 -54.127 1.00 42.09 O
ATOM 2950 CB PHE A 237 -3.562 5.770 -57.380 1.00 35.21 C
ATOM 2951 CG PHE A 237 -5.076 5.598 -57.322 1.00 41.21 C
ATOM 2952 CD1 PHE A 237 -5.897 6.552 -56.679 1.00 41.72 C
ATOM 2953 CD2 PHE A 237 -5.692 4.571 -58.069 1.00 40.94 C
ATOM 2954 CE1 PHE A 237 -7.279 6.441 -56.736 1.00 45.53 C
ATOM 2955 CE2 PHE A 237 -7.076 4.474 -58.110 1.00 37.81 C

```
ATOM   2956  CZ  PHE A 237      -7.866   5.403 -57.444  1.00 41.50           C
ATOM   2957  H   PHE A 237      -1.066   6.069 -57.222  1.00 34.16           H
ATOM   2958  HA  PHE A 237      -3.254   7.271 -55.901  1.00 38.59           H
ATOM   2959  HB2 PHE A 237      -3.353   6.459 -58.199  1.00 35.21           H
ATOM   2960  HB3 PHE A 237      -3.119   4.817 -57.672  1.00 35.21           H
ATOM   2961  HD1 PHE A 237      -5.463   7.371 -56.125  1.00 41.72           H
ATOM   2962  HD2 PHE A 237      -5.092   3.856 -58.613  1.00 40.94           H
ATOM   2963  HE1 PHE A 237      -7.900   7.168 -56.232  1.00 45.53           H
ATOM   2964  HE2 PHE A 237      -7.542   3.679 -58.674  1.00 37.81           H
ATOM   2965  HZ  PHE A 237      -8.943   5.322 -57.486  1.00 41.50           H
ATOM   2966  N   ILE A 238      -2.384   4.350 -54.658  0.57 38.27           N
ATOM   2967  CA  ILE A 238      -2.703   3.419 -53.581  0.57 40.87           C
ATOM   2968  C   ILE A 238      -2.078   3.821 -52.245  0.57 39.50           C
ATOM   2969  O   ILE A 238      -2.669   3.566 -51.192  0.57 41.36           O
ATOM   2970  CB  ILE A 238      -2.335   1.942 -53.902  0.57 47.23           C
ATOM   2971  CG1 ILE A 238      -0.818   1.683 -54.023  0.57 47.57           C
ATOM   2972  CG2 ILE A 238      -3.128   1.456 -55.135  0.57 45.13           C
ATOM   2973  CD1 ILE A 238      -0.428   0.272 -54.482  0.57 46.50           C
ATOM   2974  H   ILE A 238      -1.627   4.112 -55.286  0.57 38.27           H
ATOM   2975  HA  ILE A 238      -3.775   3.411 -53.469  0.57 40.87           H
ATOM   2976  HB  ILE A 238      -2.683   1.340 -53.060  0.57 47.23           H
ATOM   2977 HG12 ILE A 238      -0.423   2.404 -54.725  0.57 47.57           H
ATOM   2978 HG13 ILE A 238      -0.315   1.879 -53.077  0.57 47.57           H
ATOM   2979 HG21 ILE A 238      -3.014   0.384 -55.293  0.57 45.13           H
ATOM   2980 HG22 ILE A 238      -4.195   1.643 -55.015  0.57 45.13           H
ATOM   2981 HG23 ILE A 238      -2.813   1.958 -56.048  0.57 45.13           H
ATOM   2982 HD11 ILE A 238       0.611   0.058 -54.230  0.57 46.50           H
ATOM   2983 HD12 ILE A 238      -1.046  -0.490 -54.006  0.57 46.50           H
ATOM   2984 HD13 ILE A 238      -0.530   0.167 -55.563  0.57 46.50           H
ATOM   2985  N   PHE A 239      -0.898   4.446 -52.251  1.00 35.93           N
ATOM   2986  CA  PHE A 239      -0.277   4.879 -51.005  1.00 39.60           C
ATOM   2987  C   PHE A 239      -0.761   6.248 -50.553  1.00 39.94           C
ATOM   2988  O   PHE A 239      -0.740   6.539 -49.353  1.00 39.70           O
ATOM   2989  CB  PHE A 239       1.244   4.892 -51.145  1.00 45.10           C
ATOM   2990  CG  PHE A 239       1.878   3.549 -50.960  1.00 51.51           C
ATOM   2991  CD1 PHE A 239       2.095   3.041 -49.688  1.00 54.40           C
ATOM   2992  CD2 PHE A 239       2.259   2.793 -52.053  1.00 52.60           C
ATOM   2993  CE1 PHE A 239       2.678   1.803 -49.513  1.00 51.55           C
ATOM   2994  CE2 PHE A 239       2.844   1.557 -51.885  1.00 57.00           C
ATOM   2995  CZ  PHE A 239       3.053   1.060 -50.613  1.00 54.76           C
```

ATOM 2996 H PHE A 239 -0.403 4.631 -53.114 1.00 35.93 H
ATOM 2997 HA PHE A 239 -0.518 4.189 -50.193 1.00 39.60 H
ATOM 2998 HB2 PHE A 239 1.547 5.323 -52.099 1.00 45.10 H
ATOM 2999 HB3 PHE A 239 1.688 5.545 -50.391 1.00 45.10 H
ATOM 3000 HD1 PHE A 239 1.803 3.616 -48.821 1.00 54.40 H
ATOM 3001 HD2 PHE A 239 2.121 3.155 -53.055 1.00 52.60 H
ATOM 3002 HE1 PHE A 239 2.840 1.418 -48.517 1.00 51.55 H
ATOM 3003 HE2 PHE A 239 3.139 0.978 -52.748 1.00 57.00 H
ATOM 3004 HZ PHE A 239 3.510 0.091 -50.480 1.00 54.76 H
ATOM 3005 N ALA A 240 -1.185 7.102 -51.478 1.00 39.12 N
ATOM 3006 CA ALA A 240 -1.645 8.429 -51.102 1.00 41.39 C
ATOM 3007 C ALA A 240 -3.157 8.533 -51.005 1.00 40.28 C
ATOM 3008 O ALA A 240 -3.657 9.479 -50.389 1.00 43.92 O
ATOM 3009 CB ALA A 240 -1.147 9.472 -52.113 1.00 41.15 C
ATOM 3010 H ALA A 240 -1.179 6.870 -52.463 1.00 39.12 H
ATOM 3011 HA ALA A 240 -1.243 8.730 -50.133 1.00 41.39 H
ATOM 3012 HB1 ALA A 240 -1.313 10.475 -51.724 1.00 41.15 H
ATOM 3013 HB2 ALA A 240 -0.085 9.383 -52.326 1.00 41.15 H
ATOM 3014 HB3 ALA A 240 -1.680 9.411 -53.061 1.00 41.15 H
ATOM 3015 N PHE A 241 -3.899 7.589 -51.579 1.00 36.85 N
ATOM 3016 CA PHE A 241 -5.349 7.694 -51.526 1.00 37.48 C
ATOM 3017 C PHE A 241 -6.003 6.433 -50.973 1.00 36.07 C
ATOM 3018 O PHE A 241 -6.689 6.493 -49.949 1.00 38.51 O
ATOM 3019 CB PHE A 241 -5.917 8.019 -52.908 1.00 39.16 C
ATOM 3020 CG PHE A 241 -7.392 8.253 -52.898 1.00 37.29 C
ATOM 3021 CD1 PHE A 241 -7.914 9.420 -52.364 1.00 38.43 C
ATOM 3022 CD2 PHE A 241 -8.261 7.295 -53.389 1.00 47.36 C
ATOM 3023 CE1 PHE A 241 -9.273 9.632 -52.336 1.00 47.74 C
ATOM 3024 CE2 PHE A 241 -9.622 7.502 -53.365 1.00 45.98 C
ATOM 3025 CZ PHE A 241 -10.130 8.669 -52.840 1.00 45.33 C
ATOM 3026 H PHE A 241 -3.489 6.816 -52.084 1.00 36.85 H
ATOM 3027 HA PHE A 241 -5.691 8.486 -50.857 1.00 37.48 H
ATOM 3028 HB2 PHE A 241 -5.443 8.928 -53.270 1.00 39.16 H
ATOM 3029 HB3 PHE A 241 -5.709 7.267 -53.656 1.00 39.16 H
ATOM 3030 HD1 PHE A 241 -7.251 10.169 -51.956 1.00 38.43 H
ATOM 3031 HD2 PHE A 241 -7.861 6.394 -53.824 1.00 47.36 H
ATOM 3032 HE1 PHE A 241 -9.663 10.540 -51.900 1.00 47.74 H
ATOM 3033 HE2 PHE A 241 -10.290 6.747 -53.753 1.00 45.98 H
ATOM 3034 HZ PHE A 241 -11.200 8.788 -52.794 1.00 45.33 H
ATOM 3035 N ILE A 242 -5.796 5.290 -51.634 1.00 41.27 N

ATOM 3036 CA ILE A 242 -6.531 4.076 -51.276 1.00 42.31 C
ATOM 3037 C ILE A 242 -6.198 3.641 -49.852 1.00 41.45 C
ATOM 3038 O ILE A 242 -7.088 3.469 -49.013 1.00 46.71 O
ATOM 3039 CB ILE A 242 -6.242 2.948 -52.279 1.00 44.97 C
ATOM 3040 CG1 ILE A 242 -6.722 3.334 -53.681 1.00 52.29 C
ATOM 3041 CG2 ILE A 242 -6.896 1.658 -51.806 1.00 44.32 C
ATOM 3042 CD1 ILE A 242 -8.200 3.646 -53.763 1.00 53.12 C
ATOM 3043 H ILE A 242 -5.224 5.266 -52.468 1.00 41.27 H
ATOM 3044 HA ILE A 242 -7.595 4.308 -51.260 1.00 42.31 H
ATOM 3045 HB ILE A 242 -5.185 2.725 -52.277 1.00 44.97 H
ATOM 3046 HG12 ILE A 242 -6.168 4.194 -54.051 1.00 52.29 H
ATOM 3047 HG13 ILE A 242 -6.489 2.528 -54.378 1.00 52.29 H
ATOM 3048 HG21 ILE A 242 -6.861 0.901 -52.589 1.00 44.32 H
ATOM 3049 HG22 ILE A 242 -6.385 1.219 -50.949 1.00 44.32 H
ATOM 3050 HG23 ILE A 242 -7.943 1.799 -51.535 1.00 44.32 H
ATOM 3051 HD11 ILE A 242 -8.488 3.878 -54.785 1.00 53.12 H
ATOM 3052 HD12 ILE A 242 -8.803 2.787 -53.469 1.00 53.12 H
ATOM 3053 HD13 ILE A 242 -8.514 4.483 -53.148 1.00 53.12 H
ATOM 3054 N MET A 243 -4.912 3.424 -49.573 1.00 40.13 N
ATOM 3055 CA MET A 243 -4.505 3.021 -48.227 1.00 44.25 C
ATOM 3056 C MET A 243 -4.921 4.030 -47.164 1.00 43.89 C
ATOM 3057 O MET A 243 -5.448 3.608 -46.120 1.00 39.59 O
ATOM 3058 CB MET A 243 -2.993 2.773 -48.189 1.00 49.27 C
ATOM 3059 CG MET A 243 -2.449 2.433 -46.806 1.00 58.03 C
ATOM 3060 SD MET A 243 -0.653 2.584 -46.669 1.00 70.56 S
ATOM 3061 CE MET A 243 -0.450 4.363 -46.702 1.00 73.55 C
ATOM 3062 H MET A 243 -4.185 3.549 -50.268 1.00 40.13 H
ATOM 3063 HA MET A 243 -5.002 2.076 -47.996 1.00 44.25 H
ATOM 3064 HB2 MET A 243 -2.790 1.916 -48.833 1.00 49.27 H
ATOM 3065 HB3 MET A 243 -2.414 3.567 -48.640 1.00 49.27 H
ATOM 3066 HG2 MET A 243 -2.839 3.083 -46.030 1.00 58.03 H
ATOM 3067 HG3 MET A 243 -2.737 1.417 -46.534 1.00 58.03 H
ATOM 3068 HE1 MET A 243 0.611 4.611 -46.692 1.00 73.55 H
ATOM 3069 HE2 MET A 243 -0.894 4.790 -47.594 1.00 73.55 H
ATOM 3070 HE3 MET A 243 -0.920 4.818 -45.830 1.00 73.55 H
ATOM 3071 N PRO A 244 -4.716 5.341 -47.338 1.00 38.63 N
ATOM 3072 CA PRO A 244 -5.170 6.283 -46.300 1.00 38.57 C
ATOM 3073 C PRO A 244 -6.671 6.266 -46.069 1.00 36.85 C
ATOM 3074 O PRO A 244 -7.111 6.363 -44.917 1.00 38.08 O
ATOM 3075 CB PRO A 244 -4.690 7.637 -46.835 1.00 38.89 C

ATOM	3076	CG	PRO	A	244	-3.541	7.294	-47.709	1.00	37.15	C
ATOM	3077	CD	PRO	A	244	-3.901	6.020	-48.362	1.00	40.27	C
ATOM	3078	HA	PRO	A	244	-4.651	6.030	-45.376	1.00	38.57	H
ATOM	3079	HB2	PRO	A	244	-5.462	8.101	-47.452	1.00	38.89	H
ATOM	3080	HB3	PRO	A	244	-4.438	8.359	-46.063	1.00	38.89	H
ATOM	3081	HG2	PRO	A	244	-3.293	8.076	-48.407	1.00	37.15	H
ATOM	3082	HG3	PRO	A	244	-2.662	7.135	-47.082	1.00	37.15	H
ATOM	3083	HD2	PRO	A	244	-4.388	6.180	-49.277	1.00	40.27	H
ATOM	3084	HD3	PRO	A	244	-2.986	5.501	-48.603	1.00	40.27	H
ATOM	3085	N	VAL	A	245	-7.475	6.155	-47.129	1.00	34.21	N
ATOM	3086	CA	VAL	A	245	-8.923	6.085	-46.943	1.00	39.52	C
ATOM	3087	C	VAL	A	245	-9.295	4.834	-46.156	1.00	39.58	C
ATOM	3088	O	VAL	A	245	-10.163	4.873	-45.277	1.00	39.65	O
ATOM	3089	CB	VAL	A	245	-9.651	6.142	-48.300	1.00	42.28	C
ATOM	3090	CG1	VAL	A	245	-11.121	5.780	-48.131	1.00	39.81	C
ATOM	3091	CG2	VAL	A	245	-9.526	7.528	-48.910	1.00	44.96	C
ATOM	3092	H	VAL	A	245	-7.114	6.102	-48.073	1.00	34.21	H
ATOM	3093	HA	VAL	A	245	-9.237	6.953	-46.359	1.00	39.52	H
ATOM	3094	HB	VAL	A	245	-9.194	5.428	-48.987	1.00	42.28	H
ATOM	3095	HG11	VAL	A	245	-11.673	5.990	-49.048	1.00	39.81	H
ATOM	3096	HG12	VAL	A	245	-11.277	4.720	-47.927	1.00	39.81	H
ATOM	3097	HG13	VAL	A	245	-11.592	6.358	-47.335	1.00	39.81	H
ATOM	3098	HG21	VAL	A	245	-9.929	7.549	-49.923	1.00	44.96	H
ATOM	3099	HG22	VAL	A	245	-10.071	8.269	-48.324	1.00	44.96	H
ATOM	3100	HG23	VAL	A	245	-8.495	7.864	-48.964	1.00	44.96	H
ATOM	3101	N	LEU	A	246	-8.633	3.711	-46.442	1.00	34.41	N
ATOM	3102	CA	LEU	A	246	-8.915	2.488	-45.695	1.00	35.91	C
ATOM	3103	C	LEU	A	246	-8.481	2.614	-44.239	1.00	39.03	C
ATOM	3104	O	LEU	A	246	-9.201	2.179	-43.333	1.00	39.66	O
ATOM	3105	CB	LEU	A	246	-8.228	1.294	-46.357	1.00	37.16	C
ATOM	3106	CG	LEU	A	246	-8.816	0.843	-47.697	1.00	50.15	C
ATOM	3107	CD1	LEU	A	246	-7.984	-0.276	-48.303	1.00	47.65	C
ATOM	3108	CD2	LEU	A	246	-10.262	0.404	-47.520	1.00	48.16	C
ATOM	3109	H	LEU	A	246	-7.930	3.678	-47.169	1.00	34.41	H
ATOM	3110	HA	LEU	A	246	-9.992	2.317	-45.692	1.00	35.91	H
ATOM	3111	HB2	LEU	A	246	-7.171	1.529	-46.491	1.00	37.16	H
ATOM	3112	HB3	LEU	A	246	-8.250	0.441	-45.675	1.00	37.16	H
ATOM	3113	HG	LEU	A	246	-8.804	1.682	-48.392	1.00	50.15	H
ATOM	3114	HD11	LEU	A	246	-7.696	-0.028	-49.324	1.00	47.65	H
ATOM	3115	HD12	LEU	A	246	-7.061	-0.450	-47.749	1.00	47.65	H

ATOM 3116 HD13 LEU A 246 -8.507 -1.232 -48.340 1.00 47.65 H
ATOM 3117 HD21 LEU A 246 -10.536 -0.429 -48.168 1.00 48.16 H
ATOM 3118 HD22 LEU A 246 -10.483 0.100 -46.496 1.00 48.16 H
ATOM 3119 HD23 LEU A 246 -10.939 1.222 -47.768 1.00 48.16 H
ATOM 3120 N ILE A 247 -7.312 3.210 -43.994 1.00 34.52 N
ATOM 3121 CA ILE A 247 -6.798 3.305 -42.629 1.00 38.88 C
ATOM 3122 C ILE A 247 -7.724 4.153 -41.763 1.00 36.89 C
ATOM 3123 O ILE A 247 -8.126 3.741 -40.668 1.00 37.49 O
ATOM 3124 CB ILE A 247 -5.361 3.859 -42.628 1.00 39.10 C
ATOM 3125 CG1 ILE A 247 -4.383 2.814 -43.170 1.00 43.11 C
ATOM 3126 CG2 ILE A 247 -4.953 4.286 -41.223 1.00 38.28 C
ATOM 3127 CD1 ILE A 247 -2.954 3.313 -43.274 1.00 44.54 C
ATOM 3128 H ILE A 247 -6.727 3.552 -44.747 1.00 34.52 H
ATOM 3129 HA ILE A 247 -6.779 2.300 -42.201 1.00 38.88 H
ATOM 3130 HB ILE A 247 -5.316 4.736 -43.277 1.00 39.10 H
ATOM 3131 HG12 ILE A 247 -4.391 1.949 -42.506 1.00 43.11 H
ATOM 3132 HG13 ILE A 247 -4.701 2.424 -44.133 1.00 43.11 H
ATOM 3133 HG21 ILE A 247 -3.912 4.596 -41.173 1.00 38.28 H
ATOM 3134 HG22 ILE A 247 -5.503 5.154 -40.858 1.00 38.28 H
ATOM 3135 HG23 ILE A 247 -5.080 3.476 -40.504 1.00 38.28 H
ATOM 3136 HD11 ILE A 247 -2.353 2.630 -43.873 1.00 44.54 H
ATOM 3137 HD12 ILE A 247 -2.904 4.299 -43.737 1.00 44.54 H
ATOM 3138 HD13 ILE A 247 -2.469 3.367 -42.300 1.00 44.54 H
ATOM 3139 N ILE A 248 -8.086 5.345 -42.243 1.00 34.92 N
ATOM 3140 CA ILE A 248 -8.834 6.267 -41.392 1.00 35.13 C
ATOM 3141 C ILE A 248 -10.285 5.825 -41.231 1.00 39.95 C
ATOM 3142 O ILE A 248 -10.882 6.032 -40.168 1.00 40.16 O
ATOM 3143 CB ILE A 248 -8.739 7.710 -41.925 1.00 34.18 C
ATOM 3144 CG1 ILE A 248 -9.462 7.855 -43.265 1.00 41.24 C
ATOM 3145 CG2 ILE A 248 -7.275 8.142 -42.036 1.00 34.38 C
ATOM 3146 CD1 ILE A 248 -9.547 9.284 -43.766 1.00 42.90 C
ATOM 3147 H ILE A 248 -7.771 5.675 -43.147 1.00 34.92 H
ATOM 3148 HA ILE A 248 -8.381 6.253 -40.406 1.00 35.13 H
ATOM 3149 HB ILE A 248 -9.224 8.366 -41.200 1.00 34.18 H
ATOM 3150 HG12 ILE A 248 -9.070 7.206 -44.032 1.00 41.24 H
ATOM 3151 HG13 ILE A 248 -10.505 7.564 -43.171 1.00 41.24 H
ATOM 3152 HG21 ILE A 248 -7.189 9.195 -42.303 1.00 34.38 H
ATOM 3153 HG22 ILE A 248 -6.762 8.017 -41.085 1.00 34.38 H
ATOM 3154 HG23 ILE A 248 -6.714 7.575 -42.775 1.00 34.38 H
ATOM 3155 HD11 ILE A 248 -10.364 9.400 -44.479 1.00 42.90 H

ATOM 3156 HD12 ILE A 248 -9.716 9.991 -42.953 1.00 42.90 H
ATOM 3157 HD13 ILE A 248 -8.629 9.575 -44.278 1.00 42.90 H
ATOM 3158 N THR A 249 -10.882 5.211 -42.255 1.00 33.10 N
ATOM 3159 CA THR A 249 -12.263 4.761 -42.103 1.00 37.62 C
ATOM 3160 C THR A 249 -12.352 3.561 -41.164 1.00 37.73 C
ATOM 3161 O THR A 249 -13.276 3.474 -40.347 1.00 39.51 O
ATOM 3162 CB THR A 249 -12.882 4.431 -43.464 1.00 39.02 C
ATOM 3163 CG2 THR A 249 -13.026 5.695 -44.310 1.00 39.43 C
ATOM 3164 OG1 THR A 249 -12.066 3.477 -44.155 1.00 46.06 O
ATOM 3165 H THR A 249 -10.406 5.032 -43.128 1.00 33.10 H
ATOM 3166 HA THR A 249 -12.858 5.566 -41.665 1.00 37.62 H
ATOM 3167 HB THR A 249 -13.869 3.985 -43.330 1.00 39.02 H
ATOM 3168 HG1 THR A 249 -11.334 3.947 -44.574 1.00 46.06 H
ATOM 3169 HG21 THR A 249 -13.387 5.457 -45.311 1.00 39.43 H
ATOM 3170 HG22 THR A 249 -13.752 6.368 -43.855 1.00 39.43 H
ATOM 3171 HG23 THR A 249 -12.097 6.251 -44.418 1.00 39.43 H
ATOM 3172 N VAL A 250 -11.397 2.634 -41.252 1.00 39.37 N
ATOM 3173 CA VAL A 250 -11.436 1.460 -40.383 1.00 38.80 C
ATOM 3174 C VAL A 250 -11.073 1.838 -38.952 1.00 39.21 C
ATOM 3175 O VAL A 250 -11.734 1.406 -38.000 1.00 39.16 O
ATOM 3176 CB VAL A 250 -10.518 0.352 -40.930 1.00 41.51 C
ATOM 3177 CG1 VAL A 250 -10.369 -0.772 -39.911 1.00 42.18 C
ATOM 3178 CG2 VAL A 250 -11.074 -0.193 -42.236 1.00 43.79 C
ATOM 3179 H VAL A 250 -10.653 2.698 -41.935 1.00 39.37 H
ATOM 3180 HA VAL A 250 -12.454 1.064 -40.367 1.00 38.80 H
ATOM 3181 HB VAL A 250 -9.527 0.764 -41.125 1.00 41.51 H
ATOM 3182 HG11 VAL A 250 -9.859 -1.627 -40.356 1.00 42.18 H
ATOM 3183 HG12 VAL A 250 -9.769 -0.484 -39.047 1.00 42.18 H
ATOM 3184 HG13 VAL A 250 -11.338 -1.124 -39.555 1.00 42.18 H
ATOM 3185 HG21 VAL A 250 -10.399 -0.931 -42.670 1.00 43.79 H
ATOM 3186 HG22 VAL A 250 -12.039 -0.678 -42.084 1.00 43.79 H
ATOM 3187 HG23 VAL A 250 -11.219 0.586 -42.983 1.00 43.79 H
ATOM 3188 N CYS A 251 -10.028 2.648 -38.772 1.00 29.75 N
ATOM 3189 CA CYS A 251 -9.617 3.031 -37.423 1.00 33.57 C
ATOM 3190 C CYS A 251 -10.692 3.862 -36.735 1.00 37.57 C
ATOM 3191 O CYS A 251 -11.067 3.581 -35.591 1.00 37.09 O
ATOM 3192 CB CYS A 251 -8.288 3.789 -37.469 1.00 28.43 C
ATOM 3193 SG CYS A 251 -6.864 2.730 -37.803 1.00 41.82 S
ATOM 3194 H CYS A 251 -9.486 3.000 -39.552 1.00 29.75 H
ATOM 3195 HA CYS A 251 -9.470 2.127 -36.827 1.00 33.57 H

ATOM 3196 HB2 CYS A 251 -8.329 4.551 -38.244 1.00 28.43 H
ATOM 3197 HB3 CYS A 251 -8.088 4.332 -36.549 1.00 28.43 H
ATOM 3198 HG CYS A 251 -7.177 2.487 -39.079 1.00 41.82 H
ATOM 3199 N TYR A 252 -11.209 4.885 -37.421 1.00 33.61 N
ATOM 3200 CA TYR A 252 -12.259 5.708 -36.829 1.00 34.00 C
ATOM 3201 C TYR A 252 -13.556 4.925 -36.672 1.00 42.08 C
ATOM 3202 O TYR A 252 -14.289 5.117 -35.694 1.00 37.40 O
ATOM 3203 CB TYR A 252 -12.486 6.959 -37.674 1.00 30.89 C
ATOM 3204 CG TYR A 252 -11.427 8.023 -37.495 1.00 37.96 C
ATOM 3205 CD1 TYR A 252 -10.905 8.308 -36.238 1.00 37.64 C
ATOM 3206 CD2 TYR A 252 -10.950 8.745 -38.583 1.00 33.81 C
ATOM 3207 CE1 TYR A 252 -9.938 9.283 -36.070 1.00 39.15 C
ATOM 3208 CE2 TYR A 252 -9.982 9.719 -38.426 1.00 38.63 C
ATOM 3209 CZ TYR A 252 -9.482 9.985 -37.169 1.00 40.56 C
ATOM 3210 OH TYR A 252 -8.519 10.954 -37.009 1.00 36.30 O
ATOM 3211 H TYR A 252 -10.903 5.110 -38.359 1.00 33.61 H
ATOM 3212 HA TYR A 252 -11.962 5.965 -35.817 1.00 34.00 H
ATOM 3213 HB2 TYR A 252 -12.692 6.731 -38.718 1.00 30.89 H
ATOM 3214 HB3 TYR A 252 -13.390 7.435 -37.303 1.00 30.89 H
ATOM 3215 HD1 TYR A 252 -11.251 7.809 -35.349 1.00 37.64 H
ATOM 3216 HD2 TYR A 252 -11.343 8.553 -39.571 1.00 33.81 H
ATOM 3217 HE1 TYR A 252 -9.548 9.483 -35.085 1.00 39.15 H
ATOM 3218 HE2 TYR A 252 -9.627 10.267 -39.287 1.00 38.63 H
ATOM 3219 HH TYR A 252 -8.240 11.340 -37.844 1.00 36.30 H
ATOM 3220 N GLY A 253 -13.857 4.042 -37.626 1.00 34.67 N
ATOM 3221 CA GLY A 253 -15.051 3.221 -37.506 1.00 38.78 C
ATOM 3222 C GLY A 253 -14.990 2.284 -36.315 1.00 39.48 C
ATOM 3223 O GLY A 253 -15.953 2.160 -35.557 1.00 39.96 O
ATOM 3224 H GLY A 253 -13.264 3.910 -38.435 1.00 34.67 H
ATOM 3225 HA2 GLY A 253 -15.939 3.853 -37.442 1.00 38.78 H
ATOM 3226 HA3 GLY A 253 -15.156 2.624 -38.412 1.00 38.78 H
ATOM 3227 N LEU A 254 -13.853 1.607 -36.137 1.00 36.56 N
ATOM 3228 CA LEU A 254 -13.692 0.735 -34.979 1.00 40.39 C
ATOM 3229 C LEU A 254 -13.660 1.532 -33.681 1.00 43.46 C
ATOM 3230 O LEU A 254 -14.106 1.037 -32.640 1.00 39.76 O
ATOM 3231 CB LEU A 254 -12.422 -0.101 -35.122 1.00 32.00 C
ATOM 3232 CG LEU A 254 -12.453 -1.159 -36.229 1.00 41.11 C
ATOM 3233 CD1 LEU A 254 -11.169 -1.976 -36.235 1.00 42.60 C
ATOM 3234 CD2 LEU A 254 -13.672 -2.057 -36.072 1.00 41.99 C
ATOM 3235 H LEU A 254 -13.074 1.693 -36.779 1.00 36.56 H

ATOM 3236 HA LEU A 254 -14.560 0.079 -34.911 1.00 40.39 H
ATOM 3237 HB2 LEU A 254 -11.568 0.561 -35.276 1.00 32.00 H
ATOM 3238 HB3 LEU A 254 -12.236 -0.614 -34.177 1.00 32.00 H
ATOM 3239 HG LEU A 254 -12.548 -0.684 -37.201 1.00 41.11 H
ATOM 3240 HD11 LEU A 254 -10.779 -2.073 -37.248 1.00 42.60 H
ATOM 3241 HD12 LEU A 254 -10.381 -1.515 -35.643 1.00 42.60 H
ATOM 3242 HD13 LEU A 254 -11.309 -2.984 -35.844 1.00 42.60 H
ATOM 3243 HD21 LEU A 254 -13.487 -3.068 -36.437 1.00 41.99 H
ATOM 3244 HD22 LEU A 254 -13.992 -2.148 -35.033 1.00 41.99 H
ATOM 3245 HD23 LEU A 254 -14.514 -1.670 -36.647 1.00 41.99 H
ATOM 3246 N MET A 255 -13.138 2.760 -33.727 1.00 35.93 N
ATOM 3247 CA MET A 255 -13.140 3.616 -32.546 1.00 38.38 C
ATOM 3248 C MET A 255 -14.564 3.970 -32.131 1.00 42.89 C
ATOM 3249 O MET A 255 -14.911 3.906 -30.945 1.00 42.02 O
ATOM 3250 CB MET A 255 -12.322 4.877 -32.831 1.00 37.59 C
ATOM 3251 CG MET A 255 -12.210 5.852 -31.674 1.00 45.32 C
ATOM 3252 SD MET A 255 -11.495 7.429 -32.185 1.00 43.07 S
ATOM 3253 CE MET A 255 -12.892 8.181 -33.019 1.00 38.56 C
ATOM 3254 H MET A 255 -12.742 3.131 -34.581 1.00 35.93 H
ATOM 3255 HA MET A 255 -12.664 3.082 -31.721 1.00 38.38 H
ATOM 3256 HB2 MET A 255 -11.313 4.582 -33.121 1.00 37.59 H
ATOM 3257 HB3 MET A 255 -12.734 5.390 -33.695 1.00 37.59 H
ATOM 3258 HG2 MET A 255 -13.164 6.054 -31.192 1.00 45.32 H
ATOM 3259 HG3 MET A 255 -11.554 5.420 -30.928 1.00 45.32 H
ATOM 3260 HE1 MET A 255 -12.644 9.197 -33.327 1.00 38.56 H
ATOM 3261 HE2 MET A 255 -13.174 7.616 -33.907 1.00 38.56 H
ATOM 3262 HE3 MET A 255 -13.753 8.222 -32.352 1.00 38.56 H
ATOM 3263 N ILE A 256 -15.405 4.334 -33.100 1.00 37.56 N
ATOM 3264 CA ILE A 256 -16.792 4.677 -32.803 1.00 44.45 C
ATOM 3265 C ILE A 256 -17.558 3.450 -32.320 1.00 48.12 C
ATOM 3266 O ILE A 256 -18.368 3.538 -31.390 1.00 44.25 O
ATOM 3267 CB ILE A 256 -17.452 5.318 -34.037 1.00 44.45 C
ATOM 3268 CG1 ILE A 256 -16.907 6.730 -34.247 1.00 47.29 C
ATOM 3269 CG2 ILE A 256 -18.969 5.334 -33.899 1.00 44.34 C
ATOM 3270 CD1 ILE A 256 -17.409 7.392 -35.512 1.00 58.01 C
ATOM 3271 H ILE A 256 -15.100 4.393 -34.064 1.00 37.56 H
ATOM 3272 HA ILE A 256 -16.802 5.408 -31.991 1.00 44.45 H
ATOM 3273 HB ILE A 256 -17.201 4.724 -34.918 1.00 44.45 H
ATOM 3274 HG12 ILE A 256 -17.177 7.357 -33.395 1.00 47.29 H
ATOM 3275 HG13 ILE A 256 -15.818 6.723 -34.270 1.00 47.29 H

ATOM 3276 HG21 ILE A 256 -19.451 5.836 -34.736 1.00 44.34 H
ATOM 3277 HG22 ILE A 256 -19.413 4.338 -33.899 1.00 44.34 H
ATOM 3278 HG23 ILE A 256 -19.284 5.849 -32.990 1.00 44.34 H
ATOM 3279 HD11 ILE A 256 -16.896 8.327 -35.693 1.00 58.01 H
ATOM 3280 HD12 ILE A 256 -17.259 6.755 -36.384 1.00 58.01 H
ATOM 3281 HD13 ILE A 256 -18.466 7.629 -35.441 1.00 58.01 H
ATOM 3282 N LEU A 257 -17.306 2.288 -32.930 1.00 40.65 N
ATOM 3283 CA LEU A 257 -17.991 1.069 -32.508 1.00 50.46 C
ATOM 3284 C LEU A 257 -17.675 0.728 -31.057 1.00 39.24 C
ATOM 3285 O LEU A 257 -18.563 0.311 -30.304 1.00 47.40 O
ATOM 3286 CB LEU A 257 -17.615 -0.093 -33.429 1.00 53.32 C
ATOM 3287 CG LEU A 257 -18.181 -0.034 -34.850 1.00 64.57 C
ATOM 3288 CD1 LEU A 257 -17.673 -1.203 -35.685 1.00 67.76 C
ATOM 3289 CD2 LEU A 257 -19.702 -0.008 -34.824 1.00 62.78 C
ATOM 3290 H LEU A 257 -16.654 2.226 -33.702 1.00 40.65 H
ATOM 3291 HA LEU A 257 -19.066 1.243 -32.565 1.00 50.46 H
ATOM 3292 HB2 LEU A 257 -16.527 -0.164 -33.479 1.00 53.32 H
ATOM 3293 HB3 LEU A 257 -17.945 -1.030 -32.976 1.00 53.32 H
ATOM 3294 HG LEU A 257 -17.888 0.885 -35.341 1.00 64.57 H
ATOM 3295 HD11 LEU A 257 -17.113 -0.840 -36.547 1.00 67.76 H
ATOM 3296 HD12 LEU A 257 -16.999 -1.850 -35.122 1.00 67.76 H
ATOM 3297 HD13 LEU A 257 -18.472 -1.840 -36.065 1.00 67.76 H
ATOM 3298 HD21 LEU A 257 -20.141 -0.407 -35.739 1.00 62.78 H
ATOM 3299 HD22 LEU A 257 -20.093 -0.594 -33.994 1.00 62.78 H
ATOM 3300 HD23 LEU A 257 -20.072 1.013 -34.720 1.00 62.78 H
ATOM 3301 N ARG A 258 -16.414 0.890 -30.647 0.65 45.62 N
ATOM 3302 CA ARG A 258 -16.061 0.639 -29.252 0.65 46.59 C
ATOM 3303 C ARG A 258 -16.746 1.636 -28.327 0.65 47.31 C
ATOM 3304 O ARG A 258 -17.241 1.262 -27.256 0.65 52.72 O
ATOM 3305 CB ARG A 258 -14.542 0.694 -29.072 0.65 43.30 C
ATOM 3306 CG ARG A 258 -14.073 0.510 -27.631 0.65 42.45 C
ATOM 3307 CD ARG A 258 -14.350 -0.902 -27.123 0.65 46.50 C
ATOM 3308 NE ARG A 258 -13.640 -1.910 -27.907 0.65 48.61 N
ATOM 3309 CZ ARG A 258 -12.408 -2.333 -27.642 0.65 48.89 C
ATOM 3310 NH1 ARG A 258 -11.741 -1.838 -26.607 0.65 49.73 N1+
ATOM 3311 NH2 ARG A 258 -11.842 -3.251 -28.413 0.65 49.03 N1+
ATOM 3312 H ARG A 258 -15.689 1.211 -31.275 0.65 45.62 H
ATOM 3313 HA ARG A 258 -16.415 -0.364 -29.011 0.65 46.59 H
ATOM 3314 HB2 ARG A 258 -14.077 -0.047 -29.723 0.65 43.30 H
ATOM 3315 HB3 ARG A 258 -14.177 1.657 -29.432 0.65 43.30 H

ATOM 3316 HG2 ARG A 258 -12.989 0.625 -27.663 0.65 42.45 H
ATOM 3317 HG3 ARG A 258 -14.417 1.272 -26.932 0.65 42.45 H
ATOM 3318 HD2 ARG A 258 -14.228 -1.000 -26.044 0.65 46.50 H
ATOM 3319 HD3 ARG A 258 -15.398 -1.139 -27.292 0.65 46.50 H
ATOM 3320 HE ARG A 258 -14.122 -2.271 -28.718 0.65 48.61 H
ATOM 3321 HH11 ARG A 258 -12.163 -1.132 -26.022 0.65 49.73 H
ATOM 3322 HH12 ARG A 258 -10.796 -2.140 -26.414 0.65 49.73 H
ATOM 3323 HH21 ARG A 258 -12.339 -3.632 -29.206 0.65 49.03 H
ATOM 3324 HH22 ARG A 258 -10.904 -3.574 -28.217 0.65 49.03 H
ATOM 3325 N LEU A 259 -16.794 2.909 -28.727 1.00 42.90 N
ATOM 3326 CA LEU A 259 -17.423 3.922 -27.887 1.00 51.48 C
ATOM 3327 C LEU A 259 -18.933 3.736 -27.801 1.00 58.39 C
ATOM 3328 O LEU A 259 -19.540 4.099 -26.788 1.00 57.30 O
ATOM 3329 CB LEU A 259 -17.089 5.320 -28.409 1.00 46.17 C
ATOM 3330 CG LEU A 259 -15.641 5.769 -28.207 1.00 43.80 C
ATOM 3331 CD1 LEU A 259 -15.375 7.083 -28.926 1.00 41.89 C
ATOM 3332 CD2 LEU A 259 -15.329 5.894 -26.720 1.00 38.49 C
ATOM 3333 H LEU A 259 -16.380 3.206 -29.601 1.00 42.90 H
ATOM 3334 HA LEU A 259 -17.037 3.825 -26.870 1.00 51.48 H
ATOM 3335 HB2 LEU A 259 -17.334 5.357 -29.472 1.00 46.17 H
ATOM 3336 HB3 LEU A 259 -17.741 6.056 -27.934 1.00 46.17 H
ATOM 3337 HG LEU A 259 -14.993 5.014 -28.639 1.00 43.80 H
ATOM 3338 HD11 LEU A 259 -14.497 7.000 -29.565 1.00 41.89 H
ATOM 3339 HD12 LEU A 259 -16.205 7.381 -29.568 1.00 41.89 H
ATOM 3340 HD13 LEU A 259 -15.197 7.916 -28.247 1.00 41.89 H
ATOM 3341 HD21 LEU A 259 -14.674 6.735 -26.490 1.00 38.49 H
ATOM 3342 HD22 LEU A 259 -16.227 6.012 -26.113 1.00 38.49 H
ATOM 3343 HD23 LEU A 259 -14.815 5.000 -26.366 1.00 38.49 H
ATOM 3344 N LYS A 260 -19.559 3.183 -28.841 1.00 45.85 N
ATOM 3345 CA LYS A 260 -20.995 2.944 -28.786 1.00 53.40 C
ATOM 3346 C LYS A 260 -21.353 1.681 -28.012 1.00 56.66 C
ATOM 3347 O LYS A 260 -22.532 1.480 -27.698 1.00 58.41 O
ATOM 3348 CB LYS A 260 -21.582 2.877 -30.203 1.00 65.77 C
ATOM 3349 CG LYS A 260 -21.754 1.475 -30.770 1.00 76.08 C
ATOM 3350 CD LYS A 260 -22.435 1.523 -32.133 1.00 85.40 C
ATOM 3351 CE LYS A 260 -22.787 0.133 -32.643 1.00 91.86 C
ATOM 3352 NZ LYS A 260 -23.407 0.172 -33.999 1.00 94.60 N1+
ATOM 3353 H LYS A 260 -19.062 2.932 -29.687 1.00 45.85 H
ATOM 3354 HA LYS A 260 -21.511 3.743 -28.267 1.00 53.40 H
ATOM 3355 HB2 LYS A 260 -22.579 3.318 -30.154 1.00 65.77 H

ATOM 3356 HB3 LYS A 260 -21.024 3.518 -30.887 1.00 65.77 H
ATOM 3357 HG2 LYS A 260 -20.800 0.971 -30.862 1.00 76.08 H
ATOM 3358 HG3 LYS A 260 -22.396 0.842 -30.159 1.00 76.08 H
ATOM 3359 HD2 LYS A 260 -23.339 2.131 -32.079 1.00 85.40 H
ATOM 3360 HD3 LYS A 260 -21.773 2.017 -32.846 1.00 85.40 H
ATOM 3361 HE2 LYS A 260 -21.900 -0.498 -32.675 1.00 91.86 H
ATOM 3362 HE3 LYS A 260 -23.489 -0.349 -31.961 1.00 91.86 H
ATOM 3363 HZ1 LYS A 260 -22.760 0.586 -34.656 1.00 94.60 H
ATOM 3364 HZ2 LYS A 260 -23.630 -0.767 -34.296 1.00 94.60 H
ATOM 3365 HZ3 LYS A 260 -24.253 0.723 -33.969 1.00 94.60 H
ATOM 3366 N SER A 261 -20.371 0.845 -27.676 1.00 54.91 N
ATOM 3367 CA SER A 261 -20.635 -0.396 -26.961 1.00 60.10 C
ATOM 3368 C SER A 261 -20.632 -0.234 -25.448 1.00 63.52 C
ATOM 3369 O SER A 261 -21.109 -1.132 -24.747 1.00 69.84 O
ATOM 3370 CB SER A 261 -19.604 -1.459 -27.351 1.00 59.03 C
ATOM 3371 OG SER A 261 -18.314 -1.104 -26.881 1.00 60.20 O
ATOM 3372 H SER A 261 -19.416 1.040 -27.943 1.00 54.91 H
ATOM 3373 HA SER A 261 -21.612 -0.789 -27.248 1.00 60.10 H
ATOM 3374 HB2 SER A 261 -19.871 -2.430 -26.933 1.00 59.03 H
ATOM 3375 HB3 SER A 261 -19.569 -1.583 -28.434 1.00 59.03 H
ATOM 3376 HG SER A 261 -18.064 -0.242 -27.232 1.00 60.20 H
ATOM 3377 N VAL A 262 -20.108 0.874 -24.924 1.00 64.66 N
ATOM 3378 CA VAL A 262 -20.054 1.058 -23.479 1.00 69.71 C
ATOM 3379 C VAL A 262 -21.449 1.371 -22.953 1.00 78.62 C
ATOM 3380 O VAL A 262 -22.229 2.091 -23.590 1.00 75.83 O
ATOM 3381 CB VAL A 262 -19.039 2.156 -23.113 1.00 72.15 C
ATOM 3382 CG1 VAL A 262 -19.406 3.473 -23.753 1.00 71.18 C
ATOM 3383 CG2 VAL A 262 -18.939 2.310 -21.602 1.00 72.08 C
ATOM 3384 H VAL A 262 -19.719 1.600 -25.508 1.00 64.66 H
ATOM 3385 HA VAL A 262 -19.698 0.128 -23.028 1.00 69.71 H
ATOM 3386 HB VAL A 262 -18.067 1.845 -23.499 1.00 72.15 H
ATOM 3387 HG11 VAL A 262 -18.538 4.125 -23.856 1.00 71.18 H
ATOM 3388 HG12 VAL A 262 -19.821 3.299 -24.722 1.00 71.18 H
ATOM 3389 HG13 VAL A 262 -20.159 4.012 -23.177 1.00 71.18 H
ATOM 3390 HG21 VAL A 262 -18.132 2.996 -21.342 1.00 72.08 H
ATOM 3391 HG22 VAL A 262 -19.848 2.709 -21.151 1.00 72.08 H
ATOM 3392 HG23 VAL A 262 -18.718 1.357 -21.120 1.00 72.08 H
ATOM 3393 N ARG A 263 -21.763 0.750 -21.842 1.00 88.59 N
ATOM 3394 CA ARG A 263 -23.011 0.921 -21.181 1.00 98.61 C
ATOM 3395 C ARG A 263 -22.588 1.393 -19.866 1.00103.24 C

ATOM 3396 O ARG A 263 -21.887 0.716 -19.170 1.00106.40 O
ATOM 3397 CB ARG A 263 -23.671 -0.416 -20.997 1.00103.46 C
ATOM 3398 CG ARG A 263 -24.047 -1.077 -22.294 1.00108.12 C
ATOM 3399 CD ARG A 263 -25.009 -0.216 -23.092 1.00114.04 C
ATOM 3400 NE ARG A 263 -24.895 -0.506 -24.512 1.00116.98 N
ATOM 3401 CZ ARG A 263 -25.294 0.306 -25.475 1.00116.62 C
ATOM 3402 NH1 ARG A 263 -25.857 1.470 -25.179 1.00116.97 N1+
ATOM 3403 NH2 ARG A 263 -25.133 -0.053 -26.733 1.00115.34 N1+
ATOM 3404 H ARG A 263 -21.089 0.158 -21.378 1.00 88.59 H
ATOM 3405 HA ARG A 263 -23.652 1.684 -21.620 1.00 98.61 H
ATOM 3406 HB2 ARG A 263 -23.036 -1.115 -20.449 1.00103.46 H
ATOM 3407 HB3 ARG A 263 -24.574 -0.293 -20.395 1.00103.46 H
ATOM 3408 HG2 ARG A 263 -23.166 -1.343 -22.880 1.00108.12 H
ATOM 3409 HG3 ARG A 263 -24.540 -2.018 -22.047 1.00108.12 H
ATOM 3410 HD2 ARG A 263 -26.028 -0.514 -22.845 1.00114.04 H
ATOM 3411 HD3 ARG A 263 -24.969 0.846 -22.887 1.00114.04 H
ATOM 3412 HE ARG A 263 -24.502 -1.405 -24.755 1.00116.98 H
ATOM 3413 HH11 ARG A 263 -26.002 1.726 -24.213 1.00116.97 H
ATOM 3414 HH12 ARG A 263 -26.228 2.062 -25.908 1.00116.97 H
ATOM 3415 HH21 ARG A 263 -24.727 -0.951 -26.958 1.00115.34 H
ATOM 3416 HH22 ARG A 263 -25.409 0.563 -27.484 1.00115.34 H
ATOM 3417 N MET A 264 -22.976 2.590 -19.526 1.00107.41 N
ATOM 3418 CA MET A 264 -22.628 3.076 -18.230 1.00114.28 C
ATOM 3419 C MET A 264 -23.934 3.135 -17.475 1.00113.36 C
ATOM 3420 O MET A 264 -24.834 3.845 -17.864 1.00113.15 O
ATOM 3421 CB MET A 264 -21.983 4.448 -18.338 1.00121.50 C
ATOM 3422 CG MET A 264 -21.978 5.016 -19.747 1.00125.45 C
ATOM 3423 SD MET A 264 -23.574 4.681 -20.488 1.00131.50 S
ATOM 3424 CE MET A 264 -24.577 5.893 -19.647 1.00137.27 C
ATOM 3425 H MET A 264 -23.517 3.201 -20.127 1.00107.41 H
ATOM 3426 HA MET A 264 -21.937 2.459 -17.651 1.00114.28 H
ATOM 3427 HB2 MET A 264 -22.407 5.167 -17.635 1.00121.50 H
ATOM 3428 HB3 MET A 264 -20.942 4.355 -18.027 1.00121.50 H
ATOM 3429 HG2 MET A 264 -21.780 6.088 -19.744 1.00125.45 H
ATOM 3430 HG3 MET A 264 -21.202 4.547 -20.352 1.00125.45 H
ATOM 3431 HE1 MET A 264 -25.602 5.593 -19.608 1.00137.27 H
ATOM 3432 HE2 MET A 264 -24.220 6.080 -18.638 1.00137.27 H
ATOM 3433 HE3 MET A 264 -24.253 6.714 -20.259 1.00137.27 H
ATOM 3434 N LEU A 265 -24.012 2.399 -16.379 1.00114.26 N
ATOM 3435 CA LEU A 265 -25.219 2.324 -15.562 1.00115.58 C

ATOM 3436 C LEU A 265 -25.952 3.617 -15.265 1.00115.06 C
ATOM 3437 O LEU A 265 -27.097 3.577 -14.878 1.00114.43 O
ATOM 3438 CB LEU A 265 -24.943 1.612 -14.236 1.00115.68 C
ATOM 3439 CG LEU A 265 -25.208 0.120 -14.342 1.00115.15 C
ATOM 3440 CD1 LEU A 265 -23.996 -0.620 -14.863 1.00112.15 C
ATOM 3441 CD2 LEU A 265 -25.816 -0.516 -13.099 1.00119.72 C
ATOM 3442 H LEU A 265 -23.232 1.826 -16.092 1.00114.26 H
ATOM 3443 HA LEU A 265 -25.934 1.760 -16.162 1.00115.58 H
ATOM 3444 HB2 LEU A 265 -23.939 1.821 -13.863 1.00115.68 H
ATOM 3445 HB3 LEU A 265 -25.600 1.999 -13.455 1.00115.68 H
ATOM 3446 HG LEU A 265 -25.994 -0.027 -15.084 1.00115.15 H
ATOM 3447 HD11 LEU A 265 -23.541 -1.247 -14.096 1.00112.15 H
ATOM 3448 HD12 LEU A 265 -24.270 -1.275 -15.691 1.00112.15 H
ATOM 3449 HD13 LEU A 265 -23.214 0.045 -15.229 1.00112.15 H
ATOM 3450 HD21 LEU A 265 -26.393 -1.404 -13.359 1.00119.72 H
ATOM 3451 HD22 LEU A 265 -25.049 -0.820 -12.386 1.00119.72 H
ATOM 3452 HD23 LEU A 265 -26.493 0.161 -12.578 1.00119.72 H
ATOM 3453 N SER A 266 -25.354 4.757 -15.529 1.00114.06 N
ATOM 3454 CA SER A 266 -26.043 5.981 -15.133 1.00116.49 C
ATOM 3455 C SER A 266 -26.303 6.957 -16.279 1.00117.91 C
ATOM 3456 O SER A 266 -27.440 7.083 -16.751 1.00127.10 O
ATOM 3457 CB SER A 266 -25.253 6.694 -14.038 1.00111.09 C
ATOM 3458 OG SER A 266 -25.894 7.900 -13.667 1.00114.21 O
ATOM 3459 H SER A 266 -24.434 4.810 -15.939 1.00114.06 H
ATOM 3460 HA SER A 266 -27.033 5.835 -14.697 1.00116.49 H
ATOM 3461 HB2 SER A 266 -25.187 6.059 -13.154 1.00111.09 H
ATOM 3462 HB3 SER A 266 -24.228 6.905 -14.345 1.00111.09 H
ATOM 3463 HG SER A 266 -25.383 8.319 -12.970 1.00114.21 H
ATOM 3464 N GLY A 267 -25.269 7.685 -16.706 1.00110.19 N
ATOM 3465 CA GLY A 267 -25.465 8.844 -17.560 1.00108.81 C
ATOM 3466 C GLY A 267 -25.465 8.560 -19.047 1.00109.56 C
ATOM 3467 O GLY A 267 -24.526 8.944 -19.754 1.00106.95 O
ATOM 3468 H GLY A 267 -24.351 7.548 -16.310 1.00110.19 H
ATOM 3469 HA2 GLY A 267 -26.379 9.381 -17.296 1.00108.81 H
ATOM 3470 HA3 GLY A 267 -24.653 9.540 -17.347 1.00108.81 H
ATOM 3471 N SER A 268 -26.527 7.911 -19.537 1.00117.85 N
ATOM 3472 CA SER A 268 -26.609 7.581 -20.956 1.00117.10 C
ATOM 3473 C SER A 268 -26.714 8.826 -21.828 1.00110.17 C
ATOM 3474 O SER A 268 -26.297 8.798 -22.993 1.00110.31 O
ATOM 3475 CB SER A 268 -27.799 6.660 -21.213 1.00124.17 C

```
ATOM   3476  OG  SER A 268     -27.548   5.350 -20.735  1.00128.64           O
ATOM   3477  H   SER A 268     -27.266   7.583 -18.929  1.00117.85           H
ATOM   3478  HA  SER A 268     -25.767   7.083 -21.413  1.00117.10           H
ATOM   3479  HB2 SER A 268     -28.705   7.047 -20.744  1.00124.17           H
ATOM   3480  HB3 SER A 268     -28.007   6.581 -22.282  1.00124.17           H
ATOM   3481  HG  SER A 268     -28.332   4.815 -20.881  1.00128.64           H
ATOM   3482  N   LYS A 269     -27.274   9.914 -21.295  1.00106.08           N
ATOM   3483  CA  LYS A 269     -27.382  11.145 -22.070  1.00102.33           C
ATOM   3484  C   LYS A 269     -26.012  11.758 -22.328  1.00102.78           C
ATOM   3485  O   LYS A 269     -25.737  12.234 -23.437  1.00103.12           O
ATOM   3486  CB  LYS A 269     -28.289  12.138 -21.349  1.00  0.00           C
ATOM   3487  CG  LYS A 269     -28.655  13.356 -22.203  1.00  0.00           C
ATOM   3488  CD  LYS A 269     -29.524  14.396 -21.485  1.00  0.00           C
ATOM   3489  CE  LYS A 269     -29.902  15.624 -22.325  1.00  0.00           C
ATOM   3490  NZ  LYS A 269     -30.736  16.591 -21.590  1.00  0.00           N1+
ATOM   3491  H   LYS A 269     -27.618   9.916 -20.346  1.00106.08           H
ATOM   3492  HA  LYS A 269     -27.838  10.909 -23.035  1.00102.33           H
ATOM   3493  HB2 LYS A 269     -29.216  11.634 -21.071  1.00  0.00           H
ATOM   3494  HB3 LYS A 269     -27.830  12.459 -20.412  1.00  0.00           H
ATOM   3495  HG2 LYS A 269     -27.753  13.861 -22.548  1.00  0.00           H
ATOM   3496  HG3 LYS A 269     -29.171  13.018 -23.102  1.00  0.00           H
ATOM   3497  HD2 LYS A 269     -30.437  13.910 -21.138  1.00  0.00           H
ATOM   3498  HD3 LYS A 269     -29.004  14.727 -20.586  1.00  0.00           H
ATOM   3499  HE2 LYS A 269     -28.999  16.135 -22.663  1.00  0.00           H
ATOM   3500  HE3 LYS A 269     -30.443  15.311 -23.218  1.00  0.00           H
ATOM   3501  HZ1 LYS A 269     -30.237  16.919 -20.775  1.00  0.00           H
ATOM   3502  HZ2 LYS A 269     -30.954  17.375 -22.188  1.00  0.00           H
ATOM   3503  HZ3 LYS A 269     -31.594  16.144 -21.298  1.00  0.00           H
ATOM   3504  N   GLU A 270     -25.140  11.757 -21.315  1.00103.76           N
ATOM   3505  CA  GLU A 270     -23.790  12.279 -21.494  1.00107.44           C
ATOM   3506  C   GLU A 270     -22.981  11.423 -22.460  1.00104.43           C
ATOM   3507  O   GLU A 270     -22.075  11.934 -23.130  1.00109.67           O
ATOM   3508  CB  GLU A 270     -23.083  12.374 -20.145  1.00  0.00           C
ATOM   3509  CG  GLU A 270     -22.197  13.608 -19.914  1.00  0.00           C
ATOM   3510  CD  GLU A 270     -21.495  13.692 -18.562  1.00  0.00           C
ATOM   3511  OE1 GLU A 270     -20.358  13.180 -18.462  1.00  0.00           O
ATOM   3512  OE2 GLU A 270     -22.112  14.277 -17.648  1.00  0.00           O1-
ATOM   3513  H   GLU A 270     -25.373  11.352 -20.420  1.00103.76           H
ATOM   3514  HA  GLU A 270     -23.875  13.285 -21.910  1.00107.44           H
ATOM   3515  HB2 GLU A 270     -23.826  12.358 -19.345  1.00  0.00           H
```

```
ATOM   3516  HB3 GLU A 270     -22.482  11.480 -19.967  1.00  0.00           H
ATOM   3517  HG2 GLU A 270     -21.434  13.651 -20.693  1.00  0.00           H
ATOM   3518  HG3 GLU A 270     -22.798  14.510 -20.039  1.00  0.00           H
ATOM   3519  N   LYS A 271     -23.287  10.127 -22.544  1.00100.41           N
ATOM   3520  CA  LYS A 271     -22.598   9.261 -23.496  1.00 92.31           C
ATOM   3521  C   LYS A 271     -23.097   9.482 -24.918  1.00 87.07           C
ATOM   3522  O   LYS A 271     -22.294   9.551 -25.856  1.00 85.19           O
ATOM   3523  CB  LYS A 271     -22.772   7.796 -23.102  1.00 89.83           C
ATOM   3524  CG  LYS A 271     -22.185   6.818 -24.105  1.00 90.39           C
ATOM   3525  CD  LYS A 271     -23.153   5.688 -24.411  1.00 93.35           C
ATOM   3526  CE  LYS A 271     -22.565   4.714 -25.418  1.00 88.71           C
ATOM   3527  NZ  LYS A 271     -23.491   3.581 -25.697  1.00 91.01           N1+
ATOM   3528  H   LYS A 271     -24.016   9.722 -21.973  1.00100.41           H
ATOM   3529  HA  LYS A 271     -21.527   9.476 -23.473  1.00 92.31           H
ATOM   3530  HB2 LYS A 271     -22.338   7.618 -22.117  1.00 89.83           H
ATOM   3531  HB3 LYS A 271     -23.838   7.600 -22.981  1.00 89.83           H
ATOM   3532  HG2 LYS A 271     -21.824   7.253 -25.034  1.00 90.39           H
ATOM   3533  HG3 LYS A 271     -21.289   6.386 -23.658  1.00 90.39           H
ATOM   3534  HD2 LYS A 271     -23.399   5.156 -23.491  1.00 93.35           H
ATOM   3535  HD3 LYS A 271     -24.089   6.096 -24.796  1.00 93.35           H
ATOM   3536  HE2 LYS A 271     -22.422   5.233 -26.346  1.00 88.71           H
ATOM   3537  HE3 LYS A 271     -21.608   4.335 -25.088  1.00 88.71           H
ATOM   3538  HZ1 LYS A 271     -23.102   2.991 -26.421  1.00 91.01           H
ATOM   3539  HZ2 LYS A 271     -23.596   3.034 -24.853  1.00 91.01           H
ATOM   3540  HZ3 LYS A 271     -24.386   3.943 -25.992  1.00 91.01           H
ATOM   3541  N   ASP A 272     -24.416   9.585 -25.099  1.00 85.65           N
ATOM   3542  CA  ASP A 272     -24.960   9.852 -26.426  1.00 85.08           C
ATOM   3543  C   ASP A 272     -24.478  11.195 -26.958  1.00 79.45           C
ATOM   3544  O   ASP A 272     -24.233  11.343 -28.161  1.00 71.98           O
ATOM   3545  CB  ASP A 272     -26.489   9.808 -26.394  1.00 92.27           C
ATOM   3546  CG  ASP A 272     -27.034   8.394 -26.363  1.00103.12           C
ATOM   3547  OD1 ASP A 272     -26.237   7.448 -26.199  1.00107.58           O
ATOM   3548  OD2 ASP A 272     -28.263   8.229 -26.512  1.00111.41           O1-
ATOM   3549  H   ASP A 272     -25.068   9.474 -24.333  1.00 85.65           H
ATOM   3550  HA  ASP A 272     -24.584   9.095 -27.119  1.00 85.08           H
ATOM   3551  HB2 ASP A 272     -26.862  10.348 -25.522  1.00 92.27           H
ATOM   3552  HB3 ASP A 272     -26.888  10.291 -27.288  1.00 92.27           H
ATOM   3553  N   ARG A 273     -24.325  12.183 -26.073  1.00 80.33           N
ATOM   3554  CA  ARG A 273     -23.901  13.510 -26.506  1.00 75.60           C
ATOM   3555  C   ARG A 273     -22.468  13.489 -27.022  1.00 76.97           C
```

```
ATOM   3556  O   ARG A 273     -22.182  14.006 -28.108  1.00 71.69           O
ATOM   3557  CB  ARG A 273     -24.048  14.504 -25.358  1.00  0.00           C
ATOM   3558  CG  ARG A 273     -23.898  16.017 -25.635  1.00  0.00           C
ATOM   3559  CD  ARG A 273     -24.048  17.001 -24.474  1.00  0.00           C
ATOM   3560  NE  ARG A 273     -23.880  18.394 -24.900  1.00  0.00           N
ATOM   3561  CZ  ARG A 273     -22.701  19.040 -24.953  1.00  0.00           C
ATOM   3562  NH1 ARG A 273     -21.568  18.428 -24.546  1.00  0.00           N1+
ATOM   3563  NH2 ARG A 273     -22.685  20.308 -25.412  1.00  0.00           N1+
ATOM   3564  H   ARG A 273     -24.541  12.046 -25.094  1.00 80.33           H
ATOM   3565  HA  ARG A 273     -24.549  13.837 -27.321  1.00 75.60           H
ATOM   3566  HB2 ARG A 273     -25.036  14.368 -24.918  1.00  0.00           H
ATOM   3567  HB3 ARG A 273     -23.349  14.232 -24.565  1.00  0.00           H
ATOM   3568  HG2 ARG A 273     -22.973  16.233 -26.171  1.00  0.00           H
ATOM   3569  HG3 ARG A 273     -24.694  16.248 -26.343  1.00  0.00           H
ATOM   3570  HD2 ARG A 273     -25.094  16.956 -24.169  1.00  0.00           H
ATOM   3571  HD3 ARG A 273     -23.477  16.725 -23.587  1.00  0.00           H
ATOM   3572  HE  ARG A 273     -24.710  18.867 -25.225  1.00  0.00           H
ATOM   3573 HH11 ARG A 273     -20.687  18.922 -24.541  1.00  0.00           H
ATOM   3574 HH12 ARG A 273     -21.603  17.478 -24.206  1.00  0.00           H
ATOM   3575 HH21 ARG A 273     -23.542  20.764 -25.689  1.00  0.00           H
ATOM   3576 HH22 ARG A 273     -21.818  20.823 -25.460  1.00  0.00           H
ATOM   3577  N   ASN A 274     -21.550  12.892 -26.256  1.00 81.13           N
ATOM   3578  CA  ASN A 274     -20.161  12.809 -26.699  1.00 81.73           C
ATOM   3579  C   ASN A 274     -20.029  11.976 -27.966  1.00 72.25           C
ATOM   3580  O   ASN A 274     -19.188  12.275 -28.821  1.00 71.93           O
ATOM   3581  CB  ASN A 274     -19.282  12.230 -25.588  1.00 90.34           C
ATOM   3582  CG  ASN A 274     -18.566  13.301 -24.792  1.00 93.48           C
ATOM   3583  ND2 ASN A 274     -18.733  13.404 -23.576  1.00 88.75           N
ATOM   3584  OD1 ASN A 274     -17.762  14.107 -25.476  1.00 93.91           O
ATOM   3585  H   ASN A 274     -21.794  12.468 -25.371  1.00 81.13           H
ATOM   3586  HA  ASN A 274     -19.869  13.840 -26.916  1.00 81.73           H
ATOM   3587  HB2 ASN A 274     -19.839  11.543 -24.947  1.00 90.34           H
ATOM   3588  HB3 ASN A 274     -18.473  11.633 -26.015  1.00 90.34           H
ATOM   3589 HD21 ASN A 274     -19.355  12.776 -23.087  1.00 88.75           H
ATOM   3590 HD22 ASN A 274     -18.239  14.116 -23.057  1.00 88.75           H
ATOM   3591  N   LEU A 275     -20.847  10.931 -28.108  1.00 70.70           N
ATOM   3592  CA  LEU A 275     -20.783  10.110 -29.311  1.00 66.32           C
ATOM   3593  C   LEU A 275     -21.252  10.882 -30.537  1.00 62.48           C
ATOM   3594  O   LEU A 275     -20.720  10.689 -31.636  1.00 62.81           O
ATOM   3595  CB  LEU A 275     -21.610   8.841 -29.128  1.00 72.59           C
```

ATOM 3596 CG LEU A 275 -20.820 7.591 -28.750 1.00 77.60 C
ATOM 3597 CD1 LEU A 275 -21.772 6.440 -28.509 1.00 88.31 C
ATOM 3598 CD2 LEU A 275 -19.821 7.244 -29.841 1.00 73.97 C
ATOM 3599 H LEU A 275 -21.515 10.676 -27.392 1.00 70.70 H
ATOM 3600 HA LEU A 275 -19.739 9.843 -29.487 1.00 66.32 H
ATOM 3601 HB2 LEU A 275 -22.401 9.022 -28.400 1.00 72.59 H
ATOM 3602 HB3 LEU A 275 -22.150 8.604 -30.048 1.00 72.59 H
ATOM 3603 HG LEU A 275 -20.283 7.791 -27.821 1.00 77.60 H
ATOM 3604 HD11 LEU A 275 -21.222 5.750 -27.886 1.00 88.31 H
ATOM 3605 HD12 LEU A 275 -22.684 6.749 -27.997 1.00 88.31 H
ATOM 3606 HD13 LEU A 275 -22.051 5.926 -29.430 1.00 88.31 H
ATOM 3607 HD21 LEU A 275 -19.794 6.174 -30.054 1.00 73.97 H
ATOM 3608 HD22 LEU A 275 -20.033 7.749 -30.784 1.00 73.97 H
ATOM 3609 HD23 LEU A 275 -18.813 7.532 -29.544 1.00 73.97 H
ATOM 3610 N ARG A 276 -22.251 11.754 -30.375 1.00 61.37 N
ATOM 3611 CA ARG A 276 -22.689 12.576 -31.498 1.00 57.99 C
ATOM 3612 C ARG A 276 -21.581 13.515 -31.952 1.00 52.50 C
ATOM 3613 O ARG A 276 -21.376 13.709 -33.155 1.00 50.49 O
ATOM 3614 CB ARG A 276 -23.939 13.376 -31.127 1.00 61.23 C
ATOM 3615 CG ARG A 276 -25.203 12.552 -30.974 1.00 77.57 C
ATOM 3616 CD ARG A 276 -26.442 13.435 -31.054 1.00 82.77 C
ATOM 3617 NE ARG A 276 -26.309 14.652 -30.255 1.00 89.73 N
ATOM 3618 CZ ARG A 276 -26.683 14.760 -28.984 1.00 93.08 C
ATOM 3619 NH1 ARG A 276 -27.218 13.722 -28.356 1.00 94.99 N1+
ATOM 3620 NH2 ARG A 276 -26.523 15.908 -28.339 1.00 92.99 N1+
ATOM 3621 H ARG A 276 -22.703 11.883 -29.479 1.00 61.37 H
ATOM 3622 HA ARG A 276 -22.935 11.919 -32.335 1.00 57.99 H
ATOM 3623 HB2 ARG A 276 -23.754 13.957 -30.223 1.00 61.23 H
ATOM 3624 HB3 ARG A 276 -24.114 14.103 -31.923 1.00 61.23 H
ATOM 3625 HG2 ARG A 276 -25.247 11.911 -31.855 1.00 77.57 H
ATOM 3626 HG3 ARG A 276 -25.245 11.865 -30.144 1.00 77.57 H
ATOM 3627 HD2 ARG A 276 -26.662 13.672 -32.096 1.00 82.77 H
ATOM 3628 HD3 ARG A 276 -27.314 12.886 -30.701 1.00 82.77 H
ATOM 3629 HE ARG A 276 -25.871 15.440 -30.709 1.00 89.73 H
ATOM 3630 HH11 ARG A 276 -27.338 12.846 -28.843 1.00 94.99 H
ATOM 3631 HH12 ARG A 276 -27.506 13.790 -27.391 1.00 94.99 H
ATOM 3632 HH21 ARG A 276 -26.843 16.006 -27.385 1.00 92.99 H
ATOM 3633 HH22 ARG A 276 -26.114 16.704 -28.808 1.00 92.99 H
ATOM 3634 N ARG A 277 -20.851 14.101 -31.000 1.00 51.28 N
ATOM 3635 CA ARG A 277 -19.790 15.039 -31.353 1.00 55.03 C

ATOM 3636 C ARG A 277 -18.608 14.324 -31.997 1.00 54.69 C
ATOM 3637 O ARG A 277 -18.017 14.831 -32.958 1.00 43.51 O
ATOM 3638 CB ARG A 277 -19.345 15.809 -30.110 1.00 63.64 C
ATOM 3639 CG ARG A 277 -18.467 17.014 -30.400 1.00 74.64 C
ATOM 3640 CD ARG A 277 -18.324 17.891 -29.166 1.00 87.10 C
ATOM 3641 NE ARG A 277 -17.745 19.194 -29.482 1.00 94.98 N
ATOM 3642 CZ ARG A 277 -17.658 20.201 -28.619 1.00102.19 C
ATOM 3643 NH1 ARG A 277 -18.114 20.059 -27.381 1.00101.71 N1+
ATOM 3644 NH2 ARG A 277 -17.117 21.352 -28.993 1.00106.17 N1+
ATOM 3645 H ARG A 277 -21.026 13.923 -30.020 1.00 51.28 H
ATOM 3646 HA ARG A 277 -20.185 15.758 -32.074 1.00 55.03 H
ATOM 3647 HB2 ARG A 277 -20.253 16.166 -29.622 1.00 63.64 H
ATOM 3648 HB3 ARG A 277 -18.862 15.149 -29.386 1.00 63.64 H
ATOM 3649 HG2 ARG A 277 -17.484 16.645 -30.695 1.00 74.64 H
ATOM 3650 HG3 ARG A 277 -18.840 17.601 -31.238 1.00 74.64 H
ATOM 3651 HD2 ARG A 277 -19.267 17.975 -28.624 1.00 87.10 H
ATOM 3652 HD3 ARG A 277 -17.609 17.425 -28.486 1.00 87.10 H
ATOM 3653 HE ARG A 277 -17.339 19.297 -30.401 1.00 94.98 H
ATOM 3654 HH11 ARG A 277 -18.501 19.175 -27.087 1.00101.71 H
ATOM 3655 HH12 ARG A 277 -18.031 20.811 -26.713 1.00101.71 H
ATOM 3656 HH21 ARG A 277 -16.770 21.469 -29.934 1.00106.17 H
ATOM 3657 HH22 ARG A 277 -17.062 22.126 -28.346 1.00106.17 H
ATOM 3658 N ILE A 278 -18.249 13.145 -31.487 1.00 50.99 N
ATOM 3659 CA ILE A 278 -17.107 12.419 -32.036 1.00 49.06 C
ATOM 3660 C ILE A 278 -17.440 11.864 -33.415 1.00 48.06 C
ATOM 3661 O ILE A 278 -16.641 11.969 -34.354 1.00 53.28 O
ATOM 3662 CB ILE A 278 -16.662 11.307 -31.068 1.00 51.00 C
ATOM 3663 CG1 ILE A 278 -16.136 11.920 -29.770 1.00 57.34 C
ATOM 3664 CG2 ILE A 278 -15.596 10.432 -31.709 1.00 47.00 C
ATOM 3665 CD1 ILE A 278 -15.828 10.901 -28.697 1.00 65.15 C
ATOM 3666 H ILE A 278 -18.725 12.747 -30.687 1.00 50.99 H
ATOM 3667 HA ILE A 278 -16.271 13.113 -32.153 1.00 49.06 H
ATOM 3668 HB ILE A 278 -17.525 10.682 -30.828 1.00 51.00 H
ATOM 3669 HG12 ILE A 278 -15.224 12.477 -29.988 1.00 57.34 H
ATOM 3670 HG13 ILE A 278 -16.819 12.662 -29.363 1.00 57.34 H
ATOM 3671 HG21 ILE A 278 -15.208 9.698 -31.004 1.00 47.00 H
ATOM 3672 HG22 ILE A 278 -15.972 9.847 -32.549 1.00 47.00 H
ATOM 3673 HG23 ILE A 278 -14.749 11.022 -32.062 1.00 47.00 H
ATOM 3674 HD11 ILE A 278 -15.902 11.351 -27.707 1.00 65.15 H
ATOM 3675 HD12 ILE A 278 -16.526 10.063 -28.724 1.00 65.15 H

ATOM 3676 HD13 ILE A 278 -14.817 10.505 -28.800 1.00 65.15 H
ATOM 3677 N THR A 279 -18.625 11.266 -33.559 1.00 48.07 N
ATOM 3678 CA THR A 279 -19.038 10.744 -34.858 1.00 51.54 C
ATOM 3679 C THR A 279 -19.117 11.858 -35.894 1.00 47.86 C
ATOM 3680 O THR A 279 -18.729 11.669 -37.053 1.00 46.32 O
ATOM 3681 CB THR A 279 -20.385 10.031 -34.730 1.00 54.64 C
ATOM 3682 CG2 THR A 279 -20.781 9.386 -36.048 1.00 51.56 C
ATOM 3683 OG1 THR A 279 -20.292 9.018 -33.719 1.00 63.23 O
ATOM 3684 H THR A 279 -19.275 11.187 -32.788 1.00 48.07 H
ATOM 3685 HA THR A 279 -18.286 10.031 -35.190 1.00 51.54 H
ATOM 3686 HB THR A 279 -21.163 10.731 -34.421 1.00 54.64 H
ATOM 3687 HG1 THR A 279 -20.286 9.453 -32.860 1.00 63.23 H
ATOM 3688 HG21 THR A 279 -21.624 8.709 -35.909 1.00 51.56 H
ATOM 3689 HG22 THR A 279 -21.099 10.131 -36.777 1.00 51.56 H
ATOM 3690 HG23 THR A 279 -19.972 8.815 -36.499 1.00 51.56 H
ATOM 3691 N ARG A 280 -19.607 13.031 -35.489 1.00 46.07 N
ATOM 3692 CA ARG A 280 -19.668 14.169 -36.397 1.00 47.43 C
ATOM 3693 C ARG A 280 -18.275 14.585 -36.853 1.00 48.06 C
ATOM 3694 O ARG A 280 -18.056 14.850 -38.042 1.00 45.33 O
ATOM 3695 CB ARG A 280 -20.382 15.332 -35.711 1.00 48.92 C
ATOM 3696 CG ARG A 280 -20.939 16.389 -36.639 1.00 63.28 C
ATOM 3697 CD ARG A 280 -21.715 17.411 -35.829 1.00 78.29 C
ATOM 3698 NE ARG A 280 -20.849 18.104 -34.878 1.00 82.48 N
ATOM 3699 CZ ARG A 280 -21.228 18.495 -33.666 1.00 84.32 C
ATOM 3700 NH1 ARG A 280 -22.461 18.251 -33.242 1.00 86.38 N1+
ATOM 3701 NH2 ARG A 280 -20.371 19.122 -32.873 1.00 84.38 N1+
ATOM 3702 H ARG A 280 -19.936 13.166 -34.542 1.00 46.07 H
ATOM 3703 HA ARG A 280 -20.243 13.874 -37.278 1.00 47.43 H
ATOM 3704 HB2 ARG A 280 -21.254 14.920 -35.211 1.00 48.92 H
ATOM 3705 HB3 ARG A 280 -19.751 15.756 -34.929 1.00 48.92 H
ATOM 3706 HG2 ARG A 280 -20.118 16.881 -37.162 1.00 63.28 H
ATOM 3707 HG3 ARG A 280 -21.579 15.952 -37.406 1.00 63.28 H
ATOM 3708 HD2 ARG A 280 -22.243 18.122 -36.466 1.00 78.29 H
ATOM 3709 HD3 ARG A 280 -22.488 16.859 -35.296 1.00 78.29 H
ATOM 3710 HE ARG A 280 -19.922 18.338 -35.205 1.00 82.48 H
ATOM 3711 HH11 ARG A 280 -23.118 17.786 -33.851 1.00 86.38 H
ATOM 3712 HH12 ARG A 280 -22.759 18.540 -32.322 1.00 86.38 H
ATOM 3713 HH21 ARG A 280 -19.434 19.317 -33.194 1.00 84.38 H
ATOM 3714 HH22 ARG A 280 -20.658 19.438 -31.958 1.00 84.38 H
ATOM 3715 N MET A 281 -17.317 14.638 -35.923 1.00 41.46 N

ATOM 3716 CA MET A 281 -15.951 15.004 -36.287 1.00 49.30 C
ATOM 3717 C MET A 281 -15.314 13.951 -37.185 1.00 49.10 C
ATOM 3718 O MET A 281 -14.569 14.289 -38.112 1.00 41.14 O
ATOM 3719 CB MET A 281 -15.108 15.218 -35.031 1.00 49.22 C
ATOM 3720 CG MET A 281 -14.931 16.679 -34.656 1.00 69.05 C
ATOM 3721 SD MET A 281 -14.024 17.598 -35.916 1.00 74.17 S
ATOM 3722 CE MET A 281 -12.413 16.825 -35.799 1.00 79.13 C
ATOM 3723 H MET A 281 -17.509 14.421 -34.954 1.00 41.46 H
ATOM 3724 HA MET A 281 -15.993 15.925 -36.870 1.00 49.30 H
ATOM 3725 HB2 MET A 281 -15.554 14.691 -34.186 1.00 49.22 H
ATOM 3726 HB3 MET A 281 -14.124 14.758 -35.127 1.00 49.22 H
ATOM 3727 HG2 MET A 281 -15.905 17.148 -34.513 1.00 69.05 H
ATOM 3728 HG3 MET A 281 -14.399 16.763 -33.708 1.00 69.05 H
ATOM 3729 HE1 MET A 281 -11.699 17.371 -36.416 1.00 79.13 H
ATOM 3730 HE2 MET A 281 -12.054 16.837 -34.770 1.00 79.13 H
ATOM 3731 HE3 MET A 281 -12.440 15.796 -36.153 1.00 79.13 H
ATOM 3732 N VAL A 282 -15.591 12.672 -36.922 1.00 50.89 N
ATOM 3733 CA VAL A 282 -15.078 11.607 -37.782 1.00 44.87 C
ATOM 3734 C VAL A 282 -15.591 11.785 -39.204 1.00 42.27 C
ATOM 3735 O VAL A 282 -14.828 11.704 -40.175 1.00 45.91 O
ATOM 3736 CB VAL A 282 -15.458 10.228 -37.216 1.00 43.21 C
ATOM 3737 CG1 VAL A 282 -15.256 9.153 -38.272 1.00 42.99 C
ATOM 3738 CG2 VAL A 282 -14.636 9.921 -35.974 1.00 39.05 C
ATOM 3739 H VAL A 282 -16.176 12.409 -36.138 1.00 50.89 H
ATOM 3740 HA VAL A 282 -13.989 11.683 -37.807 1.00 44.87 H
ATOM 3741 HB VAL A 282 -16.510 10.230 -36.934 1.00 43.21 H
ATOM 3742 HG11 VAL A 282 -15.403 8.165 -37.836 1.00 42.99 H
ATOM 3743 HG12 VAL A 282 -15.982 9.205 -39.084 1.00 42.99 H
ATOM 3744 HG13 VAL A 282 -14.254 9.184 -38.703 1.00 42.99 H
ATOM 3745 HG21 VAL A 282 -14.849 8.939 -35.558 1.00 39.05 H
ATOM 3746 HG22 VAL A 282 -13.572 9.924 -36.212 1.00 39.05 H
ATOM 3747 HG23 VAL A 282 -14.774 10.650 -35.182 1.00 39.05 H
ATOM 3748 N LEU A 283 -16.894 12.036 -39.349 1.00 43.45 N
ATOM 3749 CA LEU A 283 -17.464 12.209 -40.679 1.00 47.70 C
ATOM 3750 C LEU A 283 -16.869 13.417 -41.391 1.00 47.05 C
ATOM 3751 O LEU A 283 -16.700 13.390 -42.614 1.00 41.03 O
ATOM 3752 CB LEU A 283 -18.985 12.331 -40.587 1.00 49.45 C
ATOM 3753 CG LEU A 283 -19.710 11.086 -40.066 1.00 57.36 C
ATOM 3754 CD1 LEU A 283 -21.192 11.360 -39.867 1.00 50.34 C
ATOM 3755 CD2 LEU A 283 -19.501 9.905 -41.002 1.00 60.15 C

ATOM 3756 H LEU A 283 -17.515 12.091 -38.552 1.00 43.45 H
ATOM 3757 HA LEU A 283 -17.213 11.331 -41.277 1.00 47.70 H
ATOM 3758 HB2 LEU A 283 -19.223 13.177 -39.940 1.00 49.45 H
ATOM 3759 HB3 LEU A 283 -19.393 12.588 -41.567 1.00 49.45 H
ATOM 3760 HG LEU A 283 -19.298 10.780 -39.114 1.00 57.36 H
ATOM 3761 HD11 LEU A 283 -21.556 10.883 -38.957 1.00 50.34 H
ATOM 3762 HD12 LEU A 283 -21.402 12.426 -39.771 1.00 50.34 H
ATOM 3763 HD13 LEU A 283 -21.801 10.994 -40.694 1.00 50.34 H
ATOM 3764 HD21 LEU A 283 -20.398 9.295 -41.115 1.00 60.15 H
ATOM 3765 HD22 LEU A 283 -19.198 10.215 -42.003 1.00 60.15 H
ATOM 3766 HD23 LEU A 283 -18.722 9.245 -40.618 1.00 60.15 H
ATOM 3767 N VAL A 284 -16.524 14.470 -40.648 1.00 43.86 N
ATOM 3768 CA VAL A 284 -15.973 15.667 -41.277 1.00 41.94 C
ATOM 3769 C VAL A 284 -14.565 15.402 -41.803 1.00 40.71 C
ATOM 3770 O VAL A 284 -14.264 15.686 -42.968 1.00 41.57 O
ATOM 3771 CB VAL A 284 -15.998 16.856 -40.300 1.00 47.01 C
ATOM 3772 CG1 VAL A 284 -15.199 18.021 -40.868 1.00 41.31 C
ATOM 3773 CG2 VAL A 284 -17.431 17.288 -40.030 1.00 50.78 C
ATOM 3774 H VAL A 284 -16.671 14.481 -39.648 1.00 43.86 H
ATOM 3775 HA VAL A 284 -16.599 15.930 -42.134 1.00 41.94 H
ATOM 3776 HB VAL A 284 -15.553 16.553 -39.351 1.00 47.01 H
ATOM 3777 HG11 VAL A 284 -15.344 18.917 -40.263 1.00 41.31 H
ATOM 3778 HG12 VAL A 284 -14.127 17.828 -40.865 1.00 41.31 H
ATOM 3779 HG13 VAL A 284 -15.501 18.265 -41.887 1.00 41.31 H
ATOM 3780 HG21 VAL A 284 -17.482 17.955 -39.169 1.00 50.78 H
ATOM 3781 HG22 VAL A 284 -17.841 17.826 -40.885 1.00 50.78 H
ATOM 3782 HG23 VAL A 284 -18.104 16.456 -39.847 1.00 50.78 H
ATOM 3783 N VAL A 285 -13.683 14.852 -40.962 1.00 34.11 N
ATOM 3784 CA VAL A 285 -12.294 14.685 -41.388 1.00 37.45 C
ATOM 3785 C VAL A 285 -12.192 13.673 -42.524 1.00 38.88 C
ATOM 3786 O VAL A 285 -11.316 13.793 -43.390 1.00 37.18 O
ATOM 3787 CB VAL A 285 -11.383 14.300 -40.203 1.00 41.52 C
ATOM 3788 CG1 VAL A 285 -11.556 15.280 -39.048 1.00 47.21 C
ATOM 3789 CG2 VAL A 285 -11.631 12.866 -39.750 1.00 46.57 C
ATOM 3790 H VAL A 285 -13.930 14.605 -40.012 1.00 34.11 H
ATOM 3791 HA VAL A 285 -11.937 15.641 -41.775 1.00 37.45 H
ATOM 3792 HB VAL A 285 -10.349 14.376 -40.537 1.00 41.52 H
ATOM 3793 HG11 VAL A 285 -10.827 15.084 -38.261 1.00 47.21 H
ATOM 3794 HG12 VAL A 285 -11.391 16.299 -39.392 1.00 47.21 H
ATOM 3795 HG13 VAL A 285 -12.533 15.268 -38.592 1.00 47.21 H

ATOM 3796 HG21 VAL A 285 -11.040 12.646 -38.861 1.00 46.57 H
ATOM 3797 HG22 VAL A 285 -12.670 12.708 -39.479 1.00 46.57 H
ATOM 3798 HG23 VAL A 285 -11.342 12.122 -40.492 1.00 46.57 H
ATOM 3799 N VAL A 286 -13.080 12.678 -42.557 1.00 38.31 N
ATOM 3800 CA VAL A 286 -13.059 11.706 -43.646 1.00 41.29 C
ATOM 3801 C VAL A 286 -13.629 12.317 -44.919 1.00 43.62 C
ATOM 3802 O VAL A 286 -13.077 12.133 -46.011 1.00 42.84 O
ATOM 3803 CB VAL A 286 -13.819 10.429 -43.234 1.00 41.63 C
ATOM 3804 CG1 VAL A 286 -13.973 9.487 -44.422 1.00 40.61 C
ATOM 3805 CG2 VAL A 286 -13.095 9.726 -42.092 1.00 37.79 C
ATOM 3806 H VAL A 286 -13.786 12.575 -41.840 1.00 38.31 H
ATOM 3807 HA VAL A 286 -12.025 11.418 -43.853 1.00 41.29 H
ATOM 3808 HB VAL A 286 -14.816 10.702 -42.882 1.00 41.63 H
ATOM 3809 HG11 VAL A 286 -14.409 8.542 -44.100 1.00 40.61 H
ATOM 3810 HG12 VAL A 286 -14.645 9.874 -45.188 1.00 40.61 H
ATOM 3811 HG13 VAL A 286 -13.013 9.263 -44.889 1.00 40.61 H
ATOM 3812 HG21 VAL A 286 -13.662 8.862 -41.745 1.00 37.79 H
ATOM 3813 HG22 VAL A 286 -12.116 9.367 -42.406 1.00 37.79 H
ATOM 3814 HG23 VAL A 286 -12.938 10.371 -41.230 1.00 37.79 H
ATOM 3815 N ALA A 287 -14.736 13.055 -44.803 1.00 37.74 N
ATOM 3816 CA ALA A 287 -15.328 13.687 -45.976 1.00 41.01 C
ATOM 3817 C ALA A 287 -14.393 14.731 -46.574 1.00 38.64 C
ATOM 3818 O ALA A 287 -14.294 14.851 -47.800 1.00 42.37 O
ATOM 3819 CB ALA A 287 -16.674 14.316 -45.617 1.00 38.91 C
ATOM 3820 H ALA A 287 -15.190 13.198 -43.910 1.00 37.74 H
ATOM 3821 HA ALA A 287 -15.509 12.920 -46.732 1.00 41.01 H
ATOM 3822 HB1 ALA A 287 -17.127 14.801 -46.483 1.00 38.91 H
ATOM 3823 HB2 ALA A 287 -17.377 13.560 -45.265 1.00 38.91 H
ATOM 3824 HB3 ALA A 287 -16.571 15.067 -44.832 1.00 38.91 H
ATOM 3825 N VAL A 288 -13.700 15.495 -45.727 1.00 37.96 N
ATOM 3826 CA VAL A 288 -12.763 16.495 -46.233 1.00 41.92 C
ATOM 3827 C VAL A 288 -11.639 15.823 -47.013 1.00 41.35 C
ATOM 3828 O VAL A 288 -11.283 16.255 -48.116 1.00 37.23 O
ATOM 3829 CB VAL A 288 -12.218 17.358 -45.082 1.00 40.95 C
ATOM 3830 CG1 VAL A 288 -10.996 18.137 -45.542 1.00 42.10 C
ATOM 3831 CG2 VAL A 288 -13.294 18.311 -44.587 1.00 39.17 C
ATOM 3832 H VAL A 288 -13.810 15.409 -44.723 1.00 37.96 H
ATOM 3833 HA VAL A 288 -13.300 17.146 -46.927 1.00 41.92 H
ATOM 3834 HB VAL A 288 -11.927 16.713 -44.251 1.00 40.95 H
ATOM 3835 HG11 VAL A 288 -10.737 18.908 -44.817 1.00 42.10 H

ATOM 3836 HG12 VAL A 288 -10.109 17.511 -45.637 1.00 42.10 H
ATOM 3837 HG13 VAL A 288 -11.169 18.645 -46.492 1.00 42.10 H
ATOM 3838 HG21 VAL A 288 -12.961 18.852 -43.701 1.00 39.17 H
ATOM 3839 HG22 VAL A 288 -13.543 19.052 -45.348 1.00 39.17 H
ATOM 3840 HG23 VAL A 288 -14.219 17.800 -44.327 1.00 39.17 H
ATOM 3841 N PHE A 289 -11.069 14.749 -46.458 1.00 36.73 N
ATOM 3842 CA PHE A 289 -10.011 14.038 -47.169 1.00 38.18 C
ATOM 3843 C PHE A 289 -10.509 13.517 -48.511 1.00 37.36 C
ATOM 3844 O PHE A 289 -9.813 13.628 -49.528 1.00 36.96 O
ATOM 3845 CB PHE A 289 -9.472 12.885 -46.319 1.00 36.26 C
ATOM 3846 CG PHE A 289 -8.263 12.210 -46.916 1.00 35.27 C
ATOM 3847 CD1 PHE A 289 -8.405 11.201 -47.859 1.00 39.24 C
ATOM 3848 CD2 PHE A 289 -6.986 12.594 -46.544 1.00 33.71 C
ATOM 3849 CE1 PHE A 289 -7.297 10.590 -48.416 1.00 34.16 C
ATOM 3850 CE2 PHE A 289 -5.872 11.984 -47.098 1.00 43.23 C
ATOM 3851 CZ PHE A 289 -6.030 10.981 -48.037 1.00 33.37 C
ATOM 3852 H PHE A 289 -11.362 14.397 -45.556 1.00 36.73 H
ATOM 3853 HA PHE A 289 -9.194 14.736 -47.357 1.00 38.18 H
ATOM 3854 HB2 PHE A 289 -9.200 13.261 -45.332 1.00 36.26 H
ATOM 3855 HB3 PHE A 289 -10.249 12.139 -46.143 1.00 36.26 H
ATOM 3856 HD1 PHE A 289 -9.390 10.880 -48.166 1.00 39.24 H
ATOM 3857 HD2 PHE A 289 -6.849 13.380 -45.816 1.00 33.71 H
ATOM 3858 HE1 PHE A 289 -7.417 9.815 -49.158 1.00 34.16 H
ATOM 3859 HE2 PHE A 289 -4.890 12.282 -46.778 1.00 43.23 H
ATOM 3860 HZ PHE A 289 -5.162 10.504 -48.467 1.00 33.37 H
ATOM 3861 N ILE A 290 -11.718 12.954 -48.535 1.00 36.80 N
ATOM 3862 CA ILE A 290 -12.242 12.362 -49.760 1.00 40.36 C
ATOM 3863 C ILE A 290 -12.568 13.441 -50.787 1.00 43.77 C
ATOM 3864 O ILE A 290 -12.258 13.296 -51.974 1.00 42.23 O
ATOM 3865 CB ILE A 290 -13.466 11.485 -49.440 1.00 40.87 C
ATOM 3866 CG1 ILE A 290 -13.027 10.233 -48.680 1.00 45.01 C
ATOM 3867 CG2 ILE A 290 -14.216 11.112 -50.713 1.00 40.33 C
ATOM 3868 CD1 ILE A 290 -14.174 9.325 -48.289 1.00 51.52 C
ATOM 3869 H ILE A 290 -12.275 12.869 -47.694 1.00 36.80 H
ATOM 3870 HA ILE A 290 -11.470 11.721 -50.194 1.00 40.36 H
ATOM 3871 HB ILE A 290 -14.146 12.052 -48.801 1.00 40.87 H
ATOM 3872 HG12 ILE A 290 -12.338 9.661 -49.304 1.00 45.01 H
ATOM 3873 HG13 ILE A 290 -12.454 10.488 -47.790 1.00 45.01 H
ATOM 3874 HG21 ILE A 290 -15.087 10.499 -50.483 1.00 40.33 H
ATOM 3875 HG22 ILE A 290 -14.624 11.961 -51.262 1.00 40.33 H

```
ATOM   3876 HG23 ILE A 290     -13.597  10.522 -51.385  1.00 40.33           H
ATOM   3877 HD11 ILE A 290     -13.891   8.692 -47.448  1.00 51.52           H
ATOM   3878 HD12 ILE A 290     -15.054   9.894 -47.988  1.00 51.52           H
ATOM   3879 HD13 ILE A 290     -14.458   8.665 -49.109  1.00 51.52           H
ATOM   3880  N   VAL A 291     -13.188  14.540 -50.352  1.00 37.21           N
ATOM   3881  CA  VAL A 291     -13.561  15.599 -51.287  1.00 44.30           C
ATOM   3882  C   VAL A 291     -12.322  16.260 -51.880  1.00 45.60           C
ATOM   3883  O   VAL A 291     -12.290  16.599 -53.070  1.00 42.97           O
ATOM   3884  CB  VAL A 291     -14.476  16.625 -50.590  1.00 45.98           C
ATOM   3885  CG1 VAL A 291     -14.599  17.892 -51.424  1.00 42.39           C
ATOM   3886  CG2 VAL A 291     -15.851  16.022 -50.334  1.00 45.71           C
ATOM   3887  H   VAL A 291     -13.451  14.652 -49.380  1.00 37.21           H
ATOM   3888  HA  VAL A 291     -14.119  15.150 -52.112  1.00 44.30           H
ATOM   3889  HB  VAL A 291     -14.042  16.896 -49.626  1.00 45.98           H
ATOM   3890 HG11 VAL A 291     -15.377  18.540 -51.018  1.00 42.39           H
ATOM   3891 HG12 VAL A 291     -13.687  18.491 -51.426  1.00 42.39           H
ATOM   3892 HG13 VAL A 291     -14.872  17.674 -52.457  1.00 42.39           H
ATOM   3893 HG21 VAL A 291     -16.467  16.694 -49.737  1.00 45.71           H
ATOM   3894 HG22 VAL A 291     -16.379  15.838 -51.270  1.00 45.71           H
ATOM   3895 HG23 VAL A 291     -15.801  15.071 -49.806  1.00 45.71           H
ATOM   3896  N   CYS A 292     -11.276  16.438 -51.070  1.00 42.73           N
ATOM   3897  CA  CYS A 292     -10.102  17.178 -51.520  1.00 43.85           C
ATOM   3898  C   CYS A 292      -9.166  16.339 -52.383  1.00 43.09           C
ATOM   3899  O   CYS A 292      -8.506  16.886 -53.275  1.00 38.94           O
ATOM   3900  CB  CYS A 292      -9.333  17.730 -50.318  1.00 40.86           C
ATOM   3901  SG  CYS A 292     -10.142  19.125 -49.480  1.00 45.63           S
ATOM   3902  H   CYS A 292     -11.310  16.152 -50.100  1.00 42.73           H
ATOM   3903  HA  CYS A 292     -10.417  18.035 -52.120  1.00 43.85           H
ATOM   3904  HB2 CYS A 292      -9.153  16.940 -49.587  1.00 40.86           H
ATOM   3905  HB3 CYS A 292      -8.349  18.070 -50.626  1.00 40.86           H
ATOM   3906  HG  CYS A 292     -11.159  18.413 -48.987  1.00 45.63           H
ATOM   3907  N   TRP A 293      -9.086  15.030 -52.141  1.00 36.06           N
ATOM   3908  CA  TRP A 293      -8.083  14.191 -52.785  1.00 38.31           C
ATOM   3909  C   TRP A 293      -8.622  13.276 -53.875  1.00 40.47           C
ATOM   3910  O   TRP A 293      -7.863  12.913 -54.777  1.00 39.22           O
ATOM   3911  CB  TRP A 293      -7.316  13.331 -51.761  1.00 31.17           C
ATOM   3912  CG  TRP A 293      -6.349  14.054 -50.880  1.00 35.04           C
ATOM   3913  CD1 TRP A 293      -6.491  14.249 -49.552  1.00 32.70           C
ATOM   3914  CD2 TRP A 293      -5.090  14.701 -51.247  1.00 37.45           C
ATOM   3915  CE2 TRP A 293      -4.499  15.233 -50.062  1.00 37.51           C
```

```
ATOM   3916  CE3 TRP A 293      -4.382  14.897 -52.458  1.00 38.20           C
ATOM   3917  NE1 TRP A 293      -5.387  14.914 -49.065  1.00 36.60           N
ATOM   3918  CZ2 TRP A 293      -3.276  15.920 -50.070  1.00 42.29           C
ATOM   3919  CZ3 TRP A 293      -3.155  15.594 -52.483  1.00 39.60           C
ATOM   3920  CH2 TRP A 293      -2.607  16.107 -51.290  1.00 44.36           C
ATOM   3921  H   TRP A 293      -9.634  14.610 -51.402  1.00 36.06           H
ATOM   3922  HA  TRP A 293      -7.338  14.794 -53.303  1.00 38.31           H
ATOM   3923  HB2 TRP A 293      -8.008  12.759 -51.142  1.00 31.17           H
ATOM   3924  HB3 TRP A 293      -6.716  12.581 -52.278  1.00 31.17           H
ATOM   3925  HD1 TRP A 293      -7.328  13.894 -48.972  1.00 32.70           H
ATOM   3926  HE1 TRP A 293      -5.272  15.175 -48.083  1.00 36.60           H
ATOM   3927  HE3 TRP A 293      -4.795  14.518 -53.380  1.00 38.20           H
ATOM   3928  HZ2 TRP A 293      -2.868  16.317 -49.152  1.00 42.29           H
ATOM   3929  HZ3 TRP A 293      -2.638  15.743 -53.420  1.00 39.60           H
ATOM   3930  HH2 TRP A 293      -1.684  16.662 -51.295  1.00 44.36           H
ATOM   3931  N   THR A 294      -9.894  12.878 -53.818  1.00 35.53           N
ATOM   3932  CA  THR A 294     -10.429  11.995 -54.855  1.00 38.74           C
ATOM   3933  C   THR A 294     -10.381  12.610 -56.250  1.00 43.28           C
ATOM   3934  O   THR A 294      -9.973  11.905 -57.190  1.00 37.41           O
ATOM   3935  CB  THR A 294     -11.859  11.566 -54.504  1.00 39.95           C
ATOM   3936  CG2 THR A 294     -12.339  10.483 -55.462  1.00 39.76           C
ATOM   3937  OG1 THR A 294     -11.900  11.060 -53.165  1.00 42.89           O
ATOM   3938  H   THR A 294     -10.517  13.183 -53.083  1.00 35.53           H
ATOM   3939  HA  THR A 294      -9.805  11.098 -54.870  1.00 38.74           H
ATOM   3940  HB  THR A 294     -12.559  12.398 -54.553  1.00 39.95           H
ATOM   3941  HG1 THR A 294     -11.919  11.817 -52.568  1.00 42.89           H
ATOM   3942 HG21 THR A 294     -13.298  10.080 -55.135  1.00 39.76           H
ATOM   3943 HG22 THR A 294     -12.489  10.861 -56.473  1.00 39.76           H
ATOM   3944 HG23 THR A 294     -11.637   9.650 -55.512  1.00 39.76           H
ATOM   3945  N   PRO A 295     -10.776  13.874 -56.470  1.00 38.48           N
ATOM   3946  CA  PRO A 295     -10.812  14.378 -57.857  1.00 41.16           C
ATOM   3947  C   PRO A 295      -9.478  14.299 -58.581  1.00 40.37           C
ATOM   3948  O   PRO A 295      -9.428  13.789 -59.706  1.00 37.29           O
ATOM   3949  CB  PRO A 295     -11.291  15.826 -57.684  1.00 34.57           C
ATOM   3950  CG  PRO A 295     -12.081  15.805 -56.429  1.00 39.83           C
ATOM   3951  CD  PRO A 295     -11.336  14.865 -55.531  1.00 37.19           C
ATOM   3952  HA  PRO A 295     -11.566  13.809 -58.406  1.00 41.16           H
ATOM   3953  HB2 PRO A 295     -10.456  16.520 -57.572  1.00 34.57           H
ATOM   3954  HB3 PRO A 295     -11.896  16.162 -58.525  1.00 34.57           H
ATOM   3955  HG2 PRO A 295     -12.227  16.789 -55.993  1.00 39.83           H
```

ATOM 3956 HG3 PRO A 295 -13.070 15.392 -56.636 1.00 39.83 H
ATOM 3957 HD2 PRO A 295 -10.513 15.386 -55.039 1.00 37.19 H
ATOM 3958 HD3 PRO A 295 -11.988 14.455 -54.763 1.00 37.19 H
ATOM 3959 N ILE A 296 -8.389 14.780 -57.974 1.00 33.55 N
ATOM 3960 CA ILE A 296 -7.124 14.784 -58.702 1.00 32.57 C
ATOM 3961 C ILE A 296 -6.616 13.361 -58.916 1.00 45.92 C
ATOM 3962 O ILE A 296 -6.043 13.054 -59.969 1.00 40.89 O
ATOM 3963 CB ILE A 296 -6.042 15.632 -57.967 1.00 33.96 C
ATOM 3964 CG1 ILE A 296 -4.758 15.853 -58.795 1.00 35.80 C
ATOM 3965 CG2 ILE A 296 -5.680 15.134 -56.549 1.00 39.02 C
ATOM 3966 CD1 ILE A 296 -5.005 16.523 -60.146 1.00 35.41 C
ATOM 3967 H ILE A 296 -8.420 15.191 -57.052 1.00 33.55 H
ATOM 3968 HA ILE A 296 -7.333 15.207 -59.685 1.00 32.57 H
ATOM 3969 HB ILE A 296 -6.479 16.615 -57.829 1.00 33.96 H
ATOM 3970 HG12 ILE A 296 -4.061 16.473 -58.231 1.00 35.80 H
ATOM 3971 HG13 ILE A 296 -4.250 14.905 -58.971 1.00 35.80 H
ATOM 3972 HG21 ILE A 296 -5.022 15.845 -56.047 1.00 39.02 H
ATOM 3973 HG22 ILE A 296 -6.564 15.029 -55.921 1.00 39.02 H
ATOM 3974 HG23 ILE A 296 -5.161 14.174 -56.565 1.00 39.02 H
ATOM 3975 HD11 ILE A 296 -4.065 16.855 -60.588 1.00 35.41 H
ATOM 3976 HD12 ILE A 296 -5.465 15.824 -60.840 1.00 35.41 H
ATOM 3977 HD13 ILE A 296 -5.662 17.387 -60.058 1.00 35.41 H
ATOM 3978 N HIS A 297 -6.844 12.460 -57.956 1.00 35.20 N
ATOM 3979 CA HIS A 297 -6.381 11.086 -58.130 1.00 37.71 C
ATOM 3980 C HIS A 297 -7.130 10.381 -59.252 1.00 38.29 C
ATOM 3981 O HIS A 297 -6.526 9.644 -60.039 1.00 40.38 O
ATOM 3982 CB HIS A 297 -6.483 10.290 -56.818 1.00 31.05 C
ATOM 3983 CG HIS A 297 -5.400 10.601 -55.816 1.00 37.83 C
ATOM 3984 CD2 HIS A 297 -4.128 10.102 -55.657 1.00 38.14 C
ATOM 3985 ND1 HIS A 297 -5.564 11.507 -54.787 1.00 39.40 N
ATOM 3986 CE1 HIS A 297 -4.445 11.512 -54.060 1.00 43.11 C
ATOM 3987 NE2 HIS A 297 -3.527 10.687 -54.543 1.00 42.96 N
ATOM 3988 H HIS A 297 -7.328 12.700 -57.102 1.00 35.20 H
ATOM 3989 HA HIS A 297 -5.329 11.096 -58.426 1.00 37.71 H
ATOM 3990 HB2 HIS A 297 -7.461 10.433 -56.355 1.00 31.05 H
ATOM 3991 HB3 HIS A 297 -6.418 9.227 -57.039 1.00 31.05 H
ATOM 3992 HD1 HIS A 297 -6.409 12.064 -54.624 1.00 39.40 H
ATOM 3993 HD2 HIS A 297 -3.594 9.380 -56.255 1.00 38.14 H
ATOM 3994 HE1 HIS A 297 -4.295 12.113 -53.173 1.00 43.11 H
ATOM 3995 N ILE A 298 -8.443 10.593 -59.346 1.00 37.36 N

```
ATOM   3996  CA  ILE A 298      -9.211   9.972 -60.419  1.00 40.86           C
ATOM   3997  C   ILE A 298      -8.865  10.613 -61.757  1.00 43.37           C
ATOM   3998  O   ILE A 298      -8.766   9.928 -62.782  1.00 46.82           O
ATOM   3999  CB  ILE A 298     -10.737  10.045 -60.118  1.00 46.71           C
ATOM   4000  CG1 ILE A 298     -11.105   9.195 -58.874  1.00 49.51           C
ATOM   4001  CG2 ILE A 298     -11.661   9.697 -61.303  1.00 41.76           C
ATOM   4002  CD1 ILE A 298     -10.785   7.695 -58.980  1.00 48.01           C
ATOM   4003  H   ILE A 298      -8.935  11.187 -58.690  1.00 37.36           H
ATOM   4004  HA  ILE A 298      -8.924   8.922 -60.507  1.00 40.86           H
ATOM   4005  HB  ILE A 298     -10.964  11.080 -59.854  1.00 46.71           H
ATOM   4006 HG12 ILE A 298     -10.577   9.579 -58.002  1.00 49.51           H
ATOM   4007 HG13 ILE A 298     -12.163   9.325 -58.641  1.00 49.51           H
ATOM   4008 HG21 ILE A 298     -12.706   9.667 -60.995  1.00 41.76           H
ATOM   4009 HG22 ILE A 298     -11.592  10.445 -62.094  1.00 41.76           H
ATOM   4010 HG23 ILE A 298     -11.414   8.730 -61.741  1.00 41.76           H
ATOM   4011 HD11 ILE A 298     -11.372   7.125 -58.260  1.00 48.01           H
ATOM   4012 HD12 ILE A 298     -11.002   7.292 -59.969  1.00 48.01           H
ATOM   4013 HD13 ILE A 298      -9.734   7.501 -58.764  1.00 48.01           H
ATOM   4014  N   TYR A 299      -8.663  11.933 -61.765  1.00 39.57           N
ATOM   4015  CA  TYR A 299      -8.295  12.629 -62.994  1.00 44.17           C
ATOM   4016  C   TYR A 299      -6.982  12.097 -63.557  1.00 43.45           C
ATOM   4017  O   TYR A 299      -6.859  11.879 -64.769  1.00 45.05           O
ATOM   4018  CB  TYR A 299      -8.197  14.130 -62.721  1.00 46.44           C
ATOM   4019  CG  TYR A 299      -8.604  15.017 -63.877  1.00 47.76           C
ATOM   4020  CD1 TYR A 299      -8.254  14.704 -65.182  1.00 52.71           C
ATOM   4021  CD2 TYR A 299      -9.340  16.173 -63.655  1.00 50.45           C
ATOM   4022  CE1 TYR A 299      -8.625  15.520 -66.235  1.00 60.66           C
ATOM   4023  CE2 TYR A 299      -9.718  16.991 -64.698  1.00 52.74           C
ATOM   4024  CZ  TYR A 299      -9.357  16.662 -65.985  1.00 58.62           C
ATOM   4025  OH  TYR A 299      -9.734  17.481 -67.021  1.00 65.76           O
ATOM   4026  H   TYR A 299      -8.769  12.486 -60.923  1.00 39.57           H
ATOM   4027  HA  TYR A 299      -9.085  12.445 -63.725  1.00 44.17           H
ATOM   4028  HB2 TYR A 299      -8.907  14.388 -61.943  1.00 46.44           H
ATOM   4029  HB3 TYR A 299      -7.221  14.424 -62.334  1.00 46.44           H
ATOM   4030  HD1 TYR A 299      -7.700  13.837 -65.432  1.00 52.71           H
ATOM   4031  HD2 TYR A 299      -9.623  16.441 -62.651  1.00 50.45           H
ATOM   4032  HE1 TYR A 299      -8.343  15.262 -67.243  1.00 60.66           H
ATOM   4033  HE2 TYR A 299     -10.295  17.880 -64.496  1.00 52.74           H
ATOM   4034  HH  TYR A 299     -10.211  18.262 -66.723  1.00 65.76           H
ATOM   4035  N   VAL A 300      -5.992  11.875 -62.689  1.00 39.62           N
```

ATOM 4036 CA VAL A 300 -4.701 11.364 -63.143 1.00 41.83 C
ATOM 4037 C VAL A 300 -4.859 9.974 -63.750 1.00 41.73 C
ATOM 4038 O VAL A 300 -4.248 9.655 -64.779 1.00 39.04 O
ATOM 4039 CB VAL A 300 -3.619 11.372 -62.027 1.00 39.45 C
ATOM 4040 CG1 VAL A 300 -2.349 10.558 -62.363 1.00 45.05 C
ATOM 4041 CG2 VAL A 300 -3.201 12.818 -61.706 1.00 33.69 C
ATOM 4042 H VAL A 300 -6.105 12.067 -61.701 1.00 39.62 H
ATOM 4043 HA VAL A 300 -4.330 12.025 -63.930 1.00 41.83 H
ATOM 4044 HB VAL A 300 -4.053 10.950 -61.118 1.00 39.45 H
ATOM 4045 HG11 VAL A 300 -1.535 10.784 -61.676 1.00 45.05 H
ATOM 4046 HG12 VAL A 300 -2.523 9.483 -62.304 1.00 45.05 H
ATOM 4047 HG13 VAL A 300 -1.983 10.786 -63.365 1.00 45.05 H
ATOM 4048 HG21 VAL A 300 -2.518 12.853 -60.859 1.00 33.69 H
ATOM 4049 HG22 VAL A 300 -2.682 13.265 -62.552 1.00 33.69 H
ATOM 4050 HG23 VAL A 300 -4.048 13.455 -61.460 1.00 33.69 H
ATOM 4051 N ILE A 301 -5.694 9.131 -63.137 1.00 40.23 N
ATOM 4052 CA ILE A 301 -5.902 7.782 -63.657 1.00 44.41 C
ATOM 4053 C ILE A 301 -6.592 7.829 -65.015 1.00 45.55 C
ATOM 4054 O ILE A 301 -6.216 7.102 -65.943 1.00 50.58 O
ATOM 4055 CB ILE A 301 -6.689 6.898 -62.640 1.00 44.34 C
ATOM 4056 CG1 ILE A 301 -5.861 6.628 -61.356 1.00 41.87 C
ATOM 4057 CG2 ILE A 301 -7.266 5.579 -63.202 1.00 44.72 C
ATOM 4058 CD1 ILE A 301 -4.662 5.678 -61.521 1.00 54.72 C
ATOM 4059 H ILE A 301 -6.193 9.398 -62.299 1.00 40.23 H
ATOM 4060 HA ILE A 301 -4.928 7.321 -63.832 1.00 44.41 H
ATOM 4061 HB ILE A 301 -7.552 7.483 -62.316 1.00 44.34 H
ATOM 4062 HG12 ILE A 301 -5.484 7.566 -60.952 1.00 41.87 H
ATOM 4063 HG13 ILE A 301 -6.519 6.233 -60.581 1.00 41.87 H
ATOM 4064 HG21 ILE A 301 -7.716 4.980 -62.410 1.00 44.72 H
ATOM 4065 HG22 ILE A 301 -8.047 5.758 -63.941 1.00 44.72 H
ATOM 4066 HG23 ILE A 301 -6.494 4.975 -63.680 1.00 44.72 H
ATOM 4067 HD11 ILE A 301 -4.174 5.513 -60.560 1.00 54.72 H
ATOM 4068 HD12 ILE A 301 -4.964 4.702 -61.899 1.00 54.72 H
ATOM 4069 HD13 ILE A 301 -3.909 6.087 -62.195 1.00 54.72 H
ATOM 4070 N ILE A 302 -7.611 8.680 -65.154 1.00 45.67 N
ATOM 4071 CA ILE A 302 -8.360 8.740 -66.406 1.00 48.98 C
ATOM 4072 C ILE A 302 -7.469 9.226 -67.541 1.00 51.01 C
ATOM 4073 O ILE A 302 -7.524 8.698 -68.658 1.00 50.19 O
ATOM 4074 CB ILE A 302 -9.609 9.624 -66.240 1.00 48.51 C
ATOM 4075 CG1 ILE A 302 -10.564 8.993 -65.226 1.00 55.47 C

ATOM 4076 CG2 ILE A 302 -10.313 9.817 -67.575 1.00 48.96 C
ATOM 4077 CD1 ILE A 302 -11.818 9.790 -64.998 1.00 50.89 C
ATOM 4078 H ILE A 302 -7.922 9.261 -64.385 1.00 45.67 H
ATOM 4079 HA ILE A 302 -8.688 7.728 -66.657 1.00 48.98 H
ATOM 4080 HB ILE A 302 -9.305 10.602 -65.863 1.00 48.51 H
ATOM 4081 HG12 ILE A 302 -10.869 8.020 -65.615 1.00 55.47 H
ATOM 4082 HG13 ILE A 302 -10.099 8.750 -64.280 1.00 55.47 H
ATOM 4083 HG21 ILE A 302 -11.216 10.417 -67.480 1.00 48.96 H
ATOM 4084 HG22 ILE A 302 -9.708 10.358 -68.303 1.00 48.96 H
ATOM 4085 HG23 ILE A 302 -10.597 8.863 -68.020 1.00 48.96 H
ATOM 4086 HD11 ILE A 302 -12.337 9.439 -64.107 1.00 50.89 H
ATOM 4087 HD12 ILE A 302 -11.602 10.850 -64.862 1.00 50.89 H
ATOM 4088 HD13 ILE A 302 -12.524 9.684 -65.822 1.00 50.89 H
ATOM 4089 N LYS A 303 -6.624 10.226 -67.279 1.00 50.46 N
ATOM 4090 CA LYS A 303 -5.708 10.689 -68.315 1.00 54.03 C
ATOM 4091 C LYS A 303 -4.666 9.640 -68.677 1.00 50.57 C
ATOM 4092 O LYS A 303 -4.126 9.681 -69.789 1.00 51.07 O
ATOM 4093 CB LYS A 303 -5.031 12.003 -67.873 1.00 51.95 C
ATOM 4094 CG LYS A 303 -5.941 13.232 -68.055 1.00 61.59 C
ATOM 4095 CD LYS A 303 -5.240 14.600 -67.924 1.00 75.40 C
ATOM 4096 CE LYS A 303 -4.709 14.949 -66.525 1.00 81.93 C
ATOM 4097 NZ LYS A 303 -3.465 14.220 -66.184 1.00 87.87 N1+
ATOM 4098 H LYS A 303 -6.586 10.665 -66.368 1.00 50.46 H
ATOM 4099 HA LYS A 303 -6.282 10.897 -69.221 1.00 54.03 H
ATOM 4100 HB2 LYS A 303 -4.674 11.910 -66.847 1.00 51.95 H
ATOM 4101 HB3 LYS A 303 -4.143 12.164 -68.486 1.00 51.95 H
ATOM 4102 HG2 LYS A 303 -6.391 13.188 -69.048 1.00 61.59 H
ATOM 4103 HG3 LYS A 303 -6.776 13.169 -67.359 1.00 61.59 H
ATOM 4104 HD2 LYS A 303 -4.428 14.667 -68.649 1.00 75.40 H
ATOM 4105 HD3 LYS A 303 -5.948 15.376 -68.219 1.00 75.40 H
ATOM 4106 HE2 LYS A 303 -4.483 16.015 -66.480 1.00 81.93 H
ATOM 4107 HE3 LYS A 303 -5.466 14.750 -65.768 1.00 81.93 H
ATOM 4108 HZ1 LYS A 303 -2.750 14.430 -66.865 1.00 87.87 H
ATOM 4109 HZ2 LYS A 303 -3.149 14.511 -65.270 1.00 87.87 H
ATOM 4110 HZ3 LYS A 303 -3.645 13.226 -66.177 1.00 87.87 H
ATOM 4111 N ALA A 304 -4.367 8.704 -67.774 1.00 46.29 N
ATOM 4112 CA ALA A 304 -3.456 7.624 -68.129 1.00 53.13 C
ATOM 4113 C ALA A 304 -4.120 6.590 -69.029 1.00 55.64 C
ATOM 4114 O ALA A 304 -3.423 5.897 -69.778 1.00 57.36 O
ATOM 4115 CB ALA A 304 -2.911 6.951 -66.871 1.00 47.34 C

ATOM 4116 H ALA A 304 -4.812 8.676 -66.866 1.00 46.29 H
ATOM 4117 HA ALA A 304 -2.596 8.033 -68.664 1.00 53.13 H
ATOM 4118 HB1 ALA A 304 -2.152 6.207 -67.117 1.00 47.34 H
ATOM 4119 HB2 ALA A 304 -2.449 7.681 -66.206 1.00 47.34 H
ATOM 4120 HB3 ALA A 304 -3.695 6.445 -66.307 1.00 47.34 H
ATOM 4121 N LEU A 305 -5.447 6.489 -68.993 1.00 55.18 N
ATOM 4122 CA LEU A 305 -6.178 5.465 -69.728 1.00 55.74 C
ATOM 4123 C LEU A 305 -6.718 5.945 -71.067 1.00 64.03 C
ATOM 4124 O LEU A 305 -6.627 5.215 -72.057 1.00 65.97 O
ATOM 4125 CB LEU A 305 -7.339 4.937 -68.880 1.00 49.70 C
ATOM 4126 CG LEU A 305 -6.953 4.135 -67.637 1.00 54.60 C
ATOM 4127 CD1 LEU A 305 -8.169 3.896 -66.759 1.00 50.28 C
ATOM 4128 CD2 LEU A 305 -6.312 2.813 -68.041 1.00 56.37 C
ATOM 4129 H LEU A 305 -5.986 7.091 -68.386 1.00 55.18 H
ATOM 4130 HA LEU A 305 -5.524 4.619 -69.945 1.00 55.74 H
ATOM 4131 HB2 LEU A 305 -7.944 5.794 -68.576 1.00 49.70 H
ATOM 4132 HB3 LEU A 305 -8.003 4.322 -69.492 1.00 49.70 H
ATOM 4133 HG LEU A 305 -6.233 4.709 -67.054 1.00 54.60 H
ATOM 4134 HD11 LEU A 305 -7.872 3.781 -65.716 1.00 50.28 H
ATOM 4135 HD12 LEU A 305 -8.874 4.727 -66.798 1.00 50.28 H
ATOM 4136 HD13 LEU A 305 -8.715 2.997 -67.048 1.00 50.28 H
ATOM 4137 HD21 LEU A 305 -6.698 1.970 -67.467 1.00 56.37 H
ATOM 4138 HD22 LEU A 305 -6.462 2.575 -69.095 1.00 56.37 H
ATOM 4139 HD23 LEU A 305 -5.236 2.845 -67.868 1.00 56.37 H
ATOM 4140 N VAL A 306 -7.290 7.150 -71.124 1.00 58.11 N
ATOM 4141 CA VAL A 306 -7.920 7.655 -72.335 1.00 67.25 C
ATOM 4142 C VAL A 306 -7.352 9.028 -72.669 1.00 72.12 C
ATOM 4143 O VAL A 306 -6.737 9.696 -71.836 1.00 72.87 O
ATOM 4144 CB VAL A 306 -9.452 7.736 -72.206 1.00 0.00 C
ATOM 4145 CG1 VAL A 306 -9.997 8.652 -73.304 1.00 0.00 C
ATOM 4146 CG2 VAL A 306 -10.120 6.359 -72.332 1.00 0.00 C
ATOM 4147 H VAL A 306 -7.330 7.744 -70.305 1.00 58.11 H
ATOM 4148 HA VAL A 306 -7.689 7.027 -73.198 1.00 67.25 H
ATOM 4149 HB VAL A 306 -9.719 8.156 -71.235 1.00 0.00 H
ATOM 4150 HG11 VAL A 306 -11.082 8.542 -73.319 1.00 0.00 H
ATOM 4151 HG12 VAL A 306 -9.891 9.709 -73.068 1.00 0.00 H
ATOM 4152 HG13 VAL A 306 -9.707 8.383 -74.320 1.00 0.00 H
ATOM 4153 HG21 VAL A 306 -11.203 6.431 -72.230 1.00 0.00 H
ATOM 4154 HG22 VAL A 306 -9.905 5.898 -73.297 1.00 0.00 H
ATOM 4155 HG23 VAL A 306 -9.766 5.680 -71.556 1.00 0.00 H

```
ATOM   4156  N   THR A 307      -7.567   9.438 -73.918  1.00 64.55           N
ATOM   4157  CA  THR A 307      -7.230  10.774 -74.393  1.00 70.31           C
ATOM   4158  C   THR A 307      -8.504  11.612 -74.415  1.00 81.35           C
ATOM   4159  O   THR A 307      -9.489  11.228 -75.052  1.00 93.09           O
ATOM   4160  CB  THR A 307      -6.599  10.718 -75.786  1.00 84.71           C
ATOM   4161  CG2 THR A 307      -6.506  12.111 -76.394  1.00 83.65           C
ATOM   4162  OG1 THR A 307      -5.287  10.145 -75.702  1.00 93.33           O
ATOM   4163  H   THR A 307      -8.079   8.851 -74.560  1.00 64.55           H
ATOM   4164  HA  THR A 307      -6.511  11.254 -73.725  1.00 70.31           H
ATOM   4165  HB  THR A 307      -7.192  10.082 -76.446  1.00 84.71           H
ATOM   4166  HG1 THR A 307      -5.368   9.245 -75.376  1.00 93.33           H
ATOM   4167 HG21 THR A 307      -5.890  12.091 -77.294  1.00 83.65           H
ATOM   4168 HG22 THR A 307      -7.474  12.508 -76.702  1.00 83.65           H
ATOM   4169 HG23 THR A 307      -6.046  12.823 -75.707  1.00 83.65           H
ATOM   4170  N   ILE A 308      -8.485  12.747 -73.722  1.00 82.28           N
ATOM   4171  CA  ILE A 308      -9.685  13.563 -73.538  1.00 92.90           C
ATOM   4172  C   ILE A 308      -9.582  14.841 -74.365  1.00100.78           C
ATOM   4173  O   ILE A 308      -8.471  15.349 -74.575  1.00100.35           O
ATOM   4174  CB  ILE A 308      -9.914  13.868 -72.048  1.00 95.81           C
ATOM   4175  CG1 ILE A 308      -8.669  14.496 -71.420  1.00 95.56           C
ATOM   4176  CG2 ILE A 308     -10.292  12.599 -71.299  1.00 94.07           C
ATOM   4177  CD1 ILE A 308      -8.806  14.752 -69.929  1.00 93.49           C
ATOM   4178  H   ILE A 308      -7.650  13.048 -73.241  1.00 82.28           H
ATOM   4179  HA  ILE A 308     -10.562  13.014 -73.879  1.00 92.90           H
ATOM   4180  HB  ILE A 308     -10.740  14.574 -71.951  1.00 95.81           H
ATOM   4181 HG12 ILE A 308      -7.824  13.815 -71.520  1.00 95.56           H
ATOM   4182 HG13 ILE A 308      -8.349  15.389 -71.940  1.00 95.56           H
ATOM   4183 HG21 ILE A 308     -10.613  12.805 -70.278  1.00 94.07           H
ATOM   4184 HG22 ILE A 308     -11.126  12.092 -71.785  1.00 94.07           H
ATOM   4185 HG23 ILE A 308      -9.464  11.890 -71.252  1.00 94.07           H
ATOM   4186 HD11 ILE A 308      -8.018  15.420 -69.583  1.00 93.49           H
ATOM   4187 HD12 ILE A 308      -9.761  15.223 -69.692  1.00 93.49           H
ATOM   4188 HD13 ILE A 308      -8.734  13.832 -69.350  1.00 93.49           H
ATOM   4189  N   PRO A 309     -10.697  15.388 -74.858  1.00107.03           N
ATOM   4190  CA  PRO A 309     -10.630  16.560 -75.750  1.00112.90           C
ATOM   4191  C   PRO A 309     -10.254  17.823 -74.990  1.00108.59           C
ATOM   4192  O   PRO A 309     -10.976  18.266 -74.095  1.00108.73           O
ATOM   4193  CB  PRO A 309     -12.049  16.648 -76.323  1.00115.55           C
ATOM   4194  CG  PRO A 309     -12.903  16.028 -75.274  1.00111.10           C
ATOM   4195  CD  PRO A 309     -12.079  14.902 -74.701  1.00106.47           C
```

ATOM 4196 HA PRO A 309 -9.923 16.391 -76.566 1.00112.90 H
ATOM 4197 HB2 PRO A 309 -12.365 17.662 -76.575 1.00115.55 H
ATOM 4198 HB3 PRO A 309 -12.105 16.056 -77.237 1.00115.55 H
ATOM 4199 HG2 PRO A 309 -13.109 16.759 -74.491 1.00111.10 H
ATOM 4200 HG3 PRO A 309 -13.867 15.692 -75.657 1.00111.10 H
ATOM 4201 HD2 PRO A 309 -12.351 14.691 -73.667 1.00106.47 H
ATOM 4202 HD3 PRO A 309 -12.207 13.993 -75.291 1.00106.47 H
ATOM 4203 N GLU A 310 -9.131 18.418 -75.379 1.00107.44 N
ATOM 4204 CA GLU A 310 -8.588 19.606 -74.723 1.00105.77 C
ATOM 4205 C GLU A 310 -9.529 20.782 -74.941 1.00101.57 C
ATOM 4206 O GLU A 310 -9.535 21.402 -76.006 1.00110.79 O
ATOM 4207 CB GLU A 310 -7.199 19.913 -75.267 1.00114.60 C
ATOM 4208 CG GLU A 310 -6.524 18.720 -75.921 1.00119.89 C
ATOM 4209 CD GLU A 310 -5.102 19.006 -76.361 1.00126.69 C
ATOM 4210 OE1 GLU A 310 -4.620 20.139 -76.152 1.00130.51 O
ATOM 4211 OE2 GLU A 310 -4.464 18.089 -76.918 1.00127.18 O1-
ATOM 4212 H GLU A 310 -8.578 18.029 -76.129 1.00107.44 H
ATOM 4213 HA GLU A 310 -8.474 19.438 -73.658 1.00105.77 H
ATOM 4214 HB2 GLU A 310 -7.194 20.740 -75.979 1.00114.60 H
ATOM 4215 HB3 GLU A 310 -6.585 20.257 -74.432 1.00114.60 H
ATOM 4216 HG2 GLU A 310 -6.494 17.873 -75.234 1.00119.89 H
ATOM 4217 HG3 GLU A 310 -7.059 18.404 -76.817 1.00119.89 H
ATOM 4218 N THR A 311 -10.338 21.088 -73.931 1.00 94.81 N
ATOM 4219 CA THR A 311 -11.302 22.180 -73.979 1.00 94.75 C
ATOM 4220 C THR A 311 -11.094 23.084 -72.767 1.00 96.99 C
ATOM 4221 O THR A 311 -10.239 22.830 -71.912 1.00 90.79 O
ATOM 4222 CB THR A 311 -12.745 21.657 -74.016 1.00 94.98 C
ATOM 4223 CG2 THR A 311 -12.964 20.795 -75.247 1.00 90.62 C
ATOM 4224 OG1 THR A 311 -13.003 20.876 -72.840 1.00 95.98 O
ATOM 4225 H THR A 311 -10.297 20.560 -73.072 1.00 94.81 H
ATOM 4226 HA THR A 311 -11.145 22.816 -74.852 1.00 94.75 H
ATOM 4227 HB THR A 311 -13.458 22.482 -74.044 1.00 94.98 H
ATOM 4228 HG1 THR A 311 -12.612 20.007 -72.969 1.00 95.98 H
ATOM 4229 HG21 THR A 311 -13.999 20.456 -75.298 1.00 90.62 H
ATOM 4230 HG22 THR A 311 -12.768 21.363 -76.157 1.00 90.62 H
ATOM 4231 HG23 THR A 311 -12.339 19.910 -75.280 1.00 90.62 H
ATOM 4232 N THR A 312 -11.897 24.150 -72.692 1.00 82.29 N
ATOM 4233 CA THR A 312 -11.830 25.053 -71.548 1.00 79.96 C
ATOM 4234 C THR A 312 -12.268 24.359 -70.264 1.00 80.22 C
ATOM 4235 O THR A 312 -11.645 24.537 -69.210 1.00 78.83 O

```
ATOM   4236  CB  THR A 312     -12.694  26.285 -71.812  1.00 78.08           C
ATOM   4237  CG2 THR A 312     -12.725  27.195 -70.594  1.00 74.44           C
ATOM   4238  OG1 THR A 312     -12.166  27.007 -72.931  1.00 86.91           O
ATOM   4239  H   THR A 312     -12.563  24.366 -73.419  1.00 82.29           H
ATOM   4240  HA  THR A 312     -10.794  25.377 -71.422  1.00 79.96           H
ATOM   4241  HB  THR A 312     -13.714  25.989 -72.064  1.00 78.08           H
ATOM   4242  HG1 THR A 312     -12.639  27.838 -73.014  1.00 86.91           H
ATOM   4243 HG21 THR A 312     -13.186  28.150 -70.847  1.00 74.44           H
ATOM   4244 HG22 THR A 312     -13.309  26.784 -69.770  1.00 74.44           H
ATOM   4245 HG23 THR A 312     -11.717  27.397 -70.230  1.00 74.44           H
ATOM   4246  N   PHE A 313     -13.334  23.557 -70.332  1.00 80.17           N
ATOM   4247  CA  PHE A 313     -13.826  22.892 -69.130  1.00 77.78           C
ATOM   4248  C   PHE A 313     -12.825  21.867 -68.612  1.00 71.21           C
ATOM   4249  O   PHE A 313     -12.668  21.711 -67.397  1.00 67.17           O
ATOM   4250  CB  PHE A 313     -15.178  22.232 -69.399  1.00 80.51           C
ATOM   4251  CG  PHE A 313     -15.814  21.640 -68.170  1.00 83.53           C
ATOM   4252  CD1 PHE A 313     -16.499  22.445 -67.273  1.00 88.19           C
ATOM   4253  CD2 PHE A 313     -15.726  20.281 -67.911  1.00 82.08           C
ATOM   4254  CE1 PHE A 313     -17.085  21.906 -66.142  1.00 91.16           C
ATOM   4255  CE2 PHE A 313     -16.309  19.736 -66.781  1.00 86.33           C
ATOM   4256  CZ  PHE A 313     -16.990  20.550 -65.895  1.00 90.23           C
ATOM   4257  H   PHE A 313     -13.825  23.399 -71.200  1.00 80.17           H
ATOM   4258  HA  PHE A 313     -13.969  23.649 -68.355  1.00 77.78           H
ATOM   4259  HB2 PHE A 313     -15.867  22.974 -69.804  1.00 80.51           H
ATOM   4260  HB3 PHE A 313     -15.082  21.465 -70.170  1.00 80.51           H
ATOM   4261  HD1 PHE A 313     -16.578  23.507 -67.454  1.00 88.19           H
ATOM   4262  HD2 PHE A 313     -15.193  19.635 -68.594  1.00 82.08           H
ATOM   4263  HE1 PHE A 313     -17.615  22.545 -65.451  1.00 91.16           H
ATOM   4264  HE2 PHE A 313     -16.230  18.676 -66.590  1.00 86.33           H
ATOM   4265  HZ  PHE A 313     -17.446  20.127 -65.012  1.00 90.23           H
ATOM   4266  N   GLN A 314     -12.137  21.159 -69.511  1.00 67.12           N
ATOM   4267  CA  GLN A 314     -11.115  20.213 -69.072  1.00 68.30           C
ATOM   4268  C   GLN A 314      -9.958  20.935 -68.395  1.00 62.26           C
ATOM   4269  O   GLN A 314      -9.480  20.515 -67.335  1.00 55.39           O
ATOM   4270  CB  GLN A 314     -10.598  19.392 -70.251  1.00 71.69           C
ATOM   4271  CG  GLN A 314      -9.351  18.594 -69.893  1.00 88.41           C
ATOM   4272  CD  GLN A 314      -8.632  18.015 -71.089  1.00103.89           C
ATOM   4273  NE2 GLN A 314      -9.244  17.714 -72.111  1.00108.31           N
ATOM   4274  OE1 GLN A 314      -7.322  17.846 -70.963  1.00115.45           O
ATOM   4275  H   GLN A 314     -12.277  21.287 -70.504  1.00 67.12           H
```

ATOM 4276 HA GLN A 314 -11.560 19.516 -68.361 1.00 68.30 H
ATOM 4277 HB2 GLN A 314 -11.385 18.730 -70.616 1.00 71.69 H
ATOM 4278 HB3 GLN A 314 -10.366 20.073 -71.072 1.00 71.69 H
ATOM 4279 HG2 GLN A 314 -8.575 19.106 -69.328 1.00 88.41 H
ATOM 4280 HG3 GLN A 314 -9.661 17.723 -69.331 1.00 88.41 H
ATOM 4281 HE21 GLN A 314 -10.240 17.864 -72.193 1.00108.31 H
ATOM 4282 HE22 GLN A 314 -8.742 17.324 -72.897 1.00108.31 H
ATOM 4283 N THR A 315 -9.489 22.024 -69.006 1.00 57.52 N
ATOM 4284 CA THR A 315 -8.383 22.785 -68.437 1.00 56.67 C
ATOM 4285 C THR A 315 -8.737 23.320 -67.056 1.00 54.16 C
ATOM 4286 O THR A 315 -7.980 23.139 -66.094 1.00 56.24 O
ATOM 4287 CB THR A 315 -8.006 23.931 -69.376 1.00 58.78 C
ATOM 4288 CG2 THR A 315 -7.144 24.957 -68.654 1.00 56.49 C
ATOM 4289 OG1 THR A 315 -7.302 23.410 -70.511 1.00 63.55 O
ATOM 4290 H THR A 315 -9.890 22.352 -69.875 1.00 57.52 H
ATOM 4291 HA THR A 315 -7.524 22.117 -68.330 1.00 56.67 H
ATOM 4292 HB THR A 315 -8.902 24.428 -69.752 1.00 58.78 H
ATOM 4293 HG1 THR A 315 -7.912 22.884 -71.036 1.00 63.55 H
ATOM 4294 HG21 THR A 315 -6.728 25.663 -69.374 1.00 56.49 H
ATOM 4295 HG22 THR A 315 -7.695 25.566 -67.936 1.00 56.49 H
ATOM 4296 HG23 THR A 315 -6.297 24.491 -68.149 1.00 56.49 H
ATOM 4297 N VAL A 316 -9.892 23.979 -66.940 1.00 51.39 N
ATOM 4298 CA VAL A 316 -10.287 24.570 -65.666 1.00 55.78 C
ATOM 4299 C VAL A 316 -10.496 23.490 -64.612 1.00 47.82 C
ATOM 4300 O VAL A 316 -10.053 23.631 -63.466 1.00 50.50 O
ATOM 4301 CB VAL A 316 -11.545 25.439 -65.847 1.00 53.30 C
ATOM 4302 CG1 VAL A 316 -12.050 25.928 -64.498 1.00 54.04 C
ATOM 4303 CG2 VAL A 316 -11.243 26.615 -66.761 1.00 54.96 C
ATOM 4304 H VAL A 316 -10.506 24.115 -67.734 1.00 51.39 H
ATOM 4305 HA VAL A 316 -9.474 25.214 -65.322 1.00 55.78 H
ATOM 4306 HB VAL A 316 -12.330 24.843 -66.317 1.00 53.30 H
ATOM 4307 HG11 VAL A 316 -12.836 26.672 -64.630 1.00 54.04 H
ATOM 4308 HG12 VAL A 316 -12.488 25.131 -63.896 1.00 54.04 H
ATOM 4309 HG13 VAL A 316 -11.256 26.398 -63.916 1.00 54.04 H
ATOM 4310 HG21 VAL A 316 -12.151 27.174 -66.987 1.00 54.96 H
ATOM 4311 HG22 VAL A 316 -10.544 27.306 -66.287 1.00 54.96 H
ATOM 4312 HG23 VAL A 316 -10.800 26.325 -67.711 1.00 54.96 H
ATOM 4313 N SER A 317 -11.163 22.393 -64.979 1.00 50.47 N
ATOM 4314 CA SER A 317 -11.410 21.328 -64.012 1.00 49.23 C
ATOM 4315 C SER A 317 -10.113 20.673 -63.555 1.00 44.14 C

ATOM 4316 O SER A 317 -10.006 20.259 -62.396 1.00 43.44 O
ATOM 4317 CB SER A 317 -12.355 20.283 -64.605 1.00 50.53 C
ATOM 4318 OG SER A 317 -11.782 19.666 -65.742 1.00 58.12 O
ATOM 4319 H SER A 317 -11.530 22.281 -65.915 1.00 50.47 H
ATOM 4320 HA SER A 317 -11.901 21.760 -63.138 1.00 49.23 H
ATOM 4321 HB2 SER A 317 -12.586 19.512 -63.869 1.00 50.53 H
ATOM 4322 HB3 SER A 317 -13.304 20.740 -64.887 1.00 50.53 H
ATOM 4323 HG SER A 317 -11.892 20.268 -66.485 1.00 58.12 H
ATOM 4324 N TRP A 318 -9.119 20.582 -64.442 1.00 45.31 N
ATOM 4325 CA TRP A 318 -7.839 19.984 -64.073 1.00 46.89 C
ATOM 4326 C TRP A 318 -7.142 20.799 -62.993 1.00 57.36 C
ATOM 4327 O TRP A 318 -6.685 20.253 -61.981 1.00 38.72 O
ATOM 4328 CB TRP A 318 -6.984 19.789 -65.341 1.00 48.57 C
ATOM 4329 CG TRP A 318 -5.548 19.414 -65.137 1.00 51.15 C
ATOM 4330 CD1 TRP A 318 -4.494 20.189 -65.476 1.00 48.66 C
ATOM 4331 CD2 TRP A 318 -4.985 18.218 -64.516 1.00 48.60 C
ATOM 4332 CE2 TRP A 318 -3.562 18.337 -64.526 1.00 48.21 C
ATOM 4333 CE3 TRP A 318 -5.523 17.046 -63.934 1.00 47.40 C
ATOM 4334 NE1 TRP A 318 -3.323 19.552 -65.127 1.00 48.89 N
ATOM 4335 CZ2 TRP A 318 -2.720 17.351 -63.990 1.00 49.10 C
ATOM 4336 CZ3 TRP A 318 -4.686 16.043 -63.402 1.00 42.95 C
ATOM 4337 CH2 TRP A 318 -3.288 16.201 -63.416 1.00 46.58 C
ATOM 4338 H TRP A 318 -9.233 20.905 -65.394 1.00 45.31 H
ATOM 4339 HA TRP A 318 -8.005 18.985 -63.665 1.00 46.89 H
ATOM 4340 HB2 TRP A 318 -7.440 19.026 -65.972 1.00 48.57 H
ATOM 4341 HB3 TRP A 318 -6.995 20.704 -65.933 1.00 48.57 H
ATOM 4342 HD1 TRP A 318 -4.574 21.163 -65.938 1.00 48.66 H
ATOM 4343 HE1 TRP A 318 -2.411 19.979 -65.254 1.00 48.89 H
ATOM 4344 HE3 TRP A 318 -6.595 16.925 -63.902 1.00 47.40 H
ATOM 4345 HZ2 TRP A 318 -1.649 17.487 -64.009 1.00 49.10 H
ATOM 4346 HZ3 TRP A 318 -5.116 15.152 -62.967 1.00 42.95 H
ATOM 4347 HH2 TRP A 318 -2.645 15.454 -62.977 1.00 46.58 H
ATOM 4348 N HIS A 319 -7.051 22.115 -63.188 1.00 40.89 N
ATOM 4349 CA HIS A 319 -6.388 22.947 -62.193 1.00 48.50 C
ATOM 4350 C HIS A 319 -7.219 23.072 -60.924 1.00 46.14 C
ATOM 4351 O HIS A 319 -6.659 23.242 -59.836 1.00 43.27 O
ATOM 4352 CB HIS A 319 -6.036 24.316 -62.806 1.00 41.28 C
ATOM 4353 CG HIS A 319 -4.888 24.236 -63.778 1.00 47.80 C
ATOM 4354 CD2 HIS A 319 -4.909 24.080 -65.147 1.00 44.70 C
ATOM 4355 ND1 HIS A 319 -3.555 24.255 -63.357 1.00 42.39 N

ATOM 4356 CE1 HIS A 319 -2.836 24.073 -64.458 1.00 53.88 C
ATOM 4357 NE2 HIS A 319 -3.588 23.959 -65.549 1.00 45.55 N
ATOM 4358 H HIS A 319 -7.431 22.557 -64.015 1.00 40.89 H
ATOM 4359 HA HIS A 319 -5.438 22.488 -61.910 1.00 48.50 H
ATOM 4360 HB2 HIS A 319 -6.903 24.753 -63.301 1.00 41.28 H
ATOM 4361 HB3 HIS A 319 -5.749 25.011 -62.016 1.00 41.28 H
ATOM 4362 HD2 HIS A 319 -5.733 24.011 -65.838 1.00 44.70 H
ATOM 4363 HE1 HIS A 319 -1.756 24.009 -64.467 1.00 53.88 H
ATOM 4364 HE2 HIS A 319 -3.244 23.770 -66.482 1.00 0.00 H
ATOM 4365 N PHE A 320 -8.546 22.973 -61.036 1.00 44.49 N
ATOM 4366 CA PHE A 320 -9.380 22.972 -59.839 1.00 46.14 C
ATOM 4367 C PHE A 320 -9.158 21.708 -59.019 1.00 45.94 C
ATOM 4368 O PHE A 320 -9.114 21.762 -57.784 1.00 43.91 O
ATOM 4369 CB PHE A 320 -10.853 23.114 -60.220 1.00 48.18 C
ATOM 4370 CG PHE A 320 -11.761 23.325 -59.042 1.00 66.95 C
ATOM 4371 CD1 PHE A 320 -11.960 24.594 -58.524 1.00 72.86 C
ATOM 4372 CD2 PHE A 320 -12.413 22.256 -58.450 1.00 70.49 C
ATOM 4373 CE1 PHE A 320 -12.791 24.793 -57.438 1.00 72.24 C
ATOM 4374 CE2 PHE A 320 -13.247 22.449 -57.364 1.00 73.40 C
ATOM 4375 CZ PHE A 320 -13.436 23.719 -56.858 1.00 71.48 C
ATOM 4376 H PHE A 320 -8.997 22.867 -61.936 1.00 44.49 H
ATOM 4377 HA PHE A 320 -9.097 23.816 -59.215 1.00 46.14 H
ATOM 4378 HB2 PHE A 320 -10.972 23.970 -60.886 1.00 48.18 H
ATOM 4379 HB3 PHE A 320 -11.189 22.247 -60.791 1.00 48.18 H
ATOM 4380 HD1 PHE A 320 -11.484 25.446 -58.980 1.00 72.86 H
ATOM 4381 HD2 PHE A 320 -12.273 21.257 -58.836 1.00 70.49 H
ATOM 4382 HE1 PHE A 320 -12.940 25.789 -57.047 1.00 72.24 H
ATOM 4383 HE2 PHE A 320 -13.765 21.612 -56.923 1.00 73.40 H
ATOM 4384 HZ PHE A 320 -14.090 23.873 -56.012 1.00 71.48 H
ATOM 4385 N CYS A 321 -9.018 20.560 -59.686 1.00 38.53 N
ATOM 4386 CA CYS A 321 -8.755 19.318 -58.970 1.00 37.83 C
ATOM 4387 C CYS A 321 -7.378 19.340 -58.319 1.00 38.04 C
ATOM 4388 O CYS A 321 -7.210 18.854 -57.194 1.00 40.50 O
ATOM 4389 CB CYS A 321 -8.869 18.098 -59.899 1.00 42.59 C
ATOM 4390 SG CYS A 321 -10.583 17.919 -60.469 1.00 47.33 S
ATOM 4391 H CYS A 321 -9.081 20.524 -60.696 1.00 38.53 H
ATOM 4392 HA CYS A 321 -9.498 19.200 -58.178 1.00 37.83 H
ATOM 4393 HB2 CYS A 321 -8.205 18.189 -60.761 1.00 42.59 H
ATOM 4394 HB3 CYS A 321 -8.588 17.190 -59.370 1.00 42.59 H
ATOM 4395 HG CYS A 321 -10.535 18.960 -61.319 1.00 47.33 H

ATOM 4396 N ILE A 322 -6.383 19.902 -59.011 1.00 35.14 N
ATOM 4397 CA ILE A 322 -5.076 20.117 -58.396 1.00 35.71 C
ATOM 4398 C ILE A 322 -5.217 20.979 -57.148 1.00 39.64 C
ATOM 4399 O ILE A 322 -4.694 20.647 -56.076 1.00 37.75 O
ATOM 4400 CB ILE A 322 -4.055 20.774 -59.381 1.00 39.56 C
ATOM 4401 CG1 ILE A 322 -3.727 19.833 -60.562 1.00 42.32 C
ATOM 4402 CG2 ILE A 322 -2.737 21.243 -58.721 1.00 41.06 C
ATOM 4403 CD1 ILE A 322 -3.159 20.552 -61.796 1.00 43.31 C
ATOM 4404 H ILE A 322 -6.531 20.266 -59.943 1.00 35.14 H
ATOM 4405 HA ILE A 322 -4.678 19.147 -58.091 1.00 35.71 H
ATOM 4406 HB ILE A 322 -4.527 21.663 -59.802 1.00 39.56 H
ATOM 4407 HG12 ILE A 322 -3.036 19.055 -60.239 1.00 42.32 H
ATOM 4408 HG13 ILE A 322 -4.619 19.302 -60.885 1.00 42.32 H
ATOM 4409 HG21 ILE A 322 -2.017 21.606 -59.455 1.00 41.06 H
ATOM 4410 HG22 ILE A 322 -2.891 22.064 -58.020 1.00 41.06 H
ATOM 4411 HG23 ILE A 322 -2.268 20.422 -58.182 1.00 41.06 H
ATOM 4412 HD11 ILE A 322 -2.314 20.003 -62.212 1.00 43.31 H
ATOM 4413 HD12 ILE A 322 -3.913 20.632 -62.578 1.00 43.31 H
ATOM 4414 HD13 ILE A 322 -2.816 21.562 -61.579 1.00 43.31 H
ATOM 4415 N ALA A 323 -5.934 22.099 -57.271 1.00 40.13 N
ATOM 4416 CA ALA A 323 -6.091 23.009 -56.141 1.00 40.25 C
ATOM 4417 C ALA A 323 -6.810 22.333 -54.982 1.00 41.82 C
ATOM 4418 O ALA A 323 -6.485 22.580 -53.815 1.00 40.83 O
ATOM 4419 CB ALA A 323 -6.843 24.267 -56.604 1.00 38.09 C
ATOM 4420 H ALA A 323 -6.344 22.369 -58.156 1.00 40.13 H
ATOM 4421 HA ALA A 323 -5.099 23.309 -55.797 1.00 40.25 H
ATOM 4422 HB1 ALA A 323 -6.959 24.980 -55.790 1.00 38.09 H
ATOM 4423 HB2 ALA A 323 -6.307 24.773 -57.407 1.00 38.09 H
ATOM 4424 HB3 ALA A 323 -7.842 24.028 -56.972 1.00 38.09 H
ATOM 4425 N LEU A 324 -7.786 21.470 -55.282 1.00 39.75 N
ATOM 4426 CA LEU A 324 -8.499 20.763 -54.222 1.00 45.08 C
ATOM 4427 C LEU A 324 -7.551 19.913 -53.386 1.00 42.26 C
ATOM 4428 O LEU A 324 -7.698 19.832 -52.160 1.00 39.12 O
ATOM 4429 CB LEU A 324 -9.655 19.909 -54.777 1.00 43.49 C
ATOM 4430 CG LEU A 324 -10.943 20.693 -55.110 1.00 48.74 C
ATOM 4431 CD1 LEU A 324 -11.974 19.733 -55.724 1.00 48.40 C
ATOM 4432 CD2 LEU A 324 -11.526 21.426 -53.880 1.00 45.58 C
ATOM 4433 H LEU A 324 -8.068 21.304 -56.240 1.00 39.75 H
ATOM 4434 HA LEU A 324 -8.919 21.512 -53.552 1.00 45.08 H
ATOM 4435 HB2 LEU A 324 -9.313 19.355 -55.652 1.00 43.49 H

ATOM 4436 HB3 LEU A 324 -9.916 19.145 -54.044 1.00 43.49 H
ATOM 4437 HG LEU A 324 -10.713 21.452 -55.857 1.00 48.74 H
ATOM 4438 HD11 LEU A 324 -13.004 20.017 -55.515 1.00 48.40 H
ATOM 4439 HD12 LEU A 324 -11.861 19.674 -56.807 1.00 48.40 H
ATOM 4440 HD13 LEU A 324 -11.843 18.734 -55.316 1.00 48.40 H
ATOM 4441 HD21 LEU A 324 -12.604 21.296 -53.779 1.00 45.58 H
ATOM 4442 HD22 LEU A 324 -11.085 21.080 -52.945 1.00 45.58 H
ATOM 4443 HD23 LEU A 324 -11.344 22.499 -53.951 1.00 45.58 H
ATOM 4444 N GLY A 325 -6.572 19.273 -54.030 1.00 40.68 N
ATOM 4445 CA GLY A 325 -5.598 18.492 -53.285 1.00 42.40 C
ATOM 4446 C GLY A 325 -4.819 19.327 -52.287 1.00 43.10 C
ATOM 4447 O GLY A 325 -4.540 18.880 -51.171 1.00 38.59 O
ATOM 4448 H GLY A 325 -6.474 19.345 -55.034 1.00 40.68 H
ATOM 4449 HA2 GLY A 325 -6.084 17.666 -52.764 1.00 42.40 H
ATOM 4450 HA3 GLY A 325 -4.896 18.050 -53.992 1.00 42.40 H
ATOM 4451 N TYR A 326 -4.461 20.553 -52.670 1.00 36.00 N
ATOM 4452 CA TYR A 326 -3.732 21.426 -51.761 1.00 35.84 C
ATOM 4453 C TYR A 326 -4.643 22.100 -50.743 1.00 40.18 C
ATOM 4454 O TYR A 326 -4.145 22.602 -49.729 1.00 43.17 O
ATOM 4455 CB TYR A 326 -2.982 22.498 -52.559 1.00 36.58 C
ATOM 4456 CG TYR A 326 -1.817 21.952 -53.343 1.00 39.48 C
ATOM 4457 CD1 TYR A 326 -0.625 21.594 -52.678 1.00 34.77 C
ATOM 4458 CD2 TYR A 326 -1.931 21.790 -54.736 1.00 34.82 C
ATOM 4459 CE1 TYR A 326 0.456 21.076 -53.412 1.00 35.29 C
ATOM 4460 CE2 TYR A 326 -0.847 21.288 -55.468 1.00 33.92 C
ATOM 4461 CZ TYR A 326 0.340 20.938 -54.805 1.00 34.96 C
ATOM 4462 OH TYR A 326 1.363 20.451 -55.545 1.00 36.28 O
ATOM 4463 H TYR A 326 -4.696 20.908 -53.587 1.00 36.00 H
ATOM 4464 HA TYR A 326 -3.003 20.842 -51.193 1.00 35.84 H
ATOM 4465 HB2 TYR A 326 -3.665 23.013 -53.234 1.00 36.58 H
ATOM 4466 HB3 TYR A 326 -2.585 23.261 -51.888 1.00 36.58 H
ATOM 4467 HD1 TYR A 326 -0.537 21.708 -51.607 1.00 34.77 H
ATOM 4468 HD2 TYR A 326 -2.836 22.069 -55.253 1.00 34.82 H
ATOM 4469 HE1 TYR A 326 1.370 20.794 -52.910 1.00 35.29 H
ATOM 4470 HE2 TYR A 326 -0.913 21.183 -56.540 1.00 33.92 H
ATOM 4471 HH TYR A 326 2.068 19.987 -55.058 1.00 36.28 H
ATOM 4472 N THR A 327 -5.957 22.108 -50.988 0.77 34.47 N
ATOM 4473 CA THR A 327 -6.899 22.616 -49.996 0.77 39.51 C
ATOM 4474 C THR A 327 -6.949 21.714 -48.769 0.77 39.47 C
ATOM 4475 O THR A 327 -7.284 22.174 -47.670 0.77 38.93 O

ATOM 4476 CB THR A 327 -8.311 22.762 -50.623 0.77 37.33 C
ATOM 4477 CG2 THR A 327 -9.381 23.375 -49.706 0.77 36.73 C
ATOM 4478 OG1 THR A 327 -8.212 23.599 -51.766 0.77 39.40 O
ATOM 4479 H THR A 327 -6.332 21.688 -51.827 0.77 34.47 H
ATOM 4480 HA THR A 327 -6.566 23.605 -49.679 0.77 39.51 H
ATOM 4481 HB THR A 327 -8.677 21.787 -50.946 0.77 37.33 H
ATOM 4482 HG1 THR A 327 -7.573 23.223 -52.393 0.77 39.40 H
ATOM 4483 HG21 THR A 327 -10.308 23.550 -50.252 0.77 36.73 H
ATOM 4484 HG22 THR A 327 -9.625 22.715 -48.874 0.77 36.73 H
ATOM 4485 HG23 THR A 327 -9.053 24.329 -49.293 0.77 36.73 H
ATOM 4486 N ASN A 328 -6.613 20.433 -48.934 1.00 37.95 N
ATOM 4487 CA ASN A 328 -6.510 19.542 -47.787 1.00 34.67 C
ATOM 4488 C ASN A 328 -5.457 20.049 -46.812 1.00 42.24 C
ATOM 4489 O ASN A 328 -5.640 19.978 -45.592 1.00 41.57 O
ATOM 4490 CB ASN A 328 -6.196 18.083 -48.193 1.00 33.38 C
ATOM 4491 CG ASN A 328 -6.268 17.106 -47.008 1.00 37.08 C
ATOM 4492 ND2 ASN A 328 -7.437 16.924 -46.406 1.00 35.00 N
ATOM 4493 OD1 ASN A 328 -5.272 16.489 -46.641 1.00 41.71 O
ATOM 4494 H ASN A 328 -6.354 20.070 -49.841 1.00 37.95 H
ATOM 4495 HA ASN A 328 -7.459 19.539 -47.245 1.00 34.67 H
ATOM 4496 HB2 ASN A 328 -6.886 17.732 -48.955 1.00 33.38 H
ATOM 4497 HB3 ASN A 328 -5.202 18.017 -48.640 1.00 33.38 H
ATOM 4498 HD21 ASN A 328 -8.272 17.398 -46.708 1.00 35.00 H
ATOM 4499 HD22 ASN A 328 -7.448 16.373 -45.560 1.00 35.00 H
ATOM 4500 N SER A 329 -4.354 20.589 -47.338 1.00 39.43 N
ATOM 4501 CA SER A 329 -3.346 21.212 -46.493 1.00 39.61 C
ATOM 4502 C SER A 329 -3.847 22.506 -45.865 1.00 47.08 C
ATOM 4503 O SER A 329 -3.344 22.904 -44.808 1.00 48.88 O
ATOM 4504 CB SER A 329 -2.105 21.484 -47.373 1.00 43.26 C
ATOM 4505 OG SER A 329 -1.612 20.282 -47.941 1.00 42.58 O
ATOM 4506 H SER A 329 -4.211 20.628 -48.337 1.00 39.43 H
ATOM 4507 HA SER A 329 -3.107 20.525 -45.681 1.00 39.61 H
ATOM 4508 HB2 SER A 329 -2.329 22.186 -48.172 1.00 43.26 H
ATOM 4509 HB3 SER A 329 -1.308 21.954 -46.805 1.00 43.26 H
ATOM 4510 HG SER A 329 -1.353 19.703 -47.209 1.00 42.58 H
ATOM 4511 N CYS A 330 -4.828 23.164 -46.487 1.00 40.13 N
ATOM 4512 CA CYS A 330 -5.420 24.363 -45.905 1.00 42.73 C
ATOM 4513 C CYS A 330 -6.389 24.035 -44.778 1.00 49.84 C
ATOM 4514 O CYS A 330 -6.523 24.822 -43.834 1.00 41.74 O
ATOM 4515 CB CYS A 330 -6.154 25.173 -46.976 1.00 44.61 C

ATOM 4516 SG CYS A 330 -5.111 25.842 -48.283 1.00 47.63 S
ATOM 4517 H CYS A 330 -5.228 22.812 -47.345 1.00 40.13 H
ATOM 4518 HA CYS A 330 -4.633 24.998 -45.492 1.00 42.73 H
ATOM 4519 HB2 CYS A 330 -6.944 24.593 -47.445 1.00 44.61 H
ATOM 4520 HB3 CYS A 330 -6.667 26.014 -46.512 1.00 44.61 H
ATOM 4521 HG CYS A 330 -4.765 24.663 -48.809 1.00 47.63 H
ATOM 4522 N LEU A 331 -7.075 22.896 -44.860 1.00 40.93 N
ATOM 4523 CA LEU A 331 -8.082 22.543 -43.869 1.00 42.24 C
ATOM 4524 C LEU A 331 -7.528 21.710 -42.721 1.00 42.25 C
ATOM 4525 O LEU A 331 -8.116 21.716 -41.634 1.00 42.05 O
ATOM 4526 CB LEU A 331 -9.237 21.793 -44.541 1.00 38.65 C
ATOM 4527 CG LEU A 331 -10.006 22.599 -45.593 1.00 45.28 C
ATOM 4528 CD1 LEU A 331 -11.029 21.733 -46.312 1.00 43.97 C
ATOM 4529 CD2 LEU A 331 -10.680 23.809 -44.960 1.00 50.98 C
ATOM 4530 H LEU A 331 -6.962 22.280 -45.654 1.00 40.93 H
ATOM 4531 HA LEU A 331 -8.496 23.442 -43.410 1.00 42.24 H
ATOM 4532 HB2 LEU A 331 -8.841 20.891 -45.012 1.00 38.65 H
ATOM 4533 HB3 LEU A 331 -9.941 21.442 -43.784 1.00 38.65 H
ATOM 4534 HG LEU A 331 -9.314 22.969 -46.348 1.00 45.28 H
ATOM 4535 HD11 LEU A 331 -11.740 22.328 -46.886 1.00 43.97 H
ATOM 4536 HD12 LEU A 331 -10.534 21.065 -47.017 1.00 43.97 H
ATOM 4537 HD13 LEU A 331 -11.597 21.118 -45.614 1.00 43.97 H
ATOM 4538 HD21 LEU A 331 -11.706 23.946 -45.303 1.00 50.98 H
ATOM 4539 HD22 LEU A 331 -10.726 23.742 -43.872 1.00 50.98 H
ATOM 4540 HD23 LEU A 331 -10.138 24.723 -45.207 1.00 50.98 H
ATOM 4541 N ASN A 332 -6.419 21.002 -42.936 1.00 33.22 N
ATOM 4542 CA ASN A 332 -5.813 20.227 -41.855 1.00 40.13 C
ATOM 4543 C ASN A 332 -5.482 21.051 -40.613 1.00 42.34 C
ATOM 4544 O ASN A 332 -5.663 20.523 -39.502 1.00 44.41 O
ATOM 4545 CB ASN A 332 -4.564 19.503 -42.372 1.00 35.44 C
ATOM 4546 CG ASN A 332 -4.904 18.285 -43.206 1.00 38.05 C
ATOM 4547 ND2 ASN A 332 -3.923 17.777 -43.941 1.00 39.09 N
ATOM 4548 OD1 ASN A 332 -6.037 17.803 -43.186 1.00 44.48 O
ATOM 4549 H ASN A 332 -5.973 20.978 -43.844 1.00 33.22 H
ATOM 4550 HA ASN A 332 -6.584 19.508 -41.570 1.00 40.13 H
ATOM 4551 HB2 ASN A 332 -3.944 20.185 -42.951 1.00 35.44 H
ATOM 4552 HB3 ASN A 332 -3.956 19.146 -41.542 1.00 35.44 H
ATOM 4553 HD21 ASN A 332 -3.005 18.206 -43.925 1.00 39.09 H
ATOM 4554 HD22 ASN A 332 -4.090 16.980 -44.539 1.00 39.09 H
ATOM 4555 N PRO A 333 -4.996 22.298 -40.703 1.00 38.90 N

ATOM 4556 CA PRO A 333 -4.837 23.093 -39.473 1.00 44.34 C
ATOM 4557 C PRO A 333 -6.130 23.262 -38.701 1.00 49.44 C
ATOM 4558 O PRO A 333 -6.105 23.296 -37.465 1.00 56.97 O
ATOM 4559 CB PRO A 333 -4.314 24.442 -39.985 1.00 50.31 C
ATOM 4560 CG PRO A 333 -3.669 24.132 -41.264 1.00 44.88 C
ATOM 4561 CD PRO A 333 -4.449 23.008 -41.873 1.00 36.54 C
ATOM 4562 HA PRO A 333 -4.080 22.619 -38.845 1.00 44.34 H
ATOM 4563 HB2 PRO A 333 -5.146 25.120 -40.187 1.00 50.31 H
ATOM 4564 HB3 PRO A 333 -3.661 24.970 -39.300 1.00 50.31 H
ATOM 4565 HG2 PRO A 333 -3.521 24.989 -41.922 1.00 44.88 H
ATOM 4566 HG3 PRO A 333 -2.706 23.754 -40.960 1.00 44.88 H
ATOM 4567 HD2 PRO A 333 -5.269 23.384 -42.456 1.00 36.54 H
ATOM 4568 HD3 PRO A 333 -3.815 22.365 -42.477 1.00 36.54 H
ATOM 4569 N VAL A 334 -7.265 23.369 -39.392 1.00 43.96 N
ATOM 4570 CA VAL A 334 -8.540 23.488 -38.696 1.00 42.00 C
ATOM 4571 C VAL A 334 -8.925 22.157 -38.067 1.00 49.70 C
ATOM 4572 O VAL A 334 -9.261 22.087 -36.879 1.00 50.19 O
ATOM 4573 CB VAL A 334 -9.633 23.992 -39.654 1.00 47.12 C
ATOM 4574 CG1 VAL A 334 -10.961 24.102 -38.921 1.00 51.15 C
ATOM 4575 CG2 VAL A 334 -9.237 25.331 -40.259 1.00 54.94 C
ATOM 4576 H VAL A 334 -7.280 23.320 -40.401 1.00 43.96 H
ATOM 4577 HA VAL A 334 -8.462 24.207 -37.885 1.00 42.00 H
ATOM 4578 HB VAL A 334 -9.770 23.288 -40.474 1.00 47.12 H
ATOM 4579 HG11 VAL A 334 -11.710 24.588 -39.547 1.00 51.15 H
ATOM 4580 HG12 VAL A 334 -11.373 23.128 -38.657 1.00 51.15 H
ATOM 4581 HG13 VAL A 334 -10.870 24.690 -38.007 1.00 51.15 H
ATOM 4582 HG21 VAL A 334 -10.033 25.725 -40.891 1.00 54.94 H
ATOM 4583 HG22 VAL A 334 -9.031 26.073 -39.486 1.00 54.94 H
ATOM 4584 HG23 VAL A 334 -8.350 25.248 -40.888 1.00 54.94 H
ATOM 4585 N LEU A 335 -8.859 21.078 -38.851 1.00 42.29 N
ATOM 4586 CA LEU A 335 -9.373 19.791 -38.395 1.00 43.08 C
ATOM 4587 C LEU A 335 -8.516 19.198 -37.285 1.00 40.27 C
ATOM 4588 O LEU A 335 -9.045 18.612 -36.335 1.00 46.11 O
ATOM 4589 CB LEU A 335 -9.459 18.815 -39.569 1.00 44.55 C
ATOM 4590 CG LEU A 335 -10.340 19.216 -40.752 1.00 53.16 C
ATOM 4591 CD1 LEU A 335 -10.269 18.161 -41.846 1.00 53.81 C
ATOM 4592 CD2 LEU A 335 -11.774 19.426 -40.299 1.00 54.61 C
ATOM 4593 H LEU A 335 -8.558 21.153 -39.815 1.00 42.29 H
ATOM 4594 HA LEU A 335 -10.376 19.937 -37.988 1.00 43.08 H
ATOM 4595 HB2 LEU A 335 -8.450 18.652 -39.952 1.00 44.55 H

ATOM 4596 HB3 LEU A 335 -9.784 17.845 -39.192 1.00 44.55 H
ATOM 4597 HG LEU A 335 -9.992 20.155 -41.174 1.00 53.16 H
ATOM 4598 HD11 LEU A 335 -11.252 17.838 -42.186 1.00 53.81 H
ATOM 4599 HD12 LEU A 335 -9.738 18.551 -42.714 1.00 53.81 H
ATOM 4600 HD13 LEU A 335 -9.735 17.265 -41.527 1.00 53.81 H
ATOM 4601 HD21 LEU A 335 -12.472 19.387 -41.135 1.00 54.61 H
ATOM 4602 HD22 LEU A 335 -12.086 18.685 -39.562 1.00 54.61 H
ATOM 4603 HD23 LEU A 335 -11.894 20.412 -39.848 1.00 54.61 H
ATOM 4604 N TYR A 336 -7.193 19.337 -37.383 1.00 38.13 N
ATOM 4605 CA TYR A 336 -6.286 18.592 -36.523 1.00 36.77 C
ATOM 4606 C TYR A 336 -5.520 19.447 -35.524 1.00 41.70 C
ATOM 4607 O TYR A 336 -4.908 18.889 -34.606 1.00 47.50 O
ATOM 4608 CB TYR A 336 -5.287 17.801 -37.378 1.00 40.31 C
ATOM 4609 CG TYR A 336 -5.948 16.732 -38.216 1.00 36.37 C
ATOM 4610 CD1 TYR A 336 -6.325 15.520 -37.651 1.00 44.65 C
ATOM 4611 CD2 TYR A 336 -6.205 16.937 -39.566 1.00 38.09 C
ATOM 4612 CE1 TYR A 336 -6.934 14.539 -38.405 1.00 41.75 C
ATOM 4613 CE2 TYR A 336 -6.813 15.959 -40.332 1.00 37.63 C
ATOM 4614 CZ TYR A 336 -7.174 14.762 -39.744 1.00 37.99 C
ATOM 4615 OH TYR A 336 -7.780 13.785 -40.493 1.00 41.06 O
ATOM 4616 H TYR A 336 -6.782 19.838 -38.161 1.00 38.13 H
ATOM 4617 HA TYR A 336 -6.818 17.857 -35.916 1.00 36.77 H
ATOM 4618 HB2 TYR A 336 -4.723 18.475 -38.024 1.00 40.31 H
ATOM 4619 HB3 TYR A 336 -4.543 17.310 -36.753 1.00 40.31 H
ATOM 4620 HD1 TYR A 336 -6.138 15.338 -36.604 1.00 44.65 H
ATOM 4621 HD2 TYR A 336 -5.920 17.868 -40.031 1.00 38.09 H
ATOM 4622 HE1 TYR A 336 -7.214 13.601 -37.949 1.00 41.75 H
ATOM 4623 HE2 TYR A 336 -7.004 16.135 -41.380 1.00 37.63 H
ATOM 4624 HH TYR A 336 -7.887 14.031 -41.415 1.00 41.06 H
ATOM 4625 N ALA A 337 -5.528 20.769 -35.667 1.00 41.62 N
ATOM 4626 CA ALA A 337 -4.845 21.652 -34.725 1.00 40.93 C
ATOM 4627 C ALA A 337 -5.840 22.510 -33.971 1.00 42.77 C
ATOM 4628 O ALA A 337 -6.023 22.330 -32.762 1.00 47.21 O
ATOM 4629 CB ALA A 337 -3.805 22.511 -35.452 1.00 40.85 C
ATOM 4630 H ALA A 337 -6.031 21.205 -36.427 1.00 41.62 H
ATOM 4631 HA ALA A 337 -4.294 21.091 -33.970 1.00 40.93 H
ATOM 4632 HB1 ALA A 337 -3.074 22.896 -34.740 1.00 40.85 H
ATOM 4633 HB2 ALA A 337 -3.253 21.926 -36.188 1.00 40.85 H
ATOM 4634 HB3 ALA A 337 -4.214 23.378 -35.963 1.00 40.85 H
ATOM 4635 N PHE A 338 -6.516 23.446 -34.648 1.00 42.79 N

ATOM 4636 CA PHE A 338 -7.403 24.377 -33.961 1.00 51.49 C
ATOM 4637 C PHE A 338 -8.590 23.685 -33.300 1.00 53.22 C
ATOM 4638 O PHE A 338 -9.168 24.243 -32.363 1.00 52.31 O
ATOM 4639 CB PHE A 338 -7.895 25.450 -34.936 1.00 50.83 C
ATOM 4640 CG PHE A 338 -6.794 26.313 -35.493 1.00 62.73 C
ATOM 4641 CD1 PHE A 338 -6.030 27.110 -34.655 1.00 74.94 C
ATOM 4642 CD2 PHE A 338 -6.529 26.334 -36.853 1.00 68.50 C
ATOM 4643 CE1 PHE A 338 -5.018 27.905 -35.163 1.00 77.44 C
ATOM 4644 CE2 PHE A 338 -5.520 27.129 -37.368 1.00 65.34 C
ATOM 4645 CZ PHE A 338 -4.763 27.913 -36.521 1.00 71.54 C
ATOM 4646 H PHE A 338 -6.374 23.575 -35.643 1.00 42.79 H
ATOM 4647 HA PHE A 338 -6.839 24.863 -33.164 1.00 51.49 H
ATOM 4648 HB2 PHE A 338 -8.443 24.981 -35.753 1.00 50.83 H
ATOM 4649 HB3 PHE A 338 -8.610 26.109 -34.440 1.00 50.83 H
ATOM 4650 HD1 PHE A 338 -6.223 27.117 -33.592 1.00 74.94 H
ATOM 4651 HD2 PHE A 338 -7.117 25.752 -37.539 1.00 68.50 H
ATOM 4652 HE1 PHE A 338 -4.424 28.516 -34.499 1.00 77.44 H
ATOM 4653 HE2 PHE A 338 -5.322 27.133 -38.430 1.00 65.34 H
ATOM 4654 HZ PHE A 338 -3.963 28.515 -36.924 1.00 71.54 H
ATOM 4655 N LEU A 339 -8.963 22.491 -33.753 1.00 52.02 N
ATOM 4656 CA LEU A 339 -10.037 21.734 -33.123 1.00 52.69 C
ATOM 4657 C LEU A 339 -9.527 20.643 -32.189 1.00 53.87 C
ATOM 4658 O LEU A 339 -10.338 19.926 -31.597 1.00 58.78 O
ATOM 4659 CB LEU A 339 -10.954 21.126 -34.189 1.00 53.30 C
ATOM 4660 CG LEU A 339 -11.822 22.138 -34.945 1.00 59.15 C
ATOM 4661 CD1 LEU A 339 -12.700 21.444 -35.975 1.00 61.20 C
ATOM 4662 CD2 LEU A 339 -12.667 22.960 -33.980 1.00 55.62 C
ATOM 4663 H LEU A 339 -8.496 22.068 -34.543 1.00 52.02 H
ATOM 4664 HA LEU A 339 -10.648 22.376 -32.504 1.00 52.69 H
ATOM 4665 HB2 LEU A 339 -10.353 20.554 -34.897 1.00 53.30 H
ATOM 4666 HB3 LEU A 339 -11.631 20.401 -33.733 1.00 53.30 H
ATOM 4667 HG LEU A 339 -11.176 22.838 -35.474 1.00 59.15 H
ATOM 4668 HD11 LEU A 339 -12.548 21.877 -36.964 1.00 61.20 H
ATOM 4669 HD12 LEU A 339 -12.468 20.382 -36.061 1.00 61.20 H
ATOM 4670 HD13 LEU A 339 -13.765 21.513 -35.751 1.00 61.20 H
ATOM 4671 HD21 LEU A 339 -13.614 23.275 -34.419 1.00 55.62 H
ATOM 4672 HD22 LEU A 339 -12.903 22.412 -33.067 1.00 55.62 H
ATOM 4673 HD23 LEU A 339 -12.143 23.873 -33.693 1.00 55.62 H
ATOM 4674 N ASP A 340 -8.211 20.499 -32.044 1.00 51.25 N
ATOM 4675 CA ASP A 340 -7.663 19.574 -31.062 1.00 50.20 C

ATOM 4676 C ASP A 340 -8.004 20.050 -29.655 1.00 55.64 C
ATOM 4677 O ASP A 340 -8.012 21.251 -29.377 1.00 59.44 O
ATOM 4678 CB ASP A 340 -6.148 19.454 -31.230 1.00 50.12 C
ATOM 4679 CG ASP A 340 -5.505 18.603 -30.149 1.00 54.70 C
ATOM 4680 OD1 ASP A 340 -5.577 17.359 -30.244 1.00 54.79 O
ATOM 4681 OD2 ASP A 340 -4.931 19.180 -29.202 1.00 55.29 O1-
ATOM 4682 H ASP A 340 -7.561 21.113 -32.517 1.00 51.25 H
ATOM 4683 HA ASP A 340 -8.105 18.589 -31.228 1.00 50.20 H
ATOM 4684 HB2 ASP A 340 -5.913 18.996 -32.190 1.00 50.12 H
ATOM 4685 HB3 ASP A 340 -5.646 20.405 -31.276 1.00 50.12 H
ATOM 4686 N GLU A 341 -8.279 19.096 -28.762 1.00 61.54 N
ATOM 4687 CA GLU A 341 -8.791 19.438 -27.436 1.00 69.20 C
ATOM 4688 C GLU A 341 -7.788 20.271 -26.645 1.00 65.00 C
ATOM 4689 O GLU A 341 -8.147 21.289 -26.043 1.00 66.97 O
ATOM 4690 CB GLU A 341 -9.154 18.164 -26.672 1.00 87.63 C
ATOM 4691 CG GLU A 341 -10.373 18.308 -25.775 1.00102.93 C
ATOM 4692 CD GLU A 341 -11.676 18.131 -26.533 1.00110.21 C
ATOM 4693 OE1 GLU A 341 -12.287 17.047 -26.420 1.00112.37 O
ATOM 4694 OE2 GLU A 341 -12.083 19.069 -27.251 1.00111.51 O1-
ATOM 4695 H GLU A 341 -8.234 18.119 -29.012 1.00 61.54 H
ATOM 4696 HA GLU A 341 -9.687 20.042 -27.591 1.00 69.20 H
ATOM 4697 HB2 GLU A 341 -9.319 17.339 -27.368 1.00 87.63 H
ATOM 4698 HB3 GLU A 341 -8.316 17.831 -26.058 1.00 87.63 H
ATOM 4699 HG2 GLU A 341 -10.334 17.548 -24.994 1.00102.93 H
ATOM 4700 HG3 GLU A 341 -10.372 19.270 -25.262 1.00102.93 H
ATOM 4701 N ASN A 342 -6.521 19.849 -26.626 1.00 55.81 N
ATOM 4702 CA ASN A 342 -5.515 20.580 -25.863 1.00 59.45 C
ATOM 4703 C ASN A 342 -5.040 21.825 -26.603 1.00 64.98 C
ATOM 4704 O ASN A 342 -4.878 22.888 -25.991 1.00 68.28 O
ATOM 4705 CB ASN A 342 -4.329 19.668 -25.541 1.00 60.27 C
ATOM 4706 CG ASN A 342 -4.690 18.561 -24.573 1.00 58.49 C
ATOM 4707 ND2 ASN A 342 -4.775 17.334 -25.077 1.00 58.27 N
ATOM 4708 OD1 ASN A 342 -4.885 18.805 -23.385 1.00 60.81 O
ATOM 4709 H ASN A 342 -6.223 19.048 -27.166 1.00 55.81 H
ATOM 4710 HA ASN A 342 -5.933 20.871 -24.914 1.00 59.45 H
ATOM 4711 HB2 ASN A 342 -3.900 19.253 -26.456 1.00 60.27 H
ATOM 4712 HB3 ASN A 342 -3.533 20.248 -25.071 1.00 60.27 H
ATOM 4713 HD21 ASN A 342 -4.606 17.168 -26.059 1.00 58.27 H
ATOM 4714 HD22 ASN A 342 -5.012 16.558 -24.477 1.00 58.27 H
ATOM 4715 N PHE A 343 -4.806 21.707 -27.915 1.00 58.15 N

ATOM	4716	CA	PHE	A	343	-4.370	22.853	-28.710	1.00	67.35	C
ATOM	4717	C	PHE	A	343	-5.390	23.983	-28.650	1.00	75.79	C
ATOM	4718	O	PHE	A	343	-5.022	25.164	-28.658	1.00	79.57	O
ATOM	4719	CB	PHE	A	343	-4.130	22.409	-30.155	1.00	62.08	C
ATOM	4720	CG	PHE	A	343	-3.437	23.437	-31.014	1.00	61.49	C
ATOM	4721	CD1	PHE	A	343	-4.151	24.468	-31.603	1.00	65.05	C
ATOM	4722	CD2	PHE	A	343	-2.076	23.349	-31.261	1.00	59.92	C
ATOM	4723	CE1	PHE	A	343	-3.523	25.403	-32.401	1.00	63.36	C
ATOM	4724	CE2	PHE	A	343	-1.440	24.282	-32.061	1.00	59.42	C
ATOM	4725	CZ	PHE	A	343	-2.166	25.310	-32.633	1.00	48.89	C
ATOM	4726	H	PHE	A	343	-4.923	20.817	-28.392	1.00	58.15	H
ATOM	4727	HA	PHE	A	343	-3.426	23.211	-28.294	1.00	67.35	H
ATOM	4728	HB2	PHE	A	343	-3.572	21.472	-30.189	1.00	62.08	H
ATOM	4729	HB3	PHE	A	343	-5.106	22.221	-30.576	1.00	62.08	H
ATOM	4730	HD1	PHE	A	343	-5.215	24.553	-31.438	1.00	65.05	H
ATOM	4731	HD2	PHE	A	343	-1.499	22.547	-30.823	1.00	59.92	H
ATOM	4732	HE1	PHE	A	343	-4.092	26.204	-32.846	1.00	63.36	H
ATOM	4733	HE2	PHE	A	343	-0.395	24.210	-32.232	1.00	59.42	H
ATOM	4734	HZ	PHE	A	343	-1.677	26.038	-33.260	1.00	48.89	H
ATOM	4735	N	LYS	A	344	-6.677	23.636	-28.578	1.00	78.56	N
ATOM	4736	CA	LYS	A	344	-7.730	24.645	-28.510	1.00	83.34	C
ATOM	4737	C	LYS	A	344	-7.545	25.549	-27.298	1.00	86.07	C
ATOM	4738	O	LYS	A	344	-7.657	26.776	-27.401	1.00	93.20	O
ATOM	4739	CB	LYS	A	344	-9.096	23.959	-28.461	1.00	84.70	C
ATOM	4740	CG	LYS	A	344	-10.252	24.779	-29.006	1.00	88.19	C
ATOM	4741	CD	LYS	A	344	-11.568	24.025	-28.864	1.00	91.41	C
ATOM	4742	CE	LYS	A	344	-11.520	22.677	-29.569	1.00	91.48	C
ATOM	4743	NZ	LYS	A	344	-12.789	21.914	-29.394	1.00	95.26	N1+
ATOM	4744	H	LYS	A	344	-6.961	22.665	-28.603	1.00	78.56	H
ATOM	4745	HA	LYS	A	344	-7.670	25.257	-29.413	1.00	83.34	H
ATOM	4746	HB2	LYS	A	344	-9.032	23.205	-29.219	1.00	84.70	H
ATOM	4747	HB3	LYS	A	344	-9.304	23.480	-27.502	1.00	84.70	H
ATOM	4748	HG2	LYS	A	344	-10.321	25.723	-28.465	1.00	88.19	H
ATOM	4749	HG3	LYS	A	344	-10.079	25.040	-30.050	1.00	88.19	H
ATOM	4750	HD2	LYS	A	344	-11.798	23.887	-27.807	1.00	91.41	H
ATOM	4751	HD3	LYS	A	344	-12.375	24.630	-29.279	1.00	91.41	H
ATOM	4752	HE2	LYS	A	344	-11.347	22.832	-30.626	1.00	91.48	H
ATOM	4753	HE3	LYS	A	344	-10.743	22.018	-29.185	1.00	91.48	H
ATOM	4754	HZ1	LYS	A	344	-13.563	22.448	-29.760	1.00	95.26	H
ATOM	4755	HZ2	LYS	A	344	-12.715	21.035	-29.887	1.00	95.26	H

ATOM 4756 HZ3 LYS A 344 -12.933 21.724 -28.412 1.00 95.26 H
ATOM 4757 N ARG A 345 -7.240 24.955 -26.141 1.00 93.57 N
ATOM 4758 CA ARG A 345 -7.148 25.717 -24.899 1.00 93.28 C
ATOM 4759 C ARG A 345 -6.035 26.755 -24.955 1.00 98.22 C
ATOM 4760 O ARG A 345 -6.197 27.877 -24.459 1.00 99.54 O
ATOM 4761 CB ARG A 345 -6.925 24.766 -23.724 1.00 92.68 C
ATOM 4762 CG ARG A 345 -8.018 23.723 -23.555 1.00 94.35 C
ATOM 4763 CD ARG A 345 -7.499 22.444 -22.900 1.00 96.66 C
ATOM 4764 NE ARG A 345 -6.785 22.694 -21.651 1.00104.45 N
ATOM 4765 CZ ARG A 345 -5.473 22.546 -21.496 1.00104.25 C
ATOM 4766 NH1 ARG A 345 -4.723 22.142 -22.512 1.00 98.59 N1+
ATOM 4767 NH2 ARG A 345 -4.910 22.797 -20.321 1.00112.71 N1+
ATOM 4768 H ARG A 345 -7.114 23.954 -26.091 1.00 93.57 H
ATOM 4769 HA ARG A 345 -8.094 26.241 -24.745 1.00 93.28 H
ATOM 4770 HB2 ARG A 345 -5.973 24.259 -23.890 1.00 92.68 H
ATOM 4771 HB3 ARG A 345 -6.810 25.322 -22.792 1.00 92.68 H
ATOM 4772 HG2 ARG A 345 -8.925 24.115 -23.093 1.00 94.35 H
ATOM 4773 HG3 ARG A 345 -8.323 23.398 -24.547 1.00 94.35 H
ATOM 4774 HD2 ARG A 345 -8.400 21.971 -22.509 1.00 96.66 H
ATOM 4775 HD3 ARG A 345 -7.105 21.685 -23.552 1.00 96.66 H
ATOM 4776 HE ARG A 345 -7.340 23.021 -20.873 1.00104.45 H
ATOM 4777 HH11 ARG A 345 -3.725 22.037 -22.408 1.00 98.59 H
ATOM 4778 HH12 ARG A 345 -5.153 21.911 -23.396 1.00 98.59 H
ATOM 4779 HH21 ARG A 345 -5.476 23.098 -19.541 1.00112.71 H
ATOM 4780 HH22 ARG A 345 -3.914 22.691 -20.194 1.00112.71 H
ATOM 4781 N CYS A 346 -4.898 26.402 -25.555 1.00103.09 N
ATOM 4782 CA CYS A 346 -3.748 27.298 -25.548 1.00113.60 C
ATOM 4783 C CYS A 346 -3.944 28.511 -26.448 1.00119.03 C
ATOM 4784 O CYS A 346 -3.238 29.509 -26.266 1.00122.36 O
ATOM 4785 CB CYS A 346 -2.494 26.538 -25.975 1.00113.30 C
ATOM 4786 SG CYS A 346 -2.175 25.035 -25.028 1.00115.03 S
ATOM 4787 H CYS A 346 -4.791 25.492 -25.981 1.00103.09 H
ATOM 4788 HA CYS A 346 -3.588 27.658 -24.529 1.00113.60 H
ATOM 4789 HB2 CYS A 346 -2.570 26.247 -27.024 1.00113.30 H
ATOM 4790 HB3 CYS A 346 -1.615 27.179 -25.896 1.00113.30 H
ATOM 4791 HG CYS A 346 -3.207 24.336 -25.511 1.00115.03 H
ATOM 4792 N PHE A 347 -4.909 28.453 -27.369 1.00118.10 N
ATOM 4793 CA PHE A 347 -5.073 29.387 -28.498 1.00119.97 C
ATOM 4794 C PHE A 347 -3.869 30.297 -28.764 1.00125.27 C
ATOM 4795 O PHE A 347 -3.939 31.516 -28.608 1.00130.00 O

ATOM   4796 CB PHE A 347   -6.346 30.243 -28.334 1.00118.65   C
ATOM   4797 CG PHE A 347   -6.597 30.750 -26.936 1.00121.96   C
ATOM   4798 CD1 PHE A 347   -7.522 30.125 -26.116 1.00121.92   C
ATOM   4799 CD2 PHE A 347   -5.950 31.880 -26.461 1.00123.35   C
ATOM   4800 CE1 PHE A 347   -7.771 30.596 -24.840 1.00124.65   C
ATOM   4801 CE2 PHE A 347   -6.193 32.354 -25.187 1.00124.88   C
ATOM   4802 CZ PHE A 347   -7.104 31.712 -24.377 1.00127.32   C
ATOM   4803 H   PHE A 347   -5.571 27.689 -27.335 1.00118.10   H
ATOM   4804 HA PHE A 347   -5.189 28.774 -29.393 1.00119.97   H
ATOM   4805 HB2 PHE A 347   -6.404 31.068 -29.047 1.00118.65   H
ATOM   4806 HB3 PHE A 347   -7.196 29.611 -28.597 1.00118.65   H
ATOM   4807 HD1 PHE A 347   -8.051 29.250 -26.463 1.00121.92   H
ATOM   4808 HD2 PHE A 347   -5.287 32.468 -27.066 1.00123.35   H
ATOM   4809 HE1 PHE A 347   -8.486 30.090 -24.208 1.00124.65   H
ATOM   4810 HE2 PHE A 347   -5.684 33.239 -24.833 1.00124.88   H
ATOM   4811 HZ PHE A 347   -7.298 32.083 -23.382 1.00127.32   H
ATOM   4812 HXT PHE A 347   -2.952 29.794 -29.071 1.00125.27   H
TER   4813   PHE A 347
HETATM 4814 C1 UNK   0   -1.077 10.232 -59.115 1.00 0.00   C
HETATM 4815 N1 UNK   0   -0.978 15.202 -60.067 1.00 0.00   N
HETATM 4816 O1 UNK   0   -0.884   8.963 -59.578 1.00 0.00   O
HETATM 4817 C2 UNK   0   -0.027 11.137 -59.196 1.00 0.00   C
HETATM 4818 N2 UNK   0   -1.091 16.715 -58.408 1.00 0.00   N
HETATM 4819 O2 UNK   0   0.634 20.326 -61.255 1.00 0.00   O
HETATM 4820 C3 UNK   0   -0.207 12.445 -58.760 1.00 0.00   C
HETATM 4821 N3 UNK   0   0.999 16.956 -55.892 1.00 0.00   N1+
HETATM 4822 C4 UNK   0   -1.450 12.850 -58.264 1.00 0.00   C
HETATM 4823 C5 UNK   0   -1.657 14.291 -57.807 1.00 0.00   C
HETATM 4824 C6 UNK   0   -1.247 15.393 -58.795 1.00 0.00   C
HETATM 4825 C7 UNK   0   -0.558 16.419 -60.530 1.00 0.00   C
HETATM 4826 C8 UNK   0   -0.090 16.770 -61.804 1.00 0.00   C
HETATM 4827 C9 UNK   0   0.292 18.100 -62.022 1.00 0.00   C
HETATM 4828 C10 UNK   0   0.216 19.053 -61.010 1.00 0.00   C
HETATM 4829 C11 UNK   0   -0.255 18.739 -59.741 1.00 0.00   C
HETATM 4830 C12 UNK   0   -0.628 17.405 -59.516 1.00 0.00   C
HETATM 4831 C13 UNK   0   -1.326 17.283 -57.083 1.00 0.00   C
HETATM 4832 C14 UNK   0   -0.059 17.927 -56.477 1.00 0.00   C
HETATM 4833 C15 UNK   0   0.543 16.247 -54.598 1.00 0.00   C
HETATM 4834 C16 UNK   0   0.166 17.252 -53.496 1.00 0.00   C
HETATM 4835 C17 UNK   0   1.658 16.024 -56.943 1.00 0.00   C

```
HETATM 4836  C18 UNK     0       2.904  15.305 -56.399  1.00  0.00           C
HETATM 4837  C19 UNK     0      -2.495  11.927 -58.163  1.00  0.00           C
HETATM 4838  C20 UNK     0      -2.309  10.614 -58.596  1.00  0.00           C
HETATM 4839  H1  UNK     0      -1.530   8.320 -59.276  1.00  0.00           H
HETATM 4840  H2  UNK     0       0.926  10.823 -59.596  1.00  0.00           H
HETATM 4841  H3  UNK     0       0.614  13.145 -58.829  1.00  0.00           H
HETATM 4842  H4  UNK     0      -1.068  14.378 -56.895  1.00  0.00           H
HETATM 4843  H5  UNK     0      -2.682  14.435 -57.474  1.00  0.00           H
HETATM 4844  H6  UNK     0      -0.010  16.028 -62.584  1.00  0.00           H
HETATM 4845  H7  UNK     0       0.672  18.403 -62.988  1.00  0.00           H
HETATM 4846  H8  UNK     0      -0.296  19.493 -58.972  1.00  0.00           H
HETATM 4847  H9  UNK     0      -1.761  16.544 -56.412  1.00  0.00           H
HETATM 4848  H10 UNK     0      -2.088  18.053 -57.208  1.00  0.00           H
HETATM 4849  H11 UNK     0      -0.346  18.568 -55.654  1.00  0.00           H
HETATM 4850  H12 UNK     0       0.507  18.545 -57.173  1.00  0.00           H
HETATM 4851  H13 UNK     0       1.415  15.681 -54.283  1.00  0.00           H
HETATM 4852  H14 UNK     0      -0.263  15.567 -54.868  1.00  0.00           H
HETATM 4853  H15 UNK     0       0.102  16.752 -52.534  1.00  0.00           H
HETATM 4854  H16 UNK     0      -0.796  17.733 -53.675  1.00  0.00           H
HETATM 4855  H17 UNK     0       0.921  18.031 -53.392  1.00  0.00           H
HETATM 4856  H18 UNK     0       0.908  15.309 -57.263  1.00  0.00           H
HETATM 4857  H19 UNK     0       1.920  16.680 -57.775  1.00  0.00           H
HETATM 4858  H20 UNK     0       3.446  14.829 -57.215  1.00  0.00           H
HETATM 4859  H21 UNK     0       3.598  15.987 -55.912  1.00  0.00           H
HETATM 4860  H22 UNK     0       2.651  14.516 -55.691  1.00  0.00           H
HETATM 4861  H23 UNK     0      -3.450  12.230 -57.760  1.00  0.00           H
HETATM 4862  H24 UNK     0      -3.124   9.910 -58.546  1.00  0.00           H
HETATM 4863  H25 UNK     0       0.653  20.874 -60.453  1.00  0.00           H
HETATM 4864  H26 UNK     0       1.758  17.567 -55.607  1.00  0.00           H
CONECT 4814 4816
CONECT 4814 4817
CONECT 4814 4817
CONECT 4814 4817
CONECT 4814 4817
CONECT 4814 4838
CONECT 4814 4838
CONECT 4814 4838
CONECT 4814 4838
CONECT 4815 4824
CONECT 4815 4824
```

CONECT 4815 4824
CONECT 4815 4824
CONECT 4815 4825
CONECT 4815 4825
CONECT 4815 4825
CONECT 4815 4825
CONECT 4816 4814 4839
CONECT 4817 4814
CONECT 4817 4814
CONECT 4817 4814
CONECT 4817 4814
CONECT 4817 4820
CONECT 4817 4820
CONECT 4817 4820
CONECT 4817 4820
CONECT 4817 4840
CONECT 4818 4824
CONECT 4818 4824
CONECT 4818 4824
CONECT 4818 4824
CONECT 4818 4830
CONECT 4818 4830
CONECT 4818 4830
CONECT 4818 4830
CONECT 4818 4831
CONECT 4819 4828 4863
CONECT 4820 4817
CONECT 4820 4817
CONECT 4820 4817
CONECT 4820 4817
CONECT 4820 4822
CONECT 4820 4822
CONECT 4820 4822
CONECT 4820 4822
CONECT 4820 4841
CONECT 4821 4832 4833 4835 4864
CONECT 4822 4820
CONECT 4822 4820
CONECT 4822 4820
CONECT 4822 4820

CONECT 4822 4823
CONECT 4822 4837
CONECT 4822 4837
CONECT 4822 4837
CONECT 4822 4837
CONECT 4823 4822 4824 4842 4843
CONECT 4824 4815
CONECT 4824 4815
CONECT 4824 4815
CONECT 4824 4815
CONECT 4824 4818
CONECT 4824 4818
CONECT 4824 4818
CONECT 4824 4818
CONECT 4824 4823
CONECT 4825 4815
CONECT 4825 4815
CONECT 4825 4815
CONECT 4825 4815
CONECT 4825 4826
CONECT 4825 4826
CONECT 4825 4826
CONECT 4825 4826
CONECT 4825 4830
CONECT 4825 4830
CONECT 4825 4830
CONECT 4825 4830
CONECT 4826 4825
CONECT 4826 4825
CONECT 4826 4825
CONECT 4826 4825
CONECT 4826 4827
CONECT 4826 4827
CONECT 4826 4827
CONECT 4826 4827
CONECT 4826 4844
CONECT 4827 4826
CONECT 4827 4826
CONECT 4827 4826
CONECT 4827 4826

CONECT 4827 4828
CONECT 4827 4828
CONECT 4827 4828
CONECT 4827 4828
CONECT 4827 4845
CONECT 4828 4819
CONECT 4828 4827
CONECT 4828 4827
CONECT 4828 4827
CONECT 4828 4827
CONECT 4828 4829
CONECT 4828 4829
CONECT 4828 4829
CONECT 4828 4829
CONECT 4829 4828
CONECT 4829 4828
CONECT 4829 4828
CONECT 4829 4828
CONECT 4829 4830
CONECT 4829 4830
CONECT 4829 4830
CONECT 4829 4830
CONECT 4829 4846
CONECT 4830 4818
CONECT 4830 4818
CONECT 4830 4818
CONECT 4830 4818
CONECT 4830 4825
CONECT 4830 4825
CONECT 4830 4825
CONECT 4830 4825
CONECT 4830 4829
CONECT 4830 4829
CONECT 4830 4829
CONECT 4830 4829
CONECT 4831 4818 4832 4847 4848
CONECT 4832 4821 4831 4849 4850
CONECT 4833 4821 4834 4851 4852
CONECT 4834 4833 4853 4854 4855
CONECT 4835 4821 4836 4856 4857

CONECT 4836 4835 4858 4859 4860
CONECT 4837 4822
CONECT 4837 4822
CONECT 4837 4822
CONECT 4837 4822
CONECT 4837 4838
CONECT 4837 4838
CONECT 4837 4838
CONECT 4837 4838
CONECT 4837 4861
CONECT 4838 4814
CONECT 4838 4814
CONECT 4838 4814
CONECT 4838 4814
CONECT 4838 4837
CONECT 4838 4837
CONECT 4838 4837
CONECT 4838 4837
CONECT 4838 4862
CONECT 4839 4816
CONECT 4840 4817
CONECT 4841 4820
CONECT 4842 4823
CONECT 4843 4823
CONECT 4844 4826
CONECT 4845 4827
CONECT 4846 4829
CONECT 4847 4831
CONECT 4848 4831
CONECT 4849 4832
CONECT 4850 4832
CONECT 4851 4833
CONECT 4852 4833
CONECT 4853 4834
CONECT 4854 4834
CONECT 4855 4834
CONECT 4856 4835
CONECT 4857 4835
CONECT 4858 4836
CONECT 4859 4836

CONECT 4860 4836
CONECT 4861 4837
CONECT 4862 4838
CONECT 4863 4819
CONECT 4864 4821
END
